# Development of a CD8+ T cell associated signature for predicting the prognosis and immunological characteristics of gastric cancer by integrating single-cell and bulk RNA-sequencing

**DOI:** 10.1038/s41598-024-54273-9

**Published:** 2024-02-24

**Authors:** Jianxin Li, Ting Han, Xin Wang, Yinchun Wang, Rui Yang, Qingqiang Yang

**Affiliations:** Department of General Surgery (Gastrointestinal Surgery), The Affiliated Hospital of Southwest Medical University, 25 Taiping Street, Luzhou, Sichuan 646000 People’s Republic of China

**Keywords:** Gastric cancer, Immune, Single-cell RNA-sequencing, Prognosis, Immunotherapy, Cancer models, Gastrointestinal cancer, Computational biology and bioinformatics, Biomarkers

## Abstract

The universally poor clinical outcome makes gastric cancer (GC) still a significant public health threat, the main goal of our research is to develop a prognostic signature that can forecast the outcomes and immunological characteristics of GC via integrating single-cell and bulk RNA-sequencing. The CD8+ T cell feature genes were screened out by exploring single-cell RNA-sequencing (scRNA-seq) profiles retrieved from the TISCH2 database. Then, Cox and LASSO regressions were exploited for constructing a prognostic model in TCGA cohort based on these CD8+ T cell feature genes. Survival analysis was conducted to investigate the predictive capability of the signature for the clinical outcome of GC patients in TCGA and GEO cohorts. Additionally, we further examined the correlations between the risk signature and tumor immunotherapeutic response from the perspectives of immune infiltration, tumor mutation burden (TMB), immune checkpoint biomarker (ICB) expression, tumor microenvironment (TME), microsatellite instability (MSI), TIDE, and TCIA scores. In total, 703 CD8+ T cell feature genes were identified, eight of which were selected for constructing a prognostic signature. GC patients who possess high-risk score had significantly poorer survival outcomes than those who possess low-risk score in TCGA and GEO cohorts. Immune infiltration analysis proved that the risk score was negatively connected with the infiltration abundance of CD8+ T cells. Then, our findings demonstrated that GC patients in the high-risk subgroup possess a higher proportion of MSI-L/MSS, lower immune checkpoint biomarker expression, lower TMB, higher TIDE scores and lower TCIA scores compared to those in the low-risk subgroup. What’s more, immunotherapy cohort analysis confirmed that patients who possess high-risk score are not sensitive to anti-cancer immunotherapy. Our study developed a reliable prognostic signature for GC that was significantly correlated with the immune landscape and immunotherapeutic responsiveness. The risk signature may guide clinicians to adopt more accurate and personalized treatment strategies for GC patients.

## Introduction

Malignant tumors are the most common cause of disease-related deaths worldwide. According to the statistics, over 19 million newly diagnosed malignant tumor cases and 10 million disease-associated deaths around the world were reported in 2020^1^. Among them, gastric cancer (GC) is responsible for 5.6% of incidence and 7.7% of mortality, making it a great threat to public health. Within a few decades, studies focused on comprehending the etiology and treatment of GC have gained unprecedented progress, and early-stage patients can be cured using comprehensive therapy based on operation. However, due to the deficiency of effective diagnostic markers and patients with early stage are generally symptom-free, the majority of GC patients are diagnosed with an aggressive stage on the first visit, and the average 5-year overall survival remains below 30%^2^. Furthermore, resistance to existing treatment modalities worsens the prognosis. Thus, investigating innovative biomarkers capable of accurately predicting GC prognosis and therapy response is of great significance.

The tumor microenvironment (TME) is composed of cellular and non-cellular components. The former primarily consists of stromal cells such as endothelial cells, fibroblasts, and immune cells, and the latter, nucleic acids, cytokines, and growth factors^3^. A growing number of researches have indicated that the dynamic crosstalk between cellular and non-cellular components in TME contributes to cancer progression, and the TME serves as a significant role in cancer initiation, progression, and therapeutic drug resistance^4,5^. Among the immune cells in TME, CD8+ T cells exhibit important anti-cancer activities and have favourable treatment effects on numerous cancers, including GC. CD8+ T cells possess the capability to specifically detect and deracinate cancer cells by secreting effector cytokines such as tumor necrosis factor (TNF) and interferon-γ (IFN-γ), and death-inducing granules such as granzymes, perforin, cathepsin C and granulysin^6,7^. CD8+ T cell dysfunction and exhaustion have been recognized as the most important immune characteristics during tumorigenesis, and immunotherapy strategies such as immune checkpoint blockade that focuses on reactivating the immunological activity of CD8+ T cells has achieved great success for many solid tumors^8^. Considering the significant role of CD8+ T cells in tumor progression and immunotherapeutic response, developing a CD8+ T cell-associated signature for forecasting the survival outcome and immunological characteristics of GC is of great worth.

**Figure 1:**
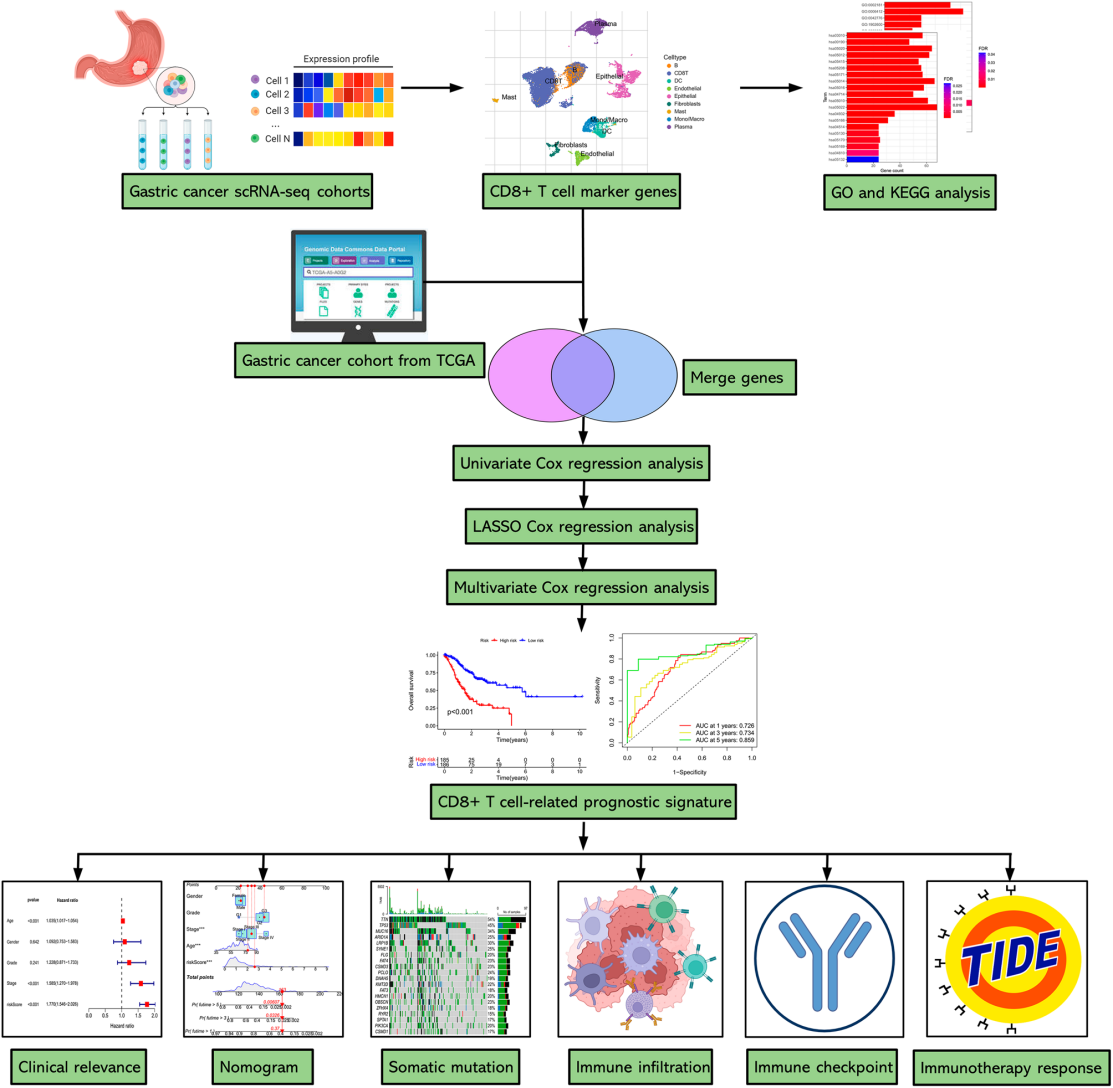
The flowchart for the entire study.

The rapidly developed high-throughput sequencing technology has revolutionized the realm of biology, researchers can access and reanalyze sequencing data in a more detailed insight based on public databases. In recent decades, many researches have launched to develop prognostic signatures for forecasting the clinical outcome and immunological landscapes of diverse cancers based on sequencing data. However, the TME is a complex environment with high heterogeneity, conventional transcriptomic investigation may ignore the biologically relevant differences between distinct cells^9^. Compared to traditional RNA sequencing, the single-cell RNA-sequencing (scRNA-seq) technology enables researchers to determine the heterogenicity of tumor and stromal cells from the perspective of cellular level, and discriminate the gene expression characteristics of distinct cell types, thereby identifying feature genes for each cell^10^. As far as we know, there were no studies focused on constructing prognostic signatures for GC from the perspective of CD8+ T cell marker genes. In this study, we identified CD8+ T cell feature genes by exploring scRNA-seq profiles and generated a novel risk signature for forecasting the clinical outcome and immunotherapeutic responsiveness of GC patients. The flowchart for the entire study was displayed in Fig. 1.

## Materials and methods

### Data acquisition and processing

Tumor Immune Single-cell Hub 2 (TISCH2, http://tisch.comp-genomics.org/home/) is an online platform that supplies detailed scRNA-seq information, enabling researchers to investigate the characteristics of TME at the cellular level across multiple types of malignant tumor^11^. In this study, detailed cell-type annotation files of GSE134520 (including 41,554 GC cells) ^12^ and GSE167297 (including 22,464 GC cells)^13^ were retrieved from the TISCH2 database. Besides, the bulk RNA-sequencing (RNA-seq) dataset, somatic mutation information, and clinicopathological paraments of GC were retrieved from The Cancer Genome Atlas (TCGA, https://portal.gdc.cancer.gov/) project. GSE62254 cohort with 300 GC samples and GSE15459 with 192 samples were downloaded from the Gene Expression Omnibus (GEO, https://www.ncbi.nlm.nih.gov/geo/) project as validation cohorts^14,15^. Moreover, the IMvigor210 cohort including RNA-sequencing data and detailed clinical information of urothelial cancer patients receiving immunotherapy was utilized to determine whether the risk signature can predict the effectiveness of immunotherapy in human cancer^16^.

### Identification of feature genes associated with CD8+ T cells in GC.

The scRNA-seq data re-analysis and subsequent differential analysis between CD8+ T cells and other cell types were processed by the TISCH2 project. Genes showing significantly different expression patterns in CD8+ T cells were identified as CD8+ T cell feature genes (adjusted p-value < 0.05). Subsequently, we applied the Database for Annotation, Visualization and Integrated Discovery (DAVID, https://david.ncifcrf.gov/) database for performing Gene Ontology (GO) and Kyoto Encyclopedia of Genes and Genomes (KEGG) enrichment analysis on the marker genes of CD8 + T cell clusters to determine their underlying molecular functions and potential mechanisms^17^.

### Development of the survival related risk signature

We first utilized univariate Cox regression to screen out survival related CD8+ T cell marker genes in TCGA cohort. Subsequently, we conducted least absolute shrinkage and selection operator (LASSO) regression to minimize the risk of overfitting and compress the number of variables. Afterwards, multivariate Cox regression was conducted to select optimal survival-related variables and develop a signature with the following formula:${\text{riskScore}} = \mathop \sum \limits_{k = 1}^{n} \left[ {Exp\left( {genes} \right)* coef\left( {genes} \right)} \right]$. Where *Exp* indicates the gene expression level and *coef* indicates the coefficients of the gene calculated by multivariate Cox regression. GC patients in TCGA cohort were classified into low- and high-risk subgroups according to the median risk score. Then, we performed Kaplan–Meier survival and time-dependent receiver operating characteristic (ROC) analyses to investigate the predictive efficiency of the signature by “survminer” and “survivalROC” R packages.

### Clinical relevance analysis

The differences in clinicopathological parameters (age, gender, grade, and tumor stage) between different subgroups were analyzed via chi-square test. Besides, we conducted univariate and multivariate Cox regression analyses to clarify whether the signature has the ability to forecast the survival outcome of GC as an independent prognostic factor. Similarly, we utilized ROC analysis to assess the predictive efficiency of different indexes in predicting patients’ outcomes.

### Independent validation of the risk signature

We utilized the same formula and coefficients to determine the generalizability of the signature in independent validation cohorts GSE62254 and GSE15459. Similarly, the Kaplan–Meier curve of the validation cohorts was plotted to investigate the predictive capability of the signature in the validation cohort, and univariate and multivariate Cox regression analyses were utilized for the independent survival analysis.

### Generation of the predictive nomogram

A nomogram comprised of risk score and other clinical parameters was generated for predicting 1-, 3- and 5-year survival rates of GC patients. The ROC and calibration plots were conducted to evaluate the consistency between actual and predicted outcomes. Moreover, we performed decision curve analysis (DCA) to determine the net benefits of the nomogram and other parameters.

### Somatic mutation analysis

Tumor mutation burden (TMB) is defined as total number of mutations present in a tumor specimen, and multiple lines of research have indicated that patients with higher TMB forecast better clinical outcomes after immunotherapy. Therefore, the Food and Drug Administration (FDA) has recently granted the clinical application of anti-PD-1 agent pembrolizumab as an alternative immunotherapy strategy for solid tumors with TMB > 10 mutations/Mb ^18^. In this study, we evaluated the TMB of each sample and assessed its relationship with the risk score. Besides, the genetic mutation landscapes in low- and high-risk subgroups were visualized by “Maftools” package.

### Tumor microenvironment and infiltrated immune cell analysis

To further investigate the association between the risk signature and TME, “CIBERSORT”, “xCELL”, “EPIC”, “TIMER”, “MCP-counter”, and “quanTIseq” algorithms were applied to quantify the density of diverse tumor-infiltrated immune cells (TIICs) for each patient^19–24^. Then, the association between the TIIC density and risk score was evaluated using Spearman’s correlation test and Wilcoxon test. Additionally, the ESTIMATE algorithm is a new approach to estimating the proportion of stromal and immune cells in TME by performing single-sample gene set enrichment analysis (ssGSEA) on the basis of specific gene expression patterns^25^. In this study, we utilized the Wilcoxon test to analyze the differences in TME scores between the risk subgroups, including stromal score, immune score, and ESTIMATE score.

### Immunophenoscore analysis

The Immunophenoscore (IPS) has been identified as a superior predictive index of sensitivity to immunotherapy, which has been applied to assess the determinants of tumor immunogenicity^26^. The IPS profiles of GC cohort were obtained from The Cancer Immunome Atlas (TCIA, https://tcia.at/). Then, the difference in IPS between the risk subgroups was assessed by the Wilcoxon test.

### Tumor immune dysfunction and exclusion analysis

Tumor Immune Dysfunction and Exclusion (TIDE, http://tide.dfci.harvard.edu/) is a computational algorithm to model two primary mechanisms of tumor immune escape: prevention of T cell infiltration and induction of T cell exhaustion in TME^27^. Patients who possess high TIDE scores are more likely to evade anti-cancer immunity, thus achieving unsatisfactory immunotherapeutic efficacy. We thus calculated the TIDE scores of each GC patient by applying the TIDE algorithm and determined the relationship between the risk score and the effectiveness of immunotherapy.

### Microsatellite instability analysis

Microsatellite instability (MSI) is a molecular index of deficient DNA mismatch repair (dMMR), MSI tumors exhibit elevated tumor mutation loads and neoantigens, which stimulate the anti-cancer immunity of the host and thereby achieving higher immunotherapeutic efficacy^28^. Thus, the FDA has recently granted pembrolizumab as an essential drug for the treatment of microsatellite instability-high (MSI-H) tumors, including GC^29^. In this research, we investigated the relationship between the risk score and the MSI status.

### Immune checkpoint biomarker expression analysis

Immune checkpoints are co-inhibitory molecules mainly expressed on the membrane of T cells to restrain the T cell-induced host immunological activity, thereby inducing T cell exhaustion and tumor tolerance^30^. Immunotherapeutic agents targeting ICBs, such as PD-L1, PD1 and CTLA-4, have obtained unprecedented progress in anti-tumor therapy in the last decade. In this study, we further determined the relationship between the risk score and immune landscapes of GC by comparing the differences in ICBs’ expression between these two risk subgroups.

### Exploring the predictive capability of the risk signature in immunotherapy cohort

The RNA-sequencing profile and clinical parameters of urothelial cancer patients being treated with anti-PD-L1 agent were retrieved from the immunotherapeutic cohort IMvigor210. According to immunotherapeutic efficacy, patients in the immunotherapy cohort were distributed into responder and non-responder subgroups. The former includes complete response (CR) and partial response (PR), and the latter, stable disease (SD) and progressive disease (PD). The differences in the risk scores between different subgroups were determined by the Wilcoxon test.

### Statistical analysis

Statistical analysis was conducted using the R software (v4.1.0, https://www.r-project.org/), and the data packages utilized for statistical analysis within R were as described above. P-value < 0.05 was considered statistical significance.

## Results

### Identification of feature genes related to CD8+ T cell in GC

**Figure 2:**
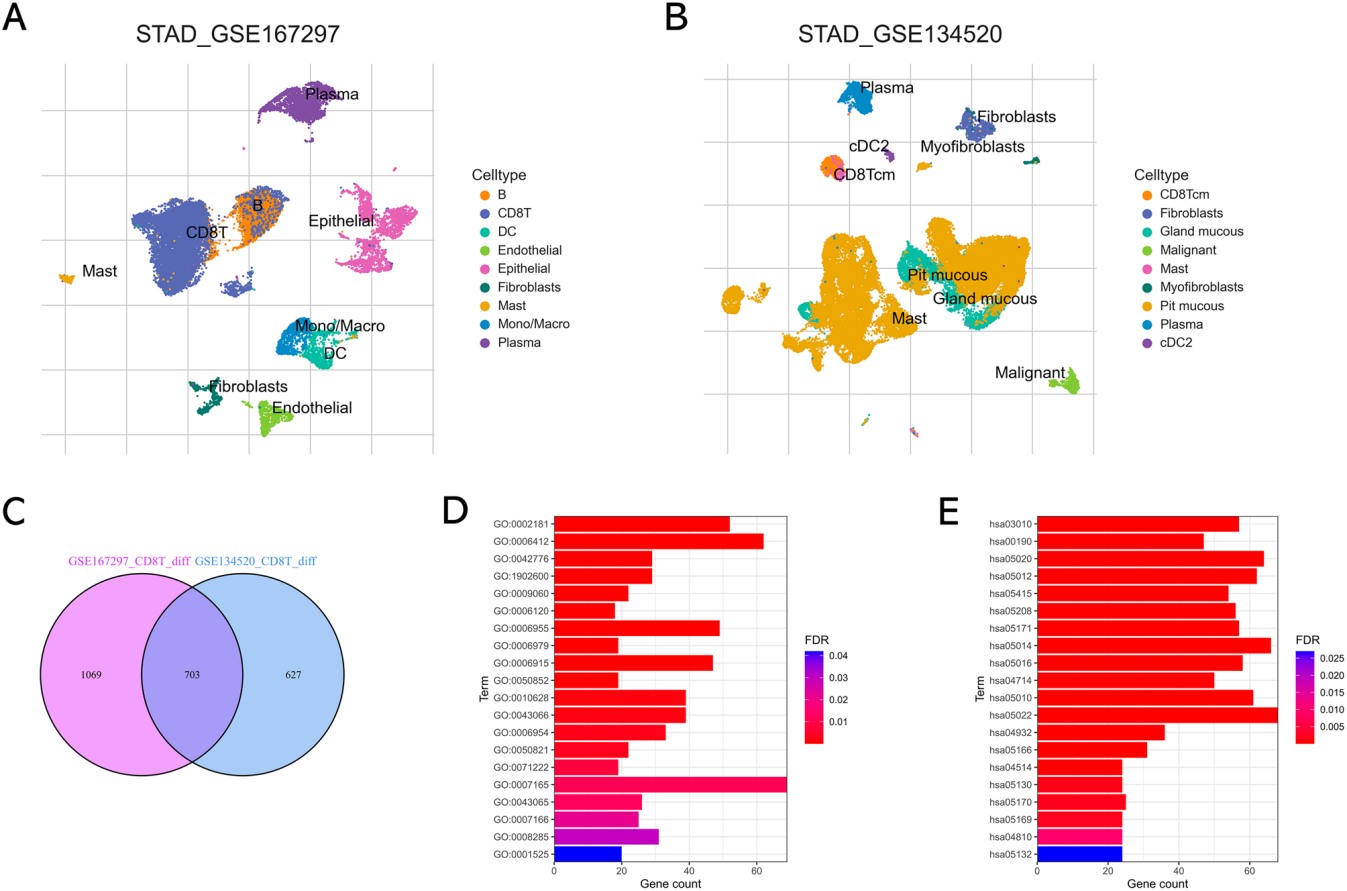
Identification of CD8+ T cell marker genes. (**A**) Cell clusters identified with marker genes for each cell type generated by TISCH2 in GSE167297. (**B**) Cell clusters identified with marker genes for each cell type generated by TISCH2 in GSE134520. (**C**) The Venn diagram indicated CD8+ T cell marker genes between GSE167297 and GSE134520 datasets. (**D**) GO analysis of CD8+ T cell marker genes. (**E**) KEGG analysis of CD8+ T cell marker genes.

The scRNA-seq datasets GSE167297 and GSE134520 were analyzed using the TISCH2 platform. As shown in Fig. 2A, nine cell clusters were annotated in GSE167297, and the significant differential feature genes of each cell cluster were displayed in Supplementary Table 1. In terms of GSE134520, a total of nine cell clusters were annotated (Fig. 2B) and the significant differential marker genes were listed in Supplementary Table 2. Ultimately, 703 candidate CD8+ T cell feature genes were screened for subsequent analysis after intersecting the marker genes obtained from GSE167297 and GSE134520 by Venn (http://bioinformatics.psb.ugent.be/webtools/Venn/) platform (Fig. 2C). Then, GO annotation analysis showed that CD8+ T cell feature genes are primarily enriched in signal transduction (GO0007165), translation (GO0006412), immune response (GO0006955), apoptotic process (GO0006915) and inflammatory response (GO0006954) (Fig. 2D), while KEGG enrichment analysis found that CD8+ T cell feature genes are primarily enriched in Pathways of neurodegeneration (hsa05022), Amyotrophic lateral sclerosis (hsa05014), Prion disease (hsa05020), and Parkinson disease (hsa05012) (Fig. 2E).

### Development of the CD8+ T cell-correlated risk signature

**Figure 3:**
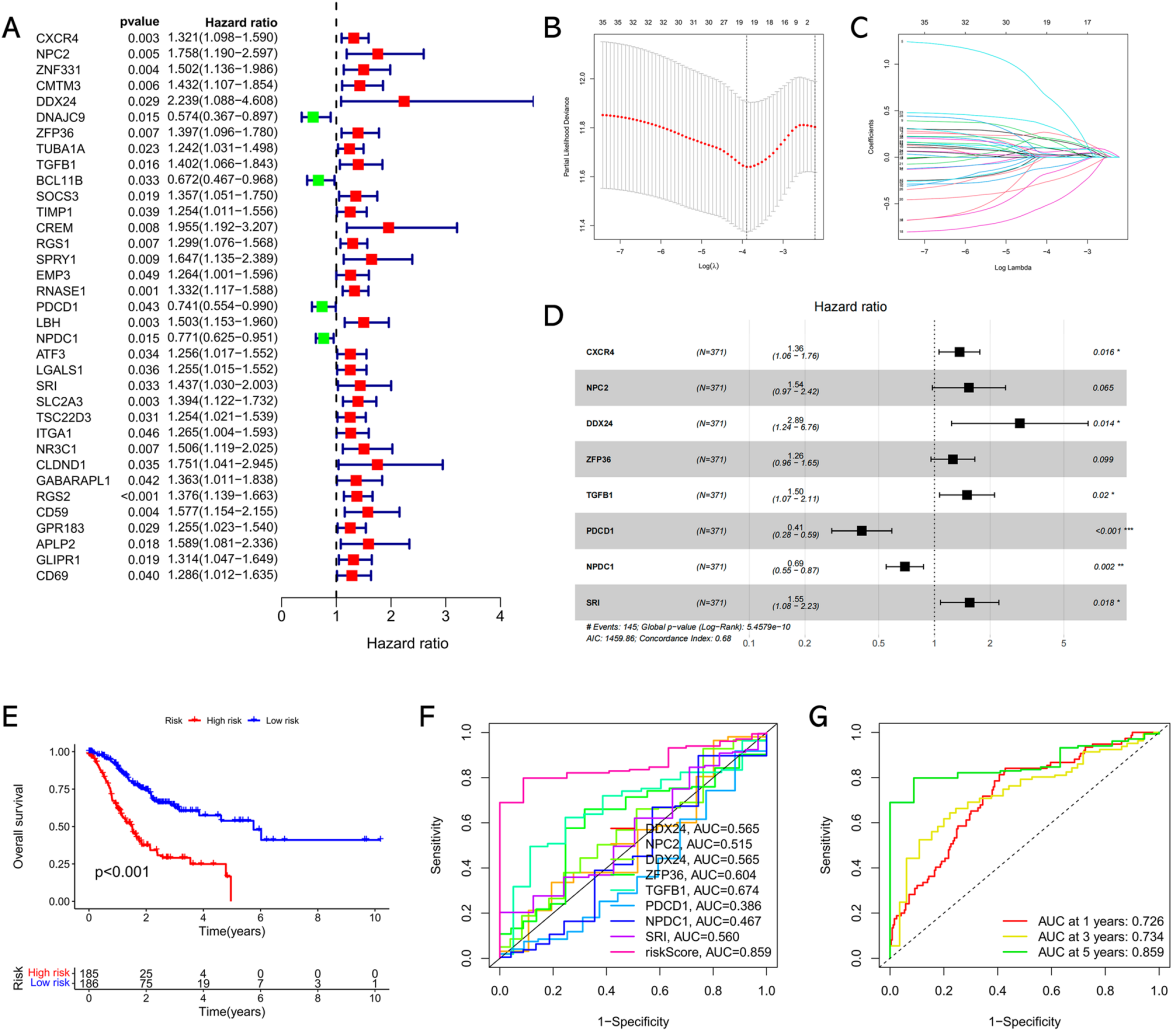
Development of CD8+ T cell-associated risk signature. (**A**) Forest plot of survival related CD8+ T cell marker genes based on univariate Cox regression analysis. (**B**) Plots of the produced coefficient distributions for the logarithmic series for parameter selection (lambda). (**C**) The trajectory of each independent variable with lambda. (**D**) Forest plot of optimal prognostic genes used for the construction of the risk signature based on multivariate Cox regression analysis. (**E**) Kaplan–Meier survival curve of the risk signature in TCGA cohort. (**F**) ROC analysis of survival status for the risk signature and eight single CD8+ T cell marker genes. (**G**) Time-dependent ROC curve of the risk signature in TCGA cohort.

Firstly, a total of 35 CD8+ T cell marker genes that obviously correlated with clinical outcomes of GC patients were identified by univariate Cox regression analysis (Fig. 3A). Then, 19 genes were filtered out via LASSO analysis (Fig. 3B and C), and eight of which were eventually selected for constructing a prognostic signature through multivariate Cox regression analysis (Fig. 3D). The coefficients of each gene are listed in Table 1. The GC patients were assigned into low- and high-risk subgroups according to the risk score of 1.001. Kaplan–Meier curve indicated that patients with low-risk score presented a significantly better survival outcome compared to those with high-risk score (Fig. 3E). Additionally, we performed time-dependent ROC analysis to verify the efficiency of the risk score. As shown in Fig. 3F and G, the predictive capability of the risk score is superior to each single gene, and the area under the ROC curve (AUC) values of the 1-, 3-, and 5-year survival rate were 0.726, 0.734, and 0.859, respectively. Besides, ROC analysis revealing that the risk signature was more accurate than other potential biomarkers such as TMB, MSI, TIDE and IPS scores in predicting prognosis (Supplementary Fig. 1). These findings suggested the significant performance of the CD8+ T cell signature in predicting the clinical outcomes of GC.

**Table 1:** The list of eight prognostic genes.

Gene	Coefficients	HR	HR.95L	HR.95H	p-value
CXCR4	0.31068	1.364353	1.058744	1.758177	0.016344
NPC2	0.429302	1.536185	0.974475	2.421678	0.064512
DDX24	1.062122	2.892502	1.237326	6.761813	0.014227
ZFP36	0.229511	1.257985	0.958099	1.651735	0.098558
TGFB1	0.405287	1.499732	1.065393	2.111143	0.020177
PDCD1	-0.90348	0.405158	0.278703	0.588988	2.21E-06
NPDC1	-0.36774	0.692299	0.549216	0.872658	0.001852
SRI	0.437998	1.549601	1.077719	2.228099	0.018083

HR, hazard ratio.

### Relationships between the risk signature and clinical features

**Figure 4:**
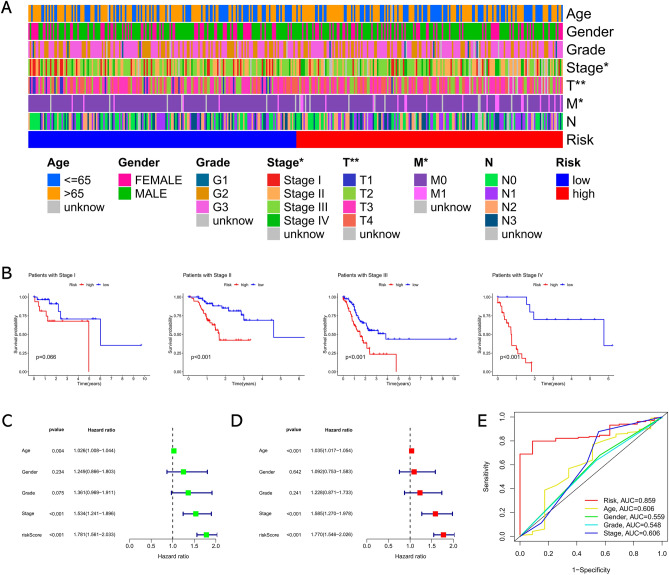
The correlation between the risk signature and clinical parameters. (**A**) Heat maps of clinical parameters between low- and high-risk groups. *P < 0.05; **P < 0.01. (**B**) The subgroup survival analysis according to the tumor stage. (**C**) Univariate Cox analysis of risk score and clinicopathological parameters. (**D**) Multivariate Cox analysis of risk score and clinicopathological parameters. (**E**) Multi-index ROC curve of risk score and clinicopathological parameters.

The relationships between the risk score and clinical features of GC were investigated via chi-square tests. As shown in Fig. 4A, a significant positive relationship between high-risk score and advanced tumor stage was observed. Then, we conducted a subgroup analysis according to tumor stage. Except for Stage I, the risk score showed significant capability in forecasting the survival outcomes of GC patients in each subgroup (Fig. 4B). Subsequently, we evaluated the independent predictive performance of the signature via univariate and multivariate Cox regression analyses and found that the risk score can be used as an independent predictor for GC (Fig. 4C and D). Besides, the multi-variable ROC curve suggested that the predictive capability of the risk score (AUC = 0.859) in forecasting the prognosis of GC patients was superior to other parameters (Fig. 4E).

### External validation confirmed the predictive ability of the risk signature

**Figure 5:**
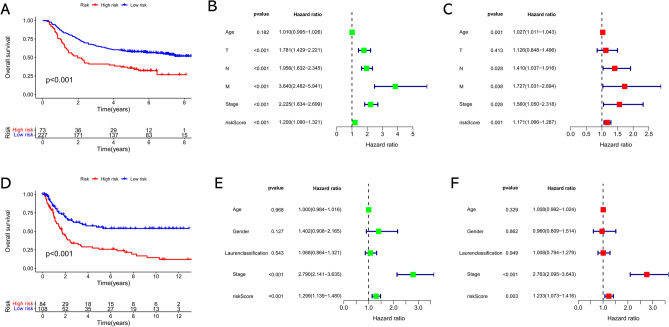
External validation of the risk signature in predicting overall survival of GC based on independent cohorts. (**A**) The Kaplan–Meier survival analysis of the risk signature in the GSE62254 cohort (n = 300). (**B**) The univariate and (**C**) multivariate Cox regression analyses of risk score and clinicopathological parameters in GSE62254 cohort. (**D**) The Kaplan–Meier survival analysis of the risk signature in the GSE15459 cohort (n = 192). (**E**) The univariate and (**F**) multivariate Cox regression analyses of risk score and clinicopathological parameters in GSE15459 cohort.

We estimated the predictive performance of the signature in two external validation cohorts (GSE62254, n = 300; GSE15459, n = 192) to further determine whether the signature can be applied in different populations. As a result, patients who possess high-risk score showed significantly worse survival outcomes compared to those who possess low-risk score in each independent cohort, and the risk score was identified as an independent predictor (Fig. 5). These findings further confirmed the widespread applicability of the risk signature among different populations.

### Development of a nomogram

**Figure 6:**
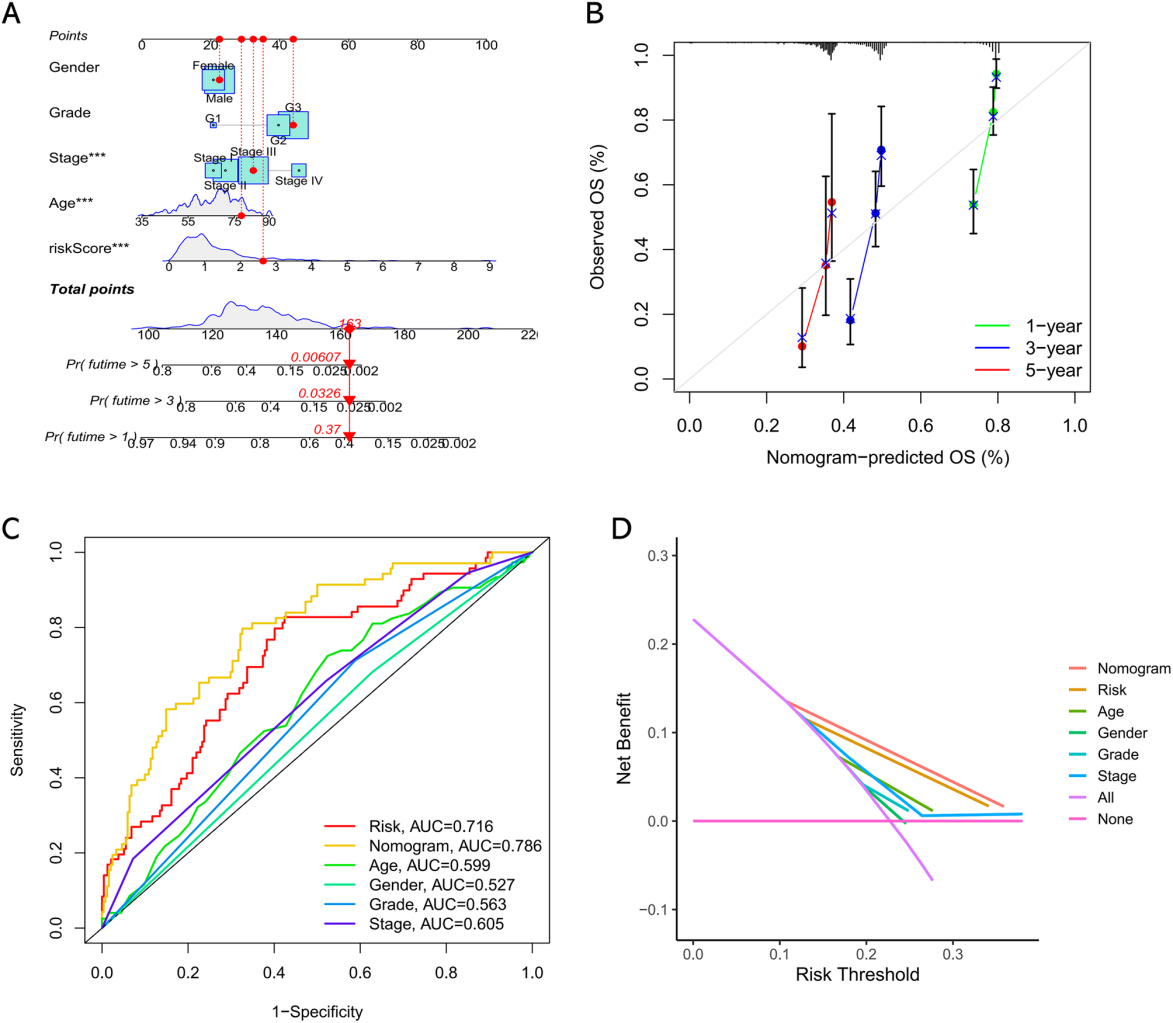
The development of a prognostic nomogram. (**A**) Nomogram model integrating the risk score and clinical parameters was constructed. (**B**) Calibration curve of the nomogram to predict the probability of 1-, 3-, and 5-year survival. (**C**) Multi-index ROC curve of nomogram model and other parameters. (**D**) DCA analysis showing the performance of the nomogram for predicting the 1- 3-, and 5-year survival.

A nomogram of overall survival was developed by incorporating risk score and other prognostic risk factors such as age, gender, grade and tumor stage given by performing the multivariate analysis (Fig. 6A). The calibration curve indicated that the probabilities of overall survival forecasted by the nomogram model was closely matched the actual survival of GC patients (Fig. 6B). Meanwhile, the ROC curve showed that the nomogram model to forecast the prognosis of GC was obviously better than traditional clinical parameters (Fig. 6C). Besides, the DCA curve also showed that the nomogram model had superior prognostic value than other variables (Fig. 6D). Taken together, our data suggested that the nomogram model has a superior clinical benefit for GC patients.

### Somatic mutations in different subgroups

**Figure 7:**
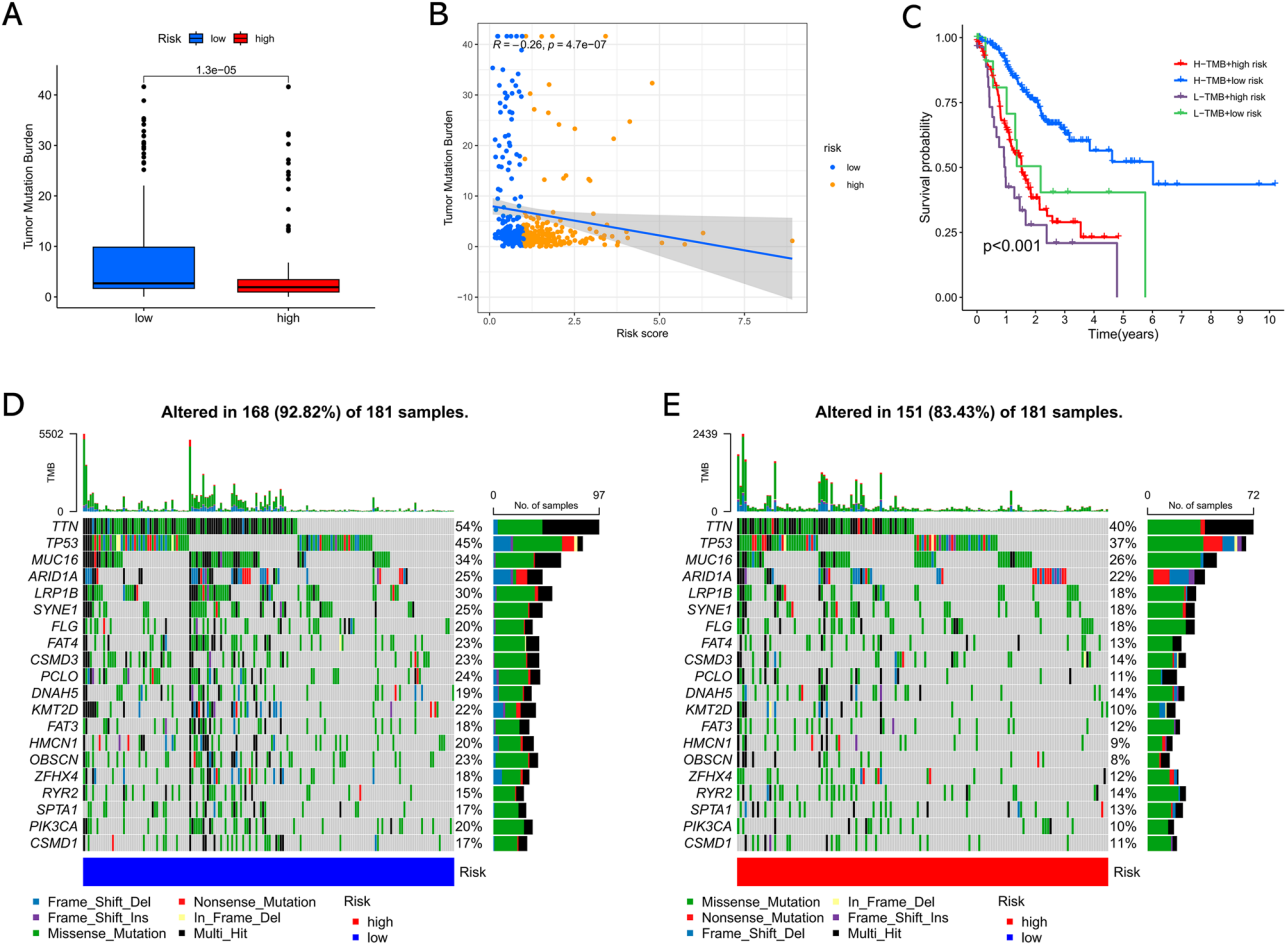
Relevance exploration of risk signature with tumor somatic mutation. (**A**) Difference in TMB between low- and high-risk groups. (**B**) Spearman correlation analysis between risk score and TMB. (**C**) The Kaplan–Meier curve showing overall survival after combining the risk score with TMB. (**D**) Waterfall plots of top 20 mutated genes in the low-risk subgroup. (**E**) Waterfall plots of top 20 mutated genes in the high-risk subgroup.

We explored the somatic mutations to achieve further biological comprehension of the immunological characteristics of the risk subgroups. As shown in Fig. 7A, the TMB was remarkably elevated in the low-risk subgroup compared with the high-risk subgroup. As expected, the Spearman correlation plot also indicated the significant negative association between the risk score and TMB in GC (Fig. 7B). Then, these two factors were taken into account together, patients in the high-TMB+ low-risk score subgroup showed a significantly better clinical outcome compared to those in the other three subgroups (Fig. 7C). The top 20 genes that possess the highest mutation frequencies in each risk subgroup are showed in Fig. 7D and E. Among them, the mutation rates of TTN, TP53, and MUC16 were not only higher than 25% in both subgroups but the most frequent in both subgroups.

### The association between the risk signature and TME

**Figure 8:**
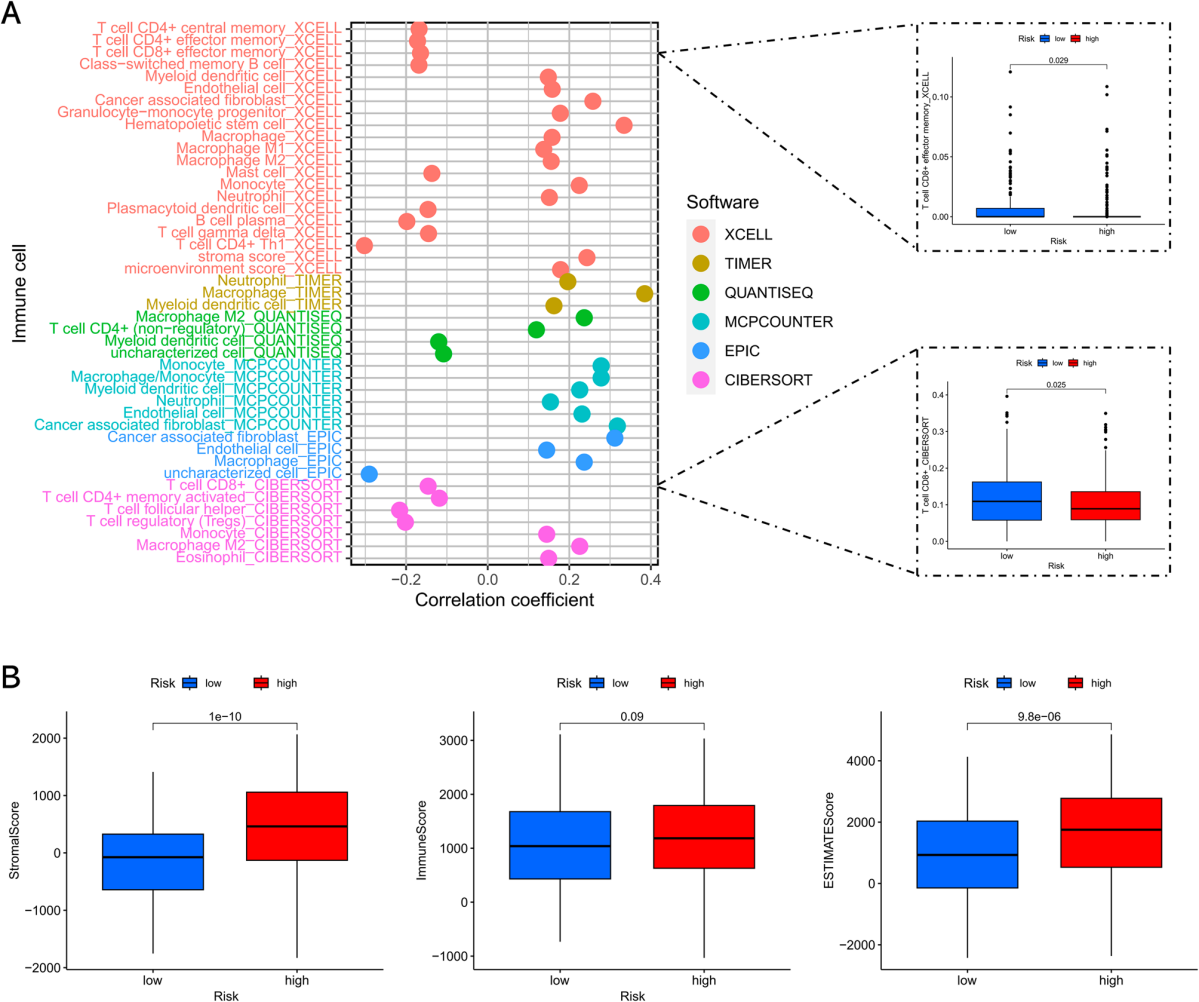
The correlation between the risk signature and tumor immune microenvironment. (**A**) The lollipop plot of the relationship between risk score and TIICs in multiple databases, and the boxplot shows the difference of CD8+ T cell infiltration between different risk groups. (**B**) The boxplot of the differences in stromal score, immune score and ESTIMATE score between low- and high-risk groups.

We utilized distinct algorithms to estimate the infiltration of TIICs in each patient and evaluated their association with the risk score. As a result, the risk score was found to be remarkably associated with the infiltration level of most of the TIICs, especially CD8+ T cell (Fig. 8A). In terms of TME scores, we found that the stromal score and estimate score were remarkably elevated in the high-risk subgroup compared to the low-risk subgroup, whereas the difference in immune score between the two risk subgroups was not significant (Fig. 8B).

### Prediction of immunotherapeutic responsiveness

**Figure 9:**
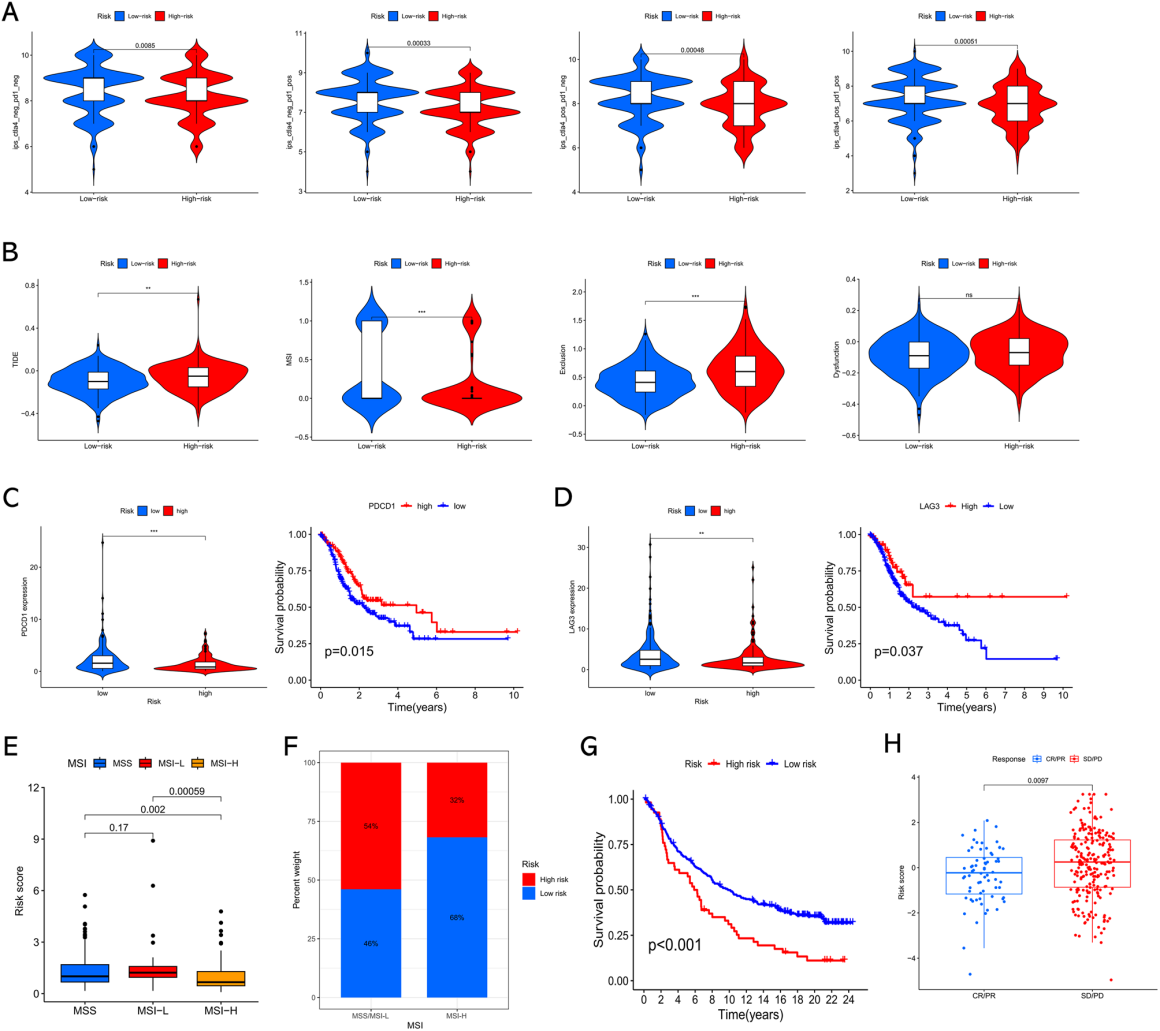
Association of risk signature with immunotherapy sensitivity. (**A**) The differences in IPS between low- and high-risk groups. (**B**) The differences in TIDE scores between low- and high-risk groups. (**C**) Expression and prognostic value of PDCD1 in GC. (**D**) Expression and prognostic value of LAG3 in GC. (**E**, **F**) The correlation between the risk score and MSI status. (**G**) Kaplan–Meier survival analysis of the risk signature in IMvigor210 cohort. (**H**) The comparison of risk score between SD/PD and CR/PR groups in IMvigor210 cohort. *P < 005; **P < 0.01; ***P < 0.001.

We further determined the capability of the risk score for forecasting the clinical immunotherapeutic responsiveness in GC. Firstly, our findings suggested that the IPSs were obviously higher in the low-risk subgroup, which means more immunogenicity to immune checkpoint inhibitors (ICIs) in the low-risk subgroup (Fig. 9A). For the TIDE, the TIDE and Exclusion scores were remarkably elevated in the high-risk subgroup, while the MSI score was higher in the low-risk subgroup, implying that immune evasion was more common in the high-risk subgroup (Fig. 9B). Subsequently, the differences in ICB expression levels between the two risk subgroups were compared. As shown in Fig. 9C and D, PD-1 and LAG3 were overexpressed in the low-risk subgroup and remarkably correlated with better overall survival in GC. Besides, we evaluated the correlation between the risk score and MSI status and revealed that a low-risk score was significantly correlated with MSI-H status, whereas a high-risk score was correlated with microsatellite stable (MSS) and MSI-low (MSI-L) status (Fig. 9E and F).

Moreover, due to the lack of data on immunotherapy for GC, we determined the performance of the risk signature in forecasting patients’ sensitivity to immunotherapy based on the IMvigor210 cohort. As a result, urothelial cancer patients possess high-risk score also showed significantly worse overall survival probability, and the CR/PR patients possessed significant lower risk score compared to SD/PD patients (Fig. 9G and H). This result partially demonstrated that our risk signature has the ability to predict tumor responsiveness to immunotherapy.

## Discussion

Developing predictive biomarkers for forecasting the survival outcome and therapy response of tumors is of great significance. Considering that performing bulk RNA-seq and scRNA-seq in clinics are time-consuming and costly, recent studies are inclined to develop clinically feasible tools based on public databases and burgeoning technologies such as bioinformatics, which is convenient and cost-efficient. In the present study, we constructed a CD8+ T cell-associated prognostic signature to evaluate the clinical outcomes and anti-cancer immunotherapeutic sensitivity of GC and validated the translatability to the clinical setting via retrospective analysis of specimens from multiple distinct populations. Our results highlighted the significance of using pre-clinical signatures to generate clinical tools as well as the benefits of applying burgeoning technologies such as bulk RNA-seq and scRNA-seq to investigate distinctive immunological landscapes in TME.

The novel risk signature was developed according to the coefficients and expression levels of eight CD8+ T cell marker genes: CXCR4, NPC2, DDX24, ZFP36, TGFB1, PDCD1, NPDC1, and SRI. Most of these genes have been reported to participate in tumorigenesis. For example, CXCR4 is a member of the G protein-coupled receptor family and serves as a receptor for SDF-1. Previously published researches have demonstrated that CXCR4 was overexpressed in GC and affects the proliferation, migration and invasion of cancer cells via the activation of diverse signaling pathways, such as ERK/Akt, NF-kB, JAK2/STAT3, and Wnt/β-catenin pathways^31^. In addition. CXCR4 has been demonstrated to serve as a significant part in modulating the differentiation and directional migration of immune cells in TME^32^. TGFB1 was overexpressed in GC cells and participated in cancer progression by inducing cell proliferation, metastasis, glycolysis, angiogenesis, and depressing apoptosis^33^. The activation of TGFB signaling depresses the biological activity of cytotoxic T-lymphocytes and natural killer cells by promoting the proliferation of regulatory T-cells (Tregs) and cancer-associated fibroblasts (CAFs), thus creating an immunosuppressive TME^34^. PDCD1 encodes the PD-1 protein, which is an immunosuppressive molecule that is widely overexpressed on the tumor-infiltrating lymphocytes. PD-1 mediates T-cell exhaustion and dysfunction in TME, thereby inducing immune evasion and tumor progression^35,36^. PD-1 targeted therapy has gained promising efficiency in GC with MSI-H or EBV (+) tumors^37^. SRI was overexpressed in GC and has oncogenic activity in tumor progression by promoting migration and invasion in vitro^38^. Besides, highly expressed SRI was involved in poor responses to chemotherapy in GC cells ^39^. NPC2 is a tumor suppressor by modulating MAPK/ERK signaling in primary hepatocellular carcinoma^40^. DDX24 has been demonstrated to be highly expressed in non-small cell lung cancer and associated with unfavourable clinical outcomes, with the silencing of DDX24 remarkably restraining cell migration and invasion in vitro and in vivo^41^. In this study, we include these genes to generate a prognostic risk signature for forecasting the clinical outcome of GC patients and found that the predictive capability of the risk signature is prior to each single gene. In addition, univariate and multivariate Cox analysis revealed that the risk signature can be used as an independent predictor for GC patients. Importantly, independent validation cohorts further confirmed the universal applicability of our signature in different populations, patients who possess high-risk score need more frequent follow-ups to monitor the recurrence of GC.

Cancer cells can use multiple strategies to modify the immunity of the immune system in recognizing and destructing them. Over the past couple of decades, immunotherapy focused on the reactivation of the host immunity has gained promising progress as an anti-tumor therapy strategy for several solid tumors. Among them, ICIs, which relieve restrictions on immune cells to recover anti-cancer immunological activity, have produced unprecedented clinical benefits, especially anti-PD-1 and anti-CTLA-4 strategies. In terms of GC, ICI targeting PD-1 combined with trastuzumab as the first-line therapy for HER-2 positive patients has received approval by the FDA^42^. Besides, ICIs combined with chemotherapy also exhibited satisfactory effects in treating advanced GC^43^. However, it is difficult to predict the clinical efficacy of immunotherapy due to the great heterogeneity in individuals. Therefore, exploring potential biomarkers for distinguishing patients who might respond well to immunotherapy is of great worth. Currently, potential biomarkers used for evaluating cancer patients who might be sensitive to immunotherapy include immune checkpoint expression^30^, TMB^18^, MSI status^28^, IPS^26^, and TIDE scores^27^. We evaluated whether the CD8+ T cell-associated signature could be applied as a predictive index for immunotherapy of GC from the above perspectives. As expected, our results demonstrated that high-risk score was obviously related to low immune checkpoint expression, low TMB, MSI-L/MSS status, low IPS, and high TIDE scores, suggesting that patients possessing high-risk score were less likely to benefit from immunotherapy. In addition, we explored our findings in the immunotherapy cohort IMvigor210 and found that patients in the PR/CR subgroup possess lower risk scores compared with those in the SD/PD subgroup, implying that high-risk patients would gain worse efficacy in response to immunotherapy.

Inevitably, several limitations exist in the present research. Firstly, the prognostic signature was developed based on retrospective data retrieved from online platforms, additional multi-center prospective research is required to validate its stability. Secondly, since there are no data on immunotherapy for GC, the performance of the risk signature in predicting patients’ sensitivity to immunotherapy was validated only using IMvigor210 cohort, which might inevitably affect the reliability of our findings. Besides, our study was almost descriptive, further in vitro and in vivo experiments are required to determine the specific biological functions of the eight genes in tumor immune infiltration.

## Conclusion

In summary, our study developed a prognostic signature comprised of eight CD8+ T cell feature genes to forecast the clinical outcomes of GC patients by integrating scRNA-seq and bulk RNA-seq technologies. The risk signature was found to be remarkably associated with the immunological characteristics and could be used as a novel biomarker in predicting immunotherapeutic responses. In the future, the risk signature is expected to provide worthwhile information for clinical decision-making and propose novel immunotherapeutic strategies for GC treatment.

### Supplementary Information

Supplementary Figure 1.

Supplementary Table 1.

Supplementary Table 2.

Supplementary Legends.

## Data Availability

Thedatasets generated and/or analyzed during the current studyare available in the Tumor Immune Single-cell Hub 2 (TISCH2, http://tisch.comp-genomics.org/), Gene Expression Omnibus (GEO, https://www.ncbi.nlm.nih.gov/geo/), and The Cancer Genome Atlas (TCGA, https://www.cancer.gov/tcga) projects.

## Abbreviations

AUC: Area under the curve
GC: Gastric cancer
scRNA-seq: Single-cell RNA sequencing
TME: Tumor microenvironment
TMB: Tumor mutation burden
MSI: Microsatellite instability
IFNγ: Interferon-γ
TNF: Tumor necrosis factor
TISCH2: Tumor Immune Single-cell Hub 2
RNA-seq: RNA sequencing
TCGA: The Cancer Genome Atlas
GEO: Gene Expression Omnibus
GO: Gene Ontology
KEGG: Kyoto Encyclopedia of Genes and Genomes
DAVID: Database for Annotation, Visualization and Integrated Discovery
LASSO: Least absolute shrinkage and selection operator
ROC: Receiver operating characteristic
DCA: Decision curve analysis
FDA: Food and Drug Administration
TIIC: Tumor infiltrated immune cell
IPS: Immunophenoscore
TCIA: The Cancer Immunome Atlas
TIDE: Tumor Immune Dysfunction and Exclusion
dMMR: Defective DNA mismatch repair
MSI-H: Microsatellite instability-high
CR: Complete response
PR: Partial response
SD: Stable disease
PD: Progressive disease
ICI: Immune checkpoint inhibitor
MSS: Microsatellite stable
MSI-L: Microsatellite instability-low
Tregs: Regulatory T-cells
CAF: Cancer-associated fibroblast
ICB: Immune checkpoint biomarker
ssGSEA: Single-sample gene set enrichment analysis
