# Supplementary figures and images for: Development of a CD8+ T cell associated signature for predicting the prognosis and immunological characteristics of gastric cancer by integrating single-cell and bulk RNA-sequencing
Source: Sci Rep. 2024 Feb 24;14:4524. doi: 10.1038/s41598-024-54273-9 (PMC10894294; doi:10.1038/s41598-024-54273-9)

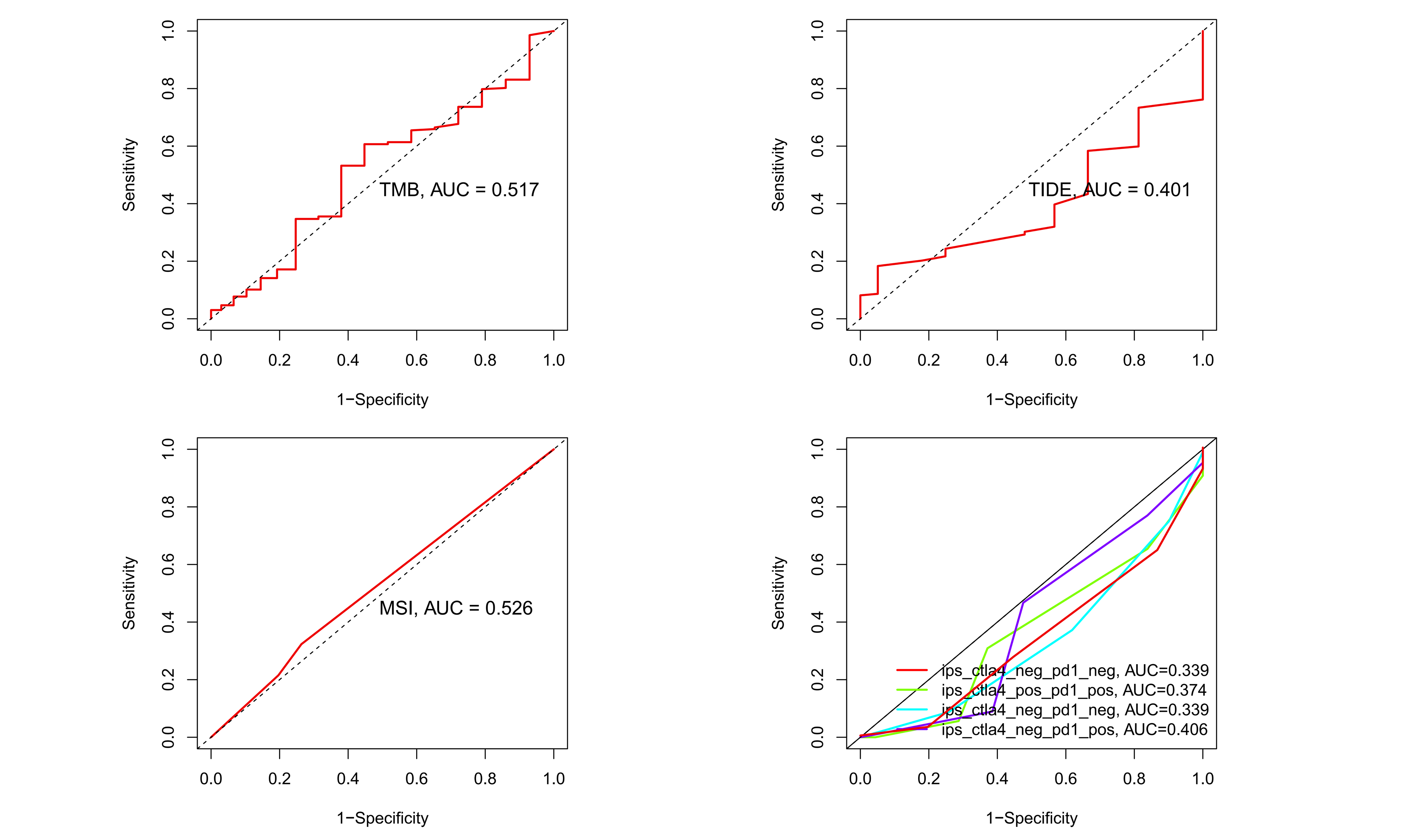

Supplement: Supplementary file 1 — Supplementary Figure 1. [file 41598_2024_54273_MOESM1_ESM.tif]
