# Supplementary material for: Development of a CD8+ T cell associated signature for predicting the prognosis and immunological characteristics of gastric cancer by integrating single-cell and bulk RNA-sequencing
Source: Sci Rep. 2024 Feb 24;14:4524. doi: 10.1038/s41598-024-54273-9 (PMC10894294; doi:10.1038/s41598-024-54273-9)
Supplement: Supplementary file 2 — Supplementary Table 1. [file 41598_2024_54273_MOESM2_ESM.docx]

| Supplementary Table 1. Differentially expressed genes of each cell cluster in GSE167297 | | | | | | |
| --- | --- | --- | --- | --- | --- | --- |
| Celltype (major-lineage) | Celltype (minor-lineage) | Gene | | log2FC | | Adjusted p-value |
| B | B | CD79A | 1.59 | | 0 | |
| B | B | HLA-DRA | 1.59 | | 0 | |
| B | B | CD74 | 1.56 | | 0 | |
| B | B | HLA-DQB1 | 1.53 | | 0 | |
| B | B | CD83 | 1.49 | | 0 | |
| B | B | MS4A1 | 1.47 | | 0 | |
| B | B | LY9 | 1.37 | | 0 | |
| B | B | HLA-DQA1 | 1.35 | | 0 | |
| B | B | HLA-DPA1 | 1.3 | | 0 | |
| B | B | CD37 | 1.25 | | 0 | |
| B | B | HLA-DQA2 | 1.24 | | 0 | |
| B | B | BANK1 | 1.24 | | 0 | |
| B | B | LAPTM5 | 1.22 | | 0 | |
| B | B | HLA-DRB1 | 1.22 | | 0 | |
| B | B | HLA-DRB5 | 1.16 | | 0 | |
| B | B | VPREB3 | 1.15 | | 0 | |
| B | B | HLA-DPB1 | 1.13 | | 0 | |
| B | B | CD69 | 1.02 | | 0 | |
| B | B | HLA-DMA | 1.02 | | 0 | |
| B | B | CXCR4 | 0.93 | | 0 | |
| B | B | CD55 | 0.92 | | 0 | |
| B | B | EZR | 0.89 | | 0 | |
| B | B | RUBCNL | 0.86 | | 0 | |
| B | B | PHACTR1 | 0.8 | | 0 | |
| B | B | CD19 | 0.8 | | 0 | |
| B | B | FCER2 | 0.79 | | 0 | |
| B | B | SPIB | 0.77 | | 0 | |
| B | B | HLA-DMB | 0.77 | | 0 | |
| B | B | SMIM14 | 0.76 | | 0 | |
| B | B | IRF8 | 0.74 | | 0 | |
| B | B | NFKBID | 0.72 | | 0 | |
| B | B | GNG7 | 0.7 | | 0 | |
| B | B | LY86 | 0.7 | | 0 | |
| B | B | RPS23 | 0.67 | | 0 | |
| B | B | MEF2C | 0.66 | | 0 | |
| B | B | EEF1B2 | 0.64 | | 0 | |
| B | B | RPS8 | 0.64 | | 0 | |
| B | B | RPLP2 | 0.64 | | 0 | |
| B | B | RPS27 | 0.62 | | 0 | |
| B | B | LINC00926 | 0.62 | | 0 | |
| B | B | KDM4B | 0.62 | | 0 | |
| B | B | FCMR | 0.61 | | 0 | |
| B | B | RPS11 | 0.58 | | 0 | |
| B | B | TNFRSF13B | 0.58 | | 0 | |
| B | B | ARHGAP24 | 0.58 | | 0 | |
| B | B | RPSA | 0.57 | | 0 | |
| B | B | RPL21 | 0.56 | | 0 | |
| B | B | SNX29P2 | 0.56 | | 0 | |
| B | B | RPL18A | 0.55 | | 0 | |
| B | B | FCRL1 | 0.53 | | 0 | |
| B | B | RPS5 | 0.53 | | 0 | |
| B | B | RPS10 | 0.53 | | 0 | |
| B | B | CD52 | 0.53 | | 0 | |
| B | B | TSC22D3 | 0.52 | | 0 | |
| B | B | BLK | 0.52 | | 0 | |
| B | B | RPL11 | 0.51 | | 0 | |
| B | B | CD22 | 0.51 | | 0 | |
| B | B | NOP53 | 0.51 | | 0 | |
| B | B | RPL9 | 0.5 | | 0 | |
| B | B | RPL32 | 0.5 | | 0 | |
| B | B | RPS29 | 0.5 | | 0 | |
| B | B | RPS21 | 0.5 | | 0 | |
| B | B | RPL34 | 0.5 | | 0 | |
| B | B | RPL19 | 0.49 | | 0 | |
| B | B | RPL30 | 0.49 | | 0 | |
| B | B | RPL39 | 0.49 | | 0 | |
| B | B | RPS20 | 0.49 | | 0 | |
| B | B | RPS25 | 0.48 | | 0 | |
| B | B | RPS3A | 0.48 | | 0 | |
| B | B | RPL23A | 0.48 | | 0 | |
| B | B | RPL10A | 0.48 | | 0 | |
| B | B | RPS13 | 0.48 | | 0 | |
| B | B | RPL5 | 0.48 | | 0 | |
| B | B | FCRLA | 0.48 | | 0 | |
| B | B | RPS6 | 0.48 | | 0 | |
| B | B | RPS27A | 0.47 | | 0 | |
| B | B | RPL13A | 0.46 | | 0 | |
| B | B | FAU | 0.46 | | 0 | |
| B | B | RPL31 | 0.45 | | 0 | |
| B | B | RPL26 | 0.45 | | 0 | |
| B | B | TNFRSF13C | 0.44 | | 0 | |
| B | B | EEF1A1 | 0.44 | | 0 | |
| B | B | H3F3A | 0.44 | | 0 | |
| B | B | RPL27 | 0.43 | | 0 | |
| B | B | RPL27A | 0.43 | | 0 | |
| B | B | RPL37 | 0.43 | | 0 | |
| B | B | PTMA | 0.42 | | 0 | |
| B | B | RPS12 | 0.42 | | 0 | |
| B | B | RPL4 | 0.42 | | 0 | |
| B | B | RPL22 | 0.42 | | 0 | |
| B | B | RPL38 | 0.41 | | 0 | |
| B | B | RPL8 | 0.41 | | 0 | |
| B | B | RPL23 | 0.4 | | 0 | |
| B | B | RPS18 | 0.4 | | 0 | |
| B | B | RPL18 | 0.39 | | 0 | |
| B | B | LINC02397 | 0.39 | | 0 | |
| B | B | RPL15 | 0.39 | | 0 | |
| B | B | RPS28 | 0.38 | | 0 | |
| B | B | RPL3 | 0.38 | | 0 | |
| B | B | RPL12 | 0.38 | | 0 | |
| B | B | RPL35A | 0.36 | | 0 | |
| B | B | RPS14 | 0.36 | | 0 | |
| B | B | RPL29 | 0.36 | | 0 | |
| B | B | RPL13 | 0.35 | | 0 | |
| B | B | FAM129C | 0.35 | | 0 | |
| B | B | RPS7 | 0.34 | | 0 | |
| B | B | RPS2 | 0.33 | | 0 | |
| B | B | RPS15A | 0.32 | | 0 | |
| B | B | RPL7 | 0.32 | | 0 | |
| B | B | RPS3 | 0.32 | | 0 | |
| B | B | RPS9 | 0.32 | | 0 | |
| B | B | UBA52 | 0.32 | | 0 | |
| B | B | RPL37A | 0.31 | | 0 | |
| B | B | RPL41 | 0.31 | | 0 | |
| B | B | RPL10 | 0.28 | | 0 | |
| B | B | DSTN | -0.81 | | 0 | |
| B | B | GAPDH | -0.92 | | 0 | |
| B | B | GSTP1 | -0.95 | | 0 | |
| B | B | ANXA2 | -0.99 | | 0 | |
| B | B | CYTOR | -0.99 | | 0 | |
| B | B | CSTB | -1 | | 0 | |
| B | B | CD3E | -1.04 | | 0 | |
| B | B | NEAT1 | -1.05 | | 0 | |
| B | B | S100A10 | -1.07 | | 0 | |
| B | B | TNFAIP3 | -1.1 | | 0 | |
| B | B | LGALS3 | -1.12 | | 0 | |
| B | B | CD2 | -1.13 | | 0 | |
| B | B | VIM | -1.34 | | 0 | |
| B | B | CD63 | -1.34 | | 0 | |
| B | B | TXN | -1.39 | | 0 | |
| B | B | CD3D | -1.43 | | 0 | |
| B | B | S100A11 | -1.43 | | 0 | |
| B | B | CD7 | -1.46 | | 0 | |
| B | B | ID2 | -1.48 | | 0 | |
| B | B | S100A4 | -1.64 | | 0 | |
| B | B | MT2A | -1.67 | | 0 | |
| B | B | ANXA1 | -1.71 | | 0 | |
| B | B | CST3 | -1.72 | | 0 | |
| B | B | IL32 | -1.83 | | 0 | |
| B | B | S100A6 | -2.08 | | 0 | |
| B | B | TIMP1 | -2.46 | | 0 | |
| B | B | LINC01781 | 0.55 | | 1.31E-303 | |
| B | B | RPS4X | 0.31 | | 7.55E-301 | |
| B | B | IFITM3 | -1.44 | | 4.16E-299 | |
| B | B | ELOB | -0.68 | | 5.42E-298 | |
| B | B | CST7 | -1.23 | | 8.30E-295 | |
| B | B | CCR7 | 0.57 | | 6.21E-294 | |
| B | B | RPS19 | 0.26 | | 1.73E-291 | |
| B | B | CTSD | -0.99 | | 6.79E-290 | |
| B | B | CXCR5 | 0.4 | | 4.88E-287 | |
| B | B | LGALS1 | -1.47 | | 1.54E-286 | |
| B | B | YPEL5 | 0.54 | | 2.97E-286 | |
| B | B | BTG1 | 0.41 | | 1.73E-283 | |
| B | B | RPL35 | 0.27 | | 4.20E-283 | |
| B | B | AFF3 | 0.35 | | 5.07E-283 | |
| B | B | IER3 | -1.17 | | 9.60E-279 | |
| B | B | FYN | -0.83 | | 7.38E-276 | |
| B | B | CCL5 | -1.88 | | 9.83E-275 | |
| B | B | CMTM6 | 0.64 | | 5.24E-268 | |
| B | B | LYZ | -2.06 | | 5.52E-268 | |
| B | B | RAC1 | -0.72 | | 6.40E-266 | |
| B | B | TSPO | -0.71 | | 3.36E-260 | |
| B | B | ITM2A | -0.82 | | 5.53E-259 | |
| B | B | PPIB | -0.66 | | 2.22E-258 | |
| B | B | XBP1 | -1.14 | | 2.91E-257 | |
| B | B | TCL1A | 0.38 | | 7.90E-255 | |
| B | B | CD72 | 0.46 | | 9.94E-255 | |
| B | B | PHLDA1 | -0.85 | | 2.08E-253 | |
| B | B | LYN | 0.62 | | 2.64E-253 | |
| B | B | SDF2L1 | -0.66 | | 2.97E-252 | |
| B | B | RPS16 | 0.26 | | 2.21E-250 | |
| B | B | RPS27L | -0.64 | | 3.98E-248 | |
| B | B | ZFAS1 | 0.53 | | 4.41E-247 | |
| B | B | HCST | -0.89 | | 1.74E-246 | |
| B | B | ITM2B | -0.66 | | 4.39E-246 | |
| B | B | RPL14 | 0.29 | | 8.73E-246 | |
| B | B | ANXA5 | -0.74 | | 1.04E-243 | |
| B | B | EEF2 | 0.36 | | 6.73E-243 | |
| B | B | CFLAR | -0.58 | | 8.29E-241 | |
| B | B | RASGRP2 | 0.49 | | 1.30E-239 | |
| B | B | PHPT1 | -0.52 | | 1.95E-238 | |
| B | B | NR4A2 | 0.63 | | 1.27E-235 | |
| B | B | CTSC | -0.68 | | 1.73E-235 | |
| B | B | RHOC | -0.62 | | 5.20E-235 | |
| B | B | LDHA | -0.72 | | 2.90E-230 | |
| B | B | SEC61G | -0.59 | | 1.82E-229 | |
| B | B | PFDN5 | 0.33 | | 1.83E-229 | |
| B | B | P4HB | -0.56 | | 4.53E-229 | |
| B | B | TRIB1 | -0.68 | | 5.46E-229 | |
| B | B | RACK1 | 0.3 | | 8.85E-229 | |
| B | B | REL | 0.57 | | 3.93E-227 | |
| B | B | PTMS | -0.59 | | 1.24E-226 | |
| B | B | NDUFB7 | -0.54 | | 2.39E-226 | |
| B | B | IFI27 | -1.37 | | 6.07E-226 | |
| B | B | MT1X | -0.87 | | 3.57E-225 | |
| B | B | CYTIP | 0.54 | | 3.63E-225 | |
| B | B | MGST3 | -0.6 | | 2.14E-224 | |
| B | B | FKBP11 | -1.13 | | 2.91E-224 | |
| B | B | NKG7 | -1.51 | | 6.58E-224 | |
| B | B | RPL7A | 0.27 | | 6.58E-224 | |
| B | B | UQCRQ | -0.63 | | 1.39E-223 | |
| B | B | HVCN1 | 0.44 | | 1.52E-223 | |
| B | B | CD48 | 0.49 | | 3.25E-222 | |
| B | B | ADAM28 | 0.6 | | 3.81E-220 | |
| B | B | ORAI2 | 0.56 | | 1.50E-218 | |
| B | B | ZNF331 | 0.64 | | 4.13E-218 | |
| B | B | RRBP1 | -0.45 | | 7.36E-217 | |
| B | B | PERP | -0.58 | | 3.90E-216 | |
| B | B | IFI6 | -0.79 | | 3.54E-214 | |
| B | B | MIR4435-2HG | -0.56 | | 1.68E-213 | |
| B | B | CD59 | -0.65 | | 5.37E-212 | |
| B | B | ATOX1 | -0.51 | | 5.44E-212 | |
| B | B | FKBP2 | -0.74 | | 6.25E-212 | |
| B | B | RPL6 | 0.27 | | 9.30E-212 | |
| B | B | FOSL2 | -0.52 | | 5.55E-210 | |
| B | B | IL7R | -0.85 | | 8.55E-210 | |
| B | B | NDUFC2 | -0.51 | | 3.18E-209 | |
| B | B | EIF1 | 0.3 | | 6.66E-207 | |
| B | B | CKLF | -0.53 | | 2.08E-206 | |
| B | B | CHPT1 | 0.56 | | 3.27E-203 | |
| B | B | CTSB | -1 | | 6.28E-203 | |
| B | B | KRT8 | -1.44 | | 9.41E-203 | |
| B | B | TECR | -0.47 | | 4.31E-202 | |
| B | B | PIM1 | -0.54 | | 6.63E-202 | |
| B | B | PLAUR | -1.27 | | 1.25E-200 | |
| B | B | HSPB1 | -0.97 | | 2.44E-200 | |
| B | B | NINJ1 | -0.62 | | 3.37E-200 | |
| B | B | SELENOW | -0.54 | | 8.74E-200 | |
| B | B | LEPROTL1 | -0.69 | | 2.32E-199 | |
| B | B | GADD45A | -0.65 | | 7.68E-199 | |
| B | B | SRGN | -0.77 | | 2.20E-196 | |
| B | B | CXCL2 | -1.65 | | 1.21E-195 | |
| B | B | SSR4 | -1.52 | | 3.81E-195 | |
| B | B | CXCL3 | -1.54 | | 3.36E-194 | |
| B | B | CEBPD | -0.62 | | 4.28E-194 | |
| B | B | EMP3 | 0.73 | | 2.34E-193 | |
| B | B | BCL11A | 0.31 | | 1.80E-191 | |
| B | B | ANKRD28 | -0.67 | | 2.92E-190 | |
| B | B | SEMA7A | 0.46 | | 4.56E-190 | |
| B | B | ATP5IF1 | -0.51 | | 4.75E-190 | |
| B | B | NUCB2 | -0.52 | | 6.44E-189 | |
| B | B | CD9 | -0.71 | | 8.58E-189 | |
| B | B | SPCS3 | -0.58 | | 8.97E-189 | |
| B | B | CXCL8 | -2.39 | | 1.44E-188 | |
| B | B | PSME2 | -0.54 | | 9.19E-188 | |
| B | B | TMEM59 | -0.64 | | 2.87E-187 | |
| B | B | COX8A | -0.52 | | 7.82E-187 | |
| B | B | PHLDA2 | -0.62 | | 2.10E-186 | |
| B | B | NDUFB10 | -0.44 | | 8.12E-186 | |
| B | B | GZMA | -1.25 | | 1.52E-185 | |
| B | B | CD151 | -0.45 | | 4.75E-185 | |
| B | B | SSR3 | -0.64 | | 8.73E-185 | |
| B | B | MGAT1 | -0.42 | | 1.29E-184 | |
| B | B | SNX2 | 0.57 | | 4.43E-184 | |
| B | B | SUB1 | -0.47 | | 1.71E-183 | |
| B | B | CEBPB | -0.6 | | 2.84E-183 | |
| B | B | MYDGF | -0.53 | | 6.96E-183 | |
| B | B | GLUL | -0.75 | | 1.75E-179 | |
| B | B | ZNHIT1 | -0.4 | | 1.85E-179 | |
| B | B | AL121944.1 | 0.52 | | 2.17E-179 | |
| B | B | NDUFS8 | -0.41 | | 4.52E-179 | |
| B | B | PRDX4 | -0.86 | | 2.35E-178 | |
| B | B | PDIA4 | -0.46 | | 3.96E-178 | |
| B | B | FYB1 | -0.6 | | 4.98E-178 | |
| B | B | NACA | 0.27 | | 6.60E-178 | |
| B | B | LGALS3BP | -0.52 | | 2.22E-176 | |
| B | B | SELENOS | -0.52 | | 7.60E-176 | |
| B | B | SERF2 | -0.37 | | 1.97E-175 | |
| B | B | RGS10 | -0.52 | | 1.37E-174 | |
| B | B | SH3BGRL3 | -0.6 | | 2.88E-174 | |
| B | B | GZMB | -1.45 | | 1.99E-173 | |
| B | B | TYROBP | -1.55 | | 2.28E-173 | |
| B | B | UPP1 | -0.45 | | 6.27E-173 | |
| B | B | HM13 | -0.39 | | 1.10E-172 | |
| B | B | KRT18 | -1.28 | | 1.71E-172 | |
| B | B | HSP90B1 | -0.77 | | 4.04E-172 | |
| B | B | DNAJC1 | -0.37 | | 1.11E-171 | |
| B | B | RGCC | -0.72 | | 7.61E-171 | |
| B | B | H2AFJ | -0.4 | | 1.53E-170 | |
| B | B | RASGEF1B | 0.53 | | 8.12E-170 | |
| B | B | C15orf48 | -1.51 | | 1.47E-169 | |
| B | B | ITM2C | -0.78 | | 5.02E-168 | |
| B | B | TSPAN8 | -1.31 | | 9.77E-168 | |
| B | B | COX5A | -0.48 | | 2.20E-167 | |
| B | B | TMEM258 | -0.51 | | 4.15E-167 | |
| B | B | CITED2 | -0.73 | | 1.31E-166 | |
| B | B | LY6E | -0.56 | | 3.03E-166 | |
| B | B | ATP5PF | -0.46 | | 3.23E-166 | |
| B | B | GZMM | -0.62 | | 8.39E-165 | |
| B | B | LIMD2 | 0.43 | | 2.15E-164 | |
| B | B | DNAJB1 | -0.67 | | 2.55E-164 | |
| B | B | SH2D2A | -0.59 | | 5.31E-164 | |
| B | B | ACADVL | -0.37 | | 1.03E-163 | |
| B | B | ETS2 | -0.46 | | 1.72E-163 | |
| B | B | MT1E | -0.82 | | 7.02E-163 | |
| B | B | KDELR1 | -0.37 | | 8.45E-163 | |
| B | B | ACTN4 | -0.42 | | 1.08E-162 | |
| B | B | ATP5MC3 | -0.5 | | 9.35E-162 | |
| B | B | EIF1B | 0.54 | | 7.36E-161 | |
| B | B | BLVRB | -0.44 | | 5.02E-160 | |
| B | B | AGR2 | -1.42 | | 1.51E-158 | |
| B | B | CORO1A | 0.32 | | 8.92E-158 | |
| B | B | RABAC1 | -0.49 | | 1.20E-157 | |
| B | B | ATP5ME | -0.5 | | 6.13E-157 | |
| B | B | RORA | -0.57 | | 1.33E-156 | |
| B | B | KLRB1 | -1.11 | | 1.51E-156 | |
| B | B | ISG15 | -0.73 | | 3.98E-156 | |
| B | B | PRDX5 | -0.45 | | 5.85E-156 | |
| B | B | MDK | -0.55 | | 1.70E-155 | |
| B | B | DDX5 | 0.29 | | 3.95E-155 | |
| B | B | LMNA | -0.6 | | 4.37E-155 | |
| B | B | GLRX | -0.47 | | 1.98E-154 | |
| B | B | FCER1G | -1.67 | | 6.72E-154 | |
| B | B | TPI1 | -0.48 | | 1.43E-153 | |
| B | B | BHLHE40 | -0.51 | | 3.34E-153 | |
| B | B | SURF4 | -0.36 | | 4.44E-152 | |
| B | B | RIC3 | 0.37 | | 6.85E-152 | |
| B | B | B2M | -0.31 | | 8.80E-152 | |
| B | B | CARD16 | -0.51 | | 1.79E-151 | |
| B | B | KRT19 | -1.03 | | 2.79E-151 | |
| B | B | CD3G | -0.55 | | 4.98E-151 | |
| B | B | NDUFS6 | -0.42 | | 1.83E-150 | |
| B | B | ELF3 | -0.9 | | 1.71E-149 | |
| B | B | RPL17 | 0.39 | | 2.22E-149 | |
| B | B | RARRES3 | -0.55 | | 3.73E-149 | |
| B | B | TMEM14C | -0.34 | | 4.54E-149 | |
| B | B | MTHFD2 | -0.35 | | 8.00E-149 | |
| B | B | GSN | -0.94 | | 1.16E-148 | |
| B | B | H1FX | -0.39 | | 1.35E-148 | |
| B | B | RAB13 | -0.38 | | 1.35E-148 | |
| B | B | TXNDC17 | -0.38 | | 1.97E-148 | |
| B | B | C7orf50 | 0.56 | | 5.26E-148 | |
| B | B | HLA-DOA | 0.32 | | 6.32E-148 | |
| B | B | STK4 | 0.42 | | 9.00E-148 | |
| B | B | BSG | -0.41 | | 1.33E-147 | |
| B | B | PRDM1 | -0.46 | | 5.04E-147 | |
| B | B | FXYD3 | -0.94 | | 9.08E-147 | |
| B | B | CALM3 | -0.39 | | 8.51E-146 | |
| B | B | ATP5MD | -0.46 | | 2.76E-145 | |
| B | B | ADIRF | -1.03 | | 3.05E-145 | |
| B | B | CTSL | -0.82 | | 6.32E-145 | |
| B | B | KDELR2 | -0.39 | | 1.83E-144 | |
| B | B | PLD3 | -0.36 | | 1.85E-144 | |
| B | B | SYNE2 | -0.4 | | 2.77E-144 | |
| B | B | SLC7A5 | -0.52 | | 1.51E-143 | |
| B | B | GPX1 | -0.67 | | 1.54E-143 | |
| B | B | CD79B | 0.56 | | 1.55E-143 | |
| B | B | BST2 | -0.48 | | 2.58E-143 | |
| B | B | DAD1 | -0.42 | | 9.91E-143 | |
| B | B | IFITM1 | -0.68 | | 1.26E-142 | |
| B | B | CD99 | -0.46 | | 2.14E-142 | |
| B | B | NDUFA4 | -0.42 | | 4.13E-142 | |
| B | B | GPR18 | 0.43 | | 5.07E-142 | |
| B | B | SDF4 | -0.31 | | 8.35E-142 | |
| B | B | LAMP2 | -0.3 | | 1.80E-141 | |
| B | B | MVP | -0.32 | | 1.92E-141 | |
| B | B | GAS6 | -0.32 | | 6.03E-141 | |
| B | B | HNRNPA1 | 0.25 | | 9.97E-141 | |
| B | B | KRTCAP2 | -0.46 | | 1.10E-140 | |
| B | B | SEC61B | -0.49 | | 1.52E-140 | |
| B | B | SIDT1 | 0.27 | | 1.54E-140 | |
| B | B | MUC1 | -0.73 | | 3.30E-140 | |
| B | B | ITGB1 | -0.38 | | 8.44E-140 | |
| B | B | POU2F2 | 0.4 | | 1.69E-139 | |
| B | B | TMSB4X | -0.5 | | 5.64E-139 | |
| B | B | TPM1 | -0.43 | | 1.15E-138 | |
| B | B | OPTN | -0.32 | | 1.32E-138 | |
| B | B | CORO1B | -0.36 | | 1.55E-138 | |
| B | B | TIMM8B | -0.32 | | 1.72E-138 | |
| B | B | MYL6 | -0.37 | | 2.41E-138 | |
| B | B | MGST2 | -0.34 | | 2.58E-138 | |
| B | B | S100A13 | -0.4 | | 2.77E-138 | |
| B | B | HDLBP | -0.26 | | 3.56E-138 | |
| B | B | RCSD1 | 0.41 | | 4.40E-138 | |
| B | B | C19orf33 | -0.62 | | 6.10E-138 | |
| B | B | CD247 | -0.54 | | 8.47E-138 | |
| B | B | CHI3L2 | 0.27 | | 9.70E-138 | |
| B | B | TM4SF1 | -1.17 | | 2.05E-137 | |
| B | B | IDH2 | -0.36 | | 7.00E-137 | |
| B | B | LSM7 | 0.54 | | 8.92E-137 | |
| B | B | VPS37B | 0.57 | | 1.77E-136 | |
| B | B | MAFF | -0.4 | | 1.88E-136 | |
| B | B | NECTIN2 | -0.3 | | 2.77E-136 | |
| B | B | NBL1 | -0.37 | | 2.78E-136 | |
| B | B | TNFRSF1A | -0.3 | | 3.99E-136 | |
| B | B | NME1 | -0.31 | | 5.17E-136 | |
| B | B | PIKFYVE | 0.34 | | 5.19E-136 | |
| B | B | FCGRT | -0.45 | | 1.26E-135 | |
| B | B | SPINK1 | -1.33 | | 1.31E-135 | |
| B | B | S100P | -1.16 | | 1.72E-135 | |
| B | B | ATF3 | -0.58 | | 2.14E-135 | |
| B | B | SEC61A1 | -0.27 | | 8.83E-135 | |
| B | B | CREG1 | -0.31 | | 2.46E-134 | |
| B | B | FNDC3B | -0.28 | | 3.52E-134 | |
| B | B | ATP1B1 | -0.4 | | 3.67E-134 | |
| B | B | VKORC1 | -0.31 | | 4.33E-134 | |
| B | B | CANX | -0.3 | | 1.79E-133 | |
| B | B | TNFRSF4 | -0.78 | | 3.03E-133 | |
| B | B | MRPS34 | -0.29 | | 4.92E-133 | |
| B | B | SPATS2L | -0.28 | | 5.23E-133 | |
| B | B | ARL4C | -0.49 | | 6.36E-133 | |
| B | B | VAMP5 | -0.4 | | 8.42E-133 | |
| B | B | WDR74 | 0.57 | | 9.09E-133 | |
| B | B | COMTD1 | -0.26 | | 1.22E-132 | |
| B | B | TNFRSF18 | -0.6 | | 1.59E-132 | |
| B | B | NDUFA1 | -0.39 | | 1.69E-132 | |
| B | B | AP2S1 | -0.4 | | 7.35E-132 | |
| B | B | SMIM22 | -0.59 | | 1.65E-131 | |
| B | B | RGS19 | 0.42 | | 2.71E-131 | |
| B | B | PCBD1 | -0.29 | | 4.03E-131 | |
| B | B | SEM1 | -0.4 | | 5.60E-131 | |
| B | B | HEXB | -0.29 | | 6.38E-131 | |
| B | B | LDLRAD4 | 0.44 | | 7.47E-131 | |
| B | B | SDC4 | -0.36 | | 1.05E-130 | |
| B | B | DUSP5 | -0.55 | | 1.88E-130 | |
| B | B | LAT | -0.46 | | 5.38E-130 | |
| B | B | BIK | -0.32 | | 3.21E-129 | |
| B | B | PRDX2 | -0.35 | | 3.36E-129 | |
| B | B | COX6B1 | -0.42 | | 4.32E-129 | |
| B | B | TMSB10 | -0.46 | | 1.14E-128 | |
| B | B | NDUFB3 | -0.32 | | 1.26E-128 | |
| B | B | MPST | -0.26 | | 5.47E-128 | |
| B | B | TSC22D1 | -0.57 | | 5.95E-128 | |
| B | B | PPA1 | -0.46 | | 7.92E-128 | |
| B | B | DNPH1 | -0.33 | | 8.07E-128 | |
| B | B | TCEAL9 | -0.33 | | 8.63E-128 | |
| B | B | ALG5 | -0.27 | | 9.14E-128 | |
| B | B | TOB1 | -0.38 | | 1.43E-127 | |
| B | B | IL2RB | -0.48 | | 4.48E-127 | |
| B | B | EFHD2 | -0.34 | | 5.46E-127 | |
| B | B | CD8A | -0.76 | | 6.72E-127 | |
| B | B | LAG3 | -0.57 | | 7.21E-127 | |
| B | B | SKIL | 0.47 | | 1.30E-126 | |
| B | B | ATP5MPL | -0.39 | | 1.51E-126 | |
| B | B | BEX3 | -0.34 | | 1.88E-126 | |
| B | B | STX11 | -0.4 | | 3.52E-126 | |
| B | B | GSTO1 | -0.45 | | 4.05E-126 | |
| B | B | SOX4 | -0.49 | | 5.60E-126 | |
| B | B | AKAP13 | -0.4 | | 5.75E-126 | |
| B | B | PRKCB | 0.4 | | 5.82E-126 | |
| B | B | ID1 | -0.63 | | 6.88E-126 | |
| B | B | STOM | -0.44 | | 1.22E-125 | |
| B | B | IFT57 | 0.5 | | 1.40E-125 | |
| B | B | DDX21 | 0.52 | | 1.92E-125 | |
| B | B | PLD4 | 0.27 | | 1.96E-125 | |
| B | B | IQGAP2 | -0.29 | | 2.18E-125 | |
| B | B | COMT | -0.28 | | 2.57E-125 | |
| B | B | DNAJB9 | -0.51 | | 5.19E-125 | |
| B | B | GUK1 | -0.36 | | 5.87E-125 | |
| B | B | SELL | 0.45 | | 7.90E-125 | |
| B | B | ALG13 | 0.59 | | 2.69E-124 | |
| B | B | VMP1 | -0.36 | | 4.26E-124 | |
| B | B | NAP1L1 | 0.32 | | 5.71E-124 | |
| B | B | TMEM176B | -0.5 | | 6.67E-124 | |
| B | B | LIMS1 | -0.37 | | 1.49E-123 | |
| B | B | LGALS4 | -0.72 | | 1.94E-123 | |
| B | B | COX6C | -0.44 | | 2.18E-123 | |
| B | B | CUTA | -0.4 | | 8.17E-123 | |
| B | B | TMEM123 | 0.61 | | 8.26E-123 | |
| B | B | LCK | -0.52 | | 1.33E-122 | |
| B | B | S100A16 | -0.4 | | 3.01E-122 | |
| B | B | NDUFA12 | -0.32 | | 6.31E-122 | |
| B | B | SPCS1 | -0.53 | | 1.13E-121 | |
| B | B | TMEM205 | -0.26 | | 1.33E-121 | |
| B | B | LAMTOR2 | -0.3 | | 2.19E-121 | |
| B | B | ALDH2 | -0.33 | | 2.73E-121 | |
| B | B | ATP5F1D | -0.41 | | 4.00E-121 | |
| B | B | BID | -0.31 | | 4.17E-121 | |
| B | B | P2RX5 | 0.3 | | 4.18E-121 | |
| B | B | MRPL57 | -0.29 | | 4.29E-121 | |
| B | B | ARRDC2 | 0.41 | | 4.57E-121 | |
| B | B | TOMM7 | 0.29 | | 5.52E-121 | |
| B | B | CRELD2 | -0.33 | | 9.13E-121 | |
| B | B | DYNLL1 | -0.36 | | 1.15E-120 | |
| B | B | IGFBP7 | -1.97 | | 1.62E-120 | |
| B | B | NAMPT | -0.45 | | 2.03E-120 | |
| B | B | EMP1 | -0.64 | | 2.43E-120 | |
| B | B | ICOS | -0.52 | | 2.78E-120 | |
| B | B | SF1 | 0.38 | | 3.29E-120 | |
| B | B | LCN2 | -0.91 | | 1.69E-119 | |
| B | B | TMEM147 | -0.3 | | 1.98E-119 | |
| B | B | ARL6IP5 | -0.38 | | 2.16E-119 | |
| B | B | CLTB | -0.32 | | 2.46E-119 | |
| B | B | ACTG1 | -0.49 | | 4.15E-119 | |
| B | B | GNLY | -1.76 | | 4.86E-119 | |
| B | B | CTSW | -0.62 | | 7.83E-119 | |
| B | B | ATRAID | -0.3 | | 8.93E-119 | |
| B | B | GBP2 | -0.36 | | 3.09E-118 | |
| B | B | RHBDD2 | -0.36 | | 4.66E-118 | |
| B | B | NAPRT | -0.34 | | 5.94E-118 | |
| B | B | GSTK1 | -0.34 | | 7.12E-118 | |
| B | B | TUBA1A | 0.33 | | 1.00E-117 | |
| B | B | C4orf48 | -0.33 | | 1.97E-117 | |
| B | B | G0S2 | -1.63 | | 2.32E-117 | |
| B | B | PDIA3 | -0.36 | | 2.85E-117 | |
| B | B | H2AFY | -0.32 | | 4.83E-117 | |
| B | B | NUCB1 | -0.27 | | 6.67E-117 | |
| B | B | PFN1 | -0.52 | | 9.14E-117 | |
| B | B | TALDO1 | -0.32 | | 2.62E-116 | |
| B | B | AQP3 | -0.34 | | 3.08E-116 | |
| B | B | SEC11C | -0.69 | | 3.09E-116 | |
| B | B | BRI3 | -0.3 | | 1.07E-115 | |
| B | B | CLU | -0.7 | | 1.60E-115 | |
| B | B | QSOX1 | -0.25 | | 1.93E-115 | |
| B | B | TMEM50A | -0.37 | | 2.73E-115 | |
| B | B | KLF2 | 0.41 | | 3.46E-115 | |
| B | B | MANF | -0.44 | | 8.99E-115 | |
| B | B | SOD2 | -1.46 | | 1.25E-114 | |
| B | B | RPN2 | -0.37 | | 2.31E-114 | |
| B | B | TIGIT | -0.61 | | 4.31E-114 | |
| B | B | UBE2J1 | -0.37 | | 5.14E-114 | |
| B | B | NDUFB1 | -0.34 | | 5.81E-114 | |
| B | B | FIS1 | -0.31 | | 9.08E-114 | |
| B | B | FKBP1A | -0.47 | | 1.07E-113 | |
| B | B | PLEC | -0.3 | | 1.11E-113 | |
| B | B | TMEM173 | -0.35 | | 2.05E-113 | |
| B | B | EMP2 | -0.27 | | 3.27E-113 | |
| B | B | ATP5F1E | -0.32 | | 4.05E-113 | |
| B | B | ACTN1 | -0.26 | | 6.54E-113 | |
| B | B | TFF1 | -2.12 | | 6.77E-113 | |
| B | B | NDUFAB1 | -0.31 | | 8.35E-113 | |
| B | B | CCL20 | -1.2 | | 8.63E-113 | |
| B | B | NR4A1 | 0.51 | | 9.14E-113 | |
| B | B | PYCARD | -0.27 | | 9.63E-113 | |
| B | B | TAGLN2 | 0.33 | | 1.17E-112 | |
| B | B | PRKCH | -0.38 | | 1.98E-112 | |
| B | B | EPCAM | -0.51 | | 2.26E-112 | |
| B | B | GZMK | -0.97 | | 4.78E-112 | |
| B | B | PIGR | -0.89 | | 1.34E-111 | |
| B | B | GNG5 | -0.37 | | 1.38E-111 | |
| B | B | TIMM13 | -0.31 | | 1.72E-111 | |
| B | B | PLA2G16 | -0.28 | | 2.44E-111 | |
| B | B | CD6 | -0.45 | | 2.73E-111 | |
| B | B | CLTA | -0.32 | | 3.02E-111 | |
| B | B | CAPN2 | -0.27 | | 3.43E-111 | |
| B | B | COX5B | -0.41 | | 3.99E-111 | |
| B | B | MINOS1 | -0.38 | | 4.18E-111 | |
| B | B | FAM129A | -0.35 | | 9.93E-111 | |
| B | B | NQO1 | -0.43 | | 1.09E-110 | |
| B | B | PPIF | -0.4 | | 1.46E-110 | |
| B | B | RPN1 | -0.25 | | 1.96E-110 | |
| B | B | NAA38 | -0.27 | | 3.05E-110 | |
| B | B | PRDX1 | -0.44 | | 3.63E-110 | |
| B | B | VSIG2 | -0.42 | | 6.38E-110 | |
| B | B | HSBP1 | -0.32 | | 6.46E-110 | |
| B | B | CMC1 | -0.4 | | 6.94E-110 | |
| B | B | PLIN2 | -0.47 | | 7.90E-110 | |
| B | B | TMEM219 | -0.28 | | 8.52E-110 | |
| B | B | IL1RN | -0.83 | | 8.87E-110 | |
| B | B | CLDN4 | -0.63 | | 9.26E-110 | |
| B | B | IL1B | -1.75 | | 1.01E-109 | |
| B | B | TMEM176A | -0.34 | | 1.14E-109 | |
| B | B | PDIA6 | -0.41 | | 1.37E-109 | |
| B | B | CHMP2A | -0.3 | | 2.02E-109 | |
| B | B | PRF1 | -0.68 | | 2.16E-109 | |
| B | B | TNFRSF12A | -0.32 | | 3.38E-109 | |
| B | B | STARD10 | -0.32 | | 4.05E-109 | |
| B | B | CD96 | -0.48 | | 4.61E-109 | |
| B | B | GADD45B | -0.65 | | 5.45E-109 | |
| B | B | SESN3 | 0.3 | | 8.46E-109 | |
| B | B | TMEM243 | 0.48 | | 8.84E-109 | |
| B | B | DUSP4 | -0.49 | | 8.86E-109 | |
| B | B | LINC01871 | -0.57 | | 1.13E-108 | |
| B | B | PTP4A2 | -0.27 | | 1.31E-108 | |
| B | B | BTK | 0.3 | | 1.72E-108 | |
| B | B | GIMAP7 | -0.46 | | 2.38E-108 | |
| B | B | REEP5 | -0.32 | | 4.16E-108 | |
| B | B | RNF149 | -0.3 | | 6.12E-108 | |
| B | B | CHMP4B | -0.26 | | 9.79E-108 | |
| B | B | EIF1AY | 0.5 | | 1.87E-107 | |
| B | B | ERN1 | -0.25 | | 2.28E-107 | |
| B | B | METTL26 | -0.25 | | 2.61E-107 | |
| B | B | ATP5MC1 | -0.34 | | 3.54E-107 | |
| B | B | SOCS3 | -0.83 | | 3.62E-107 | |
| B | B | ALDOA | -0.37 | | 4.19E-107 | |
| B | B | ATP5F1C | -0.32 | | 6.30E-107 | |
| B | B | AHR | -0.26 | | 7.31E-107 | |
| B | B | CFD | -0.79 | | 9.69E-107 | |
| B | B | PKM | -0.46 | | 1.07E-106 | |
| B | B | HSPA1B | -0.75 | | 1.40E-106 | |
| B | B | S100A14 | -0.52 | | 1.46E-106 | |
| B | B | VEGFA | -0.26 | | 3.62E-106 | |
| B | B | NPDC1 | -0.38 | | 4.44E-106 | |
| B | B | TUBA1C | -0.34 | | 4.78E-106 | |
| B | B | NCOA7 | -0.34 | | 5.43E-106 | |
| B | B | BCAS4 | 0.27 | | 6.57E-106 | |
| B | B | ANXA10 | -0.58 | | 7.48E-106 | |
| B | B | JPT1 | -0.38 | | 9.23E-106 | |
| B | B | HIST1H1C | -0.56 | | 1.01E-105 | |
| B | B | ETHE1 | -0.26 | | 1.18E-105 | |
| B | B | SMCHD1 | 0.46 | | 1.18E-105 | |
| B | B | MT1G | -0.98 | | 2.17E-105 | |
| B | B | ZNF775 | 0.35 | | 7.67E-105 | |
| B | B | EIF3E | 0.28 | | 1.16E-104 | |
| B | B | ANXA4 | -0.42 | | 1.24E-104 | |
| B | B | LMAN1 | -0.32 | | 1.71E-104 | |
| B | B | LMAN2 | -0.33 | | 1.84E-104 | |
| B | B | GZMH | -0.81 | | 2.27E-104 | |
| B | B | ABRACL | -0.29 | | 2.42E-104 | |
| B | B | C12orf75 | -0.4 | | 2.73E-104 | |
| B | B | GPX2 | -0.44 | | 3.12E-104 | |
| B | B | TM9SF3 | -0.25 | | 4.67E-104 | |
| B | B | MT1F | -0.29 | | 1.17E-103 | |
| B | B | SLIRP | -0.3 | | 1.29E-103 | |
| B | B | SPTBN1 | -0.32 | | 7.94E-103 | |
| B | B | NDFIP1 | -0.26 | | 2.79E-102 | |
| B | B | ANAPC11 | -0.3 | | 3.82E-102 | |
| B | B | AURKAIP1 | -0.31 | | 4.91E-102 | |
| B | B | APMAP | -0.29 | | 5.55E-102 | |
| B | B | IFI27L2 | -0.28 | | 5.70E-102 | |
| B | B | PSAP | -0.64 | | 1.04E-101 | |
| B | B | C9orf16 | -0.34 | | 1.10E-101 | |
| B | B | NEDD8 | -0.3 | | 3.07E-101 | |
| B | B | NDUFS5 | -0.32 | | 4.97E-101 | |
| B | B | CYC1 | -0.3 | | 6.76E-101 | |
| B | B | CCND2 | -0.38 | | 9.86E-101 | |
| B | B | GPSM3 | 0.38 | | 2.42E-100 | |
| B | B | CYB5A | -0.29 | | 3.46E-100 | |
| B | B | HSPA5 | -0.48 | | 4.10E-100 | |
| B | B | CD320 | -0.32 | | 4.21E-100 | |
| B | B | OSTC | -0.28 | | 6.82E-100 | |
| B | B | HSPA1A | -1.01 | | 8.69E-100 | |
| B | B | NDUFS7 | -0.28 | | 1.01E-99 | |
| B | B | NDUFB4 | -0.33 | | 1.21E-99 | |
| B | B | EPAS1 | -0.35 | | 2.65E-99 | |
| B | B | RHOH | 0.45 | | 2.86E-99 | |
| B | B | RAB1A | -0.26 | | 4.50E-99 | |
| B | B | ANKRD44 | 0.33 | | 5.19E-99 | |
| B | B | AC020656.1 | -0.51 | | 1.10E-98 | |
| B | B | FABP5 | -0.67 | | 1.23E-98 | |
| B | B | RILPL2 | 0.41 | | 1.56E-98 | |
| B | B | TCF4 | 0.36 | | 1.95E-98 | |
| B | B | SLC3A2 | -0.31 | | 2.87E-98 | |
| B | B | PTPRCAP | 0.27 | | 3.65E-98 | |
| B | B | TACSTD2 | -0.36 | | 4.18E-98 | |
| B | B | CLDN18 | -0.45 | | 5.42E-98 | |
| B | B | CD53 | 0.31 | | 1.33E-97 | |
| B | B | TINAGL1 | -0.27 | | 2.80E-97 | |
| B | B | BATF | -0.5 | | 2.89E-97 | |
| B | B | PPDPF | -0.43 | | 3.34E-97 | |
| B | B | TXNDC15 | -0.31 | | 3.90E-97 | |
| B | B | GNA15 | -0.29 | | 4.61E-97 | |
| B | B | GNB5 | 0.33 | | 5.35E-97 | |
| B | B | GYG1 | -0.27 | | 6.62E-97 | |
| B | B | GMDS | -0.28 | | 1.32E-96 | |
| B | B | AAK1 | -0.28 | | 5.46E-96 | |
| B | B | PDE4D | -0.33 | | 5.51E-96 | |
| B | B | REX1BD | -0.26 | | 6.04E-96 | |
| B | B | HBEGF | -0.25 | | 9.86E-96 | |
| B | B | SERPINA1 | -0.54 | | 1.10E-95 | |
| B | B | TMC5 | -0.33 | | 1.25E-95 | |
| B | B | ATP5MG | -0.28 | | 1.57E-95 | |
| B | B | EIF3L | 0.34 | | 2.61E-95 | |
| B | B | ERLEC1 | -0.29 | | 2.66E-95 | |
| B | B | YWHAZ | 0.3 | | 3.11E-95 | |
| B | B | TPM2 | -0.48 | | 3.21E-95 | |
| B | B | MTRNR2L8 | -0.55 | | 3.48E-95 | |
| B | B | GALM | -0.25 | | 5.81E-95 | |
| B | B | CXCL1 | -0.83 | | 6.19E-95 | |
| B | B | GPRC5A | -0.29 | | 1.15E-94 | |
| B | B | VAMP8 | -0.36 | | 1.27E-94 | |
| B | B | ATP5PD | -0.28 | | 2.05E-94 | |
| B | B | RAB27A | -0.27 | | 3.50E-94 | |
| B | B | TNFRSF25 | -0.35 | | 4.09E-94 | |
| B | B | SELPLG | -0.3 | | 5.37E-94 | |
| B | B | ETFB | -0.29 | | 6.09E-94 | |
| B | B | KRT7 | -0.37 | | 1.32E-93 | |
| B | B | TNFSF10 | -0.28 | | 1.56E-93 | |
| B | B | TTC39C | -0.32 | | 2.49E-93 | |
| B | B | HNRNPDL | 0.28 | | 2.71E-93 | |
| B | B | RALA | -0.26 | | 3.39E-93 | |
| B | B | DYNLRB1 | -0.27 | | 4.48E-93 | |
| B | B | BLOC1S1 | -0.3 | | 5.58E-93 | |
| B | B | CHST12 | -0.34 | | 5.89E-93 | |
| B | B | SLA | -0.4 | | 1.13E-92 | |
| B | B | NDUFB2 | -0.31 | | 1.14E-92 | |
| B | B | ROMO1 | -0.29 | | 5.33E-92 | |
| B | B | TFF2 | -1.26 | | 6.17E-92 | |
| B | B | HNRNPLL | -0.26 | | 9.16E-92 | |
| B | B | MIF | -0.35 | | 9.79E-92 | |
| B | B | SELENOP | -0.47 | | 1.59E-91 | |
| B | B | JAML | -0.36 | | 1.73E-91 | |
| B | B | SFN | -0.34 | | 3.37E-91 | |
| B | B | WARS | -0.28 | | 3.54E-91 | |
| B | B | LDHB | -0.38 | | 4.90E-91 | |
| B | B | NDRG1 | -0.29 | | 7.42E-91 | |
| B | B | AHNAK | -0.27 | | 7.87E-91 | |
| B | B | TMEM156 | 0.37 | | 1.41E-90 | |
| B | B | MYH9 | -0.31 | | 1.97E-90 | |
| B | B | RBM39 | 0.31 | | 5.00E-90 | |
| B | B | METRNL | -0.27 | | 5.64E-90 | |
| B | B | SCAND1 | -0.27 | | 1.02E-89 | |
| B | B | RPL36A | 0.27 | | 1.37E-89 | |
| B | B | RNASE1 | -0.56 | | 1.68E-89 | |
| B | B | PRDM2 | 0.38 | | 2.14E-89 | |
| B | B | COPE | -0.32 | | 2.27E-89 | |
| B | B | ARL6IP4 | -0.28 | | 2.83E-89 | |
| B | B | ANPEP | -0.34 | | 3.47E-89 | |
| B | B | SPN | -0.3 | | 6.76E-89 | |
| B | B | HNRNPK | 0.29 | | 8.48E-89 | |
| B | B | HSPG2 | -0.42 | | 1.14E-88 | |
| B | B | DRAM2 | 0.47 | | 1.86E-88 | |
| B | B | NFKBIA | -0.61 | | 3.54E-88 | |
| B | B | JSRP1 | -0.51 | | 5.02E-88 | |
| B | B | COMMD6 | 0.26 | | 7.36E-88 | |
| B | B | ERGIC3 | -0.31 | | 8.61E-88 | |
| B | B | CACYBP | -0.32 | | 1.32E-87 | |
| B | B | TRAT1 | -0.41 | | 1.36E-87 | |
| B | B | ETS1 | -0.34 | | 1.56E-87 | |
| B | B | DOK2 | -0.33 | | 1.88E-87 | |
| B | B | ATP5MF | -0.31 | | 3.46E-87 | |
| B | B | DDIT4 | -0.48 | | 8.92E-87 | |
| B | B | NSMCE1 | -0.26 | | 1.48E-86 | |
| B | B | HLA-DOB | 0.34 | | 1.53E-86 | |
| B | B | LRRFIP1 | 0.39 | | 3.33E-86 | |
| B | B | SERPINB1 | -0.38 | | 4.00E-86 | |
| B | B | KMT2E | 0.33 | | 4.31E-86 | |
| B | B | NDUFA11 | -0.29 | | 6.09E-86 | |
| B | B | CD68 | -0.43 | | 6.88E-86 | |
| B | B | SLA2 | -0.38 | | 8.27E-86 | |
| B | B | PRELID1 | -0.31 | | 1.82E-85 | |
| B | B | MRPL41 | -0.26 | | 2.07E-85 | |
| B | B | SELENOM | -0.4 | | 4.03E-85 | |
| B | B | SNX9 | 0.43 | | 1.24E-84 | |
| B | B | PKIG | 0.32 | | 2.04E-84 | |
| B | B | SLC2A3 | 0.35 | | 5.44E-84 | |
| B | B | SPCS2 | -0.44 | | 1.21E-83 | |
| B | B | IDS | 0.32 | | 1.39E-83 | |
| B | B | CTSE | -0.38 | | 1.71E-83 | |
| B | B | CD8B | -0.48 | | 3.78E-83 | |
| B | B | GABARAPL1 | -0.3 | | 4.50E-83 | |
| B | B | PLSCR1 | -0.26 | | 4.74E-83 | |
| B | B | HOXB2 | -0.29 | | 4.80E-83 | |
| B | B | TNFRSF17 | -0.34 | | 5.54E-83 | |
| B | B | MCL1 | -0.34 | | 1.32E-82 | |
| B | B | PDCL3 | -0.34 | | 4.20E-82 | |
| B | B | ICOSLG | 0.36 | | 4.51E-82 | |
| B | B | FLNA | -0.34 | | 4.89E-82 | |
| B | B | GPR171 | -0.39 | | 8.28E-82 | |
| B | B | PHF20 | 0.37 | | 1.20E-81 | |
| B | B | TESC | -0.31 | | 1.87E-81 | |
| B | B | LY96 | -0.29 | | 2.53E-81 | |
| B | B | PAXX | -0.27 | | 6.62E-81 | |
| B | B | RGS1 | -0.52 | | 1.03E-80 | |
| B | B | OASL | -0.29 | | 1.80E-80 | |
| B | B | IFNG | -0.71 | | 3.91E-80 | |
| B | B | S100A9 | -1.43 | | 5.06E-80 | |
| B | B | ARL14 | -0.36 | | 7.96E-80 | |
| B | B | NPC2 | -0.67 | | 8.67E-80 | |
| B | B | PARK7 | -0.31 | | 2.08E-79 | |
| B | B | PNP | -0.28 | | 2.35E-79 | |
| B | B | STX5 | 0.42 | | 4.36E-79 | |
| B | B | CD81 | -0.27 | | 6.51E-79 | |
| B | B | MT-CO1 | -0.78 | | 8.10E-79 | |
| B | B | ID3 | 0.39 | | 1.67E-78 | |
| B | B | GK | -0.29 | | 2.96E-78 | |
| B | B | SYTL3 | -0.36 | | 3.35E-78 | |
| B | B | CA2 | -0.7 | | 3.91E-78 | |
| B | B | RNF213 | -0.27 | | 3.94E-78 | |
| B | B | ELF1 | 0.37 | | 4.14E-78 | |
| B | B | HIST1H2BK | -0.39 | | 4.63E-78 | |
| B | B | SC5D | 0.42 | | 4.81E-78 | |
| B | B | COX7B | -0.33 | | 1.86E-77 | |
| B | B | ENO1 | -0.38 | | 2.09E-77 | |
| B | B | ICAM1 | -0.43 | | 3.39E-76 | |
| B | B | PSCA | -0.97 | | 5.44E-76 | |
| B | B | GIMAP5 | -0.29 | | 6.23E-76 | |
| B | B | AIF1 | -0.73 | | 8.41E-76 | |
| B | B | RALGPS2 | 0.31 | | 1.17E-75 | |
| B | B | CXCR3 | -0.39 | | 3.45E-75 | |
| B | B | TAP1 | -0.25 | | 7.96E-75 | |
| B | B | NCF1 | 0.37 | | 1.41E-74 | |
| B | B | PIK3R1 | -0.35 | | 2.53E-74 | |
| B | B | SGK1 | -0.39 | | 3.17E-74 | |
| B | B | GATA3 | -0.31 | | 3.84E-74 | |
| B | B | HES1 | -0.7 | | 2.49E-73 | |
| B | B | SWAP70 | 0.3 | | 2.64E-73 | |
| B | B | GLIPR2 | -0.26 | | 3.16E-73 | |
| B | B | PSMA7 | -0.29 | | 1.99E-72 | |
| B | B | TMED9 | -0.26 | | 2.98E-72 | |
| B | B | MAPK1IP1L | 0.36 | | 3.22E-72 | |
| B | B | APOE | -1.33 | | 3.77E-72 | |
| B | B | ZAP70 | -0.27 | | 5.73E-72 | |
| B | B | CD40 | 0.35 | | 1.36E-71 | |
| B | B | MZB1 | -1.67 | | 1.67E-71 | |
| B | B | UBL5 | -0.26 | | 3.28E-71 | |
| B | B | CD5 | -0.27 | | 3.30E-71 | |
| B | B | EID1 | -0.28 | | 7.90E-71 | |
| B | B | NDUFB9 | -0.26 | | 6.75E-70 | |
| B | B | GLUD1 | -0.26 | | 7.14E-69 | |
| B | B | IGFBP4 | -0.58 | | 7.53E-69 | |
| B | B | CD38 | -0.26 | | 9.06E-69 | |
| B | B | GMFG | -0.34 | | 1.21E-68 | |
| B | B | RNF130 | -0.25 | | 3.23E-68 | |
| B | B | GRN | -0.33 | | 6.58E-68 | |
| B | B | CAPZB | -0.28 | | 1.17E-67 | |
| B | B | RPS4Y1 | 0.34 | | 2.58E-67 | |
| B | B | TAGAP | 0.3 | | 6.15E-67 | |
| B | B | RBPJ | -0.25 | | 7.25E-67 | |
| B | B | STAT4 | -0.34 | | 3.58E-66 | |
| B | B | MTRNR2L2 | -0.71 | | 3.07E-65 | |
| B | B | ALOX5AP | -0.6 | | 5.55E-65 | |
| B | B | RGS2 | 0.46 | | 7.27E-65 | |
| B | B | NDUFB11 | -0.26 | | 1.15E-64 | |
| B | B | RNF19A | -0.25 | | 1.28E-64 | |
| B | B | CCDC85B | -0.25 | | 1.88E-64 | |
| B | B | SOD1 | -0.29 | | 2.66E-64 | |
| B | B | FMNL1 | 0.31 | | 4.87E-64 | |
| B | B | MTRNR2L12 | -0.46 | | 4.09E-63 | |
| B | B | DUSP6 | -0.26 | | 1.31E-62 | |
| B | B | SMAP2 | 0.36 | | 1.42E-62 | |
| B | B | MT-ND5 | -0.66 | | 3.75E-62 | |
| B | B | RNASET2 | 0.3 | | 6.68E-62 | |
| B | B | SYNGR2 | 0.41 | | 9.14E-62 | |
| B | B | APOBEC3G | -0.34 | | 1.33E-61 | |
| B | B | PPP2R5C | -0.32 | | 9.02E-61 | |
| B | B | PMAIP1 | 0.39 | | 9.46E-61 | |
| B | B | CLECL1 | 0.27 | | 2.43E-60 | |
| B | B | EGR1 | -0.52 | | 1.50E-59 | |
| B | B | ADGRE5 | 0.32 | | 1.85E-59 | |
| B | B | RSL24D1 | 0.37 | | 5.52E-59 | |
| B | B | HOPX | -0.4 | | 2.09E-58 | |
| B | B | CD70 | 0.27 | | 3.98E-58 | |
| B | B | COX7A2 | -0.25 | | 6.85E-58 | |
| B | B | DENND3 | 0.28 | | 8.26E-58 | |
| B | B | LTBP4 | -0.26 | | 3.14E-57 | |
| B | B | RARA | 0.3 | | 3.89E-57 | |
| B | B | ANKRD36C | -0.26 | | 4.84E-57 | |
| B | B | PNISR | 0.32 | | 5.90E-57 | |
| B | B | MED30 | 0.36 | | 7.41E-57 | |
| B | B | SERTAD1 | -0.29 | | 2.80E-56 | |
| B | B | GGA2 | 0.29 | | 3.98E-56 | |
| B | B | UQCR10 | -0.26 | | 1.27E-55 | |
| B | B | COL9A3 | 0.28 | | 1.41E-55 | |
| B | B | CLEC2B | -0.29 | | 7.28E-55 | |
| B | B | UCP2 | 0.36 | | 1.10E-54 | |
| B | B | KLF4 | -0.34 | | 1.58E-54 | |
| B | B | PGK1 | -0.33 | | 4.12E-54 | |
| B | B | LYAR | -0.25 | | 1.01E-53 | |
| B | B | PRNP | 0.34 | | 2.46E-53 | |
| B | B | RPL22L1 | 0.31 | | 3.97E-53 | |
| B | B | C9orf72 | 0.26 | | 9.72E-53 | |
| B | B | AC004687.1 | 0.26 | | 4.98E-52 | |
| B | B | OFD1 | 0.3 | | 1.49E-51 | |
| B | B | ARL4A | 0.31 | | 2.70E-51 | |
| B | B | TUT4 | 0.26 | | 2.32E-50 | |
| B | B | RNMT | 0.32 | | 7.35E-50 | |
| B | B | IL16 | 0.27 | | 3.85E-49 | |
| B | B | COTL1 | -0.44 | | 1.29E-48 | |
| B | B | TAF7 | 0.28 | | 4.11E-48 | |
| B | B | MTRNR2L1 | -0.27 | | 2.31E-47 | |
| B | B | CYSTM1 | -0.79 | | 7.93E-46 | |
| B | B | IDI1 | 0.37 | | 1.50E-45 | |
| B | B | ITGB2 | -0.28 | | 3.99E-45 | |
| B | B | TUBB | -0.31 | | 2.74E-44 | |
| B | B | CHMP1B | 0.43 | | 4.70E-44 | |
| B | B | NCL | 0.26 | | 5.31E-44 | |
| B | B | METTL21A | 0.27 | | 2.12E-43 | |
| B | B | C6orf48 | 0.26 | | 2.31E-43 | |
| B | B | MT-ND1 | -0.68 | | 5.05E-43 | |
| B | B | 7-Sep | 0.27 | | 7.57E-43 | |
| B | B | TRIM38 | 0.26 | | 9.41E-43 | |
| B | B | CD27 | -0.34 | | 9.50E-43 | |
| B | B | CLIC1 | -0.25 | | 4.13E-42 | |
| B | B | MGAT4A | -0.25 | | 7.30E-42 | |
| B | B | FAM177A1 | -0.26 | | 1.29E-41 | |
| B | B | DDX27 | 0.3 | | 2.79E-40 | |
| B | B | CNPPD1 | 0.31 | | 1.15E-39 | |
| B | B | KDM6B | 0.27 | | 1.30E-39 | |
| B | B | ATP2B1 | 0.29 | | 2.82E-39 | |
| B | B | RUBCN | 0.26 | | 3.26E-39 | |
| B | B | SP110 | 0.28 | | 5.25E-39 | |
| B | B | NR1H2 | 0.31 | | 3.95E-36 | |
| B | B | MT-ND2 | -0.58 | | 8.53E-36 | |
| B | B | CRIP2 | -0.34 | | 2.19E-35 | |
| B | B | LCP1 | -0.26 | | 5.97E-35 | |
| B | B | SPRY1 | -0.28 | | 4.63E-34 | |
| B | B | SLC39A3 | 0.28 | | 7.41E-34 | |
| B | B | ADPGK | 0.28 | | 2.24E-33 | |
| B | B | ARPC1B | -0.26 | | 5.24E-33 | |
| B | B | DERL3 | -0.96 | | 1.73E-32 | |
| B | B | SQSTM1 | -0.29 | | 1.81E-32 | |
| B | B | TNRC6B | 0.27 | | 2.44E-32 | |
| B | B | TXNIP | 0.27 | | 7.25E-32 | |
| B | B | ZMYM2 | 0.26 | | 1.44E-31 | |
| B | B | ZNF791 | 0.26 | | 1.60E-31 | |
| B | B | TCOF1 | 0.27 | | 1.53E-30 | |
| B | B | MT-ND4L | -0.43 | | 1.58E-30 | |
| B | B | MBP | 0.26 | | 1.94E-29 | |
| B | B | TYMP | -0.33 | | 2.03E-29 | |
| B | B | ST6GAL1 | 0.25 | | 3.24E-29 | |
| B | B | LST1 | -0.36 | | 5.31E-29 | |
| B | B | SYS1 | 0.29 | | 5.74E-29 | |
| B | B | SSH2 | 0.29 | | 1.08E-28 | |
| B | B | SLBP | 0.3 | | 1.66E-28 | |
| B | B | IFITM2 | -0.36 | | 1.95E-28 | |
| B | B | JCHAIN | -3.07 | | 2.18E-28 | |
| B | B | MT-CYB | -0.65 | | 3.23E-28 | |
| B | B | HSP90AA1 | -0.26 | | 5.97E-28 | |
| B | B | AC103591.3 | 0.3 | | 1.19E-27 | |
| B | B | TAF1D | 0.28 | | 1.52E-27 | |
| B | B | NGLY1 | 0.3 | | 3.14E-27 | |
| B | B | TLE1 | 0.31 | | 6.68E-27 | |
| B | B | PPP1CB | 0.26 | | 1.03E-25 | |
| B | B | MT-ATP6 | -0.69 | | 1.24E-25 | |
| B | B | PRKAR1A | 0.26 | | 1.30E-25 | |
| B | B | CHD1 | 0.25 | | 1.05E-24 | |
| B | B | PPM1K | 0.25 | | 1.20E-24 | |
| B | B | CDC37 | 0.31 | | 3.62E-22 | |
| B | B | PGC | -2.12 | | 7.68E-21 | |
| B | B | TTC3 | 0.31 | | 9.76E-21 | |
| B | B | CNPY3 | 0.27 | | 1.21E-20 | |
| B | B | ZFP36 | -0.39 | | 2.41E-20 | |
| B | B | PWP1 | 0.25 | | 3.77E-20 | |
| B | B | MT-CO2 | -0.72 | | 1.01E-19 | |
| B | B | PTP4A1 | 0.26 | | 3.87E-19 | |
| B | B | GOT1 | 0.26 | | 3.43E-16 | |
| B | B | FTL | -0.83 | | 5.42E-15 | |
| B | B | KLF3 | 0.29 | | 9.28E-15 | |
| B | B | PTPRC | -0.26 | | 6.74E-12 | |
| B | B | MT-ND4 | -0.6 | | 1.31E-08 | |
| B | B | YBX3 | 0.27 | | 9.05E-06 | |
| CD8T | CD8T | CCL5 | 1.4 | | 0 | |
| CD8T | CD8T | GZMK | 1.32 | | 0 | |
| CD8T | CD8T | NKG7 | 1.14 | | 0 | |
| CD8T | CD8T | CST7 | 1.08 | | 0 | |
| CD8T | CD8T | CD3D | 1.01 | | 0 | |
| CD8T | CD8T | CD8A | 0.99 | | 0 | |
| CD8T | CD8T | LEPROTL1 | 0.93 | | 0 | |
| CD8T | CD8T | CD7 | 0.91 | | 0 | |
| CD8T | CD8T | CD3E | 0.91 | | 0 | |
| CD8T | CD8T | FYN | 0.9 | | 0 | |
| CD8T | CD8T | PTPRC | 0.9 | | 0 | |
| CD8T | CD8T | GZMA | 0.88 | | 0 | |
| CD8T | CD8T | TNFAIP3 | 0.85 | | 0 | |
| CD8T | CD8T | CD2 | 0.84 | | 0 | |
| CD8T | CD8T | GZMM | 0.82 | | 0 | |
| CD8T | CD8T | HCST | 0.72 | | 0 | |
| CD8T | CD8T | CREM | 0.72 | | 0 | |
| CD8T | CD8T | SARAF | 0.7 | | 0 | |
| CD8T | CD8T | IL32 | 0.69 | | 0 | |
| CD8T | CD8T | CXCR4 | 0.65 | | 0 | |
| CD8T | CD8T | SRGN | 0.65 | | 0 | |
| CD8T | CD8T | BTG1 | 0.63 | | 0 | |
| CD8T | CD8T | HLA-A | 0.6 | | 0 | |
| CD8T | CD8T | ARHGDIB | 0.58 | | 0 | |
| CD8T | CD8T | B2M | 0.49 | | 0 | |
| CD8T | CD8T | TMSB4X | 0.41 | | 0 | |
| CD8T | CD8T | HLA-C | 0.4 | | 0 | |
| CD8T | CD8T | NPC2 | -1.01 | | 0 | |
| CD8T | CD8T | HLA-DRA | -1.58 | | 0 | |
| CD8T | CD8T | CST3 | -1.64 | | 0 | |
| CD8T | CD8T | CD79A | -1.36 | | 3.23E-299 | |
| CD8T | CD8T | HLA-B | 0.38 | | 5.46E-298 | |
| CD8T | CD8T | MALAT1 | 0.33 | | 3.25E-287 | |
| CD8T | CD8T | HLA-DRB1 | -1.13 | | 1.43E-267 | |
| CD8T | CD8T | CTSH | -0.61 | | 6.50E-267 | |
| CD8T | CD8T | CD74 | -1.15 | | 5.30E-266 | |
| CD8T | CD8T | HLA-DQB1 | -0.98 | | 2.16E-265 | |
| CD8T | CD8T | LDHA | 0.66 | | 6.15E-265 | |
| CD8T | CD8T | CD8B | 0.7 | | 5.45E-263 | |
| CD8T | CD8T | FTL | -1.13 | | 7.79E-263 | |
| CD8T | CD8T | ANXA1 | 0.93 | | 5.01E-258 | |
| CD8T | CD8T | GRN | -0.63 | | 2.39E-256 | |
| CD8T | CD8T | HLA-DMA | -0.69 | | 2.46E-254 | |
| CD8T | CD8T | CD96 | 0.69 | | 1.33E-246 | |
| CD8T | CD8T | HLA-DQA1 | -1.03 | | 7.81E-238 | |
| CD8T | CD8T | TRAT1 | 0.68 | | 1.33E-236 | |
| CD8T | CD8T | GZMH | 0.84 | | 7.80E-236 | |
| CD8T | CD8T | IL7R | 0.82 | | 8.46E-235 | |
| CD8T | CD8T | CD3G | 0.64 | | 4.31E-234 | |
| CD8T | CD8T | FCGRT | -0.57 | | 1.08E-229 | |
| CD8T | CD8T | LYZ | -1.92 | | 2.81E-227 | |
| CD8T | CD8T | TIMP1 | -2.05 | | 7.32E-225 | |
| CD8T | CD8T | KLRB1 | 0.96 | | 4.74E-224 | |
| CD8T | CD8T | SRSF7 | 0.64 | | 1.76E-220 | |
| CD8T | CD8T | RGCC | 0.68 | | 2.05E-220 | |
| CD8T | CD8T | PPP2R5C | 0.73 | | 2.29E-220 | |
| CD8T | CD8T | ITM2A | 0.74 | | 1.58E-219 | |
| CD8T | CD8T | CTSW | 0.68 | | 4.33E-218 | |
| CD8T | CD8T | ZFP36L2 | 0.72 | | 2.49E-217 | |
| CD8T | CD8T | IFITM3 | -1.25 | | 2.03E-215 | |
| CD8T | CD8T | TRIB1 | -0.62 | | 2.40E-214 | |
| CD8T | CD8T | IER3 | -0.99 | | 6.22E-213 | |
| CD8T | CD8T | LCK | 0.61 | | 1.66E-212 | |
| CD8T | CD8T | CD83 | -0.85 | | 3.85E-206 | |
| CD8T | CD8T | PRDX4 | -0.88 | | 4.78E-205 | |
| CD8T | CD8T | PRF1 | 0.74 | | 2.93E-204 | |
| CD8T | CD8T | HLA-DRB5 | -0.97 | | 2.55E-203 | |
| CD8T | CD8T | LINC01871 | 0.7 | | 5.48E-203 | |
| CD8T | CD8T | CD63 | -0.85 | | 4.07E-202 | |
| CD8T | CD8T | ATOX1 | -0.44 | | 2.15E-200 | |
| CD8T | CD8T | KLF4 | -0.74 | | 2.34E-200 | |
| CD8T | CD8T | NR4A1 | -0.55 | | 1.63E-199 | |
| CD8T | CD8T | GPR171 | 0.64 | | 2.45E-194 | |
| CD8T | CD8T | FAM177A1 | 0.72 | | 1.78E-193 | |
| CD8T | CD8T | YPEL5 | 0.46 | | 1.86E-192 | |
| CD8T | CD8T | GSN | -1 | | 7.20E-192 | |
| CD8T | CD8T | DDX5 | 0.4 | | 2.61E-188 | |
| CD8T | CD8T | TIGIT | 0.74 | | 2.13E-187 | |
| CD8T | CD8T | CTSB | -0.91 | | 2.95E-186 | |
| CD8T | CD8T | PSAP | -0.69 | | 5.99E-185 | |
| CD8T | CD8T | CALM2 | 0.42 | | 1.68E-184 | |
| CD8T | CD8T | IFI27 | -1.24 | | 3.78E-184 | |
| CD8T | CD8T | STAT4 | 0.63 | | 6.74E-182 | |
| CD8T | CD8T | XCL2 | 0.92 | | 1.62E-181 | |
| CD8T | CD8T | PDLIM1 | -0.51 | | 4.55E-181 | |
| CD8T | CD8T | MEF2C | -0.54 | | 1.94E-180 | |
| CD8T | CD8T | SRSF2 | 0.53 | | 3.22E-180 | |
| CD8T | CD8T | SH2D2A | 0.65 | | 5.55E-180 | |
| CD8T | CD8T | IFNGR2 | -0.32 | | 4.36E-177 | |
| CD8T | CD8T | HLA-DQA2 | -0.74 | | 8.45E-176 | |
| CD8T | CD8T | CDKN1A | -0.56 | | 1.03E-175 | |
| CD8T | CD8T | SPOCK2 | 0.57 | | 1.60E-175 | |
| CD8T | CD8T | ASAH1 | -0.44 | | 5.15E-175 | |
| CD8T | CD8T | SPINT2 | -0.49 | | 4.98E-173 | |
| CD8T | CD8T | GZMB | 0.81 | | 1.51E-172 | |
| CD8T | CD8T | IFNG | 0.91 | | 2.03E-172 | |
| CD8T | CD8T | FKBP2 | -0.6 | | 1.25E-171 | |
| CD8T | CD8T | CALM1 | 0.4 | | 7.35E-171 | |
| CD8T | CD8T | CD52 | 0.44 | | 8.11E-169 | |
| CD8T | CD8T | RRBP1 | -0.34 | | 4.90E-168 | |
| CD8T | CD8T | ALDH2 | -0.4 | | 5.87E-167 | |
| CD8T | CD8T | LGMN | -0.45 | | 2.06E-165 | |
| CD8T | CD8T | CD59 | -0.5 | | 4.38E-164 | |
| CD8T | CD8T | AKR1A1 | -0.3 | | 1.19E-163 | |
| CD8T | CD8T | PIK3R1 | 0.65 | | 3.08E-163 | |
| CD8T | CD8T | CTSZ | -0.39 | | 2.66E-160 | |
| CD8T | CD8T | RAB13 | -0.4 | | 5.99E-160 | |
| CD8T | CD8T | RGS1 | 0.56 | | 7.17E-160 | |
| CD8T | CD8T | CHCHD10 | -0.42 | | 7.20E-159 | |
| CD8T | CD8T | TYMP | -0.59 | | 9.78E-159 | |
| CD8T | CD8T | CXCL3 | -1.44 | | 5.01E-158 | |
| CD8T | CD8T | SYNGR2 | -0.33 | | 5.37E-158 | |
| CD8T | CD8T | GNG7 | -0.41 | | 6.18E-158 | |
| CD8T | CD8T | CD151 | -0.37 | | 9.82E-158 | |
| CD8T | CD8T | RNF125 | 0.55 | | 4.70E-157 | |
| CD8T | CD8T | GPX1 | -0.62 | | 1.52E-156 | |
| CD8T | CD8T | SELENOH | -0.32 | | 2.30E-156 | |
| CD8T | CD8T | KLF6 | 0.47 | | 2.08E-155 | |
| CD8T | CD8T | LGALS3 | -0.61 | | 1.45E-152 | |
| CD8T | CD8T | ATP5MC1 | -0.34 | | 7.83E-152 | |
| CD8T | CD8T | TMEM205 | -0.27 | | 2.53E-151 | |
| CD8T | CD8T | LY96 | -0.4 | | 3.79E-151 | |
| CD8T | CD8T | PHPT1 | -0.3 | | 4.56E-151 | |
| CD8T | CD8T | CXCL2 | -1.53 | | 1.14E-150 | |
| CD8T | CD8T | NME1 | -0.3 | | 3.92E-150 | |
| CD8T | CD8T | KRT8 | -1.31 | | 4.45E-150 | |
| CD8T | CD8T | CTSS | -0.47 | | 5.01E-150 | |
| CD8T | CD8T | PLAUR | -1.15 | | 2.31E-149 | |
| CD8T | CD8T | MRPL23 | -0.26 | | 2.99E-147 | |
| CD8T | CD8T | BLVRB | -0.38 | | 6.97E-147 | |
| CD8T | CD8T | CNOT6L | 0.62 | | 8.49E-147 | |
| CD8T | CD8T | TSC22D3 | 0.33 | | 3.44E-146 | |
| CD8T | CD8T | S100A13 | -0.4 | | 4.59E-145 | |
| CD8T | CD8T | PTPRCAP | 0.47 | | 6.90E-145 | |
| CD8T | CD8T | KDELR1 | -0.28 | | 1.03E-144 | |
| CD8T | CD8T | HLA-DPA1 | -0.92 | | 2.08E-144 | |
| CD8T | CD8T | HLA-DMB | -0.53 | | 2.24E-144 | |
| CD8T | CD8T | FYB1 | 0.59 | | 3.21E-144 | |
| CD8T | CD8T | DERL3 | -1.19 | | 1.17E-143 | |
| CD8T | CD8T | KRT18 | -1.18 | | 3.10E-143 | |
| CD8T | CD8T | KLRD1 | 0.54 | | 3.30E-143 | |
| CD8T | CD8T | HSPA8 | 0.52 | | 9.89E-143 | |
| CD8T | CD8T | TSPAN13 | -0.27 | | 4.30E-142 | |
| CD8T | CD8T | MDK | -0.52 | | 3.92E-141 | |
| CD8T | CD8T | HSP90B1 | -0.65 | | 9.22E-141 | |
| CD8T | CD8T | RPL36AL | 0.31 | | 4.80E-140 | |
| CD8T | CD8T | BASP1 | -0.41 | | 1.82E-139 | |
| CD8T | CD8T | HLA-E | 0.32 | | 2.02E-139 | |
| CD8T | CD8T | DUSP2 | 0.7 | | 1.09E-138 | |
| CD8T | CD8T | GAS6 | -0.31 | | 1.49E-138 | |
| CD8T | CD8T | NDUFB7 | -0.32 | | 5.58E-137 | |
| CD8T | CD8T | C15orf48 | -1.43 | | 1.33E-136 | |
| CD8T | CD8T | ICAM1 | -0.54 | | 1.91E-136 | |
| CD8T | CD8T | CD68 | -0.51 | | 6.54E-136 | |
| CD8T | CD8T | EML4 | 0.6 | | 1.38E-135 | |
| CD8T | CD8T | NR3C1 | 0.68 | | 1.87E-135 | |
| CD8T | CD8T | SMIM14 | -0.37 | | 2.92E-135 | |
| CD8T | CD8T | LYN | -0.33 | | 1.48E-133 | |
| CD8T | CD8T | IL2RB | 0.52 | | 2.24E-133 | |
| CD8T | CD8T | HSPB1 | -0.74 | | 2.24E-132 | |
| CD8T | CD8T | PDCD4 | 0.62 | | 4.03E-132 | |
| CD8T | CD8T | MTRNR2L8 | -0.52 | | 5.21E-132 | |
| CD8T | CD8T | TSPAN3 | -0.27 | | 6.08E-132 | |
| CD8T | CD8T | SLA2 | 0.51 | | 2.44E-131 | |
| CD8T | CD8T | GSTP1 | -0.55 | | 2.79E-131 | |
| CD8T | CD8T | ID2 | 0.59 | | 1.21E-130 | |
| CD8T | CD8T | CD9 | -0.56 | | 1.58E-130 | |
| CD8T | CD8T | NDUFB3 | -0.26 | | 2.45E-130 | |
| CD8T | CD8T | CLIC4 | -0.3 | | 7.40E-130 | |
| CD8T | CD8T | TSC22D1 | -0.55 | | 1.03E-129 | |
| CD8T | CD8T | PHLDA2 | -0.48 | | 3.06E-129 | |
| CD8T | CD8T | DDAH2 | -0.28 | | 6.78E-129 | |
| CD8T | CD8T | TCEAL9 | -0.33 | | 1.89E-128 | |
| CD8T | CD8T | HES1 | -0.86 | | 4.40E-128 | |
| CD8T | CD8T | TPD52 | -0.27 | | 1.72E-127 | |
| CD8T | CD8T | ADIRF | -0.98 | | 3.81E-126 | |
| CD8T | CD8T | PLPP5 | -0.27 | | 3.99E-126 | |
| CD8T | CD8T | APP | -0.37 | | 5.16E-126 | |
| CD8T | CD8T | KRT19 | -0.95 | | 1.15E-125 | |
| CD8T | CD8T | NECTIN2 | -0.28 | | 1.55E-125 | |
| CD8T | CD8T | LAG3 | 0.53 | | 6.55E-125 | |
| CD8T | CD8T | RHOB | -0.46 | | 7.63E-125 | |
| CD8T | CD8T | HM13 | -0.25 | | 9.32E-125 | |
| CD8T | CD8T | PLD3 | -0.29 | | 2.05E-124 | |
| CD8T | CD8T | PDIA4 | -0.3 | | 2.20E-124 | |
| CD8T | CD8T | SLC7A5 | 0.64 | | 4.42E-124 | |
| CD8T | CD8T | CD69 | 0.37 | | 7.64E-124 | |
| CD8T | CD8T | SRSF5 | 0.43 | | 1.12E-123 | |
| CD8T | CD8T | HSBP1 | -0.26 | | 2.19E-123 | |
| CD8T | CD8T | BRI3 | -0.27 | | 4.17E-123 | |
| CD8T | CD8T | ETFB | -0.26 | | 1.10E-122 | |
| CD8T | CD8T | CD6 | 0.51 | | 1.22E-122 | |
| CD8T | CD8T | TMEM176B | -0.5 | | 2.72E-122 | |
| CD8T | CD8T | MGST2 | -0.3 | | 8.72E-122 | |
| CD8T | CD8T | IRF8 | -0.38 | | 1.19E-121 | |
| CD8T | CD8T | NDUFB1 | -0.28 | | 2.26E-120 | |
| CD8T | CD8T | BST2 | -0.35 | | 3.46E-120 | |
| CD8T | CD8T | S100A4 | 0.27 | | 4.19E-120 | |
| CD8T | CD8T | POLD4 | -0.25 | | 4.79E-120 | |
| CD8T | CD8T | ATP5F1D | -0.34 | | 1.00E-119 | |
| CD8T | CD8T | CD44 | 0.38 | | 1.26E-119 | |
| CD8T | CD8T | CXCL8 | -2.18 | | 1.30E-119 | |
| CD8T | CD8T | RORA | 0.62 | | 6.23E-119 | |
| CD8T | CD8T | TCF4 | -0.41 | | 7.35E-119 | |
| CD8T | CD8T | CD247 | 0.49 | | 7.40E-119 | |
| CD8T | CD8T | CXCR3 | 0.57 | | 1.06E-118 | |
| CD8T | CD8T | ELF3 | -0.82 | | 1.32E-118 | |
| CD8T | CD8T | GLUL | -0.57 | | 2.97E-118 | |
| CD8T | CD8T | TPM1 | -0.37 | | 1.36E-117 | |
| CD8T | CD8T | SOX4 | -0.41 | | 2.88E-117 | |
| CD8T | CD8T | FAM49A | -0.3 | | 3.37E-117 | |
| CD8T | CD8T | GNG2 | 0.54 | | 9.75E-117 | |
| CD8T | CD8T | C19orf33 | -0.57 | | 1.13E-116 | |
| CD8T | CD8T | SGK1 | -0.43 | | 1.13E-116 | |
| CD8T | CD8T | CDC42SE2 | 0.56 | | 1.98E-116 | |
| CD8T | CD8T | ERGIC3 | -0.28 | | 2.32E-116 | |
| CD8T | CD8T | ANXA4 | -0.4 | | 9.64E-116 | |
| CD8T | CD8T | SMIM22 | -0.54 | | 2.90E-115 | |
| CD8T | CD8T | ETS2 | -0.36 | | 7.40E-115 | |
| CD8T | CD8T | YBX3 | -0.37 | | 1.26E-114 | |
| CD8T | CD8T | SOD2 | -1.37 | | 1.43E-114 | |
| CD8T | CD8T | ADAM28 | -0.26 | | 2.68E-114 | |
| CD8T | CD8T | MZB1 | -1.69 | | 4.51E-114 | |
| CD8T | CD8T | DDX24 | 0.54 | | 6.59E-114 | |
| CD8T | CD8T | SPINK1 | -1.25 | | 7.71E-114 | |
| CD8T | CD8T | TUBA4A | 0.76 | | 7.51E-113 | |
| CD8T | CD8T | HSPA1B | -0.69 | | 7.87E-113 | |
| CD8T | CD8T | LGALS3BP | -0.39 | | 2.12E-112 | |
| CD8T | CD8T | MTRNR2L12 | -0.46 | | 2.23E-112 | |
| CD8T | CD8T | TXN | -0.79 | | 4.45E-112 | |
| CD8T | CD8T | SAT1 | -0.78 | | 5.99E-112 | |
| CD8T | CD8T | PRDX5 | -0.3 | | 1.99E-111 | |
| CD8T | CD8T | TM4SF1 | -1.1 | | 2.37E-111 | |
| CD8T | CD8T | EMP2 | -0.26 | | 1.88E-109 | |
| CD8T | CD8T | FXYD3 | -0.84 | | 3.24E-109 | |
| CD8T | CD8T | MS4A1 | -0.59 | | 8.39E-109 | |
| CD8T | CD8T | STK17B | 0.52 | | 7.35E-108 | |
| CD8T | CD8T | TSPO | -0.38 | | 8.00E-108 | |
| CD8T | CD8T | S100A16 | -0.37 | | 1.24E-107 | |
| CD8T | CD8T | ID3 | -0.56 | | 1.28E-107 | |
| CD8T | CD8T | PPIF | -0.34 | | 1.90E-107 | |
| CD8T | CD8T | ATP5PF | -0.27 | | 5.81E-107 | |
| CD8T | CD8T | TSPAN8 | -1.13 | | 2.09E-106 | |
| CD8T | CD8T | CD55 | -0.34 | | 3.07E-106 | |
| CD8T | CD8T | PTGER4 | 0.6 | | 4.37E-106 | |
| CD8T | CD8T | RPS26 | 0.32 | | 4.37E-105 | |
| CD8T | CD8T | TMEM258 | -0.33 | | 2.12E-104 | |
| CD8T | CD8T | TMEM176A | -0.33 | | 2.70E-104 | |
| CD8T | CD8T | AGR2 | -1.25 | | 2.98E-104 | |
| CD8T | CD8T | PDIA6 | -0.34 | | 8.55E-104 | |
| CD8T | CD8T | ATF3 | -0.43 | | 1.81E-103 | |
| CD8T | CD8T | MT1E | -0.66 | | 1.30E-102 | |
| CD8T | CD8T | BANK1 | -0.42 | | 4.08E-102 | |
| CD8T | CD8T | GSTO1 | -0.31 | | 4.53E-102 | |
| CD8T | CD8T | AP2S1 | -0.26 | | 2.72E-101 | |
| CD8T | CD8T | GNG5 | -0.27 | | 6.56E-101 | |
| CD8T | CD8T | LAT | 0.48 | | 8.99E-101 | |
| CD8T | CD8T | G0S2 | -1.56 | | 1.03E-100 | |
| CD8T | CD8T | AC016831.4 | 0.53 | | 1.10E-100 | |
| CD8T | CD8T | FCER1G | -1.48 | | 1.94E-100 | |
| CD8T | CD8T | NDUFS5 | 0.39 | | 2.22E-100 | |
| CD8T | CD8T | PIGR | -0.85 | | 3.14E-100 | |
| CD8T | CD8T | LGALS4 | -0.66 | | 3.68E-100 | |
| CD8T | CD8T | IL1RN | -0.8 | | 4.78E-100 | |
| CD8T | CD8T | SPI1 | -0.31 | | 9.70E-100 | |
| CD8T | CD8T | HLA-DPB1 | -0.84 | | 1.37E-99 | |
| CD8T | CD8T | MUC1 | -0.63 | | 2.16E-99 | |
| CD8T | CD8T | VSIG2 | -0.39 | | 3.48E-99 | |
| CD8T | CD8T | NAPRT | -0.28 | | 3.90E-99 | |
| CD8T | CD8T | IFI30 | -0.33 | | 3.96E-99 | |
| CD8T | CD8T | LCN2 | -0.86 | | 4.09E-99 | |
| CD8T | CD8T | LGALS1 | -0.92 | | 2.29E-98 | |
| CD8T | CD8T | SKP1 | 0.32 | | 2.87E-98 | |
| CD8T | CD8T | CD40 | -0.26 | | 3.90E-98 | |
| CD8T | CD8T | HIST1H2BK | -0.35 | | 9.76E-98 | |
| CD8T | CD8T | ALOX5AP | 0.47 | | 1.06E-97 | |
| CD8T | CD8T | BEX3 | -0.27 | | 1.33E-97 | |
| CD8T | CD8T | SERPINA1 | -0.54 | | 4.16E-97 | |
| CD8T | CD8T | SYTL3 | 0.49 | | 4.47E-97 | |
| CD8T | CD8T | GADD45A | -0.39 | | 4.61E-97 | |
| CD8T | CD8T | AC020656.1 | -0.49 | | 1.73E-96 | |
| CD8T | CD8T | SOCS3 | -0.67 | | 1.77E-96 | |
| CD8T | CD8T | ANXA2 | -0.48 | | 3.35E-96 | |
| CD8T | CD8T | NQO1 | -0.39 | | 4.68E-96 | |
| CD8T | CD8T | EPCAM | -0.47 | | 4.81E-96 | |
| CD8T | CD8T | EMP1 | -0.57 | | 8.47E-96 | |
| CD8T | CD8T | EGR1 | -0.67 | | 2.16E-95 | |
| CD8T | CD8T | CEBPD | -0.34 | | 3.33E-95 | |
| CD8T | CD8T | S100A14 | -0.49 | | 4.74E-95 | |
| CD8T | CD8T | SELENOP | -0.47 | | 5.53E-95 | |
| CD8T | CD8T | CLEC2B | 0.56 | | 6.76E-95 | |
| CD8T | CD8T | CFD | -0.76 | | 7.27E-95 | |
| CD8T | CD8T | GMDS | -0.25 | | 1.41E-94 | |
| CD8T | CD8T | KLF2 | -0.31 | | 1.47E-94 | |
| CD8T | CD8T | ID1 | -0.51 | | 1.98E-94 | |
| CD8T | CD8T | LY9 | -0.52 | | 2.55E-94 | |
| CD8T | CD8T | CLDN4 | -0.59 | | 3.14E-94 | |
| CD8T | CD8T | GPX2 | -0.41 | | 4.68E-94 | |
| CD8T | CD8T | STARD10 | -0.26 | | 8.77E-94 | |
| CD8T | CD8T | PRDX1 | -0.35 | | 1.85E-93 | |
| CD8T | CD8T | UQCRH | -0.3 | | 2.44E-93 | |
| CD8T | CD8T | FCGR2B | -0.25 | | 4.33E-93 | |
| CD8T | CD8T | BCL11B | 0.41 | | 6.13E-93 | |
| CD8T | CD8T | EVL | 0.51 | | 1.04E-92 | |
| CD8T | CD8T | RNASE6 | -0.28 | | 5.17E-92 | |
| CD8T | CD8T | TNFRSF17 | -0.36 | | 6.19E-92 | |
| CD8T | CD8T | CD19 | -0.3 | | 7.79E-92 | |
| CD8T | CD8T | WARS | -0.25 | | 9.78E-92 | |
| CD8T | CD8T | TMC5 | -0.33 | | 1.04E-91 | |
| CD8T | CD8T | HIST1H1C | -0.46 | | 1.87E-91 | |
| CD8T | CD8T | CD79B | -0.31 | | 3.13E-91 | |
| CD8T | CD8T | SQSTM1 | -0.4 | | 1.18E-90 | |
| CD8T | CD8T | SAMSN1 | 0.47 | | 1.70E-90 | |
| CD8T | CD8T | PDE3B | 0.43 | | 2.17E-90 | |
| CD8T | CD8T | PTPN22 | 0.51 | | 2.60E-90 | |
| CD8T | CD8T | SEC11C | -0.55 | | 4.26E-90 | |
| CD8T | CD8T | IL2RG | 0.45 | | 4.56E-90 | |
| CD8T | CD8T | CLDN18 | -0.43 | | 4.93E-90 | |
| CD8T | CD8T | SERPINB1 | -0.29 | | 5.65E-90 | |
| CD8T | CD8T | CTSL | -0.65 | | 8.00E-90 | |
| CD8T | CD8T | TPM2 | -0.46 | | 8.19E-90 | |
| CD8T | CD8T | TINAGL1 | -0.25 | | 1.10E-89 | |
| CD8T | CD8T | JSRP1 | -0.5 | | 1.77E-89 | |
| CD8T | CD8T | VPREB3 | -0.43 | | 2.08E-89 | |
| CD8T | CD8T | GPRC5A | -0.27 | | 2.22E-89 | |
| CD8T | CD8T | MYADM | 0.53 | | 3.62E-89 | |
| CD8T | CD8T | CAPG | -0.35 | | 4.40E-89 | |
| CD8T | CD8T | KRT7 | -0.35 | | 9.65E-89 | |
| CD8T | CD8T | LY86 | -0.31 | | 3.22E-88 | |
| CD8T | CD8T | S100P | -1 | | 4.07E-88 | |
| CD8T | CD8T | NEAT1 | -0.57 | | 5.09E-88 | |
| CD8T | CD8T | WIPF1 | 0.49 | | 8.22E-88 | |
| CD8T | CD8T | COX6B1 | -0.29 | | 1.98E-87 | |
| CD8T | CD8T | ANXA10 | -0.52 | | 2.05E-87 | |
| CD8T | CD8T | CYSTM1 | -0.82 | | 2.32E-87 | |
| CD8T | CD8T | TACSTD2 | -0.34 | | 4.53E-87 | |
| CD8T | CD8T | SLA | 0.49 | | 5.69E-87 | |
| CD8T | CD8T | UQCRQ | -0.32 | | 8.92E-87 | |
| CD8T | CD8T | RNASE1 | -0.55 | | 1.14E-86 | |
| CD8T | CD8T | SSR3 | -0.35 | | 1.53E-86 | |
| CD8T | CD8T | ANXA5 | -0.36 | | 4.85E-86 | |
| CD8T | CD8T | GABARAPL1 | 0.51 | | 4.90E-86 | |
| CD8T | CD8T | IL1B | -1.68 | | 5.71E-86 | |
| CD8T | CD8T | SH2D1A | 0.44 | | 5.86E-86 | |
| CD8T | CD8T | CORO1A | 0.32 | | 6.20E-86 | |
| CD8T | CD8T | CTSD | -0.55 | | 6.69E-86 | |
| CD8T | CD8T | KYNU | -0.29 | | 9.56E-86 | |
| CD8T | CD8T | ATP1B1 | -0.25 | | 1.69E-85 | |
| CD8T | CD8T | HSPG2 | -0.42 | | 2.67E-85 | |
| CD8T | CD8T | SDF2L1 | -0.29 | | 5.67E-85 | |
| CD8T | CD8T | ACAP1 | 0.5 | | 2.97E-84 | |
| CD8T | CD8T | MYDGF | -0.27 | | 4.49E-84 | |
| CD8T | CD8T | ANPEP | -0.33 | | 1.42E-83 | |
| CD8T | CD8T | ATP5ME | -0.29 | | 4.83E-83 | |
| CD8T | CD8T | LAPTM4A | -0.27 | | 4.03E-82 | |
| CD8T | CD8T | RARRES3 | 0.52 | | 1.17E-81 | |
| CD8T | CD8T | RUNX3 | 0.51 | | 1.58E-81 | |
| CD8T | CD8T | PSCA | -0.96 | | 2.17E-81 | |
| CD8T | CD8T | ZAP70 | 0.38 | | 4.74E-80 | |
| CD8T | CD8T | EIF4A1 | 0.28 | | 1.09E-79 | |
| CD8T | CD8T | RNF130 | -0.25 | | 1.72E-79 | |
| CD8T | CD8T | MTRNR2L1 | -0.29 | | 1.75E-79 | |
| CD8T | CD8T | ICOS | 0.5 | | 5.03E-79 | |
| CD8T | CD8T | SFN | -0.3 | | 5.16E-79 | |
| CD8T | CD8T | PPA1 | -0.26 | | 3.94E-78 | |
| CD8T | CD8T | LYAR | 0.51 | | 4.64E-78 | |
| CD8T | CD8T | MT1G | -0.9 | | 5.43E-78 | |
| CD8T | CD8T | AIF1 | -0.71 | | 1.45E-77 | |
| CD8T | CD8T | ARL14 | -0.34 | | 3.66E-77 | |
| CD8T | CD8T | TUBB | -0.29 | | 4.51E-77 | |
| CD8T | CD8T | TFF2 | -1.19 | | 2.82E-76 | |
| CD8T | CD8T | CA2 | -0.65 | | 3.56E-76 | |
| CD8T | CD8T | PHACTR1 | -0.34 | | 4.37E-76 | |
| CD8T | CD8T | CTSE | -0.36 | | 5.45E-76 | |
| CD8T | CD8T | IGFBP7 | -1.84 | | 5.69E-76 | |
| CD8T | CD8T | ARL4C | 0.47 | | 1.57E-75 | |
| CD8T | CD8T | DUSP5 | -0.35 | | 9.98E-75 | |
| CD8T | CD8T | RAB30 | -0.26 | | 3.32E-74 | |
| CD8T | CD8T | LST1 | -0.42 | | 7.24E-74 | |
| CD8T | CD8T | PRKCH | 0.41 | | 1.15E-73 | |
| CD8T | CD8T | S100A9 | -1.41 | | 1.85E-73 | |
| CD8T | CD8T | HSPA1A | -0.6 | | 4.76E-73 | |
| CD8T | CD8T | PGK1 | 0.45 | | 2.56E-72 | |
| CD8T | CD8T | CXCL1 | -0.77 | | 3.45E-72 | |
| CD8T | CD8T | AKNA | 0.53 | | 2.03E-71 | |
| CD8T | CD8T | RAC1 | -0.32 | | 2.06E-71 | |
| CD8T | CD8T | NPDC1 | -0.3 | | 3.95E-71 | |
| CD8T | CD8T | HNRNPA0 | 0.38 | | 4.64E-71 | |
| CD8T | CD8T | JAML | 0.43 | | 4.99E-71 | |
| CD8T | CD8T | MGAT4A | 0.53 | | 2.00E-70 | |
| CD8T | CD8T | APOBEC3G | 0.55 | | 3.74E-70 | |
| CD8T | CD8T | DSTN | -0.28 | | 7.26E-70 | |
| CD8T | CD8T | ACP5 | -0.28 | | 1.37E-69 | |
| CD8T | CD8T | ELOB | -0.29 | | 2.27E-69 | |
| CD8T | CD8T | CLU | -0.58 | | 1.78E-68 | |
| CD8T | CD8T | TXNIP | -0.26 | | 5.57E-68 | |
| CD8T | CD8T | HERPUD1 | -0.84 | | 8.34E-68 | |
| CD8T | CD8T | AUTS2 | 0.41 | | 1.75E-67 | |
| CD8T | CD8T | NFKBIA | -0.5 | | 2.10E-66 | |
| CD8T | CD8T | IDS | 0.43 | | 2.51E-66 | |
| CD8T | CD8T | PKM | -0.27 | | 4.72E-65 | |
| CD8T | CD8T | MTRNR2L2 | -0.57 | | 7.27E-65 | |
| CD8T | CD8T | APOE | -1.31 | | 1.48E-64 | |
| CD8T | CD8T | PDCD1 | 0.36 | | 1.63E-63 | |
| CD8T | CD8T | CD99 | 0.37 | | 3.33E-63 | |
| CD8T | CD8T | TYROBP | -0.99 | | 3.66E-63 | |
| CD8T | CD8T | CSTB | -0.47 | | 5.20E-63 | |
| CD8T | CD8T | PDCL3 | 0.53 | | 6.49E-63 | |
| CD8T | CD8T | BCL2A1 | -0.66 | | 1.17E-62 | |
| CD8T | CD8T | CRIP2 | -0.4 | | 6.44E-62 | |
| CD8T | CD8T | PDE4D | 0.47 | | 6.49E-62 | |
| CD8T | CD8T | IFI6 | -0.31 | | 6.80E-62 | |
| CD8T | CD8T | ABL2 | -0.25 | | 7.32E-62 | |
| CD8T | CD8T | COX5B | -0.26 | | 1.05E-61 | |
| CD8T | CD8T | GATA3 | 0.37 | | 5.33E-61 | |
| CD8T | CD8T | ARHGAP9 | 0.42 | | 4.45E-59 | |
| CD8T | CD8T | CAMK4 | 0.33 | | 1.92E-58 | |
| CD8T | CD8T | PIM2 | -0.3 | | 2.61E-58 | |
| CD8T | CD8T | CLEC2D | 0.41 | | 2.83E-58 | |
| CD8T | CD8T | S100A11 | -0.45 | | 4.83E-58 | |
| CD8T | CD8T | HOPX | 0.39 | | 1.44E-57 | |
| CD8T | CD8T | TFF1 | -1.89 | | 1.52E-57 | |
| CD8T | CD8T | MT-ND4L | -0.4 | | 1.57E-57 | |
| CD8T | CD8T | SEC61G | -0.26 | | 3.12E-57 | |
| CD8T | CD8T | STK17A | 0.46 | | 1.20E-56 | |
| CD8T | CD8T | ETS1 | 0.42 | | 1.66E-56 | |
| CD8T | CD8T | MT2A | -0.77 | | 2.25E-56 | |
| CD8T | CD8T | IGFBP4 | -0.52 | | 4.15E-56 | |
| CD8T | CD8T | TSPYL2 | 0.52 | | 4.58E-55 | |
| CD8T | CD8T | CD5 | 0.35 | | 1.23E-53 | |
| CD8T | CD8T | AC058791.1 | 0.48 | | 1.85E-53 | |
| CD8T | CD8T | PGC | -2.16 | | 3.36E-52 | |
| CD8T | CD8T | FNBP1 | 0.4 | | 7.11E-52 | |
| CD8T | CD8T | PBXIP1 | 0.44 | | 6.42E-51 | |
| CD8T | CD8T | NR4A2 | 0.42 | | 9.18E-51 | |
| CD8T | CD8T | SLC38A1 | 0.46 | | 9.63E-51 | |
| CD8T | CD8T | MCL1 | 0.32 | | 2.23E-50 | |
| CD8T | CD8T | DNAJB6 | 0.4 | | 2.48E-50 | |
| CD8T | CD8T | XBP1 | -0.63 | | 3.20E-50 | |
| CD8T | CD8T | G3BP2 | 0.53 | | 5.42E-50 | |
| CD8T | CD8T | CXCR6 | 0.37 | | 9.63E-50 | |
| CD8T | CD8T | CRIP1 | 0.35 | | 7.31E-49 | |
| CD8T | CD8T | SPN | 0.35 | | 9.74E-49 | |
| CD8T | CD8T | AC114760.2 | 0.42 | | 8.43E-48 | |
| CD8T | CD8T | ITK | 0.31 | | 5.81E-46 | |
| CD8T | CD8T | PNRC1 | 0.28 | | 6.19E-46 | |
| CD8T | CD8T | EMP3 | -0.31 | | 8.18E-46 | |
| CD8T | CD8T | DOK2 | 0.37 | | 1.14E-45 | |
| CD8T | CD8T | HMGB2 | 0.35 | | 1.33E-44 | |
| CD8T | CD8T | PARP8 | 0.38 | | 1.64E-44 | |
| CD8T | CD8T | PRKX | 0.33 | | 5.05E-44 | |
| CD8T | CD8T | ADGRE5 | 0.42 | | 6.60E-44 | |
| CD8T | CD8T | DUSP4 | 0.49 | | 2.52E-43 | |
| CD8T | CD8T | ZNF331 | 0.39 | | 6.12E-43 | |
| CD8T | CD8T | IGLL5 | -3.18 | | 1.08E-42 | |
| CD8T | CD8T | GPR183 | 0.33 | | 1.40E-42 | |
| CD8T | CD8T | RNF19A | 0.46 | | 2.60E-42 | |
| CD8T | CD8T | MT-CO1 | -0.44 | | 6.43E-42 | |
| CD8T | CD8T | ITGA1 | 0.27 | | 6.64E-42 | |
| CD8T | CD8T | PTPN7 | 0.41 | | 7.37E-42 | |
| CD8T | CD8T | FAM129A | 0.39 | | 1.58E-41 | |
| CD8T | CD8T | CD53 | 0.31 | | 7.18E-41 | |
| CD8T | CD8T | GIMAP7 | 0.34 | | 1.20E-40 | |
| CD8T | CD8T | RBM8A | 0.32 | | 2.48E-40 | |
| CD8T | CD8T | IER2 | -0.33 | | 3.38E-40 | |
| CD8T | CD8T | TNFRSF1B | 0.4 | | 6.85E-40 | |
| CD8T | CD8T | SOD1 | 0.29 | | 1.22E-39 | |
| CD8T | CD8T | IFITM1 | 0.3 | | 3.50E-39 | |
| CD8T | CD8T | PPP1CB | 0.38 | | 4.72E-39 | |
| CD8T | CD8T | GADD45B | -0.4 | | 1.22E-38 | |
| CD8T | CD8T | EVI2A | 0.45 | | 3.74E-38 | |
| CD8T | CD8T | MIAT | 0.34 | | 9.07E-37 | |
| CD8T | CD8T | SPCS1 | -0.26 | | 1.14E-36 | |
| CD8T | CD8T | MT-CYB | -0.39 | | 1.90E-36 | |
| CD8T | CD8T | GPR65 | 0.34 | | 1.97E-36 | |
| CD8T | CD8T | TNF | 0.46 | | 2.69E-36 | |
| CD8T | CD8T | IFITM2 | 0.31 | | 1.35E-35 | |
| CD8T | CD8T | FKBP11 | -0.61 | | 2.58E-35 | |
| CD8T | CD8T | BICDL1 | 0.28 | | 4.10E-35 | |
| CD8T | CD8T | TTC39C | 0.37 | | 7.12E-35 | |
| CD8T | CD8T | CD28 | 0.27 | | 1.52E-34 | |
| CD8T | CD8T | CNBP | 0.29 | | 2.16E-34 | |
| CD8T | CD8T | FOSB | -0.33 | | 2.68E-34 | |
| CD8T | CD8T | S100A6 | -0.96 | | 6.44E-34 | |
| CD8T | CD8T | FAM118A | 0.37 | | 1.06E-33 | |
| CD8T | CD8T | VAMP2 | 0.36 | | 1.10E-33 | |
| CD8T | CD8T | LITAF | 0.36 | | 4.99E-33 | |
| CD8T | CD8T | SSR4 | -1.2 | | 5.61E-33 | |
| CD8T | CD8T | CBLB | 0.37 | | 2.22E-32 | |
| CD8T | CD8T | CCND2 | 0.36 | | 3.23E-32 | |
| CD8T | CD8T | PPP1R16B | 0.29 | | 3.97E-32 | |
| CD8T | CD8T | RASAL3 | 0.29 | | 9.88E-32 | |
| CD8T | CD8T | FUS | 0.31 | | 3.61E-31 | |
| CD8T | CD8T | EMB | 0.37 | | 5.14E-31 | |
| CD8T | CD8T | SKAP1 | 0.31 | | 8.33E-30 | |
| CD8T | CD8T | ANKRD12 | 0.42 | | 9.98E-30 | |
| CD8T | CD8T | CLDND1 | 0.49 | | 2.99E-28 | |
| CD8T | CD8T | LCP1 | 0.32 | | 4.62E-28 | |
| CD8T | CD8T | JCHAIN | -3.33 | | 1.50E-27 | |
| CD8T | CD8T | PITPNC1 | 0.27 | | 2.02E-27 | |
| CD8T | CD8T | CYBA | -0.29 | | 2.54E-27 | |
| CD8T | CD8T | TCF7 | 0.31 | | 3.13E-27 | |
| CD8T | CD8T | PIK3IP1 | 0.33 | | 3.73E-27 | |
| CD8T | CD8T | ODF2L | 0.37 | | 4.58E-27 | |
| CD8T | CD8T | OTULINL | 0.27 | | 5.78E-27 | |
| CD8T | CD8T | JMJD6 | 0.43 | | 1.99E-26 | |
| CD8T | CD8T | APMAP | 0.41 | | 2.29E-26 | |
| CD8T | CD8T | S1PR4 | 0.33 | | 2.68E-26 | |
| CD8T | CD8T | TGFB1 | 0.38 | | 6.23E-26 | |
| CD8T | CD8T | GIMAP5 | 0.27 | | 7.90E-26 | |
| CD8T | CD8T | SON | 0.3 | | 1.34E-25 | |
| CD8T | CD8T | C9orf78 | 0.39 | | 2.72E-25 | |
| CD8T | CD8T | LINC00513 | 0.33 | | 7.69E-25 | |
| CD8T | CD8T | GLIPR1 | 0.4 | | 1.55E-24 | |
| CD8T | CD8T | ARID4B | 0.38 | | 3.26E-24 | |
| CD8T | CD8T | ARHGEF1 | 0.37 | | 4.09E-24 | |
| CD8T | CD8T | LBH | 0.29 | | 8.28E-24 | |
| CD8T | CD8T | RPS4Y1 | 0.28 | | 6.72E-23 | |
| CD8T | CD8T | ATP8A1 | 0.29 | | 1.11E-22 | |
| CD8T | CD8T | STK4 | 0.34 | | 1.14E-22 | |
| CD8T | CD8T | ABI3 | 0.3 | | 2.78E-22 | |
| CD8T | CD8T | CCSER2 | 0.35 | | 3.67E-22 | |
| CD8T | CD8T | LDLRAD4 | 0.39 | | 4.61E-22 | |
| CD8T | CD8T | PPP1R2 | 0.4 | | 7.31E-22 | |
| CD8T | CD8T | TBC1D10C | 0.31 | | 1.76E-21 | |
| CD8T | CD8T | ITGA4 | 0.31 | | 3.73E-21 | |
| CD8T | CD8T | TMSB10 | -0.25 | | 4.71E-21 | |
| CD8T | CD8T | SELPLG | 0.31 | | 5.86E-20 | |
| CD8T | CD8T | AKAP13 | 0.38 | | 6.11E-20 | |
| CD8T | CD8T | RNF166 | 0.31 | | 8.46E-20 | |
| CD8T | CD8T | ARID5A | 0.31 | | 8.78E-20 | |
| CD8T | CD8T | NCL | 0.3 | | 1.09E-19 | |
| CD8T | CD8T | RHOH | 0.27 | | 1.38E-19 | |
| CD8T | CD8T | JUNB | -0.26 | | 1.57E-19 | |
| CD8T | CD8T | GNLY | 0.76 | | 2.19E-19 | |
| CD8T | CD8T | 1-Sep | 0.33 | | 2.52E-19 | |
| CD8T | CD8T | PTPN4 | 0.26 | | 3.80E-19 | |
| CD8T | CD8T | ELF1 | 0.35 | | 5.16E-19 | |
| CD8T | CD8T | TMEM50A | 0.31 | | 1.26E-18 | |
| CD8T | CD8T | GMFG | 0.26 | | 2.37E-18 | |
| CD8T | CD8T | NSMCE3 | 0.3 | | 3.22E-18 | |
| CD8T | CD8T | TNFRSF25 | 0.29 | | 1.27E-17 | |
| CD8T | CD8T | RCAN3 | 0.3 | | 1.57E-17 | |
| CD8T | CD8T | UBE2B | 0.31 | | 1.90E-17 | |
| CD8T | CD8T | RALGAPA1 | 0.3 | | 2.14E-17 | |
| CD8T | CD8T | ARAP2 | 0.27 | | 5.10E-17 | |
| CD8T | CD8T | CCL20 | -0.58 | | 6.15E-17 | |
| CD8T | CD8T | KMT2E | 0.29 | | 7.41E-17 | |
| CD8T | CD8T | ITGB2 | 0.27 | | 4.67E-16 | |
| CD8T | CD8T | SPRY1 | 0.26 | | 1.40E-15 | |
| CD8T | CD8T | ZC3HAV1 | 0.35 | | 1.88E-15 | |
| CD8T | CD8T | CRYBG1 | 0.29 | | 4.39E-15 | |
| CD8T | CD8T | HERPUD2 | 0.33 | | 7.51E-14 | |
| CD8T | CD8T | MT-ND2 | -0.35 | | 1.36E-13 | |
| CD8T | CD8T | P2RY10 | 0.27 | | 2.11E-13 | |
| CD8T | CD8T | PRPF38B | 0.34 | | 2.31E-13 | |
| CD8T | CD8T | AAK1 | 0.28 | | 2.97E-13 | |
| CD8T | CD8T | SF1 | 0.26 | | 3.92E-13 | |
| CD8T | CD8T | CLIC3 | 0.28 | | 7.40E-13 | |
| CD8T | CD8T | SS18L2 | 0.33 | | 1.01E-12 | |
| CD8T | CD8T | CHST12 | 0.37 | | 1.24E-12 | |
| CD8T | CD8T | TAGAP | 0.32 | | 1.69E-12 | |
| CD8T | CD8T | OASL | 0.3 | | 2.31E-12 | |
| CD8T | CD8T | 7-Sep | 0.28 | | 5.15E-12 | |
| CD8T | CD8T | HNRNPUL1 | 0.33 | | 5.93E-12 | |
| CD8T | CD8T | HOXB2 | 0.25 | | 1.03E-11 | |
| CD8T | CD8T | PIP4K2A | 0.29 | | 1.22E-11 | |
| CD8T | CD8T | LYST | 0.35 | | 3.18E-11 | |
| CD8T | CD8T | NAA50 | 0.39 | | 3.51E-11 | |
| CD8T | CD8T | CMC1 | 0.62 | | 4.90E-11 | |
| CD8T | CD8T | AC020916.1 | 0.38 | | 5.25E-11 | |
| CD8T | CD8T | TC2N | 0.28 | | 1.15E-10 | |
| CD8T | CD8T | TERF2IP | 0.33 | | 2.07E-10 | |
| CD8T | CD8T | CELF2 | 0.27 | | 5.72E-10 | |
| CD8T | CD8T | NEU1 | 0.36 | | 1.56E-09 | |
| CD8T | CD8T | ELOVL5 | 0.27 | | 3.14E-09 | |
| CD8T | CD8T | SYNE2 | 0.3 | | 4.01E-09 | |
| CD8T | CD8T | PAXX | 0.32 | | 6.54E-09 | |
| CD8T | CD8T | HLA-F | 0.27 | | 1.44E-08 | |
| CD8T | CD8T | MT-CO3 | -0.57 | | 2.04E-08 | |
| CD8T | CD8T | RSRP1 | 0.31 | | 2.18E-08 | |
| CD8T | CD8T | N4BP2L2 | 0.27 | | 2.19E-08 | |
| CD8T | CD8T | SUN2 | 0.29 | | 3.42E-08 | |
| CD8T | CD8T | TSEN54 | 0.27 | | 3.91E-08 | |
| CD8T | CD8T | ARF6 | 0.29 | | 3.95E-08 | |
| CD8T | CD8T | BAZ1A | 0.3 | | 4.45E-08 | |
| CD8T | CD8T | RASSF5 | 0.25 | | 5.57E-08 | |
| CD8T | CD8T | SRRM1 | 0.29 | | 5.70E-08 | |
| CD8T | CD8T | SYAP1 | 0.31 | | 7.82E-08 | |
| CD8T | CD8T | HNRNPLL | 0.25 | | 9.43E-08 | |
| CD8T | CD8T | PLK3 | 0.31 | | 9.58E-08 | |
| CD8T | CD8T | YIPF5 | 0.33 | | 1.16E-07 | |
| CD8T | CD8T | CCNH | 0.32 | | 1.68E-07 | |
| CD8T | CD8T | GSPT1 | 0.34 | | 3.71E-07 | |
| CD8T | CD8T | DHX36 | 0.32 | | 4.86E-07 | |
| CD8T | CD8T | TAF7 | 0.27 | | 8.75E-07 | |
| CD8T | CD8T | SRRT | 0.32 | | 9.38E-07 | |
| CD8T | CD8T | AMD1 | 0.32 | | 1.27E-06 | |
| CD8T | CD8T | ISCA1 | 0.3 | | 1.53E-06 | |
| CD8T | CD8T | MT-ND1 | -0.55 | | 1.82E-06 | |
| CD8T | CD8T | ARL6IP5 | 0.27 | | 3.09E-06 | |
| CD8T | CD8T | TBCC | 0.3 | | 4.85E-06 | |
| CD8T | CD8T | ZBTB1 | 0.28 | | 6.59E-06 | |
| CD8T | CD8T | BUB3 | 0.32 | | 9.49E-06 | |
| Mono/Macro | Mono/Macro | IL1B | 3.92 | | 0 | |
| Mono/Macro | Mono/Macro | CXCL8 | 3.71 | | 0 | |
| Mono/Macro | Mono/Macro | S100A9 | 3.62 | | 0 | |
| Mono/Macro | Mono/Macro | G0S2 | 3.58 | | 0 | |
| Mono/Macro | Mono/Macro | TIMP1 | 3.53 | | 0 | |
| Mono/Macro | Mono/Macro | S100A8 | 3.33 | | 0 | |
| Mono/Macro | Mono/Macro | C15orf48 | 3.28 | | 0 | |
| Mono/Macro | Mono/Macro | SOD2 | 3.23 | | 0 | |
| Mono/Macro | Mono/Macro | SPP1 | 3.18 | | 0 | |
| Mono/Macro | Mono/Macro | CCL20 | 3.06 | | 0 | |
| Mono/Macro | Mono/Macro | CXCL3 | 3.06 | | 0 | |
| Mono/Macro | Mono/Macro | FCER1G | 3.05 | | 0 | |
| Mono/Macro | Mono/Macro | CXCL2 | 3.03 | | 0 | |
| Mono/Macro | Mono/Macro | IL1RN | 3.03 | | 0 | |
| Mono/Macro | Mono/Macro | PLAUR | 2.93 | | 0 | |
| Mono/Macro | Mono/Macro | EREG | 2.7 | | 0 | |
| Mono/Macro | Mono/Macro | BCL2A1 | 2.44 | | 0 | |
| Mono/Macro | Mono/Macro | CXCL1 | 2.37 | | 0 | |
| Mono/Macro | Mono/Macro | TYROBP | 2.33 | | 0 | |
| Mono/Macro | Mono/Macro | FTH1 | 2.23 | | 0 | |
| Mono/Macro | Mono/Macro | CTSL | 2.15 | | 0 | |
| Mono/Macro | Mono/Macro | SERPINA1 | 2.1 | | 0 | |
| Mono/Macro | Mono/Macro | SAT1 | 2.01 | | 0 | |
| Mono/Macro | Mono/Macro | TREM1 | 1.88 | | 0 | |
| Mono/Macro | Mono/Macro | CD14 | 1.85 | | 0 | |
| Mono/Macro | Mono/Macro | PPIF | 1.83 | | 0 | |
| Mono/Macro | Mono/Macro | PTGS2 | 1.83 | | 0 | |
| Mono/Macro | Mono/Macro | AIF1 | 1.77 | | 0 | |
| Mono/Macro | Mono/Macro | CTSB | 1.72 | | 0 | |
| Mono/Macro | Mono/Macro | LGALS1 | 1.71 | | 0 | |
| Mono/Macro | Mono/Macro | ANXA5 | 1.7 | | 0 | |
| Mono/Macro | Mono/Macro | LYZ | 1.69 | | 0 | |
| Mono/Macro | Mono/Macro | IER3 | 1.66 | | 0 | |
| Mono/Macro | Mono/Macro | NINJ1 | 1.62 | | 0 | |
| Mono/Macro | Mono/Macro | CLEC4E | 1.6 | | 0 | |
| Mono/Macro | Mono/Macro | S100A12 | 1.6 | | 0 | |
| Mono/Macro | Mono/Macro | FTL | 1.6 | | 0 | |
| Mono/Macro | Mono/Macro | GSTO1 | 1.57 | | 0 | |
| Mono/Macro | Mono/Macro | INHBA | 1.57 | | 0 | |
| Mono/Macro | Mono/Macro | AQP9 | 1.55 | | 0 | |
| Mono/Macro | Mono/Macro | FCN1 | 1.55 | | 0 | |
| Mono/Macro | Mono/Macro | NAMPT | 1.52 | | 0 | |
| Mono/Macro | Mono/Macro | TNFAIP6 | 1.51 | | 0 | |
| Mono/Macro | Mono/Macro | SDC2 | 1.5 | | 0 | |
| Mono/Macro | Mono/Macro | THBS1 | 1.48 | | 0 | |
| Mono/Macro | Mono/Macro | S100A10 | 1.46 | | 0 | |
| Mono/Macro | Mono/Macro | S100A11 | 1.45 | | 0 | |
| Mono/Macro | Mono/Macro | FCGR2A | 1.45 | | 0 | |
| Mono/Macro | Mono/Macro | TYMP | 1.44 | | 0 | |
| Mono/Macro | Mono/Macro | TGFBI | 1.44 | | 0 | |
| Mono/Macro | Mono/Macro | KYNU | 1.41 | | 0 | |
| Mono/Macro | Mono/Macro | LST1 | 1.4 | | 0 | |
| Mono/Macro | Mono/Macro | CSTA | 1.39 | | 0 | |
| Mono/Macro | Mono/Macro | VCAN | 1.39 | | 0 | |
| Mono/Macro | Mono/Macro | GK | 1.38 | | 0 | |
| Mono/Macro | Mono/Macro | MMP19 | 1.38 | | 0 | |
| Mono/Macro | Mono/Macro | OLR1 | 1.37 | | 0 | |
| Mono/Macro | Mono/Macro | MCEMP1 | 1.36 | | 0 | |
| Mono/Macro | Mono/Macro | CD63 | 1.34 | | 0 | |
| Mono/Macro | Mono/Macro | CEBPB | 1.34 | | 0 | |
| Mono/Macro | Mono/Macro | MMP9 | 1.33 | | 0 | |
| Mono/Macro | Mono/Macro | C5AR1 | 1.32 | | 0 | |
| Mono/Macro | Mono/Macro | SDS | 1.32 | | 0 | |
| Mono/Macro | Mono/Macro | SLC25A37 | 1.32 | | 0 | |
| Mono/Macro | Mono/Macro | ATP13A3 | 1.28 | | 0 | |
| Mono/Macro | Mono/Macro | CD68 | 1.28 | | 0 | |
| Mono/Macro | Mono/Macro | SLC11A1 | 1.27 | | 0 | |
| Mono/Macro | Mono/Macro | BASP1 | 1.24 | | 0 | |
| Mono/Macro | Mono/Macro | PHLDA1 | 1.23 | | 0 | |
| Mono/Macro | Mono/Macro | SMIM25 | 1.23 | | 0 | |
| Mono/Macro | Mono/Macro | GLUL | 1.23 | | 0 | |
| Mono/Macro | Mono/Macro | NPC2 | 1.22 | | 0 | |
| Mono/Macro | Mono/Macro | CD300E | 1.19 | | 0 | |
| Mono/Macro | Mono/Macro | IL1A | 1.16 | | 0 | |
| Mono/Macro | Mono/Macro | PLEK | 1.15 | | 0 | |
| Mono/Macro | Mono/Macro | CLEC5A | 1.15 | | 0 | |
| Mono/Macro | Mono/Macro | HIF1A | 1.15 | | 0 | |
| Mono/Macro | Mono/Macro | ASAH1 | 1.15 | | 0 | |
| Mono/Macro | Mono/Macro | CLEC7A | 1.12 | | 0 | |
| Mono/Macro | Mono/Macro | SERPINB2 | 1.11 | | 0 | |
| Mono/Macro | Mono/Macro | RAB31 | 1.09 | | 0 | |
| Mono/Macro | Mono/Macro | CCRL2 | 1.08 | | 0 | |
| Mono/Macro | Mono/Macro | VEGFA | 1.08 | | 0 | |
| Mono/Macro | Mono/Macro | IFI30 | 1.07 | | 0 | |
| Mono/Macro | Mono/Macro | UPP1 | 1.06 | | 0 | |
| Mono/Macro | Mono/Macro | FPR1 | 1.06 | | 0 | |
| Mono/Macro | Mono/Macro | PMP22 | 1.04 | | 0 | |
| Mono/Macro | Mono/Macro | SLC16A10 | 1.01 | | 0 | |
| Mono/Macro | Mono/Macro | MXD1 | 1 | | 0 | |
| Mono/Macro | Mono/Macro | NCF2 | 0.96 | | 0 | |
| Mono/Macro | Mono/Macro | RETN | 0.94 | | 0 | |
| Mono/Macro | Mono/Macro | SLC43A2 | 0.94 | | 0 | |
| Mono/Macro | Mono/Macro | CD86 | 0.92 | | 0 | |
| Mono/Macro | Mono/Macro | ANPEP | 0.91 | | 0 | |
| Mono/Macro | Mono/Macro | CD93 | 0.91 | | 0 | |
| Mono/Macro | Mono/Macro | SPHK1 | 0.9 | | 0 | |
| Mono/Macro | Mono/Macro | PILRA | 0.89 | | 0 | |
| Mono/Macro | Mono/Macro | SPI1 | 0.89 | | 0 | |
| Mono/Macro | Mono/Macro | AC245128.3 | 0.89 | | 0 | |
| Mono/Macro | Mono/Macro | APOBEC3A | 0.87 | | 0 | |
| Mono/Macro | Mono/Macro | DSE | 0.86 | | 0 | |
| Mono/Macro | Mono/Macro | ICAM1 | 0.84 | | 0 | |
| Mono/Macro | Mono/Macro | MS4A7 | 0.82 | | 0 | |
| Mono/Macro | Mono/Macro | ITGAX | 0.82 | | 0 | |
| Mono/Macro | Mono/Macro | LILRB4 | 0.8 | | 0 | |
| Mono/Macro | Mono/Macro | LCP2 | 0.8 | | 0 | |
| Mono/Macro | Mono/Macro | CPVL | 0.79 | | 0 | |
| Mono/Macro | Mono/Macro | NLRP3 | 0.79 | | 0 | |
| Mono/Macro | Mono/Macro | GPR84 | 0.79 | | 0 | |
| Mono/Macro | Mono/Macro | HPSE | 0.78 | | 0 | |
| Mono/Macro | Mono/Macro | IL3RA | 0.78 | | 0 | |
| Mono/Macro | Mono/Macro | MAFB | 0.78 | | 0 | |
| Mono/Macro | Mono/Macro | SLAMF9 | 0.77 | | 0 | |
| Mono/Macro | Mono/Macro | BCAT1 | 0.77 | | 0 | |
| Mono/Macro | Mono/Macro | MIR3945HG | 0.76 | | 0 | |
| Mono/Macro | Mono/Macro | CFP | 0.73 | | 0 | |
| Mono/Macro | Mono/Macro | IGSF6 | 0.73 | | 0 | |
| Mono/Macro | Mono/Macro | SLC7A11 | 0.72 | | 0 | |
| Mono/Macro | Mono/Macro | HCK | 0.72 | | 0 | |
| Mono/Macro | Mono/Macro | PID1 | 0.69 | | 0 | |
| Mono/Macro | Mono/Macro | FGR | 0.68 | | 0 | |
| Mono/Macro | Mono/Macro | MARCO | 0.67 | | 0 | |
| Mono/Macro | Mono/Macro | EPB41L3 | 0.67 | | 0 | |
| Mono/Macro | Mono/Macro | TLR2 | 0.66 | | 0 | |
| Mono/Macro | Mono/Macro | PLA2G7 | 0.64 | | 0 | |
| Mono/Macro | Mono/Macro | FPR2 | 0.63 | | 0 | |
| Mono/Macro | Mono/Macro | CLEC4A | 0.63 | | 0 | |
| Mono/Macro | Mono/Macro | DENND5A | 0.63 | | 0 | |
| Mono/Macro | Mono/Macro | LILRA5 | 0.6 | | 0 | |
| Mono/Macro | Mono/Macro | ADGRE2 | 0.53 | | 0 | |
| Mono/Macro | Mono/Macro | SEMA6B | 0.53 | | 0 | |
| Mono/Macro | Mono/Macro | LILRB2 | 0.48 | | 0 | |
| Mono/Macro | Mono/Macro | LINC02376 | 0.43 | | 0 | |
| Mono/Macro | Mono/Macro | NRG1 | 0.39 | | 0 | |
| Mono/Macro | Mono/Macro | OSCAR | 0.39 | | 0 | |
| Mono/Macro | Mono/Macro | CLEC4D | 0.37 | | 0 | |
| Mono/Macro | Mono/Macro | SRGN | 1.11 | | 1.32E-305 | |
| Mono/Macro | Mono/Macro | RPL3 | -1.06 | | 6.85E-304 | |
| Mono/Macro | Mono/Macro | PDE4DIP | 0.79 | | 2.33E-302 | |
| Mono/Macro | Mono/Macro | ACSL1 | 0.74 | | 1.11E-297 | |
| Mono/Macro | Mono/Macro | PLSCR1 | 0.92 | | 6.30E-295 | |
| Mono/Macro | Mono/Macro | OSM | 0.59 | | 3.83E-290 | |
| Mono/Macro | Mono/Macro | DMXL2 | 0.51 | | 1.34E-289 | |
| Mono/Macro | Mono/Macro | CST3 | 1 | | 8.06E-289 | |
| Mono/Macro | Mono/Macro | SERPINB9 | 1.08 | | 3.39E-288 | |
| Mono/Macro | Mono/Macro | SYN1 | 0.27 | | 7.68E-287 | |
| Mono/Macro | Mono/Macro | ZFYVE16 | 0.68 | | 4.76E-282 | |
| Mono/Macro | Mono/Macro | GRINA | 0.91 | | 5.42E-282 | |
| Mono/Macro | Mono/Macro | FNDC3B | 0.97 | | 4.27E-280 | |
| Mono/Macro | Mono/Macro | LUCAT1 | 0.33 | | 1.20E-278 | |
| Mono/Macro | Mono/Macro | GLIPR2 | 0.78 | | 1.25E-278 | |
| Mono/Macro | Mono/Macro | PSAP | 1.13 | | 2.72E-277 | |
| Mono/Macro | Mono/Macro | CSF3R | 0.33 | | 1.03E-274 | |
| Mono/Macro | Mono/Macro | CD44 | 1.06 | | 9.16E-273 | |
| Mono/Macro | Mono/Macro | GNA15 | 0.68 | | 6.95E-270 | |
| Mono/Macro | Mono/Macro | THBD | 0.63 | | 9.15E-270 | |
| Mono/Macro | Mono/Macro | IRAK3 | 0.42 | | 5.02E-268 | |
| Mono/Macro | Mono/Macro | DOCK4 | 0.42 | | 2.81E-267 | |
| Mono/Macro | Mono/Macro | ATP1B3 | 1.23 | | 9.38E-267 | |
| Mono/Macro | Mono/Macro | GAPDH | 0.96 | | 1.01E-266 | |
| Mono/Macro | Mono/Macro | TNFSF15 | 0.91 | | 5.21E-264 | |
| Mono/Macro | Mono/Macro | ETS2 | 0.78 | | 8.56E-263 | |
| Mono/Macro | Mono/Macro | VIM | 1 | | 9.31E-262 | |
| Mono/Macro | Mono/Macro | CD163 | 0.56 | | 9.35E-262 | |
| Mono/Macro | Mono/Macro | BNIP3L | 1.18 | | 4.25E-261 | |
| Mono/Macro | Mono/Macro | NFKB1 | 1.02 | | 1.23E-260 | |
| Mono/Macro | Mono/Macro | ACTB | 0.87 | | 3.87E-260 | |
| Mono/Macro | Mono/Macro | AC020656.1 | 1 | | 1.50E-257 | |
| Mono/Macro | Mono/Macro | CYBB | 0.66 | | 1.33E-256 | |
| Mono/Macro | Mono/Macro | AC002456.1 | 0.36 | | 1.05E-254 | |
| Mono/Macro | Mono/Macro | CD300C | 0.33 | | 6.21E-254 | |
| Mono/Macro | Mono/Macro | STXBP2 | 1.08 | | 1.92E-249 | |
| Mono/Macro | Mono/Macro | IL1RAP | 0.42 | | 1.54E-245 | |
| Mono/Macro | Mono/Macro | STX11 | 0.93 | | 6.58E-245 | |
| Mono/Macro | Mono/Macro | CFD | 0.4 | | 4.94E-244 | |
| Mono/Macro | Mono/Macro | ATP6V1F | 1.02 | | 3.58E-241 | |
| Mono/Macro | Mono/Macro | LIMS1 | 0.95 | | 1.41E-239 | |
| Mono/Macro | Mono/Macro | ZEB2 | 0.76 | | 9.39E-239 | |
| Mono/Macro | Mono/Macro | GPX1 | 1.02 | | 2.48E-237 | |
| Mono/Macro | Mono/Macro | PTX3 | 0.4 | | 1.24E-235 | |
| Mono/Macro | Mono/Macro | RPL5 | -1.02 | | 4.84E-234 | |
| Mono/Macro | Mono/Macro | CTNNB1 | 0.97 | | 6.63E-234 | |
| Mono/Macro | Mono/Macro | H3F3A | 0.76 | | 9.31E-234 | |
| Mono/Macro | Mono/Macro | HMOX1 | 1.08 | | 2.01E-231 | |
| Mono/Macro | Mono/Macro | HBEGF | 0.81 | | 9.09E-231 | |
| Mono/Macro | Mono/Macro | SERPINB1 | 1.09 | | 6.87E-230 | |
| Mono/Macro | Mono/Macro | S100A4 | 1.12 | | 7.38E-229 | |
| Mono/Macro | Mono/Macro | CSTB | 1.78 | | 9.70E-229 | |
| Mono/Macro | Mono/Macro | ATP2B1 | 1.01 | | 5.94E-224 | |
| Mono/Macro | Mono/Macro | MS4A6A | 0.58 | | 3.95E-223 | |
| Mono/Macro | Mono/Macro | LITAF | 0.97 | | 8.88E-223 | |
| Mono/Macro | Mono/Macro | CTSS | 1.11 | | 4.12E-222 | |
| Mono/Macro | Mono/Macro | MALAT1 | -1 | | 1.50E-220 | |
| Mono/Macro | Mono/Macro | RNF130 | 0.71 | | 6.11E-219 | |
| Mono/Macro | Mono/Macro | BID | 0.85 | | 2.23E-218 | |
| Mono/Macro | Mono/Macro | ATP2B1-AS1 | 0.95 | | 1.92E-216 | |
| Mono/Macro | Mono/Macro | RPL23A | -0.79 | | 4.93E-216 | |
| Mono/Macro | Mono/Macro | IRAK2 | 0.54 | | 1.89E-215 | |
| Mono/Macro | Mono/Macro | NFKBIA | 1.19 | | 2.59E-215 | |
| Mono/Macro | Mono/Macro | RPS6 | -0.81 | | 9.44E-215 | |
| Mono/Macro | Mono/Macro | SLC7A7 | 0.63 | | 4.32E-214 | |
| Mono/Macro | Mono/Macro | ANXA2 | 0.84 | | 5.37E-214 | |
| Mono/Macro | Mono/Macro | PLD1 | 0.39 | | 9.96E-214 | |
| Mono/Macro | Mono/Macro | RPS18 | -0.73 | | 1.73E-213 | |
| Mono/Macro | Mono/Macro | RPSA | -1.04 | | 6.12E-212 | |
| Mono/Macro | Mono/Macro | EMP3 | 0.85 | | 6.41E-212 | |
| Mono/Macro | Mono/Macro | RPL13A | -0.69 | | 4.44E-209 | |
| Mono/Macro | Mono/Macro | MPP1 | 0.5 | | 1.56E-208 | |
| Mono/Macro | Mono/Macro | RAB20 | 0.59 | | 9.76E-208 | |
| Mono/Macro | Mono/Macro | RPS4X | -0.78 | | 1.65E-207 | |
| Mono/Macro | Mono/Macro | HAS1 | 0.39 | | 5.02E-204 | |
| Mono/Macro | Mono/Macro | PKM | 0.91 | | 2.10E-202 | |
| Mono/Macro | Mono/Macro | SH3BGRL3 | 0.76 | | 7.63E-200 | |
| Mono/Macro | Mono/Macro | LGALS3 | 0.95 | | 9.51E-200 | |
| Mono/Macro | Mono/Macro | ATP6V0B | 0.89 | | 3.43E-199 | |
| Mono/Macro | Mono/Macro | ABCA1 | 0.55 | | 1.49E-198 | |
| Mono/Macro | Mono/Macro | RPS3 | -0.75 | | 3.88E-198 | |
| Mono/Macro | Mono/Macro | MMP14 | 0.75 | | 6.47E-196 | |
| Mono/Macro | Mono/Macro | LPCAT1 | 0.61 | | 1.64E-194 | |
| Mono/Macro | Mono/Macro | SLC15A3 | 0.43 | | 3.01E-194 | |
| Mono/Macro | Mono/Macro | RPLP0 | -0.84 | | 5.58E-194 | |
| Mono/Macro | Mono/Macro | SDCBP | 0.8 | | 4.80E-191 | |
| Mono/Macro | Mono/Macro | PAPSS2 | 0.41 | | 4.71E-190 | |
| Mono/Macro | Mono/Macro | S100A6 | 0.6 | | 1.47E-189 | |
| Mono/Macro | Mono/Macro | TNIP3 | 0.63 | | 2.92E-188 | |
| Mono/Macro | Mono/Macro | CLEC12A | 0.32 | | 5.77E-188 | |
| Mono/Macro | Mono/Macro | MIR22HG | 0.7 | | 6.59E-186 | |
| Mono/Macro | Mono/Macro | RPS5 | -0.77 | | 1.62E-185 | |
| Mono/Macro | Mono/Macro | RPL4 | -0.88 | | 8.17E-185 | |
| Mono/Macro | Mono/Macro | SLC31A2 | 0.56 | | 8.95E-185 | |
| Mono/Macro | Mono/Macro | FCGR3A | 0.48 | | 1.25E-184 | |
| Mono/Macro | Mono/Macro | NOP53 | -1.06 | | 3.30E-184 | |
| Mono/Macro | Mono/Macro | RPS29 | -0.92 | | 4.82E-184 | |
| Mono/Macro | Mono/Macro | IL6 | 1.51 | | 3.13E-183 | |
| Mono/Macro | Mono/Macro | RPL27A | -0.66 | | 1.04E-182 | |
| Mono/Macro | Mono/Macro | CCR1 | 0.31 | | 1.09E-182 | |
| Mono/Macro | Mono/Macro | RPL31 | -0.77 | | 1.83E-182 | |
| Mono/Macro | Mono/Macro | TGM2 | 0.62 | | 3.81E-182 | |
| Mono/Macro | Mono/Macro | DUSP6 | 0.75 | | 9.28E-182 | |
| Mono/Macro | Mono/Macro | RPS2 | -0.65 | | 2.38E-181 | |
| Mono/Macro | Mono/Macro | PTPRE | 0.6 | | 6.13E-181 | |
| Mono/Macro | Mono/Macro | DRAM1 | 0.61 | | 6.28E-181 | |
| Mono/Macro | Mono/Macro | HLA-DRB5 | 0.75 | | 9.07E-179 | |
| Mono/Macro | Mono/Macro | MYL6 | 0.62 | | 4.82E-178 | |
| Mono/Macro | Mono/Macro | RPS19 | -0.61 | | 1.18E-177 | |
| Mono/Macro | Mono/Macro | HLA-DRA | 0.74 | | 1.49E-177 | |
| Mono/Macro | Mono/Macro | CARD16 | 0.88 | | 2.93E-177 | |
| Mono/Macro | Mono/Macro | RNF144B | 0.48 | | 2.49E-176 | |
| Mono/Macro | Mono/Macro | JUN | -1.76 | | 4.37E-175 | |
| Mono/Macro | Mono/Macro | NUMB | 0.71 | | 1.59E-172 | |
| Mono/Macro | Mono/Macro | CYBA | 0.61 | | 2.55E-172 | |
| Mono/Macro | Mono/Macro | EMILIN2 | 0.38 | | 3.01E-172 | |
| Mono/Macro | Mono/Macro | RPL7A | -0.69 | | 1.38E-171 | |
| Mono/Macro | Mono/Macro | FLNA | 0.73 | | 2.22E-171 | |
| Mono/Macro | Mono/Macro | FNIP2 | 0.66 | | 1.26E-170 | |
| Mono/Macro | Mono/Macro | RPS8 | -0.71 | | 1.33E-170 | |
| Mono/Macro | Mono/Macro | NRIP3 | 0.31 | | 1.69E-170 | |
| Mono/Macro | Mono/Macro | FLT1 | 0.42 | | 6.95E-169 | |
| Mono/Macro | Mono/Macro | RPL35 | -0.62 | | 1.04E-168 | |
| Mono/Macro | Mono/Macro | RPL14 | -0.68 | | 5.11E-168 | |
| Mono/Macro | Mono/Macro | IFITM3 | 0.67 | | 7.38E-168 | |
| Mono/Macro | Mono/Macro | RPL18 | -0.61 | | 1.92E-167 | |
| Mono/Macro | Mono/Macro | SH3BP5 | 0.58 | | 5.67E-167 | |
| Mono/Macro | Mono/Macro | RPS15A | -0.65 | | 1.11E-165 | |
| Mono/Macro | Mono/Macro | RPS25 | -0.66 | | 1.57E-165 | |
| Mono/Macro | Mono/Macro | LAIR1 | 0.41 | | 2.07E-165 | |
| Mono/Macro | Mono/Macro | CASP1 | 0.67 | | 3.22E-164 | |
| Mono/Macro | Mono/Macro | ALDOA | 0.76 | | 3.74E-164 | |
| Mono/Macro | Mono/Macro | SLC2A3 | 0.81 | | 5.67E-164 | |
| Mono/Macro | Mono/Macro | PTMA | -0.73 | | 1.39E-162 | |
| Mono/Macro | Mono/Macro | CXCL16 | 0.67 | | 4.69E-161 | |
| Mono/Macro | Mono/Macro | MGAT1 | 0.74 | | 7.31E-161 | |
| Mono/Macro | Mono/Macro | RAB13 | 0.84 | | 9.13E-161 | |
| Mono/Macro | Mono/Macro | ZFP36L2 | -1.49 | | 1.34E-160 | |
| Mono/Macro | Mono/Macro | KMO | 0.38 | | 2.18E-160 | |
| Mono/Macro | Mono/Macro | IL1R1 | 0.44 | | 2.58E-160 | |
| Mono/Macro | Mono/Macro | C1orf162 | 0.58 | | 3.21E-160 | |
| Mono/Macro | Mono/Macro | APLP2 | 0.76 | | 2.80E-159 | |
| Mono/Macro | Mono/Macro | HCST | 0.68 | | 5.90E-158 | |
| Mono/Macro | Mono/Macro | ARPC3 | 0.64 | | 2.46E-157 | |
| Mono/Macro | Mono/Macro | TET2 | 0.44 | | 2.06E-156 | |
| Mono/Macro | Mono/Macro | VMO1 | 0.76 | | 8.85E-156 | |
| Mono/Macro | Mono/Macro | SGK1 | 0.7 | | 2.46E-153 | |
| Mono/Macro | Mono/Macro | PLXDC2 | 0.36 | | 8.25E-153 | |
| Mono/Macro | Mono/Macro | RILPL2 | 0.74 | | 1.52E-152 | |
| Mono/Macro | Mono/Macro | RPL13 | -0.54 | | 5.83E-151 | |
| Mono/Macro | Mono/Macro | TNFAIP2 | 0.62 | | 6.63E-151 | |
| Mono/Macro | Mono/Macro | POMP | 0.75 | | 7.53E-151 | |
| Mono/Macro | Mono/Macro | RPL41 | -0.44 | | 1.11E-150 | |
| Mono/Macro | Mono/Macro | RPL10A | -0.65 | | 7.47E-150 | |
| Mono/Macro | Mono/Macro | RAC1 | 0.66 | | 1.33E-148 | |
| Mono/Macro | Mono/Macro | PLIN2 | 0.91 | | 1.46E-148 | |
| Mono/Macro | Mono/Macro | LCP1 | 0.66 | | 2.14E-147 | |
| Mono/Macro | Mono/Macro | EEF2 | -0.82 | | 2.61E-147 | |
| Mono/Macro | Mono/Macro | SLC39A8 | 0.93 | | 6.93E-147 | |
| Mono/Macro | Mono/Macro | CSF2RA | 0.36 | | 9.42E-147 | |
| Mono/Macro | Mono/Macro | SEC61G | 0.8 | | 1.89E-146 | |
| Mono/Macro | Mono/Macro | QKI | 0.55 | | 1.90E-146 | |
| Mono/Macro | Mono/Macro | EAF1 | 0.51 | | 2.21E-146 | |
| Mono/Macro | Mono/Macro | RPL15 | -0.54 | | 4.23E-146 | |
| Mono/Macro | Mono/Macro | WARS | 0.82 | | 2.73E-144 | |
| Mono/Macro | Mono/Macro | RPS27 | -0.74 | | 2.21E-143 | |
| Mono/Macro | Mono/Macro | RNF19B | 0.56 | | 3.15E-143 | |
| Mono/Macro | Mono/Macro | TMSB10 | 0.51 | | 2.41E-142 | |
| Mono/Macro | Mono/Macro | ARL8B | 0.61 | | 8.93E-142 | |
| Mono/Macro | Mono/Macro | TNFSF13B | 0.58 | | 8.94E-142 | |
| Mono/Macro | Mono/Macro | SRSF7 | -1.16 | | 1.47E-141 | |
| Mono/Macro | Mono/Macro | MS4A4A | 0.31 | | 1.64E-141 | |
| Mono/Macro | Mono/Macro | PNP | 0.79 | | 1.22E-140 | |
| Mono/Macro | Mono/Macro | SNX10 | 0.5 | | 3.57E-140 | |
| Mono/Macro | Mono/Macro | CRADD | 0.66 | | 4.43E-140 | |
| Mono/Macro | Mono/Macro | EHD1 | 0.8 | | 5.98E-139 | |
| Mono/Macro | Mono/Macro | SMS | 0.75 | | 1.52E-138 | |
| Mono/Macro | Mono/Macro | PTPN12 | 0.52 | | 2.28E-138 | |
| Mono/Macro | Mono/Macro | TPI1 | 0.8 | | 2.36E-138 | |
| Mono/Macro | Mono/Macro | CCL2 | 1.27 | | 2.95E-138 | |
| Mono/Macro | Mono/Macro | PTPRCAP | -1.29 | | 2.80E-137 | |
| Mono/Macro | Mono/Macro | TSC22D3 | -1.33 | | 3.21E-137 | |
| Mono/Macro | Mono/Macro | SERF2 | 0.49 | | 3.94E-135 | |
| Mono/Macro | Mono/Macro | RPS10 | -0.82 | | 9.85E-135 | |
| Mono/Macro | Mono/Macro | RABGEF1 | 0.29 | | 9.71E-134 | |
| Mono/Macro | Mono/Macro | CTSH | 0.75 | | 1.22E-133 | |
| Mono/Macro | Mono/Macro | MAP2K1 | 0.63 | | 7.34E-133 | |
| Mono/Macro | Mono/Macro | IL10RB-DT | 0.25 | | 8.36E-133 | |
| Mono/Macro | Mono/Macro | INSIG1 | 0.85 | | 1.17E-132 | |
| Mono/Macro | Mono/Macro | RPL19 | -0.55 | | 4.91E-132 | |
| Mono/Macro | Mono/Macro | TNFRSF1B | 0.62 | | 1.37E-131 | |
| Mono/Macro | Mono/Macro | CXCL5 | 2.62 | | 2.91E-130 | |
| Mono/Macro | Mono/Macro | IL6R | 0.45 | | 1.87E-129 | |
| Mono/Macro | Mono/Macro | FOSL2 | 0.67 | | 2.19E-129 | |
| Mono/Macro | Mono/Macro | NEAT1 | 0.59 | | 9.39E-128 | |
| Mono/Macro | Mono/Macro | HLA-DRB1 | 0.53 | | 3.40E-127 | |
| Mono/Macro | Mono/Macro | ZNF267 | 0.57 | | 7.65E-127 | |
| Mono/Macro | Mono/Macro | TXN | 0.93 | | 1.23E-126 | |
| Mono/Macro | Mono/Macro | PIK3AP1 | 0.4 | | 1.35E-126 | |
| Mono/Macro | Mono/Macro | RPS27A | -0.53 | | 2.42E-126 | |
| Mono/Macro | Mono/Macro | RPL36 | -0.55 | | 1.95E-125 | |
| Mono/Macro | Mono/Macro | RPS21 | -0.76 | | 2.90E-125 | |
| Mono/Macro | Mono/Macro | CHMP1B | 0.87 | | 5.49E-124 | |
| Mono/Macro | Mono/Macro | GNS | 0.51 | | 5.75E-124 | |
| Mono/Macro | Mono/Macro | NOTCH2 | 0.39 | | 1.40E-123 | |
| Mono/Macro | Mono/Macro | TNFRSF1A | 0.62 | | 1.52E-123 | |
| Mono/Macro | Mono/Macro | ATP6V1B2 | 0.57 | | 3.29E-123 | |
| Mono/Macro | Mono/Macro | ELL2 | 0.62 | | 5.03E-123 | |
| Mono/Macro | Mono/Macro | PTGER2 | 0.4 | | 2.90E-122 | |
| Mono/Macro | Mono/Macro | EEF1A1 | -0.55 | | 6.72E-122 | |
| Mono/Macro | Mono/Macro | RPS15 | -0.44 | | 2.13E-121 | |
| Mono/Macro | Mono/Macro | GRN | 0.67 | | 3.84E-118 | |
| Mono/Macro | Mono/Macro | SARAF | -1.09 | | 8.11E-118 | |
| Mono/Macro | Mono/Macro | SMOX | 0.57 | | 7.93E-117 | |
| Mono/Macro | Mono/Macro | RPL11 | -0.5 | | 9.98E-117 | |
| Mono/Macro | Mono/Macro | JARID2 | 0.5 | | 1.03E-116 | |
| Mono/Macro | Mono/Macro | OAZ1 | 0.44 | | 2.16E-116 | |
| Mono/Macro | Mono/Macro | FAM49A | 0.55 | | 2.62E-116 | |
| Mono/Macro | Mono/Macro | ENO1 | 0.66 | | 5.06E-116 | |
| Mono/Macro | Mono/Macro | JUNB | -1.27 | | 7.59E-116 | |
| Mono/Macro | Mono/Macro | SIRPA | 0.36 | | 1.26E-115 | |
| Mono/Macro | Mono/Macro | GNG5 | 0.62 | | 1.63E-115 | |
| Mono/Macro | Mono/Macro | WTAP | 0.84 | | 2.55E-115 | |
| Mono/Macro | Mono/Macro | TPT1 | 0.38 | | 4.50E-115 | |
| Mono/Macro | Mono/Macro | EIF4A2 | -0.91 | | 5.13E-115 | |
| Mono/Macro | Mono/Macro | CAPG | 0.65 | | 3.67E-114 | |
| Mono/Macro | Mono/Macro | ABL2 | 0.51 | | 1.31E-112 | |
| Mono/Macro | Mono/Macro | NPM1 | -0.69 | | 1.43E-112 | |
| Mono/Macro | Mono/Macro | B3GNT5 | 0.49 | | 3.49E-112 | |
| Mono/Macro | Mono/Macro | PNRC1 | 0.71 | | 4.03E-112 | |
| Mono/Macro | Mono/Macro | RPS14 | -0.45 | | 8.20E-112 | |
| Mono/Macro | Mono/Macro | SLC16A3 | 0.67 | | 1.04E-111 | |
| Mono/Macro | Mono/Macro | HK2 | 0.35 | | 1.40E-111 | |
| Mono/Macro | Mono/Macro | ARFGAP3 | 0.62 | | 1.33E-110 | |
| Mono/Macro | Mono/Macro | DNAAF1 | 0.43 | | 1.57E-110 | |
| Mono/Macro | Mono/Macro | RPL18A | -0.5 | | 4.80E-110 | |
| Mono/Macro | Mono/Macro | ZNF385A | 0.3 | | 4.99E-110 | |
| Mono/Macro | Mono/Macro | C4orf3 | 0.78 | | 7.23E-110 | |
| Mono/Macro | Mono/Macro | PHLDA2 | 0.68 | | 1.62E-109 | |
| Mono/Macro | Mono/Macro | CALHM6 | 0.56 | | 2.05E-109 | |
| Mono/Macro | Mono/Macro | ITGA5 | 0.35 | | 3.74E-109 | |
| Mono/Macro | Mono/Macro | ACSL4 | 0.43 | | 8.18E-109 | |
| Mono/Macro | Mono/Macro | RPS3A | -0.54 | | 6.39E-108 | |
| Mono/Macro | Mono/Macro | PFN1 | 0.44 | | 1.00E-107 | |
| Mono/Macro | Mono/Macro | SERPINB8 | 0.38 | | 2.41E-107 | |
| Mono/Macro | Mono/Macro | YPEL5 | -1.06 | | 8.89E-107 | |
| Mono/Macro | Mono/Macro | ITGB8 | 0.43 | | 1.03E-106 | |
| Mono/Macro | Mono/Macro | CREG1 | 0.64 | | 6.65E-106 | |
| Mono/Macro | Mono/Macro | JCHAIN | -4 | | 7.81E-106 | |
| Mono/Macro | Mono/Macro | RPL30 | -0.52 | | 2.36E-105 | |
| Mono/Macro | Mono/Macro | RPL6 | -0.49 | | 3.87E-105 | |
| Mono/Macro | Mono/Macro | TPP1 | 0.54 | | 5.73E-105 | |
| Mono/Macro | Mono/Macro | ATP5F1E | 0.45 | | 9.16E-105 | |
| Mono/Macro | Mono/Macro | IL32 | -1.47 | | 1.96E-104 | |
| Mono/Macro | Mono/Macro | TWISTNB | 0.95 | | 5.48E-104 | |
| Mono/Macro | Mono/Macro | NBPF9 | 0.43 | | 2.00E-103 | |
| Mono/Macro | Mono/Macro | CFLAR | 0.62 | | 2.16E-103 | |
| Mono/Macro | Mono/Macro | RACK1 | -0.51 | | 2.55E-103 | |
| Mono/Macro | Mono/Macro | MAP3K8 | 0.71 | | 1.36E-102 | |
| Mono/Macro | Mono/Macro | GCH1 | 0.5 | | 1.42E-102 | |
| Mono/Macro | Mono/Macro | RPL29 | -0.49 | | 2.30E-102 | |
| Mono/Macro | Mono/Macro | RPL32 | -0.46 | | 2.36E-102 | |
| Mono/Macro | Mono/Macro | HINT1 | -0.7 | | 1.00E-101 | |
| Mono/Macro | Mono/Macro | HNRNPA1 | -0.62 | | 2.13E-101 | |
| Mono/Macro | Mono/Macro | MSC | 0.34 | | 1.78E-100 | |
| Mono/Macro | Mono/Macro | TOM1 | 0.41 | | 2.13E-100 | |
| Mono/Macro | Mono/Macro | FCGRT | 0.56 | | 3.46E-100 | |
| Mono/Macro | Mono/Macro | CSF1R | 0.29 | | 2.42E-99 | |
| Mono/Macro | Mono/Macro | YBX3 | 0.59 | | 3.24E-99 | |
| Mono/Macro | Mono/Macro | MIR4435-2HG | 0.69 | | 3.95E-98 | |
| Mono/Macro | Mono/Macro | ZMIZ1 | 0.4 | | 1.27E-97 | |
| Mono/Macro | Mono/Macro | PICALM | 0.47 | | 1.63E-97 | |
| Mono/Macro | Mono/Macro | EEF1B2 | -0.66 | | 1.69E-97 | |
| Mono/Macro | Mono/Macro | LGALS2 | 0.45 | | 2.29E-97 | |
| Mono/Macro | Mono/Macro | SH2B3 | 0.42 | | 2.56E-97 | |
| Mono/Macro | Mono/Macro | CSGALNACT2 | 0.45 | | 4.22E-97 | |
| Mono/Macro | Mono/Macro | RPS7 | -0.47 | | 8.38E-97 | |
| Mono/Macro | Mono/Macro | AP2S1 | 0.64 | | 8.79E-97 | |
| Mono/Macro | Mono/Macro | RPS12 | -0.51 | | 1.28E-96 | |
| Mono/Macro | Mono/Macro | TBXAS1 | 0.41 | | 4.46E-96 | |
| Mono/Macro | Mono/Macro | EMP1 | 0.34 | | 4.50E-96 | |
| Mono/Macro | Mono/Macro | RIN3 | 0.34 | | 2.49E-94 | |
| Mono/Macro | Mono/Macro | HS3ST3B1 | 0.26 | | 1.86E-93 | |
| Mono/Macro | Mono/Macro | GPX4 | 0.6 | | 1.06E-92 | |
| Mono/Macro | Mono/Macro | MALT1 | 0.42 | | 1.23E-92 | |
| Mono/Macro | Mono/Macro | MT2A | 1.67 | | 3.29E-92 | |
| Mono/Macro | Mono/Macro | SIPA1L1 | 0.41 | | 1.05E-91 | |
| Mono/Macro | Mono/Macro | RPL10 | -0.36 | | 1.26E-91 | |
| Mono/Macro | Mono/Macro | RHOQ | 0.34 | | 5.12E-91 | |
| Mono/Macro | Mono/Macro | AP1S2 | 0.51 | | 6.69E-91 | |
| Mono/Macro | Mono/Macro | HSPA8 | -0.85 | | 3.17E-90 | |
| Mono/Macro | Mono/Macro | NOP10 | 0.64 | | 3.61E-90 | |
| Mono/Macro | Mono/Macro | SRC | 0.36 | | 2.18E-89 | |
| Mono/Macro | Mono/Macro | PEBP1 | -0.79 | | 1.04E-88 | |
| Mono/Macro | Mono/Macro | ARPC5 | 0.56 | | 1.55E-88 | |
| Mono/Macro | Mono/Macro | EZR | -0.9 | | 2.82E-88 | |
| Mono/Macro | Mono/Macro | TSPO | 0.57 | | 1.64E-87 | |
| Mono/Macro | Mono/Macro | IDO1 | 0.43 | | 2.31E-87 | |
| Mono/Macro | Mono/Macro | COTL1 | 0.62 | | 3.71E-87 | |
| Mono/Macro | Mono/Macro | FPR3 | 0.27 | | 3.75E-87 | |
| Mono/Macro | Mono/Macro | RHOA | 0.48 | | 3.47E-86 | |
| Mono/Macro | Mono/Macro | MNDA | 0.38 | | 4.41E-86 | |
| Mono/Macro | Mono/Macro | ZFP36 | -1.02 | | 4.72E-86 | |
| Mono/Macro | Mono/Macro | CD79A | -1.95 | | 6.91E-86 | |
| Mono/Macro | Mono/Macro | MARCKSL1 | 0.72 | | 1.94E-85 | |
| Mono/Macro | Mono/Macro | CD69 | -1.49 | | 2.14E-85 | |
| Mono/Macro | Mono/Macro | ACTN1 | 0.45 | | 1.09E-84 | |
| Mono/Macro | Mono/Macro | ACOT9 | 0.46 | | 7.09E-84 | |
| Mono/Macro | Mono/Macro | CXCR4 | -1.33 | | 2.56E-83 | |
| Mono/Macro | Mono/Macro | OGFRL1 | 0.39 | | 4.11E-83 | |
| Mono/Macro | Mono/Macro | RXRA | 0.35 | | 2.05E-82 | |
| Mono/Macro | Mono/Macro | RPL8 | -0.41 | | 1.22E-81 | |
| Mono/Macro | Mono/Macro | B4GALT5 | 0.43 | | 5.35E-81 | |
| Mono/Macro | Mono/Macro | TBC1D7 | 0.34 | | 5.46E-81 | |
| Mono/Macro | Mono/Macro | PLEKHO2 | 0.33 | | 3.97E-80 | |
| Mono/Macro | Mono/Macro | RPS20 | -0.41 | | 6.30E-80 | |
| Mono/Macro | Mono/Macro | METRNL | 0.43 | | 5.73E-79 | |
| Mono/Macro | Mono/Macro | GPR137B | 0.44 | | 8.70E-79 | |
| Mono/Macro | Mono/Macro | SF1 | -0.73 | | 8.86E-79 | |
| Mono/Macro | Mono/Macro | MAP2K3 | 0.53 | | 1.23E-78 | |
| Mono/Macro | Mono/Macro | RPL21 | -0.41 | | 1.30E-78 | |
| Mono/Macro | Mono/Macro | RPL36A | -0.71 | | 1.94E-78 | |
| Mono/Macro | Mono/Macro | SMPDL3A | 0.32 | | 2.05E-78 | |
| Mono/Macro | Mono/Macro | RPL12 | -0.4 | | 3.36E-78 | |
| Mono/Macro | Mono/Macro | MYL12A | 0.46 | | 5.55E-78 | |
| Mono/Macro | Mono/Macro | CLIC1 | 0.49 | | 5.71E-78 | |
| Mono/Macro | Mono/Macro | UBC | -0.59 | | 1.10E-77 | |
| Mono/Macro | Mono/Macro | DPYD | 0.26 | | 1.47E-77 | |
| Mono/Macro | Mono/Macro | RPL34 | -0.42 | | 2.46E-77 | |
| Mono/Macro | Mono/Macro | RAB32 | 0.38 | | 2.73E-77 | |
| Mono/Macro | Mono/Macro | ATP6V0D1 | 0.52 | | 7.58E-77 | |
| Mono/Macro | Mono/Macro | CTSD | 0.5 | | 3.99E-75 | |
| Mono/Macro | Mono/Macro | MPEG1 | 0.28 | | 6.26E-75 | |
| Mono/Macro | Mono/Macro | EIF1 | 0.29 | | 1.87E-74 | |
| Mono/Macro | Mono/Macro | RALA | 0.51 | | 3.96E-74 | |
| Mono/Macro | Mono/Macro | THAP2 | 0.44 | | 4.08E-74 | |
| Mono/Macro | Mono/Macro | PDE4A | 0.37 | | 4.39E-74 | |
| Mono/Macro | Mono/Macro | ERP29 | -0.66 | | 1.31E-73 | |
| Mono/Macro | Mono/Macro | FCGR2B | 0.62 | | 1.86E-73 | |
| Mono/Macro | Mono/Macro | ABHD17C | 0.37 | | 3.48E-73 | |
| Mono/Macro | Mono/Macro | ARHGDIB | -0.88 | | 3.51E-73 | |
| Mono/Macro | Mono/Macro | HSP90AB1 | -0.6 | | 3.64E-73 | |
| Mono/Macro | Mono/Macro | HNRNPDL | -0.66 | | 5.84E-73 | |
| Mono/Macro | Mono/Macro | NR4A3 | 0.45 | | 1.11E-72 | |
| Mono/Macro | Mono/Macro | RNF13 | 0.46 | | 1.95E-72 | |
| Mono/Macro | Mono/Macro | PIK3R5 | 0.26 | | 2.77E-72 | |
| Mono/Macro | Mono/Macro | MT-ND2 | -0.7 | | 3.13E-72 | |
| Mono/Macro | Mono/Macro | EIF3E | -0.64 | | 3.45E-72 | |
| Mono/Macro | Mono/Macro | RPS23 | -0.43 | | 3.55E-72 | |
| Mono/Macro | Mono/Macro | SLC2A6 | 0.28 | | 4.16E-72 | |
| Mono/Macro | Mono/Macro | SOD1 | -0.68 | | 7.53E-72 | |
| Mono/Macro | Mono/Macro | MT-CYB | -0.79 | | 7.88E-72 | |
| Mono/Macro | Mono/Macro | RPLP1 | -0.36 | | 1.03E-71 | |
| Mono/Macro | Mono/Macro | KLF4 | 0.4 | | 1.74E-71 | |
| Mono/Macro | Mono/Macro | MCL1 | 0.41 | | 2.94E-71 | |
| Mono/Macro | Mono/Macro | ITM2A | -0.98 | | 1.33E-70 | |
| Mono/Macro | Mono/Macro | RGS10 | 0.47 | | 1.79E-70 | |
| Mono/Macro | Mono/Macro | RPL37 | -0.43 | | 2.56E-70 | |
| Mono/Macro | Mono/Macro | RPL17 | -0.8 | | 2.67E-70 | |
| Mono/Macro | Mono/Macro | ERGIC1 | 0.48 | | 2.80E-70 | |
| Mono/Macro | Mono/Macro | SLC43A3 | 0.31 | | 5.89E-70 | |
| Mono/Macro | Mono/Macro | TRAF1 | 0.33 | | 1.41E-69 | |
| Mono/Macro | Mono/Macro | RPL7 | -0.38 | | 1.55E-69 | |
| Mono/Macro | Mono/Macro | SNU13 | -0.65 | | 2.50E-69 | |
| Mono/Macro | Mono/Macro | TUBA1A | -0.96 | | 7.03E-69 | |
| Mono/Macro | Mono/Macro | RBM47 | 0.42 | | 7.29E-69 | |
| Mono/Macro | Mono/Macro | PFDN5 | 0.36 | | 1.02E-68 | |
| Mono/Macro | Mono/Macro | HMGB1 | -0.64 | | 1.40E-68 | |
| Mono/Macro | Mono/Macro | LEPROTL1 | -0.96 | | 1.52E-68 | |
| Mono/Macro | Mono/Macro | ITGAV | 0.36 | | 2.15E-68 | |
| Mono/Macro | Mono/Macro | OCIAD2 | -0.71 | | 3.32E-68 | |
| Mono/Macro | Mono/Macro | UBB | -0.49 | | 3.77E-68 | |
| Mono/Macro | Mono/Macro | CDKN1A | 0.38 | | 5.88E-68 | |
| Mono/Macro | Mono/Macro | IER2 | -0.91 | | 1.26E-67 | |
| Mono/Macro | Mono/Macro | RPLP2 | -0.45 | | 3.54E-67 | |
| Mono/Macro | Mono/Macro | ITGB1 | 0.43 | | 4.59E-67 | |
| Mono/Macro | Mono/Macro | SFT2D1 | 0.51 | | 4.82E-67 | |
| Mono/Macro | Mono/Macro | DUSP4 | 0.42 | | 5.12E-67 | |
| Mono/Macro | Mono/Macro | PEA15 | 0.44 | | 8.70E-67 | |
| Mono/Macro | Mono/Macro | ADM | 0.36 | | 1.08E-66 | |
| Mono/Macro | Mono/Macro | ODC1 | -0.73 | | 1.37E-66 | |
| Mono/Macro | Mono/Macro | LY96 | 0.43 | | 1.99E-66 | |
| Mono/Macro | Mono/Macro | CD3D | -1.31 | | 2.76E-66 | |
| Mono/Macro | Mono/Macro | SLC44A1 | 0.51 | | 6.75E-66 | |
| Mono/Macro | Mono/Macro | DDX24 | -0.73 | | 1.62E-65 | |
| Mono/Macro | Mono/Macro | TNFSF14 | 0.31 | | 1.65E-65 | |
| Mono/Macro | Mono/Macro | ANGPTL4 | 0.39 | | 6.47E-65 | |
| Mono/Macro | Mono/Macro | TLNRD1 | 0.4 | | 7.71E-65 | |
| Mono/Macro | Mono/Macro | RPL9 | -0.38 | | 9.57E-65 | |
| Mono/Macro | Mono/Macro | RSL24D1 | -0.65 | | 1.63E-64 | |
| Mono/Macro | Mono/Macro | MFSD2A | 0.3 | | 1.73E-64 | |
| Mono/Macro | Mono/Macro | MPHOSPH6 | 0.39 | | 7.07E-64 | |
| Mono/Macro | Mono/Macro | H3F3B | 0.27 | | 1.24E-63 | |
| Mono/Macro | Mono/Macro | RNF149 | 0.47 | | 2.09E-63 | |
| Mono/Macro | Mono/Macro | HSPE1 | -0.46 | | 3.97E-63 | |
| Mono/Macro | Mono/Macro | PRDX2 | -0.65 | | 6.00E-63 | |
| Mono/Macro | Mono/Macro | CD7 | -1.35 | | 1.43E-62 | |
| Mono/Macro | Mono/Macro | NSD3 | -0.65 | | 1.99E-62 | |
| Mono/Macro | Mono/Macro | FKBP1A | 0.42 | | 2.18E-62 | |
| Mono/Macro | Mono/Macro | CIRBP | -0.58 | | 4.29E-62 | |
| Mono/Macro | Mono/Macro | LACTB | 0.35 | | 5.61E-62 | |
| Mono/Macro | Mono/Macro | CYTIP | -0.82 | | 6.92E-62 | |
| Mono/Macro | Mono/Macro | FKBP11 | -1.17 | | 7.07E-62 | |
| Mono/Macro | Mono/Macro | RPL35A | -0.31 | | 7.36E-62 | |
| Mono/Macro | Mono/Macro | AK4 | 0.27 | | 7.79E-62 | |
| Mono/Macro | Mono/Macro | PGK1 | 0.5 | | 8.22E-62 | |
| Mono/Macro | Mono/Macro | ARRB2 | 0.4 | | 1.34E-61 | |
| Mono/Macro | Mono/Macro | LGMN | 0.42 | | 3.10E-61 | |
| Mono/Macro | Mono/Macro | RUNX1 | 0.41 | | 4.24E-61 | |
| Mono/Macro | Mono/Macro | TALDO1 | 0.53 | | 5.81E-61 | |
| Mono/Macro | Mono/Macro | LTB | -1.07 | | 7.28E-61 | |
| Mono/Macro | Mono/Macro | GPR35 | 0.29 | | 3.90E-60 | |
| Mono/Macro | Mono/Macro | RAB8B | 0.36 | | 4.12E-60 | |
| Mono/Macro | Mono/Macro | CD3E | -1.05 | | 4.14E-60 | |
| Mono/Macro | Mono/Macro | IFI6 | 0.68 | | 8.83E-60 | |
| Mono/Macro | Mono/Macro | SBDS | -0.64 | | 9.20E-60 | |
| Mono/Macro | Mono/Macro | SAMSN1 | 0.41 | | 1.53E-59 | |
| Mono/Macro | Mono/Macro | LDHB | -0.7 | | 3.42E-59 | |
| Mono/Macro | Mono/Macro | RIPK2 | 0.38 | | 9.17E-59 | |
| Mono/Macro | Mono/Macro | RTN4 | 0.43 | | 1.45E-58 | |
| Mono/Macro | Mono/Macro | EIF5B | -0.59 | | 1.73E-58 | |
| Mono/Macro | Mono/Macro | LIMD2 | -0.74 | | 2.07E-58 | |
| Mono/Macro | Mono/Macro | UBA52 | 0.27 | | 3.30E-58 | |
| Mono/Macro | Mono/Macro | ARPC2 | 0.35 | | 4.36E-58 | |
| Mono/Macro | Mono/Macro | NRIP1 | 0.33 | | 4.61E-58 | |
| Mono/Macro | Mono/Macro | CHMP4B | 0.45 | | 4.64E-58 | |
| Mono/Macro | Mono/Macro | MAP3K20 | 0.3 | | 4.75E-58 | |
| Mono/Macro | Mono/Macro | SPOCK2 | -0.9 | | 8.47E-58 | |
| Mono/Macro | Mono/Macro | ATP6V0E1 | 0.44 | | 8.73E-58 | |
| Mono/Macro | Mono/Macro | QSOX1 | 0.4 | | 1.90E-57 | |
| Mono/Macro | Mono/Macro | PPDPF | -0.68 | | 1.92E-57 | |
| Mono/Macro | Mono/Macro | TUBA4A | -0.93 | | 2.14E-57 | |
| Mono/Macro | Mono/Macro | LYN | 0.41 | | 2.81E-57 | |
| Mono/Macro | Mono/Macro | RP2 | 0.31 | | 3.29E-57 | |
| Mono/Macro | Mono/Macro | RAB1A | 0.46 | | 4.74E-57 | |
| Mono/Macro | Mono/Macro | PPP1R15B | 0.45 | | 5.00E-57 | |
| Mono/Macro | Mono/Macro | SVIP | -0.57 | | 1.26E-56 | |
| Mono/Macro | Mono/Macro | HES4 | 0.49 | | 2.62E-56 | |
| Mono/Macro | Mono/Macro | GNA12 | 0.27 | | 2.89E-56 | |
| Mono/Macro | Mono/Macro | BRI3 | 0.44 | | 3.04E-56 | |
| Mono/Macro | Mono/Macro | TMEM107 | 0.57 | | 5.56E-56 | |
| Mono/Macro | Mono/Macro | CORO1C | 0.41 | | 6.87E-56 | |
| Mono/Macro | Mono/Macro | CDC42EP2 | 0.28 | | 9.24E-56 | |
| Mono/Macro | Mono/Macro | TECR | -0.6 | | 1.06E-55 | |
| Mono/Macro | Mono/Macro | HIST1H4C | -0.66 | | 1.10E-55 | |
| Mono/Macro | Mono/Macro | IL10RB | 0.38 | | 1.11E-55 | |
| Mono/Macro | Mono/Macro | CD58 | 0.38 | | 1.11E-55 | |
| Mono/Macro | Mono/Macro | PNISR | -0.62 | | 1.38E-55 | |
| Mono/Macro | Mono/Macro | TNIP1 | 0.49 | | 1.47E-55 | |
| Mono/Macro | Mono/Macro | CD55 | 0.39 | | 2.51E-55 | |
| Mono/Macro | Mono/Macro | RIT1 | 0.38 | | 4.42E-55 | |
| Mono/Macro | Mono/Macro | H2AFY | 0.45 | | 4.61E-55 | |
| Mono/Macro | Mono/Macro | AREG | 0.52 | | 5.46E-55 | |
| Mono/Macro | Mono/Macro | PDLIM7 | 0.35 | | 6.38E-55 | |
| Mono/Macro | Mono/Macro | BTG2 | -0.83 | | 6.85E-55 | |
| Mono/Macro | Mono/Macro | ERO1A | 0.47 | | 8.26E-55 | |
| Mono/Macro | Mono/Macro | PRKAG2 | 0.32 | | 1.03E-54 | |
| Mono/Macro | Mono/Macro | ELOC | 0.42 | | 1.40E-54 | |
| Mono/Macro | Mono/Macro | RGCC | 0.37 | | 1.84E-54 | |
| Mono/Macro | Mono/Macro | CCDC85B | -0.62 | | 1.97E-54 | |
| Mono/Macro | Mono/Macro | MZT2A | -0.55 | | 9.63E-54 | |
| Mono/Macro | Mono/Macro | ADAM8 | 0.33 | | 1.42E-53 | |
| Mono/Macro | Mono/Macro | GPCPD1 | 0.37 | | 1.47E-53 | |
| Mono/Macro | Mono/Macro | BZW1 | 0.48 | | 1.84E-53 | |
| Mono/Macro | Mono/Macro | CCDC12 | -0.56 | | 3.53E-53 | |
| Mono/Macro | Mono/Macro | MYO1G | 0.33 | | 4.44E-53 | |
| Mono/Macro | Mono/Macro | CNBP | -0.54 | | 4.93E-53 | |
| Mono/Macro | Mono/Macro | FAM102B | 0.25 | | 5.71E-53 | |
| Mono/Macro | Mono/Macro | ARL5B | 0.4 | | 5.99E-53 | |
| Mono/Macro | Mono/Macro | BACH1 | 0.38 | | 1.22E-52 | |
| Mono/Macro | Mono/Macro | RPL24 | -0.32 | | 1.60E-52 | |
| Mono/Macro | Mono/Macro | TENT5C | -0.67 | | 1.60E-52 | |
| Mono/Macro | Mono/Macro | ACTR2 | 0.41 | | 3.45E-52 | |
| Mono/Macro | Mono/Macro | N4BP2L2 | -0.58 | | 3.56E-51 | |
| Mono/Macro | Mono/Macro | GPNMB | 0.28 | | 4.31E-51 | |
| Mono/Macro | Mono/Macro | HNRNPA0 | -0.57 | | 7.87E-51 | |
| Mono/Macro | Mono/Macro | OAZ2 | 0.39 | | 9.24E-51 | |
| Mono/Macro | Mono/Macro | RBMX | -0.56 | | 1.48E-50 | |
| Mono/Macro | Mono/Macro | IL13RA1 | 0.3 | | 1.57E-50 | |
| Mono/Macro | Mono/Macro | B4GALT1 | 0.5 | | 1.83E-50 | |
| Mono/Macro | Mono/Macro | CDC42SE2 | -0.66 | | 1.91E-50 | |
| Mono/Macro | Mono/Macro | AC058791.1 | -0.68 | | 3.13E-50 | |
| Mono/Macro | Mono/Macro | RAB7A | 0.48 | | 3.59E-50 | |
| Mono/Macro | Mono/Macro | GNA13 | 0.4 | | 7.19E-50 | |
| Mono/Macro | Mono/Macro | CD37 | -0.83 | | 7.27E-50 | |
| Mono/Macro | Mono/Macro | C6orf48 | -0.6 | | 9.48E-50 | |
| Mono/Macro | Mono/Macro | GMFG | 0.36 | | 1.20E-49 | |
| Mono/Macro | Mono/Macro | ATP6V1C1 | 0.34 | | 1.40E-49 | |
| Mono/Macro | Mono/Macro | LYPD3 | 0.37 | | 2.23E-49 | |
| Mono/Macro | Mono/Macro | ACSL5 | 0.37 | | 3.10E-49 | |
| Mono/Macro | Mono/Macro | NCL | -0.57 | | 6.26E-49 | |
| Mono/Macro | Mono/Macro | AC016831.4 | -0.69 | | 8.68E-49 | |
| Mono/Macro | Mono/Macro | LINC-PINT | 0.32 | | 9.52E-49 | |
| Mono/Macro | Mono/Macro | TPM4 | 0.42 | | 1.13E-48 | |
| Mono/Macro | Mono/Macro | MAFF | 0.38 | | 1.54E-48 | |
| Mono/Macro | Mono/Macro | HSBP1 | 0.39 | | 1.71E-48 | |
| Mono/Macro | Mono/Macro | HIVEP2 | 0.3 | | 2.00E-48 | |
| Mono/Macro | Mono/Macro | STX4 | 0.42 | | 3.24E-48 | |
| Mono/Macro | Mono/Macro | MZT2B | -0.53 | | 6.11E-48 | |
| Mono/Macro | Mono/Macro | ACSL3 | 0.38 | | 6.41E-48 | |
| Mono/Macro | Mono/Macro | VAMP2 | -0.58 | | 9.89E-48 | |
| Mono/Macro | Mono/Macro | TCIRG1 | 0.4 | | 1.02E-47 | |
| Mono/Macro | Mono/Macro | AGTRAP | 0.33 | | 1.33E-47 | |
| Mono/Macro | Mono/Macro | HNMT | 0.32 | | 1.90E-47 | |
| Mono/Macro | Mono/Macro | RBM39 | -0.52 | | 2.71E-47 | |
| Mono/Macro | Mono/Macro | SRSF2 | -0.59 | | 1.15E-46 | |
| Mono/Macro | Mono/Macro | RAB10 | 0.41 | | 1.25E-46 | |
| Mono/Macro | Mono/Macro | RPS11 | -0.32 | | 1.31E-46 | |
| Mono/Macro | Mono/Macro | RPL26 | -0.3 | | 1.52E-46 | |
| Mono/Macro | Mono/Macro | STK4 | -0.63 | | 5.49E-46 | |
| Mono/Macro | Mono/Macro | TUBA1B | -0.64 | | 6.71E-46 | |
| Mono/Macro | Mono/Macro | SPAG9 | 0.38 | | 7.34E-46 | |
| Mono/Macro | Mono/Macro | ARID5B | -0.68 | | 9.46E-46 | |
| Mono/Macro | Mono/Macro | ITM2C | -0.85 | | 1.06E-45 | |
| Mono/Macro | Mono/Macro | C9orf72 | 0.26 | | 2.02E-45 | |
| Mono/Macro | Mono/Macro | GPAT3 | 0.25 | | 2.47E-45 | |
| Mono/Macro | Mono/Macro | AGAP3 | 0.26 | | 3.37E-45 | |
| Mono/Macro | Mono/Macro | HLA-C | -0.4 | | 3.86E-45 | |
| Mono/Macro | Mono/Macro | AKAP9 | -0.5 | | 5.15E-45 | |
| Mono/Macro | Mono/Macro | DNAJB1 | -0.73 | | 5.61E-45 | |
| Mono/Macro | Mono/Macro | TFRC | 0.55 | | 5.77E-45 | |
| Mono/Macro | Mono/Macro | IQGAP1 | 0.4 | | 5.85E-45 | |
| Mono/Macro | Mono/Macro | UBE2D1 | 0.41 | | 1.39E-44 | |
| Mono/Macro | Mono/Macro | NOP58 | -0.52 | | 1.61E-44 | |
| Mono/Macro | Mono/Macro | AMPD2 | 0.26 | | 2.84E-44 | |
| Mono/Macro | Mono/Macro | AC100810.1 | 0.28 | | 4.15E-44 | |
| Mono/Macro | Mono/Macro | TPD52 | -0.43 | | 7.12E-44 | |
| Mono/Macro | Mono/Macro | PDLIM1 | -0.57 | | 7.45E-44 | |
| Mono/Macro | Mono/Macro | C9orf78 | -0.5 | | 9.65E-44 | |
| Mono/Macro | Mono/Macro | PPP1CC | -0.49 | | 9.76E-44 | |
| Mono/Macro | Mono/Macro | PDE4B | 0.34 | | 1.40E-43 | |
| Mono/Macro | Mono/Macro | PHC2 | 0.28 | | 5.40E-43 | |
| Mono/Macro | Mono/Macro | TFDP1 | 0.4 | | 5.70E-43 | |
| Mono/Macro | Mono/Macro | CTSZ | 0.34 | | 7.97E-43 | |
| Mono/Macro | Mono/Macro | COX7C | -0.37 | | 7.97E-43 | |
| Mono/Macro | Mono/Macro | ATP5F1A | -0.52 | | 9.07E-43 | |
| Mono/Macro | Mono/Macro | RGS1 | -1.08 | | 1.50E-42 | |
| Mono/Macro | Mono/Macro | CMPK1 | -0.52 | | 1.60E-42 | |
| Mono/Macro | Mono/Macro | ACAP1 | -0.6 | | 2.56E-42 | |
| Mono/Macro | Mono/Macro | AL121944.1 | -0.59 | | 2.60E-42 | |
| Mono/Macro | Mono/Macro | SLC38A1 | -0.52 | | 2.67E-42 | |
| Mono/Macro | Mono/Macro | ATP5IF1 | -0.55 | | 3.29E-42 | |
| Mono/Macro | Mono/Macro | DDX60L | 0.27 | | 3.51E-42 | |
| Mono/Macro | Mono/Macro | CD52 | -0.84 | | 3.55E-42 | |
| Mono/Macro | Mono/Macro | CD2 | -0.96 | | 3.83E-42 | |
| Mono/Macro | Mono/Macro | RHOH | -0.69 | | 5.51E-42 | |
| Mono/Macro | Mono/Macro | NOP56 | -0.51 | | 5.86E-42 | |
| Mono/Macro | Mono/Macro | EVL | -0.66 | | 1.58E-41 | |
| Mono/Macro | Mono/Macro | LY9 | -0.79 | | 2.13E-41 | |
| Mono/Macro | Mono/Macro | PPP2R5C | -0.65 | | 2.33E-41 | |
| Mono/Macro | Mono/Macro | RPS28 | -0.27 | | 2.50E-41 | |
| Mono/Macro | Mono/Macro | PBXIP1 | -0.58 | | 4.66E-41 | |
| Mono/Macro | Mono/Macro | UBE2R2 | 0.36 | | 4.79E-41 | |
| Mono/Macro | Mono/Macro | GRB2 | 0.35 | | 5.05E-41 | |
| Mono/Macro | Mono/Macro | IFNGR2 | 0.38 | | 5.70E-41 | |
| Mono/Macro | Mono/Macro | RCOR1 | 0.25 | | 6.18E-41 | |
| Mono/Macro | Mono/Macro | ASAP1 | 0.29 | | 6.84E-41 | |
| Mono/Macro | Mono/Macro | TMEM167A | 0.42 | | 7.15E-41 | |
| Mono/Macro | Mono/Macro | TAF1D | -0.5 | | 8.19E-41 | |
| Mono/Macro | Mono/Macro | IFITM1 | -0.72 | | 9.68E-41 | |
| Mono/Macro | Mono/Macro | EFHD2 | 0.35 | | 9.87E-41 | |
| Mono/Macro | Mono/Macro | SDC4 | 0.27 | | 1.20E-40 | |
| Mono/Macro | Mono/Macro | ATP6AP1 | 0.38 | | 1.31E-40 | |
| Mono/Macro | Mono/Macro | FOS | -0.88 | | 1.66E-40 | |
| Mono/Macro | Mono/Macro | SNHG7 | -0.48 | | 2.08E-40 | |
| Mono/Macro | Mono/Macro | NECTIN2 | 0.32 | | 2.20E-40 | |
| Mono/Macro | Mono/Macro | CD53 | 0.34 | | 3.58E-40 | |
| Mono/Macro | Mono/Macro | ANKRD12 | -0.59 | | 4.51E-40 | |
| Mono/Macro | Mono/Macro | RARRES3 | -0.61 | | 7.02E-40 | |
| Mono/Macro | Mono/Macro | TSTD1 | -0.43 | | 1.49E-39 | |
| Mono/Macro | Mono/Macro | IDI1 | -0.56 | | 1.60E-39 | |
| Mono/Macro | Mono/Macro | IL4I1 | 0.26 | | 1.68E-39 | |
| Mono/Macro | Mono/Macro | RPL37A | -0.26 | | 2.24E-39 | |
| Mono/Macro | Mono/Macro | ATOX1 | 0.38 | | 2.42E-39 | |
| Mono/Macro | Mono/Macro | SLC6A6 | 0.28 | | 3.94E-39 | |
| Mono/Macro | Mono/Macro | OFD1 | -0.46 | | 5.77E-39 | |
| Mono/Macro | Mono/Macro | AES | -0.49 | | 7.80E-39 | |
| Mono/Macro | Mono/Macro | ARPC1B | 0.29 | | 9.19E-39 | |
| Mono/Macro | Mono/Macro | FOXP1 | -0.54 | | 9.86E-39 | |
| Mono/Macro | Mono/Macro | CST7 | -1.11 | | 1.12E-38 | |
| Mono/Macro | Mono/Macro | EIF1B | 0.57 | | 1.69E-38 | |
| Mono/Macro | Mono/Macro | CARD19 | 0.33 | | 2.06E-38 | |
| Mono/Macro | Mono/Macro | GCC2 | -0.5 | | 2.45E-38 | |
| Mono/Macro | Mono/Macro | TMEM50A | 0.4 | | 3.66E-38 | |
| Mono/Macro | Mono/Macro | FYN | -0.73 | | 4.84E-38 | |
| Mono/Macro | Mono/Macro | PURB | 0.26 | | 6.46E-38 | |
| Mono/Macro | Mono/Macro | IFRD1 | -0.5 | | 1.08E-37 | |
| Mono/Macro | Mono/Macro | ZNF331 | -0.72 | | 1.55E-37 | |
| Mono/Macro | Mono/Macro | PAXX | -0.48 | | 1.56E-37 | |
| Mono/Macro | Mono/Macro | APOBEC3B | 0.72 | | 1.61E-37 | |
| Mono/Macro | Mono/Macro | CNOT6L | -0.59 | | 1.95E-37 | |
| Mono/Macro | Mono/Macro | ABHD14B | -0.39 | | 2.10E-37 | |
| Mono/Macro | Mono/Macro | KDM6B | 0.33 | | 3.32E-37 | |
| Mono/Macro | Mono/Macro | WDR1 | 0.34 | | 3.38E-37 | |
| Mono/Macro | Mono/Macro | RORA | -0.62 | | 1.16E-36 | |
| Mono/Macro | Mono/Macro | MAP4K4 | 0.29 | | 1.24E-36 | |
| Mono/Macro | Mono/Macro | FBP1 | 0.31 | | 2.08E-36 | |
| Mono/Macro | Mono/Macro | SYNE2 | -0.45 | | 2.33E-36 | |
| Mono/Macro | Mono/Macro | KLF2 | -0.61 | | 5.99E-36 | |
| Mono/Macro | Mono/Macro | PITPNA | 0.26 | | 6.84E-36 | |
| Mono/Macro | Mono/Macro | TOMM7 | -0.39 | | 7.34E-36 | |
| Mono/Macro | Mono/Macro | 1-Sep | -0.49 | | 7.59E-36 | |
| Mono/Macro | Mono/Macro | SNX8 | 0.27 | | 8.39E-36 | |
| Mono/Macro | Mono/Macro | NKTR | -0.47 | | 1.13E-35 | |
| Mono/Macro | Mono/Macro | SEC11C | -0.78 | | 1.13E-35 | |
| Mono/Macro | Mono/Macro | DAZAP2 | 0.3 | | 1.38E-35 | |
| Mono/Macro | Mono/Macro | RPL22 | -0.3 | | 2.09E-35 | |
| Mono/Macro | Mono/Macro | CKLF | 0.43 | | 2.84E-35 | |
| Mono/Macro | Mono/Macro | RPL27 | -0.28 | | 2.94E-35 | |
| Mono/Macro | Mono/Macro | RPL38 | -0.33 | | 3.04E-35 | |
| Mono/Macro | Mono/Macro | EIF3F | -0.42 | | 3.91E-35 | |
| Mono/Macro | Mono/Macro | TNFAIP8 | 0.43 | | 4.79E-35 | |
| Mono/Macro | Mono/Macro | MRPL57 | -0.39 | | 5.11E-35 | |
| Mono/Macro | Mono/Macro | MYL12B | 0.28 | | 5.92E-35 | |
| Mono/Macro | Mono/Macro | IRF1 | -0.55 | | 6.52E-35 | |
| Mono/Macro | Mono/Macro | MS4A1 | -0.75 | | 6.72E-35 | |
| Mono/Macro | Mono/Macro | CCR7 | -0.68 | | 8.30E-35 | |
| Mono/Macro | Mono/Macro | SEC61B | 0.29 | | 1.33E-34 | |
| Mono/Macro | Mono/Macro | RNPS1 | -0.46 | | 1.71E-34 | |
| Mono/Macro | Mono/Macro | ISCU | -0.48 | | 1.79E-34 | |
| Mono/Macro | Mono/Macro | SNRPD2 | -0.41 | | 2.01E-34 | |
| Mono/Macro | Mono/Macro | PNPLA8 | 0.36 | | 2.14E-34 | |
| Mono/Macro | Mono/Macro | RSL1D1 | -0.42 | | 2.31E-34 | |
| Mono/Macro | Mono/Macro | ICAM3 | -0.49 | | 2.60E-34 | |
| Mono/Macro | Mono/Macro | NASP | -0.51 | | 3.14E-34 | |
| Mono/Macro | Mono/Macro | SDSL | 0.27 | | 3.22E-34 | |
| Mono/Macro | Mono/Macro | SLBP | -0.48 | | 3.47E-34 | |
| Mono/Macro | Mono/Macro | GNG7 | -0.45 | | 3.97E-34 | |
| Mono/Macro | Mono/Macro | ADAM9 | 0.3 | | 4.50E-34 | |
| Mono/Macro | Mono/Macro | RHOB | -0.61 | | 4.91E-34 | |
| Mono/Macro | Mono/Macro | PRELID1 | 0.36 | | 4.94E-34 | |
| Mono/Macro | Mono/Macro | TAGAP | -0.57 | | 5.19E-34 | |
| Mono/Macro | Mono/Macro | IDS | -0.56 | | 5.55E-34 | |
| Mono/Macro | Mono/Macro | GNB1 | 0.36 | | 9.00E-34 | |
| Mono/Macro | Mono/Macro | PTPN1 | 0.3 | | 1.35E-33 | |
| Mono/Macro | Mono/Macro | LCK | -0.59 | | 1.37E-33 | |
| Mono/Macro | Mono/Macro | SMDT1 | -0.44 | | 1.84E-33 | |
| Mono/Macro | Mono/Macro | C12orf75 | -0.51 | | 3.58E-33 | |
| Mono/Macro | Mono/Macro | SH2D2A | -0.58 | | 3.96E-33 | |
| Mono/Macro | Mono/Macro | AKIRIN2 | 0.38 | | 4.69E-33 | |
| Mono/Macro | Mono/Macro | STMN1 | -0.6 | | 6.26E-33 | |
| Mono/Macro | Mono/Macro | RPS24 | 0.25 | | 6.77E-33 | |
| Mono/Macro | Mono/Macro | AC114760.2 | -0.52 | | 7.18E-33 | |
| Mono/Macro | Mono/Macro | NDUFS5 | -0.44 | | 8.33E-33 | |
| Mono/Macro | Mono/Macro | RNASE6 | 0.26 | | 8.59E-33 | |
| Mono/Macro | Mono/Macro | MYO9B | 0.26 | | 1.24E-32 | |
| Mono/Macro | Mono/Macro | ETF1 | 0.29 | | 1.35E-32 | |
| Mono/Macro | Mono/Macro | SNHG8 | -0.45 | | 1.42E-32 | |
| Mono/Macro | Mono/Macro | UFC1 | -0.45 | | 1.63E-32 | |
| Mono/Macro | Mono/Macro | MBOAT7 | 0.27 | | 1.82E-32 | |
| Mono/Macro | Mono/Macro | TANK | 0.3 | | 2.16E-32 | |
| Mono/Macro | Mono/Macro | PIK3IP1 | -0.48 | | 2.83E-32 | |
| Mono/Macro | Mono/Macro | IFI27 | -1.02 | | 2.99E-32 | |
| Mono/Macro | Mono/Macro | EEF1D | -0.28 | | 3.07E-32 | |
| Mono/Macro | Mono/Macro | ARHGEF1 | -0.46 | | 3.28E-32 | |
| Mono/Macro | Mono/Macro | MPHOSPH8 | -0.43 | | 3.77E-32 | |
| Mono/Macro | Mono/Macro | ATP5MPL | 0.3 | | 4.34E-32 | |
| Mono/Macro | Mono/Macro | MTPN | 0.33 | | 4.60E-32 | |
| Mono/Macro | Mono/Macro | CCT3 | -0.4 | | 7.90E-32 | |
| Mono/Macro | Mono/Macro | EVI2B | -0.53 | | 9.87E-32 | |
| Mono/Macro | Mono/Macro | FGL2 | 0.29 | | 1.14E-31 | |
| Mono/Macro | Mono/Macro | TNRC6B | -0.43 | | 1.33E-31 | |
| Mono/Macro | Mono/Macro | DCXR | -0.44 | | 1.33E-31 | |
| Mono/Macro | Mono/Macro | PDCD4 | -0.56 | | 1.41E-31 | |
| Mono/Macro | Mono/Macro | EIF3G | -0.39 | | 1.42E-31 | |
| Mono/Macro | Mono/Macro | ARPC4 | 0.31 | | 1.62E-31 | |
| Mono/Macro | Mono/Macro | CD27 | -0.57 | | 1.73E-31 | |
| Mono/Macro | Mono/Macro | ETV3 | 0.25 | | 1.79E-31 | |
| Mono/Macro | Mono/Macro | PARP1 | -0.35 | | 1.95E-31 | |
| Mono/Macro | Mono/Macro | TMEM243 | -0.43 | | 2.25E-31 | |
| Mono/Macro | Mono/Macro | EIF1AX | -0.45 | | 2.41E-31 | |
| Mono/Macro | Mono/Macro | BRK1 | 0.33 | | 2.47E-31 | |
| Mono/Macro | Mono/Macro | PCSK7 | -0.42 | | 2.59E-31 | |
| Mono/Macro | Mono/Macro | CALM2 | -0.39 | | 3.88E-31 | |
| Mono/Macro | Mono/Macro | EIF3D | -0.41 | | 3.98E-31 | |
| Mono/Macro | Mono/Macro | TERF2IP | -0.44 | | 4.33E-31 | |
| Mono/Macro | Mono/Macro | AGR2 | -1.34 | | 4.49E-31 | |
| Mono/Macro | Mono/Macro | CDC42 | 0.28 | | 4.67E-31 | |
| Mono/Macro | Mono/Macro | GMFB | 0.25 | | 4.75E-31 | |
| Mono/Macro | Mono/Macro | ISCA1 | -0.41 | | 5.00E-31 | |
| Mono/Macro | Mono/Macro | GNL3 | -0.36 | | 6.00E-31 | |
| Mono/Macro | Mono/Macro | TSPAN13 | -0.33 | | 7.73E-31 | |
| Mono/Macro | Mono/Macro | SYTL1 | -0.33 | | 7.76E-31 | |
| Mono/Macro | Mono/Macro | P2RY10 | -0.44 | | 7.78E-31 | |
| Mono/Macro | Mono/Macro | PRMT1 | -0.38 | | 9.22E-31 | |
| Mono/Macro | Mono/Macro | IVNS1ABP | 0.35 | | 1.09E-30 | |
| Mono/Macro | Mono/Macro | PPT1 | 0.26 | | 1.66E-30 | |
| Mono/Macro | Mono/Macro | NCOR1 | -0.41 | | 1.89E-30 | |
| Mono/Macro | Mono/Macro | TMED4 | -0.33 | | 2.22E-30 | |
| Mono/Macro | Mono/Macro | SPPL2A | 0.29 | | 4.34E-30 | |
| Mono/Macro | Mono/Macro | FAM177A1 | -0.6 | | 4.85E-30 | |
| Mono/Macro | Mono/Macro | SEL1L3 | -0.32 | | 5.09E-30 | |
| Mono/Macro | Mono/Macro | CARHSP1 | -0.4 | | 5.27E-30 | |
| Mono/Macro | Mono/Macro | HERPUD1 | -1.02 | | 5.39E-30 | |
| Mono/Macro | Mono/Macro | SPCS1 | -0.56 | | 6.50E-30 | |
| Mono/Macro | Mono/Macro | FAM133B | -0.43 | | 6.97E-30 | |
| Mono/Macro | Mono/Macro | CTSC | 0.3 | | 7.65E-30 | |
| Mono/Macro | Mono/Macro | LSP1 | -0.52 | | 8.23E-30 | |
| Mono/Macro | Mono/Macro | PFKFB3 | 0.28 | | 8.45E-30 | |
| Mono/Macro | Mono/Macro | GZMM | -0.61 | | 8.58E-30 | |
| Mono/Macro | Mono/Macro | GZMA | -1.12 | | 8.78E-30 | |
| Mono/Macro | Mono/Macro | PERP | -0.46 | | 9.32E-30 | |
| Mono/Macro | Mono/Macro | IL2RG | -0.47 | | 1.25E-29 | |
| Mono/Macro | Mono/Macro | VPS51 | -0.33 | | 1.34E-29 | |
| Mono/Macro | Mono/Macro | RTF1 | -0.38 | | 1.73E-29 | |
| Mono/Macro | Mono/Macro | 7-Sep | -0.48 | | 1.80E-29 | |
| Mono/Macro | Mono/Macro | DDX18 | -0.42 | | 1.82E-29 | |
| Mono/Macro | Mono/Macro | C4orf48 | 0.32 | | 1.93E-29 | |
| Mono/Macro | Mono/Macro | NUCKS1 | -0.4 | | 2.21E-29 | |
| Mono/Macro | Mono/Macro | HMGN1 | -0.44 | | 2.41E-29 | |
| Mono/Macro | Mono/Macro | BUD23 | -0.35 | | 2.47E-29 | |
| Mono/Macro | Mono/Macro | TTC3 | -0.36 | | 2.52E-29 | |
| Mono/Macro | Mono/Macro | STK24 | 0.3 | | 3.44E-29 | |
| Mono/Macro | Mono/Macro | CSRNP1 | -0.46 | | 4.09E-29 | |
| Mono/Macro | Mono/Macro | TFF1 | -2.05 | | 5.80E-29 | |
| Mono/Macro | Mono/Macro | ODF3B | 0.35 | | 6.00E-29 | |
| Mono/Macro | Mono/Macro | MIF | 0.47 | | 6.10E-29 | |
| Mono/Macro | Mono/Macro | TSTA3 | -0.37 | | 6.37E-29 | |
| Mono/Macro | Mono/Macro | SMCO4 | 0.34 | | 6.57E-29 | |
| Mono/Macro | Mono/Macro | MRPS34 | -0.32 | | 8.15E-29 | |
| Mono/Macro | Mono/Macro | LAMTOR4 | 0.33 | | 8.76E-29 | |
| Mono/Macro | Mono/Macro | TRAF4 | -0.34 | | 1.01E-28 | |
| Mono/Macro | Mono/Macro | SQOR | 0.35 | | 1.10E-28 | |
| Mono/Macro | Mono/Macro | ADIRF | -0.94 | | 1.22E-28 | |
| Mono/Macro | Mono/Macro | CD96 | -0.51 | | 1.24E-28 | |
| Mono/Macro | Mono/Macro | RSRC2 | -0.43 | | 1.28E-28 | |
| Mono/Macro | Mono/Macro | KRT8 | -1.27 | | 1.37E-28 | |
| Mono/Macro | Mono/Macro | KCTD20 | 0.26 | | 1.38E-28 | |
| Mono/Macro | Mono/Macro | PPM1G | -0.35 | | 1.47E-28 | |
| Mono/Macro | Mono/Macro | PNN | -0.38 | | 1.78E-28 | |
| Mono/Macro | Mono/Macro | MAP1LC3B | 0.31 | | 2.74E-28 | |
| Mono/Macro | Mono/Macro | MOB1A | 0.28 | | 3.17E-28 | |
| Mono/Macro | Mono/Macro | PTGES3 | -0.4 | | 3.26E-28 | |
| Mono/Macro | Mono/Macro | RNF126 | -0.31 | | 3.54E-28 | |
| Mono/Macro | Mono/Macro | VMA21 | 0.28 | | 5.18E-28 | |
| Mono/Macro | Mono/Macro | CCL5 | -1.7 | | 7.92E-28 | |
| Mono/Macro | Mono/Macro | ZNF593 | -0.26 | | 8.16E-28 | |
| Mono/Macro | Mono/Macro | KMT2E | -0.45 | | 8.38E-28 | |
| Mono/Macro | Mono/Macro | MDK | -0.49 | | 9.07E-28 | |
| Mono/Macro | Mono/Macro | NFKBIZ | 0.29 | | 9.97E-28 | |
| Mono/Macro | Mono/Macro | BANK1 | -0.51 | | 1.06E-27 | |
| Mono/Macro | Mono/Macro | TRMT112 | -0.37 | | 1.25E-27 | |
| Mono/Macro | Mono/Macro | ELF3 | -0.81 | | 1.29E-27 | |
| Mono/Macro | Mono/Macro | SF3B2 | -0.4 | | 1.34E-27 | |
| Mono/Macro | Mono/Macro | ARGLU1 | -0.43 | | 1.42E-27 | |
| Mono/Macro | Mono/Macro | SIK1 | -0.38 | | 2.12E-27 | |
| Mono/Macro | Mono/Macro | KRT18 | -1.11 | | 2.13E-27 | |
| Mono/Macro | Mono/Macro | UBE2E1 | 0.32 | | 2.50E-27 | |
| Mono/Macro | Mono/Macro | RHOF | -0.41 | | 2.61E-27 | |
| Mono/Macro | Mono/Macro | PTPN22 | -0.47 | | 2.78E-27 | |
| Mono/Macro | Mono/Macro | TCEA1 | -0.39 | | 3.21E-27 | |
| Mono/Macro | Mono/Macro | NOL7 | -0.35 | | 3.47E-27 | |
| Mono/Macro | Mono/Macro | TMED5 | 0.32 | | 3.56E-27 | |
| Mono/Macro | Mono/Macro | AVPI1 | 0.3 | | 4.24E-27 | |
| Mono/Macro | Mono/Macro | CTSW | -0.64 | | 5.15E-27 | |
| Mono/Macro | Mono/Macro | TMEM251 | 0.27 | | 5.60E-27 | |
| Mono/Macro | Mono/Macro | HAX1 | -0.36 | | 5.69E-27 | |
| Mono/Macro | Mono/Macro | LSM7 | -0.41 | | 7.59E-27 | |
| Mono/Macro | Mono/Macro | MAGED2 | -0.32 | | 7.96E-27 | |
| Mono/Macro | Mono/Macro | APEX1 | -0.32 | | 8.74E-27 | |
| Mono/Macro | Mono/Macro | IRAK1 | 0.3 | | 1.05E-26 | |
| Mono/Macro | Mono/Macro | SELENOM | -0.49 | | 1.08E-26 | |
| Mono/Macro | Mono/Macro | CHCHD10 | -0.45 | | 1.17E-26 | |
| Mono/Macro | Mono/Macro | EIF4B | -0.38 | | 1.18E-26 | |
| Mono/Macro | Mono/Macro | GYPC | -0.44 | | 1.22E-26 | |
| Mono/Macro | Mono/Macro | HSPA1B | -0.74 | | 1.26E-26 | |
| Mono/Macro | Mono/Macro | ANAPC5 | -0.3 | | 1.29E-26 | |
| Mono/Macro | Mono/Macro | TMEM134 | -0.31 | | 1.39E-26 | |
| Mono/Macro | Mono/Macro | POLR2K | -0.39 | | 1.70E-26 | |
| Mono/Macro | Mono/Macro | PSIP1 | -0.34 | | 2.10E-26 | |
| Mono/Macro | Mono/Macro | ARHGAP15 | -0.41 | | 2.18E-26 | |
| Mono/Macro | Mono/Macro | MPC2 | -0.35 | | 2.34E-26 | |
| Mono/Macro | Mono/Macro | ARHGAP9 | -0.44 | | 2.65E-26 | |
| Mono/Macro | Mono/Macro | MFSD1 | 0.27 | | 2.94E-26 | |
| Mono/Macro | Mono/Macro | TSPYL2 | -0.48 | | 3.56E-26 | |
| Mono/Macro | Mono/Macro | DNAJC19 | -0.29 | | 4.16E-26 | |
| Mono/Macro | Mono/Macro | BCAP31 | 0.33 | | 4.46E-26 | |
| Mono/Macro | Mono/Macro | APOBEC3G | -0.48 | | 4.56E-26 | |
| Mono/Macro | Mono/Macro | FXYD3 | -0.82 | | 4.57E-26 | |
| Mono/Macro | Mono/Macro | TMED9 | -0.41 | | 4.74E-26 | |
| Mono/Macro | Mono/Macro | KRT19 | -0.92 | | 4.89E-26 | |
| Mono/Macro | Mono/Macro | CD6 | -0.47 | | 4.89E-26 | |
| Mono/Macro | Mono/Macro | SPTBN1 | -0.34 | | 5.10E-26 | |
| Mono/Macro | Mono/Macro | GORASP2 | -0.27 | | 6.19E-26 | |
| Mono/Macro | Mono/Macro | NDUFC1 | -0.35 | | 7.41E-26 | |
| Mono/Macro | Mono/Macro | IMPDH2 | -0.35 | | 7.78E-26 | |
| Mono/Macro | Mono/Macro | FLOT1 | 0.33 | | 9.14E-26 | |
| Mono/Macro | Mono/Macro | LSR | -0.28 | | 9.21E-26 | |
| Mono/Macro | Mono/Macro | REXO2 | -0.3 | | 9.80E-26 | |
| Mono/Macro | Mono/Macro | NDUFA5 | -0.36 | | 1.03E-25 | |
| Mono/Macro | Mono/Macro | S100P | -1.04 | | 1.16E-25 | |
| Mono/Macro | Mono/Macro | C19orf33 | -0.56 | | 1.17E-25 | |
| Mono/Macro | Mono/Macro | NUDC | -0.34 | | 1.29E-25 | |
| Mono/Macro | Mono/Macro | MRPS12 | -0.29 | | 1.29E-25 | |
| Mono/Macro | Mono/Macro | RAB21 | 0.3 | | 1.35E-25 | |
| Mono/Macro | Mono/Macro | ID3 | -0.72 | | 1.38E-25 | |
| Mono/Macro | Mono/Macro | CD3G | -0.49 | | 1.41E-25 | |
| Mono/Macro | Mono/Macro | PSEN1 | 0.27 | | 1.68E-25 | |
| Mono/Macro | Mono/Macro | FCMR | -0.4 | | 1.75E-25 | |
| Mono/Macro | Mono/Macro | DERL3 | -1.07 | | 1.75E-25 | |
| Mono/Macro | Mono/Macro | STK17A | -0.51 | | 1.86E-25 | |
| Mono/Macro | Mono/Macro | LPXN | 0.25 | | 1.99E-25 | |
| Mono/Macro | Mono/Macro | VPREB3 | -0.5 | | 2.12E-25 | |
| Mono/Macro | Mono/Macro | HLA-B | -0.31 | | 2.73E-25 | |
| Mono/Macro | Mono/Macro | SERBP1 | -0.39 | | 3.15E-25 | |
| Mono/Macro | Mono/Macro | LSM2 | -0.31 | | 3.36E-25 | |
| Mono/Macro | Mono/Macro | SLC25A6 | -0.28 | | 3.50E-25 | |
| Mono/Macro | Mono/Macro | SDHA | -0.29 | | 3.67E-25 | |
| Mono/Macro | Mono/Macro | ELF1 | -0.47 | | 4.14E-25 | |
| Mono/Macro | Mono/Macro | CCT2 | -0.35 | | 4.56E-25 | |
| Mono/Macro | Mono/Macro | CD320 | -0.35 | | 4.90E-25 | |
| Mono/Macro | Mono/Macro | MRPS26 | -0.28 | | 5.58E-25 | |
| Mono/Macro | Mono/Macro | ITGB2 | 0.25 | | 7.05E-25 | |
| Mono/Macro | Mono/Macro | G3BP2 | -0.46 | | 7.72E-25 | |
| Mono/Macro | Mono/Macro | MRPL9 | -0.27 | | 8.36E-25 | |
| Mono/Macro | Mono/Macro | CD79B | -0.39 | | 1.01E-24 | |
| Mono/Macro | Mono/Macro | C1QBP | -0.36 | | 1.05E-24 | |
| Mono/Macro | Mono/Macro | HINT2 | -0.26 | | 1.20E-24 | |
| Mono/Macro | Mono/Macro | PIGR | -0.83 | | 1.45E-24 | |
| Mono/Macro | Mono/Macro | ISG15 | 0.98 | | 1.51E-24 | |
| Mono/Macro | Mono/Macro | FAM102A | -0.28 | | 1.58E-24 | |
| Mono/Macro | Mono/Macro | UPF2 | -0.31 | | 1.80E-24 | |
| Mono/Macro | Mono/Macro | ADAM28 | -0.33 | | 2.53E-24 | |
| Mono/Macro | Mono/Macro | CD247 | -0.49 | | 2.69E-24 | |
| Mono/Macro | Mono/Macro | IFI16 | -0.43 | | 3.70E-24 | |
| Mono/Macro | Mono/Macro | CCPG1 | -0.3 | | 3.81E-24 | |
| Mono/Macro | Mono/Macro | MEAF6 | -0.36 | | 3.81E-24 | |
| Mono/Macro | Mono/Macro | RPL7L1 | -0.32 | | 3.96E-24 | |
| Mono/Macro | Mono/Macro | ICAM2 | -0.37 | | 4.01E-24 | |
| Mono/Macro | Mono/Macro | ANP32B | -0.37 | | 4.57E-24 | |
| Mono/Macro | Mono/Macro | EIF2A | -0.29 | | 4.86E-24 | |
| Mono/Macro | Mono/Macro | CHST12 | -0.37 | | 5.69E-24 | |
| Mono/Macro | Mono/Macro | CD8A | -0.73 | | 5.90E-24 | |
| Mono/Macro | Mono/Macro | 6-Sep | -0.44 | | 7.26E-24 | |
| Mono/Macro | Mono/Macro | RWDD1 | -0.37 | | 7.33E-24 | |
| Mono/Macro | Mono/Macro | HNRNPUL1 | -0.39 | | 7.67E-24 | |
| Mono/Macro | Mono/Macro | DSTN | -0.53 | | 7.79E-24 | |
| Mono/Macro | Mono/Macro | NOLC1 | -0.27 | | 8.00E-24 | |
| Mono/Macro | Mono/Macro | MYLIP | -0.41 | | 8.29E-24 | |
| Mono/Macro | Mono/Macro | NSMCE3 | -0.37 | | 9.50E-24 | |
| Mono/Macro | Mono/Macro | DNAAF2 | -0.3 | | 1.10E-23 | |
| Mono/Macro | Mono/Macro | VASP | 0.29 | | 1.35E-23 | |
| Mono/Macro | Mono/Macro | EIF3L | -0.37 | | 1.37E-23 | |
| Mono/Macro | Mono/Macro | HSPB1 | -0.49 | | 1.47E-23 | |
| Mono/Macro | Mono/Macro | NBL1 | -0.32 | | 1.58E-23 | |
| Mono/Macro | Mono/Macro | MAPK6 | 0.27 | | 1.71E-23 | |
| Mono/Macro | Mono/Macro | CLEC2D | -0.4 | | 1.75E-23 | |
| Mono/Macro | Mono/Macro | CHMP2A | 0.29 | | 1.77E-23 | |
| Mono/Macro | Mono/Macro | PPHLN1 | -0.33 | | 1.88E-23 | |
| Mono/Macro | Mono/Macro | SC5D | -0.4 | | 1.98E-23 | |
| Mono/Macro | Mono/Macro | ATP6AP2 | 0.36 | | 2.12E-23 | |
| Mono/Macro | Mono/Macro | RABAC1 | -0.44 | | 2.13E-23 | |
| Mono/Macro | Mono/Macro | DDX27 | -0.33 | | 2.14E-23 | |
| Mono/Macro | Mono/Macro | CD19 | -0.34 | | 2.23E-23 | |
| Mono/Macro | Mono/Macro | CCNG1 | -0.28 | | 2.26E-23 | |
| Mono/Macro | Mono/Macro | DNAJC9 | -0.32 | | 2.54E-23 | |
| Mono/Macro | Mono/Macro | GSPT1 | -0.38 | | 2.63E-23 | |
| Mono/Macro | Mono/Macro | KLRB1 | -1.11 | | 2.81E-23 | |
| Mono/Macro | Mono/Macro | CHPT1 | -0.28 | | 3.10E-23 | |
| Mono/Macro | Mono/Macro | PIM2 | -0.51 | | 3.80E-23 | |
| Mono/Macro | Mono/Macro | AKNA | -0.42 | | 4.02E-23 | |
| Mono/Macro | Mono/Macro | EBP | -0.3 | | 4.02E-23 | |
| Mono/Macro | Mono/Macro | CUTA | -0.41 | | 4.46E-23 | |
| Mono/Macro | Mono/Macro | DHX36 | -0.36 | | 4.54E-23 | |
| Mono/Macro | Mono/Macro | PSMA7 | 0.25 | | 4.74E-23 | |
| Mono/Macro | Mono/Macro | IFT57 | -0.32 | | 5.05E-23 | |
| Mono/Macro | Mono/Macro | ACP1 | -0.32 | | 5.76E-23 | |
| Mono/Macro | Mono/Macro | C16orf54 | -0.41 | | 5.86E-23 | |
| Mono/Macro | Mono/Macro | LGALS3BP | -0.4 | | 7.01E-23 | |
| Mono/Macro | Mono/Macro | TBC1D10C | -0.38 | | 8.03E-23 | |
| Mono/Macro | Mono/Macro | EDF1 | -0.31 | | 8.78E-23 | |
| Mono/Macro | Mono/Macro | ALOX5AP | 0.38 | | 9.60E-23 | |
| Mono/Macro | Mono/Macro | GIMAP7 | -0.48 | | 9.63E-23 | |
| Mono/Macro | Mono/Macro | IMP3 | -0.3 | | 1.06E-22 | |
| Mono/Macro | Mono/Macro | PA2G4 | -0.35 | | 1.08E-22 | |
| Mono/Macro | Mono/Macro | ZBTB20 | -0.29 | | 1.09E-22 | |
| Mono/Macro | Mono/Macro | IL1R2 | 0.36 | | 1.13E-22 | |
| Mono/Macro | Mono/Macro | C19orf53 | -0.34 | | 1.33E-22 | |
| Mono/Macro | Mono/Macro | SRM | -0.28 | | 1.35E-22 | |
| Mono/Macro | Mono/Macro | CD48 | 0.3 | | 1.36E-22 | |
| Mono/Macro | Mono/Macro | SMIM22 | -0.47 | | 1.42E-22 | |
| Mono/Macro | Mono/Macro | MUC1 | -0.6 | | 1.55E-22 | |
| Mono/Macro | Mono/Macro | SPINK1 | -1.11 | | 1.92E-22 | |
| Mono/Macro | Mono/Macro | ATRX | -0.31 | | 1.93E-22 | |
| Mono/Macro | Mono/Macro | STMP1 | 0.28 | | 2.01E-22 | |
| Mono/Macro | Mono/Macro | DNPH1 | -0.31 | | 2.08E-22 | |
| Mono/Macro | Mono/Macro | MZB1 | -1.59 | | 2.11E-22 | |
| Mono/Macro | Mono/Macro | RTRAF | -0.34 | | 2.22E-22 | |
| Mono/Macro | Mono/Macro | BIK | -0.3 | | 2.47E-22 | |
| Mono/Macro | Mono/Macro | LGALS4 | -0.62 | | 3.18E-22 | |
| Mono/Macro | Mono/Macro | F3 | 0.4 | | 3.32E-22 | |
| Mono/Macro | Mono/Macro | GZMK | -1.06 | | 3.86E-22 | |
| Mono/Macro | Mono/Macro | SPSB3 | -0.29 | | 4.63E-22 | |
| Mono/Macro | Mono/Macro | VPS37B | -0.44 | | 4.63E-22 | |
| Mono/Macro | Mono/Macro | MRPS7 | -0.31 | | 4.81E-22 | |
| Mono/Macro | Mono/Macro | ENSA | -0.37 | | 4.91E-22 | |
| Mono/Macro | Mono/Macro | CDK2AP2 | -0.33 | | 4.99E-22 | |
| Mono/Macro | Mono/Macro | SS18L2 | -0.36 | | 5.68E-22 | |
| Mono/Macro | Mono/Macro | NEU1 | -0.42 | | 5.91E-22 | |
| Mono/Macro | Mono/Macro | GMDS | -0.3 | | 6.30E-22 | |
| Mono/Macro | Mono/Macro | SRPRA | -0.29 | | 6.84E-22 | |
| Mono/Macro | Mono/Macro | FAM118A | -0.32 | | 6.98E-22 | |
| Mono/Macro | Mono/Macro | FNBP4 | -0.3 | | 7.14E-22 | |
| Mono/Macro | Mono/Macro | NAP1L4 | -0.29 | | 8.35E-22 | |
| Mono/Macro | Mono/Macro | APMAP | -0.34 | | 9.66E-22 | |
| Mono/Macro | Mono/Macro | VSIG2 | -0.38 | | 9.81E-22 | |
| Mono/Macro | Mono/Macro | ILF2 | -0.35 | | 1.06E-21 | |
| Mono/Macro | Mono/Macro | NR3C1 | -0.53 | | 1.08E-21 | |
| Mono/Macro | Mono/Macro | GOLGA4 | -0.29 | | 1.12E-21 | |
| Mono/Macro | Mono/Macro | RAB30 | -0.34 | | 1.38E-21 | |
| Mono/Macro | Mono/Macro | ICOS | -0.51 | | 1.40E-21 | |
| Mono/Macro | Mono/Macro | ANKRD36 | -0.27 | | 1.50E-21 | |
| Mono/Macro | Mono/Macro | C1orf122 | 0.36 | | 1.53E-21 | |
| Mono/Macro | Mono/Macro | ZC3HAV1 | -0.37 | | 1.78E-21 | |
| Mono/Macro | Mono/Macro | S100A13 | -0.32 | | 1.86E-21 | |
| Mono/Macro | Mono/Macro | PRPF38B | -0.37 | | 1.94E-21 | |
| Mono/Macro | Mono/Macro | MRPL32 | -0.27 | | 1.96E-21 | |
| Mono/Macro | Mono/Macro | ECH1 | -0.31 | | 1.97E-21 | |
| Mono/Macro | Mono/Macro | RNF125 | -0.37 | | 2.69E-21 | |
| Mono/Macro | Mono/Macro | ANXA10 | -0.53 | | 2.74E-21 | |
| Mono/Macro | Mono/Macro | XIST | -0.4 | | 3.19E-21 | |
| Mono/Macro | Mono/Macro | ZNF165 | -0.27 | | 3.36E-21 | |
| Mono/Macro | Mono/Macro | NT5C3A | -0.25 | | 3.37E-21 | |
| Mono/Macro | Mono/Macro | PPIG | -0.36 | | 3.48E-21 | |
| Mono/Macro | Mono/Macro | NR4A1 | -0.56 | | 3.68E-21 | |
| Mono/Macro | Mono/Macro | YWHAQ | -0.38 | | 4.06E-21 | |
| Mono/Macro | Mono/Macro | HLA-A | -0.33 | | 4.08E-21 | |
| Mono/Macro | Mono/Macro | MRPL4 | -0.26 | | 4.21E-21 | |
| Mono/Macro | Mono/Macro | ODF2L | -0.36 | | 5.21E-21 | |
| Mono/Macro | Mono/Macro | JTB | -0.33 | | 5.21E-21 | |
| Mono/Macro | Mono/Macro | ATRAID | -0.31 | | 5.61E-21 | |
| Mono/Macro | Mono/Macro | SRRM1 | -0.37 | | 6.44E-21 | |
| Mono/Macro | Mono/Macro | LSM5 | -0.32 | | 6.94E-21 | |
| Mono/Macro | Mono/Macro | TSPAN8 | -1.14 | | 7.40E-21 | |
| Mono/Macro | Mono/Macro | C7orf50 | -0.34 | | 7.86E-21 | |
| Mono/Macro | Mono/Macro | PSCA | -0.94 | | 8.00E-21 | |
| Mono/Macro | Mono/Macro | LINC00513 | -0.34 | | 8.28E-21 | |
| Mono/Macro | Mono/Macro | PWP1 | -0.26 | | 8.59E-21 | |
| Mono/Macro | Mono/Macro | CD9 | 0.42 | | 9.77E-21 | |
| Mono/Macro | Mono/Macro | EML4 | -0.45 | | 1.04E-20 | |
| Mono/Macro | Mono/Macro | PLA2G16 | -0.28 | | 1.07E-20 | |
| Mono/Macro | Mono/Macro | TFF2 | -1.19 | | 1.19E-20 | |
| Mono/Macro | Mono/Macro | NFE2L2 | 0.26 | | 1.49E-20 | |
| Mono/Macro | Mono/Macro | IK | -0.33 | | 1.83E-20 | |
| Mono/Macro | Mono/Macro | DUT | -0.36 | | 1.90E-20 | |
| Mono/Macro | Mono/Macro | BCAS2 | -0.37 | | 1.94E-20 | |
| Mono/Macro | Mono/Macro | EPB41L4A-AS1 | -0.29 | | 2.11E-20 | |
| Mono/Macro | Mono/Macro | SON | -0.38 | | 2.27E-20 | |
| Mono/Macro | Mono/Macro | SRSF11 | -0.32 | | 2.60E-20 | |
| Mono/Macro | Mono/Macro | POLR2I | -0.28 | | 2.75E-20 | |
| Mono/Macro | Mono/Macro | LINC01871 | -0.53 | | 3.16E-20 | |
| Mono/Macro | Mono/Macro | TBCC | -0.32 | | 3.32E-20 | |
| Mono/Macro | Mono/Macro | NXT1 | -0.33 | | 3.43E-20 | |
| Mono/Macro | Mono/Macro | RPS26 | -0.37 | | 3.51E-20 | |
| Mono/Macro | Mono/Macro | LBH | -0.35 | | 3.79E-20 | |
| Mono/Macro | Mono/Macro | VAMP3 | 0.25 | | 4.27E-20 | |
| Mono/Macro | Mono/Macro | SMARCB1 | -0.26 | | 4.37E-20 | |
| Mono/Macro | Mono/Macro | RBM38 | -0.31 | | 4.39E-20 | |
| Mono/Macro | Mono/Macro | P2RY8 | -0.32 | | 4.57E-20 | |
| Mono/Macro | Mono/Macro | CLDN18 | -0.41 | | 4.70E-20 | |
| Mono/Macro | Mono/Macro | GADD45A | -0.47 | | 5.31E-20 | |
| Mono/Macro | Mono/Macro | S100A14 | -0.46 | | 5.71E-20 | |
| Mono/Macro | Mono/Macro | TPR | -0.32 | | 6.08E-20 | |
| Mono/Macro | Mono/Macro | POLR3GL | -0.25 | | 7.22E-20 | |
| Mono/Macro | Mono/Macro | ANXA6 | -0.35 | | 7.27E-20 | |
| Mono/Macro | Mono/Macro | ITGB7 | -0.3 | | 7.27E-20 | |
| Mono/Macro | Mono/Macro | C9orf16 | -0.35 | | 7.72E-20 | |
| Mono/Macro | Mono/Macro | TUFM | -0.32 | | 7.78E-20 | |
| Mono/Macro | Mono/Macro | TMEM123 | -0.4 | | 7.81E-20 | |
| Mono/Macro | Mono/Macro | PGD | 0.29 | | 8.29E-20 | |
| Mono/Macro | Mono/Macro | SNRNP70 | -0.27 | | 8.94E-20 | |
| Mono/Macro | Mono/Macro | TRAT1 | -0.43 | | 9.23E-20 | |
| Mono/Macro | Mono/Macro | RSBN1L | -0.26 | | 9.30E-20 | |
| Mono/Macro | Mono/Macro | CLU | -0.6 | | 9.55E-20 | |
| Mono/Macro | Mono/Macro | THRAP3 | -0.29 | | 1.06E-19 | |
| Mono/Macro | Mono/Macro | FUS | -0.39 | | 1.32E-19 | |
| Mono/Macro | Mono/Macro | ARPC5L | -0.29 | | 1.38E-19 | |
| Mono/Macro | Mono/Macro | SELENOP | -0.44 | | 1.47E-19 | |
| Mono/Macro | Mono/Macro | NPDC1 | -0.33 | | 1.86E-19 | |
| Mono/Macro | Mono/Macro | TPM1 | -0.34 | | 1.87E-19 | |
| Mono/Macro | Mono/Macro | GTF3A | -0.32 | | 1.89E-19 | |
| Mono/Macro | Mono/Macro | NDUFAF8 | -0.26 | | 2.07E-19 | |
| Mono/Macro | Mono/Macro | S100A16 | -0.33 | | 2.19E-19 | |
| Mono/Macro | Mono/Macro | PIGT | -0.27 | | 2.27E-19 | |
| Mono/Macro | Mono/Macro | ARL2BP | -0.29 | | 2.32E-19 | |
| Mono/Macro | Mono/Macro | ERGIC2 | -0.26 | | 2.46E-19 | |
| Mono/Macro | Mono/Macro | UBE2S | -0.34 | | 2.65E-19 | |
| Mono/Macro | Mono/Macro | FBL | -0.33 | | 2.77E-19 | |
| Mono/Macro | Mono/Macro | CCT7 | -0.3 | | 3.26E-19 | |
| Mono/Macro | Mono/Macro | HIST1H2BK | -0.48 | | 3.34E-19 | |
| Mono/Macro | Mono/Macro | CEBPZ | -0.28 | | 3.80E-19 | |
| Mono/Macro | Mono/Macro | SARS | -0.26 | | 5.62E-19 | |
| Mono/Macro | Mono/Macro | ETS1 | -0.36 | | 6.36E-19 | |
| Mono/Macro | Mono/Macro | NR4A2 | -0.6 | | 6.64E-19 | |
| Mono/Macro | Mono/Macro | NCOA7 | -0.3 | | 6.80E-19 | |
| Mono/Macro | Mono/Macro | PGC | -2.01 | | 7.54E-19 | |
| Mono/Macro | Mono/Macro | SELENOW | -0.37 | | 7.98E-19 | |
| Mono/Macro | Mono/Macro | WIPF1 | -0.42 | | 7.98E-19 | |
| Mono/Macro | Mono/Macro | CIR1 | -0.27 | | 8.66E-19 | |
| Mono/Macro | Mono/Macro | ID1 | -0.53 | | 8.85E-19 | |
| Mono/Macro | Mono/Macro | H2AFX | -0.29 | | 1.10E-18 | |
| Mono/Macro | Mono/Macro | NAP1L1 | -0.35 | | 1.19E-18 | |
| Mono/Macro | Mono/Macro | PRPS1 | -0.27 | | 1.24E-18 | |
| Mono/Macro | Mono/Macro | GPX2 | -0.37 | | 1.33E-18 | |
| Mono/Macro | Mono/Macro | TSEN54 | -0.26 | | 1.67E-18 | |
| Mono/Macro | Mono/Macro | AC044849.1 | -0.31 | | 1.92E-18 | |
| Mono/Macro | Mono/Macro | MT-ND1 | -0.27 | | 2.06E-18 | |
| Mono/Macro | Mono/Macro | UBE2I | -0.31 | | 2.42E-18 | |
| Mono/Macro | Mono/Macro | ANKRD36C | -0.3 | | 3.12E-18 | |
| Mono/Macro | Mono/Macro | IL2RB | -0.41 | | 3.24E-18 | |
| Mono/Macro | Mono/Macro | OGA | -0.29 | | 3.26E-18 | |
| Mono/Macro | Mono/Macro | CPNE1 | -0.26 | | 3.29E-18 | |
| Mono/Macro | Mono/Macro | HEXB | 0.26 | | 3.65E-18 | |
| Mono/Macro | Mono/Macro | PRF1 | -0.61 | | 3.74E-18 | |
| Mono/Macro | Mono/Macro | CLTB | -0.31 | | 4.32E-18 | |
| Mono/Macro | Mono/Macro | STUB1 | -0.26 | | 4.65E-18 | |
| Mono/Macro | Mono/Macro | IRF7 | 0.25 | | 5.25E-18 | |
| Mono/Macro | Mono/Macro | AQP3 | -0.28 | | 5.34E-18 | |
| Mono/Macro | Mono/Macro | TOMM22 | -0.28 | | 5.78E-18 | |
| Mono/Macro | Mono/Macro | GZMH | -0.76 | | 6.35E-18 | |
| Mono/Macro | Mono/Macro | EAPP | -0.27 | | 7.44E-18 | |
| Mono/Macro | Mono/Macro | EID1 | -0.38 | | 8.08E-18 | |
| Mono/Macro | Mono/Macro | GSTK1 | -0.32 | | 8.57E-18 | |
| Mono/Macro | Mono/Macro | CORO1A | -0.52 | | 1.05E-17 | |
| Mono/Macro | Mono/Macro | SRSF3 | -0.32 | | 1.17E-17 | |
| Mono/Macro | Mono/Macro | LDLRAD4 | -0.57 | | 1.18E-17 | |
| Mono/Macro | Mono/Macro | ATF6B | -0.28 | | 1.19E-17 | |
| Mono/Macro | Mono/Macro | NARF | -0.26 | | 1.28E-17 | |
| Mono/Macro | Mono/Macro | NUCB2 | -0.35 | | 1.31E-17 | |
| Mono/Macro | Mono/Macro | OXA1L | -0.26 | | 1.47E-17 | |
| Mono/Macro | Mono/Macro | NQO1 | -0.35 | | 1.55E-17 | |
| Mono/Macro | Mono/Macro | CXCR3 | -0.4 | | 1.61E-17 | |
| Mono/Macro | Mono/Macro | SP100 | -0.31 | | 1.76E-17 | |
| Mono/Macro | Mono/Macro | EPCAM | -0.44 | | 1.93E-17 | |
| Mono/Macro | Mono/Macro | CLNS1A | -0.26 | | 1.93E-17 | |
| Mono/Macro | Mono/Macro | PRRC2C | -0.34 | | 2.54E-17 | |
| Mono/Macro | Mono/Macro | AMD1 | -0.37 | | 3.33E-17 | |
| Mono/Macro | Mono/Macro | STK17B | -0.41 | | 3.84E-17 | |
| Mono/Macro | Mono/Macro | SOCS1 | -0.29 | | 4.12E-17 | |
| Mono/Macro | Mono/Macro | ATP5F1B | -0.32 | | 4.35E-17 | |
| Mono/Macro | Mono/Macro | APRT | -0.3 | | 4.51E-17 | |
| Mono/Macro | Mono/Macro | NENF | -0.25 | | 4.58E-17 | |
| Mono/Macro | Mono/Macro | BUB3 | -0.3 | | 5.04E-17 | |
| Mono/Macro | Mono/Macro | ADAR | -0.3 | | 5.21E-17 | |
| Mono/Macro | Mono/Macro | SAFB2 | -0.29 | | 5.39E-17 | |
| Mono/Macro | Mono/Macro | 9-Sep | -0.32 | | 5.92E-17 | |
| Mono/Macro | Mono/Macro | RNF19A | -0.33 | | 6.13E-17 | |
| Mono/Macro | Mono/Macro | KLF3 | -0.31 | | 6.15E-17 | |
| Mono/Macro | Mono/Macro | COX20 | -0.25 | | 6.42E-17 | |
| Mono/Macro | Mono/Macro | CCR6 | -0.33 | | 6.59E-17 | |
| Mono/Macro | Mono/Macro | RBM4 | -0.28 | | 6.69E-17 | |
| Mono/Macro | Mono/Macro | SNAPC1 | 0.29 | | 7.87E-17 | |
| Mono/Macro | Mono/Macro | GADD45GIP1 | -0.28 | | 8.21E-17 | |
| Mono/Macro | Mono/Macro | TCF7 | -0.28 | | 8.82E-17 | |
| Mono/Macro | Mono/Macro | EMC4 | -0.26 | | 9.13E-17 | |
| Mono/Macro | Mono/Macro | ARFGAP2 | -0.26 | | 9.15E-17 | |
| Mono/Macro | Mono/Macro | IL27RA | -0.27 | | 1.02E-16 | |
| Mono/Macro | Mono/Macro | BCLAF1 | -0.32 | | 1.12E-16 | |
| Mono/Macro | Mono/Macro | TIGIT | -0.51 | | 1.17E-16 | |
| Mono/Macro | Mono/Macro | DYNLT1 | 0.27 | | 1.17E-16 | |
| Mono/Macro | Mono/Macro | CDKN1B | -0.28 | | 1.33E-16 | |
| Mono/Macro | Mono/Macro | PDXK | 0.28 | | 1.37E-16 | |
| Mono/Macro | Mono/Macro | BSG | -0.34 | | 1.48E-16 | |
| Mono/Macro | Mono/Macro | BIRC3 | -0.45 | | 1.74E-16 | |
| Mono/Macro | Mono/Macro | YWHAH | -0.36 | | 1.85E-16 | |
| Mono/Macro | Mono/Macro | LAG3 | -0.44 | | 2.33E-16 | |
| Mono/Macro | Mono/Macro | GNAS | -0.32 | | 2.37E-16 | |
| Mono/Macro | Mono/Macro | MRPL41 | -0.29 | | 2.85E-16 | |
| Mono/Macro | Mono/Macro | CTNNBL1 | 0.25 | | 4.04E-16 | |
| Mono/Macro | Mono/Macro | CASP4 | 0.27 | | 4.54E-16 | |
| Mono/Macro | Mono/Macro | PPP2R1A | -0.26 | | 4.93E-16 | |
| Mono/Macro | Mono/Macro | FABP5 | 0.56 | | 6.54E-16 | |
| Mono/Macro | Mono/Macro | ANP32E | -0.26 | | 6.89E-16 | |
| Mono/Macro | Mono/Macro | SKAP1 | -0.27 | | 7.52E-16 | |
| Mono/Macro | Mono/Macro | MAF1 | -0.29 | | 8.70E-16 | |
| Mono/Macro | Mono/Macro | RALGAPA1 | -0.31 | | 9.77E-16 | |
| Mono/Macro | Mono/Macro | PPM1K | -0.31 | | 1.08E-15 | |
| Mono/Macro | Mono/Macro | SMCHD1 | -0.33 | | 1.22E-15 | |
| Mono/Macro | Mono/Macro | ARL6IP4 | -0.29 | | 1.24E-15 | |
| Mono/Macro | Mono/Macro | HNRNPR | -0.27 | | 1.27E-15 | |
| Mono/Macro | Mono/Macro | RNF138 | -0.26 | | 1.60E-15 | |
| Mono/Macro | Mono/Macro | MORF4L2 | -0.29 | | 1.64E-15 | |
| Mono/Macro | Mono/Macro | ALKBH5 | -0.26 | | 1.72E-15 | |
| Mono/Macro | Mono/Macro | XRCC6 | -0.3 | | 1.80E-15 | |
| Mono/Macro | Mono/Macro | TRAM1 | -0.35 | | 2.25E-15 | |
| Mono/Macro | Mono/Macro | PYCARD | 0.26 | | 2.39E-15 | |
| Mono/Macro | Mono/Macro | JSRP1 | -0.42 | | 2.55E-15 | |
| Mono/Macro | Mono/Macro | BBX | -0.26 | | 2.63E-15 | |
| Mono/Macro | Mono/Macro | FNBP1 | -0.35 | | 2.82E-15 | |
| Mono/Macro | Mono/Macro | PHF1 | -0.3 | | 2.83E-15 | |
| Mono/Macro | Mono/Macro | HES1 | -0.66 | | 3.18E-15 | |
| Mono/Macro | Mono/Macro | TPM2 | -0.41 | | 3.19E-15 | |
| Mono/Macro | Mono/Macro | RCAN3 | -0.26 | | 4.08E-15 | |
| Mono/Macro | Mono/Macro | TOMM5 | -0.26 | | 4.51E-15 | |
| Mono/Macro | Mono/Macro | HMGN2 | -0.37 | | 4.95E-15 | |
| Mono/Macro | Mono/Macro | HIST1H1C | -0.39 | | 5.01E-15 | |
| Mono/Macro | Mono/Macro | LMAN1 | -0.28 | | 5.14E-15 | |
| Mono/Macro | Mono/Macro | PITHD1 | -0.28 | | 5.32E-15 | |
| Mono/Macro | Mono/Macro | KPNA2 | -0.31 | | 5.58E-15 | |
| Mono/Macro | Mono/Macro | SOX4 | -0.38 | | 6.75E-15 | |
| Mono/Macro | Mono/Macro | FGFR1OP2 | -0.26 | | 8.43E-15 | |
| Mono/Macro | Mono/Macro | CHD2 | -0.31 | | 1.08E-14 | |
| Mono/Macro | Mono/Macro | TMEM230 | -0.26 | | 1.13E-14 | |
| Mono/Macro | Mono/Macro | SDF2L1 | 0.29 | | 1.14E-14 | |
| Mono/Macro | Mono/Macro | PLPP5 | -0.26 | | 1.21E-14 | |
| Mono/Macro | Mono/Macro | HOPX | -0.43 | | 1.45E-14 | |
| Mono/Macro | Mono/Macro | RAN | -0.32 | | 1.49E-14 | |
| Mono/Macro | Mono/Macro | RBM23 | -0.3 | | 1.90E-14 | |
| Mono/Macro | Mono/Macro | UBXN1 | -0.27 | | 2.13E-14 | |
| Mono/Macro | Mono/Macro | LMNA | -0.47 | | 2.13E-14 | |
| Mono/Macro | Mono/Macro | CLDN4 | -0.52 | | 2.30E-14 | |
| Mono/Macro | Mono/Macro | TOB1 | -0.32 | | 2.41E-14 | |
| Mono/Macro | Mono/Macro | AHI1 | -0.25 | | 2.57E-14 | |
| Mono/Macro | Mono/Macro | RF00598 | -0.27 | | 2.73E-14 | |
| Mono/Macro | Mono/Macro | CBLB | -0.25 | | 3.44E-14 | |
| Mono/Macro | Mono/Macro | USP11 | -0.26 | | 3.52E-14 | |
| Mono/Macro | Mono/Macro | APOBEC3C | -0.27 | | 3.75E-14 | |
| Mono/Macro | Mono/Macro | HMGN3 | -0.27 | | 3.84E-14 | |
| Mono/Macro | Mono/Macro | CMC1 | -0.38 | | 4.49E-14 | |
| Mono/Macro | Mono/Macro | METTL9 | 0.26 | | 4.71E-14 | |
| Mono/Macro | Mono/Macro | RPA3 | -0.26 | | 5.67E-14 | |
| Mono/Macro | Mono/Macro | SYNGR2 | -0.3 | | 6.51E-14 | |
| Mono/Macro | Mono/Macro | ARID4B | -0.35 | | 6.58E-14 | |
| Mono/Macro | Mono/Macro | KRTCAP2 | -0.34 | | 6.66E-14 | |
| Mono/Macro | Mono/Macro | WBP11 | -0.27 | | 6.79E-14 | |
| Mono/Macro | Mono/Macro | SMAP2 | -0.29 | | 8.40E-14 | |
| Mono/Macro | Mono/Macro | SRI | -0.28 | | 8.76E-14 | |
| Mono/Macro | Mono/Macro | S1PR4 | -0.3 | | 9.63E-14 | |
| Mono/Macro | Mono/Macro | ARF6 | -0.33 | | 9.83E-14 | |
| Mono/Macro | Mono/Macro | ARHGAP45 | -0.25 | | 9.93E-14 | |
| Mono/Macro | Mono/Macro | ADGRE5 | -0.39 | | 1.14E-13 | |
| Mono/Macro | Mono/Macro | SCAF11 | -0.3 | | 1.28E-13 | |
| Mono/Macro | Mono/Macro | VOPP1 | -0.26 | | 1.37E-13 | |
| Mono/Macro | Mono/Macro | DDX6 | -0.26 | | 1.41E-13 | |
| Mono/Macro | Mono/Macro | HERPUD2 | -0.27 | | 1.53E-13 | |
| Mono/Macro | Mono/Macro | HMGB2 | -0.42 | | 1.55E-13 | |
| Mono/Macro | Mono/Macro | SRRM2 | -0.28 | | 1.63E-13 | |
| Mono/Macro | Mono/Macro | GZMB | -1.19 | | 1.85E-13 | |
| Mono/Macro | Mono/Macro | CFAP20 | -0.26 | | 1.88E-13 | |
| Mono/Macro | Mono/Macro | PIK3R1 | -0.38 | | 2.15E-13 | |
| Mono/Macro | Mono/Macro | RPL22L1 | -0.3 | | 2.25E-13 | |
| Mono/Macro | Mono/Macro | ARRDC2 | -0.25 | | 2.29E-13 | |
| Mono/Macro | Mono/Macro | CCND3 | -0.27 | | 2.95E-13 | |
| Mono/Macro | Mono/Macro | LCN2 | -0.74 | | 3.29E-13 | |
| Mono/Macro | Mono/Macro | TSPYL1 | -0.28 | | 4.20E-13 | |
| Mono/Macro | Mono/Macro | EGR1 | -0.67 | | 4.26E-13 | |
| Mono/Macro | Mono/Macro | SCP2 | -0.28 | | 4.66E-13 | |
| Mono/Macro | Mono/Macro | SNRPD1 | -0.25 | | 5.09E-13 | |
| Mono/Macro | Mono/Macro | TRABD | -0.26 | | 5.43E-13 | |
| Mono/Macro | Mono/Macro | SELENOT | -0.29 | | 5.59E-13 | |
| Mono/Macro | Mono/Macro | RBM25 | -0.29 | | 8.10E-13 | |
| Mono/Macro | Mono/Macro | LYST | -0.31 | | 8.28E-13 | |
| Mono/Macro | Mono/Macro | GBP1 | 0.25 | | 8.30E-13 | |
| Mono/Macro | Mono/Macro | DDIT4 | -0.48 | | 9.59E-13 | |
| Mono/Macro | Mono/Macro | TXNDC15 | -0.26 | | 1.32E-12 | |
| Mono/Macro | Mono/Macro | NDUFB7 | -0.29 | | 1.33E-12 | |
| Mono/Macro | Mono/Macro | IGFBP4 | -0.54 | | 1.48E-12 | |
| Mono/Macro | Mono/Macro | ARPP19 | -0.26 | | 1.58E-12 | |
| Mono/Macro | Mono/Macro | MBP | -0.27 | | 1.59E-12 | |
| Mono/Macro | Mono/Macro | CCNDBP1 | -0.25 | | 1.61E-12 | |
| Mono/Macro | Mono/Macro | PSME2 | 0.3 | | 1.63E-12 | |
| Mono/Macro | Mono/Macro | SMIM14 | -0.32 | | 1.90E-12 | |
| Mono/Macro | Mono/Macro | EIF4H | -0.25 | | 1.95E-12 | |
| Mono/Macro | Mono/Macro | IDH2 | -0.27 | | 2.07E-12 | |
| Mono/Macro | Mono/Macro | CLK1 | -0.28 | | 2.17E-12 | |
| Mono/Macro | Mono/Macro | IGLL5 | -2.57 | | 2.20E-12 | |
| Mono/Macro | Mono/Macro | LYAR | -0.31 | | 2.52E-12 | |
| Mono/Macro | Mono/Macro | MYC | -0.4 | | 2.68E-12 | |
| Mono/Macro | Mono/Macro | SF3B1 | -0.28 | | 3.42E-12 | |
| Mono/Macro | Mono/Macro | CYB5A | -0.25 | | 4.38E-12 | |
| Mono/Macro | Mono/Macro | DUSP2 | -0.54 | | 4.47E-12 | |
| Mono/Macro | Mono/Macro | MEF2C | -0.33 | | 5.34E-12 | |
| Mono/Macro | Mono/Macro | JPT1 | -0.33 | | 5.36E-12 | |
| Mono/Macro | Mono/Macro | MGAT4A | -0.33 | | 6.60E-12 | |
| Mono/Macro | Mono/Macro | ATP5MC1 | -0.27 | | 7.80E-12 | |
| Mono/Macro | Mono/Macro | TNFRSF25 | -0.28 | | 8.16E-12 | |
| Mono/Macro | Mono/Macro | PSMB8 | -0.25 | | 1.52E-11 | |
| Mono/Macro | Mono/Macro | MT-ND5 | -0.56 | | 1.56E-11 | |
| Mono/Macro | Mono/Macro | PCNA | -0.25 | | 2.21E-11 | |
| Mono/Macro | Mono/Macro | PRDX5 | -0.3 | | 2.49E-11 | |
| Mono/Macro | Mono/Macro | KLF6 | -0.38 | | 3.39E-11 | |
| Mono/Macro | Mono/Macro | PPA1 | -0.29 | | 3.78E-11 | |
| Mono/Macro | Mono/Macro | CCNL1 | -0.3 | | 4.14E-11 | |
| Mono/Macro | Mono/Macro | TMED2 | -0.25 | | 6.10E-11 | |
| Mono/Macro | Mono/Macro | CRELD2 | -0.26 | | 1.19E-10 | |
| Mono/Macro | Mono/Macro | LAT | -0.31 | | 1.30E-10 | |
| Mono/Macro | Mono/Macro | PIM1 | -0.34 | | 1.47E-10 | |
| Mono/Macro | Mono/Macro | SYTL3 | -0.33 | | 1.53E-10 | |
| Mono/Macro | Mono/Macro | SET | -0.26 | | 1.91E-10 | |
| Mono/Macro | Mono/Macro | ZFP36L1 | -0.3 | | 3.47E-10 | |
| Mono/Macro | Mono/Macro | BRD2 | -0.27 | | 4.35E-10 | |
| Mono/Macro | Mono/Macro | EMB | -0.27 | | 4.81E-10 | |
| Mono/Macro | Mono/Macro | DNAJB9 | -0.39 | | 4.98E-10 | |
| Mono/Macro | Mono/Macro | MTRNR2L2 | -0.61 | | 1.53E-09 | |
| Mono/Macro | Mono/Macro | PTPRC | -0.45 | | 1.75E-09 | |
| Mono/Macro | Mono/Macro | IFITM2 | -0.38 | | 1.86E-09 | |
| Mono/Macro | Mono/Macro | WSB1 | -0.27 | | 1.94E-09 | |
| Mono/Macro | Mono/Macro | CD74 | -0.41 | | 2.19E-09 | |
| Mono/Macro | Mono/Macro | GLUD1 | -0.25 | | 2.91E-09 | |
| Mono/Macro | Mono/Macro | CYC1 | -0.26 | | 3.03E-09 | |
| Mono/Macro | Mono/Macro | ALG13 | -0.26 | | 1.17E-08 | |
| Mono/Macro | Mono/Macro | MT1F | 0.37 | | 1.23E-08 | |
| Mono/Macro | Mono/Macro | DDX5 | -0.26 | | 1.81E-08 | |
| Mono/Macro | Mono/Macro | EVI2A | -0.26 | | 2.79E-08 | |
| Mono/Macro | Mono/Macro | AC020916.1 | -0.35 | | 3.23E-08 | |
| Mono/Macro | Mono/Macro | STAT4 | -0.3 | | 3.64E-08 | |
| Mono/Macro | Mono/Macro | SPINT2 | -0.29 | | 5.25E-08 | |
| Mono/Macro | Mono/Macro | TMEM59 | -0.34 | | 5.66E-08 | |
| Mono/Macro | Mono/Macro | GSN | -0.32 | | 6.93E-08 | |
| Mono/Macro | Mono/Macro | PRDX4 | -0.56 | | 7.39E-08 | |
| Mono/Macro | Mono/Macro | UCP2 | -0.34 | | 1.01E-07 | |
| Mono/Macro | Mono/Macro | SSR4 | -1.13 | | 1.39E-07 | |
| Mono/Macro | Mono/Macro | PTPN7 | -0.27 | | 6.72E-07 | |
| Mono/Macro | Mono/Macro | GNG2 | -0.26 | | 7.63E-07 | |
| Mono/Macro | Mono/Macro | WDR74 | -0.25 | | 9.19E-07 | |
| Mono/Macro | Mono/Macro | TCF4 | -0.25 | | 9.42E-07 | |
| Mono/Macro | Mono/Macro | TAF7 | -0.26 | | 9.60E-07 | |
| Mono/Macro | Mono/Macro | FOSB | -0.45 | | 1.86E-06 | |
| Mono/Macro | Mono/Macro | CRIP1 | -0.41 | | 3.02E-06 | |
| Mono/Macro | Mono/Macro | CA2 | -0.44 | | 8.25E-06 | |
| B | B | MT-CO3 | 0.63 | | 2.14E-157 | |
| B | B | MT-ND4 | 0.62 | | 3.39E-148 | |
| B | B | MT-ND3 | 0.66 | | 3.17E-141 | |
| B | B | MT-CYB | 0.62 | | 4.66E-140 | |
| B | B | MT-CO1 | 0.61 | | 3.22E-136 | |
| B | B | MT-CO2 | 0.55 | | 1.82E-134 | |
| B | B | MT-ATP6 | 0.65 | | 3.35E-134 | |
| B | B | MT-ND2 | 0.57 | | 8.76E-129 | |
| B | B | CXCR4 | 0.89 | | 1.28E-108 | |
| B | B | GAPDH | -0.82 | | 4.14E-90 | |
| B | B | MS4A1 | 0.9 | | 1.56E-86 | |
| B | B | MALAT1 | 0.46 | | 1.60E-80 | |
| B | B | MT-ND1 | 0.44 | | 7.20E-73 | |
| B | B | S100A11 | -1.06 | | 1.32E-72 | |
| B | B | TXN | -1.22 | | 5.43E-72 | |
| B | B | CD63 | -1.06 | | 1.99E-65 | |
| B | B | GSTP1 | -0.87 | | 2.57E-65 | |
| B | B | ATP5MC3 | -0.67 | | 5.02E-61 | |
| B | B | CSTB | -0.91 | | 2.08E-59 | |
| B | B | LGALS3 | -0.92 | | 3.22E-57 | |
| B | B | TIMP1 | -2.16 | | 3.95E-56 | |
| B | B | CST3 | -1.53 | | 2.26E-55 | |
| B | B | SERF2 | -0.45 | | 4.07E-55 | |
| B | B | S100A6 | -1.36 | | 8.09E-53 | |
| B | B | S100A10 | -0.81 | | 2.05E-52 | |
| B | B | ACTG1 | -0.66 | | 7.77E-52 | |
| B | B | ANXA2 | -0.81 | | 3.42E-51 | |
| B | B | UQCRQ | -0.59 | | 2.93E-50 | |
| B | B | ELOB | -0.58 | | 5.61E-50 | |
| B | B | MYL6 | -0.48 | | 1.42E-49 | |
| B | B | CD69 | 0.79 | | 5.34E-49 | |
| B | B | SEC61G | -0.57 | | 1.17E-48 | |
| B | B | CTSD | -0.84 | | 5.92E-47 | |
| B | B | CD37 | 0.56 | | 1.16E-46 | |
| B | B | TMSB10 | -0.52 | | 1.53E-46 | |
| B | B | COX8A | -0.55 | | 2.45E-46 | |
| B | B | COX5A | -0.52 | | 8.59E-46 | |
| B | B | LGALS1 | -1.24 | | 1.91E-45 | |
| B | B | MT-ND5 | 0.52 | | 1.23E-43 | |
| B | B | RAC1 | -0.6 | | 1.64E-43 | |
| B | B | VIM | -0.72 | | 2.44E-42 | |
| B | B | FTL | -1.02 | | 3.65E-42 | |
| B | B | TMEM258 | -0.53 | | 5.46E-42 | |
| B | B | GPX1 | -0.67 | | 8.10E-42 | |
| B | B | CD79A | 0.57 | | 2.56E-41 | |
| B | B | ATOX1 | -0.5 | | 2.59E-41 | |
| B | B | COX6C | -0.48 | | 6.89E-41 | |
| B | B | CTSB | -0.95 | | 9.90E-41 | |
| B | B | MIF | -0.49 | | 2.74E-40 | |
| B | B | ENO1 | -0.53 | | 5.63E-40 | |
| B | B | PHPT1 | -0.44 | | 7.64E-40 | |
| B | B | P4HB | -0.48 | | 8.78E-40 | |
| B | B | PRDX5 | -0.47 | | 9.23E-40 | |
| B | B | IFITM3 | -1.25 | | 2.58E-39 | |
| B | B | NDUFB7 | -0.46 | | 2.93E-39 | |
| B | B | MT2A | -1.15 | | 4.38E-39 | |
| B | B | LY6E | -0.6 | | 8.01E-39 | |
| B | B | SSR4 | -1.43 | | 9.73E-39 | |
| B | B | RPS27L | -0.48 | | 3.43E-38 | |
| B | B | ATP5PF | -0.44 | | 3.74E-38 | |
| B | B | CD59 | -0.52 | | 3.74E-38 | |
| B | B | DSTN | -0.58 | | 4.83E-38 | |
| B | B | ATP5F1E | -0.38 | | 7.50E-38 | |
| B | B | HLA-DQB1 | 0.7 | | 8.44E-38 | |
| B | B | NDUFC2 | -0.42 | | 1.14E-37 | |
| B | B | BANK1 | 0.59 | | 2.19E-37 | |
| B | B | PSME2 | -0.48 | | 5.58E-37 | |
| B | B | FIS1 | -0.39 | | 6.11E-37 | |
| B | B | PSAP | -0.69 | | 1.05E-36 | |
| B | B | NDUFB2 | -0.37 | | 2.02E-36 | |
| B | B | S100A4 | -0.92 | | 2.07E-36 | |
| B | B | SDF2L1 | -0.53 | | 2.10E-36 | |
| B | B | PPIB | -0.49 | | 4.50E-36 | |
| B | B | CFLAR | -0.44 | | 5.59E-36 | |
| B | B | NDUFA4 | -0.43 | | 1.25E-35 | |
| B | B | PPA1 | -0.49 | | 1.66E-35 | |
| B | B | ATP5MD | -0.43 | | 2.53E-35 | |
| B | B | NDUFB10 | -0.39 | | 3.30E-35 | |
| B | B | CXCL8 | -2.29 | | 3.94E-35 | |
| B | B | ATP5F1D | -0.44 | | 4.09E-35 | |
| B | B | ATP5ME | -0.44 | | 5.23E-35 | |
| B | B | NDUFS6 | -0.4 | | 9.17E-35 | |
| B | B | BST2 | -0.5 | | 1.24E-34 | |
| B | B | REEP5 | -0.38 | | 1.27E-34 | |
| B | B | GNG5 | -0.38 | | 2.17E-34 | |
| B | B | RHOC | -0.53 | | 2.25E-34 | |
| B | B | NEDD8 | -0.35 | | 2.47E-34 | |
| B | B | HSPB1 | -0.72 | | 2.73E-34 | |
| B | B | KDELR1 | -0.36 | | 4.71E-34 | |
| B | B | CYTOR | -0.6 | | 7.60E-34 | |
| B | B | PKM | -0.47 | | 7.65E-34 | |
| B | B | AP2S1 | -0.39 | | 1.16E-33 | |
| B | B | TRIB1 | -0.54 | | 1.38E-33 | |
| B | B | NINJ1 | -0.58 | | 1.46E-33 | |
| B | B | KRTCAP2 | -0.42 | | 1.47E-33 | |
| B | B | HLA-DQA1 | 0.58 | | 1.57E-33 | |
| B | B | TUBB | -0.51 | | 1.65E-33 | |
| B | B | CALM3 | -0.36 | | 1.65E-33 | |
| B | B | NPC2 | -0.74 | | 1.80E-33 | |
| B | B | FKBP2 | -0.6 | | 2.73E-33 | |
| B | B | JTB | -0.37 | | 2.73E-33 | |
| B | B | PRDX4 | -0.77 | | 3.35E-33 | |
| B | B | LDHA | -0.46 | | 4.71E-33 | |
| B | B | NDUFS8 | -0.34 | | 4.90E-33 | |
| B | B | TPI1 | -0.4 | | 5.39E-33 | |
| B | B | IFI6 | -0.67 | | 5.39E-33 | |
| B | B | CD74 | 0.66 | | 9.37E-33 | |
| B | B | VPREB3 | 0.58 | | 1.48E-32 | |
| B | B | SEC61B | -0.48 | | 1.48E-32 | |
| B | B | MGST3 | -0.47 | | 1.76E-32 | |
| B | B | XBP1 | -0.88 | | 1.81E-32 | |
| B | B | SPCS1 | -0.51 | | 3.97E-32 | |
| B | B | NAA38 | -0.33 | | 3.97E-32 | |
| B | B | CD151 | -0.41 | | 4.59E-32 | |
| B | B | TSPO | -0.5 | | 4.65E-32 | |
| B | B | ALDOA | -0.39 | | 7.49E-32 | |
| B | B | IER3 | -0.92 | | 8.96E-32 | |
| B | B | SUB1 | -0.38 | | 1.04E-31 | |
| B | B | PRELID1 | -0.37 | | 1.63E-31 | |
| B | B | NDUFB4 | -0.35 | | 1.77E-31 | |
| B | B | ANAPC11 | -0.34 | | 1.77E-31 | |
| B | B | ATP5IF1 | -0.37 | | 2.87E-31 | |
| B | B | ANXA5 | -0.56 | | 5.35E-31 | |
| B | B | BSG | -0.35 | | 9.71E-31 | |
| B | B | SELENOS | -0.42 | | 1.15E-30 | |
| B | B | GSN | -0.9 | | 1.42E-30 | |
| B | B | NME1 | -0.34 | | 1.50E-30 | |
| B | B | SSR3 | -0.53 | | 1.70E-30 | |
| B | B | HLA-DRA | 0.61 | | 1.83E-30 | |
| B | B | NFKBIA | -0.47 | | 5.36E-30 | |
| B | B | ETFB | -0.35 | | 7.30E-30 | |
| B | B | ATP5PD | -0.34 | | 7.46E-30 | |
| B | B | PEBP1 | -0.34 | | 1.02E-29 | |
| B | B | ARL6IP4 | -0.34 | | 1.21E-29 | |
| B | B | MANF | -0.44 | | 1.28E-29 | |
| B | B | DAD1 | -0.35 | | 1.44E-29 | |
| B | B | AURKAIP1 | -0.3 | | 1.82E-29 | |
| B | B | ZNHIT1 | -0.32 | | 2.52E-29 | |
| B | B | IDH2 | -0.38 | | 2.84E-29 | |
| B | B | PDIA6 | -0.42 | | 3.41E-29 | |
| B | B | MIR4435-2HG | -0.43 | | 3.46E-29 | |
| B | B | DNPH1 | -0.34 | | 4.63E-29 | |
| B | B | NDUFA3 | -0.29 | | 4.78E-29 | |
| B | B | NDUFAB1 | -0.31 | | 5.02E-29 | |
| B | B | ID2 | -0.61 | | 7.30E-29 | |
| B | B | TPT1 | -0.26 | | 9.60E-29 | |
| B | B | COX6B1 | -0.4 | | 9.96E-29 | |
| B | B | NDUFA1 | -0.37 | | 1.20E-28 | |
| B | B | WDR83OS | -0.28 | | 1.69E-28 | |
| B | B | SCAND1 | -0.28 | | 1.72E-28 | |
| B | B | SH3BGRL3 | -0.51 | | 2.38E-28 | |
| B | B | C4orf48 | -0.32 | | 2.72E-28 | |
| B | B | HM13 | -0.31 | | 2.81E-28 | |
| B | B | TALDO1 | -0.32 | | 2.98E-28 | |
| B | B | FKBP1A | -0.43 | | 2.98E-28 | |
| B | B | ISG15 | -0.63 | | 4.11E-28 | |
| B | B | PRDX2 | -0.34 | | 4.29E-28 | |
| B | B | LYZ | -1.7 | | 4.38E-28 | |
| B | B | CXCL2 | -1.49 | | 6.14E-28 | |
| B | B | HSP90B1 | -0.61 | | 6.15E-28 | |
| B | B | CAPZB | -0.35 | | 6.31E-28 | |
| B | B | PLAUR | -1.11 | | 7.23E-28 | |
| B | B | IFI27 | -1.18 | | 7.79E-28 | |
| B | B | ATP5F1C | -0.33 | | 7.79E-28 | |
| B | B | TMEM59 | -0.48 | | 1.08E-27 | |
| B | B | BRK1 | -0.34 | | 1.42E-27 | |
| B | B | PFN1 | -0.49 | | 1.74E-27 | |
| B | B | GLUL | -0.63 | | 1.90E-27 | |
| B | B | COX5B | -0.38 | | 2.96E-27 | |
| B | B | GLRX | -0.41 | | 3.16E-27 | |
| B | B | PSMB2 | -0.29 | | 3.70E-27 | |
| B | B | SOD2 | -1.36 | | 3.78E-27 | |
| B | B | RRBP1 | -0.33 | | 4.11E-27 | |
| B | B | CD9 | -0.61 | | 5.52E-27 | |
| B | B | OSTC | -0.28 | | 5.53E-27 | |
| B | B | PDIA4 | -0.37 | | 5.78E-27 | |
| B | B | PARK7 | -0.33 | | 6.48E-27 | |
| B | B | EZR | 0.47 | | 8.96E-27 | |
| B | B | PSMA7 | -0.31 | | 9.11E-27 | |
| B | B | TMEM14C | -0.3 | | 9.67E-27 | |
| B | B | RARRES3 | -0.46 | | 1.21E-26 | |
| B | B | PTMS | -0.42 | | 1.45E-26 | |
| B | B | PRDX1 | -0.4 | | 1.57E-26 | |
| B | B | CTSC | -0.43 | | 1.62E-26 | |
| B | B | CYC1 | -0.31 | | 1.78E-26 | |
| B | B | BLOC1S1 | -0.34 | | 1.93E-26 | |
| B | B | GRN | -0.4 | | 3.56E-26 | |
| B | B | GSTO1 | -0.38 | | 3.56E-26 | |
| B | B | LAMP2 | -0.25 | | 3.80E-26 | |
| B | B | IL32 | -0.61 | | 4.58E-26 | |
| B | B | PHLDA2 | -0.53 | | 6.49E-26 | |
| B | B | MRPL57 | -0.28 | | 6.96E-26 | |
| B | B | MINOS1 | -0.33 | | 8.07E-26 | |
| B | B | BLVRB | -0.37 | | 8.32E-26 | |
| B | B | NDUFB9 | -0.31 | | 9.22E-26 | |
| B | B | FKBP11 | -0.88 | | 9.67E-26 | |
| B | B | SEM1 | -0.31 | | 1.12E-25 | |
| B | B | TIMM13 | -0.3 | | 1.32E-25 | |
| B | B | ATP5F1A | -0.3 | | 1.61E-25 | |
| B | B | CD19 | 0.54 | | 1.68E-25 | |
| B | B | ATP5MG | -0.29 | | 1.79E-25 | |
| B | B | NDUFB11 | -0.28 | | 2.03E-25 | |
| B | B | COPE | -0.33 | | 2.22E-25 | |
| B | B | TMEM205 | -0.26 | | 2.81E-25 | |
| B | B | UBL5 | -0.31 | | 3.23E-25 | |
| B | B | ERGIC3 | -0.32 | | 4.37E-25 | |
| B | B | MZT2B | -0.29 | | 4.66E-25 | |
| B | B | CHCHD2 | -0.29 | | 5.40E-25 | |
| B | B | COX7B | -0.36 | | 5.69E-25 | |
| B | B | LAMTOR2 | -0.27 | | 6.01E-25 | |
| B | B | IFI27L2 | -0.28 | | 6.34E-25 | |
| B | B | NDUFA11 | -0.28 | | 6.37E-25 | |
| B | B | NDUFA13 | -0.26 | | 6.90E-25 | |
| B | B | GUK1 | -0.32 | | 6.94E-25 | |
| B | B | APRT | -0.29 | | 7.60E-25 | |
| B | B | MGAT1 | -0.3 | | 8.82E-25 | |
| B | B | CNPY2 | -0.25 | | 9.07E-25 | |
| B | B | KDELR2 | -0.29 | | 9.51E-25 | |
| B | B | RPN2 | -0.32 | | 1.01E-24 | |
| B | B | C15orf48 | -1.36 | | 1.17E-24 | |
| B | B | MDH2 | -0.27 | | 1.20E-24 | |
| B | B | TYROBP | -1.34 | | 1.24E-24 | |
| B | B | NDUFB3 | -0.28 | | 1.37E-24 | |
| B | B | RGS10 | -0.38 | | 1.60E-24 | |
| B | B | PDIA3 | -0.29 | | 1.98E-24 | |
| B | B | LAMTOR4 | -0.28 | | 2.85E-24 | |
| B | B | GADD45A | -0.44 | | 4.21E-24 | |
| B | B | IFITM1 | -0.54 | | 4.36E-24 | |
| B | B | PRDX6 | -0.27 | | 4.45E-24 | |
| B | B | HLA-DPB1 | 0.32 | | 4.81E-24 | |
| B | B | PPIF | -0.4 | | 5.88E-24 | |
| B | B | LY9 | 0.52 | | 7.98E-24 | |
| B | B | TPM3 | -0.29 | | 8.46E-24 | |
| B | B | TMED9 | -0.27 | | 1.00E-23 | |
| B | B | SELENOW | -0.34 | | 1.05E-23 | |
| B | B | GPX4 | -0.31 | | 1.19E-23 | |
| B | B | HLA-DRB1 | 0.52 | | 1.19E-23 | |
| B | B | CXCL3 | -1.29 | | 1.19E-23 | |
| B | B | PPDPF | -0.4 | | 1.73E-23 | |
| B | B | TNIP1 | -0.26 | | 2.02E-23 | |
| B | B | ATP5MC1 | -0.29 | | 2.03E-23 | |
| B | B | NDUFB1 | -0.29 | | 2.10E-23 | |
| B | B | MRPL12 | -0.27 | | 2.42E-23 | |
| B | B | FCER1G | -1.52 | | 2.68E-23 | |
| B | B | BRI3 | -0.28 | | 2.68E-23 | |
| B | B | CEBPB | -0.4 | | 3.26E-23 | |
| B | B | COX7A2 | -0.33 | | 3.43E-23 | |
| B | B | ACTN4 | -0.28 | | 3.90E-23 | |
| B | B | ARPC1B | -0.39 | | 4.02E-23 | |
| B | B | SEC11C | -0.6 | | 4.48E-23 | |
| B | B | COX6A1 | -0.3 | | 4.83E-23 | |
| B | B | PCBD1 | -0.27 | | 5.59E-23 | |
| B | B | C9orf16 | -0.28 | | 5.65E-23 | |
| B | B | RAB13 | -0.33 | | 5.77E-23 | |
| B | B | TIMM8B | -0.28 | | 5.89E-23 | |
| B | B | CHCHD10 | -0.33 | | 6.47E-23 | |
| B | B | ERLEC1 | -0.3 | | 6.70E-23 | |
| B | B | EFHD2 | -0.26 | | 7.30E-23 | |
| B | B | HSBP1 | -0.26 | | 7.71E-23 | |
| B | B | SLIRP | -0.25 | | 8.26E-23 | |
| B | B | PLD3 | -0.3 | | 9.15E-23 | |
| B | B | TUBA1C | -0.28 | | 9.50E-23 | |
| B | B | FCGRT | -0.35 | | 9.70E-23 | |
| B | B | BID | -0.26 | | 1.01E-22 | |
| B | B | SLC25A5 | -0.32 | | 1.11E-22 | |
| B | B | RABAC1 | -0.33 | | 1.30E-22 | |
| B | B | CAPN2 | -0.26 | | 1.32E-22 | |
| B | B | SRM | -0.27 | | 1.45E-22 | |
| B | B | TSC22D3 | 0.31 | | 1.57E-22 | |
| B | B | SRP14 | -0.3 | | 3.67E-22 | |
| B | B | VDAC1 | -0.26 | | 4.04E-22 | |
| B | B | SERPINB1 | -0.37 | | 4.45E-22 | |
| B | B | CREG1 | -0.28 | | 4.63E-22 | |
| B | B | GAS6 | -0.28 | | 4.79E-22 | |
| B | B | ITM2C | -0.55 | | 5.01E-22 | |
| B | B | MYDGF | -0.34 | | 5.66E-22 | |
| B | B | PPP1CA | -0.27 | | 6.45E-22 | |
| B | B | CEBPD | -0.4 | | 7.80E-22 | |
| B | B | FABP5 | -0.55 | | 1.03E-21 | |
| B | B | SPCS3 | -0.35 | | 1.09E-21 | |
| B | B | HLA-DPA1 | 0.38 | | 1.19E-21 | |
| B | B | ITGB1 | -0.31 | | 1.19E-21 | |
| B | B | H2AFJ | -0.26 | | 1.24E-21 | |
| B | B | ELOC | -0.25 | | 1.36E-21 | |
| B | B | ITM2B | -0.37 | | 1.37E-21 | |
| B | B | UQCRH | -0.32 | | 1.40E-21 | |
| B | B | CTSH | -0.33 | | 1.46E-21 | |
| B | B | C12orf75 | -0.37 | | 2.23E-21 | |
| B | B | MT1X | -0.56 | | 2.48E-21 | |
| B | B | PIM1 | -0.31 | | 2.66E-21 | |
| B | B | CTSZ | -0.28 | | 2.73E-21 | |
| B | B | SCP2 | -0.25 | | 3.11E-21 | |
| B | B | PRDM1 | -0.38 | | 3.78E-21 | |
| B | B | TSC22D1 | -0.5 | | 3.93E-21 | |
| B | B | CKLF | -0.28 | | 4.25E-21 | |
| B | B | BHLHE40 | -0.29 | | 4.79E-21 | |
| B | B | ANXA11 | -0.26 | | 4.95E-21 | |
| B | B | LAPTM5 | 0.41 | | 4.98E-21 | |
| B | B | NDUFS5 | -0.27 | | 5.05E-21 | |
| B | B | MDK | -0.45 | | 5.72E-21 | |
| B | B | LGALS3BP | -0.39 | | 5.86E-21 | |
| B | B | MT-ND4L | 0.5 | | 6.12E-21 | |
| B | B | PHLDA1 | -0.41 | | 6.65E-21 | |
| B | B | TPM1 | -0.36 | | 6.95E-21 | |
| B | B | NUCB2 | -0.34 | | 7.72E-21 | |
| B | B | PGK1 | -0.31 | | 8.69E-21 | |
| B | B | ALDH2 | -0.3 | | 8.71E-21 | |
| B | B | LY96 | -0.29 | | 9.62E-21 | |
| B | B | RUBCNL | 0.43 | | 1.15E-20 | |
| B | B | HMGA1 | -0.32 | | 1.21E-20 | |
| B | B | NR4A2 | 0.57 | | 1.26E-20 | |
| B | B | POMP | -0.28 | | 1.41E-20 | |
| B | B | CD68 | -0.44 | | 1.42E-20 | |
| B | B | MGST2 | -0.28 | | 1.57E-20 | |
| B | B | UPP1 | -0.28 | | 1.58E-20 | |
| B | B | EDF1 | -0.26 | | 1.71E-20 | |
| B | B | CTSL | -0.67 | | 1.83E-20 | |
| B | B | VAMP8 | -0.27 | | 1.89E-20 | |
| B | B | TPM4 | -0.27 | | 1.96E-20 | |
| B | B | ATP5MPL | -0.25 | | 2.22E-20 | |
| B | B | NECTIN2 | -0.26 | | 2.82E-20 | |
| B | B | ANKRD28 | -0.38 | | 2.89E-20 | |
| B | B | MT1E | -0.66 | | 3.54E-20 | |
| B | B | MZB1 | -1.61 | | 4.85E-20 | |
| B | B | SOX4 | -0.41 | | 5.16E-20 | |
| B | B | ETS2 | -0.32 | | 5.89E-20 | |
| B | B | TCEAL9 | -0.29 | | 6.79E-20 | |
| B | B | PLIN2 | -0.35 | | 8.96E-20 | |
| B | B | ATP1B1 | -0.32 | | 9.95E-20 | |
| B | B | UBE2J1 | -0.28 | | 1.13E-19 | |
| B | B | H1FX | -0.29 | | 1.23E-19 | |
| B | B | OST4 | -0.28 | | 1.30E-19 | |
| B | B | JPT1 | -0.3 | | 1.43E-19 | |
| B | B | CD99 | -0.26 | | 1.48E-19 | |
| B | B | S100A13 | -0.32 | | 1.88E-19 | |
| B | B | PSME1 | -0.29 | | 2.16E-19 | |
| B | B | CARD16 | -0.35 | | 2.35E-19 | |
| B | B | BTG1 | 0.26 | | 3.02E-19 | |
| B | B | SQSTM1 | -0.33 | | 3.08E-19 | |
| B | B | TMSB4X | -0.38 | | 4.16E-19 | |
| B | B | NAMPT | -0.3 | | 4.51E-19 | |
| B | B | CD83 | 0.57 | | 5.17E-19 | |
| B | B | ATP6V1F | -0.27 | | 5.59E-19 | |
| B | B | ANXA4 | -0.36 | | 8.26E-19 | |
| B | B | CYB5A | -0.25 | | 9.23E-19 | |
| B | B | CFL1 | -0.3 | | 9.29E-19 | |
| B | B | TMEM176B | -0.43 | | 1.35E-18 | |
| B | B | HIST1H2BK | -0.34 | | 2.14E-18 | |
| B | B | KRT18 | -1.01 | | 3.43E-18 | |
| B | B | EMP1 | -0.51 | | 6.33E-18 | |
| B | B | MYL12A | -0.29 | | 7.79E-18 | |
| B | B | MAFF | -0.28 | | 1.00E-17 | |
| B | B | VAMP5 | -0.27 | | 1.41E-17 | |
| B | B | IL1B | -1.61 | | 1.49E-17 | |
| B | B | DUSP5 | -0.38 | | 1.61E-17 | |
| B | B | ADIRF | -0.83 | | 1.62E-17 | |
| B | B | DERL3 | -1 | | 2.04E-17 | |
| B | B | TM4SF1 | -0.99 | | 2.44E-17 | |
| B | B | SELENOM | -0.34 | | 3.01E-17 | |
| B | B | GMDS | -0.25 | | 3.48E-17 | |
| B | B | HIST1H1C | -0.46 | | 3.62E-17 | |
| B | B | SLC7A5 | -0.3 | | 3.70E-17 | |
| B | B | APP | -0.29 | | 4.02E-17 | |
| B | B | EPCAM | -0.45 | | 4.90E-17 | |
| B | B | NEAT1 | -0.44 | | 5.85E-17 | |
| B | B | ID1 | -0.47 | | 7.29E-17 | |
| B | B | CLIC1 | -0.27 | | 1.05E-16 | |
| B | B | DNAJB9 | -0.33 | | 1.56E-16 | |
| B | B | SDC4 | -0.28 | | 2.17E-16 | |
| B | B | KRT19 | -0.82 | | 2.28E-16 | |
| B | B | CD320 | -0.27 | | 2.49E-16 | |
| B | B | IFITM2 | -0.38 | | 2.60E-16 | |
| B | B | TMEM176A | -0.3 | | 2.80E-16 | |
| B | B | SOCS3 | -0.63 | | 3.62E-16 | |
| B | B | G0S2 | -1.45 | | 4.20E-16 | |
| B | B | IL1RN | -0.72 | | 4.37E-16 | |
| B | B | C19orf33 | -0.49 | | 4.61E-16 | |
| B | B | SMIM22 | -0.45 | | 7.67E-16 | |
| B | B | TPM2 | -0.43 | | 7.89E-16 | |
| B | B | S100A16 | -0.32 | | 7.98E-16 | |
| B | B | SGK1 | -0.31 | | 8.45E-16 | |
| B | B | TNFRSF18 | -0.42 | | 1.33E-15 | |
| B | B | KRT8 | -1.1 | | 1.42E-15 | |
| B | B | DUSP4 | -0.3 | | 1.54E-15 | |
| B | B | CITED2 | -0.34 | | 2.38E-15 | |
| B | B | REL | 0.46 | | 2.41E-15 | |
| B | B | JSRP1 | -0.44 | | 3.18E-15 | |
| B | B | S100A14 | -0.43 | | 3.29E-15 | |
| B | B | PERP | -0.25 | | 3.73E-15 | |
| B | B | TNFRSF12A | -0.26 | | 4.40E-15 | |
| B | B | ATF3 | -0.36 | | 4.40E-15 | |
| B | B | SELENOP | -0.41 | | 5.16E-15 | |
| B | B | SPINT2 | -0.25 | | 1.14E-14 | |
| B | B | STK4 | 0.44 | | 1.18E-14 | |
| B | B | STARD10 | -0.25 | | 1.20E-14 | |
| B | B | COTL1 | -0.37 | | 1.73E-14 | |
| B | B | EPAS1 | -0.26 | | 1.92E-14 | |
| B | B | CDKN1A | -0.32 | | 4.39E-14 | |
| B | B | CAPG | -0.34 | | 4.69E-14 | |
| B | B | KLF4 | -0.31 | | 5.35E-14 | |
| B | B | ARPC2 | -0.27 | | 6.00E-14 | |
| B | B | CLU | -0.55 | | 6.42E-14 | |
| B | B | AC020656.1 | -0.42 | | 8.14E-14 | |
| B | B | STOM | -0.26 | | 1.20E-13 | |
| B | B | ARHGAP24 | 0.41 | | 1.26E-13 | |
| B | B | GPX2 | -0.34 | | 1.33E-13 | |
| B | B | YPEL5 | 0.34 | | 2.05E-13 | |
| B | B | ELF3 | -0.59 | | 2.07E-13 | |
| B | B | TYMP | -0.35 | | 2.45E-13 | |
| B | B | LINC00926 | 0.38 | | 2.97E-13 | |
| B | B | ICAM1 | -0.31 | | 3.34E-13 | |
| B | B | ZNF331 | 0.48 | | 3.57E-13 | |
| B | B | NQO1 | -0.31 | | 3.90E-13 | |
| B | B | CCL20 | -0.93 | | 4.05E-13 | |
| B | B | TNFRSF4 | -0.35 | | 8.00E-13 | |
| B | B | CD22 | 0.37 | | 9.12E-13 | |
| B | B | SAT1 | -0.55 | | 1.31E-12 | |
| B | B | BATF | -0.37 | | 1.61E-12 | |
| B | B | IGFBP7 | -1.68 | | 2.65E-12 | |
| B | B | RIC3 | 0.33 | | 2.94E-12 | |
| B | B | HSPA5 | -0.28 | | 3.89E-12 | |
| B | B | PHACTR1 | 0.52 | | 4.06E-12 | |
| B | B | HCST | -0.26 | | 5.37E-12 | |
| B | B | SPCS2 | -0.27 | | 7.42E-12 | |
| B | B | FCER2 | 0.36 | | 1.36E-11 | |
| B | B | CD27 | -0.31 | | 1.53E-11 | |
| B | B | GNLY | -0.82 | | 2.08E-11 | |
| B | B | PIGR | -0.73 | | 2.27E-11 | |
| B | B | ACTB | -0.46 | | 3.46E-11 | |
| B | B | GZMB | -0.89 | | 3.69E-11 | |
| B | B | CFD | -0.62 | | 3.69E-11 | |
| B | B | VSIG2 | -0.28 | | 4.03E-11 | |
| B | B | AIF1 | -0.61 | | 6.06E-11 | |
| B | B | LGALS4 | -0.54 | | 6.50E-11 | |
| B | B | IGLL5 | -2.91 | | 9.00E-11 | |
| B | B | IGFBP4 | -0.47 | | 9.40E-11 | |
| B | B | CD55 | 0.39 | | 1.71E-10 | |
| B | B | HES1 | -0.5 | | 3.24E-10 | |
| B | B | HLA-DQA2 | 0.38 | | 3.60E-10 | |
| B | B | CLDN4 | -0.44 | | 4.44E-10 | |
| B | B | ANKRD44 | 0.36 | | 4.63E-10 | |
| B | B | FXYD3 | -0.62 | | 6.50E-10 | |
| B | B | LST1 | -0.35 | | 1.19E-09 | |
| B | B | PSCA | -0.82 | | 1.52E-09 | |
| B | B | NFKBID | 0.46 | | 2.02E-09 | |
| B | B | MUC1 | -0.46 | | 2.06E-09 | |
| B | B | S100P | -0.83 | | 2.23E-09 | |
| B | B | CCR7 | 0.38 | | 2.54E-09 | |
| B | B | LCN2 | -0.69 | | 3.41E-09 | |
| B | B | ANXA10 | -0.37 | | 4.60E-09 | |
| B | B | MT1G | -0.7 | | 5.16E-09 | |
| B | B | SPINK1 | -1 | | 8.07E-09 | |
| B | B | FCMR | 0.37 | | 9.15E-09 | |
| B | B | HLA-DMA | 0.46 | | 9.49E-09 | |
| B | B | CD3D | -0.38 | | 1.10E-08 | |
| B | B | GPR183 | 0.32 | | 2.02E-08 | |
| B | B | FTH1 | -0.57 | | 2.53E-08 | |
| B | B | CD7 | -0.46 | | 2.56E-08 | |
| B | B | KLF6 | 0.35 | | 2.85E-08 | |
| B | B | AGR2 | -0.98 | | 3.18E-08 | |
| B | B | TSPAN8 | -0.88 | | 4.96E-08 | |
| B | B | SPIB | 0.34 | | 6.43E-08 | |
| B | B | CD247 | -0.27 | | 6.59E-08 | |
| B | B | RIPOR2 | 0.28 | | 8.89E-08 | |
| B | B | HOPX | -0.29 | | 9.21E-08 | |
| B | B | CST7 | -0.34 | | 1.12E-07 | |
| B | B | CYBA | -0.28 | | 2.06E-07 | |
| B | B | CA2 | -0.5 | | 2.43E-07 | |
| B | B | SNX29P2 | 0.29 | | 3.75E-07 | |
| B | B | MTRNR2L8 | 0.31 | | 6.81E-07 | |
| B | B | SRSF7 | 0.29 | | 9.27E-07 | |
| B | B | CTSW | -0.27 | | 1.12E-06 | |
| B | B | AC020916.1 | 0.46 | | 1.22E-06 | |
| B | B | CYSTM1 | -0.54 | | 1.25E-06 | |
| B | B | MEF2C | 0.4 | | 1.27E-06 | |
| B | B | GZMA | -0.5 | | 1.46E-06 | |
| B | B | CD2 | -0.32 | | 1.55E-06 | |
| B | B | LINC00513 | 0.34 | | 1.69E-06 | |
| B | B | SLC2A3 | 0.32 | | 1.77E-06 | |
| B | B | SELL | 0.35 | | 2.12E-06 | |
| B | B | SMCHD1 | 0.41 | | 2.34E-06 | |
| B | B | PRF1 | -0.35 | | 2.35E-06 | |
| B | B | NKG7 | -0.47 | | 2.47E-06 | |
| B | B | HLA-DRB5 | 0.35 | | 3.73E-06 | |
| B | B | MTRNR2L1 | 0.34 | | 4.18E-06 | |
| B | B | MTRNR2L2 | 0.28 | | 9.89E-06 | |
| Epithelial | Epithelial | PGC | 5.22 | | 0 | |
| Epithelial | Epithelial | LIPF | 5.07 | | 0 | |
| Epithelial | Epithelial | TFF2 | 3.44 | | 0 | |
| Epithelial | Epithelial | PSCA | 3.01 | | 0 | |
| Epithelial | Epithelial | MUC5AC | 2.9 | | 0 | |
| Epithelial | Epithelial | BPIFB1 | 2.89 | | 0 | |
| Epithelial | Epithelial | TFF1 | 2.85 | | 0 | |
| Epithelial | Epithelial | MUC6 | 2.81 | | 0 | |
| Epithelial | Epithelial | SPINK1 | 2.62 | | 0 | |
| Epithelial | Epithelial | PGA3 | 2.51 | | 0 | |
| Epithelial | Epithelial | MSMB | 2.4 | | 0 | |
| Epithelial | Epithelial | PGA4 | 2.37 | | 0 | |
| Epithelial | Epithelial | AGR2 | 2.35 | | 0 | |
| Epithelial | Epithelial | LYZ | 2.18 | | 0 | |
| Epithelial | Epithelial | GKN2 | 2.11 | | 0 | |
| Epithelial | Epithelial | REG3A | 2.1 | | 0 | |
| Epithelial | Epithelial | PIGR | 2.09 | | 0 | |
| Epithelial | Epithelial | REG1A | 2.07 | | 0 | |
| Epithelial | Epithelial | CXCL17 | 1.97 | | 0 | |
| Epithelial | Epithelial | LCN2 | 1.96 | | 0 | |
| Epithelial | Epithelial | LTF | 1.92 | | 0 | |
| Epithelial | Epithelial | MT1G | 1.88 | | 0 | |
| Epithelial | Epithelial | CYSTM1 | 1.87 | | 0 | |
| Epithelial | Epithelial | MT1E | 1.79 | | 0 | |
| Epithelial | Epithelial | MUC1 | 1.65 | | 0 | |
| Epithelial | Epithelial | CLDN18 | 1.59 | | 0 | |
| Epithelial | Epithelial | RNASE1 | 1.59 | | 0 | |
| Epithelial | Epithelial | ELF3 | 1.5 | | 0 | |
| Epithelial | Epithelial | SMIM22 | 1.45 | | 0 | |
| Epithelial | Epithelial | KRT19 | 1.44 | | 0 | |
| Epithelial | Epithelial | KRT8 | 1.4 | | 0 | |
| Epithelial | Epithelial | VSIG2 | 1.39 | | 0 | |
| Epithelial | Epithelial | CA2 | 1.38 | | 0 | |
| Epithelial | Epithelial | ANXA10 | 1.38 | | 0 | |
| Epithelial | Epithelial | GPX2 | 1.35 | | 0 | |
| Epithelial | Epithelial | C19orf33 | 1.34 | | 0 | |
| Epithelial | Epithelial | ALDH3A1 | 1.3 | | 0 | |
| Epithelial | Epithelial | KRT18 | 1.28 | | 0 | |
| Epithelial | Epithelial | C6orf58 | 1.22 | | 0 | |
| Epithelial | Epithelial | IGFBP2 | 1.22 | | 0 | |
| Epithelial | Epithelial | ALDH1A1 | 1.21 | | 0 | |
| Epithelial | Epithelial | FAM3D | 1.18 | | 0 | |
| Epithelial | Epithelial | KCNE2 | 1.17 | | 0 | |
| Epithelial | Epithelial | CTSE | 1.14 | | 0 | |
| Epithelial | Epithelial | FXYD3 | 1.13 | | 0 | |
| Epithelial | Epithelial | STARD10 | 1.07 | | 0 | |
| Epithelial | Epithelial | SMIM24 | 1.03 | | 0 | |
| Epithelial | Epithelial | GSTA1 | 1.03 | | 0 | |
| Epithelial | Epithelial | WFDC2 | 0.99 | | 0 | |
| Epithelial | Epithelial | MT1M | 0.93 | | 0 | |
| Epithelial | Epithelial | FAM3B | 0.83 | | 0 | |
| Epithelial | Epithelial | AZGP1 | 0.79 | | 0 | |
| Epithelial | Epithelial | AQP5 | 0.64 | | 0 | |
| Epithelial | Epithelial | CA9 | 0.63 | | 0 | |
| Epithelial | Epithelial | KIAA1324 | 0.57 | | 0 | |
| Epithelial | Epithelial | GATA6-AS1 | 0.55 | | 0 | |
| Epithelial | Epithelial | FOLR1 | 0.4 | | 0 | |
| Epithelial | Epithelial | REP15 | 0.37 | | 0 | |
| Epithelial | Epithelial | C16orf89 | 0.31 | | 0 | |
| Epithelial | Epithelial | ORM2 | 0.3 | | 0 | |
| Epithelial | Epithelial | FUT9 | 0.29 | | 0 | |
| Epithelial | Epithelial | B2M | -1.76 | | 0 | |
| Epithelial | Epithelial | S100P | 1.62 | | 5.49E-303 | |
| Epithelial | Epithelial | SPDEF | 0.42 | | 6.03E-302 | |
| Epithelial | Epithelial | SCNN1A | 0.48 | | 4.72E-299 | |
| Epithelial | Epithelial | TMPRSS2 | 0.71 | | 6.62E-299 | |
| Epithelial | Epithelial | ZG16B | 1.01 | | 1.84E-298 | |
| Epithelial | Epithelial | TCEA3 | 0.57 | | 3.01E-291 | |
| Epithelial | Epithelial | CLIC6 | 0.31 | | 1.13E-284 | |
| Epithelial | Epithelial | TSPAN8 | 0.73 | | 9.36E-272 | |
| Epithelial | Epithelial | IFI27 | 1.22 | | 1.52E-271 | |
| Epithelial | Epithelial | FOXQ1 | 0.69 | | 4.39E-271 | |
| Epithelial | Epithelial | HLA-B | -1.5 | | 3.58E-265 | |
| Epithelial | Epithelial | TESC | 0.98 | | 8.86E-265 | |
| Epithelial | Epithelial | HLA-C | -1.47 | | 1.92E-263 | |
| Epithelial | Epithelial | AKR7A3 | 1.03 | | 3.38E-263 | |
| Epithelial | Epithelial | FOXA2 | 0.3 | | 1.10E-259 | |
| Epithelial | Epithelial | SOSTDC1 | 0.27 | | 2.80E-256 | |
| Epithelial | Epithelial | GOLM1 | 0.91 | | 4.86E-256 | |
| Epithelial | Epithelial | GPRC5C | 0.53 | | 1.10E-255 | |
| Epithelial | Epithelial | SRGN | -2.78 | | 3.94E-255 | |
| Epithelial | Epithelial | CDC42EP1 | 0.74 | | 4.34E-255 | |
| Epithelial | Epithelial | MUCL3 | 0.67 | | 9.49E-255 | |
| Epithelial | Epithelial | SLC44A4 | 0.74 | | 6.68E-253 | |
| Epithelial | Epithelial | NQO1 | 1.02 | | 5.35E-251 | |
| Epithelial | Epithelial | COLCA1 | 0.46 | | 7.59E-251 | |
| Epithelial | Epithelial | MTRNR2L8 | 1.44 | | 3.75E-249 | |
| Epithelial | Epithelial | PDIA2 | 0.67 | | 3.05E-248 | |
| Epithelial | Epithelial | CST3 | 1.08 | | 5.44E-247 | |
| Epithelial | Epithelial | VSIG1 | 0.63 | | 9.24E-247 | |
| Epithelial | Epithelial | CLU | 0.73 | | 6.51E-240 | |
| Epithelial | Epithelial | SELENBP1 | 0.71 | | 6.03E-239 | |
| Epithelial | Epithelial | PRSS8 | 0.59 | | 1.36E-237 | |
| Epithelial | Epithelial | LINC00982 | 0.26 | | 9.62E-237 | |
| Epithelial | Epithelial | LGALS3BP | 0.96 | | 8.01E-236 | |
| Epithelial | Epithelial | TMC5 | 0.8 | | 3.29E-235 | |
| Epithelial | Epithelial | MTRNR2L2 | 1.93 | | 5.64E-235 | |
| Epithelial | Epithelial | SULT1C2 | 0.84 | | 7.59E-232 | |
| Epithelial | Epithelial | FMOD | 0.35 | | 3.47E-226 | |
| Epithelial | Epithelial | MGST1 | 0.72 | | 1.57E-225 | |
| Epithelial | Epithelial | BTG1 | -1.89 | | 4.48E-225 | |
| Epithelial | Epithelial | GKN1 | 3.35 | | 2.66E-222 | |
| Epithelial | Epithelial | AKR1C3 | 0.87 | | 2.04E-218 | |
| Epithelial | Epithelial | GALE | 0.7 | | 1.87E-216 | |
| Epithelial | Epithelial | EIF1 | -1 | | 8.86E-213 | |
| Epithelial | Epithelial | TSPAN1 | 0.76 | | 1.47E-211 | |
| Epithelial | Epithelial | PIK3C2G | 0.34 | | 6.59E-211 | |
| Epithelial | Epithelial | BLVRB | 0.98 | | 1.63E-209 | |
| Epithelial | Epithelial | MLPH | 0.66 | | 4.37E-209 | |
| Epithelial | Epithelial | MT-ND4 | 1.31 | | 1.65E-208 | |
| Epithelial | Epithelial | ATP1B1 | 0.9 | | 4.85E-208 | |
| Epithelial | Epithelial | HLA-A | -1.23 | | 4.40E-205 | |
| Epithelial | Epithelial | MT-ND4L | 1.41 | | 7.26E-202 | |
| Epithelial | Epithelial | ARHGDIB | -1.9 | | 2.17E-201 | |
| Epithelial | Epithelial | LIPH | 0.69 | | 3.32E-201 | |
| Epithelial | Epithelial | GIF | 1.49 | | 1.58E-200 | |
| Epithelial | Epithelial | MTRNR2L12 | 1.57 | | 1.95E-200 | |
| Epithelial | Epithelial | ERN2 | 0.63 | | 1.10E-198 | |
| Epithelial | Epithelial | HMGCS2 | 0.47 | | 3.23E-193 | |
| Epithelial | Epithelial | AKR1B10 | 0.99 | | 2.16E-192 | |
| Epithelial | Epithelial | FUT2 | 0.53 | | 5.79E-191 | |
| Epithelial | Epithelial | NPDC1 | 0.64 | | 9.85E-190 | |
| Epithelial | Epithelial | MT-ND3 | 1.45 | | 7.20E-189 | |
| Epithelial | Epithelial | MTRNR2L1 | 1.21 | | 1.33E-186 | |
| Epithelial | Epithelial | SARAF | -1.62 | | 4.17E-186 | |
| Epithelial | Epithelial | RPL10 | -0.8 | | 1.45E-185 | |
| Epithelial | Epithelial | TSC22D3 | -1.91 | | 6.64E-185 | |
| Epithelial | Epithelial | MUC4 | 0.55 | | 7.35E-185 | |
| Epithelial | Epithelial | HLA-E | -1.18 | | 1.53E-183 | |
| Epithelial | Epithelial | MT-ATP6 | 1.23 | | 9.60E-183 | |
| Epithelial | Epithelial | LYPD6B | 0.3 | | 3.38E-182 | |
| Epithelial | Epithelial | CREM | -2.1 | | 1.78E-180 | |
| Epithelial | Epithelial | MYRF | 0.4 | | 2.62E-180 | |
| Epithelial | Epithelial | MIA | 0.7 | | 1.89E-179 | |
| Epithelial | Epithelial | VIM | -2.36 | | 2.20E-179 | |
| Epithelial | Epithelial | FAU | -0.78 | | 4.68E-179 | |
| Epithelial | Epithelial | MT-CO3 | 1.08 | | 1.41E-175 | |
| Epithelial | Epithelial | MT-CYB | 1.16 | | 1.41E-175 | |
| Epithelial | Epithelial | MT-ND5 | 1.44 | | 5.14E-175 | |
| Epithelial | Epithelial | EPN3 | 0.28 | | 4.25E-172 | |
| Epithelial | Epithelial | CXCR4 | -2.35 | | 1.37E-170 | |
| Epithelial | Epithelial | AKR1C1 | 0.42 | | 1.72E-170 | |
| Epithelial | Epithelial | MT-CO1 | 1.2 | | 2.84E-170 | |
| Epithelial | Epithelial | CD44 | -1.59 | | 9.03E-170 | |
| Epithelial | Epithelial | MT-ND1 | 1.09 | | 6.60E-169 | |
| Epithelial | Epithelial | DMKN | 0.3 | | 5.32E-167 | |
| Epithelial | Epithelial | EEF1A1 | -0.85 | | 1.07E-166 | |
| Epithelial | Epithelial | MT-CO2 | 1.15 | | 1.33E-165 | |
| Epithelial | Epithelial | ID1 | 1.05 | | 1.56E-163 | |
| Epithelial | Epithelial | H3F3B | -1.02 | | 1.52E-162 | |
| Epithelial | Epithelial | CYB5A | 0.92 | | 1.64E-161 | |
| Epithelial | Epithelial | GATA6 | 0.39 | | 1.53E-160 | |
| Epithelial | Epithelial | XYLT2 | 0.28 | | 2.70E-159 | |
| Epithelial | Epithelial | ENAH | 0.31 | | 5.89E-159 | |
| Epithelial | Epithelial | OAZ1 | -0.94 | | 6.51E-159 | |
| Epithelial | Epithelial | LAPTM5 | -1.86 | | 2.91E-158 | |
| Epithelial | Epithelial | CKB | 1.65 | | 4.20E-158 | |
| Epithelial | Epithelial | C5orf38 | 0.3 | | 2.27E-157 | |
| Epithelial | Epithelial | ABCC3 | 0.63 | | 4.86E-156 | |
| Epithelial | Epithelial | MT-ATP8 | 0.84 | | 1.71E-155 | |
| Epithelial | Epithelial | ADIRF | 0.42 | | 2.82E-154 | |
| Epithelial | Epithelial | TCIM | 0.75 | | 1.78E-152 | |
| Epithelial | Epithelial | GMDS | 0.71 | | 3.88E-152 | |
| Epithelial | Epithelial | RPL21 | -0.76 | | 6.04E-152 | |
| Epithelial | Epithelial | ST6GALNAC1 | 0.41 | | 7.00E-152 | |
| Epithelial | Epithelial | TMEM98 | 0.46 | | 1.19E-151 | |
| Epithelial | Epithelial | GATM | 0.38 | | 5.57E-150 | |
| Epithelial | Epithelial | YPEL5 | -1.52 | | 6.13E-150 | |
| Epithelial | Epithelial | MT1X | 1.46 | | 1.45E-149 | |
| Epithelial | Epithelial | PTGR1 | 0.51 | | 4.25E-149 | |
| Epithelial | Epithelial | SDC4 | 0.81 | | 4.84E-148 | |
| Epithelial | Epithelial | PPDPF | 1.01 | | 3.54E-147 | |
| Epithelial | Epithelial | CD52 | -1.9 | | 1.73E-146 | |
| Epithelial | Epithelial | PDZK1IP1 | 0.53 | | 6.03E-145 | |
| Epithelial | Epithelial | SLC7A8 | 0.28 | | 1.49E-144 | |
| Epithelial | Epithelial | SLPI | 0.71 | | 1.60E-144 | |
| Epithelial | Epithelial | ATP5ME | 0.99 | | 2.84E-143 | |
| Epithelial | Epithelial | AADAC | 0.3 | | 5.18E-142 | |
| Epithelial | Epithelial | RGS5 | 0.35 | | 8.55E-140 | |
| Epithelial | Epithelial | BCAS1 | 0.44 | | 2.57E-139 | |
| Epithelial | Epithelial | CDC42EP5 | 0.5 | | 7.34E-138 | |
| Epithelial | Epithelial | TMEM54 | 0.58 | | 1.51E-137 | |
| Epithelial | Epithelial | DGKD | 0.4 | | 1.56E-135 | |
| Epithelial | Epithelial | EMP3 | -1.67 | | 2.03E-135 | |
| Epithelial | Epithelial | ATP4B | 1.41 | | 6.01E-135 | |
| Epithelial | Epithelial | RPS27A | -0.69 | | 1.44E-134 | |
| Epithelial | Epithelial | CXADR | 0.36 | | 6.24E-134 | |
| Epithelial | Epithelial | HES1 | 0.73 | | 4.09E-133 | |
| Epithelial | Epithelial | SOX9 | 0.5 | | 7.66E-132 | |
| Epithelial | Epithelial | CD37 | -1.55 | | 2.60E-131 | |
| Epithelial | Epithelial | KCNQ1 | 0.27 | | 2.23E-130 | |
| Epithelial | Epithelial | EPCAM | 0.46 | | 2.67E-130 | |
| Epithelial | Epithelial | PLXNB2 | 0.57 | | 1.08E-129 | |
| Epithelial | Epithelial | CREB3L1 | 0.33 | | 2.75E-129 | |
| Epithelial | Epithelial | RPS25 | -0.68 | | 3.54E-128 | |
| Epithelial | Epithelial | MT-ND2 | 0.98 | | 4.29E-126 | |
| Epithelial | Epithelial | ATP4A | 1.14 | | 6.56E-126 | |
| Epithelial | Epithelial | CYP3A5 | 0.64 | | 4.14E-125 | |
| Epithelial | Epithelial | DDX5 | -0.96 | | 8.64E-125 | |
| Epithelial | Epithelial | FTH1 | -1.53 | | 1.43E-124 | |
| Epithelial | Epithelial | KAZALD1 | 0.26 | | 1.63E-124 | |
| Epithelial | Epithelial | RPS15A | -0.66 | | 4.76E-124 | |
| Epithelial | Epithelial | MECOM | 0.36 | | 5.28E-124 | |
| Epithelial | Epithelial | LINC01133 | 0.67 | | 5.97E-124 | |
| Epithelial | Epithelial | MAL2 | 0.51 | | 6.51E-124 | |
| Epithelial | Epithelial | UBC | -0.87 | | 7.62E-123 | |
| Epithelial | Epithelial | RAB25 | 0.44 | | 1.02E-121 | |
| Epithelial | Epithelial | TCEAL9 | 0.6 | | 9.82E-121 | |
| Epithelial | Epithelial | CORO1A | -1.43 | | 1.16E-120 | |
| Epithelial | Epithelial | RPS20 | -0.65 | | 2.83E-120 | |
| Epithelial | Epithelial | RPS4X | -0.66 | | 3.51E-120 | |
| Epithelial | Epithelial | RPL30 | -0.68 | | 4.09E-120 | |
| Epithelial | Epithelial | SMIM6 | 0.3 | | 5.50E-120 | |
| Epithelial | Epithelial | CAPS | 0.44 | | 1.21E-119 | |
| Epithelial | Epithelial | ANKRD22 | 0.28 | | 1.33E-119 | |
| Epithelial | Epithelial | RPL9 | -0.64 | | 7.24E-119 | |
| Epithelial | Epithelial | PTPRCAP | -1.4 | | 1.93E-117 | |
| Epithelial | Epithelial | ERBB3 | 0.42 | | 3.65E-117 | |
| Epithelial | Epithelial | IMPA2 | 0.37 | | 2.02E-116 | |
| Epithelial | Epithelial | TRNP1 | 0.43 | | 2.24E-116 | |
| Epithelial | Epithelial | RGS1 | -1.94 | | 3.14E-116 | |
| Epithelial | Epithelial | RPS7 | -0.65 | | 3.54E-116 | |
| Epithelial | Epithelial | AC023090.1 | 0.27 | | 1.41E-115 | |
| Epithelial | Epithelial | GDF15 | 0.71 | | 3.38E-115 | |
| Epithelial | Epithelial | RPL4 | -0.75 | | 9.20E-115 | |
| Epithelial | Epithelial | SLC12A2 | 0.5 | | 6.09E-114 | |
| Epithelial | Epithelial | CALM1 | -0.93 | | 3.23E-113 | |
| Epithelial | Epithelial | HOOK2 | 0.56 | | 3.53E-113 | |
| Epithelial | Epithelial | HSPA1B | 0.91 | | 4.38E-113 | |
| Epithelial | Epithelial | H2AFJ | 0.75 | | 9.84E-113 | |
| Epithelial | Epithelial | GPRC5A | 0.69 | | 5.12E-112 | |
| Epithelial | Epithelial | RPLP1 | -0.57 | | 2.24E-110 | |
| Epithelial | Epithelial | SUB1 | -0.9 | | 3.82E-110 | |
| Epithelial | Epithelial | GALNT7 | 0.36 | | 3.82E-110 | |
| Epithelial | Epithelial | PDE4C | 0.55 | | 1.45E-109 | |
| Epithelial | Epithelial | RPSA | -0.77 | | 5.31E-109 | |
| Epithelial | Epithelial | GPR183 | -1.6 | | 6.45E-109 | |
| Epithelial | Epithelial | S100A4 | -1.99 | | 8.71E-109 | |
| Epithelial | Epithelial | NR2F2 | 0.26 | | 1.54E-108 | |
| Epithelial | Epithelial | LSP1 | -1.22 | | 1.94E-108 | |
| Epithelial | Epithelial | PNRC1 | -1.08 | | 1.59E-106 | |
| Epithelial | Epithelial | SH3BGRL2 | 0.34 | | 3.25E-106 | |
| Epithelial | Epithelial | KRTCAP3 | 0.38 | | 5.22E-106 | |
| Epithelial | Epithelial | RPL23A | -0.6 | | 5.92E-106 | |
| Epithelial | Epithelial | GALNT12 | 0.29 | | 7.61E-106 | |
| Epithelial | Epithelial | RFLNA | 0.3 | | 8.08E-106 | |
| Epithelial | Epithelial | HSPA1A | 0.96 | | 2.29E-105 | |
| Epithelial | Epithelial | FOXA3 | 0.3 | | 2.42E-105 | |
| Epithelial | Epithelial | SELENOK | -0.99 | | 2.61E-105 | |
| Epithelial | Epithelial | CD53 | -1.17 | | 4.79E-105 | |
| Epithelial | Epithelial | RPL3 | -0.59 | | 5.74E-105 | |
| Epithelial | Epithelial | ANKRD36C | 1.1 | | 7.54E-105 | |
| Epithelial | Epithelial | RPL15 | -0.55 | | 7.89E-105 | |
| Epithelial | Epithelial | C6orf132 | 0.36 | | 1.75E-104 | |
| Epithelial | Epithelial | PTPRC | -1.29 | | 3.97E-104 | |
| Epithelial | Epithelial | LSR | 0.59 | | 6.63E-104 | |
| Epithelial | Epithelial | NUPR1 | 0.56 | | 9.31E-104 | |
| Epithelial | Epithelial | MCL1 | -1.06 | | 4.15E-103 | |
| Epithelial | Epithelial | CTNND1 | 0.39 | | 1.09E-102 | |
| Epithelial | Epithelial | RPL5 | -0.67 | | 2.72E-102 | |
| Epithelial | Epithelial | PLPP2 | 0.43 | | 3.03E-102 | |
| Epithelial | Epithelial | H1F0 | 0.36 | | 2.93E-101 | |
| Epithelial | Epithelial | TPT1 | -0.57 | | 3.52E-101 | |
| Epithelial | Epithelial | MT1F | 0.76 | | 5.03E-101 | |
| Epithelial | Epithelial | PABPC1 | -0.81 | | 5.03E-101 | |
| Epithelial | Epithelial | IL2RG | -1.09 | | 7.23E-101 | |
| Epithelial | Epithelial | SERINC2 | 0.38 | | 2.20E-99 | |
| Epithelial | Epithelial | AC020656.1 | 0.93 | | 4.10E-99 | |
| Epithelial | Epithelial | GMFG | -1.1 | | 8.59E-99 | |
| Epithelial | Epithelial | RPS16 | -0.54 | | 1.01E-98 | |
| Epithelial | Epithelial | LDHA | -1.18 | | 2.74E-98 | |
| Epithelial | Epithelial | ATP8B1 | 0.36 | | 1.02E-97 | |
| Epithelial | Epithelial | RASSF7 | 0.49 | | 1.34E-97 | |
| Epithelial | Epithelial | MTRNR2L6 | 0.35 | | 5.17E-97 | |
| Epithelial | Epithelial | TPM1 | 0.44 | | 5.85E-97 | |
| Epithelial | Epithelial | LINC02381 | 0.27 | | 6.11E-97 | |
| Epithelial | Epithelial | RGCC | -1.63 | | 8.52E-97 | |
| Epithelial | Epithelial | TSPO | 0.74 | | 2.85E-96 | |
| Epithelial | Epithelial | RPL6 | -0.57 | | 2.87E-96 | |
| Epithelial | Epithelial | TMEM97 | 0.4 | | 7.44E-96 | |
| Epithelial | Epithelial | UBA52 | -0.53 | | 3.94E-95 | |
| Epithelial | Epithelial | CAPN8 | 0.5 | | 4.38E-95 | |
| Epithelial | Epithelial | ARL14 | 0.57 | | 6.93E-95 | |
| Epithelial | Epithelial | MFSD4A | 0.3 | | 8.21E-95 | |
| Epithelial | Epithelial | RPS27 | -0.67 | | 1.39E-94 | |
| Epithelial | Epithelial | CDH1 | 0.37 | | 1.46E-94 | |
| Epithelial | Epithelial | SAMSN1 | -1.22 | | 1.79E-94 | |
| Epithelial | Epithelial | NAP1L1 | -0.94 | | 4.42E-94 | |
| Epithelial | Epithelial | FXYD5 | -1.02 | | 4.09E-93 | |
| Epithelial | Epithelial | ANXA3 | 0.34 | | 2.01E-92 | |
| Epithelial | Epithelial | AAMDC | 0.48 | | 3.54E-92 | |
| Epithelial | Epithelial | TST | 0.45 | | 5.67E-92 | |
| Epithelial | Epithelial | SH3BGRL3 | -0.96 | | 7.29E-92 | |
| Epithelial | Epithelial | NDUFB7 | 0.74 | | 7.91E-92 | |
| Epithelial | Epithelial | DUOX2 | 0.27 | | 1.93E-91 | |
| Epithelial | Epithelial | CFL1 | -0.72 | | 2.98E-91 | |
| Epithelial | Epithelial | CDCA7 | 0.31 | | 4.48E-91 | |
| Epithelial | Epithelial | RPS23 | -0.57 | | 4.64E-91 | |
| Epithelial | Epithelial | HNRNPA1 | -0.66 | | 6.63E-91 | |
| Epithelial | Epithelial | NR2F6 | 0.32 | | 1.03E-90 | |
| Epithelial | Epithelial | DSP | 0.35 | | 1.04E-90 | |
| Epithelial | Epithelial | SPINT1 | 0.42 | | 2.94E-90 | |
| Epithelial | Epithelial | KLK11 | 0.28 | | 6.21E-90 | |
| Epithelial | Epithelial | UQCRQ | 0.79 | | 2.56E-89 | |
| Epithelial | Epithelial | RRBP1 | 0.74 | | 3.09E-89 | |
| Epithelial | Epithelial | CYTIP | -1.21 | | 6.12E-89 | |
| Epithelial | Epithelial | CD48 | -1.05 | | 1.02E-88 | |
| Epithelial | Epithelial | S100A14 | 0.44 | | 1.17E-88 | |
| Epithelial | Epithelial | RND3 | 0.56 | | 4.16E-88 | |
| Epithelial | Epithelial | LLGL2 | 0.5 | | 4.20E-88 | |
| Epithelial | Epithelial | TMSB4X | -0.77 | | 8.58E-88 | |
| Epithelial | Epithelial | CD69 | -1.68 | | 1.27E-87 | |
| Epithelial | Epithelial | COL27A1 | 0.33 | | 4.08E-87 | |
| Epithelial | Epithelial | EGR1 | 0.65 | | 8.78E-87 | |
| Epithelial | Epithelial | BACE2 | 0.4 | | 1.05E-86 | |
| Epithelial | Epithelial | CYBA | -0.85 | | 1.44E-86 | |
| Epithelial | Epithelial | COMTD1 | 0.56 | | 6.93E-86 | |
| Epithelial | Epithelial | DHCR24 | 0.29 | | 8.30E-86 | |
| Epithelial | Epithelial | TACSTD2 | 0.55 | | 1.43E-85 | |
| Epithelial | Epithelial | ISG20 | -1.1 | | 3.54E-85 | |
| Epithelial | Epithelial | ATP5IF1 | 0.7 | | 4.41E-85 | |
| Epithelial | Epithelial | TUBA1A | -1.19 | | 1.40E-84 | |
| Epithelial | Epithelial | ATP1B3 | -1.2 | | 1.92E-84 | |
| Epithelial | Epithelial | PLLP | 0.26 | | 3.51E-84 | |
| Epithelial | Epithelial | ACTB | -0.87 | | 5.05E-84 | |
| Epithelial | Epithelial | SRSF2 | -0.87 | | 7.05E-84 | |
| Epithelial | Epithelial | CRACR2B | 0.44 | | 9.31E-84 | |
| Epithelial | Epithelial | ELOB | 0.72 | | 1.37E-83 | |
| Epithelial | Epithelial | LAMB3 | 0.57 | | 1.38E-83 | |
| Epithelial | Epithelial | COX5B | 0.77 | | 3.68E-83 | |
| Epithelial | Epithelial | CD151 | 0.54 | | 1.37E-82 | |
| Epithelial | Epithelial | SPINT2 | 0.56 | | 2.39E-82 | |
| Epithelial | Epithelial | PTMA | -0.55 | | 5.45E-82 | |
| Epithelial | Epithelial | RPS3A | -0.55 | | 6.18E-82 | |
| Epithelial | Epithelial | MAGI1 | 0.26 | | 3.25E-81 | |
| Epithelial | Epithelial | SPINT1-AS1 | 0.32 | | 4.04E-81 | |
| Epithelial | Epithelial | ARPC2 | -0.82 | | 7.72E-81 | |
| Epithelial | Epithelial | CRIP1 | -1.32 | | 1.06E-80 | |
| Epithelial | Epithelial | LGALS1 | -1.96 | | 1.50E-80 | |
| Epithelial | Epithelial | RPL7 | -0.48 | | 1.70E-80 | |
| Epithelial | Epithelial | TSTA3 | 0.6 | | 2.24E-80 | |
| Epithelial | Epithelial | SOX4 | 0.83 | | 8.03E-80 | |
| Epithelial | Epithelial | SFN | 0.47 | | 1.17E-78 | |
| Epithelial | Epithelial | KLF5 | 0.46 | | 2.09E-78 | |
| Epithelial | Epithelial | RBM47 | 0.45 | | 3.21E-78 | |
| Epithelial | Epithelial | ANG | 0.29 | | 3.65E-78 | |
| Epithelial | Epithelial | CALM2 | -0.71 | | 5.58E-78 | |
| Epithelial | Epithelial | BICDL2 | 0.35 | | 8.99E-78 | |
| Epithelial | Epithelial | NR0B2 | 0.37 | | 1.51E-77 | |
| Epithelial | Epithelial | HADH | 0.42 | | 1.78E-77 | |
| Epithelial | Epithelial | FERMT1 | 0.26 | | 1.94E-76 | |
| Epithelial | Epithelial | HCST | -1.24 | | 2.19E-76 | |
| Epithelial | Epithelial | EDN1 | 0.4 | | 3.48E-76 | |
| Epithelial | Epithelial | RPL19 | -0.47 | | 5.15E-76 | |
| Epithelial | Epithelial | IDS | -0.96 | | 5.37E-76 | |
| Epithelial | Epithelial | SRSF5 | -0.83 | | 8.13E-76 | |
| Epithelial | Epithelial | EHF | 0.27 | | 1.12E-75 | |
| Epithelial | Epithelial | IL32 | -1.5 | | 1.34E-74 | |
| Epithelial | Epithelial | JUP | 0.37 | | 8.30E-74 | |
| Epithelial | Epithelial | METTL7A | 0.4 | | 1.67E-73 | |
| Epithelial | Epithelial | SLC2A3 | -1.15 | | 1.93E-73 | |
| Epithelial | Epithelial | UBE2D3 | -0.73 | | 1.96E-73 | |
| Epithelial | Epithelial | RPLP2 | -0.53 | | 3.36E-73 | |
| Epithelial | Epithelial | RPS6 | -0.48 | | 9.08E-73 | |
| Epithelial | Epithelial | IDH1 | 0.36 | | 9.56E-73 | |
| Epithelial | Epithelial | PFDN5 | -0.54 | | 1.25E-72 | |
| Epithelial | Epithelial | TSPAN15 | 0.33 | | 2.13E-72 | |
| Epithelial | Epithelial | CHCHD10 | 0.72 | | 4.36E-72 | |
| Epithelial | Epithelial | TMC4 | 0.31 | | 1.54E-71 | |
| Epithelial | Epithelial | RHOH | -1.03 | | 3.71E-71 | |
| Epithelial | Epithelial | ANXA1 | -1.69 | | 4.70E-71 | |
| Epithelial | Epithelial | CCND1 | 0.42 | | 6.72E-71 | |
| Epithelial | Epithelial | RPL27 | -0.49 | | 7.47E-71 | |
| Epithelial | Epithelial | BMP2 | 0.27 | | 1.20E-70 | |
| Epithelial | Epithelial | BTF3 | -0.51 | | 2.56E-70 | |
| Epithelial | Epithelial | AC016831.4 | -0.97 | | 4.39E-70 | |
| Epithelial | Epithelial | GPSM3 | -0.85 | | 3.07E-69 | |
| Epithelial | Epithelial | GLUL | 0.62 | | 3.71E-69 | |
| Epithelial | Epithelial | AP1M2 | 0.29 | | 7.99E-69 | |
| Epithelial | Epithelial | H3F3A | -0.58 | | 1.13E-68 | |
| Epithelial | Epithelial | MGST2 | 0.42 | | 1.42E-68 | |
| Epithelial | Epithelial | RPS5 | -0.48 | | 2.35E-68 | |
| Epithelial | Epithelial | HID1 | 0.34 | | 2.72E-68 | |
| Epithelial | Epithelial | GYPC | -0.83 | | 3.45E-68 | |
| Epithelial | Epithelial | CD3D | -1.59 | | 5.96E-68 | |
| Epithelial | Epithelial | MIR4458HG | 0.3 | | 9.72E-68 | |
| Epithelial | Epithelial | PXMP2 | 0.41 | | 3.16E-67 | |
| Epithelial | Epithelial | BAIAP2L1 | 0.34 | | 5.35E-67 | |
| Epithelial | Epithelial | EFNA1 | 0.41 | | 6.57E-67 | |
| Epithelial | Epithelial | GALNT3 | 0.31 | | 1.10E-66 | |
| Epithelial | Epithelial | PRDX5 | 0.73 | | 1.38E-66 | |
| Epithelial | Epithelial | HNRNPA0 | -0.72 | | 3.89E-66 | |
| Epithelial | Epithelial | ATP5F1D | 0.65 | | 8.49E-66 | |
| Epithelial | Epithelial | PPP1R16A | 0.34 | | 8.54E-66 | |
| Epithelial | Epithelial | CXCL3 | 0.45 | | 8.97E-66 | |
| Epithelial | Epithelial | IFITM2 | -1.12 | | 9.15E-66 | |
| Epithelial | Epithelial | LMO7 | 0.43 | | 9.50E-66 | |
| Epithelial | Epithelial | ATF3 | 0.76 | | 2.41E-65 | |
| Epithelial | Epithelial | PGK1 | -0.83 | | 2.68E-65 | |
| Epithelial | Epithelial | MRPL52 | 0.62 | | 2.90E-65 | |
| Epithelial | Epithelial | COA3 | 0.52 | | 3.56E-65 | |
| Epithelial | Epithelial | NACA | -0.46 | | 6.21E-65 | |
| Epithelial | Epithelial | TM9SF3 | 0.5 | | 1.04E-64 | |
| Epithelial | Epithelial | DDAH1 | 0.29 | | 1.59E-64 | |
| Epithelial | Epithelial | EZR | -0.85 | | 2.89E-64 | |
| Epithelial | Epithelial | DDR1 | 0.28 | | 4.43E-64 | |
| Epithelial | Epithelial | LITAF | -1.07 | | 4.95E-64 | |
| Epithelial | Epithelial | MGAT4B | 0.35 | | 1.93E-63 | |
| Epithelial | Epithelial | ADAM15 | 0.38 | | 2.27E-63 | |
| Epithelial | Epithelial | WIPF1 | -0.81 | | 4.78E-63 | |
| Epithelial | Epithelial | NDUFB1 | 0.65 | | 6.43E-63 | |
| Epithelial | Epithelial | ARID5A | -0.83 | | 1.07E-62 | |
| Epithelial | Epithelial | RPS3 | -0.44 | | 1.84E-62 | |
| Epithelial | Epithelial | STK4 | -0.84 | | 1.87E-62 | |
| Epithelial | Epithelial | PKP3 | 0.36 | | 1.95E-62 | |
| Epithelial | Epithelial | COX6B1 | 0.6 | | 2.89E-62 | |
| Epithelial | Epithelial | LIMD2 | -0.89 | | 3.13E-62 | |
| Epithelial | Epithelial | BEX3 | 0.38 | | 3.19E-62 | |
| Epithelial | Epithelial | PRR4 | 1.27 | | 7.25E-62 | |
| Epithelial | Epithelial | LEPROTL1 | -1.11 | | 1.28E-61 | |
| Epithelial | Epithelial | EVI2B | -0.86 | | 1.55E-61 | |
| Epithelial | Epithelial | CD7 | -1.54 | | 1.95E-61 | |
| Epithelial | Epithelial | TGFB1 | -0.91 | | 4.15E-61 | |
| Epithelial | Epithelial | GLIPR1 | -0.85 | | 4.50E-61 | |
| Epithelial | Epithelial | PNKD | 0.47 | | 7.22E-61 | |
| Epithelial | Epithelial | KLF6 | -0.68 | | 7.56E-61 | |
| Epithelial | Epithelial | GALNT6 | 0.4 | | 1.07E-60 | |
| Epithelial | Epithelial | HNRNPDL | -0.66 | | 2.28E-60 | |
| Epithelial | Epithelial | LGALS4 | 0.26 | | 5.01E-60 | |
| Epithelial | Epithelial | CD3E | -1.18 | | 5.24E-60 | |
| Epithelial | Epithelial | RPL36AL | -0.54 | | 6.06E-60 | |
| Epithelial | Epithelial | STK17B | -0.89 | | 1.79E-59 | |
| Epithelial | Epithelial | ARPC1B | -0.86 | | 4.39E-59 | |
| Epithelial | Epithelial | UQCC2 | 0.48 | | 4.61E-59 | |
| Epithelial | Epithelial | ELF1 | -0.85 | | 5.53E-59 | |
| Epithelial | Epithelial | GSTP1 | 0.67 | | 5.65E-59 | |
| Epithelial | Epithelial | LDLR | 0.43 | | 7.93E-59 | |
| Epithelial | Epithelial | RAB27B | 0.3 | | 3.16E-58 | |
| Epithelial | Epithelial | EIF4A1 | -0.59 | | 3.97E-58 | |
| Epithelial | Epithelial | MT1H | 0.42 | | 4.26E-58 | |
| Epithelial | Epithelial | RBM8A | -0.7 | | 5.00E-58 | |
| Epithelial | Epithelial | KCNE3 | 0.3 | | 5.42E-58 | |
| Epithelial | Epithelial | CYP2S1 | 0.35 | | 1.41E-57 | |
| Epithelial | Epithelial | CCDC34 | 0.25 | | 1.76E-57 | |
| Epithelial | Epithelial | NR3C1 | -0.94 | | 1.96E-57 | |
| Epithelial | Epithelial | CENPX | 0.53 | | 2.73E-57 | |
| Epithelial | Epithelial | S100A6 | 0.63 | | 2.98E-57 | |
| Epithelial | Epithelial | RAC2 | -0.88 | | 3.92E-57 | |
| Epithelial | Epithelial | UQCC3 | 0.38 | | 6.91E-57 | |
| Epithelial | Epithelial | EEF1B2 | -0.53 | | 7.39E-57 | |
| Epithelial | Epithelial | HLA-DPA1 | -1.54 | | 1.19E-56 | |
| Epithelial | Epithelial | RPL24 | -0.41 | | 1.54E-56 | |
| Epithelial | Epithelial | CCNI | -0.63 | | 2.03E-56 | |
| Epithelial | Epithelial | ASPH | 0.31 | | 2.49E-56 | |
| Epithelial | Epithelial | TNFAIP3 | -1.02 | | 3.44E-56 | |
| Epithelial | Epithelial | CNBP | -0.63 | | 5.33E-56 | |
| Epithelial | Epithelial | RPS13 | -0.42 | | 8.52E-56 | |
| Epithelial | Epithelial | RPL34 | -0.4 | | 1.09E-55 | |
| Epithelial | Epithelial | CLEC2B | -0.93 | | 4.02E-55 | |
| Epithelial | Epithelial | NR4A2 | -1.11 | | 4.51E-55 | |
| Epithelial | Epithelial | ASL | 0.41 | | 4.99E-55 | |
| Epithelial | Epithelial | NDUFV3 | 0.41 | | 1.25E-54 | |
| Epithelial | Epithelial | SPOCK2 | -1 | | 1.39E-54 | |
| Epithelial | Epithelial | NPM1 | -0.47 | | 1.44E-54 | |
| Epithelial | Epithelial | MSN | -0.74 | | 2.15E-54 | |
| Epithelial | Epithelial | LTB | -1.21 | | 2.31E-54 | |
| Epithelial | Epithelial | TIMM13 | 0.54 | | 2.73E-54 | |
| Epithelial | Epithelial | PDE4B | -0.78 | | 2.97E-54 | |
| Epithelial | Epithelial | RF00598 | 0.79 | | 3.00E-54 | |
| Epithelial | Epithelial | ARPC3 | -0.6 | | 3.57E-54 | |
| Epithelial | Epithelial | CTTN | 0.31 | | 3.91E-54 | |
| Epithelial | Epithelial | CIRBP | -0.56 | | 4.30E-54 | |
| Epithelial | Epithelial | NAPRT | 0.42 | | 4.79E-54 | |
| Epithelial | Epithelial | CD79A | -1.87 | | 5.02E-54 | |
| Epithelial | Epithelial | RPL32 | -0.39 | | 7.00E-54 | |
| Epithelial | Epithelial | REL | -0.93 | | 1.06E-53 | |
| Epithelial | Epithelial | ARHGEF16 | 0.27 | | 1.07E-53 | |
| Epithelial | Epithelial | HPGD | 0.35 | | 1.62E-53 | |
| Epithelial | Epithelial | ANXA4 | 0.34 | | 1.62E-53 | |
| Epithelial | Epithelial | TMEM141 | 0.44 | | 1.64E-53 | |
| Epithelial | Epithelial | DUSP1 | -0.72 | | 2.18E-53 | |
| Epithelial | Epithelial | DNAJB6 | -0.73 | | 3.32E-53 | |
| Epithelial | Epithelial | SKP1 | -0.54 | | 6.37E-53 | |
| Epithelial | Epithelial | NEDD4L | 0.26 | | 7.95E-53 | |
| Epithelial | Epithelial | ROMO1 | 0.64 | | 9.82E-53 | |
| Epithelial | Epithelial | ADGRE5 | -0.85 | | 1.06E-52 | |
| Epithelial | Epithelial | CD2 | -1.16 | | 1.20E-52 | |
| Epithelial | Epithelial | EIF3E | -0.55 | | 1.25E-52 | |
| Epithelial | Epithelial | IER3 | 0.53 | | 1.33E-52 | |
| Epithelial | Epithelial | FNBP1 | -0.77 | | 1.70E-52 | |
| Epithelial | Epithelial | ALDH2 | 0.49 | | 2.56E-52 | |
| Epithelial | Epithelial | YBX1 | -0.5 | | 3.33E-52 | |
| Epithelial | Epithelial | RHOG | -0.77 | | 4.08E-52 | |
| Epithelial | Epithelial | RAP1B | -0.69 | | 7.23E-52 | |
| Epithelial | Epithelial | FGD4 | 0.26 | | 7.75E-52 | |
| Epithelial | Epithelial | RPL39 | -0.4 | | 8.35E-52 | |
| Epithelial | Epithelial | MICALL2 | 0.3 | | 1.45E-51 | |
| Epithelial | Epithelial | SH3YL1 | 0.35 | | 1.99E-51 | |
| Epithelial | Epithelial | B4GALNT3 | 0.27 | | 1.99E-51 | |
| Epithelial | Epithelial | MYADM | -0.93 | | 3.29E-51 | |
| Epithelial | Epithelial | PAIP2 | -0.66 | | 3.44E-51 | |
| Epithelial | Epithelial | RPS14 | -0.36 | | 1.27E-50 | |
| Epithelial | Epithelial | RPL28 | -0.33 | | 1.38E-50 | |
| Epithelial | Epithelial | SNHG19 | 0.35 | | 1.47E-50 | |
| Epithelial | Epithelial | 7-Sep | -0.75 | | 4.05E-50 | |
| Epithelial | Epithelial | NOP53 | -0.54 | | 4.57E-50 | |
| Epithelial | Epithelial | PTPRK | 0.27 | | 5.03E-50 | |
| Epithelial | Epithelial | CRNDE | 0.26 | | 7.80E-50 | |
| Epithelial | Epithelial | CBR1 | 0.42 | | 8.46E-50 | |
| Epithelial | Epithelial | MALAT1 | -0.35 | | 1.04E-49 | |
| Epithelial | Epithelial | EPS8L2 | 0.33 | | 1.28E-49 | |
| Epithelial | Epithelial | SHROOM3 | 0.25 | | 2.38E-49 | |
| Epithelial | Epithelial | FUT8 | 0.25 | | 2.82E-49 | |
| Epithelial | Epithelial | ZFP36L2 | -0.88 | | 2.87E-49 | |
| Epithelial | Epithelial | RGS2 | -1.09 | | 5.09E-49 | |
| Epithelial | Epithelial | ALOX5AP | -1.02 | | 5.74E-49 | |
| Epithelial | Epithelial | GNG2 | -0.8 | | 9.98E-49 | |
| Epithelial | Epithelial | CYTOR | -0.95 | | 1.22E-48 | |
| Epithelial | Epithelial | ARID5B | -0.85 | | 1.60E-48 | |
| Epithelial | Epithelial | SERPINB9 | -0.82 | | 2.77E-48 | |
| Epithelial | Epithelial | ITM2B | -0.67 | | 5.09E-48 | |
| Epithelial | Epithelial | TSC22D1 | 0.25 | | 7.07E-48 | |
| Epithelial | Epithelial | KLF4 | 0.43 | | 7.44E-48 | |
| Epithelial | Epithelial | SLIRP | 0.52 | | 8.96E-48 | |
| Epithelial | Epithelial | EPS8L1 | 0.31 | | 9.50E-48 | |
| Epithelial | Epithelial | SELENOT | -0.66 | | 1.06E-47 | |
| Epithelial | Epithelial | RPL10A | -0.38 | | 1.42E-47 | |
| Epithelial | Epithelial | ACADVL | 0.53 | | 1.65E-47 | |
| Epithelial | Epithelial | ICAM3 | -0.71 | | 2.61E-47 | |
| Epithelial | Epithelial | FMNL1 | -0.67 | | 2.75E-47 | |
| Epithelial | Epithelial | TAGAP | -0.77 | | 3.34E-47 | |
| Epithelial | Epithelial | FAM129B | 0.3 | | 6.89E-47 | |
| Epithelial | Epithelial | EIF3H | -0.58 | | 1.55E-46 | |
| Epithelial | Epithelial | CST7 | -1.29 | | 1.67E-46 | |
| Epithelial | Epithelial | AK1 | 0.25 | | 1.94E-46 | |
| Epithelial | Epithelial | PPP2R5C | -0.84 | | 2.03E-46 | |
| Epithelial | Epithelial | ERBB2 | 0.27 | | 4.02E-46 | |
| Epithelial | Epithelial | LCP1 | -0.73 | | 4.46E-46 | |
| Epithelial | Epithelial | TENT5A | 0.41 | | 6.35E-46 | |
| Epithelial | Epithelial | PDK4 | 0.31 | | 6.84E-46 | |
| Epithelial | Epithelial | CD55 | -0.74 | | 9.01E-46 | |
| Epithelial | Epithelial | ETFB | 0.47 | | 9.34E-46 | |
| Epithelial | Epithelial | CCL5 | -2.18 | | 1.09E-45 | |
| Epithelial | Epithelial | BAIAP2 | 0.31 | | 2.58E-45 | |
| Epithelial | Epithelial | PYCR1 | 0.3 | | 2.96E-45 | |
| Epithelial | Epithelial | SLC9A1 | 0.28 | | 7.04E-45 | |
| Epithelial | Epithelial | BRI3 | 0.43 | | 7.23E-45 | |
| Epithelial | Epithelial | HSBP1L1 | 0.27 | | 1.29E-44 | |
| Epithelial | Epithelial | VILL | 0.35 | | 1.95E-44 | |
| Epithelial | Epithelial | PRNP | -0.71 | | 6.97E-44 | |
| Epithelial | Epithelial | HSPA6 | 0.48 | | 7.36E-44 | |
| Epithelial | Epithelial | PLA2G10 | 0.34 | | 8.01E-44 | |
| Epithelial | Epithelial | RPL13A | -0.32 | | 8.51E-44 | |
| Epithelial | Epithelial | ACAP1 | -0.67 | | 9.64E-44 | |
| Epithelial | Epithelial | RPS2 | -0.29 | | 1.06E-43 | |
| Epithelial | Epithelial | CD99 | -0.69 | | 1.09E-43 | |
| Epithelial | Epithelial | UQCR10 | 0.64 | | 1.40E-43 | |
| Epithelial | Epithelial | ECI1 | 0.34 | | 1.45E-43 | |
| Epithelial | Epithelial | IFITM1 | -0.96 | | 1.72E-43 | |
| Epithelial | Epithelial | SUMO2 | -0.48 | | 1.78E-43 | |
| Epithelial | Epithelial | VEGFA | 0.26 | | 3.62E-43 | |
| Epithelial | Epithelial | ABHD2 | 0.3 | | 4.42E-43 | |
| Epithelial | Epithelial | QSOX1 | 0.34 | | 5.38E-43 | |
| Epithelial | Epithelial | CDC42SE2 | -0.76 | | 1.60E-42 | |
| Epithelial | Epithelial | ADAM28 | 0.41 | | 2.14E-42 | |
| Epithelial | Epithelial | PCBD1 | 0.37 | | 2.45E-42 | |
| Epithelial | Epithelial | FAM177A1 | -0.83 | | 2.58E-42 | |
| Epithelial | Epithelial | ZNF331 | -0.96 | | 2.95E-42 | |
| Epithelial | Epithelial | PPP1CB | -0.76 | | 3.46E-42 | |
| Epithelial | Epithelial | ARHGDIA | -0.58 | | 4.01E-42 | |
| Epithelial | Epithelial | RPL35A | -0.31 | | 6.78E-42 | |
| Epithelial | Epithelial | PARD6B | 0.26 | | 8.15E-42 | |
| Epithelial | Epithelial | TXNDC17 | 0.49 | | 8.97E-42 | |
| Epithelial | Epithelial | FBP1 | 0.3 | | 1.11E-41 | |
| Epithelial | Epithelial | MORF4L1 | -0.56 | | 1.12E-41 | |
| Epithelial | Epithelial | PPP1R2 | -0.67 | | 1.17E-41 | |
| Epithelial | Epithelial | UBE2B | -0.62 | | 1.18E-41 | |
| Epithelial | Epithelial | KMT2E | -0.65 | | 2.10E-41 | |
| Epithelial | Epithelial | TMEM50A | -0.65 | | 2.17E-41 | |
| Epithelial | Epithelial | MPST | 0.37 | | 2.47E-41 | |
| Epithelial | Epithelial | HNRNPK | -0.53 | | 4.09E-41 | |
| Epithelial | Epithelial | ECHS1 | 0.41 | | 4.24E-41 | |
| Epithelial | Epithelial | RPS29 | -0.48 | | 5.64E-41 | |
| Epithelial | Epithelial | ZFP36 | -0.76 | | 1.00E-40 | |
| Epithelial | Epithelial | FYN | -0.87 | | 1.06E-40 | |
| Epithelial | Epithelial | PSME1 | -0.51 | | 1.82E-40 | |
| Epithelial | Epithelial | NDUFB3 | 0.46 | | 2.08E-40 | |
| Epithelial | Epithelial | ATP6V0E1 | -0.52 | | 2.33E-40 | |
| Epithelial | Epithelial | EPHA2 | 0.34 | | 3.55E-40 | |
| Epithelial | Epithelial | AC058791.1 | -0.71 | | 5.97E-40 | |
| Epithelial | Epithelial | GSTM3 | 0.3 | | 6.01E-40 | |
| Epithelial | Epithelial | HCLS1 | -0.54 | | 8.52E-40 | |
| Epithelial | Epithelial | EVI2A | -0.63 | | 1.24E-39 | |
| Epithelial | Epithelial | CCR7 | -0.86 | | 1.25E-39 | |
| Epithelial | Epithelial | CDC42 | -0.57 | | 1.41E-39 | |
| Epithelial | Epithelial | S100A13 | 0.28 | | 1.49E-39 | |
| Epithelial | Epithelial | PHLDA2 | 0.43 | | 1.72E-39 | |
| Epithelial | Epithelial | BST2 | -0.6 | | 2.31E-39 | |
| Epithelial | Epithelial | SCOC | 0.33 | | 2.50E-39 | |
| Epithelial | Epithelial | TNFRSF1B | -0.69 | | 2.60E-39 | |
| Epithelial | Epithelial | RBM3 | -0.5 | | 6.34E-39 | |
| Epithelial | Epithelial | 6-Sep | -0.6 | | 7.71E-39 | |
| Epithelial | Epithelial | FLNA | -0.63 | | 1.33E-38 | |
| Epithelial | Epithelial | NDUFA1 | 0.5 | | 1.46E-38 | |
| Epithelial | Epithelial | LIMA1 | 0.34 | | 1.50E-38 | |
| Epithelial | Epithelial | SERP1 | -0.52 | | 2.53E-38 | |
| Epithelial | Epithelial | HLA-DPB1 | -1.33 | | 3.55E-38 | |
| Epithelial | Epithelial | RILPL2 | -0.75 | | 5.03E-38 | |
| Epithelial | Epithelial | EIF3F | -0.48 | | 6.27E-38 | |
| Epithelial | Epithelial | RPL36 | 0.38 | | 7.32E-38 | |
| Epithelial | Epithelial | NDUFS6 | 0.46 | | 1.02E-37 | |
| Epithelial | Epithelial | SPATS2L | 0.4 | | 1.88E-37 | |
| Epithelial | Epithelial | RACK1 | -0.32 | | 2.00E-37 | |
| Epithelial | Epithelial | KRT7 | 0.39 | | 3.29E-37 | |
| Epithelial | Epithelial | TRIR | -0.49 | | 3.42E-37 | |
| Epithelial | Epithelial | RPL14 | -0.32 | | 3.77E-37 | |
| Epithelial | Epithelial | RPS8 | -0.34 | | 6.73E-37 | |
| Epithelial | Epithelial | CD83 | -1.13 | | 8.18E-37 | |
| Epithelial | Epithelial | FHL2 | 0.27 | | 1.17E-36 | |
| Epithelial | Epithelial | IL7R | -0.95 | | 1.33E-36 | |
| Epithelial | Epithelial | ZKSCAN1 | 0.3 | | 1.82E-36 | |
| Epithelial | Epithelial | YWHAZ | -0.49 | | 2.28E-36 | |
| Epithelial | Epithelial | RND1 | 0.3 | | 2.35E-36 | |
| Epithelial | Epithelial | HBEGF | 0.26 | | 2.54E-36 | |
| Epithelial | Epithelial | DMAC1 | 0.35 | | 2.93E-36 | |
| Epithelial | Epithelial | MAP1LC3B | -0.57 | | 3.81E-36 | |
| Epithelial | Epithelial | LDLRAD4 | -0.78 | | 4.25E-36 | |
| Epithelial | Epithelial | ANKRD12 | -0.66 | | 5.14E-36 | |
| Epithelial | Epithelial | ELOVL5 | -0.54 | | 5.17E-36 | |
| Epithelial | Epithelial | RPL27A | -0.29 | | 5.24E-36 | |
| Epithelial | Epithelial | TAX1BP3 | 0.32 | | 5.26E-36 | |
| Epithelial | Epithelial | NKG7 | -1.6 | | 8.35E-36 | |
| Epithelial | Epithelial | RAB13 | 0.29 | | 1.46E-35 | |
| Epithelial | Epithelial | EIF3L | -0.53 | | 1.60E-35 | |
| Epithelial | Epithelial | MRPL27 | 0.41 | | 2.52E-35 | |
| Epithelial | Epithelial | NEU1 | -0.61 | | 6.91E-35 | |
| Epithelial | Epithelial | TUBA1B | -0.59 | | 1.21E-34 | |
| Epithelial | Epithelial | TERF2IP | -0.57 | | 1.31E-34 | |
| Epithelial | Epithelial | EEF2 | -0.39 | | 1.44E-34 | |
| Epithelial | Epithelial | SERPINA1 | 0.3 | | 1.44E-34 | |
| Epithelial | Epithelial | MYO6 | 0.26 | | 1.47E-34 | |
| Epithelial | Epithelial | RELB | -0.53 | | 1.60E-34 | |
| Epithelial | Epithelial | NDUFS8 | 0.43 | | 1.72E-34 | |
| Epithelial | Epithelial | HSPB1 | 0.32 | | 2.02E-34 | |
| Epithelial | Epithelial | SGSM3 | 0.28 | | 2.44E-34 | |
| Epithelial | Epithelial | RNPEPL1 | 0.32 | | 2.45E-34 | |
| Epithelial | Epithelial | BZW1 | -0.58 | | 3.62E-34 | |
| Epithelial | Epithelial | EVL | -0.69 | | 3.90E-34 | |
| Epithelial | Epithelial | SLC7A5 | -0.71 | | 4.37E-34 | |
| Epithelial | Epithelial | SRSF7 | -0.64 | | 4.84E-34 | |
| Epithelial | Epithelial | SMIM11A | 0.3 | | 5.20E-34 | |
| Epithelial | Epithelial | EIF4G2 | -0.53 | | 5.61E-34 | |
| Epithelial | Epithelial | ARHGAP15 | -0.51 | | 7.40E-34 | |
| Epithelial | Epithelial | ATP5MPL | 0.44 | | 8.75E-34 | |
| Epithelial | Epithelial | PPP1R15A | -0.51 | | 1.04E-33 | |
| Epithelial | Epithelial | RAN | -0.44 | | 1.11E-33 | |
| Epithelial | Epithelial | PHGR1 | 0.59 | | 1.17E-33 | |
| Epithelial | Epithelial | VAMP2 | -0.59 | | 1.30E-33 | |
| Epithelial | Epithelial | HSPA8 | -0.55 | | 1.44E-33 | |
| Epithelial | Epithelial | ATP5MC1 | 0.51 | | 1.52E-33 | |
| Epithelial | Epithelial | MYL6 | -0.4 | | 2.64E-33 | |
| Epithelial | Epithelial | HDLBP | 0.37 | | 2.88E-33 | |
| Epithelial | Epithelial | HNRNPC | -0.49 | | 2.88E-33 | |
| Epithelial | Epithelial | SEC61B | -0.58 | | 3.50E-33 | |
| Epithelial | Epithelial | PAPOLA | -0.51 | | 3.81E-33 | |
| Epithelial | Epithelial | MTLN | 0.32 | | 5.42E-33 | |
| Epithelial | Epithelial | ITGB2 | -0.58 | | 6.78E-33 | |
| Epithelial | Epithelial | ECHDC2 | 0.27 | | 6.85E-33 | |
| Epithelial | Epithelial | TYMP | -0.84 | | 7.81E-33 | |
| Epithelial | Epithelial | UQCRC1 | 0.47 | | 8.00E-33 | |
| Epithelial | Epithelial | INF2 | 0.32 | | 8.23E-33 | |
| Epithelial | Epithelial | ATP2B1 | -0.64 | | 9.47E-33 | |
| Epithelial | Epithelial | SRSF3 | -0.5 | | 1.02E-32 | |
| Epithelial | Epithelial | STX11 | -0.52 | | 1.57E-32 | |
| Epithelial | Epithelial | STAT4 | -0.6 | | 1.82E-32 | |
| Epithelial | Epithelial | FYB1 | -0.62 | | 2.24E-32 | |
| Epithelial | Epithelial | STK17A | -0.66 | | 2.41E-32 | |
| Epithelial | Epithelial | RUNX3 | -0.56 | | 4.44E-32 | |
| Epithelial | Epithelial | RBM39 | -0.47 | | 5.94E-32 | |
| Epithelial | Epithelial | NECTIN2 | 0.25 | | 7.48E-32 | |
| Epithelial | Epithelial | DDX24 | -0.6 | | 7.79E-32 | |
| Epithelial | Epithelial | EPN1 | 0.32 | | 9.38E-32 | |
| Epithelial | Epithelial | HEBP2 | 0.31 | | 1.11E-31 | |
| Epithelial | Epithelial | MRPL12 | 0.38 | | 1.12E-31 | |
| Epithelial | Epithelial | WTAP | -0.58 | | 1.37E-31 | |
| Epithelial | Epithelial | PFN1 | -0.5 | | 1.43E-31 | |
| Epithelial | Epithelial | LCK | -0.62 | | 1.60E-31 | |
| Epithelial | Epithelial | LPCAT4 | 0.29 | | 1.91E-31 | |
| Epithelial | Epithelial | TUBA4A | -0.89 | | 2.23E-31 | |
| Epithelial | Epithelial | RSL24D1 | -0.52 | | 2.26E-31 | |
| Epithelial | Epithelial | ANXA6 | -0.5 | | 2.27E-31 | |
| Epithelial | Epithelial | NFKBIA | -0.71 | | 2.37E-31 | |
| Epithelial | Epithelial | MT2A | 0.42 | | 3.01E-31 | |
| Epithelial | Epithelial | LPXN | -0.47 | | 3.11E-31 | |
| Epithelial | Epithelial | C19orf70 | 0.39 | | 4.23E-31 | |
| Epithelial | Epithelial | CTNNA1 | 0.3 | | 4.27E-31 | |
| Epithelial | Epithelial | RHOA | -0.46 | | 4.87E-31 | |
| Epithelial | Epithelial | CYCS | -0.4 | | 6.06E-31 | |
| Epithelial | Epithelial | NDUFC1 | 0.45 | | 7.56E-31 | |
| Epithelial | Epithelial | IL10RA | -0.45 | | 9.43E-31 | |
| Epithelial | Epithelial | C4orf3 | -0.51 | | 1.09E-30 | |
| Epithelial | Epithelial | ICAM2 | -0.5 | | 1.10E-30 | |
| Epithelial | Epithelial | CNOT6L | -0.63 | | 1.14E-30 | |
| Epithelial | Epithelial | RORA | -0.63 | | 1.22E-30 | |
| Epithelial | Epithelial | MRPL23 | 0.41 | | 1.30E-30 | |
| Epithelial | Epithelial | SH3BGRL | -0.52 | | 1.53E-30 | |
| Epithelial | Epithelial | MAPK1IP1L | -0.56 | | 1.99E-30 | |
| Epithelial | Epithelial | ARHGAP9 | -0.52 | | 2.16E-30 | |
| Epithelial | Epithelial | ITPKC | 0.33 | | 2.87E-30 | |
| Epithelial | Epithelial | YWHAQ | -0.53 | | 2.97E-30 | |
| Epithelial | Epithelial | SNRPB | -0.48 | | 4.07E-30 | |
| Epithelial | Epithelial | TMED3 | 0.36 | | 4.13E-30 | |
| Epithelial | Epithelial | 1-Sep | -0.52 | | 8.50E-30 | |
| Epithelial | Epithelial | HLA-DQB1 | -1.29 | | 9.19E-30 | |
| Epithelial | Epithelial | CELF2 | -0.48 | | 9.59E-30 | |
| Epithelial | Epithelial | UBE2D2 | -0.46 | | 1.06E-29 | |
| Epithelial | Epithelial | ITM2A | -0.83 | | 1.32E-29 | |
| Epithelial | Epithelial | PLEKHJ1 | 0.3 | | 1.52E-29 | |
| Epithelial | Epithelial | FAM49B | -0.53 | | 1.71E-29 | |
| Epithelial | Epithelial | GZMA | -1.27 | | 1.86E-29 | |
| Epithelial | Epithelial | CHP1 | 0.36 | | 2.51E-29 | |
| Epithelial | Epithelial | LY9 | -0.79 | | 2.68E-29 | |
| Epithelial | Epithelial | MEF2C | -0.56 | | 3.75E-29 | |
| Epithelial | Epithelial | ISOC2 | 0.32 | | 4.40E-29 | |
| Epithelial | Epithelial | TRAM1 | -0.53 | | 4.64E-29 | |
| Epithelial | Epithelial | H2AFZ | -0.52 | | 5.00E-29 | |
| Epithelial | Epithelial | CEBPD | 0.4 | | 5.62E-29 | |
| Epithelial | Epithelial | ARL4C | -0.57 | | 6.10E-29 | |
| Epithelial | Epithelial | MAP3K8 | -0.56 | | 6.28E-29 | |
| Epithelial | Epithelial | TRMT112 | -0.45 | | 6.29E-29 | |
| Epithelial | Epithelial | RGS10 | -0.61 | | 9.89E-29 | |
| Epithelial | Epithelial | RPL23 | -0.3 | | 1.06E-28 | |
| Epithelial | Epithelial | CTSS | -0.62 | | 1.70E-28 | |
| Epithelial | Epithelial | TSPAN13 | 0.25 | | 1.91E-28 | |
| Epithelial | Epithelial | DYNC1I2 | 0.29 | | 1.97E-28 | |
| Epithelial | Epithelial | CD27 | -0.61 | | 2.68E-28 | |
| Epithelial | Epithelial | RPS11 | -0.28 | | 3.45E-28 | |
| Epithelial | Epithelial | PTPN7 | -0.49 | | 3.45E-28 | |
| Epithelial | Epithelial | COTL1 | -0.74 | | 3.72E-28 | |
| Epithelial | Epithelial | HLA-DQA1 | -1.21 | | 3.74E-28 | |
| Epithelial | Epithelial | ODC1 | -0.59 | | 3.93E-28 | |
| Epithelial | Epithelial | PLP2 | -0.51 | | 4.35E-28 | |
| Epithelial | Epithelial | HMGB1 | -0.4 | | 5.55E-28 | |
| Epithelial | Epithelial | BCL2A1 | -0.92 | | 6.44E-28 | |
| Epithelial | Epithelial | SLC38A1 | -0.5 | | 6.47E-28 | |
| Epithelial | Epithelial | DSTN | 0.34 | | 6.87E-28 | |
| Epithelial | Epithelial | PIM1 | -0.59 | | 7.70E-28 | |
| Epithelial | Epithelial | NCF1 | -0.44 | | 8.10E-28 | |
| Epithelial | Epithelial | GZMM | -0.64 | | 9.44E-28 | |
| Epithelial | Epithelial | LGALS9 | 0.35 | | 1.12E-27 | |
| Epithelial | Epithelial | LY96 | -0.43 | | 1.75E-27 | |
| Epithelial | Epithelial | CIB1 | -0.47 | | 1.96E-27 | |
| Epithelial | Epithelial | ISCU | -0.37 | | 2.37E-27 | |
| Epithelial | Epithelial | SLC3A2 | -0.55 | | 2.55E-27 | |
| Epithelial | Epithelial | HBB | 0.43 | | 2.80E-27 | |
| Epithelial | Epithelial | NCOA7 | 0.35 | | 2.84E-27 | |
| Epithelial | Epithelial | WAS | -0.4 | | 2.91E-27 | |
| Epithelial | Epithelial | CD247 | -0.56 | | 4.41E-27 | |
| Epithelial | Epithelial | POMP | -0.48 | | 5.57E-27 | |
| Epithelial | Epithelial | HINT1 | -0.36 | | 5.90E-27 | |
| Epithelial | Epithelial | SERF2 | 0.33 | | 6.18E-27 | |
| Epithelial | Epithelial | CHMP1B | -0.73 | | 8.20E-27 | |
| Epithelial | Epithelial | PIM2 | -0.62 | | 8.28E-27 | |
| Epithelial | Epithelial | EIF1B | -0.54 | | 8.92E-27 | |
| Epithelial | Epithelial | HLA-F | -0.49 | | 9.41E-27 | |
| Epithelial | Epithelial | ANKRD28 | -0.71 | | 1.09E-26 | |
| Epithelial | Epithelial | RPL26 | -0.26 | | 1.10E-26 | |
| Epithelial | Epithelial | P2RY10 | -0.44 | | 1.11E-26 | |
| Epithelial | Epithelial | ARID4B | -0.59 | | 1.13E-26 | |
| Epithelial | Epithelial | LMO4 | 0.32 | | 1.52E-26 | |
| Epithelial | Epithelial | TOMM7 | -0.36 | | 1.59E-26 | |
| Epithelial | Epithelial | ARL4A | -0.61 | | 1.92E-26 | |
| Epithelial | Epithelial | BAD | 0.27 | | 1.95E-26 | |
| Epithelial | Epithelial | PFKL | 0.32 | | 1.96E-26 | |
| Epithelial | Epithelial | UBE2N | -0.51 | | 2.09E-26 | |
| Epithelial | Epithelial | HINT2 | 0.36 | | 2.27E-26 | |
| Epithelial | Epithelial | TYROBP | -1.45 | | 2.63E-26 | |
| Epithelial | Epithelial | PHF1 | -0.47 | | 3.23E-26 | |
| Epithelial | Epithelial | MGAT4A | -0.54 | | 4.26E-26 | |
| Epithelial | Epithelial | SDCBP2 | 0.28 | | 7.28E-26 | |
| Epithelial | Epithelial | SYAP1 | -0.59 | | 7.97E-26 | |
| Epithelial | Epithelial | NDUFA13 | 0.45 | | 8.16E-26 | |
| Epithelial | Epithelial | VOPP1 | -0.41 | | 1.00E-25 | |
| Epithelial | Epithelial | IFI16 | -0.53 | | 1.16E-25 | |
| Epithelial | Epithelial | ARF6 | -0.51 | | 1.24E-25 | |
| Epithelial | Epithelial | NDUFS7 | 0.42 | | 1.27E-25 | |
| Epithelial | Epithelial | TNFRSF18 | -0.61 | | 1.45E-25 | |
| Epithelial | Epithelial | FTL | -0.79 | | 2.14E-25 | |
| Epithelial | Epithelial | G3BP2 | -0.56 | | 2.61E-25 | |
| Epithelial | Epithelial | C16orf54 | -0.46 | | 3.15E-25 | |
| Epithelial | Epithelial | RHEB | -0.44 | | 3.62E-25 | |
| Epithelial | Epithelial | EEF1D | -0.26 | | 3.79E-25 | |
| Epithelial | Epithelial | ODF2L | -0.44 | | 3.92E-25 | |
| Epithelial | Epithelial | TAGLN2 | -0.4 | | 5.12E-25 | |
| Epithelial | Epithelial | PRXL2C | -0.49 | | 5.19E-25 | |
| Epithelial | Epithelial | SH2D2A | -0.58 | | 5.70E-25 | |
| Epithelial | Epithelial | MZT2B | 0.37 | | 6.82E-25 | |
| Epithelial | Epithelial | NDUFB10 | 0.38 | | 7.35E-25 | |
| Epithelial | Epithelial | EMB | -0.42 | | 8.30E-25 | |
| Epithelial | Epithelial | PSMB9 | -0.5 | | 9.19E-25 | |
| Epithelial | Epithelial | BAZ1A | -0.55 | | 9.23E-25 | |
| Epithelial | Epithelial | CD3G | -0.54 | | 1.16E-24 | |
| Epithelial | Epithelial | UCP2 | -0.63 | | 1.19E-24 | |
| Epithelial | Epithelial | TBC1D10C | -0.44 | | 1.40E-24 | |
| Epithelial | Epithelial | SF1 | -0.49 | | 1.99E-24 | |
| Epithelial | Epithelial | ATP6AP2 | -0.46 | | 2.03E-24 | |
| Epithelial | Epithelial | ZEB2 | -0.44 | | 2.44E-24 | |
| Epithelial | Epithelial | SUCLG2 | 0.33 | | 2.59E-24 | |
| Epithelial | Epithelial | AC114760.2 | -0.48 | | 3.32E-24 | |
| Epithelial | Epithelial | CMTM6 | -0.56 | | 4.15E-24 | |
| Epithelial | Epithelial | EML4 | -0.58 | | 4.82E-24 | |
| Epithelial | Epithelial | GAPDH | -0.43 | | 4.92E-24 | |
| Epithelial | Epithelial | CCND2 | -0.46 | | 5.25E-24 | |
| Epithelial | Epithelial | DUSP2 | -0.88 | | 5.57E-24 | |
| Epithelial | Epithelial | ORAI2 | -0.39 | | 5.70E-24 | |
| Epithelial | Epithelial | CD59 | 0.26 | | 6.06E-24 | |
| Epithelial | Epithelial | SDCBP | -0.58 | | 7.50E-24 | |
| Epithelial | Epithelial | PPP1R18 | -0.36 | | 7.89E-24 | |
| Epithelial | Epithelial | PRDX2 | 0.37 | | 8.10E-24 | |
| Epithelial | Epithelial | UPP1 | -0.44 | | 8.32E-24 | |
| Epithelial | Epithelial | UBE2A | -0.45 | | 8.47E-24 | |
| Epithelial | Epithelial | CD96 | -0.53 | | 8.86E-24 | |
| Epithelial | Epithelial | KLRB1 | -1.24 | | 1.01E-23 | |
| Epithelial | Epithelial | SRP14 | -0.31 | | 1.02E-23 | |
| Epithelial | Epithelial | ARHGAP45 | -0.37 | | 1.03E-23 | |
| Epithelial | Epithelial | SSR2 | -0.38 | | 1.11E-23 | |
| Epithelial | Epithelial | MS4A1 | -0.73 | | 1.13E-23 | |
| Epithelial | Epithelial | GZMB | -1.4 | | 1.50E-23 | |
| Epithelial | Epithelial | PTPN22 | -0.48 | | 1.90E-23 | |
| Epithelial | Epithelial | OST4 | -0.31 | | 1.99E-23 | |
| Epithelial | Epithelial | GRB2 | -0.41 | | 2.42E-23 | |
| Epithelial | Epithelial | RHOB | 0.48 | | 2.44E-23 | |
| Epithelial | Epithelial | SLC25A6 | -0.28 | | 2.58E-23 | |
| Epithelial | Epithelial | CD6 | -0.49 | | 3.47E-23 | |
| Epithelial | Epithelial | RASSF5 | -0.5 | | 3.83E-23 | |
| Epithelial | Epithelial | HNRNPUL1 | -0.51 | | 3.83E-23 | |
| Epithelial | Epithelial | PIN4 | 0.27 | | 3.86E-23 | |
| Epithelial | Epithelial | ARF1 | -0.37 | | 3.87E-23 | |
| Epithelial | Epithelial | GNAI2 | -0.45 | | 4.13E-23 | |
| Epithelial | Epithelial | CYC1 | 0.43 | | 4.61E-23 | |
| Epithelial | Epithelial | BAG3 | 0.28 | | 5.77E-23 | |
| Epithelial | Epithelial | PBXIP1 | -0.56 | | 7.57E-23 | |
| Epithelial | Epithelial | RPL22 | -0.28 | | 1.03E-22 | |
| Epithelial | Epithelial | AURKAIP1 | 0.36 | | 1.23E-22 | |
| Epithelial | Epithelial | SLA | -0.5 | | 1.25E-22 | |
| Epithelial | Epithelial | RPS27L | 0.36 | | 1.42E-22 | |
| Epithelial | Epithelial | DNAJB9 | -0.64 | | 1.56E-22 | |
| Epithelial | Epithelial | ATP5MC3 | 0.54 | | 1.57E-22 | |
| Epithelial | Epithelial | CD8A | -0.77 | | 1.58E-22 | |
| Epithelial | Epithelial | SNX3 | -0.41 | | 1.69E-22 | |
| Epithelial | Epithelial | CSRNP1 | -0.49 | | 1.90E-22 | |
| Epithelial | Epithelial | FAM49A | -0.34 | | 1.95E-22 | |
| Epithelial | Epithelial | CLDND1 | -0.51 | | 2.05E-22 | |
| Epithelial | Epithelial | ISG15 | -0.74 | | 2.28E-22 | |
| Epithelial | Epithelial | HMGB2 | -0.54 | | 2.34E-22 | |
| Epithelial | Epithelial | P4HB | 0.44 | | 2.50E-22 | |
| Epithelial | Epithelial | A1BG | -0.3 | | 3.08E-22 | |
| Epithelial | Epithelial | MOB1A | -0.47 | | 4.19E-22 | |
| Epithelial | Epithelial | TIMP1 | -1.99 | | 4.60E-22 | |
| Epithelial | Epithelial | CCNH | -0.49 | | 5.40E-22 | |
| Epithelial | Epithelial | CFLAR | -0.53 | | 5.60E-22 | |
| Epithelial | Epithelial | JOSD2 | 0.26 | | 5.75E-22 | |
| Epithelial | Epithelial | RPS12 | -0.26 | | 5.75E-22 | |
| Epithelial | Epithelial | ITGB7 | -0.34 | | 6.01E-22 | |
| Epithelial | Epithelial | TPM3 | -0.44 | | 7.84E-22 | |
| Epithelial | Epithelial | PTPN1 | -0.37 | | 9.01E-22 | |
| Epithelial | Epithelial | RUNX1 | 0.28 | | 9.15E-22 | |
| Epithelial | Epithelial | ICOS | -0.55 | | 1.12E-21 | |
| Epithelial | Epithelial | COMT | 0.29 | | 1.29E-21 | |
| Epithelial | Epithelial | CD79B | -0.39 | | 1.67E-21 | |
| Epithelial | Epithelial | PHF14 | 0.31 | | 1.69E-21 | |
| Epithelial | Epithelial | TCF4 | -0.42 | | 1.94E-21 | |
| Epithelial | Epithelial | CHD1 | -0.47 | | 2.06E-21 | |
| Epithelial | Epithelial | BCAS2 | -0.52 | | 2.25E-21 | |
| Epithelial | Epithelial | ISCA1 | -0.48 | | 2.32E-21 | |
| Epithelial | Epithelial | CYLD | -0.39 | | 2.32E-21 | |
| Epithelial | Epithelial | CHCHD5 | 0.28 | | 2.85E-21 | |
| Epithelial | Epithelial | CAP1 | -0.46 | | 2.89E-21 | |
| Epithelial | Epithelial | FGFR1OP2 | -0.4 | | 3.10E-21 | |
| Epithelial | Epithelial | JAK1 | -0.44 | | 3.15E-21 | |
| Epithelial | Epithelial | ZNF593 | 0.31 | | 3.26E-21 | |
| Epithelial | Epithelial | SMAP2 | -0.45 | | 3.89E-21 | |
| Epithelial | Epithelial | PSMB1 | -0.37 | | 4.88E-21 | |
| Epithelial | Epithelial | CLEC2D | -0.43 | | 5.61E-21 | |
| Epithelial | Epithelial | VSIR | -0.37 | | 5.72E-21 | |
| Epithelial | Epithelial | MYO1G | -0.33 | | 6.18E-21 | |
| Epithelial | Epithelial | LAPTM4A | -0.46 | | 6.36E-21 | |
| Epithelial | Epithelial | TMEM243 | -0.47 | | 6.99E-21 | |
| Epithelial | Epithelial | TNFRSF4 | -0.75 | | 7.54E-21 | |
| Epithelial | Epithelial | CD38 | -0.34 | | 1.01E-20 | |
| Epithelial | Epithelial | FCER1G | -1.54 | | 1.04E-20 | |
| Epithelial | Epithelial | GIMAP7 | -0.51 | | 1.04E-20 | |
| Epithelial | Epithelial | CTSW | -0.64 | | 1.09E-20 | |
| Epithelial | Epithelial | RPS10 | -0.3 | | 1.23E-20 | |
| Epithelial | Epithelial | AKIRIN2 | -0.4 | | 1.39E-20 | |
| Epithelial | Epithelial | APOBEC3G | -0.49 | | 1.53E-20 | |
| Epithelial | Epithelial | OSTF1 | -0.43 | | 1.87E-20 | |
| Epithelial | Epithelial | LINC00513 | -0.38 | | 1.99E-20 | |
| Epithelial | Epithelial | HERPUD1 | -1.03 | | 2.27E-20 | |
| Epithelial | Epithelial | BLOC1S1 | 0.34 | | 2.29E-20 | |
| Epithelial | Epithelial | LAT | -0.43 | | 2.37E-20 | |
| Epithelial | Epithelial | BANK1 | -0.51 | | 3.33E-20 | |
| Epithelial | Epithelial | GZMK | -1.09 | | 4.10E-20 | |
| Epithelial | Epithelial | SYTL3 | -0.43 | | 4.48E-20 | |
| Epithelial | Epithelial | GTF3A | -0.41 | | 5.22E-20 | |
| Epithelial | Epithelial | ENO1 | -0.45 | | 6.09E-20 | |
| Epithelial | Epithelial | C11orf58 | -0.39 | | 6.31E-20 | |
| Epithelial | Epithelial | MAGOH | -0.41 | | 6.32E-20 | |
| Epithelial | Epithelial | STRAP | -0.4 | | 6.36E-20 | |
| Epithelial | Epithelial | THEMIS2 | -0.27 | | 6.43E-20 | |
| Epithelial | Epithelial | EPC1 | -0.46 | | 9.64E-20 | |
| Epithelial | Epithelial | S1PR4 | -0.37 | | 1.22E-19 | |
| Epithelial | Epithelial | CDKN1A | -0.63 | | 1.22E-19 | |
| Epithelial | Epithelial | GPBP1 | -0.46 | | 1.34E-19 | |
| Epithelial | Epithelial | TMEM123 | -0.51 | | 1.55E-19 | |
| Epithelial | Epithelial | C9orf78 | -0.47 | | 1.60E-19 | |
| Epithelial | Epithelial | AKNA | -0.45 | | 1.84E-19 | |
| Epithelial | Epithelial | TIGIT | -0.6 | | 1.99E-19 | |
| Epithelial | Epithelial | EMD | -0.41 | | 2.09E-19 | |
| Epithelial | Epithelial | ZNHIT1 | 0.28 | | 2.18E-19 | |
| Epithelial | Epithelial | LRRFIP1 | -0.46 | | 2.19E-19 | |
| Epithelial | Epithelial | GNAI3 | -0.43 | | 2.25E-19 | |
| Epithelial | Epithelial | GABARAPL1 | -0.49 | | 2.29E-19 | |
| Epithelial | Epithelial | DAZAP2 | -0.4 | | 2.43E-19 | |
| Epithelial | Epithelial | SH3KBP1 | -0.35 | | 2.44E-19 | |
| Epithelial | Epithelial | YWHAB | -0.4 | | 2.74E-19 | |
| Epithelial | Epithelial | VPREB3 | -0.5 | | 2.81E-19 | |
| Epithelial | Epithelial | UBE2I | -0.42 | | 2.97E-19 | |
| Epithelial | Epithelial | CDC42SE1 | -0.47 | | 3.25E-19 | |
| Epithelial | Epithelial | SPCS2 | -0.52 | | 3.38E-19 | |
| Epithelial | Epithelial | CCDC124 | 0.29 | | 3.51E-19 | |
| Epithelial | Epithelial | BOLA3 | 0.27 | | 3.71E-19 | |
| Epithelial | Epithelial | STOM | -0.44 | | 3.85E-19 | |
| Epithelial | Epithelial | CD74 | -1.19 | | 5.00E-19 | |
| Epithelial | Epithelial | NINJ1 | -0.6 | | 5.12E-19 | |
| Epithelial | Epithelial | DOK2 | -0.37 | | 5.13E-19 | |
| Epithelial | Epithelial | PDCL3 | -0.53 | | 5.61E-19 | |
| Epithelial | Epithelial | NSMCE3 | -0.39 | | 5.61E-19 | |
| Epithelial | Epithelial | CCDC69 | -0.28 | | 6.01E-19 | |
| Epithelial | Epithelial | HSPA5 | -0.51 | | 6.49E-19 | |
| Epithelial | Epithelial | DPP7 | -0.39 | | 7.31E-19 | |
| Epithelial | Epithelial | CD47 | -0.4 | | 7.50E-19 | |
| Epithelial | Epithelial | IL2RB | -0.45 | | 7.96E-19 | |
| Epithelial | Epithelial | ATP5MD | 0.46 | | 9.85E-19 | |
| Epithelial | Epithelial | NDUFB2 | 0.36 | | 1.06E-18 | |
| Epithelial | Epithelial | ARPC5 | -0.42 | | 1.10E-18 | |
| Epithelial | Epithelial | IKZF1 | -0.3 | | 1.13E-18 | |
| Epithelial | Epithelial | BASP1 | -0.37 | | 1.16E-18 | |
| Epithelial | Epithelial | DNPH1 | 0.32 | | 1.24E-18 | |
| Epithelial | Epithelial | RHOF | -0.38 | | 1.30E-18 | |
| Epithelial | Epithelial | FCMR | -0.4 | | 1.40E-18 | |
| Epithelial | Epithelial | CSDE1 | -0.37 | | 1.53E-18 | |
| Epithelial | Epithelial | SMCHD1 | -0.47 | | 1.56E-18 | |
| Epithelial | Epithelial | SELL | -0.41 | | 1.63E-18 | |
| Epithelial | Epithelial | HNRNPF | -0.36 | | 2.00E-18 | |
| Epithelial | Epithelial | PTGER4 | -0.55 | | 2.24E-18 | |
| Epithelial | Epithelial | MAPRE1 | -0.41 | | 2.30E-18 | |
| Epithelial | Epithelial | BIN2 | -0.32 | | 2.31E-18 | |
| Epithelial | Epithelial | ABRACL | -0.46 | | 2.35E-18 | |
| Epithelial | Epithelial | PRDM1 | -0.42 | | 2.36E-18 | |
| Epithelial | Epithelial | LAG3 | -0.52 | | 2.43E-18 | |
| Epithelial | Epithelial | DRAP1 | -0.44 | | 2.54E-18 | |
| Epithelial | Epithelial | SATB1 | -0.41 | | 2.67E-18 | |
| Epithelial | Epithelial | PRKCB | -0.33 | | 2.74E-18 | |
| Epithelial | Epithelial | PGAM1 | -0.41 | | 2.81E-18 | |
| Epithelial | Epithelial | NAA38 | 0.29 | | 2.85E-18 | |
| Epithelial | Epithelial | PLIN2 | -0.58 | | 2.94E-18 | |
| Epithelial | Epithelial | MEI1 | -0.27 | | 3.00E-18 | |
| Epithelial | Epithelial | VAMP5 | -0.4 | | 3.25E-18 | |
| Epithelial | Epithelial | HLA-DQA2 | -0.82 | | 3.61E-18 | |
| Epithelial | Epithelial | ITM2C | -0.76 | | 4.03E-18 | |
| Epithelial | Epithelial | TAP1 | -0.4 | | 4.47E-18 | |
| Epithelial | Epithelial | MBNL1 | -0.43 | | 4.58E-18 | |
| Epithelial | Epithelial | DDT | 0.44 | | 5.00E-18 | |
| Epithelial | Epithelial | EIF4G1 | 0.28 | | 6.04E-18 | |
| Epithelial | Epithelial | PRKCH | -0.38 | | 6.16E-18 | |
| Epithelial | Epithelial | P2RY8 | -0.33 | | 6.72E-18 | |
| Epithelial | Epithelial | SYTL2 | 0.29 | | 6.76E-18 | |
| Epithelial | Epithelial | ALG13 | -0.49 | | 7.51E-18 | |
| Epithelial | Epithelial | SH3GLB1 | -0.35 | | 7.70E-18 | |
| Epithelial | Epithelial | DNAJA1 | -0.37 | | 8.56E-18 | |
| Epithelial | Epithelial | TENT5C | -0.55 | | 8.70E-18 | |
| Epithelial | Epithelial | MCUB | -0.37 | | 9.45E-18 | |
| Epithelial | Epithelial | CAPZA1 | -0.39 | | 1.01E-17 | |
| Epithelial | Epithelial | ETS1 | -0.42 | | 1.03E-17 | |
| Epithelial | Epithelial | CTSC | -0.55 | | 1.15E-17 | |
| Epithelial | Epithelial | CMTM3 | -0.28 | | 1.44E-17 | |
| Epithelial | Epithelial | ARHGAP30 | -0.3 | | 1.59E-17 | |
| Epithelial | Epithelial | GNG7 | -0.41 | | 1.74E-17 | |
| Epithelial | Epithelial | CD19 | -0.32 | | 1.79E-17 | |
| Epithelial | Epithelial | PDCD4 | -0.48 | | 1.80E-17 | |
| Epithelial | Epithelial | MRPS6 | -0.47 | | 1.85E-17 | |
| Epithelial | Epithelial | UBXN1 | -0.35 | | 1.90E-17 | |
| Epithelial | Epithelial | LST1 | -0.51 | | 1.90E-17 | |
| Epithelial | Epithelial | TSPYL2 | -0.46 | | 1.99E-17 | |
| Epithelial | Epithelial | XRCC6 | -0.39 | | 2.00E-17 | |
| Epithelial | Epithelial | NXT1 | -0.4 | | 2.24E-17 | |
| Epithelial | Epithelial | TRAT1 | -0.43 | | 2.39E-17 | |
| Epithelial | Epithelial | HMGN3 | 0.27 | | 2.74E-17 | |
| Epithelial | Epithelial | NCF4 | -0.25 | | 2.91E-17 | |
| Epithelial | Epithelial | SELENOH | 0.31 | | 3.08E-17 | |
| Epithelial | Epithelial | NT5C | 0.26 | | 3.09E-17 | |
| Epithelial | Epithelial | SKIL | -0.48 | | 3.11E-17 | |
| Epithelial | Epithelial | LY86 | -0.34 | | 3.28E-17 | |
| Epithelial | Epithelial | C6orf48 | -0.38 | | 3.41E-17 | |
| Epithelial | Epithelial | ENSA | -0.39 | | 4.19E-17 | |
| Epithelial | Epithelial | AC004687.1 | -0.33 | | 4.33E-17 | |
| Epithelial | Epithelial | AP1S2 | -0.29 | | 4.46E-17 | |
| Epithelial | Epithelial | RNF125 | -0.38 | | 4.53E-17 | |
| Epithelial | Epithelial | RASAL3 | -0.3 | | 5.00E-17 | |
| Epithelial | Epithelial | PIK3IP1 | -0.45 | | 5.46E-17 | |
| Epithelial | Epithelial | GBP2 | -0.37 | | 5.70E-17 | |
| Epithelial | Epithelial | ELMO1 | -0.28 | | 6.12E-17 | |
| Epithelial | Epithelial | ARPC1A | 0.27 | | 6.82E-17 | |
| Epithelial | Epithelial | ENTPD1 | -0.31 | | 7.49E-17 | |
| Epithelial | Epithelial | PHPT1 | 0.26 | | 8.58E-17 | |
| Epithelial | Epithelial | SELPLG | -0.3 | | 9.29E-17 | |
| Epithelial | Epithelial | UBE2J1 | -0.45 | | 9.60E-17 | |
| Epithelial | Epithelial | POLR2L | 0.32 | | 1.06E-16 | |
| Epithelial | Epithelial | ARL6IP5 | -0.46 | | 1.08E-16 | |
| Epithelial | Epithelial | GZMH | -0.79 | | 1.15E-16 | |
| Epithelial | Epithelial | PSMA7 | -0.31 | | 1.20E-16 | |
| Epithelial | Epithelial | RBMX | -0.4 | | 1.22E-16 | |
| Epithelial | Epithelial | SLC16A3 | -0.44 | | 1.60E-16 | |
| Epithelial | Epithelial | TANK | -0.41 | | 1.68E-16 | |
| Epithelial | Epithelial | ARHGAP4 | -0.3 | | 1.71E-16 | |
| Epithelial | Epithelial | SPI1 | -0.29 | | 1.76E-16 | |
| Epithelial | Epithelial | NDUFS2 | 0.29 | | 2.05E-16 | |
| Epithelial | Epithelial | ABI3 | -0.32 | | 2.13E-16 | |
| Epithelial | Epithelial | DUSP10 | -0.4 | | 2.28E-16 | |
| Epithelial | Epithelial | RAP1A | -0.4 | | 2.38E-16 | |
| Epithelial | Epithelial | PRF1 | -0.64 | | 2.43E-16 | |
| Epithelial | Epithelial | N4BP2L1 | -0.28 | | 2.83E-16 | |
| Epithelial | Epithelial | LINC01871 | -0.54 | | 2.90E-16 | |
| Epithelial | Epithelial | ACTR3 | -0.37 | | 3.00E-16 | |
| Epithelial | Epithelial | DERL1 | -0.35 | | 3.22E-16 | |
| Epithelial | Epithelial | JAML | -0.36 | | 3.83E-16 | |
| Epithelial | Epithelial | TAF7 | -0.4 | | 4.89E-16 | |
| Epithelial | Epithelial | APBB1IP | -0.31 | | 5.31E-16 | |
| Epithelial | Epithelial | SCP2 | 0.33 | | 5.85E-16 | |
| Epithelial | Epithelial | DNTTIP2 | -0.38 | | 6.51E-16 | |
| Epithelial | Epithelial | DNAJC8 | -0.39 | | 7.09E-16 | |
| Epithelial | Epithelial | HNRNPA2B1 | -0.28 | | 7.38E-16 | |
| Epithelial | Epithelial | NR1H2 | -0.34 | | 7.41E-16 | |
| Epithelial | Epithelial | HLA-DRB5 | -0.92 | | 7.46E-16 | |
| Epithelial | Epithelial | SAP18 | -0.31 | | 7.60E-16 | |
| Epithelial | Epithelial | RNF149 | -0.4 | | 8.95E-16 | |
| Epithelial | Epithelial | EHD1 | -0.38 | | 9.18E-16 | |
| Epithelial | Epithelial | CUTA | -0.39 | | 9.97E-16 | |
| Epithelial | Epithelial | PRSS3 | 0.26 | | 1.13E-15 | |
| Epithelial | Epithelial | CHST11 | -0.29 | | 1.24E-15 | |
| Epithelial | Epithelial | BLOC1S2 | -0.36 | | 1.34E-15 | |
| Epithelial | Epithelial | TPM4 | -0.4 | | 1.78E-15 | |
| Epithelial | Epithelial | RPS4Y1 | -0.39 | | 1.80E-15 | |
| Epithelial | Epithelial | ANKRD44 | -0.3 | | 1.89E-15 | |
| Epithelial | Epithelial | U2AF1 | -0.33 | | 2.19E-15 | |
| Epithelial | Epithelial | HERPUD2 | -0.33 | | 2.20E-15 | |
| Epithelial | Epithelial | SNU13 | -0.3 | | 2.36E-15 | |
| Epithelial | Epithelial | PHLDA1 | -0.65 | | 2.41E-15 | |
| Epithelial | Epithelial | AHI1 | 0.25 | | 2.48E-15 | |
| Epithelial | Epithelial | SP140 | -0.28 | | 2.52E-15 | |
| Epithelial | Epithelial | CAPZB | -0.35 | | 2.64E-15 | |
| Epithelial | Epithelial | RAB7A | -0.37 | | 3.05E-15 | |
| Epithelial | Epithelial | PPM1K | -0.35 | | 3.27E-15 | |
| Epithelial | Epithelial | LMNA | -0.7 | | 3.64E-15 | |
| Epithelial | Epithelial | ZFAS1 | -0.34 | | 3.85E-15 | |
| Epithelial | Epithelial | DOCK8 | -0.3 | | 3.93E-15 | |
| Epithelial | Epithelial | CKLF | -0.41 | | 3.95E-15 | |
| Epithelial | Epithelial | MIR4435-2HG | -0.39 | | 3.96E-15 | |
| Epithelial | Epithelial | IFNAR2 | -0.25 | | 4.65E-15 | |
| Epithelial | Epithelial | NDUFA3 | 0.36 | | 5.00E-15 | |
| Epithelial | Epithelial | SKAP2 | -0.26 | | 5.30E-15 | |
| Epithelial | Epithelial | KDM6B | -0.42 | | 6.39E-15 | |
| Epithelial | Epithelial | PTGES3 | -0.31 | | 6.43E-15 | |
| Epithelial | Epithelial | FUS | -0.35 | | 6.89E-15 | |
| Epithelial | Epithelial | GPR65 | -0.32 | | 7.56E-15 | |
| Epithelial | Epithelial | TXNDC15 | -0.33 | | 7.78E-15 | |
| Epithelial | Epithelial | GPX4 | -0.36 | | 8.22E-15 | |
| Epithelial | Epithelial | DNAJB1 | 0.44 | | 8.74E-15 | |
| Epithelial | Epithelial | RAB8B | -0.29 | | 8.82E-15 | |
| Epithelial | Epithelial | IL16 | -0.27 | | 9.04E-15 | |
| Epithelial | Epithelial | BIRC3 | -0.41 | | 1.10E-14 | |
| Epithelial | Epithelial | VMP1 | 0.29 | | 1.26E-14 | |
| Epithelial | Epithelial | PPP2CA | -0.37 | | 1.32E-14 | |
| Epithelial | Epithelial | GTF2B | -0.34 | | 1.38E-14 | |
| Epithelial | Epithelial | SUMO1 | -0.35 | | 1.52E-14 | |
| Epithelial | Epithelial | FAM162A | 0.32 | | 1.54E-14 | |
| Epithelial | Epithelial | BRK1 | -0.29 | | 1.65E-14 | |
| Epithelial | Epithelial | ROCK1 | -0.39 | | 1.65E-14 | |
| Epithelial | Epithelial | AC044849.1 | -0.32 | | 1.69E-14 | |
| Epithelial | Epithelial | DDX3X | -0.37 | | 1.72E-14 | |
| Epithelial | Epithelial | RNF166 | -0.3 | | 1.80E-14 | |
| Epithelial | Epithelial | KPNA2 | -0.34 | | 2.26E-14 | |
| Epithelial | Epithelial | ZC3HAV1 | -0.46 | | 2.43E-14 | |
| Epithelial | Epithelial | FAM129A | -0.33 | | 2.59E-14 | |
| Epithelial | Epithelial | ARPP19 | -0.33 | | 2.79E-14 | |
| Epithelial | Epithelial | RAB30 | -0.32 | | 2.81E-14 | |
| Epithelial | Epithelial | RPS26 | -0.3 | | 2.90E-14 | |
| Epithelial | Epithelial | EFHD2 | 0.28 | | 3.04E-14 | |
| Epithelial | Epithelial | BCL7B | -0.35 | | 3.15E-14 | |
| Epithelial | Epithelial | POU2F2 | -0.26 | | 3.45E-14 | |
| Epithelial | Epithelial | MIR155HG | -0.35 | | 3.58E-14 | |
| Epithelial | Epithelial | TNFRSF25 | -0.32 | | 3.86E-14 | |
| Epithelial | Epithelial | MRPL57 | 0.26 | | 3.99E-14 | |
| Epithelial | Epithelial | TPST2 | -0.27 | | 4.13E-14 | |
| Epithelial | Epithelial | NFKBID | -0.38 | | 4.14E-14 | |
| Epithelial | Epithelial | SLBP | -0.42 | | 4.15E-14 | |
| Epithelial | Epithelial | CCR6 | -0.34 | | 4.25E-14 | |
| Epithelial | Epithelial | NDUFA2 | 0.29 | | 4.44E-14 | |
| Epithelial | Epithelial | REEP5 | -0.38 | | 4.93E-14 | |
| Epithelial | Epithelial | EID1 | -0.42 | | 5.07E-14 | |
| Epithelial | Epithelial | HLA-DRA | -1.84 | | 5.35E-14 | |
| Epithelial | Epithelial | NRBP1 | -0.31 | | 5.61E-14 | |
| Epithelial | Epithelial | AKAP13 | -0.41 | | 5.88E-14 | |
| Epithelial | Epithelial | NASP | -0.4 | | 6.36E-14 | |
| Epithelial | Epithelial | MAF1 | -0.33 | | 6.67E-14 | |
| Epithelial | Epithelial | PRKAR1A | -0.37 | | 7.12E-14 | |
| Epithelial | Epithelial | ETF1 | -0.36 | | 7.30E-14 | |
| Epithelial | Epithelial | ATP5PF | 0.39 | | 8.08E-14 | |
| Epithelial | Epithelial | CXCR3 | -0.4 | | 8.54E-14 | |
| Epithelial | Epithelial | UXT | -0.29 | | 8.97E-14 | |
| Epithelial | Epithelial | SF3B2 | -0.35 | | 9.10E-14 | |
| Epithelial | Epithelial | SNX9 | -0.4 | | 9.59E-14 | |
| Epithelial | Epithelial | CLK1 | -0.38 | | 9.67E-14 | |
| Epithelial | Epithelial | CD58 | -0.33 | | 1.13E-13 | |
| Epithelial | Epithelial | NSD3 | -0.36 | | 1.19E-13 | |
| Epithelial | Epithelial | IRF7 | -0.35 | | 1.19E-13 | |
| Epithelial | Epithelial | LIMS1 | -0.4 | | 1.39E-13 | |
| Epithelial | Epithelial | MYL12A | -0.36 | | 1.53E-13 | |
| Epithelial | Epithelial | VPS37B | -0.5 | | 1.60E-13 | |
| Epithelial | Epithelial | ATP6V0B | -0.36 | | 1.62E-13 | |
| Epithelial | Epithelial | METTL26 | 0.26 | | 1.73E-13 | |
| Epithelial | Epithelial | SKAP1 | -0.29 | | 1.92E-13 | |
| Epithelial | Epithelial | RASGRP2 | -0.3 | | 1.99E-13 | |
| Epithelial | Epithelial | RSRC2 | -0.38 | | 2.26E-13 | |
| Epithelial | Epithelial | HMGN1 | -0.3 | | 2.37E-13 | |
| Epithelial | Epithelial | NFKBIZ | 0.37 | | 2.40E-13 | |
| Epithelial | Epithelial | CASP4 | -0.33 | | 2.48E-13 | |
| Epithelial | Epithelial | NCL | -0.34 | | 2.53E-13 | |
| Epithelial | Epithelial | GABARAPL2 | -0.33 | | 2.91E-13 | |
| Epithelial | Epithelial | HBP1 | -0.33 | | 3.26E-13 | |
| Epithelial | Epithelial | PET100 | 0.28 | | 3.34E-13 | |
| Epithelial | Epithelial | SP100 | -0.35 | | 3.60E-13 | |
| Epithelial | Epithelial | RARA | -0.28 | | 3.88E-13 | |
| Epithelial | Epithelial | SS18L2 | -0.4 | | 4.18E-13 | |
| Epithelial | Epithelial | GNLY | -1.75 | | 4.22E-13 | |
| Epithelial | Epithelial | HNRNPM | -0.35 | | 4.67E-13 | |
| Epithelial | Epithelial | ITGA4 | -0.26 | | 5.09E-13 | |
| Epithelial | Epithelial | TINF2 | -0.29 | | 5.61E-13 | |
| Epithelial | Epithelial | PLK3 | -0.37 | | 5.63E-13 | |
| Epithelial | Epithelial | MPC2 | 0.36 | | 7.61E-13 | |
| Epithelial | Epithelial | RNPS1 | -0.34 | | 8.27E-13 | |
| Epithelial | Epithelial | PFKFB3 | -0.36 | | 8.43E-13 | |
| Epithelial | Epithelial | HLA-DMA | -0.58 | | 8.99E-13 | |
| Epithelial | Epithelial | MAP2K1 | -0.29 | | 1.19E-12 | |
| Epithelial | Epithelial | BSG | 0.27 | | 1.22E-12 | |
| Epithelial | Epithelial | PSMB8-AS1 | -0.25 | | 1.31E-12 | |
| Epithelial | Epithelial | AIF1 | -0.66 | | 1.63E-12 | |
| Epithelial | Epithelial | TCEA1 | -0.35 | | 1.85E-12 | |
| Epithelial | Epithelial | SH3BP5 | -0.27 | | 1.90E-12 | |
| Epithelial | Epithelial | TBCC | -0.31 | | 1.95E-12 | |
| Epithelial | Epithelial | TFF3 | 0.49 | | 2.14E-12 | |
| Epithelial | Epithelial | PRDX3 | 0.26 | | 2.20E-12 | |
| Epithelial | Epithelial | EDF1 | 0.26 | | 2.46E-12 | |
| Epithelial | Epithelial | SRPRA | -0.27 | | 2.50E-12 | |
| Epithelial | Epithelial | RNASET2 | -0.37 | | 2.54E-12 | |
| Epithelial | Epithelial | GNA13 | -0.3 | | 2.67E-12 | |
| Epithelial | Epithelial | RHBDD2 | -0.42 | | 3.11E-12 | |
| Epithelial | Epithelial | NUDT4 | -0.32 | | 3.30E-12 | |
| Epithelial | Epithelial | IGFBP7 | -1.79 | | 3.44E-12 | |
| Epithelial | Epithelial | UBE2D1 | -0.32 | | 3.55E-12 | |
| Epithelial | Epithelial | EMC7 | -0.31 | | 3.65E-12 | |
| Epithelial | Epithelial | 9-Sep | -0.35 | | 4.25E-12 | |
| Epithelial | Epithelial | TMEM59 | -0.37 | | 4.38E-12 | |
| Epithelial | Epithelial | METTL9 | -0.35 | | 4.55E-12 | |
| Epithelial | Epithelial | IDI1 | -0.4 | | 4.81E-12 | |
| Epithelial | Epithelial | EIF1AY | -0.37 | | 4.84E-12 | |
| Epithelial | Epithelial | SP110 | -0.3 | | 4.87E-12 | |
| Epithelial | Epithelial | SELENOF | -0.28 | | 4.93E-12 | |
| Epithelial | Epithelial | NDUFA8 | 0.25 | | 5.39E-12 | |
| Epithelial | Epithelial | TWISTNB | -0.44 | | 5.43E-12 | |
| Epithelial | Epithelial | PSMD8 | -0.28 | | 5.88E-12 | |
| Epithelial | Epithelial | INSIG1 | -0.49 | | 5.91E-12 | |
| Epithelial | Epithelial | H2AFV | -0.33 | | 6.01E-12 | |
| Epithelial | Epithelial | EIF3D | -0.28 | | 6.02E-12 | |
| Epithelial | Epithelial | PPHLN1 | -0.31 | | 7.42E-12 | |
| Epithelial | Epithelial | USP11 | -0.27 | | 8.09E-12 | |
| Epithelial | Epithelial | DDX6 | -0.3 | | 8.13E-12 | |
| Epithelial | Epithelial | GLOD4 | 0.28 | | 9.13E-12 | |
| Epithelial | Epithelial | NOP58 | -0.39 | | 9.47E-12 | |
| Epithelial | Epithelial | ZBTB1 | -0.29 | | 1.08E-11 | |
| Epithelial | Epithelial | WDR74 | -0.33 | | 1.08E-11 | |
| Epithelial | Epithelial | TMEM9B | -0.31 | | 1.11E-11 | |
| Epithelial | Epithelial | HSD17B11 | -0.27 | | 1.26E-11 | |
| Epithelial | Epithelial | GLIPR2 | -0.3 | | 1.27E-11 | |
| Epithelial | Epithelial | NEAT1 | 0.43 | | 1.37E-11 | |
| Epithelial | Epithelial | IL27RA | -0.26 | | 1.41E-11 | |
| Epithelial | Epithelial | HIF1A | -0.41 | | 2.02E-11 | |
| Epithelial | Epithelial | DDX3Y | -0.31 | | 2.03E-11 | |
| Epithelial | Epithelial | SERBP1 | -0.28 | | 2.08E-11 | |
| Epithelial | Epithelial | COX7B | 0.44 | | 2.16E-11 | |
| Epithelial | Epithelial | PIK3R1 | -0.4 | | 2.19E-11 | |
| Epithelial | Epithelial | MGAT1 | -0.3 | | 2.20E-11 | |
| Epithelial | Epithelial | SELENOM | -0.45 | | 2.35E-11 | |
| Epithelial | Epithelial | C1orf56 | -0.39 | | 2.37E-11 | |
| Epithelial | Epithelial | PIP4K2A | -0.32 | | 2.42E-11 | |
| Epithelial | Epithelial | NFATC1 | -0.25 | | 2.61E-11 | |
| Epithelial | Epithelial | LPIN1 | -0.29 | | 2.63E-11 | |
| Epithelial | Epithelial | STX5 | -0.3 | | 2.69E-11 | |
| Epithelial | Epithelial | SUPT4H1 | -0.32 | | 2.88E-11 | |
| Epithelial | Epithelial | RSL1D1 | -0.32 | | 3.02E-11 | |
| Epithelial | Epithelial | ATP2B1-AS1 | -0.3 | | 3.37E-11 | |
| Epithelial | Epithelial | PCMT1 | -0.31 | | 3.40E-11 | |
| Epithelial | Epithelial | MED10 | -0.36 | | 3.55E-11 | |
| Epithelial | Epithelial | LYN | -0.32 | | 3.87E-11 | |
| Epithelial | Epithelial | BTG3 | -0.35 | | 3.90E-11 | |
| Epithelial | Epithelial | PRDM2 | -0.3 | | 4.08E-11 | |
| Epithelial | Epithelial | ATP6V1F | -0.37 | | 4.30E-11 | |
| Epithelial | Epithelial | CMTM7 | -0.34 | | 4.64E-11 | |
| Epithelial | Epithelial | TTC39C | -0.32 | | 4.79E-11 | |
| Epithelial | Epithelial | EIF4A3 | -0.36 | | 5.10E-11 | |
| Epithelial | Epithelial | BNIP2 | -0.29 | | 5.89E-11 | |
| Epithelial | Epithelial | MED28 | -0.31 | | 5.96E-11 | |
| Epithelial | Epithelial | ICAM1 | -0.43 | | 6.84E-11 | |
| Epithelial | Epithelial | IQGAP1 | -0.34 | | 7.24E-11 | |
| Epithelial | Epithelial | PSMA1 | -0.29 | | 8.38E-11 | |
| Epithelial | Epithelial | NFKB2 | -0.29 | | 8.63E-11 | |
| Epithelial | Epithelial | JUNB | -0.46 | | 8.73E-11 | |
| Epithelial | Epithelial | MED30 | -0.3 | | 1.02E-10 | |
| Epithelial | Epithelial | CYTH1 | -0.3 | | 1.07E-10 | |
| Epithelial | Epithelial | CLIC1 | -0.28 | | 1.10E-10 | |
| Epithelial | Epithelial | CCNDBP1 | -0.33 | | 1.10E-10 | |
| Epithelial | Epithelial | CDKN1B | -0.29 | | 1.11E-10 | |
| Epithelial | Epithelial | GLA | -0.34 | | 1.12E-10 | |
| Epithelial | Epithelial | PPP1CC | -0.29 | | 1.20E-10 | |
| Epithelial | Epithelial | COX16 | 0.29 | | 1.39E-10 | |
| Epithelial | Epithelial | BCL2L11 | -0.28 | | 1.43E-10 | |
| Epithelial | Epithelial | ARRB2 | -0.28 | | 1.44E-10 | |
| Epithelial | Epithelial | BNIP3L | -0.34 | | 1.45E-10 | |
| Epithelial | Epithelial | ANXA7 | -0.28 | | 1.49E-10 | |
| Epithelial | Epithelial | JMJD1C | -0.35 | | 1.49E-10 | |
| Epithelial | Epithelial | SCAF11 | -0.34 | | 2.07E-10 | |
| Epithelial | Epithelial | ILF2 | -0.28 | | 2.07E-10 | |
| Epithelial | Epithelial | LYAR | -0.37 | | 2.29E-10 | |
| Epithelial | Epithelial | BUB3 | -0.33 | | 2.60E-10 | |
| Epithelial | Epithelial | ZNF267 | -0.25 | | 2.68E-10 | |
| Epithelial | Epithelial | IFT57 | -0.31 | | 2.76E-10 | |
| Epithelial | Epithelial | MZB1 | -1.61 | | 2.99E-10 | |
| Epithelial | Epithelial | DYNLL1 | -0.26 | | 3.20E-10 | |
| Epithelial | Epithelial | PRPF38B | -0.32 | | 3.30E-10 | |
| Epithelial | Epithelial | MATR3 | -0.33 | | 3.36E-10 | |
| Epithelial | Epithelial | SLC39A8 | -0.25 | | 3.48E-10 | |
| Epithelial | Epithelial | PDE7A | -0.29 | | 3.54E-10 | |
| Epithelial | Epithelial | ARL2BP | -0.27 | | 3.87E-10 | |
| Epithelial | Epithelial | DEGS1 | -0.35 | | 4.06E-10 | |
| Epithelial | Epithelial | RTN4 | -0.35 | | 4.25E-10 | |
| Epithelial | Epithelial | CCDC85B | -0.35 | | 4.66E-10 | |
| Epithelial | Epithelial | MED4 | -0.31 | | 4.73E-10 | |
| Epithelial | Epithelial | CHST12 | -0.33 | | 5.42E-10 | |
| Epithelial | Epithelial | IGLL5 | -2.68 | | 6.04E-10 | |
| Epithelial | Epithelial | PPIG | -0.33 | | 6.10E-10 | |
| Epithelial | Epithelial | RAB5C | -0.27 | | 6.21E-10 | |
| Epithelial | Epithelial | PCNP | -0.29 | | 6.22E-10 | |
| Epithelial | Epithelial | PTPN2 | -0.3 | | 7.07E-10 | |
| Epithelial | Epithelial | GK | -0.27 | | 7.37E-10 | |
| Epithelial | Epithelial | HOPX | -0.47 | | 8.15E-10 | |
| Epithelial | Epithelial | CTSL | -0.68 | | 8.21E-10 | |
| Epithelial | Epithelial | PHF20 | -0.31 | | 8.22E-10 | |
| Epithelial | Epithelial | PMEPA1 | -0.28 | | 9.02E-10 | |
| Epithelial | Epithelial | IFNGR1 | -0.27 | | 1.02E-09 | |
| Epithelial | Epithelial | COX5A | 0.34 | | 1.09E-09 | |
| Epithelial | Epithelial | ARL6IP4 | 0.25 | | 1.12E-09 | |
| Epithelial | Epithelial | IER2 | 0.27 | | 1.24E-09 | |
| Epithelial | Epithelial | CDC42EP3 | -0.3 | | 1.50E-09 | |
| Epithelial | Epithelial | ABT1 | -0.28 | | 1.55E-09 | |
| Epithelial | Epithelial | LAMTOR4 | 0.25 | | 1.57E-09 | |
| Epithelial | Epithelial | NFKB1 | -0.35 | | 1.59E-09 | |
| Epithelial | Epithelial | WDR33 | -0.28 | | 1.78E-09 | |
| Epithelial | Epithelial | EIF4H | -0.3 | | 1.81E-09 | |
| Epithelial | Epithelial | CMC2 | -0.31 | | 1.87E-09 | |
| Epithelial | Epithelial | HNRNPU | -0.31 | | 1.91E-09 | |
| Epithelial | Epithelial | RBMS1 | -0.28 | | 1.94E-09 | |
| Epithelial | Epithelial | HECA | -0.27 | | 1.97E-09 | |
| Epithelial | Epithelial | MRPL41 | 0.28 | | 1.97E-09 | |
| Epithelial | Epithelial | C4orf48 | -0.26 | | 2.11E-09 | |
| Epithelial | Epithelial | SPCS1 | -0.36 | | 2.22E-09 | |
| Epithelial | Epithelial | PHF20L1 | -0.27 | | 2.28E-09 | |
| Epithelial | Epithelial | RNF139 | -0.31 | | 2.32E-09 | |
| Epithelial | Epithelial | ZNF394 | -0.26 | | 2.49E-09 | |
| Epithelial | Epithelial | SPCS3 | -0.46 | | 2.54E-09 | |
| Epithelial | Epithelial | SEC14L1 | -0.25 | | 2.60E-09 | |
| Epithelial | Epithelial | ARPC4 | -0.3 | | 2.69E-09 | |
| Epithelial | Epithelial | DNAJC9 | -0.26 | | 2.84E-09 | |
| Epithelial | Epithelial | UBE2L3 | -0.27 | | 2.86E-09 | |
| Epithelial | Epithelial | OSBPL8 | -0.28 | | 3.23E-09 | |
| Epithelial | Epithelial | GGA2 | -0.27 | | 3.30E-09 | |
| Epithelial | Epithelial | SAFB2 | -0.29 | | 3.30E-09 | |
| Epithelial | Epithelial | TACC1 | -0.28 | | 3.43E-09 | |
| Epithelial | Epithelial | NR4A3 | -0.33 | | 3.61E-09 | |
| Epithelial | Epithelial | PPP1R12A | -0.28 | | 4.56E-09 | |
| Epithelial | Epithelial | ANP32E | -0.27 | | 4.65E-09 | |
| Epithelial | Epithelial | MBP | -0.31 | | 4.72E-09 | |
| Epithelial | Epithelial | RASGEF1B | -0.34 | | 4.79E-09 | |
| Epithelial | Epithelial | EIF1AX | -0.26 | | 5.35E-09 | |
| Epithelial | Epithelial | SFPQ | -0.27 | | 5.37E-09 | |
| Epithelial | Epithelial | SIAH2 | -0.26 | | 5.40E-09 | |
| Epithelial | Epithelial | ACP5 | -0.35 | | 5.66E-09 | |
| Epithelial | Epithelial | ARHGEF1 | -0.32 | | 5.90E-09 | |
| Epithelial | Epithelial | SAR1A | -0.28 | | 6.17E-09 | |
| Epithelial | Epithelial | SURF4 | -0.29 | | 6.58E-09 | |
| Epithelial | Epithelial | YIPF5 | -0.28 | | 6.66E-09 | |
| Epithelial | Epithelial | APMAP | -0.29 | | 6.86E-09 | |
| Epithelial | Epithelial | EIF5 | -0.31 | | 7.24E-09 | |
| Epithelial | Epithelial | MYH9 | -0.32 | | 7.29E-09 | |
| Epithelial | Epithelial | TNFAIP8 | -0.31 | | 7.40E-09 | |
| Epithelial | Epithelial | ATP6V0D1 | -0.29 | | 7.79E-09 | |
| Epithelial | Epithelial | TMEM173 | -0.28 | | 7.94E-09 | |
| Epithelial | Epithelial | PPT1 | -0.29 | | 8.41E-09 | |
| Epithelial | Epithelial | M6PR | -0.27 | | 8.80E-09 | |
| Epithelial | Epithelial | TNIP1 | -0.26 | | 9.19E-09 | |
| Epithelial | Epithelial | DUSP5 | -0.47 | | 9.75E-09 | |
| Epithelial | Epithelial | CNN2 | -0.31 | | 1.10E-08 | |
| Epithelial | Epithelial | TNRC6B | -0.29 | | 1.12E-08 | |
| Epithelial | Epithelial | SNRPB2 | -0.27 | | 1.15E-08 | |
| Epithelial | Epithelial | COX6C | 0.26 | | 1.29E-08 | |
| Epithelial | Epithelial | RBM38 | -0.27 | | 1.30E-08 | |
| Epithelial | Epithelial | HMGN2 | -0.26 | | 1.34E-08 | |
| Epithelial | Epithelial | CHRAC1 | -0.26 | | 1.42E-08 | |
| Epithelial | Epithelial | ATP5MF | 0.25 | | 1.44E-08 | |
| Epithelial | Epithelial | AP3S1 | -0.3 | | 1.53E-08 | |
| Epithelial | Epithelial | HLA-DRB1 | -0.97 | | 1.68E-08 | |
| Epithelial | Epithelial | TMEM176B | -0.36 | | 1.71E-08 | |
| Epithelial | Epithelial | SLC25A4 | 0.31 | | 1.76E-08 | |
| Epithelial | Epithelial | RABIF | -0.27 | | 1.82E-08 | |
| Epithelial | Epithelial | DR1 | -0.26 | | 1.85E-08 | |
| Epithelial | Epithelial | KHDRBS1 | -0.26 | | 1.86E-08 | |
| Epithelial | Epithelial | CIAO2B | -0.26 | | 1.87E-08 | |
| Epithelial | Epithelial | ATP5PD | 0.27 | | 1.91E-08 | |
| Epithelial | Epithelial | RNF138 | -0.26 | | 2.04E-08 | |
| Epithelial | Epithelial | AKIRIN1 | -0.32 | | 2.07E-08 | |
| Epithelial | Epithelial | JOSD1 | -0.27 | | 2.07E-08 | |
| Epithelial | Epithelial | PLEKHA2 | -0.25 | | 2.21E-08 | |
| Epithelial | Epithelial | CSNK1A1 | -0.27 | | 2.35E-08 | |
| Epithelial | Epithelial | COX8A | 0.27 | | 2.39E-08 | |
| Epithelial | Epithelial | SERINC1 | -0.28 | | 3.10E-08 | |
| Epithelial | Epithelial | BHLHE40 | -0.38 | | 3.51E-08 | |
| Epithelial | Epithelial | PDE4A | -0.25 | | 3.79E-08 | |
| Epithelial | Epithelial | BCLAF1 | -0.32 | | 3.85E-08 | |
| Epithelial | Epithelial | PRCP | -0.26 | | 4.14E-08 | |
| Epithelial | Epithelial | ZNF706 | -0.26 | | 4.18E-08 | |
| Epithelial | Epithelial | TMEM165 | -0.26 | | 4.28E-08 | |
| Epithelial | Epithelial | POLR2K | -0.26 | | 4.34E-08 | |
| Epithelial | Epithelial | APOBEC3C | -0.25 | | 4.61E-08 | |
| Epithelial | Epithelial | ATP5PO | 0.37 | | 4.76E-08 | |
| Epithelial | Epithelial | DNAJC3 | -0.27 | | 4.95E-08 | |
| Epithelial | Epithelial | HNRNPH3 | -0.29 | | 5.02E-08 | |
| Epithelial | Epithelial | WBP11 | -0.29 | | 5.03E-08 | |
| Epithelial | Epithelial | NDUFB9 | 0.25 | | 5.40E-08 | |
| Epithelial | Epithelial | IL1B | -1.52 | | 5.57E-08 | |
| Epithelial | Epithelial | RAB9A | -0.29 | | 6.01E-08 | |
| Epithelial | Epithelial | RARRES3 | -0.35 | | 6.23E-08 | |
| Epithelial | Epithelial | DHX36 | -0.3 | | 6.40E-08 | |
| Epithelial | Epithelial | DERL3 | -0.99 | | 7.29E-08 | |
| Epithelial | Epithelial | ATG12 | -0.28 | | 7.35E-08 | |
| Epithelial | Epithelial | ANXA5 | -0.44 | | 8.03E-08 | |
| Epithelial | Epithelial | AC020916.1 | -0.29 | | 8.48E-08 | |
| Epithelial | Epithelial | TRAPPC1 | -0.28 | | 8.81E-08 | |
| Epithelial | Epithelial | YTHDF2 | -0.27 | | 8.84E-08 | |
| Epithelial | Epithelial | MRFAP1 | -0.28 | | 9.39E-08 | |
| Epithelial | Epithelial | PITHD1 | -0.29 | | 1.01E-07 | |
| Epithelial | Epithelial | RBPJ | -0.32 | | 1.15E-07 | |
| Epithelial | Epithelial | JMJD6 | -0.3 | | 1.25E-07 | |
| Epithelial | Epithelial | PDE4D | -0.3 | | 1.27E-07 | |
| Epithelial | Epithelial | DDX27 | -0.27 | | 1.39E-07 | |
| Epithelial | Epithelial | CTNNB1 | -0.34 | | 1.43E-07 | |
| Epithelial | Epithelial | ELOC | -0.26 | | 1.52E-07 | |
| Epithelial | Epithelial | SC5D | -0.35 | | 1.63E-07 | |
| Epithelial | Epithelial | XRN2 | -0.26 | | 1.67E-07 | |
| Epithelial | Epithelial | DDIT4 | -0.47 | | 1.82E-07 | |
| Epithelial | Epithelial | BATF | -0.42 | | 1.85E-07 | |
| Epithelial | Epithelial | THAP2 | -0.28 | | 1.90E-07 | |
| Epithelial | Epithelial | C15orf48 | -0.55 | | 1.92E-07 | |
| Epithelial | Epithelial | LYST | -0.32 | | 1.94E-07 | |
| Epithelial | Epithelial | IK | -0.25 | | 2.03E-07 | |
| Epithelial | Epithelial | LMAN2 | -0.29 | | 2.09E-07 | |
| Epithelial | Epithelial | CACYBP | -0.35 | | 2.26E-07 | |
| Epithelial | Epithelial | CITED2 | -0.48 | | 2.67E-07 | |
| Epithelial | Epithelial | SNHG15 | -0.26 | | 2.74E-07 | |
| Epithelial | Epithelial | NAA50 | -0.29 | | 3.44E-07 | |
| Epithelial | Epithelial | RAB5IF | -0.26 | | 3.54E-07 | |
| Epithelial | Epithelial | RNF19A | -0.31 | | 3.96E-07 | |
| Epithelial | Epithelial | GSPT1 | -0.29 | | 4.68E-07 | |
| Epithelial | Epithelial | CSNK1D | -0.27 | | 5.51E-07 | |
| Epithelial | Epithelial | IGFBP4 | -0.48 | | 5.78E-07 | |
| Epithelial | Epithelial | FKBP1A | -0.32 | | 7.14E-07 | |
| Epithelial | Epithelial | LBH | -0.25 | | 7.88E-07 | |
| Epithelial | Epithelial | CD68 | -0.41 | | 8.70E-07 | |
| Epithelial | Epithelial | RBM25 | -0.25 | | 9.01E-07 | |
| Epithelial | Epithelial | APOC1 | -0.48 | | 9.69E-07 | |
| Epithelial | Epithelial | RABAC1 | -0.28 | | 1.23E-06 | |
| Epithelial | Epithelial | TBCB | -0.26 | | 1.43E-06 | |
| Epithelial | Epithelial | MEAF6 | -0.29 | | 1.65E-06 | |
| Epithelial | Epithelial | HNRNPH1 | -0.3 | | 1.66E-06 | |
| Epithelial | Epithelial | TRA2B | -0.25 | | 1.70E-06 | |
| Epithelial | Epithelial | COX7C | 0.27 | | 1.73E-06 | |
| Epithelial | Epithelial | WSB1 | -0.26 | | 1.84E-06 | |
| Epithelial | Epithelial | CBLB | -0.25 | | 1.95E-06 | |
| Epithelial | Epithelial | CHD2 | -0.27 | | 2.87E-06 | |
| Epithelial | Epithelial | RBM23 | -0.27 | | 3.18E-06 | |
| Epithelial | Epithelial | AMD1 | -0.3 | | 3.40E-06 | |
| Epithelial | Epithelial | TMED10 | -0.25 | | 3.51E-06 | |
| Epithelial | Epithelial | CCDC59 | -0.27 | | 3.85E-06 | |
| Epithelial | Epithelial | IRF1 | -0.25 | | 6.81E-06 | |
| Epithelial | Epithelial | OGA | -0.25 | | 7.40E-06 | |
| Epithelial | Epithelial | PSMA3-AS1 | -0.27 | | 7.79E-06 | |
| Epithelial | Epithelial | S100A10 | -0.42 | | 8.60E-06 | |
| Epithelial | Epithelial | IFRD1 | -0.28 | | 9.07E-06 | |
| Fibroblasts | Fibroblasts | DCN | 3.83 | | 0 | |
| Fibroblasts | Fibroblasts | MGP | 3.67 | | 0 | |
| Fibroblasts | Fibroblasts | APOD | 3.59 | | 0 | |
| Fibroblasts | Fibroblasts | LUM | 3.47 | | 0 | |
| Fibroblasts | Fibroblasts | IGFBP7 | 3.33 | | 0 | |
| Fibroblasts | Fibroblasts | TAGLN | 3.2 | | 0 | |
| Fibroblasts | Fibroblasts | COL1A2 | 3.13 | | 0 | |
| Fibroblasts | Fibroblasts | COL1A1 | 3.11 | | 0 | |
| Fibroblasts | Fibroblasts | CFD | 3.07 | | 0 | |
| Fibroblasts | Fibroblasts | COL3A1 | 3.02 | | 0 | |
| Fibroblasts | Fibroblasts | CCL2 | 2.84 | | 0 | |
| Fibroblasts | Fibroblasts | FBLN1 | 2.79 | | 0 | |
| Fibroblasts | Fibroblasts | ACTA2 | 2.74 | | 0 | |
| Fibroblasts | Fibroblasts | C1S | 2.72 | | 0 | |
| Fibroblasts | Fibroblasts | PTGDS | 2.71 | | 0 | |
| Fibroblasts | Fibroblasts | C1R | 2.68 | | 0 | |
| Fibroblasts | Fibroblasts | CALD1 | 2.63 | | 0 | |
| Fibroblasts | Fibroblasts | MYL9 | 2.61 | | 0 | |
| Fibroblasts | Fibroblasts | SERPINF1 | 2.59 | | 0 | |
| Fibroblasts | Fibroblasts | RARRES2 | 2.58 | | 0 | |
| Fibroblasts | Fibroblasts | GSN | 2.57 | | 0 | |
| Fibroblasts | Fibroblasts | MFAP4 | 2.46 | | 0 | |
| Fibroblasts | Fibroblasts | SERPING1 | 2.44 | | 0 | |
| Fibroblasts | Fibroblasts | APOE | 2.44 | | 0 | |
| Fibroblasts | Fibroblasts | CXCL14 | 2.42 | | 0 | |
| Fibroblasts | Fibroblasts | SPARC | 2.42 | | 0 | |
| Fibroblasts | Fibroblasts | CCDC80 | 2.41 | | 0 | |
| Fibroblasts | Fibroblasts | SOD3 | 2.41 | | 0 | |
| Fibroblasts | Fibroblasts | BGN | 2.39 | | 0 | |
| Fibroblasts | Fibroblasts | COL6A2 | 2.36 | | 0 | |
| Fibroblasts | Fibroblasts | C11orf96 | 2.31 | | 0 | |
| Fibroblasts | Fibroblasts | EGR1 | 2.28 | | 0 | |
| Fibroblasts | Fibroblasts | C7 | 2.26 | | 0 | |
| Fibroblasts | Fibroblasts | CCL11 | 2.2 | | 0 | |
| Fibroblasts | Fibroblasts | IGFBP5 | 2.17 | | 0 | |
| Fibroblasts | Fibroblasts | TPM2 | 2.15 | | 0 | |
| Fibroblasts | Fibroblasts | C3 | 2.08 | | 0 | |
| Fibroblasts | Fibroblasts | IFITM3 | 2.05 | | 0 | |
| Fibroblasts | Fibroblasts | CYR61 | 2.04 | | 0 | |
| Fibroblasts | Fibroblasts | IGFBP6 | 2 | | 0 | |
| Fibroblasts | Fibroblasts | DPT | 1.98 | | 0 | |
| Fibroblasts | Fibroblasts | SFRP2 | 1.97 | | 0 | |
| Fibroblasts | Fibroblasts | MMP2 | 1.92 | | 0 | |
| Fibroblasts | Fibroblasts | IGFBP4 | 1.92 | | 0 | |
| Fibroblasts | Fibroblasts | COL6A1 | 1.92 | | 0 | |
| Fibroblasts | Fibroblasts | CTGF | 1.9 | | 0 | |
| Fibroblasts | Fibroblasts | RGS5 | 1.9 | | 0 | |
| Fibroblasts | Fibroblasts | SPARCL1 | 1.9 | | 0 | |
| Fibroblasts | Fibroblasts | LTBP4 | 1.88 | | 0 | |
| Fibroblasts | Fibroblasts | NNMT | 1.86 | | 0 | |
| Fibroblasts | Fibroblasts | GEM | 1.84 | | 0 | |
| Fibroblasts | Fibroblasts | PCOLCE | 1.82 | | 0 | |
| Fibroblasts | Fibroblasts | GPC3 | 1.79 | | 0 | |
| Fibroblasts | Fibroblasts | EFEMP1 | 1.78 | | 0 | |
| Fibroblasts | Fibroblasts | CFH | 1.78 | | 0 | |
| Fibroblasts | Fibroblasts | PLAC9 | 1.77 | | 0 | |
| Fibroblasts | Fibroblasts | THY1 | 1.74 | | 0 | |
| Fibroblasts | Fibroblasts | SPON2 | 1.67 | | 0 | |
| Fibroblasts | Fibroblasts | CST3 | 1.65 | | 0 | |
| Fibroblasts | Fibroblasts | SELENOM | 1.63 | | 0 | |
| Fibroblasts | Fibroblasts | FRZB | 1.63 | | 0 | |
| Fibroblasts | Fibroblasts | COL6A3 | 1.6 | | 0 | |
| Fibroblasts | Fibroblasts | TMEM176B | 1.59 | | 0 | |
| Fibroblasts | Fibroblasts | LHFPL6 | 1.58 | | 0 | |
| Fibroblasts | Fibroblasts | TPM1 | 1.58 | | 0 | |
| Fibroblasts | Fibroblasts | A2M | 1.56 | | 0 | |
| Fibroblasts | Fibroblasts | MFGE8 | 1.55 | | 0 | |
| Fibroblasts | Fibroblasts | FSTL1 | 1.53 | | 0 | |
| Fibroblasts | Fibroblasts | GGT5 | 1.51 | | 0 | |
| Fibroblasts | Fibroblasts | CEBPD | 1.51 | | 0 | |
| Fibroblasts | Fibroblasts | CNN3 | 1.49 | | 0 | |
| Fibroblasts | Fibroblasts | NUPR1 | 1.48 | | 0 | |
| Fibroblasts | Fibroblasts | CXCL12 | 1.47 | | 0 | |
| Fibroblasts | Fibroblasts | POSTN | 1.47 | | 0 | |
| Fibroblasts | Fibroblasts | TIMP3 | 1.47 | | 0 | |
| Fibroblasts | Fibroblasts | MEG3 | 1.46 | | 0 | |
| Fibroblasts | Fibroblasts | LGALS3BP | 1.45 | | 0 | |
| Fibroblasts | Fibroblasts | TIMP1 | 1.45 | | 0 | |
| Fibroblasts | Fibroblasts | AEBP1 | 1.44 | | 0 | |
| Fibroblasts | Fibroblasts | CRISPLD2 | 1.43 | | 0 | |
| Fibroblasts | Fibroblasts | CAVIN3 | 1.4 | | 0 | |
| Fibroblasts | Fibroblasts | CPE | 1.4 | | 0 | |
| Fibroblasts | Fibroblasts | ABCA8 | 1.38 | | 0 | |
| Fibroblasts | Fibroblasts | CTSK | 1.37 | | 0 | |
| Fibroblasts | Fibroblasts | ADAMTS1 | 1.35 | | 0 | |
| Fibroblasts | Fibroblasts | FXYD1 | 1.35 | | 0 | |
| Fibroblasts | Fibroblasts | OGN | 1.35 | | 0 | |
| Fibroblasts | Fibroblasts | CAVIN1 | 1.33 | | 0 | |
| Fibroblasts | Fibroblasts | EMILIN1 | 1.32 | | 0 | |
| Fibroblasts | Fibroblasts | GPX3 | 1.32 | | 0 | |
| Fibroblasts | Fibroblasts | ADH1B | 1.32 | | 0 | |
| Fibroblasts | Fibroblasts | THBS4 | 1.29 | | 0 | |
| Fibroblasts | Fibroblasts | MFAP5 | 1.29 | | 0 | |
| Fibroblasts | Fibroblasts | NBL1 | 1.26 | | 0 | |
| Fibroblasts | Fibroblasts | FN1 | 1.25 | | 0 | |
| Fibroblasts | Fibroblasts | ASPN | 1.25 | | 0 | |
| Fibroblasts | Fibroblasts | PMP22 | 1.24 | | 0 | |
| Fibroblasts | Fibroblasts | TMEM176A | 1.23 | | 0 | |
| Fibroblasts | Fibroblasts | FHL1 | 1.22 | | 0 | |
| Fibroblasts | Fibroblasts | TIMP2 | 1.21 | | 0 | |
| Fibroblasts | Fibroblasts | PLTP | 1.2 | | 0 | |
| Fibroblasts | Fibroblasts | BEX3 | 1.19 | | 0 | |
| Fibroblasts | Fibroblasts | NDUFA4L2 | 1.19 | | 0 | |
| Fibroblasts | Fibroblasts | SELENOP | 1.19 | | 0 | |
| Fibroblasts | Fibroblasts | IGFBP3 | 1.19 | | 0 | |
| Fibroblasts | Fibroblasts | MXRA8 | 1.19 | | 0 | |
| Fibroblasts | Fibroblasts | TCEAL9 | 1.18 | | 0 | |
| Fibroblasts | Fibroblasts | COL18A1 | 1.17 | | 0 | |
| Fibroblasts | Fibroblasts | S100A13 | 1.17 | | 0 | |
| Fibroblasts | Fibroblasts | FBLN5 | 1.17 | | 0 | |
| Fibroblasts | Fibroblasts | CRYAB | 1.16 | | 0 | |
| Fibroblasts | Fibroblasts | SDC2 | 1.15 | | 0 | |
| Fibroblasts | Fibroblasts | PDLIM3 | 1.14 | | 0 | |
| Fibroblasts | Fibroblasts | VCAN | 1.13 | | 0 | |
| Fibroblasts | Fibroblasts | PDGFRB | 1.13 | | 0 | |
| Fibroblasts | Fibroblasts | SERPINH1 | 1.13 | | 0 | |
| Fibroblasts | Fibroblasts | AGT | 1.08 | | 0 | |
| Fibroblasts | Fibroblasts | CYGB | 1.08 | | 0 | |
| Fibroblasts | Fibroblasts | TFPI | 1.08 | | 0 | |
| Fibroblasts | Fibroblasts | CLEC11A | 1.08 | | 0 | |
| Fibroblasts | Fibroblasts | COX7A1 | 1.06 | | 0 | |
| Fibroblasts | Fibroblasts | THBS1 | 1.06 | | 0 | |
| Fibroblasts | Fibroblasts | FGF7 | 1.05 | | 0 | |
| Fibroblasts | Fibroblasts | RND3 | 1.05 | | 0 | |
| Fibroblasts | Fibroblasts | CAV1 | 1.04 | | 0 | |
| Fibroblasts | Fibroblasts | CYBRD1 | 1.04 | | 0 | |
| Fibroblasts | Fibroblasts | PLAT | 1.04 | | 0 | |
| Fibroblasts | Fibroblasts | MATN2 | 1.04 | | 0 | |
| Fibroblasts | Fibroblasts | GPNMB | 1.03 | | 0 | |
| Fibroblasts | Fibroblasts | PPIC | 1.02 | | 0 | |
| Fibroblasts | Fibroblasts | EFEMP2 | 1.01 | | 0 | |
| Fibroblasts | Fibroblasts | DKK3 | 1.01 | | 0 | |
| Fibroblasts | Fibroblasts | PTMS | 1.01 | | 0 | |
| Fibroblasts | Fibroblasts | HTRA3 | 1.01 | | 0 | |
| Fibroblasts | Fibroblasts | SFRP4 | 1.01 | | 0 | |
| Fibroblasts | Fibroblasts | NR2F2 | 1 | | 0 | |
| Fibroblasts | Fibroblasts | SGCE | 0.99 | | 0 | |
| Fibroblasts | Fibroblasts | NFIA | 0.99 | | 0 | |
| Fibroblasts | Fibroblasts | SMOC2 | 0.98 | | 0 | |
| Fibroblasts | Fibroblasts | SRPX | 0.97 | | 0 | |
| Fibroblasts | Fibroblasts | COL4A1 | 0.97 | | 0 | |
| Fibroblasts | Fibroblasts | OLFML3 | 0.97 | | 0 | |
| Fibroblasts | Fibroblasts | CSRP2 | 0.96 | | 0 | |
| Fibroblasts | Fibroblasts | APP | 0.96 | | 0 | |
| Fibroblasts | Fibroblasts | TNXB | 0.95 | | 0 | |
| Fibroblasts | Fibroblasts | COL5A1 | 0.95 | | 0 | |
| Fibroblasts | Fibroblasts | NFIB | 0.94 | | 0 | |
| Fibroblasts | Fibroblasts | NOTCH3 | 0.94 | | 0 | |
| Fibroblasts | Fibroblasts | PTN | 0.94 | | 0 | |
| Fibroblasts | Fibroblasts | MYLK | 0.93 | | 0 | |
| Fibroblasts | Fibroblasts | COL4A2 | 0.93 | | 0 | |
| Fibroblasts | Fibroblasts | LRP1 | 0.93 | | 0 | |
| Fibroblasts | Fibroblasts | MAFB | 0.93 | | 0 | |
| Fibroblasts | Fibroblasts | ADAMTS4 | 0.92 | | 0 | |
| Fibroblasts | Fibroblasts | TSPAN4 | 0.92 | | 0 | |
| Fibroblasts | Fibroblasts | EMP2 | 0.91 | | 0 | |
| Fibroblasts | Fibroblasts | COL5A2 | 0.89 | | 0 | |
| Fibroblasts | Fibroblasts | PRSS23 | 0.88 | | 0 | |
| Fibroblasts | Fibroblasts | PRELP | 0.88 | | 0 | |
| Fibroblasts | Fibroblasts | COL14A1 | 0.87 | | 0 | |
| Fibroblasts | Fibroblasts | SERPINE2 | 0.87 | | 0 | |
| Fibroblasts | Fibroblasts | NDN | 0.86 | | 0 | |
| Fibroblasts | Fibroblasts | GNG11 | 0.86 | | 0 | |
| Fibroblasts | Fibroblasts | NDRG2 | 0.86 | | 0 | |
| Fibroblasts | Fibroblasts | EPHX1 | 0.86 | | 0 | |
| Fibroblasts | Fibroblasts | PI16 | 0.85 | | 0 | |
| Fibroblasts | Fibroblasts | PDGFRA | 0.85 | | 0 | |
| Fibroblasts | Fibroblasts | NFIC | 0.83 | | 0 | |
| Fibroblasts | Fibroblasts | HTRA1 | 0.83 | | 0 | |
| Fibroblasts | Fibroblasts | PLPP3 | 0.82 | | 0 | |
| Fibroblasts | Fibroblasts | FXYD6 | 0.82 | | 0 | |
| Fibroblasts | Fibroblasts | MAP1B | 0.82 | | 0 | |
| Fibroblasts | Fibroblasts | TMEM98 | 0.82 | | 0 | |
| Fibroblasts | Fibroblasts | TCEAL4 | 0.81 | | 0 | |
| Fibroblasts | Fibroblasts | PODN | 0.81 | | 0 | |
| Fibroblasts | Fibroblasts | ECM1 | 0.81 | | 0 | |
| Fibroblasts | Fibroblasts | TCF21 | 0.81 | | 0 | |
| Fibroblasts | Fibroblasts | LAMA4 | 0.81 | | 0 | |
| Fibroblasts | Fibroblasts | TGFB1I1 | 0.81 | | 0 | |
| Fibroblasts | Fibroblasts | ACTG2 | 0.81 | | 0 | |
| Fibroblasts | Fibroblasts | RBP1 | 0.81 | | 0 | |
| Fibroblasts | Fibroblasts | OLFML2B | 0.8 | | 0 | |
| Fibroblasts | Fibroblasts | RAB34 | 0.79 | | 0 | |
| Fibroblasts | Fibroblasts | HSPB6 | 0.79 | | 0 | |
| Fibroblasts | Fibroblasts | GUCY1A1 | 0.78 | | 0 | |
| Fibroblasts | Fibroblasts | CYP1B1 | 0.78 | | 0 | |
| Fibroblasts | Fibroblasts | ID4 | 0.78 | | 0 | |
| Fibroblasts | Fibroblasts | CPQ | 0.77 | | 0 | |
| Fibroblasts | Fibroblasts | EPB41L2 | 0.76 | | 0 | |
| Fibroblasts | Fibroblasts | RCN3 | 0.75 | | 0 | |
| Fibroblasts | Fibroblasts | DAB2 | 0.75 | | 0 | |
| Fibroblasts | Fibroblasts | CTSF | 0.74 | | 0 | |
| Fibroblasts | Fibroblasts | FBN1 | 0.74 | | 0 | |
| Fibroblasts | Fibroblasts | PALLD | 0.73 | | 0 | |
| Fibroblasts | Fibroblasts | FILIP1L | 0.72 | | 0 | |
| Fibroblasts | Fibroblasts | PROCR | 0.72 | | 0 | |
| Fibroblasts | Fibroblasts | AC245595.1 | 0.71 | | 0 | |
| Fibroblasts | Fibroblasts | COL15A1 | 0.71 | | 0 | |
| Fibroblasts | Fibroblasts | FKBP10 | 0.71 | | 0 | |
| Fibroblasts | Fibroblasts | AXL | 0.71 | | 0 | |
| Fibroblasts | Fibroblasts | NID1 | 0.7 | | 0 | |
| Fibroblasts | Fibroblasts | ELN | 0.7 | | 0 | |
| Fibroblasts | Fibroblasts | AKAP12 | 0.7 | | 0 | |
| Fibroblasts | Fibroblasts | LAMB2 | 0.69 | | 0 | |
| Fibroblasts | Fibroblasts | PDPN | 0.69 | | 0 | |
| Fibroblasts | Fibroblasts | ISLR | 0.69 | | 0 | |
| Fibroblasts | Fibroblasts | BMP4 | 0.68 | | 0 | |
| Fibroblasts | Fibroblasts | NEXN | 0.67 | | 0 | |
| Fibroblasts | Fibroblasts | MRC2 | 0.67 | | 0 | |
| Fibroblasts | Fibroblasts | SSPN | 0.66 | | 0 | |
| Fibroblasts | Fibroblasts | RTL8C | 0.66 | | 0 | |
| Fibroblasts | Fibroblasts | CD302 | 0.66 | | 0 | |
| Fibroblasts | Fibroblasts | GLT8D2 | 0.65 | | 0 | |
| Fibroblasts | Fibroblasts | FGFR1 | 0.65 | | 0 | |
| Fibroblasts | Fibroblasts | BICC1 | 0.64 | | 0 | |
| Fibroblasts | Fibroblasts | VASN | 0.64 | | 0 | |
| Fibroblasts | Fibroblasts | MMP23B | 0.63 | | 0 | |
| Fibroblasts | Fibroblasts | WFDC1 | 0.63 | | 0 | |
| Fibroblasts | Fibroblasts | SLIT3 | 0.62 | | 0 | |
| Fibroblasts | Fibroblasts | PLN | 0.61 | | 0 | |
| Fibroblasts | Fibroblasts | EHD2 | 0.61 | | 0 | |
| Fibroblasts | Fibroblasts | CNN1 | 0.61 | | 0 | |
| Fibroblasts | Fibroblasts | FBLN2 | 0.61 | | 0 | |
| Fibroblasts | Fibroblasts | MSC | 0.61 | | 0 | |
| Fibroblasts | Fibroblasts | PRRX1 | 0.6 | | 0 | |
| Fibroblasts | Fibroblasts | ABI3BP | 0.6 | | 0 | |
| Fibroblasts | Fibroblasts | SVIL | 0.6 | | 0 | |
| Fibroblasts | Fibroblasts | PLXDC1 | 0.6 | | 0 | |
| Fibroblasts | Fibroblasts | DDR2 | 0.6 | | 0 | |
| Fibroblasts | Fibroblasts | CD248 | 0.59 | | 0 | |
| Fibroblasts | Fibroblasts | FMOD | 0.58 | | 0 | |
| Fibroblasts | Fibroblasts | FOXS1 | 0.57 | | 0 | |
| Fibroblasts | Fibroblasts | KCNMB1 | 0.57 | | 0 | |
| Fibroblasts | Fibroblasts | C1QTNF3 | 0.56 | | 0 | |
| Fibroblasts | Fibroblasts | FSTL3 | 0.56 | | 0 | |
| Fibroblasts | Fibroblasts | LTBP1 | 0.56 | | 0 | |
| Fibroblasts | Fibroblasts | LAMC1 | 0.55 | | 0 | |
| Fibroblasts | Fibroblasts | PDLIM4 | 0.55 | | 0 | |
| Fibroblasts | Fibroblasts | COL8A1 | 0.55 | | 0 | |
| Fibroblasts | Fibroblasts | FERMT2 | 0.55 | | 0 | |
| Fibroblasts | Fibroblasts | ALDH1A3 | 0.55 | | 0 | |
| Fibroblasts | Fibroblasts | COL12A1 | 0.55 | | 0 | |
| Fibroblasts | Fibroblasts | PBX1 | 0.55 | | 0 | |
| Fibroblasts | Fibroblasts | ANGPTL2 | 0.55 | | 0 | |
| Fibroblasts | Fibroblasts | RBMS3 | 0.55 | | 0 | |
| Fibroblasts | Fibroblasts | LOXL1 | 0.54 | | 0 | |
| Fibroblasts | Fibroblasts | CDH11 | 0.54 | | 0 | |
| Fibroblasts | Fibroblasts | EDNRB | 0.54 | | 0 | |
| Fibroblasts | Fibroblasts | TCEAL7 | 0.54 | | 0 | |
| Fibroblasts | Fibroblasts | TMEM47 | 0.53 | | 0 | |
| Fibroblasts | Fibroblasts | THBS2 | 0.53 | | 0 | |
| Fibroblasts | Fibroblasts | TMEM119 | 0.53 | | 0 | |
| Fibroblasts | Fibroblasts | OMD | 0.53 | | 0 | |
| Fibroblasts | Fibroblasts | DIO2 | 0.52 | | 0 | |
| Fibroblasts | Fibroblasts | STEAP1 | 0.52 | | 0 | |
| Fibroblasts | Fibroblasts | PHLDB1 | 0.52 | | 0 | |
| Fibroblasts | Fibroblasts | SGCB | 0.52 | | 0 | |
| Fibroblasts | Fibroblasts | ECM2 | 0.52 | | 0 | |
| Fibroblasts | Fibroblasts | TWSG1 | 0.52 | | 0 | |
| Fibroblasts | Fibroblasts | ANTXR1 | 0.52 | | 0 | |
| Fibroblasts | Fibroblasts | PFN2 | 0.51 | | 0 | |
| Fibroblasts | Fibroblasts | NR2F1 | 0.51 | | 0 | |
| Fibroblasts | Fibroblasts | MXRA5 | 0.51 | | 0 | |
| Fibroblasts | Fibroblasts | PID1 | 0.5 | | 0 | |
| Fibroblasts | Fibroblasts | FOXF1 | 0.5 | | 0 | |
| Fibroblasts | Fibroblasts | IGF2 | 0.5 | | 0 | |
| Fibroblasts | Fibroblasts | PDGFRL | 0.5 | | 0 | |
| Fibroblasts | Fibroblasts | LRRC32 | 0.5 | | 0 | |
| Fibroblasts | Fibroblasts | NGFR | 0.49 | | 0 | |
| Fibroblasts | Fibroblasts | BDKRB1 | 0.49 | | 0 | |
| Fibroblasts | Fibroblasts | FKBP7 | 0.49 | | 0 | |
| Fibroblasts | Fibroblasts | GPX8 | 0.49 | | 0 | |
| Fibroblasts | Fibroblasts | KANK2 | 0.48 | | 0 | |
| Fibroblasts | Fibroblasts | PROS1 | 0.48 | | 0 | |
| Fibroblasts | Fibroblasts | RERG | 0.48 | | 0 | |
| Fibroblasts | Fibroblasts | RUNX1T1 | 0.48 | | 0 | |
| Fibroblasts | Fibroblasts | SULF1 | 0.47 | | 0 | |
| Fibroblasts | Fibroblasts | MFAP2 | 0.47 | | 0 | |
| Fibroblasts | Fibroblasts | 4-Sep | 0.47 | | 0 | |
| Fibroblasts | Fibroblasts | LAMB1 | 0.46 | | 0 | |
| Fibroblasts | Fibroblasts | SVEP1 | 0.46 | | 0 | |
| Fibroblasts | Fibroblasts | TPBG | 0.46 | | 0 | |
| Fibroblasts | Fibroblasts | RHOBTB3 | 0.46 | | 0 | |
| Fibroblasts | Fibroblasts | COX4I2 | 0.46 | | 0 | |
| Fibroblasts | Fibroblasts | PLEKHA4 | 0.45 | | 0 | |
| Fibroblasts | Fibroblasts | EFHD1 | 0.45 | | 0 | |
| Fibroblasts | Fibroblasts | PLOD2 | 0.45 | | 0 | |
| Fibroblasts | Fibroblasts | ZNF503 | 0.45 | | 0 | |
| Fibroblasts | Fibroblasts | TNC | 0.44 | | 0 | |
| Fibroblasts | Fibroblasts | DLC1 | 0.44 | | 0 | |
| Fibroblasts | Fibroblasts | ST3GAL4 | 0.44 | | 0 | |
| Fibroblasts | Fibroblasts | LMOD1 | 0.44 | | 0 | |
| Fibroblasts | Fibroblasts | IL34 | 0.44 | | 0 | |
| Fibroblasts | Fibroblasts | SYNPO2 | 0.44 | | 0 | |
| Fibroblasts | Fibroblasts | PLSCR4 | 0.44 | | 0 | |
| Fibroblasts | Fibroblasts | RCAN2 | 0.44 | | 0 | |
| Fibroblasts | Fibroblasts | SPOCK1 | 0.43 | | 0 | |
| Fibroblasts | Fibroblasts | COL16A1 | 0.43 | | 0 | |
| Fibroblasts | Fibroblasts | C2orf40 | 0.43 | | 0 | |
| Fibroblasts | Fibroblasts | ABCA6 | 0.43 | | 0 | |
| Fibroblasts | Fibroblasts | F10 | 0.42 | | 0 | |
| Fibroblasts | Fibroblasts | LTBP2 | 0.42 | | 0 | |
| Fibroblasts | Fibroblasts | PARVA | 0.42 | | 0 | |
| Fibroblasts | Fibroblasts | CH25H | 0.42 | | 0 | |
| Fibroblasts | Fibroblasts | TNS1 | 0.41 | | 0 | |
| Fibroblasts | Fibroblasts | GUCY1B1 | 0.41 | | 0 | |
| Fibroblasts | Fibroblasts | FIBIN | 0.41 | | 0 | |
| Fibroblasts | Fibroblasts | ITIH5 | 0.4 | | 0 | |
| Fibroblasts | Fibroblasts | MAP1A | 0.4 | | 0 | |
| Fibroblasts | Fibroblasts | SCN7A | 0.4 | | 0 | |
| Fibroblasts | Fibroblasts | ADGRA2 | 0.4 | | 0 | |
| Fibroblasts | Fibroblasts | CCDC3 | 0.4 | | 0 | |
| Fibroblasts | Fibroblasts | CCDC102B | 0.4 | | 0 | |
| Fibroblasts | Fibroblasts | SGIP1 | 0.4 | | 0 | |
| Fibroblasts | Fibroblasts | HEYL | 0.4 | | 0 | |
| Fibroblasts | Fibroblasts | SLIT2 | 0.4 | | 0 | |
| Fibroblasts | Fibroblasts | PTCH1 | 0.39 | | 0 | |
| Fibroblasts | Fibroblasts | MRGPRF | 0.39 | | 0 | |
| Fibroblasts | Fibroblasts | GPC6 | 0.39 | | 0 | |
| Fibroblasts | Fibroblasts | HSPB8 | 0.39 | | 0 | |
| Fibroblasts | Fibroblasts | HSD11B1 | 0.39 | | 0 | |
| Fibroblasts | Fibroblasts | EDIL3 | 0.39 | | 0 | |
| Fibroblasts | Fibroblasts | PDE1A | 0.39 | | 0 | |
| Fibroblasts | Fibroblasts | TNS2 | 0.38 | | 0 | |
| Fibroblasts | Fibroblasts | FLRT2 | 0.38 | | 0 | |
| Fibroblasts | Fibroblasts | TPST1 | 0.38 | | 0 | |
| Fibroblasts | Fibroblasts | INMT | 0.38 | | 0 | |
| Fibroblasts | Fibroblasts | FAM20C | 0.38 | | 0 | |
| Fibroblasts | Fibroblasts | PTGES | 0.38 | | 0 | |
| Fibroblasts | Fibroblasts | NOVA1 | 0.38 | | 0 | |
| Fibroblasts | Fibroblasts | SNHG18 | 0.37 | | 0 | |
| Fibroblasts | Fibroblasts | AOC3 | 0.37 | | 0 | |
| Fibroblasts | Fibroblasts | MSRB3 | 0.37 | | 0 | |
| Fibroblasts | Fibroblasts | TMEM100 | 0.37 | | 0 | |
| Fibroblasts | Fibroblasts | ITGA7 | 0.37 | | 0 | |
| Fibroblasts | Fibroblasts | NTN4 | 0.37 | | 0 | |
| Fibroblasts | Fibroblasts | HHIP | 0.37 | | 0 | |
| Fibroblasts | Fibroblasts | SNAI2 | 0.36 | | 0 | |
| Fibroblasts | Fibroblasts | A4GALT | 0.36 | | 0 | |
| Fibroblasts | Fibroblasts | EPHA3 | 0.36 | | 0 | |
| Fibroblasts | Fibroblasts | COLEC12 | 0.36 | | 0 | |
| Fibroblasts | Fibroblasts | ADAM33 | 0.36 | | 0 | |
| Fibroblasts | Fibroblasts | HSPB2 | 0.36 | | 0 | |
| Fibroblasts | Fibroblasts | DPYSL3 | 0.36 | | 0 | |
| Fibroblasts | Fibroblasts | NFASC | 0.35 | | 0 | |
| Fibroblasts | Fibroblasts | FEZ1 | 0.35 | | 0 | |
| Fibroblasts | Fibroblasts | PAPPA | 0.35 | | 0 | |
| Fibroblasts | Fibroblasts | LARP6 | 0.35 | | 0 | |
| Fibroblasts | Fibroblasts | WNT2B | 0.35 | | 0 | |
| Fibroblasts | Fibroblasts | LOX | 0.35 | | 0 | |
| Fibroblasts | Fibroblasts | DACT1 | 0.35 | | 0 | |
| Fibroblasts | Fibroblasts | PLEKHH2 | 0.35 | | 0 | |
| Fibroblasts | Fibroblasts | C16orf45 | 0.35 | | 0 | |
| Fibroblasts | Fibroblasts | FAM162B | 0.34 | | 0 | |
| Fibroblasts | Fibroblasts | FENDRR | 0.34 | | 0 | |
| Fibroblasts | Fibroblasts | PDE5A | 0.34 | | 0 | |
| Fibroblasts | Fibroblasts | EDNRA | 0.34 | | 0 | |
| Fibroblasts | Fibroblasts | C1QTNF2 | 0.34 | | 0 | |
| Fibroblasts | Fibroblasts | CNRIP1 | 0.34 | | 0 | |
| Fibroblasts | Fibroblasts | KCNE4 | 0.34 | | 0 | |
| Fibroblasts | Fibroblasts | LAMA2 | 0.34 | | 0 | |
| Fibroblasts | Fibroblasts | TUSC3 | 0.34 | | 0 | |
| Fibroblasts | Fibroblasts | BNC2 | 0.34 | | 0 | |
| Fibroblasts | Fibroblasts | BOC | 0.33 | | 0 | |
| Fibroblasts | Fibroblasts | CPXM1 | 0.33 | | 0 | |
| Fibroblasts | Fibroblasts | FRMD6 | 0.33 | | 0 | |
| Fibroblasts | Fibroblasts | MEOX2 | 0.33 | | 0 | |
| Fibroblasts | Fibroblasts | CHRDL1 | 0.33 | | 0 | |
| Fibroblasts | Fibroblasts | OLFML1 | 0.32 | | 0 | |
| Fibroblasts | Fibroblasts | PXDN | 0.32 | | 0 | |
| Fibroblasts | Fibroblasts | CILP | 0.32 | | 0 | |
| Fibroblasts | Fibroblasts | FAM92A | 0.32 | | 0 | |
| Fibroblasts | Fibroblasts | RGS4 | 0.32 | | 0 | |
| Fibroblasts | Fibroblasts | PRR16 | 0.32 | | 0 | |
| Fibroblasts | Fibroblasts | ADAMTSL3 | 0.32 | | 0 | |
| Fibroblasts | Fibroblasts | HAS2 | 0.32 | | 0 | |
| Fibroblasts | Fibroblasts | FMO2 | 0.32 | | 0 | |
| Fibroblasts | Fibroblasts | TGFB3 | 0.31 | | 0 | |
| Fibroblasts | Fibroblasts | C16orf89 | 0.31 | | 0 | |
| Fibroblasts | Fibroblasts | LRRN4CL | 0.31 | | 0 | |
| Fibroblasts | Fibroblasts | FAP | 0.31 | | 0 | |
| Fibroblasts | Fibroblasts | GREM1 | 0.3 | | 0 | |
| Fibroblasts | Fibroblasts | CDO1 | 0.3 | | 0 | |
| Fibroblasts | Fibroblasts | FBXO17 | 0.3 | | 0 | |
| Fibroblasts | Fibroblasts | VIPR2 | 0.3 | | 0 | |
| Fibroblasts | Fibroblasts | MIR99AHG | 0.3 | | 0 | |
| Fibroblasts | Fibroblasts | SEMA5A | 0.29 | | 0 | |
| Fibroblasts | Fibroblasts | ANKRD35 | 0.29 | | 0 | |
| Fibroblasts | Fibroblasts | VSTM4 | 0.29 | | 0 | |
| Fibroblasts | Fibroblasts | TBX2-AS1 | 0.29 | | 0 | |
| Fibroblasts | Fibroblasts | ANGPTL1 | 0.29 | | 0 | |
| Fibroblasts | Fibroblasts | GSTM5 | 0.29 | | 0 | |
| Fibroblasts | Fibroblasts | MRVI1 | 0.29 | | 0 | |
| Fibroblasts | Fibroblasts | ZCCHC24 | 0.29 | | 0 | |
| Fibroblasts | Fibroblasts | LSAMP | 0.29 | | 0 | |
| Fibroblasts | Fibroblasts | PCDH18 | 0.29 | | 0 | |
| Fibroblasts | Fibroblasts | ITGBL1 | 0.29 | | 0 | |
| Fibroblasts | Fibroblasts | GFRA1 | 0.29 | | 0 | |
| Fibroblasts | Fibroblasts | MEIS1 | 0.29 | | 0 | |
| Fibroblasts | Fibroblasts | MIR497HG | 0.29 | | 0 | |
| Fibroblasts | Fibroblasts | NEGR1 | 0.28 | | 0 | |
| Fibroblasts | Fibroblasts | ABCA9 | 0.28 | | 0 | |
| Fibroblasts | Fibroblasts | PLPPR4 | 0.28 | | 0 | |
| Fibroblasts | Fibroblasts | HHIP-AS1 | 0.28 | | 0 | |
| Fibroblasts | Fibroblasts | IGFN1 | 0.28 | | 0 | |
| Fibroblasts | Fibroblasts | PTGIS | 0.27 | | 0 | |
| Fibroblasts | Fibroblasts | SGCA | 0.27 | | 0 | |
| Fibroblasts | Fibroblasts | BARX1 | 0.27 | | 0 | |
| Fibroblasts | Fibroblasts | SMIM10 | 0.27 | | 0 | |
| Fibroblasts | Fibroblasts | SMARCA1 | 0.27 | | 0 | |
| Fibroblasts | Fibroblasts | SGCD | 0.27 | | 0 | |
| Fibroblasts | Fibroblasts | TBX2 | 0.27 | | 0 | |
| Fibroblasts | Fibroblasts | SHISA3 | 0.26 | | 0 | |
| Fibroblasts | Fibroblasts | NFATC4 | 0.26 | | 0 | |
| Fibroblasts | Fibroblasts | NR2F2-AS1 | 0.26 | | 0 | |
| Fibroblasts | Fibroblasts | LINC01082 | 0.26 | | 0 | |
| Fibroblasts | Fibroblasts | RGMA | 0.26 | | 0 | |
| Fibroblasts | Fibroblasts | EPDR1 | 0.25 | | 0 | |
| Fibroblasts | Fibroblasts | KCNJ8 | 0.25 | | 0 | |
| Fibroblasts | Fibroblasts | ADAMTS2 | 0.25 | | 0 | |
| Fibroblasts | Fibroblasts | MMP14 | 0.69 | | 1.88E-305 | |
| Fibroblasts | Fibroblasts | RBPMS | 0.58 | | 6.67E-303 | |
| Fibroblasts | Fibroblasts | EBF1 | 0.6 | | 1.18E-302 | |
| Fibroblasts | Fibroblasts | CNPY4 | 0.31 | | 1.44E-302 | |
| Fibroblasts | Fibroblasts | ARMCX1 | 0.32 | | 4.92E-301 | |
| Fibroblasts | Fibroblasts | BDH2 | 0.6 | | 6.64E-298 | |
| Fibroblasts | Fibroblasts | SCPEP1 | 0.94 | | 4.79E-297 | |
| Fibroblasts | Fibroblasts | CYP27A1 | 0.3 | | 3.89E-296 | |
| Fibroblasts | Fibroblasts | PKIG | 0.75 | | 1.19E-295 | |
| Fibroblasts | Fibroblasts | RARRES1 | 0.86 | | 1.86E-289 | |
| Fibroblasts | Fibroblasts | PDLIM7 | 0.77 | | 1.83E-286 | |
| Fibroblasts | Fibroblasts | TSC22D1 | 1.06 | | 1.85E-286 | |
| Fibroblasts | Fibroblasts | HOXA5 | 0.31 | | 1.56E-285 | |
| Fibroblasts | Fibroblasts | NTRK2 | 0.32 | | 7.55E-285 | |
| Fibroblasts | Fibroblasts | CD63 | 1.33 | | 9.46E-285 | |
| Fibroblasts | Fibroblasts | OAF | 0.57 | | 2.08E-283 | |
| Fibroblasts | Fibroblasts | LOXL2 | 0.34 | | 6.08E-282 | |
| Fibroblasts | Fibroblasts | ADIRF | 1.89 | | 4.89E-279 | |
| Fibroblasts | Fibroblasts | HES1 | 1.47 | | 1.99E-277 | |
| Fibroblasts | Fibroblasts | MYH11 | 1.06 | | 8.99E-277 | |
| Fibroblasts | Fibroblasts | PPP1R14A | 1 | | 1.02E-276 | |
| Fibroblasts | Fibroblasts | PGF | 0.41 | | 1.25E-275 | |
| Fibroblasts | Fibroblasts | HSPB1 | 1.42 | | 2.48E-275 | |
| Fibroblasts | Fibroblasts | CTSO | 0.44 | | 9.22E-275 | |
| Fibroblasts | Fibroblasts | MEG8 | 0.25 | | 2.44E-274 | |
| Fibroblasts | Fibroblasts | PLPP1 | 0.68 | | 1.84E-273 | |
| Fibroblasts | Fibroblasts | CAV2 | 0.57 | | 3.44E-273 | |
| Fibroblasts | Fibroblasts | LGALS1 | 1.38 | | 5.08E-273 | |
| Fibroblasts | Fibroblasts | WWTR1 | 0.39 | | 1.94E-270 | |
| Fibroblasts | Fibroblasts | DSTN | 1.45 | | 1.16E-269 | |
| Fibroblasts | Fibroblasts | CRTAP | 0.72 | | 2.54E-268 | |
| Fibroblasts | Fibroblasts | PLS3 | 0.48 | | 2.02E-265 | |
| Fibroblasts | Fibroblasts | NRP1 | 0.49 | | 1.67E-263 | |
| Fibroblasts | Fibroblasts | CAPN6 | 0.29 | | 2.10E-262 | |
| Fibroblasts | Fibroblasts | SORBS3 | 0.44 | | 2.34E-262 | |
| Fibroblasts | Fibroblasts | GSTA4 | 0.31 | | 9.53E-261 | |
| Fibroblasts | Fibroblasts | TMEM204 | 0.38 | | 4.95E-259 | |
| Fibroblasts | Fibroblasts | TPPP3 | 0.78 | | 4.83E-257 | |
| Fibroblasts | Fibroblasts | WLS | 0.48 | | 1.79E-256 | |
| Fibroblasts | Fibroblasts | EMP1 | 0.98 | | 6.75E-256 | |
| Fibroblasts | Fibroblasts | HSPA1A | 1.83 | | 1.81E-252 | |
| Fibroblasts | Fibroblasts | MEDAG | 0.29 | | 1.19E-251 | |
| Fibroblasts | Fibroblasts | NFIX | 0.41 | | 1.68E-251 | |
| Fibroblasts | Fibroblasts | PTGIR | 0.29 | | 9.35E-251 | |
| Fibroblasts | Fibroblasts | ALDH1A1 | 1 | | 4.59E-250 | |
| Fibroblasts | Fibroblasts | TSHZ2 | 0.6 | | 6.31E-250 | |
| Fibroblasts | Fibroblasts | HAS1 | 0.51 | | 4.34E-247 | |
| Fibroblasts | Fibroblasts | INAFM1 | 0.62 | | 1.49E-246 | |
| Fibroblasts | Fibroblasts | PLXDC2 | 0.4 | | 8.13E-246 | |
| Fibroblasts | Fibroblasts | ENG | 0.45 | | 2.09E-245 | |
| Fibroblasts | Fibroblasts | MMP11 | 0.44 | | 6.27E-244 | |
| Fibroblasts | Fibroblasts | SRGN | -2.6 | | 7.99E-244 | |
| Fibroblasts | Fibroblasts | IL33 | 0.36 | | 9.28E-243 | |
| Fibroblasts | Fibroblasts | PLAGL1 | 0.43 | | 1.34E-241 | |
| Fibroblasts | Fibroblasts | DST | 0.6 | | 4.66E-240 | |
| Fibroblasts | Fibroblasts | PAM | 0.51 | | 6.22E-240 | |
| Fibroblasts | Fibroblasts | TNFAIP6 | 0.51 | | 1.21E-239 | |
| Fibroblasts | Fibroblasts | GSTM3 | 0.62 | | 1.44E-238 | |
| Fibroblasts | Fibroblasts | MAP3K20 | 0.45 | | 3.00E-237 | |
| Fibroblasts | Fibroblasts | HNMT | 0.5 | | 8.54E-234 | |
| Fibroblasts | Fibroblasts | C1orf21 | 0.51 | | 2.23E-233 | |
| Fibroblasts | Fibroblasts | ST5 | 0.4 | | 6.09E-232 | |
| Fibroblasts | Fibroblasts | VCAM1 | 0.36 | | 5.29E-231 | |
| Fibroblasts | Fibroblasts | MXRA7 | 0.4 | | 1.25E-228 | |
| Fibroblasts | Fibroblasts | CCL21 | 1.04 | | 3.40E-228 | |
| Fibroblasts | Fibroblasts | DEPP1 | 1.17 | | 1.01E-226 | |
| Fibroblasts | Fibroblasts | SPTBN1 | 0.77 | | 1.04E-226 | |
| Fibroblasts | Fibroblasts | LAPTM4A | 1.16 | | 1.49E-225 | |
| Fibroblasts | Fibroblasts | RCN1 | 0.65 | | 4.82E-224 | |
| Fibroblasts | Fibroblasts | P2RY14 | 0.32 | | 1.48E-223 | |
| Fibroblasts | Fibroblasts | UACA | 0.42 | | 1.10E-221 | |
| Fibroblasts | Fibroblasts | ITGB1 | 0.93 | | 4.22E-221 | |
| Fibroblasts | Fibroblasts | PHLDA3 | 0.4 | | 5.87E-221 | |
| Fibroblasts | Fibroblasts | ACTN1 | 0.76 | | 6.73E-219 | |
| Fibroblasts | Fibroblasts | EID1 | 1.02 | | 1.55E-215 | |
| Fibroblasts | Fibroblasts | GAS6 | 0.87 | | 6.35E-212 | |
| Fibroblasts | Fibroblasts | JAM3 | 0.27 | | 4.61E-211 | |
| Fibroblasts | Fibroblasts | MTRNR2L2 | 1.13 | | 1.36E-209 | |
| Fibroblasts | Fibroblasts | FHL2 | 0.69 | | 4.98E-207 | |
| Fibroblasts | Fibroblasts | CTHRC1 | 0.84 | | 2.48E-206 | |
| Fibroblasts | Fibroblasts | HCFC1R1 | 0.82 | | 3.10E-206 | |
| Fibroblasts | Fibroblasts | OSMR | 0.3 | | 6.90E-206 | |
| Fibroblasts | Fibroblasts | TUBB6 | 0.49 | | 1.04E-204 | |
| Fibroblasts | Fibroblasts | CYB5R3 | 0.75 | | 2.16E-204 | |
| Fibroblasts | Fibroblasts | HSPA1B | 1.55 | | 7.85E-203 | |
| Fibroblasts | Fibroblasts | F3 | 0.82 | | 1.22E-202 | |
| Fibroblasts | Fibroblasts | TAX1BP3 | 0.73 | | 1.51E-202 | |
| Fibroblasts | Fibroblasts | CERCAM | 0.42 | | 1.84E-201 | |
| Fibroblasts | Fibroblasts | SOCS3 | 1.46 | | 2.81E-201 | |
| Fibroblasts | Fibroblasts | CD151 | 0.91 | | 3.58E-200 | |
| Fibroblasts | Fibroblasts | FKBP9 | 0.41 | | 2.13E-199 | |
| Fibroblasts | Fibroblasts | ACKR3 | 0.5 | | 4.19E-199 | |
| Fibroblasts | Fibroblasts | FAM198B | 0.27 | | 2.45E-198 | |
| Fibroblasts | Fibroblasts | INHBA | 0.5 | | 5.61E-198 | |
| Fibroblasts | Fibroblasts | RBFOX2 | 0.32 | | 6.04E-198 | |
| Fibroblasts | Fibroblasts | CRIP2 | 0.63 | | 1.99E-197 | |
| Fibroblasts | Fibroblasts | PDLIM2 | 0.62 | | 8.45E-197 | |
| Fibroblasts | Fibroblasts | SMTN | 0.44 | | 3.90E-196 | |
| Fibroblasts | Fibroblasts | 11-Sep | 0.54 | | 5.64E-196 | |
| Fibroblasts | Fibroblasts | DNAJB4 | 0.52 | | 8.94E-195 | |
| Fibroblasts | Fibroblasts | C1orf54 | 0.39 | | 1.85E-194 | |
| Fibroblasts | Fibroblasts | KLF9 | 0.58 | | 5.64E-191 | |
| Fibroblasts | Fibroblasts | C1RL | 0.28 | | 6.23E-189 | |
| Fibroblasts | Fibroblasts | IL6ST | 0.68 | | 1.72E-188 | |
| Fibroblasts | Fibroblasts | CTSL | 0.34 | | 5.84E-188 | |
| Fibroblasts | Fibroblasts | H1F0 | 0.57 | | 8.31E-188 | |
| Fibroblasts | Fibroblasts | LEPROT | 0.58 | | 9.62E-188 | |
| Fibroblasts | Fibroblasts | C9orf3 | 0.54 | | 8.50E-186 | |
| Fibroblasts | Fibroblasts | SOX4 | 0.72 | | 1.19E-185 | |
| Fibroblasts | Fibroblasts | PLAU | 0.82 | | 1.37E-185 | |
| Fibroblasts | Fibroblasts | EPS8 | 0.51 | | 1.91E-185 | |
| Fibroblasts | Fibroblasts | P4HA2 | 0.33 | | 4.87E-185 | |
| Fibroblasts | Fibroblasts | ERRFI1 | 0.68 | | 1.49E-184 | |
| Fibroblasts | Fibroblasts | CYBA | -1.4 | | 1.15E-183 | |
| Fibroblasts | Fibroblasts | RAB13 | 0.74 | | 2.82E-183 | |
| Fibroblasts | Fibroblasts | FAM114A1 | 0.43 | | 1.20E-181 | |
| Fibroblasts | Fibroblasts | C2 | 0.38 | | 2.53E-181 | |
| Fibroblasts | Fibroblasts | BAG3 | 0.66 | | 1.40E-178 | |
| Fibroblasts | Fibroblasts | ITGB5 | 0.34 | | 6.45E-177 | |
| Fibroblasts | Fibroblasts | KLF4 | 1.28 | | 9.68E-177 | |
| Fibroblasts | Fibroblasts | IL1R1 | 0.37 | | 1.85E-176 | |
| Fibroblasts | Fibroblasts | NREP | 0.3 | | 9.59E-176 | |
| Fibroblasts | Fibroblasts | F2R | 0.56 | | 5.38E-174 | |
| Fibroblasts | Fibroblasts | ID3 | 1.07 | | 7.50E-174 | |
| Fibroblasts | Fibroblasts | S100A6 | 0.72 | | 2.03E-173 | |
| Fibroblasts | Fibroblasts | EVA1B | 0.35 | | 3.32E-173 | |
| Fibroblasts | Fibroblasts | ISYNA1 | 0.48 | | 7.66E-173 | |
| Fibroblasts | Fibroblasts | EZR | -1.67 | | 1.42E-172 | |
| Fibroblasts | Fibroblasts | CD276 | 0.27 | | 2.53E-172 | |
| Fibroblasts | Fibroblasts | STMN3 | 0.28 | | 4.34E-172 | |
| Fibroblasts | Fibroblasts | TNFRSF1A | 0.53 | | 4.42E-172 | |
| Fibroblasts | Fibroblasts | ITM2B | 0.91 | | 5.01E-172 | |
| Fibroblasts | Fibroblasts | CD74 | -2.2 | | 5.43E-172 | |
| Fibroblasts | Fibroblasts | TCF4 | 0.56 | | 3.98E-171 | |
| Fibroblasts | Fibroblasts | FILIP1 | 0.3 | | 3.36E-170 | |
| Fibroblasts | Fibroblasts | TGFBI | 0.81 | | 3.56E-170 | |
| Fibroblasts | Fibroblasts | GNG12 | 0.45 | | 1.38E-169 | |
| Fibroblasts | Fibroblasts | PTPRS | 0.3 | | 1.71E-169 | |
| Fibroblasts | Fibroblasts | GULP1 | 0.33 | | 2.03E-169 | |
| Fibroblasts | Fibroblasts | C1QTNF1 | 0.33 | | 4.63E-169 | |
| Fibroblasts | Fibroblasts | CSF1 | 0.29 | | 9.48E-168 | |
| Fibroblasts | Fibroblasts | GPC1 | 0.31 | | 3.21E-167 | |
| Fibroblasts | Fibroblasts | CEBPB | 1.07 | | 1.22E-164 | |
| Fibroblasts | Fibroblasts | LTBP3 | 0.58 | | 1.69E-164 | |
| Fibroblasts | Fibroblasts | LMCD1 | 0.44 | | 5.73E-164 | |
| Fibroblasts | Fibroblasts | ANXA5 | 0.82 | | 2.37E-163 | |
| Fibroblasts | Fibroblasts | ATF3 | 1.15 | | 4.05E-163 | |
| Fibroblasts | Fibroblasts | CD81 | 0.73 | | 4.82E-163 | |
| Fibroblasts | Fibroblasts | YBX3 | 0.74 | | 2.13E-160 | |
| Fibroblasts | Fibroblasts | TCEAL8 | 0.56 | | 2.73E-160 | |
| Fibroblasts | Fibroblasts | TCIM | 0.78 | | 5.98E-160 | |
| Fibroblasts | Fibroblasts | SASH1 | 0.28 | | 8.13E-160 | |
| Fibroblasts | Fibroblasts | RPL28 | -0.69 | | 1.86E-159 | |
| Fibroblasts | Fibroblasts | GADD45B | 1.73 | | 9.60E-159 | |
| Fibroblasts | Fibroblasts | PRAF2 | 0.36 | | 1.59E-158 | |
| Fibroblasts | Fibroblasts | MTRNR2L8 | 0.84 | | 3.09E-158 | |
| Fibroblasts | Fibroblasts | ESD | 0.64 | | 6.97E-157 | |
| Fibroblasts | Fibroblasts | CXCR4 | -2.26 | | 8.39E-157 | |
| Fibroblasts | Fibroblasts | CD59 | 0.89 | | 1.55E-156 | |
| Fibroblasts | Fibroblasts | PTCH2 | 0.6 | | 1.42E-155 | |
| Fibroblasts | Fibroblasts | LAPTM5 | -1.83 | | 2.99E-154 | |
| Fibroblasts | Fibroblasts | MDK | 0.69 | | 1.17E-153 | |
| Fibroblasts | Fibroblasts | ARHGDIB | -1.52 | | 5.18E-153 | |
| Fibroblasts | Fibroblasts | DDAH2 | 0.66 | | 3.64E-151 | |
| Fibroblasts | Fibroblasts | ZNF703 | 0.36 | | 5.21E-151 | |
| Fibroblasts | Fibroblasts | APOL1 | 0.44 | | 7.57E-150 | |
| Fibroblasts | Fibroblasts | ARL2 | 0.56 | | 1.30E-149 | |
| Fibroblasts | Fibroblasts | RPS19 | -0.65 | | 1.97E-149 | |
| Fibroblasts | Fibroblasts | TRIP6 | 0.42 | | 4.67E-149 | |
| Fibroblasts | Fibroblasts | JAG1 | 0.51 | | 8.68E-149 | |
| Fibroblasts | Fibroblasts | RAI14 | 0.26 | | 3.42E-148 | |
| Fibroblasts | Fibroblasts | TCEAL1 | 0.35 | | 3.69E-147 | |
| Fibroblasts | Fibroblasts | QSOX1 | 0.71 | | 8.07E-146 | |
| Fibroblasts | Fibroblasts | PIP4P2 | 0.27 | | 3.98E-145 | |
| Fibroblasts | Fibroblasts | JUNB | 1.58 | | 7.05E-145 | |
| Fibroblasts | Fibroblasts | CD52 | -1.91 | | 1.67E-144 | |
| Fibroblasts | Fibroblasts | STOM | 0.49 | | 1.02E-143 | |
| Fibroblasts | Fibroblasts | KLF10 | 0.83 | | 1.62E-143 | |
| Fibroblasts | Fibroblasts | CTTN | 0.51 | | 3.11E-142 | |
| Fibroblasts | Fibroblasts | HLA-B | -0.87 | | 3.42E-142 | |
| Fibroblasts | Fibroblasts | MYO1B | 0.25 | | 9.76E-142 | |
| Fibroblasts | Fibroblasts | NUCKS1 | 0.76 | | 1.72E-140 | |
| Fibroblasts | Fibroblasts | CCL19 | 1.88 | | 3.12E-140 | |
| Fibroblasts | Fibroblasts | CALU | 0.63 | | 4.00E-140 | |
| Fibroblasts | Fibroblasts | ADM | 0.72 | | 6.73E-140 | |
| Fibroblasts | Fibroblasts | RIN2 | 0.3 | | 9.72E-140 | |
| Fibroblasts | Fibroblasts | NR4A1 | 1.14 | | 1.16E-139 | |
| Fibroblasts | Fibroblasts | RRAS | 0.41 | | 1.53E-139 | |
| Fibroblasts | Fibroblasts | RHOC | 0.71 | | 2.30E-139 | |
| Fibroblasts | Fibroblasts | CRABP2 | 0.4 | | 7.22E-139 | |
| Fibroblasts | Fibroblasts | RBMS1 | 0.47 | | 8.75E-139 | |
| Fibroblasts | Fibroblasts | PLD3 | 0.57 | | 2.24E-138 | |
| Fibroblasts | Fibroblasts | SELENBP1 | 0.44 | | 8.98E-138 | |
| Fibroblasts | Fibroblasts | SERPINE1 | 0.35 | | 1.16E-137 | |
| Fibroblasts | Fibroblasts | IER3 | 1.52 | | 1.16E-136 | |
| Fibroblasts | Fibroblasts | HEBP1 | 0.43 | | 3.79E-136 | |
| Fibroblasts | Fibroblasts | GRK5 | 0.36 | | 9.27E-136 | |
| Fibroblasts | Fibroblasts | NENF | 0.67 | | 2.09E-133 | |
| Fibroblasts | Fibroblasts | GINM1 | 0.42 | | 7.72E-133 | |
| Fibroblasts | Fibroblasts | RGS16 | 1.12 | | 2.26E-131 | |
| Fibroblasts | Fibroblasts | CREM | -1.64 | | 2.90E-131 | |
| Fibroblasts | Fibroblasts | CDKN1C | 0.3 | | 4.38E-131 | |
| Fibroblasts | Fibroblasts | HOXB-AS1 | 0.28 | | 2.10E-130 | |
| Fibroblasts | Fibroblasts | PLK2 | 0.55 | | 2.19E-130 | |
| Fibroblasts | Fibroblasts | ADH5 | 0.5 | | 3.10E-130 | |
| Fibroblasts | Fibroblasts | DTWD1 | 0.32 | | 4.32E-130 | |
| Fibroblasts | Fibroblasts | IL6 | 1.12 | | 1.64E-129 | |
| Fibroblasts | Fibroblasts | CD37 | -1.54 | | 1.86E-129 | |
| Fibroblasts | Fibroblasts | FCGRT | 0.62 | | 1.11E-128 | |
| Fibroblasts | Fibroblasts | NPDC1 | 0.31 | | 6.28E-128 | |
| Fibroblasts | Fibroblasts | SARAF | -1.32 | | 7.10E-127 | |
| Fibroblasts | Fibroblasts | GOLIM4 | 0.41 | | 9.91E-127 | |
| Fibroblasts | Fibroblasts | NPTN | 0.47 | | 1.40E-126 | |
| Fibroblasts | Fibroblasts | IGF1 | 0.58 | | 1.84E-125 | |
| Fibroblasts | Fibroblasts | SCARB2 | 0.51 | | 4.97E-125 | |
| Fibroblasts | Fibroblasts | RNF146 | 0.31 | | 6.07E-125 | |
| Fibroblasts | Fibroblasts | RPS29 | -0.85 | | 3.09E-124 | |
| Fibroblasts | Fibroblasts | ANGPT2 | 0.27 | | 3.94E-124 | |
| Fibroblasts | Fibroblasts | YPEL5 | -1.39 | | 1.34E-123 | |
| Fibroblasts | Fibroblasts | NME4 | 0.49 | | 4.01E-123 | |
| Fibroblasts | Fibroblasts | MYC | 1.17 | | 6.01E-123 | |
| Fibroblasts | Fibroblasts | LPP | 0.56 | | 1.56E-122 | |
| Fibroblasts | Fibroblasts | SLC29A1 | 0.35 | | 3.60E-122 | |
| Fibroblasts | Fibroblasts | HES4 | 0.59 | | 3.91E-121 | |
| Fibroblasts | Fibroblasts | VIM | 0.65 | | 7.07E-121 | |
| Fibroblasts | Fibroblasts | CORO1A | -1.41 | | 6.13E-120 | |
| Fibroblasts | Fibroblasts | TRIP10 | 0.33 | | 1.17E-119 | |
| Fibroblasts | Fibroblasts | TMEM9 | 0.35 | | 1.29E-119 | |
| Fibroblasts | Fibroblasts | SPART | 0.31 | | 1.45E-119 | |
| Fibroblasts | Fibroblasts | CD9 | 0.81 | | 1.49E-119 | |
| Fibroblasts | Fibroblasts | CRIM1 | 0.36 | | 1.85E-119 | |
| Fibroblasts | Fibroblasts | 10-Sep | 0.28 | | 3.64E-119 | |
| Fibroblasts | Fibroblasts | AMOTL2 | 0.29 | | 7.21E-119 | |
| Fibroblasts | Fibroblasts | SPATS2L | 0.48 | | 9.89E-119 | |
| Fibroblasts | Fibroblasts | PGRMC1 | 0.45 | | 1.62E-118 | |
| Fibroblasts | Fibroblasts | EGR3 | 0.45 | | 1.93E-118 | |
| Fibroblasts | Fibroblasts | RPLP2 | -0.67 | | 2.95E-118 | |
| Fibroblasts | Fibroblasts | FOS | 1.53 | | 4.73E-118 | |
| Fibroblasts | Fibroblasts | LIMA1 | 0.47 | | 5.79E-118 | |
| Fibroblasts | Fibroblasts | DPYSL2 | 0.37 | | 1.58E-117 | |
| Fibroblasts | Fibroblasts | RPS3 | -0.61 | | 2.12E-117 | |
| Fibroblasts | Fibroblasts | CSRP1 | 0.89 | | 2.31E-117 | |
| Fibroblasts | Fibroblasts | PLOD1 | 0.34 | | 4.77E-117 | |
| Fibroblasts | Fibroblasts | ILK | 0.51 | | 5.03E-117 | |
| Fibroblasts | Fibroblasts | VCL | 0.39 | | 9.15E-117 | |
| Fibroblasts | Fibroblasts | ST6GALNAC6 | 0.3 | | 5.98E-116 | |
| Fibroblasts | Fibroblasts | TSC22D3 | -1.4 | | 9.93E-115 | |
| Fibroblasts | Fibroblasts | TENT5A | 0.57 | | 1.39E-114 | |
| Fibroblasts | Fibroblasts | VKORC1 | 0.62 | | 7.12E-114 | |
| Fibroblasts | Fibroblasts | IFITM2 | 0.72 | | 8.84E-114 | |
| Fibroblasts | Fibroblasts | RGS1 | -1.93 | | 1.47E-113 | |
| Fibroblasts | Fibroblasts | PTPRCAP | -1.37 | | 2.06E-113 | |
| Fibroblasts | Fibroblasts | KCNQ1OT1 | 0.6 | | 2.62E-113 | |
| Fibroblasts | Fibroblasts | LAPTM4B | 0.4 | | 2.85E-113 | |
| Fibroblasts | Fibroblasts | CLIC4 | 0.44 | | 7.13E-113 | |
| Fibroblasts | Fibroblasts | MCAM | 0.59 | | 9.26E-113 | |
| Fibroblasts | Fibroblasts | ADD3 | 0.5 | | 1.38E-112 | |
| Fibroblasts | Fibroblasts | CLDN1 | 0.27 | | 1.58E-112 | |
| Fibroblasts | Fibroblasts | SELENOW | 0.63 | | 2.40E-112 | |
| Fibroblasts | Fibroblasts | SORBS2 | 0.54 | | 1.15E-111 | |
| Fibroblasts | Fibroblasts | CYTIP | -1.35 | | 2.19E-111 | |
| Fibroblasts | Fibroblasts | KLF6 | -1.09 | | 3.56E-111 | |
| Fibroblasts | Fibroblasts | PLEKHA5 | 0.31 | | 4.40E-111 | |
| Fibroblasts | Fibroblasts | FOSB | 1.5 | | 4.88E-111 | |
| Fibroblasts | Fibroblasts | ZFHX3 | 0.3 | | 1.31E-110 | |
| Fibroblasts | Fibroblasts | RPL39 | -0.6 | | 1.39E-110 | |
| Fibroblasts | Fibroblasts | ARC | 0.32 | | 1.89E-110 | |
| Fibroblasts | Fibroblasts | GCSH | 0.36 | | 2.25E-110 | |
| Fibroblasts | Fibroblasts | SPSB1 | 0.43 | | 2.37E-110 | |
| Fibroblasts | Fibroblasts | BTG1 | -1.18 | | 1.51E-109 | |
| Fibroblasts | Fibroblasts | SERPINB6 | 0.5 | | 1.71E-109 | |
| Fibroblasts | Fibroblasts | PSMB5 | 0.52 | | 1.49E-108 | |
| Fibroblasts | Fibroblasts | MAFF | 0.77 | | 4.68E-108 | |
| Fibroblasts | Fibroblasts | EGR2 | 0.7 | | 1.09E-107 | |
| Fibroblasts | Fibroblasts | VAT1 | 0.32 | | 1.65E-107 | |
| Fibroblasts | Fibroblasts | SLC39A1 | 0.42 | | 3.33E-107 | |
| Fibroblasts | Fibroblasts | ISG20 | -1.29 | | 3.65E-107 | |
| Fibroblasts | Fibroblasts | C12orf57 | 0.63 | | 5.51E-107 | |
| Fibroblasts | Fibroblasts | RPS2 | -0.53 | | 2.53E-106 | |
| Fibroblasts | Fibroblasts | TMEM14C | 0.58 | | 3.29E-106 | |
| Fibroblasts | Fibroblasts | CD69 | -1.8 | | 8.57E-105 | |
| Fibroblasts | Fibroblasts | METTL7A | 0.48 | | 1.36E-104 | |
| Fibroblasts | Fibroblasts | RTL8A | 0.39 | | 3.01E-103 | |
| Fibroblasts | Fibroblasts | REXO2 | 0.53 | | 8.95E-103 | |
| Fibroblasts | Fibroblasts | RPS14 | -0.48 | | 1.44E-102 | |
| Fibroblasts | Fibroblasts | IL2RG | -1.11 | | 1.78E-102 | |
| Fibroblasts | Fibroblasts | CD53 | -1.16 | | 8.17E-102 | |
| Fibroblasts | Fibroblasts | GPR183 | -1.57 | | 1.11E-101 | |
| Fibroblasts | Fibroblasts | RPLP1 | -0.48 | | 1.15E-101 | |
| Fibroblasts | Fibroblasts | PDGFD | 0.28 | | 2.39E-101 | |
| Fibroblasts | Fibroblasts | NUCB1 | 0.54 | | 8.07E-101 | |
| Fibroblasts | Fibroblasts | TMEM45A | 0.26 | | 1.29E-100 | |
| Fibroblasts | Fibroblasts | PHLDA1 | 0.94 | | 1.31E-100 | |
| Fibroblasts | Fibroblasts | COMT | 0.55 | | 1.38E-100 | |
| Fibroblasts | Fibroblasts | VAMP5 | 0.49 | | 1.63E-100 | |
| Fibroblasts | Fibroblasts | CBR1 | 0.41 | | 1.83E-100 | |
| Fibroblasts | Fibroblasts | UROD | 0.41 | | 2.71E-100 | |
| Fibroblasts | Fibroblasts | MT2A | 1.42 | | 3.10E-100 | |
| Fibroblasts | Fibroblasts | ZFP36L1 | 0.91 | | 7.90E-100 | |
| Fibroblasts | Fibroblasts | PPP1R12B | 0.32 | | 4.72E-99 | |
| Fibroblasts | Fibroblasts | PTPRC | -1.27 | | 9.75E-99 | |
| Fibroblasts | Fibroblasts | RPS15A | -0.53 | | 2.15E-98 | |
| Fibroblasts | Fibroblasts | FXYD5 | -1.05 | | 4.27E-98 | |
| Fibroblasts | Fibroblasts | UGDH | 0.42 | | 5.53E-98 | |
| Fibroblasts | Fibroblasts | MAP1LC3A | 0.47 | | 6.91E-98 | |
| Fibroblasts | Fibroblasts | TCEAL3 | 0.31 | | 9.64E-98 | |
| Fibroblasts | Fibroblasts | LAMP1 | 0.52 | | 1.68E-97 | |
| Fibroblasts | Fibroblasts | SPTSSA | 0.49 | | 8.13E-97 | |
| Fibroblasts | Fibroblasts | HSPA2 | 0.35 | | 2.18E-96 | |
| Fibroblasts | Fibroblasts | SYNE1 | 0.3 | | 5.00E-96 | |
| Fibroblasts | Fibroblasts | TCEA2 | 0.26 | | 8.82E-96 | |
| Fibroblasts | Fibroblasts | CTSC | 1.02 | | 7.66E-95 | |
| Fibroblasts | Fibroblasts | TUBB | 0.62 | | 3.68E-94 | |
| Fibroblasts | Fibroblasts | CREG1 | 0.41 | | 7.56E-94 | |
| Fibroblasts | Fibroblasts | RPS18 | -0.48 | | 2.34E-93 | |
| Fibroblasts | Fibroblasts | RRAD | 0.44 | | 3.13E-93 | |
| Fibroblasts | Fibroblasts | NFIL3 | 0.31 | | 7.65E-93 | |
| Fibroblasts | Fibroblasts | 8-Sep | 0.27 | | 2.07E-92 | |
| Fibroblasts | Fibroblasts | PTGS2 | 0.56 | | 1.21E-91 | |
| Fibroblasts | Fibroblasts | ZFYVE21 | 0.32 | | 1.42E-91 | |
| Fibroblasts | Fibroblasts | RNF24 | 0.31 | | 1.74E-91 | |
| Fibroblasts | Fibroblasts | LMO4 | 0.46 | | 2.67E-91 | |
| Fibroblasts | Fibroblasts | CDC42EP1 | 0.33 | | 6.71E-91 | |
| Fibroblasts | Fibroblasts | TCF7L2 | 0.34 | | 7.50E-91 | |
| Fibroblasts | Fibroblasts | IFI27 | 0.47 | | 7.73E-91 | |
| Fibroblasts | Fibroblasts | RTN4 | 0.61 | | 1.06E-90 | |
| Fibroblasts | Fibroblasts | PHACTR2 | 0.35 | | 2.37E-90 | |
| Fibroblasts | Fibroblasts | CD48 | -1.1 | | 4.55E-90 | |
| Fibroblasts | Fibroblasts | KDELR3 | 0.26 | | 3.47E-89 | |
| Fibroblasts | Fibroblasts | MTRNR2L12 | 0.59 | | 7.52E-89 | |
| Fibroblasts | Fibroblasts | RPS28 | -0.46 | | 9.74E-89 | |
| Fibroblasts | Fibroblasts | ENAH | 0.26 | | 1.17E-88 | |
| Fibroblasts | Fibroblasts | TXNIP | 0.69 | | 3.02E-88 | |
| Fibroblasts | Fibroblasts | PTK2 | 0.36 | | 3.49E-88 | |
| Fibroblasts | Fibroblasts | SAMSN1 | -1.17 | | 3.73E-88 | |
| Fibroblasts | Fibroblasts | PSAP | 0.5 | | 4.93E-88 | |
| Fibroblasts | Fibroblasts | LTBR | 0.31 | | 8.76E-88 | |
| Fibroblasts | Fibroblasts | RPL27A | -0.47 | | 1.07E-87 | |
| Fibroblasts | Fibroblasts | FBLIM1 | 0.31 | | 1.09E-87 | |
| Fibroblasts | Fibroblasts | LURAP1L | 0.26 | | 1.16E-87 | |
| Fibroblasts | Fibroblasts | MGST3 | 0.61 | | 1.25E-87 | |
| Fibroblasts | Fibroblasts | ARHGAP6 | 0.25 | | 1.33E-87 | |
| Fibroblasts | Fibroblasts | CHMP5 | 0.41 | | 3.06E-87 | |
| Fibroblasts | Fibroblasts | CLU | 1.28 | | 1.52E-86 | |
| Fibroblasts | Fibroblasts | MPZL1 | 0.34 | | 1.56E-86 | |
| Fibroblasts | Fibroblasts | UBE2E2 | 0.26 | | 2.28E-86 | |
| Fibroblasts | Fibroblasts | HEXB | 0.46 | | 4.42E-86 | |
| Fibroblasts | Fibroblasts | MYL6B | 0.37 | | 1.83E-85 | |
| Fibroblasts | Fibroblasts | DYNC1I2 | 0.45 | | 5.46E-85 | |
| Fibroblasts | Fibroblasts | MLF1 | 0.27 | | 7.38E-85 | |
| Fibroblasts | Fibroblasts | MAGEF1 | 0.28 | | 1.17E-84 | |
| Fibroblasts | Fibroblasts | OSBPL1A | 0.28 | | 6.99E-84 | |
| Fibroblasts | Fibroblasts | ZNF106 | 0.32 | | 7.40E-84 | |
| Fibroblasts | Fibroblasts | CKAP4 | 0.31 | | 2.97E-83 | |
| Fibroblasts | Fibroblasts | PDK4 | 0.89 | | 8.36E-83 | |
| Fibroblasts | Fibroblasts | HLA-DRB1 | -1.8 | | 1.05E-82 | |
| Fibroblasts | Fibroblasts | ABL2 | 0.47 | | 1.23E-82 | |
| Fibroblasts | Fibroblasts | PHPT1 | 0.56 | | 1.25E-82 | |
| Fibroblasts | Fibroblasts | NME3 | 0.44 | | 5.26E-82 | |
| Fibroblasts | Fibroblasts | PABPC1 | -0.72 | | 1.41E-81 | |
| Fibroblasts | Fibroblasts | PPIB | 0.61 | | 1.57E-81 | |
| Fibroblasts | Fibroblasts | TNFRSF12A | 0.65 | | 1.75E-81 | |
| Fibroblasts | Fibroblasts | RAB32 | 0.27 | | 1.24E-80 | |
| Fibroblasts | Fibroblasts | PLBD1 | 0.3 | | 1.41E-80 | |
| Fibroblasts | Fibroblasts | ATRAID | 0.49 | | 4.46E-80 | |
| Fibroblasts | Fibroblasts | GLT8D1 | 0.26 | | 1.02E-79 | |
| Fibroblasts | Fibroblasts | RASD1 | 0.43 | | 6.78E-79 | |
| Fibroblasts | Fibroblasts | MAGED2 | 0.43 | | 8.13E-79 | |
| Fibroblasts | Fibroblasts | ITGA1 | 0.28 | | 5.03E-78 | |
| Fibroblasts | Fibroblasts | CXCL2 | 0.97 | | 5.52E-78 | |
| Fibroblasts | Fibroblasts | RPS12 | -0.51 | | 2.17E-77 | |
| Fibroblasts | Fibroblasts | RPL30 | -0.5 | | 2.79E-77 | |
| Fibroblasts | Fibroblasts | CCDC71L | 0.3 | | 6.49E-77 | |
| Fibroblasts | Fibroblasts | ABI2 | 0.25 | | 1.52E-76 | |
| Fibroblasts | Fibroblasts | NCKAP1 | 0.3 | | 1.55E-76 | |
| Fibroblasts | Fibroblasts | DNAJB1 | 1.24 | | 2.35E-76 | |
| Fibroblasts | Fibroblasts | IL15RA | 0.26 | | 4.11E-76 | |
| Fibroblasts | Fibroblasts | FLNA | 0.68 | | 6.84E-76 | |
| Fibroblasts | Fibroblasts | NDUFS4 | 0.4 | | 8.76E-76 | |
| Fibroblasts | Fibroblasts | SPRY1 | 0.29 | | 1.32E-75 | |
| Fibroblasts | Fibroblasts | LY6E | 0.6 | | 1.88E-75 | |
| Fibroblasts | Fibroblasts | CFL2 | 0.27 | | 2.03E-75 | |
| Fibroblasts | Fibroblasts | IER2 | 1.13 | | 3.66E-75 | |
| Fibroblasts | Fibroblasts | OAZ1 | -0.59 | | 3.73E-75 | |
| Fibroblasts | Fibroblasts | RNF130 | 0.26 | | 6.83E-75 | |
| Fibroblasts | Fibroblasts | RSU1 | 0.38 | | 7.46E-75 | |
| Fibroblasts | Fibroblasts | GBP1 | 0.39 | | 7.81E-75 | |
| Fibroblasts | Fibroblasts | HLA-DPA1 | -1.72 | | 1.04E-74 | |
| Fibroblasts | Fibroblasts | H2AFJ | 0.5 | | 1.70E-74 | |
| Fibroblasts | Fibroblasts | RPS16 | -0.42 | | 2.52E-74 | |
| Fibroblasts | Fibroblasts | HCST | -1.22 | | 2.66E-74 | |
| Fibroblasts | Fibroblasts | EMC2 | 0.29 | | 3.35E-74 | |
| Fibroblasts | Fibroblasts | TMEM173 | 0.29 | | 6.24E-74 | |
| Fibroblasts | Fibroblasts | RDX | 0.32 | | 7.39E-74 | |
| Fibroblasts | Fibroblasts | HLA-DPB1 | -1.72 | | 1.49E-73 | |
| Fibroblasts | Fibroblasts | ZNF428 | 0.38 | | 1.62E-73 | |
| Fibroblasts | Fibroblasts | MAGED1 | 0.31 | | 2.25E-73 | |
| Fibroblasts | Fibroblasts | CLN5 | 0.27 | | 2.53E-73 | |
| Fibroblasts | Fibroblasts | SERTAD1 | 0.82 | | 4.75E-73 | |
| Fibroblasts | Fibroblasts | RGCC | -1.31 | | 5.90E-73 | |
| Fibroblasts | Fibroblasts | JUN | 0.98 | | 9.10E-73 | |
| Fibroblasts | Fibroblasts | CAMK2N1 | 0.29 | | 1.83E-72 | |
| Fibroblasts | Fibroblasts | RPS6 | -0.46 | | 2.10E-72 | |
| Fibroblasts | Fibroblasts | NECTIN2 | 0.34 | | 3.24E-72 | |
| Fibroblasts | Fibroblasts | RPL18A | -0.44 | | 1.21E-71 | |
| Fibroblasts | Fibroblasts | C1orf123 | 0.34 | | 1.57E-71 | |
| Fibroblasts | Fibroblasts | CBX6 | 0.33 | | 1.78E-71 | |
| Fibroblasts | Fibroblasts | MAP4 | 0.31 | | 2.34E-71 | |
| Fibroblasts | Fibroblasts | HSBP1 | 0.45 | | 2.71E-71 | |
| Fibroblasts | Fibroblasts | AK1 | 0.32 | | 2.83E-71 | |
| Fibroblasts | Fibroblasts | MOCS2 | 0.28 | | 4.72E-71 | |
| Fibroblasts | Fibroblasts | AHCYL1 | 0.36 | | 7.00E-71 | |
| Fibroblasts | Fibroblasts | RPS27 | -0.58 | | 7.39E-71 | |
| Fibroblasts | Fibroblasts | LGALS3 | 0.41 | | 7.40E-71 | |
| Fibroblasts | Fibroblasts | DAAM1 | 0.39 | | 8.51E-71 | |
| Fibroblasts | Fibroblasts | HLA-DQB1 | -1.62 | | 1.86E-70 | |
| Fibroblasts | Fibroblasts | RHOB | 0.72 | | 2.07E-70 | |
| Fibroblasts | Fibroblasts | FAU | -0.38 | | 2.34E-70 | |
| Fibroblasts | Fibroblasts | RPL19 | -0.43 | | 2.41E-70 | |
| Fibroblasts | Fibroblasts | CD99 | 0.57 | | 2.60E-70 | |
| Fibroblasts | Fibroblasts | RPS15 | -0.36 | | 5.78E-70 | |
| Fibroblasts | Fibroblasts | EEF1B2 | -0.65 | | 6.80E-70 | |
| Fibroblasts | Fibroblasts | CISD1 | 0.36 | | 6.98E-70 | |
| Fibroblasts | Fibroblasts | ECHDC2 | 0.35 | | 1.21E-69 | |
| Fibroblasts | Fibroblasts | PEBP1 | 0.51 | | 2.01E-69 | |
| Fibroblasts | Fibroblasts | ZBTB20 | 0.34 | | 2.35E-69 | |
| Fibroblasts | Fibroblasts | CHD9 | 0.37 | | 1.03E-68 | |
| Fibroblasts | Fibroblasts | ANKRD10 | 0.41 | | 1.25E-68 | |
| Fibroblasts | Fibroblasts | VAMP8 | -0.85 | | 2.33E-68 | |
| Fibroblasts | Fibroblasts | NFKBIZ | 0.54 | | 3.65E-68 | |
| Fibroblasts | Fibroblasts | HYI | 0.3 | | 7.53E-68 | |
| Fibroblasts | Fibroblasts | ID1 | 0.75 | | 8.98E-68 | |
| Fibroblasts | Fibroblasts | FAM3C | 0.46 | | 1.01E-67 | |
| Fibroblasts | Fibroblasts | ALDH2 | 0.38 | | 1.16E-67 | |
| Fibroblasts | Fibroblasts | MGST1 | 0.56 | | 1.51E-67 | |
| Fibroblasts | Fibroblasts | RPS27A | -0.41 | | 1.86E-67 | |
| Fibroblasts | Fibroblasts | SNX3 | 0.48 | | 3.26E-67 | |
| Fibroblasts | Fibroblasts | WSB1 | 0.55 | | 3.46E-67 | |
| Fibroblasts | Fibroblasts | IFI16 | 0.48 | | 3.91E-67 | |
| Fibroblasts | Fibroblasts | SELENOK | -0.81 | | 4.22E-67 | |
| Fibroblasts | Fibroblasts | RHOH | -0.99 | | 4.48E-67 | |
| Fibroblasts | Fibroblasts | EPAS1 | 0.37 | | 8.73E-67 | |
| Fibroblasts | Fibroblasts | RPSA | -0.58 | | 2.01E-66 | |
| Fibroblasts | Fibroblasts | CD3D | -1.56 | | 2.52E-66 | |
| Fibroblasts | Fibroblasts | RDH10 | 0.35 | | 8.13E-66 | |
| Fibroblasts | Fibroblasts | OSR2 | 0.25 | | 1.26E-65 | |
| Fibroblasts | Fibroblasts | HLA-C | -0.53 | | 1.47E-65 | |
| Fibroblasts | Fibroblasts | RAC2 | -0.9 | | 1.69E-65 | |
| Fibroblasts | Fibroblasts | ADD1 | 0.32 | | 2.79E-65 | |
| Fibroblasts | Fibroblasts | TNFAIP2 | 0.44 | | 3.59E-65 | |
| Fibroblasts | Fibroblasts | ARL3 | 0.27 | | 4.76E-65 | |
| Fibroblasts | Fibroblasts | PITX1 | 0.26 | | 6.06E-65 | |
| Fibroblasts | Fibroblasts | RUFY3 | 0.26 | | 1.12E-64 | |
| Fibroblasts | Fibroblasts | SH3BGRL3 | -0.76 | | 1.20E-64 | |
| Fibroblasts | Fibroblasts | MT1A | 0.97 | | 1.71E-64 | |
| Fibroblasts | Fibroblasts | TPM4 | 0.61 | | 2.13E-64 | |
| Fibroblasts | Fibroblasts | PTEN | 0.35 | | 2.60E-64 | |
| Fibroblasts | Fibroblasts | RPS7 | -0.41 | | 3.19E-64 | |
| Fibroblasts | Fibroblasts | AGTRAP | 0.27 | | 6.17E-64 | |
| Fibroblasts | Fibroblasts | MANBAL | 0.29 | | 7.26E-64 | |
| Fibroblasts | Fibroblasts | CDK4 | 0.32 | | 7.29E-64 | |
| Fibroblasts | Fibroblasts | OAZ2 | 0.4 | | 1.01E-63 | |
| Fibroblasts | Fibroblasts | ITGB1BP1 | 0.35 | | 1.07E-63 | |
| Fibroblasts | Fibroblasts | DCTN2 | 0.38 | | 1.65E-63 | |
| Fibroblasts | Fibroblasts | RPL23A | -0.41 | | 2.07E-63 | |
| Fibroblasts | Fibroblasts | IRF1 | 0.88 | | 2.09E-63 | |
| Fibroblasts | Fibroblasts | ARL1 | 0.37 | | 2.25E-63 | |
| Fibroblasts | Fibroblasts | GSTP1 | 0.37 | | 4.48E-63 | |
| Fibroblasts | Fibroblasts | PQLC3 | 0.29 | | 5.47E-63 | |
| Fibroblasts | Fibroblasts | STK4 | -0.86 | | 5.93E-63 | |
| Fibroblasts | Fibroblasts | EVI2B | -0.87 | | 7.05E-63 | |
| Fibroblasts | Fibroblasts | CD79A | -1.95 | | 7.76E-63 | |
| Fibroblasts | Fibroblasts | HLA-DMA | -1.01 | | 3.49E-62 | |
| Fibroblasts | Fibroblasts | BSG | 0.48 | | 5.46E-62 | |
| Fibroblasts | Fibroblasts | BANF1 | 0.4 | | 5.83E-62 | |
| Fibroblasts | Fibroblasts | CDC42SE2 | -0.9 | | 7.26E-62 | |
| Fibroblasts | Fibroblasts | SEM1 | 0.44 | | 8.06E-62 | |
| Fibroblasts | Fibroblasts | ATP6AP2 | 0.41 | | 1.15E-61 | |
| Fibroblasts | Fibroblasts | HLA-A | -0.59 | | 1.19E-61 | |
| Fibroblasts | Fibroblasts | ASPH | 0.27 | | 1.22E-61 | |
| Fibroblasts | Fibroblasts | 2-Sep | 0.41 | | 2.26E-61 | |
| Fibroblasts | Fibroblasts | CUEDC2 | 0.31 | | 2.35E-61 | |
| Fibroblasts | Fibroblasts | RPS21 | -0.6 | | 2.38E-61 | |
| Fibroblasts | Fibroblasts | RRBP1 | 0.4 | | 3.20E-61 | |
| Fibroblasts | Fibroblasts | ZFP36 | 1.12 | | 3.41E-61 | |
| Fibroblasts | Fibroblasts | FABP4 | 0.59 | | 4.02E-61 | |
| Fibroblasts | Fibroblasts | YAP1 | 0.26 | | 8.01E-61 | |
| Fibroblasts | Fibroblasts | RPL32 | -0.39 | | 1.31E-60 | |
| Fibroblasts | Fibroblasts | SOD2 | 0.47 | | 1.88E-60 | |
| Fibroblasts | Fibroblasts | RCAN1 | 0.38 | | 2.61E-60 | |
| Fibroblasts | Fibroblasts | LMNA | 0.51 | | 2.85E-60 | |
| Fibroblasts | Fibroblasts | RPL10 | -0.33 | | 4.10E-60 | |
| Fibroblasts | Fibroblasts | 7-Sep | 0.51 | | 4.45E-60 | |
| Fibroblasts | Fibroblasts | CTNNA1 | 0.32 | | 8.97E-60 | |
| Fibroblasts | Fibroblasts | GPSM3 | -0.81 | | 1.33E-59 | |
| Fibroblasts | Fibroblasts | ATP2A2 | 0.34 | | 4.40E-59 | |
| Fibroblasts | Fibroblasts | CYB5A | 0.36 | | 7.61E-59 | |
| Fibroblasts | Fibroblasts | CD7 | -1.53 | | 9.15E-59 | |
| Fibroblasts | Fibroblasts | CKB | 0.32 | | 1.02E-58 | |
| Fibroblasts | Fibroblasts | ABCA1 | 0.27 | | 1.38E-58 | |
| Fibroblasts | Fibroblasts | C5orf15 | 0.28 | | 2.49E-58 | |
| Fibroblasts | Fibroblasts | C6orf48 | 0.54 | | 3.30E-58 | |
| Fibroblasts | Fibroblasts | HLA-DRA | -2.39 | | 4.30E-58 | |
| Fibroblasts | Fibroblasts | GMFG | -0.78 | | 4.52E-58 | |
| Fibroblasts | Fibroblasts | RPL13 | -0.34 | | 5.46E-58 | |
| Fibroblasts | Fibroblasts | PDLIM1 | 0.57 | | 7.96E-58 | |
| Fibroblasts | Fibroblasts | CD3E | -1.14 | | 1.05E-57 | |
| Fibroblasts | Fibroblasts | CETN2 | 0.3 | | 1.45E-57 | |
| Fibroblasts | Fibroblasts | CHID1 | 0.36 | | 2.53E-57 | |
| Fibroblasts | Fibroblasts | TMEM109 | 0.29 | | 2.72E-57 | |
| Fibroblasts | Fibroblasts | TM2D1 | 0.33 | | 4.44E-57 | |
| Fibroblasts | Fibroblasts | RPL41 | -0.28 | | 6.60E-57 | |
| Fibroblasts | Fibroblasts | RPS20 | -0.39 | | 1.20E-56 | |
| Fibroblasts | Fibroblasts | DYNLL1 | 0.47 | | 1.37E-56 | |
| Fibroblasts | Fibroblasts | LTB | -1.26 | | 1.46E-56 | |
| Fibroblasts | Fibroblasts | KDELR1 | 0.37 | | 1.62E-56 | |
| Fibroblasts | Fibroblasts | ICAM1 | 0.53 | | 2.57E-56 | |
| Fibroblasts | Fibroblasts | ODF3B | 0.34 | | 5.64E-56 | |
| Fibroblasts | Fibroblasts | ZEB2 | 0.3 | | 6.41E-56 | |
| Fibroblasts | Fibroblasts | THYN1 | 0.27 | | 9.18E-56 | |
| Fibroblasts | Fibroblasts | TMEM14A | 0.3 | | 9.25E-56 | |
| Fibroblasts | Fibroblasts | TRIM47 | 0.26 | | 9.58E-56 | |
| Fibroblasts | Fibroblasts | APLP2 | 0.46 | | 9.99E-56 | |
| Fibroblasts | Fibroblasts | XIST | 0.45 | | 1.12E-55 | |
| Fibroblasts | Fibroblasts | ICAM3 | -0.78 | | 1.30E-55 | |
| Fibroblasts | Fibroblasts | IFI27L2 | 0.45 | | 2.70E-55 | |
| Fibroblasts | Fibroblasts | RPS27L | 0.49 | | 3.33E-55 | |
| Fibroblasts | Fibroblasts | ANGPTL4 | 0.34 | | 3.91E-55 | |
| Fibroblasts | Fibroblasts | FAM200B | 0.26 | | 5.73E-55 | |
| Fibroblasts | Fibroblasts | HLA-DQA1 | -1.46 | | 6.33E-55 | |
| Fibroblasts | Fibroblasts | LAMP2 | 0.36 | | 6.37E-55 | |
| Fibroblasts | Fibroblasts | IGLL5 | -2.59 | | 7.03E-55 | |
| Fibroblasts | Fibroblasts | MTCH1 | 0.39 | | 7.23E-55 | |
| Fibroblasts | Fibroblasts | STK17B | -0.84 | | 1.05E-54 | |
| Fibroblasts | Fibroblasts | MYO1C | 0.28 | | 1.21E-54 | |
| Fibroblasts | Fibroblasts | AK3 | 0.3 | | 4.53E-54 | |
| Fibroblasts | Fibroblasts | TUBA4A | -1.05 | | 6.59E-54 | |
| Fibroblasts | Fibroblasts | RPL36AL | -0.51 | | 6.72E-54 | |
| Fibroblasts | Fibroblasts | RUNX1 | 0.31 | | 9.00E-54 | |
| Fibroblasts | Fibroblasts | RPL18 | -0.35 | | 1.56E-53 | |
| Fibroblasts | Fibroblasts | MRFAP1 | 0.42 | | 1.78E-53 | |
| Fibroblasts | Fibroblasts | HDDC2 | 0.28 | | 2.57E-53 | |
| Fibroblasts | Fibroblasts | RPL21 | -0.37 | | 3.98E-53 | |
| Fibroblasts | Fibroblasts | CD2 | -1.17 | | 9.12E-53 | |
| Fibroblasts | Fibroblasts | RAC1 | 0.34 | | 1.14E-52 | |
| Fibroblasts | Fibroblasts | ADI1 | 0.38 | | 1.18E-52 | |
| Fibroblasts | Fibroblasts | GTF2H5 | 0.33 | | 1.50E-52 | |
| Fibroblasts | Fibroblasts | MT1M | 0.64 | | 2.33E-52 | |
| Fibroblasts | Fibroblasts | B2M | -0.39 | | 2.44E-52 | |
| Fibroblasts | Fibroblasts | RPL37 | -0.43 | | 7.34E-52 | |
| Fibroblasts | Fibroblasts | MUM1 | 0.29 | | 9.05E-52 | |
| Fibroblasts | Fibroblasts | RAMP1 | 0.5 | | 1.04E-51 | |
| Fibroblasts | Fibroblasts | CLTA | 0.37 | | 1.10E-51 | |
| Fibroblasts | Fibroblasts | UGCG | 0.3 | | 1.40E-51 | |
| Fibroblasts | Fibroblasts | LIMD2 | -0.85 | | 1.49E-51 | |
| Fibroblasts | Fibroblasts | NPC2 | 0.27 | | 2.17E-51 | |
| Fibroblasts | Fibroblasts | RPL8 | -0.35 | | 2.46E-51 | |
| Fibroblasts | Fibroblasts | RCN2 | 0.35 | | 2.60E-51 | |
| Fibroblasts | Fibroblasts | OS9 | 0.37 | | 6.34E-51 | |
| Fibroblasts | Fibroblasts | TAGAP | -0.81 | | 6.62E-51 | |
| Fibroblasts | Fibroblasts | HLA-E | -0.5 | | 7.04E-51 | |
| Fibroblasts | Fibroblasts | IFIT3 | 0.5 | | 9.96E-51 | |
| Fibroblasts | Fibroblasts | PEA15 | 0.29 | | 1.33E-50 | |
| Fibroblasts | Fibroblasts | RPL11 | -0.35 | | 1.56E-50 | |
| Fibroblasts | Fibroblasts | TUBA1A | 0.66 | | 3.03E-50 | |
| Fibroblasts | Fibroblasts | UCP2 | -0.8 | | 3.35E-50 | |
| Fibroblasts | Fibroblasts | GADD45G | 0.42 | | 1.47E-49 | |
| Fibroblasts | Fibroblasts | GRN | 0.28 | | 2.67E-49 | |
| Fibroblasts | Fibroblasts | RPL27 | -0.37 | | 3.00E-49 | |
| Fibroblasts | Fibroblasts | RPS11 | -0.37 | | 4.97E-49 | |
| Fibroblasts | Fibroblasts | AP2M1 | 0.38 | | 6.45E-49 | |
| Fibroblasts | Fibroblasts | SLC39A7 | 0.29 | | 7.41E-49 | |
| Fibroblasts | Fibroblasts | SPOCK2 | -0.96 | | 9.09E-49 | |
| Fibroblasts | Fibroblasts | JCHAIN | -3.65 | | 1.53E-48 | |
| Fibroblasts | Fibroblasts | RPS5 | -0.4 | | 1.71E-48 | |
| Fibroblasts | Fibroblasts | MRPL32 | 0.32 | | 1.92E-48 | |
| Fibroblasts | Fibroblasts | BCL6 | 0.28 | | 2.05E-48 | |
| Fibroblasts | Fibroblasts | PTTG1IP | 0.3 | | 2.70E-48 | |
| Fibroblasts | Fibroblasts | GABARAPL2 | 0.38 | | 2.91E-48 | |
| Fibroblasts | Fibroblasts | COTL1 | -0.9 | | 2.98E-48 | |
| Fibroblasts | Fibroblasts | RPL35 | -0.32 | | 3.28E-48 | |
| Fibroblasts | Fibroblasts | PLSCR1 | 0.33 | | 4.03E-48 | |
| Fibroblasts | Fibroblasts | ALOX5AP | -1.02 | | 5.55E-48 | |
| Fibroblasts | Fibroblasts | FLOT1 | 0.3 | | 6.84E-48 | |
| Fibroblasts | Fibroblasts | ADGRE5 | -0.81 | | 8.12E-48 | |
| Fibroblasts | Fibroblasts | RPS25 | -0.37 | | 8.78E-48 | |
| Fibroblasts | Fibroblasts | IAH1 | 0.29 | | 1.30E-47 | |
| Fibroblasts | Fibroblasts | CREB3L2 | 0.26 | | 2.86E-47 | |
| Fibroblasts | Fibroblasts | HLA-DRB5 | -1.28 | | 2.97E-47 | |
| Fibroblasts | Fibroblasts | TMEM126B | 0.26 | | 3.44E-47 | |
| Fibroblasts | Fibroblasts | MVP | 0.28 | | 7.63E-47 | |
| Fibroblasts | Fibroblasts | FUCA2 | 0.26 | | 1.53E-46 | |
| Fibroblasts | Fibroblasts | UBA52 | -0.33 | | 1.65E-46 | |
| Fibroblasts | Fibroblasts | BIRC3 | -0.82 | | 5.00E-46 | |
| Fibroblasts | Fibroblasts | BST2 | 0.62 | | 1.49E-45 | |
| Fibroblasts | Fibroblasts | HOTAIRM1 | 0.29 | | 1.56E-45 | |
| Fibroblasts | Fibroblasts | NDUFS2 | 0.33 | | 2.13E-45 | |
| Fibroblasts | Fibroblasts | KDELR2 | 0.38 | | 2.17E-45 | |
| Fibroblasts | Fibroblasts | RNASET2 | -0.7 | | 2.61E-45 | |
| Fibroblasts | Fibroblasts | CHURC1 | 0.28 | | 4.88E-45 | |
| Fibroblasts | Fibroblasts | NFE2L2 | 0.38 | | 5.67E-45 | |
| Fibroblasts | Fibroblasts | SEC63 | 0.26 | | 7.02E-45 | |
| Fibroblasts | Fibroblasts | OCIAD2 | -0.69 | | 1.35E-44 | |
| Fibroblasts | Fibroblasts | OAT | 0.32 | | 1.85E-44 | |
| Fibroblasts | Fibroblasts | EBPL | 0.29 | | 2.23E-44 | |
| Fibroblasts | Fibroblasts | IFI6 | 0.46 | | 2.58E-44 | |
| Fibroblasts | Fibroblasts | SMIM7 | 0.32 | | 2.77E-44 | |
| Fibroblasts | Fibroblasts | DUT | 0.3 | | 3.54E-44 | |
| Fibroblasts | Fibroblasts | SNCG | 0.51 | | 4.31E-44 | |
| Fibroblasts | Fibroblasts | CD44 | -0.74 | | 4.34E-44 | |
| Fibroblasts | Fibroblasts | RNASEH2C | 0.38 | | 4.94E-44 | |
| Fibroblasts | Fibroblasts | PRDX6 | 0.4 | | 7.10E-44 | |
| Fibroblasts | Fibroblasts | MIR22HG | 0.3 | | 1.20E-43 | |
| Fibroblasts | Fibroblasts | LEPROTL1 | -0.99 | | 1.27E-43 | |
| Fibroblasts | Fibroblasts | CXCL1 | 1.1 | | 1.92E-43 | |
| Fibroblasts | Fibroblasts | MT1E | 0.5 | | 2.04E-43 | |
| Fibroblasts | Fibroblasts | RRAGA | 0.27 | | 2.45E-43 | |
| Fibroblasts | Fibroblasts | BAD | 0.28 | | 4.01E-43 | |
| Fibroblasts | Fibroblasts | CST7 | -1.28 | | 5.35E-43 | |
| Fibroblasts | Fibroblasts | HCLS1 | -0.59 | | 5.45E-43 | |
| Fibroblasts | Fibroblasts | GRHPR | 0.26 | | 5.54E-43 | |
| Fibroblasts | Fibroblasts | RPS26 | -0.52 | | 5.87E-43 | |
| Fibroblasts | Fibroblasts | BTG2 | 0.64 | | 2.00E-42 | |
| Fibroblasts | Fibroblasts | PHF14 | 0.32 | | 2.12E-42 | |
| Fibroblasts | Fibroblasts | UBE2E3 | 0.28 | | 3.50E-42 | |
| Fibroblasts | Fibroblasts | COPS6 | 0.27 | | 4.25E-42 | |
| Fibroblasts | Fibroblasts | BCAM | 0.42 | | 7.52E-42 | |
| Fibroblasts | Fibroblasts | TNFSF13B | 0.35 | | 1.05E-41 | |
| Fibroblasts | Fibroblasts | MSRB2 | 0.26 | | 1.96E-41 | |
| Fibroblasts | Fibroblasts | DUSP2 | -1 | | 2.35E-41 | |
| Fibroblasts | Fibroblasts | MPRIP | 0.26 | | 2.71E-41 | |
| Fibroblasts | Fibroblasts | RNH1 | 0.33 | | 2.74E-41 | |
| Fibroblasts | Fibroblasts | ELF1 | -0.77 | | 6.61E-41 | |
| Fibroblasts | Fibroblasts | PNKD | 0.27 | | 1.11E-40 | |
| Fibroblasts | Fibroblasts | LCP1 | -0.7 | | 1.71E-40 | |
| Fibroblasts | Fibroblasts | MICALL2 | 0.26 | | 1.96E-40 | |
| Fibroblasts | Fibroblasts | SDHD | 0.27 | | 1.24E-39 | |
| Fibroblasts | Fibroblasts | NDUFB1 | 0.37 | | 1.39E-39 | |
| Fibroblasts | Fibroblasts | FAM49B | -0.62 | | 1.66E-39 | |
| Fibroblasts | Fibroblasts | NSRP1 | 0.32 | | 1.87E-39 | |
| Fibroblasts | Fibroblasts | ATP5PF | 0.33 | | 3.23E-39 | |
| Fibroblasts | Fibroblasts | BRK1 | 0.35 | | 3.66E-39 | |
| Fibroblasts | Fibroblasts | SERTAD3 | 0.26 | | 4.04E-39 | |
| Fibroblasts | Fibroblasts | DUSP4 | -0.83 | | 5.01E-39 | |
| Fibroblasts | Fibroblasts | YWHAE | 0.34 | | 5.04E-39 | |
| Fibroblasts | Fibroblasts | RAB7A | 0.41 | | 8.28E-39 | |
| Fibroblasts | Fibroblasts | CCR7 | -0.89 | | 2.35E-38 | |
| Fibroblasts | Fibroblasts | LXN | 0.25 | | 2.44E-38 | |
| Fibroblasts | Fibroblasts | HIKESHI | 0.26 | | 2.92E-38 | |
| Fibroblasts | Fibroblasts | CTSS | -0.69 | | 3.15E-38 | |
| Fibroblasts | Fibroblasts | HLA-DQA2 | -0.95 | | 3.16E-38 | |
| Fibroblasts | Fibroblasts | YWHAZ | -0.54 | | 3.70E-38 | |
| Fibroblasts | Fibroblasts | AHNAK | 0.35 | | 3.88E-38 | |
| Fibroblasts | Fibroblasts | TMEM230 | 0.34 | | 4.01E-38 | |
| Fibroblasts | Fibroblasts | ANXA2 | 0.49 | | 4.81E-38 | |
| Fibroblasts | Fibroblasts | SUB1 | -0.51 | | 7.08E-38 | |
| Fibroblasts | Fibroblasts | FMC1 | 0.27 | | 7.98E-38 | |
| Fibroblasts | Fibroblasts | SERP1 | -0.55 | | 8.26E-38 | |
| Fibroblasts | Fibroblasts | EVI2A | -0.62 | | 1.11E-37 | |
| Fibroblasts | Fibroblasts | H3F3A | -0.46 | | 1.21E-37 | |
| Fibroblasts | Fibroblasts | CAMLG | 0.27 | | 2.19E-37 | |
| Fibroblasts | Fibroblasts | DPM3 | 0.27 | | 3.05E-37 | |
| Fibroblasts | Fibroblasts | ACTG1 | 0.27 | | 4.15E-37 | |
| Fibroblasts | Fibroblasts | CRIP1 | -0.71 | | 7.78E-37 | |
| Fibroblasts | Fibroblasts | RPL14 | -0.32 | | 8.14E-37 | |
| Fibroblasts | Fibroblasts | AC016831.4 | -0.75 | | 1.21E-36 | |
| Fibroblasts | Fibroblasts | CCL5 | -2.01 | | 2.72E-36 | |
| Fibroblasts | Fibroblasts | CDKN1A | 0.67 | | 3.30E-36 | |
| Fibroblasts | Fibroblasts | PRDX5 | 0.34 | | 4.51E-36 | |
| Fibroblasts | Fibroblasts | TMEM205 | 0.3 | | 5.86E-36 | |
| Fibroblasts | Fibroblasts | PPP1R12A | 0.34 | | 6.85E-36 | |
| Fibroblasts | Fibroblasts | NDUFAF3 | 0.3 | | 8.23E-36 | |
| Fibroblasts | Fibroblasts | RPL9 | -0.31 | | 8.32E-36 | |
| Fibroblasts | Fibroblasts | MPG | 0.31 | | 1.34E-35 | |
| Fibroblasts | Fibroblasts | IFITM1 | 0.3 | | 1.58E-35 | |
| Fibroblasts | Fibroblasts | SDF4 | 0.28 | | 1.76E-35 | |
| Fibroblasts | Fibroblasts | SEC31A | 0.28 | | 1.91E-35 | |
| Fibroblasts | Fibroblasts | DNAJA1 | 0.67 | | 3.21E-35 | |
| Fibroblasts | Fibroblasts | RHOF | -0.55 | | 4.30E-35 | |
| Fibroblasts | Fibroblasts | SLC7A5 | -0.7 | | 5.62E-35 | |
| Fibroblasts | Fibroblasts | RABAC1 | 0.29 | | 1.36E-34 | |
| Fibroblasts | Fibroblasts | ACAP1 | -0.62 | | 1.52E-34 | |
| Fibroblasts | Fibroblasts | NEAT1 | 0.41 | | 2.28E-34 | |
| Fibroblasts | Fibroblasts | LRRFIP1 | -0.67 | | 2.86E-34 | |
| Fibroblasts | Fibroblasts | RPS3A | -0.34 | | 2.90E-34 | |
| Fibroblasts | Fibroblasts | HMGN3 | 0.27 | | 3.88E-34 | |
| Fibroblasts | Fibroblasts | PPP2R5C | -0.73 | | 4.69E-34 | |
| Fibroblasts | Fibroblasts | CD55 | -0.64 | | 1.13E-33 | |
| Fibroblasts | Fibroblasts | RPL13A | -0.26 | | 1.13E-33 | |
| Fibroblasts | Fibroblasts | ARF4 | 0.33 | | 1.39E-33 | |
| Fibroblasts | Fibroblasts | TMEM30A | 0.27 | | 1.48E-33 | |
| Fibroblasts | Fibroblasts | LRPAP1 | 0.26 | | 1.52E-33 | |
| Fibroblasts | Fibroblasts | EIF4A3 | 0.63 | | 1.58E-33 | |
| Fibroblasts | Fibroblasts | ARL6IP5 | 0.27 | | 1.90E-33 | |
| Fibroblasts | Fibroblasts | PARK7 | 0.31 | | 2.00E-33 | |
| Fibroblasts | Fibroblasts | SLC38A2 | 0.39 | | 2.74E-33 | |
| Fibroblasts | Fibroblasts | BHLHE40 | 0.41 | | 3.60E-33 | |
| Fibroblasts | Fibroblasts | DAD1 | 0.34 | | 4.19E-33 | |
| Fibroblasts | Fibroblasts | TNFRSF1B | -0.65 | | 4.22E-33 | |
| Fibroblasts | Fibroblasts | ERH | 0.34 | | 5.68E-33 | |
| Fibroblasts | Fibroblasts | YIF1A | 0.28 | | 5.91E-33 | |
| Fibroblasts | Fibroblasts | SEC62 | 0.35 | | 6.17E-33 | |
| Fibroblasts | Fibroblasts | MAT2A | 0.38 | | 9.45E-33 | |
| Fibroblasts | Fibroblasts | LY9 | -0.81 | | 1.08E-32 | |
| Fibroblasts | Fibroblasts | VPS37B | -0.64 | | 1.08E-32 | |
| Fibroblasts | Fibroblasts | PRDX2 | 0.3 | | 1.55E-32 | |
| Fibroblasts | Fibroblasts | SAT2 | 0.27 | | 1.69E-32 | |
| Fibroblasts | Fibroblasts | PPP1R15A | 0.66 | | 2.24E-32 | |
| Fibroblasts | Fibroblasts | CPM | 0.36 | | 2.66E-32 | |
| Fibroblasts | Fibroblasts | ZFAND5 | 0.35 | | 3.11E-32 | |
| Fibroblasts | Fibroblasts | SEC11A | 0.26 | | 3.51E-32 | |
| Fibroblasts | Fibroblasts | SH3BGRL | 0.38 | | 4.05E-32 | |
| Fibroblasts | Fibroblasts | ITGB2 | -0.6 | | 5.82E-32 | |
| Fibroblasts | Fibroblasts | HSP90AB1 | 0.3 | | 6.15E-32 | |
| Fibroblasts | Fibroblasts | ARPC1A | 0.32 | | 6.20E-32 | |
| Fibroblasts | Fibroblasts | ATP5PD | 0.28 | | 6.83E-32 | |
| Fibroblasts | Fibroblasts | LUC7L3 | 0.32 | | 9.23E-32 | |
| Fibroblasts | Fibroblasts | SPINT2 | -0.57 | | 1.05E-31 | |
| Fibroblasts | Fibroblasts | PLAC8 | -0.58 | | 1.27E-31 | |
| Fibroblasts | Fibroblasts | ATP5F1E | -0.35 | | 1.37E-31 | |
| Fibroblasts | Fibroblasts | RHOG | -0.66 | | 1.42E-31 | |
| Fibroblasts | Fibroblasts | PEPD | 0.25 | | 1.46E-31 | |
| Fibroblasts | Fibroblasts | NKG7 | -1.44 | | 1.75E-31 | |
| Fibroblasts | Fibroblasts | TPD52 | -0.44 | | 2.09E-31 | |
| Fibroblasts | Fibroblasts | GPRC5A | 0.28 | | 2.14E-31 | |
| Fibroblasts | Fibroblasts | MORF4L2 | 0.3 | | 2.53E-31 | |
| Fibroblasts | Fibroblasts | DYNLRB1 | 0.28 | | 2.84E-31 | |
| Fibroblasts | Fibroblasts | AL121944.1 | -0.59 | | 3.17E-31 | |
| Fibroblasts | Fibroblasts | TPM3 | -0.58 | | 3.55E-31 | |
| Fibroblasts | Fibroblasts | SRSF2 | -0.55 | | 5.24E-31 | |
| Fibroblasts | Fibroblasts | FIS1 | 0.31 | | 7.04E-31 | |
| Fibroblasts | Fibroblasts | ARPC3 | -0.49 | | 1.44E-30 | |
| Fibroblasts | Fibroblasts | CAMTA1 | 0.29 | | 1.56E-30 | |
| Fibroblasts | Fibroblasts | ANXA6 | 0.25 | | 1.64E-30 | |
| Fibroblasts | Fibroblasts | RPS8 | -0.31 | | 3.84E-30 | |
| Fibroblasts | Fibroblasts | LPXN | -0.47 | | 3.92E-30 | |
| Fibroblasts | Fibroblasts | SYNGR2 | -0.52 | | 4.34E-30 | |
| Fibroblasts | Fibroblasts | TPT1 | -0.28 | | 6.19E-30 | |
| Fibroblasts | Fibroblasts | EIF4A2 | 0.31 | | 6.41E-30 | |
| Fibroblasts | Fibroblasts | RPL34 | -0.29 | | 6.71E-30 | |
| Fibroblasts | Fibroblasts | C4orf3 | 0.33 | | 6.78E-30 | |
| Fibroblasts | Fibroblasts | EIF1 | -0.27 | | 9.00E-30 | |
| Fibroblasts | Fibroblasts | SMIM26 | 0.26 | | 9.44E-30 | |
| Fibroblasts | Fibroblasts | ST13 | 0.29 | | 3.05E-29 | |
| Fibroblasts | Fibroblasts | MIDN | 0.3 | | 3.97E-29 | |
| Fibroblasts | Fibroblasts | CCNL1 | 0.5 | | 4.17E-29 | |
| Fibroblasts | Fibroblasts | PRNP | 0.27 | | 4.46E-29 | |
| Fibroblasts | Fibroblasts | CD83 | -1.01 | | 4.63E-29 | |
| Fibroblasts | Fibroblasts | MGAT4A | -0.56 | | 4.66E-29 | |
| Fibroblasts | Fibroblasts | RPL29 | -0.27 | | 5.09E-29 | |
| Fibroblasts | Fibroblasts | EVL | -0.64 | | 5.41E-29 | |
| Fibroblasts | Fibroblasts | PDE4B | -0.62 | | 6.26E-29 | |
| Fibroblasts | Fibroblasts | LCK | -0.61 | | 6.38E-29 | |
| Fibroblasts | Fibroblasts | CIRBP | 0.29 | | 6.49E-29 | |
| Fibroblasts | Fibroblasts | SLC38A1 | -0.5 | | 7.32E-29 | |
| Fibroblasts | Fibroblasts | ZNHIT1 | 0.25 | | 8.52E-29 | |
| Fibroblasts | Fibroblasts | DYNLT1 | 0.31 | | 9.79E-29 | |
| Fibroblasts | Fibroblasts | MINOS1 | 0.27 | | 1.00E-28 | |
| Fibroblasts | Fibroblasts | ATOX1 | 0.28 | | 1.42E-28 | |
| Fibroblasts | Fibroblasts | STAT4 | -0.58 | | 1.76E-28 | |
| Fibroblasts | Fibroblasts | WIPF1 | -0.63 | | 1.81E-28 | |
| Fibroblasts | Fibroblasts | IL7R | -0.88 | | 1.88E-28 | |
| Fibroblasts | Fibroblasts | FMNL1 | -0.55 | | 1.98E-28 | |
| Fibroblasts | Fibroblasts | CD27 | -0.62 | | 2.76E-28 | |
| Fibroblasts | Fibroblasts | RPL3 | -0.25 | | 2.92E-28 | |
| Fibroblasts | Fibroblasts | TLN1 | 0.27 | | 3.14E-28 | |
| Fibroblasts | Fibroblasts | JPT1 | -0.63 | | 4.90E-28 | |
| Fibroblasts | Fibroblasts | SMAP2 | -0.46 | | 5.45E-28 | |
| Fibroblasts | Fibroblasts | RUNX3 | -0.55 | | 8.21E-28 | |
| Fibroblasts | Fibroblasts | NDUFA11 | 0.3 | | 8.92E-28 | |
| Fibroblasts | Fibroblasts | ARHGAP9 | -0.51 | | 1.02E-27 | |
| Fibroblasts | Fibroblasts | FYB1 | -0.6 | | 1.12E-27 | |
| Fibroblasts | Fibroblasts | NDUFB7 | 0.26 | | 1.13E-27 | |
| Fibroblasts | Fibroblasts | FNBP1 | -0.62 | | 1.36E-27 | |
| Fibroblasts | Fibroblasts | ARL5B | 0.27 | | 1.66E-27 | |
| Fibroblasts | Fibroblasts | HSP90AA1 | 0.33 | | 1.85E-27 | |
| Fibroblasts | Fibroblasts | KLRB1 | -1.21 | | 2.43E-27 | |
| Fibroblasts | Fibroblasts | SKP1 | 0.26 | | 2.99E-27 | |
| Fibroblasts | Fibroblasts | VMP1 | 0.33 | | 3.14E-27 | |
| Fibroblasts | Fibroblasts | NCF1 | -0.44 | | 3.57E-27 | |
| Fibroblasts | Fibroblasts | SEC11C | -0.87 | | 3.64E-27 | |
| Fibroblasts | Fibroblasts | P4HB | 0.3 | | 4.23E-27 | |
| Fibroblasts | Fibroblasts | GZMA | -1.21 | | 5.22E-27 | |
| Fibroblasts | Fibroblasts | HINT1 | -0.39 | | 5.65E-27 | |
| Fibroblasts | Fibroblasts | RWDD1 | 0.27 | | 6.14E-27 | |
| Fibroblasts | Fibroblasts | CNPY2 | 0.27 | | 7.29E-27 | |
| Fibroblasts | Fibroblasts | STXBP2 | -0.4 | | 8.83E-27 | |
| Fibroblasts | Fibroblasts | RASSF5 | -0.52 | | 1.10E-26 | |
| Fibroblasts | Fibroblasts | PTPN7 | -0.5 | | 1.39E-26 | |
| Fibroblasts | Fibroblasts | DHRS3 | 0.27 | | 1.55E-26 | |
| Fibroblasts | Fibroblasts | GZMM | -0.64 | | 1.75E-26 | |
| Fibroblasts | Fibroblasts | ATF4 | 0.3 | | 1.89E-26 | |
| Fibroblasts | Fibroblasts | FAM177A1 | -0.71 | | 2.40E-26 | |
| Fibroblasts | Fibroblasts | TINAGL1 | 0.69 | | 3.22E-26 | |
| Fibroblasts | Fibroblasts | NEDD8 | 0.28 | | 3.27E-26 | |
| Fibroblasts | Fibroblasts | HLA-DMB | -0.59 | | 5.57E-26 | |
| Fibroblasts | Fibroblasts | IL10RA | -0.43 | | 8.16E-26 | |
| Fibroblasts | Fibroblasts | AC114760.2 | -0.54 | | 9.83E-26 | |
| Fibroblasts | Fibroblasts | PDIA3 | 0.3 | | 1.30E-25 | |
| Fibroblasts | Fibroblasts | MYH9 | 0.27 | | 1.40E-25 | |
| Fibroblasts | Fibroblasts | ARF6 | -0.55 | | 1.64E-25 | |
| Fibroblasts | Fibroblasts | SYTL1 | -0.35 | | 2.12E-25 | |
| Fibroblasts | Fibroblasts | MS4A1 | -0.75 | | 2.57E-25 | |
| Fibroblasts | Fibroblasts | PTPN22 | -0.51 | | 3.07E-25 | |
| Fibroblasts | Fibroblasts | HMGA1 | -0.54 | | 3.27E-25 | |
| Fibroblasts | Fibroblasts | CIB1 | -0.53 | | 4.70E-25 | |
| Fibroblasts | Fibroblasts | RPL31 | -0.29 | | 5.29E-25 | |
| Fibroblasts | Fibroblasts | ELOC | 0.34 | | 5.69E-25 | |
| Fibroblasts | Fibroblasts | TRABD | -0.46 | | 5.74E-25 | |
| Fibroblasts | Fibroblasts | TNFAIP3 | -0.67 | | 7.16E-25 | |
| Fibroblasts | Fibroblasts | REL | -0.71 | | 7.28E-25 | |
| Fibroblasts | Fibroblasts | SERPINB9 | -0.65 | | 1.28E-24 | |
| Fibroblasts | Fibroblasts | P2RY10 | -0.43 | | 2.09E-24 | |
| Fibroblasts | Fibroblasts | TSTD1 | -0.45 | | 2.31E-24 | |
| Fibroblasts | Fibroblasts | PDCD4 | -0.66 | | 3.52E-24 | |
| Fibroblasts | Fibroblasts | HEXIM1 | 0.28 | | 7.12E-24 | |
| Fibroblasts | Fibroblasts | FYN | -0.79 | | 9.57E-24 | |
| Fibroblasts | Fibroblasts | CD96 | -0.53 | | 1.13E-23 | |
| Fibroblasts | Fibroblasts | CNOT6L | -0.57 | | 1.32E-23 | |
| Fibroblasts | Fibroblasts | ODC1 | -0.54 | | 1.68E-23 | |
| Fibroblasts | Fibroblasts | UAP1 | 0.3 | | 1.98E-23 | |
| Fibroblasts | Fibroblasts | WAS | -0.39 | | 2.12E-23 | |
| Fibroblasts | Fibroblasts | EMB | -0.41 | | 2.40E-23 | |
| Fibroblasts | Fibroblasts | RPL36 | -0.26 | | 2.52E-23 | |
| Fibroblasts | Fibroblasts | LAP3 | 0.26 | | 2.96E-23 | |
| Fibroblasts | Fibroblasts | WDR83OS | 0.26 | | 5.58E-23 | |
| Fibroblasts | Fibroblasts | RAB11FIP1 | -0.38 | | 5.91E-23 | |
| Fibroblasts | Fibroblasts | TENT5C | -0.56 | | 7.16E-23 | |
| Fibroblasts | Fibroblasts | TBC1D10C | -0.42 | | 9.08E-23 | |
| Fibroblasts | Fibroblasts | PMAIP1 | -0.54 | | 9.43E-23 | |
| Fibroblasts | Fibroblasts | G3BP2 | -0.57 | | 9.69E-23 | |
| Fibroblasts | Fibroblasts | CD3G | -0.53 | | 1.30E-22 | |
| Fibroblasts | Fibroblasts | TUBA1B | 0.28 | | 1.36E-22 | |
| Fibroblasts | Fibroblasts | PIM2 | -0.6 | | 1.43E-22 | |
| Fibroblasts | Fibroblasts | LBH | 0.35 | | 1.45E-22 | |
| Fibroblasts | Fibroblasts | RPS10 | -0.4 | | 1.52E-22 | |
| Fibroblasts | Fibroblasts | IDS | -0.57 | | 1.60E-22 | |
| Fibroblasts | Fibroblasts | ITGB7 | -0.35 | | 1.68E-22 | |
| Fibroblasts | Fibroblasts | C16orf54 | -0.46 | | 2.62E-22 | |
| Fibroblasts | Fibroblasts | PHLDA2 | 0.27 | | 2.76E-22 | |
| Fibroblasts | Fibroblasts | ARID5B | 0.31 | | 3.06E-22 | |
| Fibroblasts | Fibroblasts | SELENOT | -0.49 | | 3.13E-22 | |
| Fibroblasts | Fibroblasts | NUDT4 | 0.27 | | 3.25E-22 | |
| Fibroblasts | Fibroblasts | PPP1CA | -0.48 | | 3.69E-22 | |
| Fibroblasts | Fibroblasts | RILPL2 | -0.63 | | 4.24E-22 | |
| Fibroblasts | Fibroblasts | SLC2A3 | 0.39 | | 4.53E-22 | |
| Fibroblasts | Fibroblasts | RPL4 | -0.3 | | 4.91E-22 | |
| Fibroblasts | Fibroblasts | SAT1 | -0.74 | | 5.87E-22 | |
| Fibroblasts | Fibroblasts | CYTH1 | -0.42 | | 6.45E-22 | |
| Fibroblasts | Fibroblasts | ARHGAP45 | -0.37 | | 9.90E-22 | |
| Fibroblasts | Fibroblasts | LYN | -0.38 | | 1.41E-21 | |
| Fibroblasts | Fibroblasts | SH2D2A | -0.56 | | 1.63E-21 | |
| Fibroblasts | Fibroblasts | EML4 | -0.55 | | 1.70E-21 | |
| Fibroblasts | Fibroblasts | SYAP1 | -0.57 | | 2.91E-21 | |
| Fibroblasts | Fibroblasts | CD6 | -0.49 | | 3.90E-21 | |
| Fibroblasts | Fibroblasts | SLA | -0.51 | | 3.93E-21 | |
| Fibroblasts | Fibroblasts | STK17A | -0.55 | | 4.24E-21 | |
| Fibroblasts | Fibroblasts | CD247 | -0.52 | | 4.45E-21 | |
| Fibroblasts | Fibroblasts | SEL1L3 | -0.31 | | 7.15E-21 | |
| Fibroblasts | Fibroblasts | ARL4C | -0.58 | | 8.36E-21 | |
| Fibroblasts | Fibroblasts | MZB1 | -1.75 | | 1.00E-20 | |
| Fibroblasts | Fibroblasts | CCNI | 0.28 | | 1.57E-20 | |
| Fibroblasts | Fibroblasts | CMTM7 | -0.4 | | 1.70E-20 | |
| Fibroblasts | Fibroblasts | ICOS | -0.55 | | 1.84E-20 | |
| Fibroblasts | Fibroblasts | BIK | -0.33 | | 1.84E-20 | |
| Fibroblasts | Fibroblasts | NAMPT | 0.27 | | 2.36E-20 | |
| Fibroblasts | Fibroblasts | BCL2A1 | -0.84 | | 2.85E-20 | |
| Fibroblasts | Fibroblasts | BRD2 | 0.29 | | 2.95E-20 | |
| Fibroblasts | Fibroblasts | CTSW | -0.63 | | 2.99E-20 | |
| Fibroblasts | Fibroblasts | CAPG | -0.61 | | 3.25E-20 | |
| Fibroblasts | Fibroblasts | BANK1 | -0.51 | | 3.68E-20 | |
| Fibroblasts | Fibroblasts | ICAM2 | -0.42 | | 4.24E-20 | |
| Fibroblasts | Fibroblasts | CD8A | -0.76 | | 5.77E-20 | |
| Fibroblasts | Fibroblasts | TMC6 | -0.32 | | 6.99E-20 | |
| Fibroblasts | Fibroblasts | RPL38 | -0.27 | | 7.09E-20 | |
| Fibroblasts | Fibroblasts | IL2RB | -0.46 | | 1.09E-19 | |
| Fibroblasts | Fibroblasts | STX11 | -0.45 | | 1.32E-19 | |
| Fibroblasts | Fibroblasts | MT-CO2 | -0.53 | | 1.53E-19 | |
| Fibroblasts | Fibroblasts | ARL4A | -0.5 | | 2.07E-19 | |
| Fibroblasts | Fibroblasts | GIMAP7 | -0.5 | | 4.56E-19 | |
| Fibroblasts | Fibroblasts | ARPC2 | -0.43 | | 4.89E-19 | |
| Fibroblasts | Fibroblasts | ARID5A | -0.47 | | 5.11E-19 | |
| Fibroblasts | Fibroblasts | NDUFA4 | 0.39 | | 5.20E-19 | |
| Fibroblasts | Fibroblasts | GNG7 | -0.41 | | 6.36E-19 | |
| Fibroblasts | Fibroblasts | HMGN1 | -0.44 | | 6.62E-19 | |
| Fibroblasts | Fibroblasts | PRKCB | -0.34 | | 7.17E-19 | |
| Fibroblasts | Fibroblasts | DERL3 | -1.09 | | 7.23E-19 | |
| Fibroblasts | Fibroblasts | CLEC2D | -0.42 | | 7.42E-19 | |
| Fibroblasts | Fibroblasts | LY86 | -0.36 | | 1.13E-18 | |
| Fibroblasts | Fibroblasts | CD79B | -0.39 | | 1.57E-18 | |
| Fibroblasts | Fibroblasts | COX4I1 | -0.27 | | 1.63E-18 | |
| Fibroblasts | Fibroblasts | GZMB | -1.26 | | 1.85E-18 | |
| Fibroblasts | Fibroblasts | LAT | -0.43 | | 1.87E-18 | |
| Fibroblasts | Fibroblasts | SLAMF7 | -0.29 | | 1.93E-18 | |
| Fibroblasts | Fibroblasts | VPREB3 | -0.5 | | 2.02E-18 | |
| Fibroblasts | Fibroblasts | BIN2 | -0.32 | | 2.12E-18 | |
| Fibroblasts | Fibroblasts | SELL | -0.44 | | 2.52E-18 | |
| Fibroblasts | Fibroblasts | FUS | 0.26 | | 2.95E-18 | |
| Fibroblasts | Fibroblasts | GRB2 | -0.4 | | 3.82E-18 | |
| Fibroblasts | Fibroblasts | AC058791.1 | -0.54 | | 7.42E-18 | |
| Fibroblasts | Fibroblasts | TNFRSF18 | -0.57 | | 7.68E-18 | |
| Fibroblasts | Fibroblasts | ABRACL | -0.46 | | 1.11E-17 | |
| Fibroblasts | Fibroblasts | LYZ | -1.76 | | 1.24E-17 | |
| Fibroblasts | Fibroblasts | TNFRSF4 | -0.7 | | 2.56E-17 | |
| Fibroblasts | Fibroblasts | S1PR4 | -0.36 | | 3.17E-17 | |
| Fibroblasts | Fibroblasts | ARHGAP30 | -0.31 | | 3.47E-17 | |
| Fibroblasts | Fibroblasts | ARHGAP4 | -0.29 | | 4.04E-17 | |
| Fibroblasts | Fibroblasts | POU2AF1 | -0.26 | | 4.51E-17 | |
| Fibroblasts | Fibroblasts | TIGIT | -0.6 | | 4.68E-17 | |
| Fibroblasts | Fibroblasts | P2RY8 | -0.33 | | 5.04E-17 | |
| Fibroblasts | Fibroblasts | CD19 | -0.33 | | 9.25E-17 | |
| Fibroblasts | Fibroblasts | AC004687.1 | -0.33 | | 9.26E-17 | |
| Fibroblasts | Fibroblasts | RNF125 | -0.38 | | 1.07E-16 | |
| Fibroblasts | Fibroblasts | ATP5MG | -0.27 | | 1.15E-16 | |
| Fibroblasts | Fibroblasts | GPR65 | -0.35 | | 1.23E-16 | |
| Fibroblasts | Fibroblasts | TRAT1 | -0.44 | | 1.24E-16 | |
| Fibroblasts | Fibroblasts | MYO1G | -0.31 | | 1.32E-16 | |
| Fibroblasts | Fibroblasts | GZMK | -1.09 | | 1.34E-16 | |
| Fibroblasts | Fibroblasts | DOK2 | -0.35 | | 2.03E-16 | |
| Fibroblasts | Fibroblasts | RBM38 | -0.33 | | 2.21E-16 | |
| Fibroblasts | Fibroblasts | SP140 | -0.29 | | 3.20E-16 | |
| Fibroblasts | Fibroblasts | 6-Sep | -0.46 | | 3.89E-16 | |
| Fibroblasts | Fibroblasts | NCF4 | -0.25 | | 5.49E-16 | |
| Fibroblasts | Fibroblasts | PRF1 | -0.64 | | 6.36E-16 | |
| Fibroblasts | Fibroblasts | IKZF1 | -0.3 | | 8.27E-16 | |
| Fibroblasts | Fibroblasts | LINC01871 | -0.53 | | 8.66E-16 | |
| Fibroblasts | Fibroblasts | SYTL3 | -0.4 | | 9.34E-16 | |
| Fibroblasts | Fibroblasts | SS18L2 | -0.43 | | 1.05E-15 | |
| Fibroblasts | Fibroblasts | JSRP1 | -0.47 | | 1.33E-15 | |
| Fibroblasts | Fibroblasts | GNG2 | -0.59 | | 1.60E-15 | |
| Fibroblasts | Fibroblasts | RASAL3 | -0.28 | | 3.11E-15 | |
| Fibroblasts | Fibroblasts | CCR6 | -0.36 | | 4.84E-15 | |
| Fibroblasts | Fibroblasts | SMIM22 | -0.5 | | 7.14E-15 | |
| Fibroblasts | Fibroblasts | PGK1 | -0.48 | | 7.78E-15 | |
| Fibroblasts | Fibroblasts | PRKCH | -0.36 | | 8.41E-15 | |
| Fibroblasts | Fibroblasts | ATP2B1 | -0.49 | | 1.08E-14 | |
| Fibroblasts | Fibroblasts | DEF6 | -0.26 | | 1.09E-14 | |
| Fibroblasts | Fibroblasts | GZMH | -0.78 | | 1.34E-14 | |
| Fibroblasts | Fibroblasts | SVIP | -0.39 | | 1.78E-14 | |
| Fibroblasts | Fibroblasts | SLC16A3 | -0.41 | | 2.31E-14 | |
| Fibroblasts | Fibroblasts | TMSB4X | -0.37 | | 2.87E-14 | |
| Fibroblasts | Fibroblasts | SKAP1 | -0.29 | | 2.90E-14 | |
| Fibroblasts | Fibroblasts | PIGR | -0.8 | | 3.24E-14 | |
| Fibroblasts | Fibroblasts | PPP1CB | -0.52 | | 3.68E-14 | |
| Fibroblasts | Fibroblasts | JAML | -0.35 | | 3.84E-14 | |
| Fibroblasts | Fibroblasts | SNRPB | -0.38 | | 3.99E-14 | |
| Fibroblasts | Fibroblasts | LCN2 | -0.81 | | 4.04E-14 | |
| Fibroblasts | Fibroblasts | DOCK8 | -0.28 | | 4.08E-14 | |
| Fibroblasts | Fibroblasts | PIM1 | 0.31 | | 4.48E-14 | |
| Fibroblasts | Fibroblasts | FAM107B | -0.35 | | 5.33E-14 | |
| Fibroblasts | Fibroblasts | AKNA | -0.4 | | 6.28E-14 | |
| Fibroblasts | Fibroblasts | CSRNP1 | 0.3 | | 6.91E-14 | |
| Fibroblasts | Fibroblasts | SRSF7 | -0.44 | | 6.93E-14 | |
| Fibroblasts | Fibroblasts | TSPAN13 | -0.27 | | 9.78E-14 | |
| Fibroblasts | Fibroblasts | TYROBP | -1.34 | | 1.06E-13 | |
| Fibroblasts | Fibroblasts | CAPZA1 | -0.41 | | 1.20E-13 | |
| Fibroblasts | Fibroblasts | ZNF165 | -0.26 | | 1.24E-13 | |
| Fibroblasts | Fibroblasts | CXCR3 | -0.42 | | 1.40E-13 | |
| Fibroblasts | Fibroblasts | ABI3 | -0.32 | | 1.47E-13 | |
| Fibroblasts | Fibroblasts | ARHGEF1 | -0.4 | | 1.52E-13 | |
| Fibroblasts | Fibroblasts | TNFAIP8 | -0.34 | | 1.60E-13 | |
| Fibroblasts | Fibroblasts | SPCS3 | -0.52 | | 1.69E-13 | |
| Fibroblasts | Fibroblasts | TAGLN2 | -0.32 | | 1.89E-13 | |
| Fibroblasts | Fibroblasts | AC020656.1 | -0.45 | | 2.54E-13 | |
| Fibroblasts | Fibroblasts | PRR13 | -0.41 | | 2.54E-13 | |
| Fibroblasts | Fibroblasts | FABP5 | -0.5 | | 3.53E-13 | |
| Fibroblasts | Fibroblasts | ELF3 | -0.74 | | 3.54E-13 | |
| Fibroblasts | Fibroblasts | SPINK1 | -1.19 | | 4.00E-13 | |
| Fibroblasts | Fibroblasts | CDC42 | -0.36 | | 5.07E-13 | |
| Fibroblasts | Fibroblasts | C19orf33 | -0.48 | | 5.36E-13 | |
| Fibroblasts | Fibroblasts | MAPK1IP1L | -0.42 | | 5.61E-13 | |
| Fibroblasts | Fibroblasts | CFL1 | -0.26 | | 5.73E-13 | |
| Fibroblasts | Fibroblasts | SELPLG | -0.29 | | 6.98E-13 | |
| Fibroblasts | Fibroblasts | CD38 | -0.31 | | 7.66E-13 | |
| Fibroblasts | Fibroblasts | TNFRSF25 | -0.33 | | 8.83E-13 | |
| Fibroblasts | Fibroblasts | LDLRAD4 | -0.53 | | 9.42E-13 | |
| Fibroblasts | Fibroblasts | KRT19 | -0.86 | | 1.19E-12 | |
| Fibroblasts | Fibroblasts | POU2F2 | -0.25 | | 1.32E-12 | |
| Fibroblasts | Fibroblasts | SPI1 | -0.26 | | 1.46E-12 | |
| Fibroblasts | Fibroblasts | SATB1 | -0.37 | | 1.57E-12 | |
| Fibroblasts | Fibroblasts | RELB | -0.36 | | 1.61E-12 | |
| Fibroblasts | Fibroblasts | RCAN3 | -0.28 | | 2.46E-12 | |
| Fibroblasts | Fibroblasts | PDCL3 | -0.45 | | 3.04E-12 | |
| Fibroblasts | Fibroblasts | WDR74 | -0.42 | | 3.21E-12 | |
| Fibroblasts | Fibroblasts | ARRB2 | -0.27 | | 3.49E-12 | |
| Fibroblasts | Fibroblasts | BATF | -0.43 | | 4.85E-12 | |
| Fibroblasts | Fibroblasts | AIF1 | -0.68 | | 6.92E-12 | |
| Fibroblasts | Fibroblasts | S100A14 | -0.43 | | 7.47E-12 | |
| Fibroblasts | Fibroblasts | AC044849.1 | -0.29 | | 7.93E-12 | |
| Fibroblasts | Fibroblasts | ATP1B3 | -0.47 | | 9.47E-12 | |
| Fibroblasts | Fibroblasts | APBB1IP | -0.28 | | 1.00E-11 | |
| Fibroblasts | Fibroblasts | JOSD1 | -0.32 | | 1.74E-11 | |
| Fibroblasts | Fibroblasts | PCGF5 | -0.29 | | 2.06E-11 | |
| Fibroblasts | Fibroblasts | CLDN4 | -0.55 | | 2.34E-11 | |
| Fibroblasts | Fibroblasts | PLAUR | -0.46 | | 2.88E-11 | |
| Fibroblasts | Fibroblasts | MIR155HG | -0.33 | | 3.05E-11 | |
| Fibroblasts | Fibroblasts | EPCAM | -0.41 | | 3.14E-11 | |
| Fibroblasts | Fibroblasts | GSPT1 | -0.4 | | 3.21E-11 | |
| Fibroblasts | Fibroblasts | CASP8 | -0.25 | | 3.29E-11 | |
| Fibroblasts | Fibroblasts | ITGA4 | -0.27 | | 5.51E-11 | |
| Fibroblasts | Fibroblasts | IL16 | -0.26 | | 6.19E-11 | |
| Fibroblasts | Fibroblasts | PAPOLA | -0.36 | | 7.70E-11 | |
| Fibroblasts | Fibroblasts | GPX2 | -0.33 | | 9.73E-11 | |
| Fibroblasts | Fibroblasts | SMCHD1 | -0.4 | | 1.06E-10 | |
| Fibroblasts | Fibroblasts | LST1 | -0.46 | | 1.22E-10 | |
| Fibroblasts | Fibroblasts | FCER1G | -1.44 | | 1.23E-10 | |
| Fibroblasts | Fibroblasts | HMGB2 | -0.5 | | 1.38E-10 | |
| Fibroblasts | Fibroblasts | PPP1R2 | -0.4 | | 1.45E-10 | |
| Fibroblasts | Fibroblasts | ARHGAP15 | 0.29 | | 1.53E-10 | |
| Fibroblasts | Fibroblasts | COX5A | -0.38 | | 1.86E-10 | |
| Fibroblasts | Fibroblasts | RASGEF1B | -0.35 | | 3.17E-10 | |
| Fibroblasts | Fibroblasts | IL1RN | -0.68 | | 3.22E-10 | |
| Fibroblasts | Fibroblasts | SIAH2 | -0.29 | | 4.15E-10 | |
| Fibroblasts | Fibroblasts | HERPUD1 | -0.96 | | 4.56E-10 | |
| Fibroblasts | Fibroblasts | RBM39 | -0.32 | | 6.82E-10 | |
| Fibroblasts | Fibroblasts | ANKRD44 | -0.27 | | 7.24E-10 | |
| Fibroblasts | Fibroblasts | PNP | -0.31 | | 7.63E-10 | |
| Fibroblasts | Fibroblasts | EFHD2 | -0.35 | | 1.07E-09 | |
| Fibroblasts | Fibroblasts | CALM1 | -0.28 | | 1.08E-09 | |
| Fibroblasts | Fibroblasts | TXN | -0.26 | | 1.14E-09 | |
| Fibroblasts | Fibroblasts | SNU13 | -0.32 | | 1.17E-09 | |
| Fibroblasts | Fibroblasts | MOB1A | -0.35 | | 1.30E-09 | |
| Fibroblasts | Fibroblasts | C15orf48 | -1.2 | | 1.33E-09 | |
| Fibroblasts | Fibroblasts | GNA13 | -0.28 | | 1.37E-09 | |
| Fibroblasts | Fibroblasts | PTP4A1 | -0.37 | | 1.37E-09 | |
| Fibroblasts | Fibroblasts | DUSP1 | 0.33 | | 1.97E-09 | |
| Fibroblasts | Fibroblasts | VSIG2 | -0.32 | | 2.35E-09 | |
| Fibroblasts | Fibroblasts | CFLAR | -0.39 | | 2.38E-09 | |
| Fibroblasts | Fibroblasts | VOPP1 | -0.29 | | 2.44E-09 | |
| Fibroblasts | Fibroblasts | FCMR | -0.33 | | 3.44E-09 | |
| Fibroblasts | Fibroblasts | PRELID3B | -0.26 | | 3.63E-09 | |
| Fibroblasts | Fibroblasts | HIST1H1C | -0.48 | | 4.64E-09 | |
| Fibroblasts | Fibroblasts | TESC | -0.26 | | 7.40E-09 | |
| Fibroblasts | Fibroblasts | SRSF5 | -0.32 | | 1.09E-08 | |
| Fibroblasts | Fibroblasts | IL27RA | -0.26 | | 1.10E-08 | |
| Fibroblasts | Fibroblasts | APOBEC3G | -0.39 | | 1.25E-08 | |
| Fibroblasts | Fibroblasts | IDH2 | -0.28 | | 1.72E-08 | |
| Fibroblasts | Fibroblasts | DDX6 | -0.26 | | 1.74E-08 | |
| Fibroblasts | Fibroblasts | SC5D | -0.35 | | 1.88E-08 | |
| Fibroblasts | Fibroblasts | BID | -0.26 | | 2.27E-08 | |
| Fibroblasts | Fibroblasts | ANXA1 | -0.37 | | 2.33E-08 | |
| Fibroblasts | Fibroblasts | PSCA | -0.86 | | 2.36E-08 | |
| Fibroblasts | Fibroblasts | CCL20 | -0.81 | | 2.36E-08 | |
| Fibroblasts | Fibroblasts | MT1X | 0.5 | | 2.40E-08 | |
| Fibroblasts | Fibroblasts | AQP3 | -0.26 | | 2.84E-08 | |
| Fibroblasts | Fibroblasts | LAG3 | -0.41 | | 3.09E-08 | |
| Fibroblasts | Fibroblasts | UBALD2 | -0.27 | | 3.74E-08 | |
| Fibroblasts | Fibroblasts | ANKRD12 | -0.4 | | 3.78E-08 | |
| Fibroblasts | Fibroblasts | NANS | -0.3 | | 4.37E-08 | |
| Fibroblasts | Fibroblasts | IDI1 | -0.38 | | 5.02E-08 | |
| Fibroblasts | Fibroblasts | ABT1 | -0.26 | | 5.16E-08 | |
| Fibroblasts | Fibroblasts | DCXR | -0.31 | | 5.19E-08 | |
| Fibroblasts | Fibroblasts | ANKRD28 | -0.49 | | 6.14E-08 | |
| Fibroblasts | Fibroblasts | MT-CO1 | -0.45 | | 7.41E-08 | |
| Fibroblasts | Fibroblasts | 1-Sep | -0.35 | | 7.84E-08 | |
| Fibroblasts | Fibroblasts | ATP2B1-AS1 | -0.3 | | 8.29E-08 | |
| Fibroblasts | Fibroblasts | TMEM123 | -0.42 | | 8.46E-08 | |
| Fibroblasts | Fibroblasts | PDE7A | -0.27 | | 8.81E-08 | |
| Fibroblasts | Fibroblasts | G0S2 | -0.91 | | 1.15E-07 | |
| Fibroblasts | Fibroblasts | CTSH | -0.37 | | 1.21E-07 | |
| Fibroblasts | Fibroblasts | ZNF331 | -0.33 | | 1.24E-07 | |
| Fibroblasts | Fibroblasts | EIF1AY | -0.29 | | 1.40E-07 | |
| Fibroblasts | Fibroblasts | ANXA10 | -0.44 | | 1.81E-07 | |
| Fibroblasts | Fibroblasts | ALG13 | -0.37 | | 2.01E-07 | |
| Fibroblasts | Fibroblasts | TGFB1 | -0.49 | | 2.24E-07 | |
| Fibroblasts | Fibroblasts | CMTM6 | -0.37 | | 2.54E-07 | |
| Fibroblasts | Fibroblasts | NFKBIA | 0.3 | | 3.06E-07 | |
| Fibroblasts | Fibroblasts | RNF19A | -0.31 | | 3.59E-07 | |
| Fibroblasts | Fibroblasts | ARPP19 | -0.31 | | 3.64E-07 | |
| Fibroblasts | Fibroblasts | MUC1 | -0.54 | | 3.89E-07 | |
| Fibroblasts | Fibroblasts | TRAF4 | -0.25 | | 4.74E-07 | |
| Fibroblasts | Fibroblasts | RAB5IF | -0.3 | | 4.89E-07 | |
| Fibroblasts | Fibroblasts | ORAI2 | -0.28 | | 4.89E-07 | |
| Fibroblasts | Fibroblasts | LINC00513 | -0.3 | | 5.20E-07 | |
| Fibroblasts | Fibroblasts | RPL22L1 | -0.32 | | 5.42E-07 | |
| Fibroblasts | Fibroblasts | NFKBID | -0.34 | | 5.52E-07 | |
| Fibroblasts | Fibroblasts | GDF15 | 0.38 | | 6.31E-07 | |
| Fibroblasts | Fibroblasts | S100P | -0.9 | | 7.21E-07 | |
| Fibroblasts | Fibroblasts | LGALS4 | -0.59 | | 7.50E-07 | |
| Fibroblasts | Fibroblasts | EBP | -0.26 | | 8.21E-07 | |
| Fibroblasts | Fibroblasts | GLIPR1 | -0.4 | | 1.04E-06 | |
| Fibroblasts | Fibroblasts | PIM3 | -0.31 | | 1.08E-06 | |
| Fibroblasts | Fibroblasts | RAB30 | -0.27 | | 1.16E-06 | |
| Fibroblasts | Fibroblasts | NSD3 | -0.32 | | 1.21E-06 | |
| Fibroblasts | Fibroblasts | RAP1B | -0.33 | | 1.45E-06 | |
| Fibroblasts | Fibroblasts | PIK3IP1 | -0.34 | | 2.25E-06 | |
| Fibroblasts | Fibroblasts | ENO1 | -0.29 | | 2.39E-06 | |
| Fibroblasts | Fibroblasts | H2AFV | -0.31 | | 2.47E-06 | |
| Fibroblasts | Fibroblasts | HLA-F | -0.3 | | 2.77E-06 | |
| Fibroblasts | Fibroblasts | SF1 | -0.33 | | 3.37E-06 | |
| Fibroblasts | Fibroblasts | KMT2E | -0.29 | | 4.34E-06 | |
| Fibroblasts | Fibroblasts | GNLY | -1.41 | | 4.86E-06 | |
| Fibroblasts | Fibroblasts | UPP1 | -0.27 | | 5.45E-06 | |
| Fibroblasts | Fibroblasts | IVNS1ABP | -0.34 | | 6.29E-06 | |
| Fibroblasts | Fibroblasts | SLC25A5 | -0.25 | | 6.73E-06 | |
| Fibroblasts | Fibroblasts | CCDC12 | -0.26 | | 6.82E-06 | |
| Fibroblasts | Fibroblasts | PARP8 | -0.25 | | 7.08E-06 | |
| Fibroblasts | Fibroblasts | OSTF1 | -0.26 | | 8.31E-06 | |
| Fibroblasts | Fibroblasts | LYST | -0.33 | | 9.81E-06 | |
| Plasma | Plasma | MZB1 | 1.7 | | 0 | |
| Plasma | Plasma | DERL3 | 1.61 | | 0 | |
| Plasma | Plasma | FKBP11 | 1.3 | | 0 | |
| Plasma | Plasma | PRDX4 | 1.02 | | 0 | |
| Plasma | Plasma | DUSP5 | 1.01 | | 0 | |
| Plasma | Plasma | JSRP1 | 0.97 | | 0 | |
| Plasma | Plasma | TNFRSF17 | 0.89 | | 0 | |
| Plasma | Plasma | PRDM1 | 0.77 | | 0 | |
| Plasma | Plasma | RAB30 | 0.7 | | 0 | |
| Plasma | Plasma | IGLL1 | 0.63 | | 0 | |
| Plasma | Plasma | FCRL5 | 0.61 | | 0 | |
| Plasma | Plasma | SLAMF7 | 0.55 | | 0 | |
| Plasma | Plasma | CD38 | 0.54 | | 0 | |
| Plasma | Plasma | SPAG4 | 0.53 | | 0 | |
| Plasma | Plasma | CCR10 | 0.52 | | 0 | |
| Plasma | Plasma | AC104699.1 | 0.52 | | 0 | |
| Plasma | Plasma | CHPF | 0.51 | | 0 | |
| Plasma | Plasma | SDC1 | 0.46 | | 0 | |
| Plasma | Plasma | MEI1 | 0.45 | | 0 | |
| Plasma | Plasma | ZBP1 | 0.43 | | 0 | |
| Plasma | Plasma | POU2AF1 | 0.43 | | 0 | |
| Plasma | Plasma | ARSA | 0.42 | | 0 | |
| Plasma | Plasma | LINC02362 | 0.39 | | 0 | |
| Plasma | Plasma | LINC02384 | 0.34 | | 0 | |
| Plasma | Plasma | AC012236.1 | 0.25 | | 1.30E-300 | |
| Plasma | Plasma | TXNDC11 | 0.61 | | 1.35E-296 | |
| Plasma | Plasma | U62317.4 | 0.33 | | 1.63E-293 | |
| Plasma | Plasma | HBB | 0.81 | | 3.42E-293 | |
| Plasma | Plasma | TXNDC15 | 0.57 | | 7.99E-290 | |
| Plasma | Plasma | TRIB1 | 0.84 | | 3.89E-287 | |
| Plasma | Plasma | QPRT | 0.27 | | 1.76E-286 | |
| Plasma | Plasma | SEC11C | 1.17 | | 4.89E-281 | |
| Plasma | Plasma | PNOC | 0.29 | | 8.35E-279 | |
| Plasma | Plasma | IGLL5 | 3.96 | | 5.02E-274 | |
| Plasma | Plasma | CRELD2 | 0.7 | | 1.28E-270 | |
| Plasma | Plasma | LY96 | 0.49 | | 4.93E-262 | |
| Plasma | Plasma | PIM2 | 0.77 | | 2.04E-259 | |
| Plasma | Plasma | ITM2C | 1.02 | | 3.76E-255 | |
| Plasma | Plasma | IFNAR2 | 0.36 | | 3.04E-251 | |
| Plasma | Plasma | HBA2 | 0.27 | | 5.63E-245 | |
| Plasma | Plasma | PLPP5 | 0.53 | | 1.83E-242 | |
| Plasma | Plasma | SIL1 | 0.38 | | 3.85E-242 | |
| Plasma | Plasma | JCHAIN | 2.08 | | 2.04E-241 | |
| Plasma | Plasma | CD27 | 0.68 | | 5.94E-241 | |
| Plasma | Plasma | LMAN1 | 0.52 | | 4.75E-230 | |
| Plasma | Plasma | AL928742.1 | 0.39 | | 4.75E-230 | |
| Plasma | Plasma | DNAAF1 | 0.77 | | 1.19E-228 | |
| Plasma | Plasma | NUCB2 | 0.53 | | 1.40E-228 | |
| Plasma | Plasma | SEL1L | 0.29 | | 1.53E-227 | |
| Plasma | Plasma | DNAJB9 | 0.83 | | 2.60E-226 | |
| Plasma | Plasma | ERLEC1 | 0.48 | | 1.80E-224 | |
| Plasma | Plasma | SSR4 | 1.45 | | 8.99E-223 | |
| Plasma | Plasma | BEST1 | 0.27 | | 3.96E-222 | |
| Plasma | Plasma | TPST2 | 0.32 | | 5.38E-222 | |
| Plasma | Plasma | PDIA4 | 0.74 | | 2.50E-221 | |
| Plasma | Plasma | XBP1 | 1.02 | | 3.18E-221 | |
| Plasma | Plasma | EDEM2 | 0.3 | | 1.40E-219 | |
| Plasma | Plasma | CHST2 | 0.37 | | 5.18E-218 | |
| Plasma | Plasma | HM13 | 0.55 | | 7.09E-217 | |
| Plasma | Plasma | SSR3 | 0.84 | | 4.50E-216 | |
| Plasma | Plasma | SDF2L1 | 0.79 | | 2.77E-215 | |
| Plasma | Plasma | SRPRB | 0.34 | | 8.85E-214 | |
| Plasma | Plasma | ANKRD28 | 0.68 | | 2.05E-213 | |
| Plasma | Plasma | FKBP2 | 0.95 | | 8.27E-213 | |
| Plasma | Plasma | GNG7 | 0.36 | | 4.29E-212 | |
| Plasma | Plasma | HIST2H2AA4 | 1 | | 1.80E-211 | |
| Plasma | Plasma | B4GALT3 | 0.29 | | 1.97E-208 | |
| Plasma | Plasma | UBE2J1 | 0.68 | | 7.37E-203 | |
| Plasma | Plasma | KLF13 | 0.39 | | 4.48E-202 | |
| Plasma | Plasma | HERPUD1 | 1.22 | | 3.94E-200 | |
| Plasma | Plasma | ALG5 | 0.41 | | 1.68E-195 | |
| Plasma | Plasma | CHID1 | 0.32 | | 3.96E-190 | |
| Plasma | Plasma | LINC01480 | 0.39 | | 1.11E-189 | |
| Plasma | Plasma | TAPBPL | 0.26 | | 1.43E-187 | |
| Plasma | Plasma | SPCS3 | 0.66 | | 5.27E-185 | |
| Plasma | Plasma | H1FX | 0.68 | | 2.12E-182 | |
| Plasma | Plasma | TENT5C | 0.54 | | 9.29E-182 | |
| Plasma | Plasma | SEC61A1 | 0.35 | | 1.12E-181 | |
| Plasma | Plasma | TPD52 | 0.36 | | 4.11E-180 | |
| Plasma | Plasma | HIST1H2BC | 0.29 | | 2.74E-177 | |
| Plasma | Plasma | SEL1L3 | 0.28 | | 7.44E-177 | |
| Plasma | Plasma | Z93241.1 | 0.66 | | 3.91E-176 | |
| Plasma | Plasma | HSP90B1 | 1.03 | | 1.33E-174 | |
| Plasma | Plasma | HIST1H1C | 0.59 | | 1.39E-174 | |
| Plasma | Plasma | ICAM2 | 0.25 | | 8.58E-174 | |
| Plasma | Plasma | PELI1 | 0.49 | | 1.87E-172 | |
| Plasma | Plasma | HIST1H2BK | 0.68 | | 9.87E-171 | |
| Plasma | Plasma | KDELR1 | 0.43 | | 2.61E-170 | |
| Plasma | Plasma | MIR155HG | 0.51 | | 6.86E-169 | |
| Plasma | Plasma | CYTOR | 0.86 | | 4.77E-166 | |
| Plasma | Plasma | MANF | 0.76 | | 5.43E-164 | |
| Plasma | Plasma | TMEM205 | 0.33 | | 9.67E-161 | |
| Plasma | Plasma | LIPF | -0.5 | | 7.95E-160 | |
| Plasma | Plasma | SELENOS | 0.68 | | 1.18E-159 | |
| Plasma | Plasma | MT-CO1 | -1.24 | | 1.11E-158 | |
| Plasma | Plasma | SRM | 0.36 | | 5.81E-158 | |
| Plasma | Plasma | MT-CO3 | -1.22 | | 5.10E-154 | |
| Plasma | Plasma | GADD45A | 0.77 | | 4.87E-152 | |
| Plasma | Plasma | PTMA | -1.02 | | 4.59E-150 | |
| Plasma | Plasma | TOR3A | 0.32 | | 4.37E-149 | |
| Plasma | Plasma | COMMD3 | 0.27 | | 4.82E-148 | |
| Plasma | Plasma | GLRX | 0.43 | | 2.39E-146 | |
| Plasma | Plasma | SELENOM | 0.32 | | 2.87E-146 | |
| Plasma | Plasma | SPCS1 | 0.78 | | 5.90E-146 | |
| Plasma | Plasma | MT-ATP6 | -1.18 | | 9.16E-145 | |
| Plasma | Plasma | ACTB | -1.33 | | 3.38E-141 | |
| Plasma | Plasma | PGC | -0.79 | | 3.71E-140 | |
| Plasma | Plasma | MYDGF | 0.7 | | 2.60E-139 | |
| Plasma | Plasma | KRTCAP2 | 0.75 | | 9.66E-139 | |
| Plasma | Plasma | CD79A | 0.64 | | 3.34E-134 | |
| Plasma | Plasma | RPN2 | 0.51 | | 6.58E-134 | |
| Plasma | Plasma | TMEM208 | 0.35 | | 6.95E-134 | |
| Plasma | Plasma | ERN1 | 0.29 | | 1.08E-130 | |
| Plasma | Plasma | SPCS2 | 0.64 | | 1.17E-129 | |
| Plasma | Plasma | PDIA6 | 0.57 | | 1.79E-127 | |
| Plasma | Plasma | TMSB4X | -1.17 | | 7.05E-127 | |
| Plasma | Plasma | SPINK2 | 0.25 | | 8.08E-124 | |
| Plasma | Plasma | VOPP1 | 0.27 | | 3.86E-121 | |
| Plasma | Plasma | AP001160.1 | 0.29 | | 2.08E-120 | |
| Plasma | Plasma | LMAN2 | 0.54 | | 5.96E-120 | |
| Plasma | Plasma | TMEM59 | 0.63 | | 3.10E-118 | |
| Plasma | Plasma | DNAJB11 | 0.33 | | 2.07E-117 | |
| Plasma | Plasma | TMSB10 | -1.04 | | 1.06E-114 | |
| Plasma | Plasma | RPS27A | -0.71 | | 5.50E-114 | |
| Plasma | Plasma | CITED2 | 0.58 | | 1.33E-112 | |
| Plasma | Plasma | RPL31 | -0.81 | | 1.44E-112 | |
| Plasma | Plasma | RPS12 | -0.77 | | 2.72E-112 | |
| Plasma | Plasma | TRAM1 | 0.5 | | 4.10E-112 | |
| Plasma | Plasma | PABPC4 | 0.41 | | 9.74E-112 | |
| Plasma | Plasma | RPS24 | -0.68 | | 1.44E-111 | |
| Plasma | Plasma | LGALSL | 0.31 | | 1.05E-110 | |
| Plasma | Plasma | DDOST | 0.35 | | 1.08E-108 | |
| Plasma | Plasma | KDELR2 | 0.4 | | 4.26E-108 | |
| Plasma | Plasma | CFL1 | -0.95 | | 5.05E-106 | |
| Plasma | Plasma | RPL30 | -0.74 | | 1.42E-105 | |
| Plasma | Plasma | MT-CYB | -1.01 | | 9.97E-105 | |
| Plasma | Plasma | RPS20 | -0.68 | | 2.61E-104 | |
| Plasma | Plasma | RPL32 | -0.66 | | 3.04E-104 | |
| Plasma | Plasma | RPL28 | -0.65 | | 2.33E-103 | |
| Plasma | Plasma | RPL38 | -0.78 | | 3.13E-103 | |
| Plasma | Plasma | RPS27 | -0.85 | | 2.36E-102 | |
| Plasma | Plasma | RPS3 | -0.66 | | 4.48E-101 | |
| Plasma | Plasma | TMED9 | 0.44 | | 3.92E-99 | |
| Plasma | Plasma | RPL12 | -0.61 | | 4.99E-99 | |
| Plasma | Plasma | HLA-A | -0.9 | | 1.08E-97 | |
| Plasma | Plasma | TMED10 | 0.41 | | 7.66E-97 | |
| Plasma | Plasma | RPL39 | -0.68 | | 2.55E-96 | |
| Plasma | Plasma | RPLP2 | -0.73 | | 3.11E-96 | |
| Plasma | Plasma | RPL11 | -0.62 | | 9.77E-96 | |
| Plasma | Plasma | RPL34 | -0.66 | | 9.69E-95 | |
| Plasma | Plasma | RPL13A | -0.58 | | 3.69E-94 | |
| Plasma | Plasma | ERGIC3 | 0.41 | | 7.75E-94 | |
| Plasma | Plasma | H3F3A | -0.85 | | 2.40E-92 | |
| Plasma | Plasma | TMEM258 | 0.58 | | 6.58E-92 | |
| Plasma | Plasma | UBA52 | -0.61 | | 6.87E-92 | |
| Plasma | Plasma | THAP2 | 0.55 | | 9.25E-92 | |
| Plasma | Plasma | ATRAID | 0.29 | | 1.37E-91 | |
| Plasma | Plasma | RPS25 | -0.64 | | 2.60E-89 | |
| Plasma | Plasma | OTUD1 | 0.27 | | 2.62E-88 | |
| Plasma | Plasma | PHPT1 | 0.27 | | 2.85E-88 | |
| Plasma | Plasma | MT-ND4 | -0.91 | | 3.09E-87 | |
| Plasma | Plasma | RPL21 | -0.6 | | 4.40E-87 | |
| Plasma | Plasma | RPS23 | -0.65 | | 5.10E-87 | |
| Plasma | Plasma | RPL26 | -0.61 | | 1.75E-85 | |
| Plasma | Plasma | RPL13 | -0.53 | | 3.79E-85 | |
| Plasma | Plasma | RABAC1 | 0.48 | | 2.16E-84 | |
| Plasma | Plasma | ZNF165 | 0.34 | | 1.48E-83 | |
| Plasma | Plasma | PFN1 | -1.01 | | 6.78E-83 | |
| Plasma | Plasma | RPL9 | -0.6 | | 9.28E-83 | |
| Plasma | Plasma | RPL27 | -0.6 | | 8.37E-82 | |
| Plasma | Plasma | RPL7 | -0.55 | | 5.77E-81 | |
| Plasma | Plasma | RPS29 | -0.78 | | 2.01E-79 | |
| Plasma | Plasma | RPL37 | -0.64 | | 2.96E-79 | |
| Plasma | Plasma | FTH1 | -1.54 | | 4.69E-79 | |
| Plasma | Plasma | PGA3 | 0.33 | | 1.59E-77 | |
| Plasma | Plasma | RPL22 | -0.63 | | 5.26E-77 | |
| Plasma | Plasma | MT-CO2 | -0.89 | | 1.08E-76 | |
| Plasma | Plasma | RPS13 | -0.56 | | 1.60E-76 | |
| Plasma | Plasma | RPL23 | -0.62 | | 2.50E-75 | |
| Plasma | Plasma | CDKN1A | 0.36 | | 3.70E-75 | |
| Plasma | Plasma | RPS6 | -0.57 | | 7.33E-75 | |
| Plasma | Plasma | DDX5 | -0.9 | | 7.48E-75 | |
| Plasma | Plasma | AC103591.3 | 0.4 | | 1.98E-74 | |
| Plasma | Plasma | RPS3A | -0.61 | | 2.87E-74 | |
| Plasma | Plasma | BTG1 | -1.18 | | 4.82E-74 | |
| Plasma | Plasma | RPL19 | -0.53 | | 3.21E-72 | |
| Plasma | Plasma | PABPC1 | -0.81 | | 3.23E-72 | |
| Plasma | Plasma | MT-ND1 | -0.89 | | 4.77E-72 | |
| Plasma | Plasma | OSTC | 0.26 | | 5.55E-72 | |
| Plasma | Plasma | CTSH | 0.26 | | 7.57E-72 | |
| Plasma | Plasma | MT-ND3 | -0.86 | | 8.35E-72 | |
| Plasma | Plasma | RPL10A | -0.55 | | 4.89E-71 | |
| Plasma | Plasma | RPL41 | -0.42 | | 8.06E-71 | |
| Plasma | Plasma | SEC61B | 0.47 | | 1.68E-70 | |
| Plasma | Plasma | CYBA | 0.49 | | 9.46E-70 | |
| Plasma | Plasma | DDIT4 | 0.26 | | 9.51E-69 | |
| Plasma | Plasma | PSCA | -0.28 | | 1.06E-68 | |
| Plasma | Plasma | RPL35A | -0.47 | | 4.84E-68 | |
| Plasma | Plasma | RPS9 | -0.44 | | 8.98E-68 | |
| Plasma | Plasma | REEP5 | 0.25 | | 1.28E-66 | |
| Plasma | Plasma | RPS18 | -0.47 | | 1.79E-66 | |
| Plasma | Plasma | RPL14 | -0.51 | | 1.15E-65 | |
| Plasma | Plasma | RPS19 | -0.47 | | 4.21E-64 | |
| Plasma | Plasma | CALM2 | -0.86 | | 2.82E-63 | |
| Plasma | Plasma | P4HB | 0.3 | | 1.20E-62 | |
| Plasma | Plasma | RPL27A | -0.46 | | 3.13E-62 | |
| Plasma | Plasma | RPL23A | -0.49 | | 3.25E-62 | |
| Plasma | Plasma | RPS21 | -0.68 | | 3.40E-62 | |
| Plasma | Plasma | CUTA | 0.37 | | 4.68E-62 | |
| Plasma | Plasma | BTF3 | -0.6 | | 1.17E-61 | |
| Plasma | Plasma | RPS16 | -0.46 | | 1.45E-61 | |
| Plasma | Plasma | MT-ND2 | -0.77 | | 1.11E-60 | |
| Plasma | Plasma | SH3BGRL3 | -1 | | 1.41E-60 | |
| Plasma | Plasma | RPS15A | -0.48 | | 2.48E-60 | |
| Plasma | Plasma | ATF4 | 0.4 | | 1.51E-59 | |
| Plasma | Plasma | RPL36 | -0.49 | | 1.54E-59 | |
| Plasma | Plasma | EEF1A1 | -0.5 | | 1.26E-58 | |
| Plasma | Plasma | RPS2 | -0.42 | | 2.58E-58 | |
| Plasma | Plasma | RPS15 | -0.4 | | 5.74E-58 | |
| Plasma | Plasma | HNRNPA1 | -0.62 | | 7.30E-58 | |
| Plasma | Plasma | TPSAB1 | -1.61 | | 1.21E-57 | |
| Plasma | Plasma | RPS7 | -0.47 | | 1.55E-57 | |
| Plasma | Plasma | TMED2 | 0.29 | | 1.77E-57 | |
| Plasma | Plasma | ISCU | 0.31 | | 2.19E-57 | |
| Plasma | Plasma | MYL6 | -0.61 | | 2.43E-57 | |
| Plasma | Plasma | RPS26 | -0.72 | | 1.17E-55 | |
| Plasma | Plasma | DAD1 | 0.3 | | 2.46E-55 | |
| Plasma | Plasma | FAU | -0.41 | | 3.86E-55 | |
| Plasma | Plasma | RPL37A | -0.44 | | 5.73E-55 | |
| Plasma | Plasma | PPIA | -0.68 | | 1.48E-54 | |
| Plasma | Plasma | ISG20 | 0.39 | | 1.73E-53 | |
| Plasma | Plasma | RPSA | -0.6 | | 1.19E-52 | |
| Plasma | Plasma | RPL35 | -0.41 | | 2.04E-52 | |
| Plasma | Plasma | RPS14 | -0.4 | | 1.54E-51 | |
| Plasma | Plasma | PFDN5 | -0.51 | | 2.32E-51 | |
| Plasma | Plasma | MYL12B | -0.87 | | 1.47E-50 | |
| Plasma | Plasma | TOMM7 | -0.69 | | 1.84E-50 | |
| Plasma | Plasma | HMGB1 | -0.79 | | 7.77E-50 | |
| Plasma | Plasma | MALAT1 | -0.52 | | 8.94E-49 | |
| Plasma | Plasma | PPIB | 0.36 | | 1.78E-48 | |
| Plasma | Plasma | LAPTM5 | -1.45 | | 2.38E-47 | |
| Plasma | Plasma | GNLY | -1.36 | | 3.18E-47 | |
| Plasma | Plasma | CXCR4 | -1.71 | | 2.79E-46 | |
| Plasma | Plasma | YBX1 | -0.6 | | 1.45E-45 | |
| Plasma | Plasma | TIMP1 | -0.97 | | 1.49E-45 | |
| Plasma | Plasma | CALM1 | -0.72 | | 9.53E-45 | |
| Plasma | Plasma | SQSTM1 | 0.51 | | 3.69E-44 | |
| Plasma | Plasma | RPL24 | -0.38 | | 2.14E-43 | |
| Plasma | Plasma | COMMD6 | -0.78 | | 1.45E-42 | |
| Plasma | Plasma | SKP1 | -0.67 | | 1.76E-41 | |
| Plasma | Plasma | RPS10 | -0.57 | | 1.02E-39 | |
| Plasma | Plasma | B2M | -0.41 | | 1.06E-39 | |
| Plasma | Plasma | RPS28 | -0.35 | | 2.36E-39 | |
| Plasma | Plasma | S100A6 | -1.16 | | 2.70E-39 | |
| Plasma | Plasma | RPS8 | -0.41 | | 2.99E-38 | |
| Plasma | Plasma | RPS11 | -0.38 | | 6.92E-38 | |
| Plasma | Plasma | CD52 | -1.49 | | 9.26E-38 | |
| Plasma | Plasma | RGS1 | 0.26 | | 2.16E-37 | |
| Plasma | Plasma | NPM1 | -0.51 | | 1.09E-36 | |
| Plasma | Plasma | EIF4A1 | -0.59 | | 1.24E-36 | |
| Plasma | Plasma | HSPA5 | 0.48 | | 8.56E-36 | |
| Plasma | Plasma | NACA | -0.39 | | 1.08E-35 | |
| Plasma | Plasma | ATP5F1E | -0.46 | | 1.23E-35 | |
| Plasma | Plasma | CREM | -1.27 | | 1.90E-35 | |
| Plasma | Plasma | SERP1 | 0.32 | | 1.82E-34 | |
| Plasma | Plasma | RPL29 | -0.33 | | 2.26E-33 | |
| Plasma | Plasma | HLA-E | -0.52 | | 1.29E-32 | |
| Plasma | Plasma | MYL12A | -0.84 | | 1.52E-32 | |
| Plasma | Plasma | KLF6 | -0.74 | | 3.21E-32 | |
| Plasma | Plasma | RPL15 | -0.3 | | 5.65E-32 | |
| Plasma | Plasma | ARHGDIB | -0.97 | | 6.11E-32 | |
| Plasma | Plasma | REG1A | -0.52 | | 7.77E-32 | |
| Plasma | Plasma | ZFAS1 | -0.84 | | 8.07E-32 | |
| Plasma | Plasma | UQCRB | -0.43 | | 1.68E-31 | |
| Plasma | Plasma | HSPB1 | 0.36 | | 1.73E-31 | |
| Plasma | Plasma | SRP14 | -0.47 | | 2.95E-31 | |
| Plasma | Plasma | TMA7 | -0.48 | | 9.51E-31 | |
| Plasma | Plasma | C1QB | -0.68 | | 1.18E-30 | |
| Plasma | Plasma | REL | -1.02 | | 1.80E-30 | |
| Plasma | Plasma | HSP90AA1 | -0.45 | | 4.60E-30 | |
| Plasma | Plasma | SARAF | -0.83 | | 6.97E-30 | |
| Plasma | Plasma | COTL1 | -1.07 | | 1.66E-29 | |
| Plasma | Plasma | TNFRSF4 | -0.39 | | 1.74E-29 | |
| Plasma | Plasma | UBB | -0.38 | | 3.05E-29 | |
| Plasma | Plasma | ACTG1 | -0.61 | | 3.50E-29 | |
| Plasma | Plasma | COX4I1 | -0.4 | | 4.97E-29 | |
| Plasma | Plasma | RPL6 | -0.31 | | 1.58E-28 | |
| Plasma | Plasma | RPL18A | -0.31 | | 2.26E-27 | |
| Plasma | Plasma | TFF2 | -0.42 | | 3.16E-27 | |
| Plasma | Plasma | HNRNPK | -0.67 | | 5.68E-27 | |
| Plasma | Plasma | SUMO2 | -0.59 | | 8.65E-27 | |
| Plasma | Plasma | HSP90AB1 | -0.6 | | 8.18E-26 | |
| Plasma | Plasma | GAPDH | -0.54 | | 1.09E-25 | |
| Plasma | Plasma | GKN1 | -0.82 | | 1.49E-25 | |
| Plasma | Plasma | APOE | -1.05 | | 2.47E-25 | |
| Plasma | Plasma | NAP1L1 | -0.76 | | 3.72E-25 | |
| Plasma | Plasma | APOA1 | -0.36 | | 9.83E-25 | |
| Plasma | Plasma | LRRFIP1 | -0.84 | | 2.61E-24 | |
| Plasma | Plasma | LDHA | -0.91 | | 9.01E-24 | |
| Plasma | Plasma | ARPC2 | -0.65 | | 1.06E-23 | |
| Plasma | Plasma | RACK1 | -0.29 | | 4.41E-23 | |
| Plasma | Plasma | RPL5 | -0.34 | | 5.21E-23 | |
| Plasma | Plasma | HNRNPA2B1 | -0.6 | | 1.43E-22 | |
| Plasma | Plasma | ITM2B | -0.67 | | 1.64E-22 | |
| Plasma | Plasma | HLA-DPB1 | -1.68 | | 2.54E-22 | |
| Plasma | Plasma | EIF1 | -0.28 | | 3.03E-21 | |
| Plasma | Plasma | TFF1 | -0.88 | | 3.31E-21 | |
| Plasma | Plasma | SDCBP | -0.91 | | 5.85E-21 | |
| Plasma | Plasma | HSPA8 | -0.62 | | 1.22E-20 | |
| Plasma | Plasma | YWHAZ | -0.57 | | 2.78E-20 | |
| Plasma | Plasma | CXCL8 | -1.89 | | 4.16E-20 | |
| Plasma | Plasma | ARPC3 | -0.57 | | 5.24E-20 | |
| Plasma | Plasma | CHCHD2 | -0.34 | | 5.91E-20 | |
| Plasma | Plasma | FUS | -0.8 | | 1.28E-19 | |
| Plasma | Plasma | NDUFS5 | -0.62 | | 1.95E-19 | |
| Plasma | Plasma | CDC42 | -0.66 | | 2.18E-19 | |
| Plasma | Plasma | TPT1 | -0.25 | | 2.27E-19 | |
| Plasma | Plasma | S100A10 | -0.95 | | 2.82E-18 | |
| Plasma | Plasma | DCN | -0.81 | | 3.74E-18 | |
| Plasma | Plasma | MT-ND5 | -0.66 | | 4.17E-18 | |
| Plasma | Plasma | COX7C | -0.32 | | 7.01E-18 | |
| Plasma | Plasma | ICAM1 | -0.27 | | 9.00E-18 | |
| Plasma | Plasma | NOP53 | -0.43 | | 9.06E-18 | |
| Plasma | Plasma | TYMP | -0.28 | | 1.42E-17 | |
| Plasma | Plasma | ENO1 | -0.74 | | 2.56E-17 | |
| Plasma | Plasma | SNRPD2 | -0.53 | | 4.56E-17 | |
| Plasma | Plasma | CD37 | -1.04 | | 8.26E-17 | |
| Plasma | Plasma | PTPRC | -0.96 | | 1.04E-16 | |
| Plasma | Plasma | TUBA4A | -0.27 | | 5.07E-16 | |
| Plasma | Plasma | C1QA | -0.67 | | 7.51E-16 | |
| Plasma | Plasma | AC058791.1 | -0.29 | | 9.39E-16 | |
| Plasma | Plasma | DEK | -0.54 | | 1.07E-15 | |
| Plasma | Plasma | CCNI | -0.54 | | 1.08E-15 | |
| Plasma | Plasma | HLA-B | -0.28 | | 1.79E-15 | |
| Plasma | Plasma | SRSF2 | -0.59 | | 4.66E-15 | |
| Plasma | Plasma | CKLF | -0.25 | | 5.43E-15 | |
| Plasma | Plasma | C1R | -0.31 | | 8.50E-15 | |
| Plasma | Plasma | SRSF5 | -0.57 | | 1.70E-14 | |
| Plasma | Plasma | SRGN | -0.71 | | 1.74E-14 | |
| Plasma | Plasma | PPDPF | -0.61 | | 4.46E-14 | |
| Plasma | Plasma | GZMB | -1.09 | | 9.34E-14 | |
| Plasma | Plasma | HNRNPA0 | -0.6 | | 1.19E-13 | |
| Plasma | Plasma | NINJ1 | -0.34 | | 1.19E-13 | |
| Plasma | Plasma | SOD1 | -0.56 | | 1.23E-13 | |
| Plasma | Plasma | RPS4Y1 | -0.87 | | 1.27E-13 | |
| Plasma | Plasma | CIB1 | -0.66 | | 1.56E-13 | |
| Plasma | Plasma | PHGR1 | -0.71 | | 1.71E-13 | |
| Plasma | Plasma | SLC2A3 | -0.94 | | 3.63E-13 | |
| Plasma | Plasma | HLA-DRB1 | -1.61 | | 4.97E-13 | |
| Plasma | Plasma | TAGLN2 | -0.4 | | 1.07E-12 | |
| Plasma | Plasma | CLDND1 | -0.25 | | 1.24E-12 | |
| Plasma | Plasma | PNRC1 | -0.59 | | 1.89E-12 | |
| Plasma | Plasma | OAZ1 | -0.28 | | 2.80E-12 | |
| Plasma | Plasma | HLA-DRB5 | -1.28 | | 2.98E-12 | |
| Plasma | Plasma | UBE2D3 | -0.48 | | 3.16E-12 | |
| Plasma | Plasma | IL32 | -1.27 | | 4.46E-12 | |
| Plasma | Plasma | PGK1 | -0.71 | | 5.33E-12 | |
| Plasma | Plasma | FXYD5 | -0.64 | | 6.69E-12 | |
| Plasma | Plasma | SNRPB | -0.57 | | 7.04E-12 | |
| Plasma | Plasma | SOCS3 | -0.52 | | 7.37E-12 | |
| Plasma | Plasma | ALDOA | -0.43 | | 8.20E-12 | |
| Plasma | Plasma | RPL4 | -0.25 | | 8.66E-12 | |
| Plasma | Plasma | CORO1A | -0.87 | | 1.61E-11 | |
| Plasma | Plasma | TPM3 | -0.66 | | 1.62E-11 | |
| Plasma | Plasma | TGFB1 | -0.25 | | 1.92E-11 | |
| Plasma | Plasma | SON | -0.61 | | 7.75E-11 | |
| Plasma | Plasma | ARPC1B | -0.74 | | 8.49E-11 | |
| Plasma | Plasma | ARL6IP1 | -0.67 | | 8.74E-11 | |
| Plasma | Plasma | ANXA5 | -0.42 | | 9.94E-11 | |
| Plasma | Plasma | TPI1 | -0.61 | | 1.02E-10 | |
| Plasma | Plasma | OCIAD2 | -0.27 | | 1.32E-10 | |
| Plasma | Plasma | EML4 | -0.68 | | 1.54E-10 | |
| Plasma | Plasma | PDLIM1 | -0.3 | | 4.07E-10 | |
| Plasma | Plasma | AGR2 | -0.61 | | 4.31E-10 | |
| Plasma | Plasma | CTSS | -0.3 | | 4.31E-10 | |
| Plasma | Plasma | LUM | -0.62 | | 4.57E-10 | |
| Plasma | Plasma | HLA-DQA2 | -0.36 | | 5.40E-10 | |
| Plasma | Plasma | SAP18 | -0.47 | | 5.45E-10 | |
| Plasma | Plasma | IFITM2 | -0.93 | | 5.52E-10 | |
| Plasma | Plasma | TGIF1 | -0.54 | | 5.52E-10 | |
| Plasma | Plasma | SLC25A3 | -0.44 | | 6.30E-10 | |
| Plasma | Plasma | ATP1B3 | -0.93 | | 6.86E-10 | |
| Plasma | Plasma | SRSF7 | -0.66 | | 7.20E-10 | |
| Plasma | Plasma | RBM39 | -0.49 | | 8.72E-10 | |
| Plasma | Plasma | COX6C | -0.43 | | 9.59E-10 | |
| Plasma | Plasma | RPL36A | -0.38 | | 1.02E-09 | |
| Plasma | Plasma | DAZAP2 | -0.62 | | 1.04E-09 | |
| Plasma | Plasma | RHOA | -0.49 | | 1.50E-09 | |
| Plasma | Plasma | NR4A2 | -0.98 | | 1.65E-09 | |
| Plasma | Plasma | JUND | -0.57 | | 1.90E-09 | |
| Plasma | Plasma | CTSD | -0.58 | | 2.04E-09 | |
| Plasma | Plasma | C1QC | -0.55 | | 2.65E-09 | |
| Plasma | Plasma | S100A4 | -1.36 | | 4.46E-09 | |
| Plasma | Plasma | CCDC85B | -0.31 | | 4.93E-09 | |
| Plasma | Plasma | 1-Sep | -0.28 | | 5.43E-09 | |
| Plasma | Plasma | S100A11 | -0.88 | | 5.95E-09 | |
| Plasma | Plasma | PSMA7 | -0.53 | | 6.67E-09 | |
| Plasma | Plasma | MGST3 | -0.27 | | 7.76E-09 | |
| Plasma | Plasma | RAN | -0.5 | | 7.92E-09 | |
| Plasma | Plasma | CD3E | -0.93 | | 7.94E-09 | |
| Plasma | Plasma | TUBA1B | -0.67 | | 8.19E-09 | |
| Plasma | Plasma | CTSB | -0.7 | | 8.98E-09 | |
| Plasma | Plasma | TRIR | -0.46 | | 9.90E-09 | |
| Plasma | Plasma | YWHAB | -0.58 | | 1.13E-08 | |
| Plasma | Plasma | TUBA1A | -0.91 | | 1.15E-08 | |
| Plasma | Plasma | ANXA1 | -1.38 | | 3.62E-08 | |
| Plasma | Plasma | PPP1CB | -0.76 | | 3.90E-08 | |
| Plasma | Plasma | HLA-DPA1 | -1.46 | | 4.49E-08 | |
| Plasma | Plasma | FOXP1 | -0.58 | | 4.64E-08 | |
| Plasma | Plasma | G0S2 | -0.99 | | 5.87E-08 | |
| Plasma | Plasma | CAPZB | -0.6 | | 6.80E-08 | |
| Plasma | Plasma | EGR1 | -0.59 | | 6.87E-08 | |
| Plasma | Plasma | CHMP1B | -0.82 | | 8.29E-08 | |
| Plasma | Plasma | APOC1 | -0.55 | | 1.09E-07 | |
| Plasma | Plasma | RAP1A | -0.26 | | 1.17E-07 | |
| Plasma | Plasma | MYH9 | -0.5 | | 1.88E-07 | |
| Plasma | Plasma | SLC7A5 | -0.35 | | 1.88E-07 | |
| Plasma | Plasma | ID3 | -0.33 | | 1.90E-07 | |
| Plasma | Plasma | KRT8 | -0.82 | | 2.40E-07 | |
| Plasma | Plasma | ARHGAP15 | -0.27 | | 2.57E-07 | |
| Plasma | Plasma | LTB | -1.02 | | 2.61E-07 | |
| Plasma | Plasma | APP | -0.38 | | 3.41E-07 | |
| Plasma | Plasma | HLA-DMB | -0.55 | | 3.50E-07 | |
| Plasma | Plasma | CD47 | -0.25 | | 3.65E-07 | |
| Plasma | Plasma | NR1H2 | -0.27 | | 3.70E-07 | |
| Plasma | Plasma | MUC5AC | -0.52 | | 3.78E-07 | |
| Plasma | Plasma | MT2A | -0.65 | | 3.91E-07 | |
| Plasma | Plasma | PMAIP1 | -0.29 | | 4.73E-07 | |
| Plasma | Plasma | RASGEF1B | -0.26 | | 4.90E-07 | |
| Plasma | Plasma | HNRNPDL | -0.4 | | 5.33E-07 | |
| Plasma | Plasma | ALG13 | -0.32 | | 5.75E-07 | |
| Plasma | Plasma | SNRPG | -0.53 | | 5.92E-07 | |
| Plasma | Plasma | MAPRE1 | -0.26 | | 6.27E-07 | |
| Plasma | Plasma | ARID4B | -0.59 | | 6.54E-07 | |
| Plasma | Plasma | FXYD3 | -0.42 | | 8.43E-07 | |
| Plasma | Plasma | INSIG1 | -0.25 | | 9.00E-07 | |
| Plasma | Plasma | HINT1 | -0.27 | | 9.14E-07 | |
| Plasma | Plasma | TNRC6B | -0.27 | | 9.38E-07 | |
| Plasma | Plasma | HLA-DRA | -2.07 | | 9.43E-07 | |
| Plasma | Plasma | ATP6V0E1 | -0.43 | | 9.66E-07 | |
| Plasma | Plasma | RARRES2 | -0.29 | | 9.80E-07 | |
| Plasma | Plasma | DYNLL1 | -0.51 | | 1.11E-06 | |
| Plasma | Plasma | KLF4 | -0.62 | | 1.17E-06 | |
| Plasma | Plasma | GSN | -0.89 | | 1.25E-06 | |
| Plasma | Plasma | ATP6V0D1 | -0.26 | | 1.33E-06 | |
| Plasma | Plasma | CMTM6 | -0.61 | | 1.40E-06 | |
| Plasma | Plasma | EIF3E | -0.38 | | 1.47E-06 | |
| Plasma | Plasma | SNX9 | -0.47 | | 1.83E-06 | |
| Plasma | Plasma | RBM8A | -0.5 | | 1.98E-06 | |
| Plasma | Plasma | CALM3 | -0.27 | | 2.12E-06 | |
| Plasma | Plasma | SRI | -0.26 | | 2.15E-06 | |
| Plasma | Plasma | TERF2IP | -0.32 | | 2.26E-06 | |
| Plasma | Plasma | MT1G | -0.54 | | 2.39E-06 | |
| Plasma | Plasma | HES1 | -0.76 | | 2.83E-06 | |
| Plasma | Plasma | RBM3 | -0.35 | | 3.18E-06 | |
| Plasma | Plasma | BATF | -0.32 | | 3.19E-06 | |
| Plasma | Plasma | HCST | -0.79 | | 3.70E-06 | |
| Plasma | Plasma | DNAJB6 | -0.59 | | 4.88E-06 | |
| Plasma | Plasma | EIF4G2 | -0.53 | | 4.97E-06 | |
| Plasma | Plasma | EVL | -0.63 | | 6.21E-06 | |
| Plasma | Plasma | IFI27 | -0.75 | | 6.38E-06 | |
| Plasma | Plasma | COX7B | -0.42 | | 6.66E-06 | |
| Plasma | Plasma | CAST | -0.29 | | 7.22E-06 | |
| Plasma | Plasma | PTGER4 | -0.54 | | 7.28E-06 | |
| Plasma | Plasma | DDX18 | -0.25 | | 8.06E-06 | |
| Plasma | Plasma | ARL4C | -0.53 | | 9.03E-06 | |
| Plasma | Plasma | LYZ | -1.33 | | 9.33E-06 | |
| Plasma | Plasma | CCDC59 | -0.26 | | 9.89E-06 | |
| CD8T | CD8T | STMN1 | 2.21 | | 0 | |
| CD8T | CD8T | PCLAF | 1.86 | | 0 | |
| CD8T | CD8T | TYMS | 1.54 | | 0 | |
| CD8T | CD8T | TK1 | 1.27 | | 0 | |
| CD8T | CD8T | UBE2C | 1.24 | | 0 | |
| CD8T | CD8T | PTTG1 | 1.23 | | 0 | |
| CD8T | CD8T | RRM2 | 1.2 | | 0 | |
| CD8T | CD8T | MCM7 | 1.01 | | 0 | |
| CD8T | CD8T | BIRC5 | 0.98 | | 0 | |
| CD8T | CD8T | ZWINT | 0.91 | | 0 | |
| CD8T | CD8T | NUSAP1 | 0.87 | | 0 | |
| CD8T | CD8T | MAD2L1 | 0.81 | | 0 | |
| CD8T | CD8T | CDK1 | 0.78 | | 0 | |
| CD8T | CD8T | CDKN3 | 0.77 | | 0 | |
| CD8T | CD8T | FEN1 | 0.75 | | 0 | |
| CD8T | CD8T | ASF1B | 0.74 | | 0 | |
| CD8T | CD8T | MKI67 | 0.74 | | 0 | |
| CD8T | CD8T | MCM3 | 0.71 | | 0 | |
| CD8T | CD8T | CDT1 | 0.71 | | 0 | |
| CD8T | CD8T | CENPW | 0.7 | | 0 | |
| CD8T | CD8T | DHFR | 0.69 | | 0 | |
| CD8T | CD8T | AURKB | 0.69 | | 0 | |
| CD8T | CD8T | PHF19 | 0.64 | | 0 | |
| CD8T | CD8T | GINS2 | 0.64 | | 0 | |
| CD8T | CD8T | CENPH | 0.64 | | 0 | |
| CD8T | CD8T | TOP2A | 0.63 | | 0 | |
| CD8T | CD8T | CCNB2 | 0.61 | | 0 | |
| CD8T | CD8T | CLSPN | 0.61 | | 0 | |
| CD8T | CD8T | CENPN | 0.6 | | 0 | |
| CD8T | CD8T | UBE2T | 0.57 | | 0 | |
| CD8T | CD8T | MYBL2 | 0.56 | | 0 | |
| CD8T | CD8T | SMC2 | 0.55 | | 0 | |
| CD8T | CD8T | CDC20 | 0.54 | | 0 | |
| CD8T | CD8T | CCNA2 | 0.53 | | 0 | |
| CD8T | CD8T | CENPF | 0.53 | | 0 | |
| CD8T | CD8T | RNASEH2A | 0.52 | | 0 | |
| CD8T | CD8T | CENPU | 0.5 | | 0 | |
| CD8T | CD8T | TCF19 | 0.46 | | 0 | |
| CD8T | CD8T | NUF2 | 0.45 | | 0 | |
| CD8T | CD8T | KIFC1 | 0.44 | | 0 | |
| CD8T | CD8T | CDCA5 | 0.41 | | 0 | |
| CD8T | CD8T | CENPK | 0.41 | | 0 | |
| CD8T | CD8T | RAD51AP1 | 0.39 | | 0 | |
| CD8T | CD8T | GTSE1 | 0.39 | | 0 | |
| CD8T | CD8T | PLK1 | 0.39 | | 0 | |
| CD8T | CD8T | SPC25 | 0.39 | | 0 | |
| CD8T | CD8T | RFC3 | 0.38 | | 0 | |
| CD8T | CD8T | TROAP | 0.38 | | 0 | |
| CD8T | CD8T | CDC45 | 0.37 | | 0 | |
| CD8T | CD8T | MND1 | 0.35 | | 0 | |
| CD8T | CD8T | PKMYT1 | 0.35 | | 0 | |
| CD8T | CD8T | CDCA3 | 0.35 | | 0 | |
| CD8T | CD8T | TPX2 | 0.35 | | 0 | |
| CD8T | CD8T | E2F1 | 0.34 | | 0 | |
| CD8T | CD8T | FANCI | 0.34 | | 0 | |
| CD8T | CD8T | WDR76 | 0.33 | | 0 | |
| CD8T | CD8T | CENPE | 0.33 | | 0 | |
| CD8T | CD8T | CHEK1 | 0.33 | | 0 | |
| CD8T | CD8T | MCM2 | 0.33 | | 0 | |
| CD8T | CD8T | CENPA | 0.32 | | 0 | |
| CD8T | CD8T | RAD51 | 0.31 | | 0 | |
| CD8T | CD8T | SHCBP1 | 0.31 | | 0 | |
| CD8T | CD8T | SGO1 | 0.31 | | 0 | |
| CD8T | CD8T | NCAPH | 0.3 | | 0 | |
| CD8T | CD8T | CKAP2L | 0.29 | | 0 | |
| CD8T | CD8T | ORC6 | 0.29 | | 0 | |
| CD8T | CD8T | MELK | 0.27 | | 0 | |
| CD8T | CD8T | ASPM | 0.27 | | 0 | |
| CD8T | CD8T | KIF2C | 0.26 | | 0 | |
| CD8T | CD8T | NDC80 | 0.25 | | 0 | |
| CD8T | CD8T | DTYMK | 0.68 | | 1.56E-289 | |
| CD8T | CD8T | LIG1 | 0.4 | | 7.70E-278 | |
| CD8T | CD8T | RFC4 | 0.39 | | 3.71E-275 | |
| CD8T | CD8T | CKS1B | 1.23 | | 6.25E-274 | |
| CD8T | CD8T | CDC6 | 0.26 | | 4.77E-273 | |
| CD8T | CD8T | PRC1 | 0.3 | | 2.77E-267 | |
| CD8T | CD8T | NUDT1 | 0.83 | | 6.24E-267 | |
| CD8T | CD8T | HELLS | 0.47 | | 6.24E-254 | |
| CD8T | CD8T | CDCA7 | 0.43 | | 9.93E-253 | |
| CD8T | CD8T | TMEM106C | 0.74 | | 1.33E-251 | |
| CD8T | CD8T | PCNA | 1.42 | | 2.16E-250 | |
| CD8T | CD8T | HIRIP3 | 0.43 | | 3.80E-249 | |
| CD8T | CD8T | MCM4 | 0.39 | | 1.64E-248 | |
| CD8T | CD8T | GMNN | 0.55 | | 4.64E-244 | |
| CD8T | CD8T | ATAD2 | 0.3 | | 7.57E-243 | |
| CD8T | CD8T | MCM5 | 0.76 | | 9.77E-234 | |
| CD8T | CD8T | DUT | 1.59 | | 1.18E-232 | |
| CD8T | CD8T | SKA2 | 0.68 | | 8.01E-232 | |
| CD8T | CD8T | RPL39L | 0.47 | | 1.07E-230 | |
| CD8T | CD8T | SAE1 | 0.54 | | 5.04E-227 | |
| CD8T | CD8T | CHAF1A | 0.32 | | 6.58E-223 | |
| CD8T | CD8T | TUBB | 2.06 | | 5.09E-222 | |
| CD8T | CD8T | CENPM | 0.74 | | 2.18E-218 | |
| CD8T | CD8T | RFC2 | 0.44 | | 4.04E-218 | |
| CD8T | CD8T | SAC3D1 | 0.39 | | 1.96E-217 | |
| CD8T | CD8T | DNMT1 | 0.68 | | 9.04E-217 | |
| CD8T | CD8T | CCNB1 | 0.49 | | 8.13E-215 | |
| CD8T | CD8T | HMGB3 | 0.54 | | 4.73E-210 | |
| CD8T | CD8T | VRK1 | 0.37 | | 1.92E-194 | |
| CD8T | CD8T | ACOT7 | 0.52 | | 1.46E-193 | |
| CD8T | CD8T | RFC5 | 0.27 | | 6.56E-193 | |
| CD8T | CD8T | TMPO | 0.54 | | 1.30E-192 | |
| CD8T | CD8T | LRR1 | 0.3 | | 3.40E-192 | |
| CD8T | CD8T | EZH2 | 0.44 | | 5.54E-192 | |
| CD8T | CD8T | GAPDH | 1.27 | | 7.54E-185 | |
| CD8T | CD8T | POLR3K | 0.46 | | 5.86E-184 | |
| CD8T | CD8T | CBX5 | 0.43 | | 1.12E-183 | |
| CD8T | CD8T | HAUS1 | 0.45 | | 1.18E-183 | |
| CD8T | CD8T | HMGB2 | 1.91 | | 5.49E-183 | |
| CD8T | CD8T | RRM1 | 0.47 | | 1.17E-181 | |
| CD8T | CD8T | HMGN2 | 1.7 | | 3.38E-180 | |
| CD8T | CD8T | TACC3 | 0.44 | | 2.59E-178 | |
| CD8T | CD8T | ACAT2 | 0.44 | | 3.29E-172 | |
| CD8T | CD8T | TPGS2 | 0.51 | | 1.32E-171 | |
| CD8T | CD8T | LMNB1 | 0.36 | | 3.26E-170 | |
| CD8T | CD8T | RANBP1 | 1.23 | | 1.38E-167 | |
| CD8T | CD8T | PFN1 | 1.33 | | 7.99E-166 | |
| CD8T | CD8T | DNAJC9 | 0.77 | | 1.05E-165 | |
| CD8T | CD8T | TUBG1 | 0.26 | | 7.05E-163 | |
| CD8T | CD8T | BCL2L12 | 0.38 | | 4.77E-161 | |
| CD8T | CD8T | KIF22 | 0.54 | | 8.77E-161 | |
| CD8T | CD8T | ANP32E | 0.77 | | 1.73E-160 | |
| CD8T | CD8T | AL441992.1 | 0.27 | | 6.87E-160 | |
| CD8T | CD8T | GGH | 0.43 | | 9.52E-159 | |
| CD8T | CD8T | SMC4 | 0.69 | | 2.30E-157 | |
| CD8T | CD8T | MCM6 | 0.41 | | 2.89E-157 | |
| CD8T | CD8T | CCDC34 | 0.32 | | 4.43E-157 | |
| CD8T | CD8T | CDCA4 | 0.34 | | 6.56E-157 | |
| CD8T | CD8T | RAD51C | 0.35 | | 1.79E-155 | |
| CD8T | CD8T | CCDC167 | 0.71 | | 2.19E-154 | |
| CD8T | CD8T | CDKN2A | 0.42 | | 7.37E-154 | |
| CD8T | CD8T | SNRNP25 | 0.62 | | 1.41E-153 | |
| CD8T | CD8T | NABP2 | 0.47 | | 2.13E-152 | |
| CD8T | CD8T | TFDP1 | 0.58 | | 1.79E-150 | |
| CD8T | CD8T | USP1 | 0.49 | | 1.63E-146 | |
| CD8T | CD8T | ACTB | 1.26 | | 3.02E-143 | |
| CD8T | CD8T | HMGB1 | 1.29 | | 4.96E-143 | |
| CD8T | CD8T | H2AFZ | 1.34 | | 8.81E-143 | |
| CD8T | CD8T | RPA3 | 0.95 | | 6.45E-142 | |
| CD8T | CD8T | SSRP1 | 0.55 | | 2.01E-141 | |
| CD8T | CD8T | C12orf75 | 0.84 | | 1.23E-140 | |
| CD8T | CD8T | PKM | 1.19 | | 1.28E-139 | |
| CD8T | CD8T | KIF20B | 0.3 | | 2.67E-139 | |
| CD8T | CD8T | PPIA | 0.9 | | 2.92E-139 | |
| CD8T | CD8T | DEK | 0.98 | | 1.39E-138 | |
| CD8T | CD8T | ANP32B | 1.08 | | 1.52E-138 | |
| CD8T | CD8T | CFL1 | 0.98 | | 2.59E-138 | |
| CD8T | CD8T | FANCA | 0.27 | | 4.19E-138 | |
| CD8T | CD8T | ACTG1 | 1.18 | | 1.38E-135 | |
| CD8T | CD8T | PAFAH1B3 | 0.48 | | 2.99E-135 | |
| CD8T | CD8T | RAN | 1.02 | | 1.32E-133 | |
| CD8T | CD8T | PRDX3 | 0.65 | | 3.66E-133 | |
| CD8T | CD8T | YEATS4 | 0.39 | | 1.62E-132 | |
| CD8T | CD8T | CORO1A | 1.28 | | 1.36E-131 | |
| CD8T | CD8T | H2AFV | 1.09 | | 2.82E-130 | |
| CD8T | CD8T | PMVK | 0.67 | | 4.62E-130 | |
| CD8T | CD8T | HMGN1 | 1.12 | | 1.05E-129 | |
| CD8T | CD8T | NCAPH2 | 0.41 | | 8.34E-129 | |
| CD8T | CD8T | PSMD14 | 0.55 | | 3.82E-127 | |
| CD8T | CD8T | ITGB3BP | 0.3 | | 1.69E-126 | |
| CD8T | CD8T | PARP1 | 0.72 | | 2.82E-126 | |
| CD8T | CD8T | MTHFD1 | 0.3 | | 3.74E-126 | |
| CD8T | CD8T | ARPC1B | 1.09 | | 9.22E-126 | |
| CD8T | CD8T | ENO1 | 1.15 | | 1.31E-125 | |
| CD8T | CD8T | COMMD8 | 0.48 | | 1.37E-125 | |
| CD8T | CD8T | TUBA1B | 1.74 | | 2.44E-125 | |
| CD8T | CD8T | WDR34 | 0.43 | | 3.13E-125 | |
| CD8T | CD8T | CALM3 | 0.98 | | 1.85E-124 | |
| CD8T | CD8T | GGCT | 0.51 | | 1.87E-123 | |
| CD8T | CD8T | COMMD4 | 0.48 | | 5.78E-123 | |
| CD8T | CD8T | MRPL51 | 0.86 | | 2.37E-122 | |
| CD8T | CD8T | DDB2 | 0.27 | | 2.63E-122 | |
| CD8T | CD8T | ATP5MC3 | 0.91 | | 6.95E-122 | |
| CD8T | CD8T | ARPC5 | 0.93 | | 8.01E-122 | |
| CD8T | CD8T | MRPL37 | 0.45 | | 1.22E-121 | |
| CD8T | CD8T | IDH2 | 0.89 | | 1.33E-121 | |
| CD8T | CD8T | TADA3 | 0.47 | | 3.46E-121 | |
| CD8T | CD8T | HPRT1 | 0.57 | | 1.29E-120 | |
| CD8T | CD8T | CDC25B | 0.34 | | 5.07E-120 | |
| CD8T | CD8T | RAC2 | 1 | | 2.61E-119 | |
| CD8T | CD8T | SNRNP40 | 0.41 | | 8.83E-119 | |
| CD8T | CD8T | POLD2 | 0.46 | | 7.07E-118 | |
| CD8T | CD8T | RPA1 | 0.33 | | 4.47E-117 | |
| CD8T | CD8T | NUDT21 | 0.6 | | 4.51E-117 | |
| CD8T | CD8T | PPP1CA | 0.87 | | 4.63E-117 | |
| CD8T | CD8T | SLC25A5 | 0.96 | | 2.56E-116 | |
| CD8T | CD8T | FKBP3 | 0.56 | | 3.26E-116 | |
| CD8T | CD8T | RBBP7 | 0.65 | | 5.18E-116 | |
| CD8T | CD8T | LSM4 | 0.76 | | 6.39E-116 | |
| CD8T | CD8T | CARHSP1 | 0.75 | | 4.99E-115 | |
| CD8T | CD8T | NUDT5 | 0.56 | | 5.45E-115 | |
| CD8T | CD8T | H2AFY | 0.8 | | 2.22E-114 | |
| CD8T | CD8T | BATF3 | 0.41 | | 1.18E-113 | |
| CD8T | CD8T | MALAT1 | -1.19 | | 2.08E-113 | |
| CD8T | CD8T | HSPB11 | 0.56 | | 3.29E-113 | |
| CD8T | CD8T | MZT1 | 0.39 | | 1.34E-112 | |
| CD8T | CD8T | MAD2L2 | 0.42 | | 1.96E-112 | |
| CD8T | CD8T | PXMP2 | 0.31 | | 7.63E-112 | |
| CD8T | CD8T | NME1 | 0.86 | | 8.20E-111 | |
| CD8T | CD8T | THOC3 | 0.34 | | 1.06E-110 | |
| CD8T | CD8T | TPI1 | 0.92 | | 2.08E-110 | |
| CD8T | CD8T | POP7 | 0.37 | | 2.56E-110 | |
| CD8T | CD8T | UNG | 0.28 | | 3.94E-110 | |
| CD8T | CD8T | SNRPD1 | 0.81 | | 1.31E-109 | |
| CD8T | CD8T | PGAM1 | 0.9 | | 1.55E-109 | |
| CD8T | CD8T | LSM2 | 0.62 | | 5.35E-109 | |
| CD8T | CD8T | DHRS4 | 0.27 | | 6.40E-109 | |
| CD8T | CD8T | MTCH2 | 0.48 | | 7.29E-109 | |
| CD8T | CD8T | VBP1 | 0.44 | | 1.38E-108 | |
| CD8T | CD8T | ANAPC15 | 0.44 | | 1.55E-108 | |
| CD8T | CD8T | ANP32A | 0.59 | | 2.01E-108 | |
| CD8T | CD8T | ACTL6A | 0.32 | | 4.63E-108 | |
| CD8T | CD8T | CBX3 | 0.76 | | 1.13E-107 | |
| CD8T | CD8T | CSK | 0.33 | | 5.09E-107 | |
| CD8T | CD8T | RNASEH2B | 0.45 | | 1.15E-106 | |
| CD8T | CD8T | DDX39A | 0.7 | | 3.58E-106 | |
| CD8T | CD8T | ITGAE | 0.51 | | 1.31E-105 | |
| CD8T | CD8T | BANF1 | 0.73 | | 1.32E-105 | |
| CD8T | CD8T | COX8A | 0.84 | | 1.80E-105 | |
| CD8T | CD8T | SMC1A | 0.28 | | 3.65E-105 | |
| CD8T | CD8T | SMC3 | 0.46 | | 7.56E-105 | |
| CD8T | CD8T | DCTPP1 | 0.54 | | 8.19E-105 | |
| CD8T | CD8T | SMS | 0.6 | | 1.07E-104 | |
| CD8T | CD8T | BATF | 0.81 | | 3.27E-104 | |
| CD8T | CD8T | HADH | 0.36 | | 5.50E-104 | |
| CD8T | CD8T | PTPN18 | 0.31 | | 6.53E-104 | |
| CD8T | CD8T | CENPX | 0.61 | | 1.68E-103 | |
| CD8T | CD8T | PSIP1 | 0.48 | | 1.80E-103 | |
| CD8T | CD8T | PA2G4 | 0.72 | | 3.79E-103 | |
| CD8T | CD8T | FIBP | 0.53 | | 5.25E-103 | |
| CD8T | CD8T | ITGB1BP1 | 0.56 | | 5.63E-103 | |
| CD8T | CD8T | PAICS | 0.42 | | 3.83E-102 | |
| CD8T | CD8T | PSMB2 | 0.76 | | 6.53E-102 | |
| CD8T | CD8T | DCPS | 0.34 | | 2.06E-101 | |
| CD8T | CD8T | NUCKS1 | 0.75 | | 4.30E-101 | |
| CD8T | CD8T | SNRPA1 | 0.53 | | 5.47E-101 | |
| CD8T | CD8T | OXCT1 | 0.26 | | 2.02E-100 | |
| CD8T | CD8T | HMGA1 | 1.02 | | 2.08E-100 | |
| CD8T | CD8T | CHCHD2 | 0.7 | | 2.29E-100 | |
| CD8T | CD8T | CACYBP | 0.73 | | 3.98E-100 | |
| CD8T | CD8T | MRPL13 | 0.47 | | 6.60E-100 | |
| CD8T | CD8T | LDHB | 0.9 | | 1.20E-99 | |
| CD8T | CD8T | PIN1 | 0.5 | | 1.53E-99 | |
| CD8T | CD8T | ACYP1 | 0.28 | | 1.62E-99 | |
| CD8T | CD8T | CTLA4 | 0.45 | | 1.93E-99 | |
| CD8T | CD8T | PPIH | 0.36 | | 3.94E-99 | |
| CD8T | CD8T | SNRPA | 0.42 | | 2.56E-98 | |
| CD8T | CD8T | COPS3 | 0.42 | | 3.76E-98 | |
| CD8T | CD8T | ARPC4 | 0.69 | | 4.13E-98 | |
| CD8T | CD8T | COTL1 | 0.99 | | 4.35E-98 | |
| CD8T | CD8T | CKS2 | 0.72 | | 5.67E-98 | |
| CD8T | CD8T | IFI27L1 | 0.34 | | 1.84E-97 | |
| CD8T | CD8T | SNRPE | 0.8 | | 8.31E-97 | |
| CD8T | CD8T | MAP4K1 | 0.35 | | 1.50E-96 | |
| CD8T | CD8T | PSMA5 | 0.67 | | 1.63E-96 | |
| CD8T | CD8T | YBX1 | 0.73 | | 2.48E-96 | |
| CD8T | CD8T | SASH3 | 0.28 | | 5.26E-96 | |
| CD8T | CD8T | COX5A | 0.81 | | 1.22E-95 | |
| CD8T | CD8T | RCC1 | 0.29 | | 1.95E-95 | |
| CD8T | CD8T | DCK | 0.41 | | 2.40E-95 | |
| CD8T | CD8T | ADSL | 0.33 | | 9.50E-95 | |
| CD8T | CD8T | TALDO1 | 0.64 | | 2.07E-94 | |
| CD8T | CD8T | MZT2B | 0.76 | | 3.70E-94 | |
| CD8T | CD8T | CBX1 | 0.25 | | 4.13E-94 | |
| CD8T | CD8T | ARPC2 | 0.83 | | 4.58E-94 | |
| CD8T | CD8T | AC017002.3 | 0.39 | | 8.02E-94 | |
| CD8T | CD8T | ACAA2 | 0.39 | | 1.24E-93 | |
| CD8T | CD8T | WDR1 | 0.65 | | 2.25E-93 | |
| CD8T | CD8T | AC084033.3 | 0.26 | | 3.78E-93 | |
| CD8T | CD8T | C19orf48 | 0.39 | | 1.28E-92 | |
| CD8T | CD8T | LSM6 | 0.52 | | 1.49E-92 | |
| CD8T | CD8T | CDK4 | 0.52 | | 1.71E-92 | |
| CD8T | CD8T | HIST1H4C | 1.61 | | 3.16E-92 | |
| CD8T | CD8T | RPL26L1 | 0.38 | | 3.28E-92 | |
| CD8T | CD8T | HNRNPA2B1 | 0.76 | | 4.44E-92 | |
| CD8T | CD8T | RRP7A | 0.4 | | 6.31E-92 | |
| CD8T | CD8T | SIT1 | 0.3 | | 8.01E-92 | |
| CD8T | CD8T | URM1 | 0.41 | | 8.01E-92 | |
| CD8T | CD8T | GLRX3 | 0.48 | | 1.23E-91 | |
| CD8T | CD8T | GCHFR | 0.42 | | 1.28E-91 | |
| CD8T | CD8T | PSMB8 | 0.68 | | 1.38E-91 | |
| CD8T | CD8T | ERH | 0.75 | | 1.84E-91 | |
| CD8T | CD8T | EXOSC8 | 0.29 | | 2.61E-91 | |
| CD8T | CD8T | TEX30 | 0.27 | | 5.73E-91 | |
| CD8T | CD8T | ANAPC11 | 0.74 | | 7.68E-91 | |
| CD8T | CD8T | MRPS14 | 0.32 | | 6.25E-90 | |
| CD8T | CD8T | LSM3 | 0.68 | | 1.05E-89 | |
| CD8T | CD8T | GLRX5 | 0.32 | | 1.24E-89 | |
| CD8T | CD8T | BUB3 | 0.56 | | 1.29E-89 | |
| CD8T | CD8T | PSMB9 | 0.81 | | 2.56E-89 | |
| CD8T | CD8T | MYL6B | 0.36 | | 3.86E-89 | |
| CD8T | CD8T | STOML2 | 0.54 | | 1.21E-88 | |
| CD8T | CD8T | SHMT2 | 0.41 | | 1.31E-88 | |
| CD8T | CD8T | GALM | 0.48 | | 3.03E-88 | |
| CD8T | CD8T | SUPT16H | 0.27 | | 1.22E-87 | |
| CD8T | CD8T | RFC1 | 0.28 | | 1.68E-87 | |
| CD8T | CD8T | PPM1G | 0.56 | | 1.83E-87 | |
| CD8T | CD8T | MRPS23 | 0.33 | | 2.20E-87 | |
| CD8T | CD8T | PARK7 | 0.75 | | 3.04E-87 | |
| CD8T | CD8T | SRP9 | 0.68 | | 3.70E-87 | |
| CD8T | CD8T | ZDHHC12 | 0.46 | | 4.02E-87 | |
| CD8T | CD8T | EIF2B1 | 0.26 | | 4.36E-87 | |
| CD8T | CD8T | AP1S1 | 0.34 | | 4.44E-87 | |
| CD8T | CD8T | SNRPG | 0.8 | | 5.84E-87 | |
| CD8T | CD8T | ATIC | 0.31 | | 6.23E-87 | |
| CD8T | CD8T | PSMC3 | 0.6 | | 1.04E-86 | |
| CD8T | CD8T | SNRPF | 0.72 | | 1.49E-86 | |
| CD8T | CD8T | TRAPPC1 | 0.68 | | 2.73E-86 | |
| CD8T | CD8T | SNRPB | 0.77 | | 6.45E-86 | |
| CD8T | CD8T | PDZD11 | 0.32 | | 7.01E-86 | |
| CD8T | CD8T | HINT2 | 0.47 | | 1.94E-85 | |
| CD8T | CD8T | HDAC1 | 0.44 | | 2.91E-85 | |
| CD8T | CD8T | SNRPD3 | 0.66 | | 3.42E-85 | |
| CD8T | CD8T | LCP1 | 0.76 | | 3.70E-85 | |
| CD8T | CD8T | CKLF | 0.64 | | 8.44E-85 | |
| CD8T | CD8T | MRPS16 | 0.45 | | 2.86E-84 | |
| CD8T | CD8T | H2AFX | 0.47 | | 3.39E-84 | |
| CD8T | CD8T | PDCD5 | 0.55 | | 3.99E-84 | |
| CD8T | CD8T | SCCPDH | 0.27 | | 1.22E-83 | |
| CD8T | CD8T | NHP2 | 0.65 | | 1.52E-83 | |
| CD8T | CD8T | FDPS | 0.51 | | 1.95E-83 | |
| CD8T | CD8T | IL2RA | 0.89 | | 2.24E-83 | |
| CD8T | CD8T | POLR2H | 0.4 | | 3.17E-83 | |
| CD8T | CD8T | ADA | 0.29 | | 3.67E-83 | |
| CD8T | CD8T | SIVA1 | 0.69 | | 8.04E-83 | |
| CD8T | CD8T | JPT1 | 0.75 | | 1.23E-82 | |
| CD8T | CD8T | NAA38 | 0.57 | | 1.36E-82 | |
| CD8T | CD8T | RBBP4 | 0.48 | | 2.40E-82 | |
| CD8T | CD8T | MT-ND3 | -0.96 | | 3.97E-82 | |
| CD8T | CD8T | ABRACL | 0.72 | | 4.13E-82 | |
| CD8T | CD8T | RPIA | 0.31 | | 5.11E-82 | |
| CD8T | CD8T | TBCB | 0.54 | | 8.69E-82 | |
| CD8T | CD8T | MRPL16 | 0.34 | | 1.42E-81 | |
| CD8T | CD8T | MRPS34 | 0.54 | | 3.78E-81 | |
| CD8T | CD8T | SIRPG | 0.26 | | 6.76E-81 | |
| CD8T | CD8T | NDUFB3 | 0.58 | | 1.06E-80 | |
| CD8T | CD8T | RNASEH2C | 0.53 | | 1.23E-80 | |
| CD8T | CD8T | CPSF6 | 0.32 | | 1.99E-80 | |
| CD8T | CD8T | FKBP1A | 0.65 | | 4.91E-80 | |
| CD8T | CD8T | NUTF2 | 0.5 | | 5.29E-80 | |
| CD8T | CD8T | MRPS11 | 0.3 | | 5.95E-80 | |
| CD8T | CD8T | MRPL11 | 0.49 | | 7.54E-80 | |
| CD8T | CD8T | SRPK1 | 0.28 | | 8.71E-80 | |
| CD8T | CD8T | MRPL22 | 0.36 | | 1.66E-79 | |
| CD8T | CD8T | DAZAP1 | 0.36 | | 1.81E-79 | |
| CD8T | CD8T | POLR2E | 0.53 | | 3.91E-79 | |
| CD8T | CD8T | LBHD1 | 0.46 | | 1.05E-78 | |
| CD8T | CD8T | PSMA4 | 0.71 | | 1.22E-78 | |
| CD8T | CD8T | BOLA3 | 0.42 | | 8.64E-78 | |
| CD8T | CD8T | FAM192A | 0.29 | | 1.09E-77 | |
| CD8T | CD8T | PGP | 0.26 | | 1.28E-77 | |
| CD8T | CD8T | SUMO2 | 0.66 | | 2.89E-77 | |
| CD8T | CD8T | HNRNPAB | 0.51 | | 4.36E-77 | |
| CD8T | CD8T | ELAVL1 | 0.34 | | 6.69E-77 | |
| CD8T | CD8T | SLC25A11 | 0.36 | | 8.56E-77 | |
| CD8T | CD8T | FABP5 | 0.56 | | 1.34E-76 | |
| CD8T | CD8T | MRPL12 | 0.52 | | 1.35E-76 | |
| CD8T | CD8T | MRPL28 | 0.36 | | 3.00E-76 | |
| CD8T | CD8T | LSM5 | 0.7 | | 4.98E-76 | |
| CD8T | CD8T | THOC7 | 0.5 | | 2.20E-75 | |
| CD8T | CD8T | PDAP1 | 0.47 | | 2.75E-75 | |
| CD8T | CD8T | HNRNPR | 0.52 | | 2.75E-75 | |
| CD8T | CD8T | FAM207A | 0.29 | | 2.97E-75 | |
| CD8T | CD8T | NDUFA12 | 0.54 | | 3.66E-75 | |
| CD8T | CD8T | POLR2G | 0.47 | | 4.00E-75 | |
| CD8T | CD8T | RPA2 | 0.32 | | 8.63E-75 | |
| CD8T | CD8T | PPP1R7 | 0.36 | | 9.75E-75 | |
| CD8T | CD8T | FARSA | 0.25 | | 2.09E-74 | |
| CD8T | CD8T | MDH1 | 0.54 | | 8.37E-74 | |
| CD8T | CD8T | MDH2 | 0.59 | | 1.30E-73 | |
| CD8T | CD8T | POP5 | 0.25 | | 1.63E-73 | |
| CD8T | CD8T | POLE3 | 0.27 | | 2.06E-73 | |
| CD8T | CD8T | EEF1E1 | 0.3 | | 2.44E-73 | |
| CD8T | CD8T | IAH1 | 0.38 | | 3.97E-73 | |
| CD8T | CD8T | MZT2A | 0.64 | | 6.19E-73 | |
| CD8T | CD8T | PPIL3 | 0.3 | | 9.18E-73 | |
| CD8T | CD8T | MEA1 | 0.4 | | 1.95E-72 | |
| CD8T | CD8T | VPS25 | 0.27 | | 1.97E-72 | |
| CD8T | CD8T | MRPS15 | 0.46 | | 2.62E-72 | |
| CD8T | CD8T | MRPL4 | 0.47 | | 3.95E-72 | |
| CD8T | CD8T | NPM3 | 0.36 | | 6.79E-72 | |
| CD8T | CD8T | SNRPC | 0.54 | | 7.55E-72 | |
| CD8T | CD8T | ATP5MG | 0.6 | | 7.93E-72 | |
| CD8T | CD8T | SLBP | 0.61 | | 8.14E-72 | |
| CD8T | CD8T | RAD21 | 0.43 | | 8.43E-72 | |
| CD8T | CD8T | PPP1R18 | 0.42 | | 8.96E-72 | |
| CD8T | CD8T | PSMC5 | 0.52 | | 9.55E-72 | |
| CD8T | CD8T | RUVBL2 | 0.31 | | 1.01E-71 | |
| CD8T | CD8T | RBX1 | 0.64 | | 1.13E-71 | |
| CD8T | CD8T | ATP5MF | 0.69 | | 1.18E-71 | |
| CD8T | CD8T | VPS29 | 0.52 | | 1.57E-71 | |
| CD8T | CD8T | PSMA7 | 0.67 | | 1.94E-71 | |
| CD8T | CD8T | PSMD4 | 0.46 | | 3.36E-71 | |
| CD8T | CD8T | SH2D1A | 0.31 | | 4.94E-71 | |
| CD8T | CD8T | VDAC3 | 0.47 | | 7.46E-71 | |
| CD8T | CD8T | CYB5B | 0.27 | | 8.95E-71 | |
| CD8T | CD8T | UBE2V2 | 0.32 | | 1.17E-70 | |
| CD8T | CD8T | PPIE | 0.26 | | 1.24E-70 | |
| CD8T | CD8T | PYCR2 | 0.26 | | 1.68E-70 | |
| CD8T | CD8T | AP2S1 | 0.5 | | 2.07E-70 | |
| CD8T | CD8T | GPS1 | 0.26 | | 2.38E-70 | |
| CD8T | CD8T | PTMA | 0.56 | | 4.20E-70 | |
| CD8T | CD8T | BAX | 0.54 | | 4.36E-70 | |
| CD8T | CD8T | RALY | 0.58 | | 6.10E-70 | |
| CD8T | CD8T | BABAM1 | 0.34 | | 8.60E-70 | |
| CD8T | CD8T | PSME2 | 0.74 | | 8.60E-70 | |
| CD8T | CD8T | ATP5PF | 0.61 | | 1.12E-69 | |
| CD8T | CD8T | DCTN3 | 0.5 | | 1.36E-69 | |
| CD8T | CD8T | EBNA1BP2 | 0.4 | | 1.42E-69 | |
| CD8T | CD8T | CCT2 | 0.51 | | 1.69E-69 | |
| CD8T | CD8T | ACTR3 | 0.7 | | 1.72E-69 | |
| CD8T | CD8T | POLDIP2 | 0.27 | | 1.73E-69 | |
| CD8T | CD8T | RPP30 | 0.26 | | 4.76E-69 | |
| CD8T | CD8T | C20orf27 | 0.32 | | 6.63E-69 | |
| CD8T | CD8T | DCUN1D5 | 0.28 | | 7.83E-69 | |
| CD8T | CD8T | PSMC2 | 0.34 | | 7.95E-69 | |
| CD8T | CD8T | DBNL | 0.4 | | 8.23E-69 | |
| CD8T | CD8T | SHKBP1 | 0.31 | | 9.19E-69 | |
| CD8T | CD8T | UFD1 | 0.39 | | 1.07E-68 | |
| CD8T | CD8T | ZCRB1 | 0.36 | | 3.27E-68 | |
| CD8T | CD8T | LYRM4 | 0.29 | | 3.91E-68 | |
| CD8T | CD8T | MYL6 | 0.56 | | 5.96E-68 | |
| CD8T | CD8T | UQCC2 | 0.44 | | 2.20E-67 | |
| CD8T | CD8T | ETFB | 0.49 | | 2.40E-67 | |
| CD8T | CD8T | RAB27A | 0.36 | | 3.00E-67 | |
| CD8T | CD8T | SUMO3 | 0.46 | | 3.85E-67 | |
| CD8T | CD8T | FOXP3 | 0.25 | | 5.09E-67 | |
| CD8T | CD8T | MYO1G | 0.35 | | 7.70E-67 | |
| CD8T | CD8T | NEDD8 | 0.57 | | 2.48E-66 | |
| CD8T | CD8T | CHCHD3 | 0.38 | | 2.87E-66 | |
| CD8T | CD8T | NONO | 0.46 | | 3.47E-66 | |
| CD8T | CD8T | TPRKB | 0.36 | | 5.01E-66 | |
| CD8T | CD8T | THOC6 | 0.27 | | 6.76E-66 | |
| CD8T | CD8T | TXN2 | 0.4 | | 7.64E-66 | |
| CD8T | CD8T | HSD17B10 | 0.5 | | 8.59E-66 | |
| CD8T | CD8T | CCT5 | 0.47 | | 8.96E-66 | |
| CD8T | CD8T | MRPS12 | 0.4 | | 1.05E-65 | |
| CD8T | CD8T | XRCC5 | 0.51 | | 1.22E-65 | |
| CD8T | CD8T | PHF5A | 0.33 | | 1.25E-65 | |
| CD8T | CD8T | MRPL15 | 0.32 | | 2.33E-65 | |
| CD8T | CD8T | ATP5F1C | 0.58 | | 2.97E-65 | |
| CD8T | CD8T | HNRNPA1 | 0.59 | | 3.89E-65 | |
| CD8T | CD8T | MRPL27 | 0.4 | | 4.38E-65 | |
| CD8T | CD8T | ARPC5L | 0.42 | | 4.42E-65 | |
| CD8T | CD8T | LCK | 0.52 | | 5.69E-65 | |
| CD8T | CD8T | DBI | 0.69 | | 5.87E-65 | |
| CD8T | CD8T | EBP | 0.39 | | 6.29E-65 | |
| CD8T | CD8T | POP4 | 0.27 | | 7.06E-65 | |
| CD8T | CD8T | MIR4435-2HG | 0.42 | | 7.10E-65 | |
| CD8T | CD8T | NDUFA9 | 0.31 | | 9.84E-65 | |
| CD8T | CD8T | ATP5PB | 0.54 | | 1.89E-64 | |
| CD8T | CD8T | NDUFV1 | 0.45 | | 1.97E-64 | |
| CD8T | CD8T | ARL6IP6 | 0.28 | | 2.48E-64 | |
| CD8T | CD8T | RAB8A | 0.35 | | 3.03E-64 | |
| CD8T | CD8T | PSMB10 | 0.43 | | 3.27E-64 | |
| CD8T | CD8T | SSBP1 | 0.59 | | 3.41E-64 | |
| CD8T | CD8T | CNN2 | 0.51 | | 9.36E-64 | |
| CD8T | CD8T | C1QBP | 0.68 | | 1.03E-63 | |
| CD8T | CD8T | KPNA2 | 0.5 | | 1.08E-63 | |
| CD8T | CD8T | CCT8 | 0.61 | | 2.06E-63 | |
| CD8T | CD8T | TTC39C | 0.25 | | 2.40E-63 | |
| CD8T | CD8T | BAK1 | 0.27 | | 5.78E-63 | |
| CD8T | CD8T | GMFG | 0.71 | | 6.05E-63 | |
| CD8T | CD8T | YWHAQ | 0.59 | | 6.11E-63 | |
| CD8T | CD8T | RBM17 | 0.45 | | 7.68E-63 | |
| CD8T | CD8T | RNF167 | 0.34 | | 8.06E-63 | |
| CD8T | CD8T | MRPL9 | 0.29 | | 8.67E-63 | |
| CD8T | CD8T | YWHAH | 0.46 | | 8.79E-63 | |
| CD8T | CD8T | MRTO4 | 0.27 | | 9.44E-63 | |
| CD8T | CD8T | PSMG2 | 0.38 | | 1.18E-62 | |
| CD8T | CD8T | APOBEC3C | 0.35 | | 1.29E-62 | |
| CD8T | CD8T | ILK | 0.27 | | 1.34E-62 | |
| CD8T | CD8T | ETFA | 0.39 | | 1.40E-62 | |
| CD8T | CD8T | PHB | 0.51 | | 1.70E-62 | |
| CD8T | CD8T | MRPL52 | 0.51 | | 1.73E-62 | |
| CD8T | CD8T | MRPL17 | 0.27 | | 5.98E-62 | |
| CD8T | CD8T | 1-Sep | 0.44 | | 6.12E-62 | |
| CD8T | CD8T | CLIC1 | 0.6 | | 7.20E-62 | |
| CD8T | CD8T | BLOC1S1 | 0.58 | | 7.68E-62 | |
| CD8T | CD8T | PSMD3 | 0.33 | | 8.32E-62 | |
| CD8T | CD8T | SSNA1 | 0.44 | | 2.64E-61 | |
| CD8T | CD8T | UBA2 | 0.29 | | 3.05E-61 | |
| CD8T | CD8T | NAP1L4 | 0.29 | | 4.30E-61 | |
| CD8T | CD8T | EIF4EBP1 | 0.29 | | 4.72E-61 | |
| CD8T | CD8T | CAP1 | 0.53 | | 5.05E-61 | |
| CD8T | CD8T | NDUFB6 | 0.47 | | 5.17E-61 | |
| CD8T | CD8T | EIF5A | 0.68 | | 5.20E-61 | |
| CD8T | CD8T | AKR1B1 | 0.4 | | 6.09E-61 | |
| CD8T | CD8T | EIF4E2 | 0.32 | | 8.66E-61 | |
| CD8T | CD8T | MRPL34 | 0.29 | | 1.27E-60 | |
| CD8T | CD8T | PRDX2 | 0.67 | | 1.27E-60 | |
| CD8T | CD8T | NAA10 | 0.46 | | 1.43E-60 | |
| CD8T | CD8T | MRPL23 | 0.52 | | 1.49E-60 | |
| CD8T | CD8T | UQCRH | 0.61 | | 2.36E-60 | |
| CD8T | CD8T | PSMD8 | 0.53 | | 2.46E-60 | |
| CD8T | CD8T | RPSA | 0.47 | | 2.62E-60 | |
| CD8T | CD8T | NUDCD2 | 0.29 | | 3.07E-60 | |
| CD8T | CD8T | OLA1 | 0.33 | | 3.38E-60 | |
| CD8T | CD8T | COMMD7 | 0.3 | | 5.65E-60 | |
| CD8T | CD8T | DECR1 | 0.35 | | 6.63E-60 | |
| CD8T | CD8T | TMEM160 | 0.44 | | 7.90E-60 | |
| CD8T | CD8T | CLPP | 0.33 | | 7.93E-60 | |
| CD8T | CD8T | PRPF19 | 0.26 | | 8.45E-60 | |
| CD8T | CD8T | PHB2 | 0.46 | | 8.71E-60 | |
| CD8T | CD8T | CAPZB | 0.6 | | 8.93E-60 | |
| CD8T | CD8T | MPC2 | 0.48 | | 1.15E-59 | |
| CD8T | CD8T | UCP2 | 0.5 | | 1.68E-59 | |
| CD8T | CD8T | EIF2S1 | 0.3 | | 2.00E-59 | |
| CD8T | CD8T | AKR7A2 | 0.36 | | 2.16E-59 | |
| CD8T | CD8T | PRELID1 | 0.69 | | 2.19E-59 | |
| CD8T | CD8T | UBE2S | 0.59 | | 2.31E-59 | |
| CD8T | CD8T | TESC | 0.28 | | 2.44E-59 | |
| CD8T | CD8T | DCTN2 | 0.34 | | 3.04E-59 | |
| CD8T | CD8T | CSNK2B | 0.52 | | 3.53E-59 | |
| CD8T | CD8T | NDUFA4 | 0.52 | | 3.58E-59 | |
| CD8T | CD8T | STIP1 | 0.27 | | 3.67E-59 | |
| CD8T | CD8T | TMEM14A | 0.27 | | 4.20E-59 | |
| CD8T | CD8T | HAT1 | 0.33 | | 6.33E-59 | |
| CD8T | CD8T | UBE2I | 0.54 | | 8.22E-59 | |
| CD8T | CD8T | LAIR2 | 0.54 | | 8.92E-59 | |
| CD8T | CD8T | AK2 | 0.35 | | 9.23E-59 | |
| CD8T | CD8T | ATP5IF1 | 0.54 | | 1.22E-58 | |
| CD8T | CD8T | LAMTOR2 | 0.45 | | 1.87E-58 | |
| CD8T | CD8T | HINT1 | 0.51 | | 1.90E-58 | |
| CD8T | CD8T | CIAO2A | 0.37 | | 2.04E-58 | |
| CD8T | CD8T | AHSA1 | 0.32 | | 2.39E-58 | |
| CD8T | CD8T | HIKESHI | 0.3 | | 2.39E-58 | |
| CD8T | CD8T | ARPC3 | 0.63 | | 2.87E-58 | |
| CD8T | CD8T | CCT7 | 0.44 | | 3.48E-58 | |
| CD8T | CD8T | ACP1 | 0.43 | | 4.37E-58 | |
| CD8T | CD8T | FAM136A | 0.26 | | 4.75E-58 | |
| CD8T | CD8T | NUDC | 0.46 | | 5.49E-58 | |
| CD8T | CD8T | CCDC124 | 0.3 | | 5.59E-58 | |
| CD8T | CD8T | NDUFS8 | 0.5 | | 5.97E-58 | |
| CD8T | CD8T | TWF2 | 0.29 | | 6.92E-58 | |
| CD8T | CD8T | TOMM40 | 0.3 | | 8.62E-58 | |
| CD8T | CD8T | FOS | -1.54 | | 9.42E-58 | |
| CD8T | CD8T | TMEM109 | 0.27 | | 1.00E-57 | |
| CD8T | CD8T | RPL34 | -0.55 | | 1.38E-57 | |
| CD8T | CD8T | HDDC2 | 0.31 | | 1.44E-57 | |
| CD8T | CD8T | GLO1 | 0.31 | | 1.77E-57 | |
| CD8T | CD8T | CCT3 | 0.49 | | 3.04E-57 | |
| CD8T | CD8T | SLC25A3 | 0.57 | | 3.06E-57 | |
| CD8T | CD8T | SERBP1 | 0.61 | | 3.43E-57 | |
| CD8T | CD8T | ATP5MC1 | 0.61 | | 4.57E-57 | |
| CD8T | CD8T | EIF2S2 | 0.55 | | 6.75E-57 | |
| CD8T | CD8T | DMAC1 | 0.35 | | 7.32E-57 | |
| CD8T | CD8T | MRPL14 | 0.36 | | 1.67E-56 | |
| CD8T | CD8T | MRPL18 | 0.37 | | 1.89E-56 | |
| CD8T | CD8T | NDUFV2 | 0.54 | | 1.99E-56 | |
| CD8T | CD8T | CCDC69 | 0.32 | | 2.27E-56 | |
| CD8T | CD8T | MRPL42 | 0.3 | | 2.90E-56 | |
| CD8T | CD8T | NPM1 | 0.6 | | 3.54E-56 | |
| CD8T | CD8T | NUBP2 | 0.28 | | 4.93E-56 | |
| CD8T | CD8T | GRHPR | 0.35 | | 8.10E-56 | |
| CD8T | CD8T | PSMA1 | 0.5 | | 9.01E-56 | |
| CD8T | CD8T | COX6B1 | 0.52 | | 9.68E-56 | |
| CD8T | CD8T | ZBTB8OS | 0.36 | | 1.07E-55 | |
| CD8T | CD8T | TCP1 | 0.43 | | 1.12E-55 | |
| CD8T | CD8T | SLC25A39 | 0.43 | | 1.18E-55 | |
| CD8T | CD8T | TIMM8B | 0.42 | | 1.38E-55 | |
| CD8T | CD8T | GTF3C6 | 0.5 | | 2.54E-55 | |
| CD8T | CD8T | PSMC1 | 0.37 | | 3.74E-55 | |
| CD8T | CD8T | NDUFAB1 | 0.54 | | 3.77E-55 | |
| CD8T | CD8T | TMSB4X | 0.63 | | 3.79E-55 | |
| CD8T | CD8T | PLEKHJ1 | 0.3 | | 4.60E-55 | |
| CD8T | CD8T | HNRNPA3 | 0.56 | | 4.72E-55 | |
| CD8T | CD8T | GDI2 | 0.57 | | 5.88E-55 | |
| CD8T | CD8T | KXD1 | 0.3 | | 1.06E-54 | |
| CD8T | CD8T | LUC7L2 | 0.28 | | 1.37E-54 | |
| CD8T | CD8T | HDGF | 0.28 | | 1.62E-54 | |
| CD8T | CD8T | HNRNPF | 0.53 | | 1.71E-54 | |
| CD8T | CD8T | CISD1 | 0.28 | | 1.84E-54 | |
| CD8T | CD8T | POLE4 | 0.34 | | 2.01E-54 | |
| CD8T | CD8T | LDHA | 0.61 | | 2.21E-54 | |
| CD8T | CD8T | SRSF9 | 0.5 | | 2.71E-54 | |
| CD8T | CD8T | IFI16 | 0.38 | | 3.08E-54 | |
| CD8T | CD8T | ARF5 | 0.43 | | 4.54E-54 | |
| CD8T | CD8T | ALDOA | 0.54 | | 4.99E-54 | |
| CD8T | CD8T | SELENOH | 0.56 | | 5.12E-54 | |
| CD8T | CD8T | MRPL36 | 0.26 | | 5.23E-54 | |
| CD8T | CD8T | CDC123 | 0.27 | | 5.27E-54 | |
| CD8T | CD8T | RPS27 | -0.66 | | 6.25E-54 | |
| CD8T | CD8T | GNG5 | 0.54 | | 6.75E-54 | |
| CD8T | CD8T | COX6C | 0.51 | | 8.97E-54 | |
| CD8T | CD8T | ATP5F1B | 0.56 | | 9.17E-54 | |
| CD8T | CD8T | NDUFB2 | 0.55 | | 1.00E-53 | |
| CD8T | CD8T | ACADM | 0.26 | | 1.01E-53 | |
| CD8T | CD8T | LSP1 | 0.66 | | 1.88E-53 | |
| CD8T | CD8T | ARHGDIB | 0.64 | | 1.97E-53 | |
| CD8T | CD8T | VDAC1 | 0.52 | | 2.11E-53 | |
| CD8T | CD8T | PSMD2 | 0.29 | | 2.38E-53 | |
| CD8T | CD8T | LAGE3 | 0.35 | | 2.57E-53 | |
| CD8T | CD8T | ECH1 | 0.47 | | 2.85E-53 | |
| CD8T | CD8T | TIMM10 | 0.38 | | 3.77E-53 | |
| CD8T | CD8T | PSMB6 | 0.53 | | 3.95E-53 | |
| CD8T | CD8T | HCLS1 | 0.45 | | 4.03E-53 | |
| CD8T | CD8T | AHCY | 0.32 | | 4.19E-53 | |
| CD8T | CD8T | PSMA3 | 0.44 | | 7.22E-53 | |
| CD8T | CD8T | CYC1 | 0.57 | | 7.46E-53 | |
| CD8T | CD8T | PSMD7 | 0.44 | | 1.49E-52 | |
| CD8T | CD8T | PSMD13 | 0.35 | | 1.52E-52 | |
| CD8T | CD8T | GTF2A2 | 0.39 | | 1.53E-52 | |
| CD8T | CD8T | CMSS1 | 0.27 | | 2.34E-52 | |
| CD8T | CD8T | GTF3A | 0.51 | | 2.74E-52 | |
| CD8T | CD8T | NDUFS3 | 0.35 | | 3.13E-52 | |
| CD8T | CD8T | H3F3A | 0.52 | | 3.24E-52 | |
| CD8T | CD8T | LYPLA1 | 0.36 | | 3.92E-52 | |
| CD8T | CD8T | CCT6A | 0.42 | | 5.15E-52 | |
| CD8T | CD8T | COX17 | 0.5 | | 5.60E-52 | |
| CD8T | CD8T | NDUFS6 | 0.51 | | 7.24E-52 | |
| CD8T | CD8T | UBE2K | 0.31 | | 1.29E-51 | |
| CD8T | CD8T | DNPH1 | 0.5 | | 1.71E-51 | |
| CD8T | CD8T | HNRNPD | 0.41 | | 1.92E-51 | |
| CD8T | CD8T | SUCLG1 | 0.31 | | 3.24E-51 | |
| CD8T | CD8T | PFDN6 | 0.34 | | 3.85E-51 | |
| CD8T | CD8T | TXNL4A | 0.28 | | 5.66E-51 | |
| CD8T | CD8T | DUSP1 | -1.16 | | 9.26E-51 | |
| CD8T | CD8T | RGS10 | 0.41 | | 1.24E-50 | |
| CD8T | CD8T | SNX17 | 0.29 | | 1.30E-50 | |
| CD8T | CD8T | STMP1 | 0.35 | | 1.55E-50 | |
| CD8T | CD8T | SQOR | 0.3 | | 1.80E-50 | |
| CD8T | CD8T | GSDMD | 0.31 | | 2.03E-50 | |
| CD8T | CD8T | PPP4C | 0.44 | | 2.27E-50 | |
| CD8T | CD8T | UBE2N | 0.47 | | 3.05E-50 | |
| CD8T | CD8T | FERMT3 | 0.26 | | 4.59E-50 | |
| CD8T | CD8T | CD70 | 0.37 | | 4.74E-50 | |
| CD8T | CD8T | TCEA1 | 0.56 | | 5.66E-50 | |
| CD8T | CD8T | COMMD3 | 0.27 | | 6.34E-50 | |
| CD8T | CD8T | POLR2J | 0.37 | | 6.62E-50 | |
| CD8T | CD8T | DGUOK | 0.36 | | 7.13E-50 | |
| CD8T | CD8T | HIGD1A | 0.31 | | 7.89E-50 | |
| CD8T | CD8T | COX20 | 0.31 | | 1.55E-49 | |
| CD8T | CD8T | BRK1 | 0.5 | | 2.07E-49 | |
| CD8T | CD8T | CLTA | 0.46 | | 2.10E-49 | |
| CD8T | CD8T | PDCD2 | 0.3 | | 2.26E-49 | |
| CD8T | CD8T | MRPS18C | 0.28 | | 2.54E-49 | |
| CD8T | CD8T | LIMD2 | 0.56 | | 3.96E-49 | |
| CD8T | CD8T | UBE2L3 | 0.51 | | 4.15E-49 | |
| CD8T | CD8T | SH3KBP1 | 0.28 | | 4.65E-49 | |
| CD8T | CD8T | POMP | 0.5 | | 4.98E-49 | |
| CD8T | CD8T | RPLP0 | 0.41 | | 5.45E-49 | |
| CD8T | CD8T | PSMD11 | 0.25 | | 5.52E-49 | |
| CD8T | CD8T | ITPA | 0.29 | | 5.87E-49 | |
| CD8T | CD8T | GTF2H5 | 0.29 | | 6.46E-49 | |
| CD8T | CD8T | OSTF1 | 0.4 | | 8.15E-49 | |
| CD8T | CD8T | CDK2AP2 | 0.38 | | 1.04E-48 | |
| CD8T | CD8T | ADRM1 | 0.41 | | 1.11E-48 | |
| CD8T | CD8T | NASP | 0.44 | | 1.66E-48 | |
| CD8T | CD8T | SET | 0.51 | | 1.71E-48 | |
| CD8T | CD8T | DDX46 | 0.3 | | 2.06E-48 | |
| CD8T | CD8T | ATP5F1A | 0.56 | | 2.15E-48 | |
| CD8T | CD8T | CHCHD1 | 0.29 | | 2.30E-48 | |
| CD8T | CD8T | SF3B6 | 0.5 | | 2.44E-48 | |
| CD8T | CD8T | CCND3 | 0.31 | | 3.26E-48 | |
| CD8T | CD8T | MTHFD2 | 0.3 | | 3.63E-48 | |
| CD8T | CD8T | ASNA1 | 0.29 | | 5.29E-48 | |
| CD8T | CD8T | GYG1 | 0.27 | | 6.57E-48 | |
| CD8T | CD8T | ATXN10 | 0.26 | | 7.58E-48 | |
| CD8T | CD8T | METTL5 | 0.26 | | 9.77E-48 | |
| CD8T | CD8T | TKT | 0.43 | | 1.05E-47 | |
| CD8T | CD8T | IMP4 | 0.29 | | 1.14E-47 | |
| CD8T | CD8T | UBE2D2 | 0.48 | | 1.35E-47 | |
| CD8T | CD8T | SNX5 | 0.27 | | 1.46E-47 | |
| CD8T | CD8T | MRPS6 | 0.38 | | 1.72E-47 | |
| CD8T | CD8T | HSPD1 | 0.55 | | 2.02E-47 | |
| CD8T | CD8T | ARRB2 | 0.27 | | 2.92E-47 | |
| CD8T | CD8T | ISOC2 | 0.32 | | 3.83E-47 | |
| CD8T | CD8T | NSMCE1 | 0.26 | | 5.95E-47 | |
| CD8T | CD8T | DNAJC15 | 0.37 | | 6.43E-47 | |
| CD8T | CD8T | PPA1 | 0.52 | | 7.02E-47 | |
| CD8T | CD8T | SDHB | 0.32 | | 7.66E-47 | |
| CD8T | CD8T | GSTO1 | 0.3 | | 7.70E-47 | |
| CD8T | CD8T | NDUFAF3 | 0.38 | | 8.10E-47 | |
| CD8T | CD8T | PTRHD1 | 0.29 | | 1.05E-46 | |
| CD8T | CD8T | PCMT1 | 0.31 | | 2.17E-46 | |
| CD8T | CD8T | TUBA1C | 0.52 | | 2.88E-46 | |
| CD8T | CD8T | SUMO1 | 0.47 | | 4.84E-46 | |
| CD8T | CD8T | UQCR10 | 0.51 | | 5.79E-46 | |
| CD8T | CD8T | SEM1 | 0.47 | | 6.04E-46 | |
| CD8T | CD8T | MCTS1 | 0.28 | | 8.68E-46 | |
| CD8T | CD8T | SNF8 | 0.27 | | 9.62E-46 | |
| CD8T | CD8T | UBE2L6 | 0.3 | | 1.04E-45 | |
| CD8T | CD8T | PSMB7 | 0.4 | | 1.04E-45 | |
| CD8T | CD8T | PNKD | 0.26 | | 1.36E-45 | |
| CD8T | CD8T | ARL6IP4 | 0.49 | | 1.39E-45 | |
| CD8T | CD8T | ANAPC5 | 0.25 | | 1.70E-45 | |
| CD8T | CD8T | AIP | 0.3 | | 2.00E-45 | |
| CD8T | CD8T | ELOB | 0.45 | | 2.37E-45 | |
| CD8T | CD8T | CD247 | 0.34 | | 3.59E-45 | |
| CD8T | CD8T | ECHS1 | 0.33 | | 4.31E-45 | |
| CD8T | CD8T | THRAP3 | 0.26 | | 4.82E-45 | |
| CD8T | CD8T | PMF1 | 0.33 | | 5.65E-45 | |
| CD8T | CD8T | NDUFB10 | 0.44 | | 6.02E-45 | |
| CD8T | CD8T | EIF1 | -0.49 | | 6.24E-45 | |
| CD8T | CD8T | TIMM17A | 0.27 | | 6.43E-45 | |
| CD8T | CD8T | SRP14 | 0.41 | | 7.85E-45 | |
| CD8T | CD8T | SLIRP | 0.5 | | 9.54E-45 | |
| CD8T | CD8T | NDUFS7 | 0.37 | | 1.13E-44 | |
| CD8T | CD8T | MRPL47 | 0.27 | | 1.31E-44 | |
| CD8T | CD8T | TPM3 | 0.52 | | 1.44E-44 | |
| CD8T | CD8T | SNRNP70 | 0.34 | | 1.68E-44 | |
| CD8T | CD8T | MRPS33 | 0.31 | | 1.88E-44 | |
| CD8T | CD8T | TXNDC17 | 0.39 | | 2.62E-44 | |
| CD8T | CD8T | ANXA6 | 0.35 | | 2.92E-44 | |
| CD8T | CD8T | UQCRQ | 0.49 | | 5.40E-44 | |
| CD8T | CD8T | TMA7 | 0.46 | | 5.92E-44 | |
| CD8T | CD8T | LSM14A | 0.26 | | 6.03E-44 | |
| CD8T | CD8T | ILF2 | 0.46 | | 6.03E-44 | |
| CD8T | CD8T | PSMF1 | 0.26 | | 7.07E-44 | |
| CD8T | CD8T | GNAI2 | 0.32 | | 7.39E-44 | |
| CD8T | CD8T | PRDX6 | 0.5 | | 8.56E-44 | |
| CD8T | CD8T | ELOF1 | 0.25 | | 1.07E-43 | |
| CD8T | CD8T | MCUB | 0.26 | | 1.10E-43 | |
| CD8T | CD8T | MIEN1 | 0.28 | | 1.21E-43 | |
| CD8T | CD8T | UQCRC1 | 0.34 | | 1.62E-43 | |
| CD8T | CD8T | COPS6 | 0.3 | | 1.67E-43 | |
| CD8T | CD8T | SMARCB1 | 0.36 | | 1.82E-43 | |
| CD8T | CD8T | PSMB5 | 0.31 | | 1.93E-43 | |
| CD8T | CD8T | DNAJC8 | 0.36 | | 2.24E-43 | |
| CD8T | CD8T | MT2A | 0.41 | | 2.85E-43 | |
| CD8T | CD8T | GLRX | 0.47 | | 3.22E-43 | |
| CD8T | CD8T | DPY30 | 0.26 | | 3.26E-43 | |
| CD8T | CD8T | ATP5PD | 0.47 | | 3.31E-43 | |
| CD8T | CD8T | NDUFAF8 | 0.39 | | 3.56E-43 | |
| CD8T | CD8T | IMPDH2 | 0.4 | | 7.36E-43 | |
| CD8T | CD8T | TSTA3 | 0.31 | | 8.39E-43 | |
| CD8T | CD8T | PTBP1 | 0.3 | | 9.62E-43 | |
| CD8T | CD8T | RPF1 | 0.25 | | 1.11E-42 | |
| CD8T | CD8T | JUN | -1.23 | | 1.46E-42 | |
| CD8T | CD8T | MRPL20 | 0.44 | | 1.54E-42 | |
| CD8T | CD8T | NDUFA2 | 0.44 | | 1.92E-42 | |
| CD8T | CD8T | LSM7 | 0.42 | | 2.05E-42 | |
| CD8T | CD8T | LAT | 0.33 | | 2.91E-42 | |
| CD8T | CD8T | KHDRBS1 | 0.34 | | 2.92E-42 | |
| CD8T | CD8T | QARS | 0.3 | | 3.01E-42 | |
| CD8T | CD8T | PSMB1 | 0.46 | | 3.44E-42 | |
| CD8T | CD8T | RTF2 | 0.25 | | 3.55E-42 | |
| CD8T | CD8T | ZFP36 | -1.11 | | 3.68E-42 | |
| CD8T | CD8T | CWC15 | 0.26 | | 3.91E-42 | |
| CD8T | CD8T | TXN | 0.39 | | 5.67E-42 | |
| CD8T | CD8T | CXCR6 | 0.36 | | 7.72E-42 | |
| CD8T | CD8T | ATP6V1E1 | 0.26 | | 1.12E-41 | |
| CD8T | CD8T | MMADHC | 0.29 | | 1.39E-41 | |
| CD8T | CD8T | TMEM14B | 0.34 | | 1.79E-41 | |
| CD8T | CD8T | SDHC | 0.33 | | 1.80E-41 | |
| CD8T | CD8T | SRP19 | 0.25 | | 2.24E-41 | |
| CD8T | CD8T | U2AF1 | 0.43 | | 3.36E-41 | |
| CD8T | CD8T | NAA50 | 0.26 | | 3.55E-41 | |
| CD8T | CD8T | PRDX1 | 0.56 | | 3.68E-41 | |
| CD8T | CD8T | FAM49B | 0.33 | | 4.07E-41 | |
| CD8T | CD8T | UQCRFS1 | 0.4 | | 4.54E-41 | |
| CD8T | CD8T | KARS | 0.26 | | 7.24E-41 | |
| CD8T | CD8T | HMGN3 | 0.38 | | 7.92E-41 | |
| CD8T | CD8T | EMC6 | 0.26 | | 7.99E-41 | |
| CD8T | CD8T | HNRNPC | 0.5 | | 9.48E-41 | |
| CD8T | CD8T | PRMT1 | 0.41 | | 1.09E-40 | |
| CD8T | CD8T | MRPL21 | 0.28 | | 1.72E-40 | |
| CD8T | CD8T | ATP5MC2 | 0.45 | | 2.24E-40 | |
| CD8T | CD8T | COA3 | 0.32 | | 2.83E-40 | |
| CD8T | CD8T | LSM1 | 0.25 | | 4.05E-40 | |
| CD8T | CD8T | TUFM | 0.43 | | 5.13E-40 | |
| CD8T | CD8T | PPP1CC | 0.41 | | 6.13E-40 | |
| CD8T | CD8T | ATP5F1E | 0.41 | | 6.91E-40 | |
| CD8T | CD8T | TCL1A | 0.96 | | 7.39E-40 | |
| CD8T | CD8T | CCT4 | 0.42 | | 9.98E-40 | |
| CD8T | CD8T | RHOA | 0.43 | | 1.32E-39 | |
| CD8T | CD8T | ELOC | 0.4 | | 1.41E-39 | |
| CD8T | CD8T | RGS13 | 0.61 | | 3.26E-39 | |
| CD8T | CD8T | COPE | 0.47 | | 3.32E-39 | |
| CD8T | CD8T | UBE2A | 0.33 | | 3.55E-39 | |
| CD8T | CD8T | PIH1D1 | 0.28 | | 3.76E-39 | |
| CD8T | CD8T | TNFRSF18 | 0.86 | | 3.78E-39 | |
| CD8T | CD8T | SH3BGRL3 | 0.62 | | 5.23E-39 | |
| CD8T | CD8T | NDUFB9 | 0.46 | | 6.07E-39 | |
| CD8T | CD8T | MPG | 0.25 | | 7.51E-39 | |
| CD8T | CD8T | METAP2 | 0.41 | | 7.56E-39 | |
| CD8T | CD8T | TOMM5 | 0.28 | | 9.59E-39 | |
| CD8T | CD8T | JUNB | -1.28 | | 9.81E-39 | |
| CD8T | CD8T | BUD31 | 0.36 | | 1.02E-38 | |
| CD8T | CD8T | PGLS | 0.29 | | 1.06E-38 | |
| CD8T | CD8T | RPS27L | 0.49 | | 1.07E-38 | |
| CD8T | CD8T | APOBEC3G | 0.26 | | 1.25E-38 | |
| CD8T | CD8T | MT-ND2 | -0.62 | | 1.33E-38 | |
| CD8T | CD8T | IFI27L2 | 0.31 | | 1.33E-38 | |
| CD8T | CD8T | SRI | 0.4 | | 1.77E-38 | |
| CD8T | CD8T | SERF2 | 0.43 | | 3.67E-38 | |
| CD8T | CD8T | ESD | 0.28 | | 4.45E-38 | |
| CD8T | CD8T | GNB2 | 0.34 | | 5.21E-38 | |
| CD8T | CD8T | SH2D2A | 0.28 | | 5.55E-38 | |
| CD8T | CD8T | 6-Sep | 0.33 | | 7.59E-38 | |
| CD8T | CD8T | MYL12A | 0.5 | | 7.77E-38 | |
| CD8T | CD8T | SOD1 | 0.49 | | 1.09E-37 | |
| CD8T | CD8T | YWHAE | 0.43 | | 1.10E-37 | |
| CD8T | CD8T | TAP1 | 0.3 | | 2.29E-37 | |
| CD8T | CD8T | LSM8 | 0.34 | | 2.39E-37 | |
| CD8T | CD8T | FKBP8 | 0.37 | | 3.31E-37 | |
| CD8T | CD8T | PTGES3 | 0.44 | | 3.41E-37 | |
| CD8T | CD8T | FOSB | -1.11 | | 3.79E-37 | |
| CD8T | CD8T | ATP5PO | 0.43 | | 4.46E-37 | |
| CD8T | CD8T | NDUFB7 | 0.35 | | 7.13E-37 | |
| CD8T | CD8T | RTRAF | 0.4 | | 8.55E-37 | |
| CD8T | CD8T | ANXA5 | 0.29 | | 1.08E-36 | |
| CD8T | CD8T | CAPZA1 | 0.36 | | 1.14E-36 | |
| CD8T | CD8T | EWSR1 | 0.27 | | 1.16E-36 | |
| CD8T | CD8T | COA4 | 0.25 | | 1.62E-36 | |
| CD8T | CD8T | NAA20 | 0.26 | | 1.74E-36 | |
| CD8T | CD8T | MRPL3 | 0.25 | | 1.96E-36 | |
| CD8T | CD8T | H1FX | 0.29 | | 2.24E-36 | |
| CD8T | CD8T | YWHAB | 0.44 | | 2.70E-36 | |
| CD8T | CD8T | AURKAIP1 | 0.41 | | 2.71E-36 | |
| CD8T | CD8T | VAMP8 | 0.36 | | 4.20E-36 | |
| CD8T | CD8T | TNFRSF25 | 0.28 | | 7.26E-36 | |
| CD8T | CD8T | ENSA | 0.4 | | 7.70E-36 | |
| CD8T | CD8T | POLR2K | 0.36 | | 9.73E-36 | |
| CD8T | CD8T | RPS19BP1 | 0.31 | | 1.07E-35 | |
| CD8T | CD8T | KPNB1 | 0.27 | | 1.30E-35 | |
| CD8T | CD8T | ATP5F1D | 0.39 | | 1.59E-35 | |
| CD8T | CD8T | PSMB4 | 0.28 | | 2.40E-35 | |
| CD8T | CD8T | ADI1 | 0.28 | | 3.42E-35 | |
| CD8T | CD8T | MINOS1 | 0.42 | | 3.62E-35 | |
| CD8T | CD8T | BCAS4 | 0.34 | | 4.69E-35 | |
| CD8T | CD8T | AK6 | 0.27 | | 5.06E-35 | |
| CD8T | CD8T | EPSTI1 | 0.27 | | 6.04E-35 | |
| CD8T | CD8T | NDUFB4 | 0.44 | | 6.53E-35 | |
| CD8T | CD8T | DYNLRB1 | 0.31 | | 8.34E-35 | |
| CD8T | CD8T | COX6A1 | 0.42 | | 1.61E-34 | |
| CD8T | CD8T | RAB5IF | 0.3 | | 2.68E-34 | |
| CD8T | CD8T | PPP2R1A | 0.3 | | 2.90E-34 | |
| CD8T | CD8T | NDUFA6 | 0.34 | | 4.44E-34 | |
| CD8T | CD8T | NDUFC1 | 0.33 | | 6.51E-34 | |
| CD8T | CD8T | RER1 | 0.28 | | 7.66E-34 | |
| CD8T | CD8T | LRMP | 0.36 | | 7.75E-34 | |
| CD8T | CD8T | CIAO2B | 0.33 | | 8.56E-34 | |
| CD8T | CD8T | AP2M1 | 0.35 | | 8.67E-34 | |
| CD8T | CD8T | SNRPD2 | 0.4 | | 1.07E-33 | |
| CD8T | CD8T | RNPS1 | 0.32 | | 1.18E-33 | |
| CD8T | CD8T | TRAPPC2L | 0.3 | | 1.76E-33 | |
| CD8T | CD8T | CORO1B | 0.41 | | 1.78E-33 | |
| CD8T | CD8T | AES | 0.31 | | 2.07E-33 | |
| CD8T | CD8T | COPZ1 | 0.26 | | 2.69E-33 | |
| CD8T | CD8T | PAXX | 0.3 | | 5.20E-33 | |
| CD8T | CD8T | EIF4H | 0.3 | | 5.35E-33 | |
| CD8T | CD8T | CHMP2A | 0.31 | | 7.94E-33 | |
| CD8T | CD8T | PPP1R15A | -0.85 | | 8.17E-33 | |
| CD8T | CD8T | TIMM13 | 0.38 | | 8.20E-33 | |
| CD8T | CD8T | SRSF3 | 0.46 | | 8.31E-33 | |
| CD8T | CD8T | DDT | 0.37 | | 8.74E-33 | |
| CD8T | CD8T | NDUFB1 | 0.41 | | 9.43E-33 | |
| CD8T | CD8T | OCIAD2 | 0.3 | | 9.63E-33 | |
| CD8T | CD8T | NAP1L1 | 0.44 | | 1.55E-32 | |
| CD8T | CD8T | COX7B | 0.36 | | 1.56E-32 | |
| CD8T | CD8T | EIF3I | 0.36 | | 1.95E-32 | |
| CD8T | CD8T | MAGOH | 0.3 | | 2.24E-32 | |
| CD8T | CD8T | KLF6 | -0.74 | | 2.64E-32 | |
| CD8T | CD8T | HNRNPM | 0.36 | | 3.77E-32 | |
| CD8T | CD8T | PUF60 | 0.27 | | 4.02E-32 | |
| CD8T | CD8T | COPS9 | 0.35 | | 5.49E-32 | |
| CD8T | CD8T | COX7A2 | 0.37 | | 5.95E-32 | |
| CD8T | CD8T | COX16 | 0.29 | | 6.75E-32 | |
| CD8T | CD8T | PNRC1 | -0.83 | | 1.62E-31 | |
| CD8T | CD8T | TSC22D3 | -0.93 | | 2.02E-31 | |
| CD8T | CD8T | ROMO1 | 0.36 | | 2.36E-31 | |
| CD8T | CD8T | ARHGDIA | 0.35 | | 2.49E-31 | |
| CD8T | CD8T | DRAP1 | 0.31 | | 2.64E-31 | |
| CD8T | CD8T | PGK1 | 0.35 | | 2.65E-31 | |
| CD8T | CD8T | PSME1 | 0.43 | | 3.48E-31 | |
| CD8T | CD8T | MRPL57 | 0.29 | | 3.59E-31 | |
| CD8T | CD8T | RAD23A | 0.35 | | 5.81E-31 | |
| CD8T | CD8T | TMEM256 | 0.27 | | 6.26E-31 | |
[truncated: 496,267 more chars]
