# Supplementary material for: Development of a CD8+ T cell associated signature for predicting the prognosis and immunological characteristics of gastric cancer by integrating single-cell and bulk RNA-sequencing
Source: Sci Rep. 2024 Feb 24;14:4524. doi: 10.1038/s41598-024-54273-9 (PMC10894294; doi:10.1038/s41598-024-54273-9)
Supplement: Supplementary file 3 — Supplementary Table 2. [file 41598_2024_54273_MOESM3_ESM.docx]

| Supplementary Table 2. Differentially expressed genes of each cell cluster in GSE134520 | | | | |
| --- | --- | --- | --- | --- |
| Celltype (major-lineage) | Celltype (minor-lineage) | Gene | log2FC | Adjusted p-value |
| Pit mucous | Pit mucous | TFF1 | 1.2 | 0 |
| Pit mucous | Pit mucous | MUC5AC | 1.14 | 0 |
| Pit mucous | Pit mucous | MUCL3 | 1 | 0 |
| Pit mucous | Pit mucous | CLDN18 | 1 | 0 |
| Pit mucous | Pit mucous | GKN2 | 0.98 | 0 |
| Pit mucous | Pit mucous | S100P | 0.94 | 0 |
| Pit mucous | Pit mucous | KLF2 | 0.94 | 0 |
| Pit mucous | Pit mucous | CA2 | 0.94 | 0 |
| Pit mucous | Pit mucous | CYSTM1 | 0.89 | 0 |
| Pit mucous | Pit mucous | ALDH3A1 | 0.88 | 0 |
| Pit mucous | Pit mucous | GPX2 | 0.85 | 0 |
| Pit mucous | Pit mucous | SPINK1 | 0.83 | 0 |
| Pit mucous | Pit mucous | PDE4C | 0.8 | 0 |
| Pit mucous | Pit mucous | TAGLN2 | 0.76 | 0 |
| Pit mucous | Pit mucous | VSIG1 | 0.76 | 0 |
| Pit mucous | Pit mucous | FOXQ1 | 0.76 | 0 |
| Pit mucous | Pit mucous | MUC1 | 0.75 | 0 |
| Pit mucous | Pit mucous | AGR2 | 0.75 | 0 |
| Pit mucous | Pit mucous | PPDPF | 0.74 | 0 |
| Pit mucous | Pit mucous | AKR1C1 | 0.74 | 0 |
| Pit mucous | Pit mucous | C19orf33 | 0.73 | 0 |
| Pit mucous | Pit mucous | LAMB3 | 0.73 | 0 |
| Pit mucous | Pit mucous | CTSE | 0.72 | 0 |
| Pit mucous | Pit mucous | TACSTD2 | 0.71 | 0 |
| Pit mucous | Pit mucous | NQO1 | 0.7 | 0 |
| Pit mucous | Pit mucous | RNASE1 | 0.69 | 0 |
| Pit mucous | Pit mucous | TMSB4X | 0.68 | 0 |
| Pit mucous | Pit mucous | SMIM22 | 0.67 | 0 |
| Pit mucous | Pit mucous | AKR1C2 | 0.67 | 0 |
| Pit mucous | Pit mucous | SOSTDC1 | 0.66 | 0 |
| Pit mucous | Pit mucous | VSIG2 | 0.66 | 0 |
| Pit mucous | Pit mucous | KLF6 | 0.65 | 0 |
| Pit mucous | Pit mucous | IER3 | 0.63 | 0 |
| Pit mucous | Pit mucous | CRIP1 | 0.62 | 0 |
| Pit mucous | Pit mucous | S100A11 | 0.62 | 0 |
| Pit mucous | Pit mucous | TESC | 0.62 | 0 |
| Pit mucous | Pit mucous | SEC61G | 0.61 | 0 |
| Pit mucous | Pit mucous | JUND | 0.61 | 0 |
| Pit mucous | Pit mucous | AL138930.1 | 0.61 | 0 |
| Pit mucous | Pit mucous | STARD10 | 0.61 | 0 |
| Pit mucous | Pit mucous | AKR1B10 | 0.6 | 0 |
| Pit mucous | Pit mucous | TSPO | 0.58 | 0 |
| Pit mucous | Pit mucous | ANXA10 | 0.56 | 0 |
| Pit mucous | Pit mucous | SERF2 | 0.52 | 0 |
| Pit mucous | Pit mucous | EZR | 0.51 | 0 |
| Pit mucous | Pit mucous | GSN | 0.48 | 0 |
| Pit mucous | Pit mucous | GSTP1 | 0.46 | 0 |
| Pit mucous | Pit mucous | FXYD3 | 0.46 | 0 |
| Pit mucous | Pit mucous | TMSB10 | 0.44 | 0 |
| Pit mucous | Pit mucous | HSPB1 | 0.43 | 0 |
| Pit mucous | Pit mucous | ATP5F1E | 0.42 | 0 |
| Pit mucous | Pit mucous | TFF2 | 0.39 | 0 |
| Pit mucous | Pit mucous | IFI27 | 0.33 | 0 |
| Pit mucous | Pit mucous | S100A6 | 0.33 | 0 |
| Pit mucous | Pit mucous | RPL15 | -0.49 | 0 |
| Pit mucous | Pit mucous | LDHB | -0.58 | 0 |
| Pit mucous | Pit mucous | RPL6 | -0.58 | 0 |
| Pit mucous | Pit mucous | MALAT1 | -0.68 | 0 |
| Pit mucous | Pit mucous | HLA-C | -0.72 | 0 |
| Pit mucous | Pit mucous | IL32 | -0.91 | 0 |
| Pit mucous | Pit mucous | CLDN3 | -0.96 | 0 |
| Pit mucous | Pit mucous | CLDN7 | -1.02 | 0 |
| Pit mucous | Pit mucous | CD74 | -1.14 | 0 |
| Pit mucous | Pit mucous | PRAP1 | -1.25 | 0 |
| Pit mucous | Pit mucous | CLDN4 | -1.43 | 0 |
| Pit mucous | Pit mucous | TFF3 | -1.99 | 0 |
| Pit mucous | Pit mucous | REG4 | -2.2 | 0 |
| Pit mucous | Pit mucous | REG1A | -2.37 | 0 |
| Pit mucous | Pit mucous | FABP1 | -2.64 | 0 |
| Pit mucous | Pit mucous | CDH17 | -0.72 | 5.72E-305 |
| Pit mucous | Pit mucous | RPL7 | -0.74 | 1.16E-303 |
| Pit mucous | Pit mucous | BCAS1 | 0.66 | 1.36E-301 |
| Pit mucous | Pit mucous | ANPEP | -1.36 | 4.51E-299 |
| Pit mucous | Pit mucous | REP15 | 0.54 | 4.01E-293 |
| Pit mucous | Pit mucous | KRT19 | 0.29 | 6.33E-293 |
| Pit mucous | Pit mucous | OLFM4 | -1.58 | 1.05E-291 |
| Pit mucous | Pit mucous | ACTG1 | 0.3 | 1.05E-288 |
| Pit mucous | Pit mucous | RPL21 | -0.48 | 4.21E-288 |
| Pit mucous | Pit mucous | FABP2 | -1.42 | 9.84E-284 |
| Pit mucous | Pit mucous | MT1E | 0.64 | 1.33E-281 |
| Pit mucous | Pit mucous | RAB27B | 0.65 | 4.71E-281 |
| Pit mucous | Pit mucous | IDH2 | -0.49 | 1.79E-277 |
| Pit mucous | Pit mucous | CD44 | -0.54 | 3.72E-271 |
| Pit mucous | Pit mucous | MAL2 | 0.58 | 5.89E-270 |
| Pit mucous | Pit mucous | GAST | -1.47 | 4.83E-269 |
| Pit mucous | Pit mucous | PRDX5 | 0.46 | 6.21E-267 |
| Pit mucous | Pit mucous | ATP1B3 | -0.48 | 5.66E-263 |
| Pit mucous | Pit mucous | RAP2B | 0.66 | 8.25E-259 |
| Pit mucous | Pit mucous | RHOB | 0.59 | 7.37E-258 |
| Pit mucous | Pit mucous | PPP1R1B | -0.39 | 6.18E-257 |
| Pit mucous | Pit mucous | TM4SF4 | -0.87 | 6.24E-257 |
| Pit mucous | Pit mucous | PIGR | -0.89 | 1.36E-256 |
| Pit mucous | Pit mucous | RPS7 | -0.47 | 4.86E-255 |
| Pit mucous | Pit mucous | RAB11FIP1 | 0.58 | 1.62E-253 |
| Pit mucous | Pit mucous | SLC7A8 | 0.61 | 1.62E-253 |
| Pit mucous | Pit mucous | SQSTM1 | 0.54 | 1.32E-250 |
| Pit mucous | Pit mucous | RASEF | 0.65 | 1.34E-249 |
| Pit mucous | Pit mucous | LINC01133 | 0.52 | 9.74E-245 |
| Pit mucous | Pit mucous | SFN | 0.45 | 5.22E-239 |
| Pit mucous | Pit mucous | PLA2G10 | 0.62 | 3.98E-237 |
| Pit mucous | Pit mucous | RPLP2 | 0.27 | 1.22E-234 |
| Pit mucous | Pit mucous | CXCL17 | 0.45 | 1.63E-229 |
| Pit mucous | Pit mucous | TMEM176B | -0.62 | 2.45E-228 |
| Pit mucous | Pit mucous | SULT1C2 | 0.6 | 2.95E-222 |
| Pit mucous | Pit mucous | DMBT1 | -0.62 | 3.37E-221 |
| Pit mucous | Pit mucous | SMIM14 | 0.59 | 3.44E-220 |
| Pit mucous | Pit mucous | FAM3D | -0.41 | 3.94E-218 |
| Pit mucous | Pit mucous | ARPC1B | -0.46 | 9.51E-217 |
| Pit mucous | Pit mucous | ALDOB | -0.84 | 2.90E-213 |
| Pit mucous | Pit mucous | GLRX | -0.43 | 2.63E-212 |
| Pit mucous | Pit mucous | HSD17B11 | -0.36 | 1.02E-211 |
| Pit mucous | Pit mucous | GPA33 | -0.43 | 4.78E-211 |
| Pit mucous | Pit mucous | UGCG | 0.62 | 4.72E-209 |
| Pit mucous | Pit mucous | RGS2 | -0.54 | 1.64E-208 |
| Pit mucous | Pit mucous | EIF3L | -0.39 | 8.61E-208 |
| Pit mucous | Pit mucous | TMEM176A | -0.47 | 1.32E-206 |
| Pit mucous | Pit mucous | RPS4Y1 | -0.53 | 8.21E-205 |
| Pit mucous | Pit mucous | DEFB1 | 0.49 | 2.34E-204 |
| Pit mucous | Pit mucous | RPL5 | -0.45 | 5.22E-204 |
| Pit mucous | Pit mucous | B2M | -0.65 | 1.37E-203 |
| Pit mucous | Pit mucous | KRT20 | -0.85 | 1.74E-203 |
| Pit mucous | Pit mucous | AHNAK | 0.53 | 5.96E-202 |
| Pit mucous | Pit mucous | CIRBP | -0.47 | 4.90E-200 |
| Pit mucous | Pit mucous | SLC25A6 | -0.41 | 3.19E-199 |
| Pit mucous | Pit mucous | MALL | -0.61 | 4.35E-197 |
| Pit mucous | Pit mucous | CAPN8 | 0.57 | 2.72E-194 |
| Pit mucous | Pit mucous | FAM3C | -0.34 | 1.12E-193 |
| Pit mucous | Pit mucous | CLIC3 | 0.45 | 2.22E-191 |
| Pit mucous | Pit mucous | LDHA | -0.52 | 6.45E-188 |
| Pit mucous | Pit mucous | HSPA8 | -0.46 | 1.16E-186 |
| Pit mucous | Pit mucous | CCND1 | 0.59 | 5.54E-186 |
| Pit mucous | Pit mucous | CALM1 | -0.44 | 1.25E-185 |
| Pit mucous | Pit mucous | SELENOM | -0.49 | 1.41E-185 |
| Pit mucous | Pit mucous | AC023090.1 | 0.51 | 1.51E-185 |
| Pit mucous | Pit mucous | TM4SF20 | -0.66 | 1.57E-185 |
| Pit mucous | Pit mucous | PSAPL1 | 0.4 | 2.44E-185 |
| Pit mucous | Pit mucous | LY6E | -0.46 | 9.11E-185 |
| Pit mucous | Pit mucous | CSTB | 0.4 | 1.09E-184 |
| Pit mucous | Pit mucous | CLDN15 | -0.29 | 2.01E-184 |
| Pit mucous | Pit mucous | CAMK2N1 | -0.32 | 1.59E-183 |
| Pit mucous | Pit mucous | WFDC2 | -0.48 | 2.05E-183 |
| Pit mucous | Pit mucous | VIM | -1.22 | 6.93E-183 |
| Pit mucous | Pit mucous | SERPINA1 | -0.51 | 7.16E-183 |
| Pit mucous | Pit mucous | EPCAM | -0.6 | 9.51E-181 |
| Pit mucous | Pit mucous | ADM | -0.39 | 3.02E-178 |
| Pit mucous | Pit mucous | MT1G | 0.5 | 7.75E-178 |
| Pit mucous | Pit mucous | SMIM31 | -0.33 | 4.56E-177 |
| Pit mucous | Pit mucous | ADIRF | -0.81 | 1.55E-176 |
| Pit mucous | Pit mucous | APP | -0.29 | 7.49E-176 |
| Pit mucous | Pit mucous | BLVRB | 0.51 | 2.35E-174 |
| Pit mucous | Pit mucous | ETHE1 | -0.42 | 3.28E-174 |
| Pit mucous | Pit mucous | PGC | -1.25 | 3.79E-174 |
| Pit mucous | Pit mucous | RPL36AL | 0.31 | 4.87E-174 |
| Pit mucous | Pit mucous | SOD2 | -0.46 | 6.04E-174 |
| Pit mucous | Pit mucous | LRPAP1 | -0.26 | 1.98E-173 |
| Pit mucous | Pit mucous | RFLNA | 0.52 | 2.82E-173 |
| Pit mucous | Pit mucous | PCK1 | -0.54 | 1.49E-172 |
| Pit mucous | Pit mucous | F2RL1 | 0.59 | 1.62E-172 |
| Pit mucous | Pit mucous | ANXA13 | -0.46 | 1.43E-171 |
| Pit mucous | Pit mucous | CPS1 | -0.29 | 5.21E-171 |
| Pit mucous | Pit mucous | SYTL2 | 0.52 | 3.97E-169 |
| Pit mucous | Pit mucous | KLF4 | 0.48 | 4.79E-169 |
| Pit mucous | Pit mucous | SPCS1 | -0.38 | 1.05E-168 |
| Pit mucous | Pit mucous | MUC13 | -0.53 | 4.44E-168 |
| Pit mucous | Pit mucous | PMP22 | -0.47 | 9.15E-167 |
| Pit mucous | Pit mucous | HPGD | 0.43 | 2.40E-165 |
| Pit mucous | Pit mucous | SOX21 | 0.41 | 1.89E-163 |
| Pit mucous | Pit mucous | GAPDH | -0.42 | 3.61E-162 |
| Pit mucous | Pit mucous | ABLIM1 | 0.57 | 1.74E-161 |
| Pit mucous | Pit mucous | SUCLG1 | -0.29 | 1.93E-161 |
| Pit mucous | Pit mucous | FAM177B | 0.39 | 2.14E-161 |
| Pit mucous | Pit mucous | SELENOW | -0.34 | 2.48E-161 |
| Pit mucous | Pit mucous | CISD1 | -0.25 | 7.45E-161 |
| Pit mucous | Pit mucous | SI | -0.4 | 9.07E-161 |
| Pit mucous | Pit mucous | RBP2 | -1.47 | 5.69E-160 |
| Pit mucous | Pit mucous | UNC5B-AS1 | 0.38 | 1.13E-159 |
| Pit mucous | Pit mucous | PRDX4 | -0.32 | 1.41E-159 |
| Pit mucous | Pit mucous | HSPD1 | -0.39 | 1.88E-159 |
| Pit mucous | Pit mucous | AKR1C3 | 0.5 | 8.91E-159 |
| Pit mucous | Pit mucous | RAMP1 | -0.26 | 6.27E-158 |
| Pit mucous | Pit mucous | PRSS3 | -0.5 | 2.23E-157 |
| Pit mucous | Pit mucous | ASS1 | -0.25 | 1.28E-156 |
| Pit mucous | Pit mucous | MUC17 | -0.3 | 4.90E-156 |
| Pit mucous | Pit mucous | SERPINB6 | -0.36 | 8.75E-156 |
| Pit mucous | Pit mucous | NBEAL1 | 0.39 | 2.00E-155 |
| Pit mucous | Pit mucous | ATP5ME | 0.31 | 5.93E-155 |
| Pit mucous | Pit mucous | KHK | -0.35 | 6.08E-155 |
| Pit mucous | Pit mucous | UQCRC2 | -0.27 | 6.08E-155 |
| Pit mucous | Pit mucous | SPTSSB | 0.39 | 1.31E-154 |
| Pit mucous | Pit mucous | IFI6 | -0.4 | 1.90E-154 |
| Pit mucous | Pit mucous | ARF6 | 0.53 | 1.39E-151 |
| Pit mucous | Pit mucous | IFITM1 | -0.66 | 6.36E-150 |
| Pit mucous | Pit mucous | BST2 | -0.42 | 5.30E-149 |
| Pit mucous | Pit mucous | CYC1 | -0.35 | 5.82E-149 |
| Pit mucous | Pit mucous | LGALS1 | -1.24 | 1.26E-148 |
| Pit mucous | Pit mucous | C11orf86 | -0.59 | 1.38E-148 |
| Pit mucous | Pit mucous | ISG15 | -0.45 | 2.42E-148 |
| Pit mucous | Pit mucous | TRNP1 | 0.48 | 3.27E-148 |
| Pit mucous | Pit mucous | RPL23 | -0.35 | 3.72E-148 |
| Pit mucous | Pit mucous | ARL14 | 0.5 | 5.63E-148 |
| Pit mucous | Pit mucous | CCND2 | -0.25 | 7.84E-148 |
| Pit mucous | Pit mucous | SELENOP | -0.42 | 2.50E-147 |
| Pit mucous | Pit mucous | CKLF | -0.3 | 3.79E-147 |
| Pit mucous | Pit mucous | MT1X | 0.58 | 5.24E-146 |
| Pit mucous | Pit mucous | FUOM | -0.28 | 5.68E-146 |
| Pit mucous | Pit mucous | NKX6-2 | 0.39 | 9.58E-146 |
| Pit mucous | Pit mucous | HLA-DRA | -0.83 | 1.10E-145 |
| Pit mucous | Pit mucous | STMN1 | -0.48 | 3.42E-145 |
| Pit mucous | Pit mucous | UQCRB | -0.34 | 2.72E-144 |
| Pit mucous | Pit mucous | ELOB | 0.27 | 4.80E-144 |
| Pit mucous | Pit mucous | IFITM2 | -0.58 | 1.63E-143 |
| Pit mucous | Pit mucous | PSME2 | -0.37 | 1.00E-142 |
| Pit mucous | Pit mucous | SDC4 | 0.52 | 1.86E-142 |
| Pit mucous | Pit mucous | PKM | -0.34 | 2.64E-142 |
| Pit mucous | Pit mucous | CUTA | -0.27 | 3.41E-142 |
| Pit mucous | Pit mucous | RAC1 | 0.44 | 6.06E-142 |
| Pit mucous | Pit mucous | RPL10A | -0.35 | 1.60E-141 |
| Pit mucous | Pit mucous | ATP5F1A | -0.31 | 7.21E-141 |
| Pit mucous | Pit mucous | RHOA | 0.39 | 1.04E-140 |
| Pit mucous | Pit mucous | DPP7 | -0.26 | 1.29E-140 |
| Pit mucous | Pit mucous | CYBRD1 | -0.35 | 6.70E-140 |
| Pit mucous | Pit mucous | LMO4 | 0.51 | 6.95E-140 |
| Pit mucous | Pit mucous | TMPRSS15 | -0.55 | 1.85E-139 |
| Pit mucous | Pit mucous | EDN1 | -0.32 | 6.92E-139 |
| Pit mucous | Pit mucous | JUNB | -0.8 | 1.23E-138 |
| Pit mucous | Pit mucous | DECR1 | -0.26 | 2.80E-138 |
| Pit mucous | Pit mucous | ID2 | -0.41 | 2.83E-136 |
| Pit mucous | Pit mucous | RPL3 | -0.34 | 1.41E-135 |
| Pit mucous | Pit mucous | YWHAZ | 0.33 | 2.45E-135 |
| Pit mucous | Pit mucous | GCLC | 0.55 | 4.65E-135 |
| Pit mucous | Pit mucous | PYCARD | -0.26 | 7.18E-135 |
| Pit mucous | Pit mucous | UGDH | 0.56 | 7.39E-135 |
| Pit mucous | Pit mucous | MPC2 | -0.26 | 3.58E-134 |
| Pit mucous | Pit mucous | MT-CO2 | -0.6 | 6.85E-134 |
| Pit mucous | Pit mucous | EFHD2 | 0.51 | 2.30E-133 |
| Pit mucous | Pit mucous | SEC62 | -0.28 | 4.23E-133 |
| Pit mucous | Pit mucous | PRDX1 | 0.36 | 6.17E-133 |
| Pit mucous | Pit mucous | TUBA1A | -0.73 | 9.60E-133 |
| Pit mucous | Pit mucous | CAPN9 | 0.35 | 1.47E-132 |
| Pit mucous | Pit mucous | MTTP | -0.43 | 1.86E-132 |
| Pit mucous | Pit mucous | RPL4 | -0.35 | 3.50E-131 |
| Pit mucous | Pit mucous | GPX4 | -0.28 | 4.00E-131 |
| Pit mucous | Pit mucous | GADD45B | -0.64 | 2.16E-130 |
| Pit mucous | Pit mucous | CNN3 | -0.29 | 8.08E-130 |
| Pit mucous | Pit mucous | MT-ND2 | -0.26 | 2.47E-129 |
| Pit mucous | Pit mucous | CLIC1 | 0.3 | 5.73E-129 |
| Pit mucous | Pit mucous | PARD6B | 0.56 | 1.63E-128 |
| Pit mucous | Pit mucous | SEC11C | -0.38 | 3.77E-128 |
| Pit mucous | Pit mucous | TMEM258 | 0.34 | 3.86E-127 |
| Pit mucous | Pit mucous | JPT1 | 0.34 | 2.25E-126 |
| Pit mucous | Pit mucous | CTSC | -0.32 | 2.81E-126 |
| Pit mucous | Pit mucous | HSD17B2 | -0.33 | 1.27E-125 |
| Pit mucous | Pit mucous | NPC2 | -0.33 | 5.29E-125 |
| Pit mucous | Pit mucous | PRDX2 | -0.29 | 5.02E-123 |
| Pit mucous | Pit mucous | SRGN | -0.99 | 1.41E-122 |
| Pit mucous | Pit mucous | LMO7 | 0.42 | 2.23E-122 |
| Pit mucous | Pit mucous | FKBP11 | -0.47 | 8.75E-120 |
| Pit mucous | Pit mucous | SDCBP | 0.44 | 8.81E-120 |
| Pit mucous | Pit mucous | SRI | -0.3 | 1.54E-118 |
| Pit mucous | Pit mucous | LMNA | 0.25 | 3.70E-118 |
| Pit mucous | Pit mucous | NAP1L1 | -0.28 | 4.49E-118 |
| Pit mucous | Pit mucous | ELF3 | 0.34 | 1.38E-115 |
| Pit mucous | Pit mucous | CD9 | -0.41 | 4.15E-115 |
| Pit mucous | Pit mucous | ITM2C | -0.36 | 2.06E-114 |
| Pit mucous | Pit mucous | IL2RG | -0.3 | 2.10E-114 |
| Pit mucous | Pit mucous | MT-CYB | -0.35 | 2.81E-114 |
| Pit mucous | Pit mucous | CD320 | -0.59 | 6.03E-114 |
| Pit mucous | Pit mucous | HLA-DRB1 | -0.61 | 6.44E-114 |
| Pit mucous | Pit mucous | CDHR5 | -0.4 | 6.67E-114 |
| Pit mucous | Pit mucous | ID3 | -0.45 | 2.32E-113 |
| Pit mucous | Pit mucous | ANO1 | 0.38 | 2.69E-113 |
| Pit mucous | Pit mucous | IFITM3 | -0.58 | 6.63E-113 |
| Pit mucous | Pit mucous | EEF1B2 | -0.32 | 1.80E-112 |
| Pit mucous | Pit mucous | CXCL2 | -0.72 | 2.01E-112 |
| Pit mucous | Pit mucous | PPA1 | -0.29 | 3.86E-112 |
| Pit mucous | Pit mucous | CXCL1 | -0.5 | 1.07E-111 |
| Pit mucous | Pit mucous | HINT1 | -0.26 | 1.14E-110 |
| Pit mucous | Pit mucous | UBL3 | 0.48 | 2.25E-110 |
| Pit mucous | Pit mucous | TIMP1 | -0.45 | 3.34E-110 |
| Pit mucous | Pit mucous | RBM47 | 0.42 | 2.29E-109 |
| Pit mucous | Pit mucous | EIF3E | -0.34 | 2.86E-109 |
| Pit mucous | Pit mucous | EMP3 | -0.45 | 3.09E-109 |
| Pit mucous | Pit mucous | PEBP1 | -0.29 | 6.47E-109 |
| Pit mucous | Pit mucous | HSPE1 | -0.33 | 1.65E-108 |
| Pit mucous | Pit mucous | CES2 | -0.45 | 2.29E-108 |
| Pit mucous | Pit mucous | RANBP1 | -0.27 | 2.70E-107 |
| Pit mucous | Pit mucous | ARHGDIB | -0.47 | 6.38E-107 |
| Pit mucous | Pit mucous | SULT1A1 | -0.31 | 1.37E-106 |
| Pit mucous | Pit mucous | HLA-DPA1 | -0.55 | 1.61E-106 |
| Pit mucous | Pit mucous | DGAT1 | -0.38 | 1.80E-106 |
| Pit mucous | Pit mucous | DNAJB1 | -0.46 | 4.35E-106 |
| Pit mucous | Pit mucous | GALNT6 | 0.49 | 5.29E-106 |
| Pit mucous | Pit mucous | IL1RN | 0.48 | 7.69E-106 |
| Pit mucous | Pit mucous | SLC39A11 | 0.44 | 1.42E-104 |
| Pit mucous | Pit mucous | RPS27A | -0.28 | 4.18E-104 |
| Pit mucous | Pit mucous | ALDH1A1 | 0.43 | 1.28E-103 |
| Pit mucous | Pit mucous | SLPI | 0.34 | 2.96E-103 |
| Pit mucous | Pit mucous | SH3BGRL2 | 0.49 | 3.62E-103 |
| Pit mucous | Pit mucous | TUBB | -0.3 | 4.86E-103 |
| Pit mucous | Pit mucous | UQCR10 | 0.28 | 5.13E-102 |
| Pit mucous | Pit mucous | SPINK4 | -1.47 | 6.40E-101 |
| Pit mucous | Pit mucous | ARL6IP1 | 0.36 | 7.31E-101 |
| Pit mucous | Pit mucous | TAX1BP3 | 0.48 | 1.83E-100 |
| Pit mucous | Pit mucous | TPI1 | -0.28 | 2.03E-100 |
| Pit mucous | Pit mucous | UPK1B | 0.28 | 1.27E-98 |
| Pit mucous | Pit mucous | FAM107B | 0.47 | 5.36E-97 |
| Pit mucous | Pit mucous | HSPA5 | -0.33 | 5.90E-97 |
| Pit mucous | Pit mucous | HLA-DPB1 | -0.61 | 1.40E-96 |
| Pit mucous | Pit mucous | CREM | -0.51 | 1.57E-96 |
| Pit mucous | Pit mucous | ACSL5 | -0.28 | 1.54E-95 |
| Pit mucous | Pit mucous | PLAC8 | 0.31 | 4.65E-95 |
| Pit mucous | Pit mucous | SLC9A1 | 0.46 | 7.38E-95 |
| Pit mucous | Pit mucous | MS4A8 | -0.3 | 3.04E-93 |
| Pit mucous | Pit mucous | CLTB | 0.43 | 3.28E-92 |
| Pit mucous | Pit mucous | SLC7A11 | 0.42 | 7.65E-92 |
| Pit mucous | Pit mucous | PRR4 | -0.71 | 1.08E-91 |
| Pit mucous | Pit mucous | MDK | -0.48 | 1.52E-91 |
| Pit mucous | Pit mucous | POLR2L | 0.25 | 1.72E-91 |
| Pit mucous | Pit mucous | TES | 0.47 | 3.68E-90 |
| Pit mucous | Pit mucous | PAM | -0.27 | 1.27E-89 |
| Pit mucous | Pit mucous | MLPH | 0.45 | 7.18E-88 |
| Pit mucous | Pit mucous | RGS10 | -0.27 | 1.42E-87 |
| Pit mucous | Pit mucous | HLA-DRB5 | -0.28 | 6.79E-86 |
| Pit mucous | Pit mucous | FOXA3 | 0.45 | 2.37E-85 |
| Pit mucous | Pit mucous | KLF5 | 0.41 | 5.83E-85 |
| Pit mucous | Pit mucous | YOD1 | 0.47 | 1.14E-84 |
| Pit mucous | Pit mucous | FAM102A | 0.48 | 2.39E-84 |
| Pit mucous | Pit mucous | NR4A2 | -0.28 | 4.82E-84 |
| Pit mucous | Pit mucous | TUBA1B | -0.33 | 1.86E-83 |
| Pit mucous | Pit mucous | ID1 | -0.35 | 2.10E-83 |
| Pit mucous | Pit mucous | TPPP3 | -0.3 | 2.29E-83 |
| Pit mucous | Pit mucous | CEACAM6 | -0.31 | 3.50E-83 |
| Pit mucous | Pit mucous | MZT2B | 0.36 | 2.03E-82 |
| Pit mucous | Pit mucous | RAB27A | 0.39 | 8.63E-82 |
| Pit mucous | Pit mucous | P4HB | 0.3 | 3.03E-81 |
| Pit mucous | Pit mucous | COX8A | 0.25 | 5.59E-81 |
| Pit mucous | Pit mucous | PHLDA2 | 0.27 | 9.05E-81 |
| Pit mucous | Pit mucous | DYNLL1 | 0.28 | 9.95E-81 |
| Pit mucous | Pit mucous | FAM120A | 0.45 | 4.46E-80 |
| Pit mucous | Pit mucous | VAMP8 | 0.29 | 4.79E-80 |
| Pit mucous | Pit mucous | AKR7A3 | 0.36 | 9.32E-80 |
| Pit mucous | Pit mucous | TSPAN1 | 0.34 | 4.44E-79 |
| Pit mucous | Pit mucous | ATP5PF | -0.25 | 6.27E-79 |
| Pit mucous | Pit mucous | S100A4 | -0.78 | 1.10E-78 |
| Pit mucous | Pit mucous | PLPP1 | -0.26 | 3.07E-78 |
| Pit mucous | Pit mucous | EPHA2 | 0.44 | 3.88E-78 |
| Pit mucous | Pit mucous | VAMP5 | -0.27 | 3.89E-78 |
| Pit mucous | Pit mucous | CYR61 | -0.28 | 6.23E-78 |
| Pit mucous | Pit mucous | CLU | -0.49 | 1.57E-77 |
| Pit mucous | Pit mucous | LGALS9B | 0.25 | 3.38E-77 |
| Pit mucous | Pit mucous | OTUD1 | 0.39 | 7.29E-77 |
| Pit mucous | Pit mucous | ANO7 | 0.27 | 2.29E-76 |
| Pit mucous | Pit mucous | ACTN4 | 0.36 | 5.26E-76 |
| Pit mucous | Pit mucous | RPS2 | -0.34 | 7.87E-75 |
| Pit mucous | Pit mucous | MUC6 | -1.07 | 1.25E-74 |
| Pit mucous | Pit mucous | DEPP1 | -0.28 | 2.56E-74 |
| Pit mucous | Pit mucous | ANKRD28 | -0.39 | 2.76E-74 |
| Pit mucous | Pit mucous | HLA-DQB1 | -0.28 | 2.77E-74 |
| Pit mucous | Pit mucous | PLXNB2 | 0.46 | 4.08E-74 |
| Pit mucous | Pit mucous | CHP1 | 0.44 | 5.61E-74 |
| Pit mucous | Pit mucous | SMIM6 | 0.4 | 2.79E-73 |
| Pit mucous | Pit mucous | SLC44A1 | 0.45 | 2.88E-73 |
| Pit mucous | Pit mucous | CA9 | 0.4 | 4.28E-73 |
| Pit mucous | Pit mucous | LGALS9 | 0.44 | 7.25E-73 |
| Pit mucous | Pit mucous | KDELR2 | 0.36 | 1.04E-72 |
| Pit mucous | Pit mucous | PCLAF | -0.27 | 1.20E-72 |
| Pit mucous | Pit mucous | HLA-E | -0.25 | 1.99E-72 |
| Pit mucous | Pit mucous | SCP2 | 0.31 | 2.04E-72 |
| Pit mucous | Pit mucous | H3F3B | -0.31 | 3.65E-71 |
| Pit mucous | Pit mucous | HSP90AA1 | -0.33 | 5.76E-71 |
| Pit mucous | Pit mucous | AAMDC | 0.47 | 1.49E-70 |
| Pit mucous | Pit mucous | SDCBP2 | 0.26 | 2.09E-70 |
| Pit mucous | Pit mucous | OST4 | 0.26 | 9.17E-70 |
| Pit mucous | Pit mucous | IL1R2 | 0.35 | 1.11E-69 |
| Pit mucous | Pit mucous | ATP8B1 | 0.45 | 2.52E-69 |
| Pit mucous | Pit mucous | RGS1 | -0.84 | 3.41E-69 |
| Pit mucous | Pit mucous | ZBTB43 | 0.46 | 7.27E-67 |
| Pit mucous | Pit mucous | JAG1 | 0.42 | 1.18E-66 |
| Pit mucous | Pit mucous | ABHD2 | 0.43 | 2.82E-66 |
| Pit mucous | Pit mucous | NOP10 | 0.35 | 3.51E-66 |
| Pit mucous | Pit mucous | TMEM134 | 0.44 | 4.31E-66 |
| Pit mucous | Pit mucous | ANKRD36C | 0.45 | 1.04E-65 |
| Pit mucous | Pit mucous | FTL | -0.31 | 2.24E-65 |
| Pit mucous | Pit mucous | SFTA2 | 0.29 | 4.39E-65 |
| Pit mucous | Pit mucous | ETNK1 | 0.42 | 3.33E-63 |
| Pit mucous | Pit mucous | B4GALT1 | 0.41 | 4.82E-63 |
| Pit mucous | Pit mucous | ARPC1A | 0.39 | 2.06E-62 |
| Pit mucous | Pit mucous | GALE | 0.42 | 4.92E-62 |
| Pit mucous | Pit mucous | NPM1 | -0.27 | 5.11E-62 |
| Pit mucous | Pit mucous | PCBP1 | 0.37 | 6.60E-62 |
| Pit mucous | Pit mucous | LASP1 | 0.42 | 9.56E-62 |
| Pit mucous | Pit mucous | SLC44A4 | 0.4 | 1.01E-61 |
| Pit mucous | Pit mucous | HLA-B | -0.35 | 7.34E-61 |
| Pit mucous | Pit mucous | RPS3A | -0.26 | 3.39E-60 |
| Pit mucous | Pit mucous | DSP | 0.41 | 2.02E-59 |
| Pit mucous | Pit mucous | RPL17 | -0.26 | 4.62E-59 |
| Pit mucous | Pit mucous | NET1 | 0.43 | 4.65E-59 |
| Pit mucous | Pit mucous | SGSM3 | 0.41 | 5.90E-59 |
| Pit mucous | Pit mucous | MT2A | 0.41 | 6.10E-59 |
| Pit mucous | Pit mucous | SLC38A2 | 0.38 | 2.56E-58 |
| Pit mucous | Pit mucous | TUBA1C | 0.37 | 4.99E-58 |
| Pit mucous | Pit mucous | TM9SF3 | 0.36 | 3.95E-57 |
| Pit mucous | Pit mucous | SVIP | 0.4 | 8.12E-57 |
| Pit mucous | Pit mucous | ATF4 | 0.35 | 1.53E-56 |
| Pit mucous | Pit mucous | TSC22D3 | -0.67 | 2.06E-56 |
| Pit mucous | Pit mucous | SLC44A2 | 0.35 | 4.31E-56 |
| Pit mucous | Pit mucous | ERO1B | -0.28 | 9.72E-56 |
| Pit mucous | Pit mucous | LYZ | -0.71 | 1.79E-55 |
| Pit mucous | Pit mucous | SGMS2 | 0.46 | 1.86E-55 |
| Pit mucous | Pit mucous | ZBTB7C | 0.28 | 1.03E-54 |
| Pit mucous | Pit mucous | YWHAH | 0.4 | 2.68E-54 |
| Pit mucous | Pit mucous | CYP2S1 | 0.4 | 4.23E-54 |
| Pit mucous | Pit mucous | MRPL52 | 0.37 | 5.86E-54 |
| Pit mucous | Pit mucous | OCLN | 0.43 | 5.96E-54 |
| Pit mucous | Pit mucous | IFNGR2 | 0.41 | 6.63E-53 |
| Pit mucous | Pit mucous | FRMD4B | 0.41 | 1.49E-52 |
| Pit mucous | Pit mucous | LDLR | 0.36 | 1.05E-50 |
| Pit mucous | Pit mucous | MT-ATP6 | -0.35 | 1.49E-49 |
| Pit mucous | Pit mucous | S100A10 | -0.36 | 2.80E-49 |
| Pit mucous | Pit mucous | AKAP13 | 0.4 | 3.14E-49 |
| Pit mucous | Pit mucous | SCIN | 0.28 | 7.56E-49 |
| Pit mucous | Pit mucous | RNF223 | 0.28 | 1.12E-48 |
| Pit mucous | Pit mucous | DUSP1 | 0.27 | 1.77E-48 |
| Pit mucous | Pit mucous | BRI3 | 0.41 | 2.19E-48 |
| Pit mucous | Pit mucous | CTNNA1 | 0.36 | 7.84E-48 |
| Pit mucous | Pit mucous | CTNND1 | 0.39 | 9.22E-48 |
| Pit mucous | Pit mucous | B4GALNT3 | 0.29 | 3.69E-47 |
| Pit mucous | Pit mucous | EPS8 | 0.38 | 4.51E-47 |
| Pit mucous | Pit mucous | AGPAT2 | -0.43 | 5.42E-47 |
| Pit mucous | Pit mucous | FAM241A | 0.26 | 2.89E-46 |
| Pit mucous | Pit mucous | EPS8L1 | 0.35 | 1.22E-45 |
| Pit mucous | Pit mucous | BPIFB1 | -0.67 | 1.30E-45 |
| Pit mucous | Pit mucous | JUP | 0.36 | 5.33E-45 |
| Pit mucous | Pit mucous | LIPF | -1.55 | 8.42E-45 |
| Pit mucous | Pit mucous | ERBIN | 0.32 | 1.76E-44 |
| Pit mucous | Pit mucous | TENT5A | 0.38 | 1.01E-43 |
| Pit mucous | Pit mucous | ITPKC | 0.38 | 1.20E-43 |
| Pit mucous | Pit mucous | ME1 | 0.3 | 2.34E-43 |
| Pit mucous | Pit mucous | CTTN | 0.38 | 3.92E-43 |
| Pit mucous | Pit mucous | ZFP36 | -0.42 | 4.92E-42 |
| Pit mucous | Pit mucous | TCEAL9 | 0.34 | 3.44E-41 |
| Pit mucous | Pit mucous | ATP2A3 | 0.3 | 3.92E-41 |
| Pit mucous | Pit mucous | SIK1 | 0.47 | 9.13E-41 |
| Pit mucous | Pit mucous | CYBA | -0.31 | 2.34E-40 |
| Pit mucous | Pit mucous | PCSK1N | -0.88 | 2.70E-40 |
| Pit mucous | Pit mucous | DDX17 | 0.37 | 1.15E-39 |
| Pit mucous | Pit mucous | TXNRD1 | 0.4 | 4.79E-39 |
| Pit mucous | Pit mucous | BLOC1S1 | 0.26 | 9.86E-39 |
| Pit mucous | Pit mucous | COTL1 | 0.35 | 1.05E-38 |
| Pit mucous | Pit mucous | TMEM38A | 0.27 | 3.16E-38 |
| Pit mucous | Pit mucous | HMGA1 | 0.31 | 1.03E-37 |
| Pit mucous | Pit mucous | NFKB1 | 0.37 | 1.29E-37 |
| Pit mucous | Pit mucous | RASSF6 | 0.35 | 5.49E-37 |
| Pit mucous | Pit mucous | HMGCS1 | 0.36 | 3.50E-36 |
| Pit mucous | Pit mucous | GAREM1 | 0.3 | 4.91E-36 |
| Pit mucous | Pit mucous | ATF3 | 0.33 | 1.21E-35 |
| Pit mucous | Pit mucous | SRCAP | 0.39 | 2.39E-35 |
| Pit mucous | Pit mucous | SFPQ | 0.34 | 7.71E-35 |
| Pit mucous | Pit mucous | ABCC5 | 0.31 | 8.20E-35 |
| Pit mucous | Pit mucous | ARID3B | 0.33 | 2.18E-34 |
| Pit mucous | Pit mucous | INF2 | 0.37 | 3.32E-34 |
| Pit mucous | Pit mucous | KCNK1 | 0.4 | 4.87E-34 |
| Pit mucous | Pit mucous | EFNB2 | 0.36 | 2.15E-33 |
| Pit mucous | Pit mucous | SST | -1.81 | 3.79E-33 |
| Pit mucous | Pit mucous | CDC42 | 0.25 | 2.10E-32 |
| Pit mucous | Pit mucous | OASL | 0.27 | 2.74E-32 |
| Pit mucous | Pit mucous | PPP1CB | 0.32 | 3.30E-32 |
| Pit mucous | Pit mucous | CDH1 | 0.32 | 1.15E-31 |
| Pit mucous | Pit mucous | FOSB | 0.25 | 1.27E-31 |
| Pit mucous | Pit mucous | B3GNT5 | 0.35 | 1.50E-31 |
| Pit mucous | Pit mucous | SH3RF1 | 0.36 | 1.83E-31 |
| Pit mucous | Pit mucous | COX17 | 0.27 | 2.56E-31 |
| Pit mucous | Pit mucous | IL17RE | 0.27 | 3.06E-31 |
| Pit mucous | Pit mucous | TMEM246 | 0.26 | 3.96E-31 |
| Pit mucous | Pit mucous | METTL7A | 0.33 | 1.38E-30 |
| Pit mucous | Pit mucous | HIST1H4C | -0.31 | 2.82E-30 |
| Pit mucous | Pit mucous | TMEM30B | 0.37 | 8.02E-30 |
| Pit mucous | Pit mucous | SSBP3 | 0.34 | 6.61E-29 |
| Pit mucous | Pit mucous | LSM7 | 0.27 | 1.28E-28 |
| Pit mucous | Pit mucous | PIM1 | 0.37 | 1.67E-28 |
| Pit mucous | Pit mucous | RARRES2 | -0.38 | 3.96E-28 |
| Pit mucous | Pit mucous | C3orf52 | 0.31 | 5.86E-28 |
| Pit mucous | Pit mucous | PTP4A2 | 0.33 | 9.54E-28 |
| Pit mucous | Pit mucous | JCHAIN | -2.53 | 1.01E-27 |
| Pit mucous | Pit mucous | SQLE | 0.33 | 1.09E-27 |
| Pit mucous | Pit mucous | LGALS3 | -0.41 | 2.93E-27 |
| Pit mucous | Pit mucous | FAR1 | 0.32 | 1.75E-26 |
| Pit mucous | Pit mucous | RAB11A | 0.29 | 2.86E-26 |
| Pit mucous | Pit mucous | MSMO1 | 0.34 | 4.08E-26 |
| Pit mucous | Pit mucous | CCDC88B | 0.34 | 4.13E-26 |
| Pit mucous | Pit mucous | CAPG | 0.29 | 9.26E-26 |
| Pit mucous | Pit mucous | B3GNT7 | 0.25 | 1.30E-25 |
| Pit mucous | Pit mucous | TLNRD1 | 0.28 | 1.89E-25 |
| Pit mucous | Pit mucous | PDIA4 | 0.32 | 5.38E-25 |
| Pit mucous | Pit mucous | TOB1 | 0.34 | 8.14E-25 |
| Pit mucous | Pit mucous | INO80C | 0.37 | 9.97E-25 |
| Pit mucous | Pit mucous | GALNT7 | 0.33 | 3.51E-24 |
| Pit mucous | Pit mucous | PET100 | 0.29 | 3.70E-24 |
| Pit mucous | Pit mucous | ABHD17C | 0.35 | 9.24E-24 |
| Pit mucous | Pit mucous | SUCLG2 | 0.35 | 2.76E-23 |
| Pit mucous | Pit mucous | EDEM3 | 0.29 | 3.81E-23 |
| Pit mucous | Pit mucous | CXCL3 | -0.45 | 3.89E-23 |
| Pit mucous | Pit mucous | CALR | 0.25 | 4.13E-23 |
| Pit mucous | Pit mucous | VAPA | 0.28 | 8.77E-23 |
| Pit mucous | Pit mucous | LGMN | 0.33 | 1.06E-22 |
| Pit mucous | Pit mucous | HSPA1A | -0.34 | 1.45E-22 |
| Pit mucous | Pit mucous | ALDH3A2 | 0.34 | 3.43E-22 |
| Pit mucous | Pit mucous | DHCR24 | 0.3 | 5.24E-22 |
| Pit mucous | Pit mucous | ARHGAP21 | 0.29 | 5.61E-22 |
| Pit mucous | Pit mucous | LPCAT4 | 0.29 | 7.09E-22 |
| Pit mucous | Pit mucous | PKP3 | 0.3 | 1.56E-21 |
| Pit mucous | Pit mucous | DAZAP2 | 0.29 | 1.75E-21 |
| Pit mucous | Pit mucous | BACE2 | 0.31 | 2.50E-21 |
| Pit mucous | Pit mucous | BCAR1 | 0.33 | 5.38E-21 |
| Pit mucous | Pit mucous | ANG | 0.32 | 5.89E-21 |
| Pit mucous | Pit mucous | GATA6 | 0.3 | 2.65E-20 |
| Pit mucous | Pit mucous | WEE1 | 0.34 | 2.95E-20 |
| Pit mucous | Pit mucous | LAMA3 | 0.32 | 3.69E-20 |
| Pit mucous | Pit mucous | GNAQ | 0.31 | 4.30E-20 |
| Pit mucous | Pit mucous | CYP3A5 | 0.26 | 4.38E-20 |
| Pit mucous | Pit mucous | MT-CO1 | -0.28 | 4.54E-20 |
| Pit mucous | Pit mucous | ACBD3 | 0.29 | 8.30E-20 |
| Pit mucous | Pit mucous | EIF4G2 | 0.26 | 1.25E-19 |
| Pit mucous | Pit mucous | LRRFIP1 | 0.28 | 1.54E-19 |
| Pit mucous | Pit mucous | MRPL23 | 0.26 | 3.09E-19 |
| Pit mucous | Pit mucous | DNMBP | 0.29 | 3.20E-19 |
| Pit mucous | Pit mucous | ADD3 | 0.32 | 5.48E-19 |
| Pit mucous | Pit mucous | CMBL | 0.3 | 8.04E-19 |
| Pit mucous | Pit mucous | LIPH | 0.35 | 1.10E-18 |
| Pit mucous | Pit mucous | NEDD9 | 0.34 | 1.66E-18 |
| Pit mucous | Pit mucous | TNKS1BP1 | 0.26 | 2.49E-18 |
| Pit mucous | Pit mucous | TCF7L2 | 0.33 | 1.04E-17 |
| Pit mucous | Pit mucous | QSOX1 | 0.27 | 1.49E-17 |
| Pit mucous | Pit mucous | ARHGAP12 | 0.3 | 3.15E-17 |
| Pit mucous | Pit mucous | NDRG1 | 0.3 | 3.29E-17 |
| Pit mucous | Pit mucous | FA2H | 0.26 | 3.96E-17 |
| Pit mucous | Pit mucous | DGKD | 0.25 | 5.80E-17 |
| Pit mucous | Pit mucous | EIF1AX | 0.28 | 6.25E-17 |
| Pit mucous | Pit mucous | MRPL14 | 0.29 | 1.41E-16 |
| Pit mucous | Pit mucous | BAG1 | 0.31 | 1.71E-16 |
| Pit mucous | Pit mucous | SMAD3 | 0.27 | 1.71E-16 |
| Pit mucous | Pit mucous | TMEM63B | 0.27 | 4.28E-16 |
| Pit mucous | Pit mucous | HERPUD1 | -0.32 | 4.59E-16 |
| Pit mucous | Pit mucous | RIOK3 | 0.3 | 4.92E-16 |
| Pit mucous | Pit mucous | FAM84B | 0.3 | 8.89E-16 |
| Pit mucous | Pit mucous | TMBIM1 | 0.29 | 1.14E-14 |
| Pit mucous | Pit mucous | KLF3 | 0.32 | 1.33E-14 |
| Pit mucous | Pit mucous | ITPR3 | 0.25 | 4.40E-14 |
| Pit mucous | Pit mucous | IGLL5 | -2.45 | 5.90E-14 |
| Pit mucous | Pit mucous | GNB2 | 0.29 | 6.53E-14 |
| Pit mucous | Pit mucous | MIR22HG | 0.3 | 7.45E-14 |
| Pit mucous | Pit mucous | BAIAP2L1 | 0.3 | 8.23E-14 |
| Pit mucous | Pit mucous | CKAP4 | 0.28 | 8.76E-14 |
| Pit mucous | Pit mucous | DR1 | 0.26 | 1.58E-13 |
| Pit mucous | Pit mucous | CYP2C18 | 0.25 | 1.87E-13 |
| Pit mucous | Pit mucous | INAVA | 0.3 | 2.93E-13 |
| Pit mucous | Pit mucous | VPS37B | 0.31 | 6.85E-13 |
| Pit mucous | Pit mucous | TICAM1 | 0.29 | 7.30E-13 |
| Pit mucous | Pit mucous | GFPT1 | 0.3 | 9.52E-13 |
| Pit mucous | Pit mucous | GALNT3 | 0.3 | 1.50E-12 |
| Pit mucous | Pit mucous | TST | 0.29 | 1.52E-12 |
| Pit mucous | Pit mucous | TNFRSF21 | 0.29 | 1.94E-12 |
| Pit mucous | Pit mucous | BHLHE40 | 0.27 | 2.95E-12 |
| Pit mucous | Pit mucous | MXD1 | 0.27 | 4.07E-12 |
| Pit mucous | Pit mucous | MAPK3 | 0.27 | 4.53E-12 |
| Pit mucous | Pit mucous | STK24 | 0.29 | 6.60E-12 |
| Pit mucous | Pit mucous | ITGAV | 0.27 | 7.12E-12 |
| Pit mucous | Pit mucous | RAB5B | 0.26 | 3.50E-11 |
| Pit mucous | Pit mucous | RNF103 | 0.25 | 4.36E-11 |
| Pit mucous | Pit mucous | KLHL21 | 0.27 | 6.90E-11 |
| Pit mucous | Pit mucous | PLLP | 0.27 | 1.61E-10 |
| Pit mucous | Pit mucous | NR2F6 | 0.26 | 2.07E-10 |
| Pit mucous | Pit mucous | MCU | 0.26 | 2.22E-10 |
| Pit mucous | Pit mucous | TSC22D2 | 0.28 | 8.17E-10 |
| Pit mucous | Pit mucous | SUN1 | 0.28 | 9.80E-10 |
| Pit mucous | Pit mucous | EIF4EBP2 | 0.28 | 1.02E-09 |
| Pit mucous | Pit mucous | MAFF | 0.25 | 2.13E-09 |
| Pit mucous | Pit mucous | TLE4 | 0.27 | 2.99E-09 |
| Pit mucous | Pit mucous | MAP2K3 | 0.28 | 4.48E-09 |
| Pit mucous | Pit mucous | PTTG1IP | 0.29 | 7.10E-09 |
| Pit mucous | Pit mucous | SPINT1 | 0.27 | 9.64E-09 |
| Pit mucous | Pit mucous | NFE2L2 | 0.25 | 1.30E-08 |
| Pit mucous | Pit mucous | HDLBP | 0.28 | 2.00E-08 |
| Pit mucous | Pit mucous | EFCAB14 | 0.27 | 8.60E-08 |
| Pit mucous | Pit mucous | HACD3 | 0.27 | 9.71E-08 |
| Pit mucous | Pit mucous | NEDD4L | 0.29 | 2.02E-07 |
| Pit mucous | Pit mucous | IMPA2 | 0.27 | 4.42E-07 |
| Pit mucous | Pit mucous | CREB3L1 | 0.25 | 4.51E-07 |
| Pit mucous | Pit mucous | TMPRSS2 | 0.27 | 1.01E-06 |
| Pit mucous | Pit mucous | RDH10 | 0.26 | 1.44E-06 |
| Pit mucous | Pit mucous | TNIP1 | 0.28 | 1.77E-06 |
| Pit mucous | Pit mucous | CHD2 | 0.27 | 1.80E-06 |
| Pit mucous | Pit mucous | PHGR1 | -0.98 | 2.77E-06 |
| Pit mucous | Pit mucous | MAPK6 | 0.28 | 2.86E-06 |
| Pit mucous | Pit mucous | B4GALT5 | 0.27 | 3.16E-06 |
| Pit mucous | Pit mucous | HDAC1 | 0.26 | 3.77E-06 |
| Pit mucous | Pit mucous | TOMM5 | 0.26 | 5.31E-06 |
| Pit mucous | Pit mucous | VPS35 | 0.27 | 6.53E-06 |
| Pit mucous | Pit mucous | TJP1 | 0.27 | 6.72E-06 |
| Pit mucous | Pit mucous | GKN1 | 3.57 | 0 |
| Pit mucous | Pit mucous | GKN2 | 2.62 | 0 |
| Pit mucous | Pit mucous | PSCA | 2.47 | 0 |
| Pit mucous | Pit mucous | TFF1 | 2.03 | 0 |
| Pit mucous | Pit mucous | MUCL3 | 1.83 | 0 |
| Pit mucous | Pit mucous | MUC5AC | 1.78 | 0 |
| Pit mucous | Pit mucous | TFF2 | 1.62 | 0 |
| Pit mucous | Pit mucous | S100P | 1.52 | 0 |
| Pit mucous | Pit mucous | RNASE1 | 1.49 | 0 |
| Pit mucous | Pit mucous | PSAPL1 | 1.47 | 0 |
| Pit mucous | Pit mucous | MUC1 | 1.46 | 0 |
| Pit mucous | Pit mucous | MT1G | 1.34 | 0 |
| Pit mucous | Pit mucous | LINC01133 | 1.33 | 0 |
| Pit mucous | Pit mucous | CA2 | 1.31 | 0 |
| Pit mucous | Pit mucous | GSN | 1.13 | 0 |
| Pit mucous | Pit mucous | TESC | 1.09 | 0 |
| Pit mucous | Pit mucous | SMIM22 | 1.09 | 0 |
| Pit mucous | Pit mucous | IFI27 | 1.08 | 0 |
| Pit mucous | Pit mucous | CEACAM5 | 1.04 | 0 |
| Pit mucous | Pit mucous | CAPN8 | 1.04 | 0 |
| Pit mucous | Pit mucous | VILL | 1.03 | 0 |
| Pit mucous | Pit mucous | TAGLN2 | 1 | 0 |
| Pit mucous | Pit mucous | UNC5B-AS1 | 1 | 0 |
| Pit mucous | Pit mucous | C19orf33 | 0.99 | 0 |
| Pit mucous | Pit mucous | AKR1C1 | 0.99 | 0 |
| Pit mucous | Pit mucous | CLDN18 | 0.99 | 0 |
| Pit mucous | Pit mucous | VSIG2 | 0.98 | 0 |
| Pit mucous | Pit mucous | BCAS1 | 0.96 | 0 |
| Pit mucous | Pit mucous | PDE4C | 0.96 | 0 |
| Pit mucous | Pit mucous | SLPI | 0.95 | 0 |
| Pit mucous | Pit mucous | DEFB1 | 0.95 | 0 |
| Pit mucous | Pit mucous | IGFBP4 | 0.94 | 0 |
| Pit mucous | Pit mucous | LAMB3 | 0.93 | 0 |
| Pit mucous | Pit mucous | HRASLS2 | 0.92 | 0 |
| Pit mucous | Pit mucous | CYP2S1 | 0.92 | 0 |
| Pit mucous | Pit mucous | SLC5A5 | 0.91 | 0 |
| Pit mucous | Pit mucous | CTSE | 0.9 | 0 |
| Pit mucous | Pit mucous | KLF2 | 0.9 | 0 |
| Pit mucous | Pit mucous | PLAC8 | 0.89 | 0 |
| Pit mucous | Pit mucous | VSIG1 | 0.88 | 0 |
| Pit mucous | Pit mucous | SOSTDC1 | 0.88 | 0 |
| Pit mucous | Pit mucous | AKR1C2 | 0.87 | 0 |
| Pit mucous | Pit mucous | CLTB | 0.87 | 0 |
| Pit mucous | Pit mucous | CSTA | 0.87 | 0 |
| Pit mucous | Pit mucous | RASEF | 0.86 | 0 |
| Pit mucous | Pit mucous | SLC7A8 | 0.86 | 0 |
| Pit mucous | Pit mucous | B4GALNT3 | 0.85 | 0 |
| Pit mucous | Pit mucous | CRIP1 | 0.83 | 0 |
| Pit mucous | Pit mucous | S100A6 | 0.82 | 0 |
| Pit mucous | Pit mucous | EFHD2 | 0.82 | 0 |
| Pit mucous | Pit mucous | HPGD | 0.82 | 0 |
| Pit mucous | Pit mucous | OASL | 0.82 | 0 |
| Pit mucous | Pit mucous | SPINK1 | 0.81 | 0 |
| Pit mucous | Pit mucous | LGALS9B | 0.78 | 0 |
| Pit mucous | Pit mucous | LGALS9C | 0.76 | 0 |
| Pit mucous | Pit mucous | JUND | 0.76 | 0 |
| Pit mucous | Pit mucous | TACSTD2 | 0.74 | 0 |
| Pit mucous | Pit mucous | FXYD3 | 0.73 | 0 |
| Pit mucous | Pit mucous | AKR1B10 | 0.73 | 0 |
| Pit mucous | Pit mucous | CYSTM1 | 0.73 | 0 |
| Pit mucous | Pit mucous | TMSB4X | 0.72 | 0 |
| Pit mucous | Pit mucous | PHGR1 | 0.71 | 0 |
| Pit mucous | Pit mucous | GPX2 | 0.71 | 0 |
| Pit mucous | Pit mucous | SPTSSB | 0.69 | 0 |
| Pit mucous | Pit mucous | PKIB | 0.68 | 0 |
| Pit mucous | Pit mucous | NKX6-2 | 0.66 | 0 |
| Pit mucous | Pit mucous | MSMB | 0.62 | 0 |
| Pit mucous | Pit mucous | PPDPF | 0.62 | 0 |
| Pit mucous | Pit mucous | AGR2 | 0.61 | 0 |
| Pit mucous | Pit mucous | SERF2 | 0.6 | 0 |
| Pit mucous | Pit mucous | S100A11 | 0.56 | 0 |
| Pit mucous | Pit mucous | MAL | 0.55 | 0 |
| Pit mucous | Pit mucous | ATP5F1E | 0.48 | 0 |
| Pit mucous | Pit mucous | RPL41 | -0.45 | 0 |
| Pit mucous | Pit mucous | RPL10 | -0.52 | 0 |
| Pit mucous | Pit mucous | RPL28 | -0.52 | 0 |
| Pit mucous | Pit mucous | RPLP1 | -0.53 | 0 |
| Pit mucous | Pit mucous | RPS28 | -0.54 | 0 |
| Pit mucous | Pit mucous | RPL9 | -0.54 | 0 |
| Pit mucous | Pit mucous | RPL35 | -0.54 | 0 |
| Pit mucous | Pit mucous | RPL18A | -0.55 | 0 |
| Pit mucous | Pit mucous | EEF1D | -0.57 | 0 |
| Pit mucous | Pit mucous | RPLP0 | -0.59 | 0 |
| Pit mucous | Pit mucous | RPL18 | -0.59 | 0 |
| Pit mucous | Pit mucous | RPL31 | -0.61 | 0 |
| Pit mucous | Pit mucous | RPL29 | -0.61 | 0 |
| Pit mucous | Pit mucous | RPS12 | -0.62 | 0 |
| Pit mucous | Pit mucous | RPS3 | -0.63 | 0 |
| Pit mucous | Pit mucous | PRDX2 | -0.63 | 0 |
| Pit mucous | Pit mucous | HINT1 | -0.64 | 0 |
| Pit mucous | Pit mucous | RPL35A | -0.65 | 0 |
| Pit mucous | Pit mucous | EIF3L | -0.65 | 0 |
| Pit mucous | Pit mucous | RPL7A | -0.67 | 0 |
| Pit mucous | Pit mucous | EEF1A1 | -0.67 | 0 |
| Pit mucous | Pit mucous | RPS15 | -0.69 | 0 |
| Pit mucous | Pit mucous | RPS16 | -0.69 | 0 |
| Pit mucous | Pit mucous | HNRNPA1 | -0.69 | 0 |
| Pit mucous | Pit mucous | RPL14 | -0.71 | 0 |
| Pit mucous | Pit mucous | RPSA | -0.73 | 0 |
| Pit mucous | Pit mucous | RPL13A | -0.74 | 0 |
| Pit mucous | Pit mucous | RPL37 | -0.74 | 0 |
| Pit mucous | Pit mucous | NACA | -0.74 | 0 |
| Pit mucous | Pit mucous | RPL11 | -0.74 | 0 |
| Pit mucous | Pit mucous | RPL8 | -0.77 | 0 |
| Pit mucous | Pit mucous | RPL36A | -0.77 | 0 |
| Pit mucous | Pit mucous | RPL19 | -0.77 | 0 |
| Pit mucous | Pit mucous | RPS9 | -0.78 | 0 |
| Pit mucous | Pit mucous | RPL30 | -0.78 | 0 |
| Pit mucous | Pit mucous | RPL23 | -0.8 | 0 |
| Pit mucous | Pit mucous | RPS23 | -0.8 | 0 |
| Pit mucous | Pit mucous | RPS18 | -0.82 | 0 |
| Pit mucous | Pit mucous | RACK1 | -0.82 | 0 |
| Pit mucous | Pit mucous | S100A10 | -0.84 | 0 |
| Pit mucous | Pit mucous | NPM1 | -0.85 | 0 |
| Pit mucous | Pit mucous | EEF1B2 | -0.85 | 0 |
| Pit mucous | Pit mucous | RPS20 | -0.86 | 0 |
| Pit mucous | Pit mucous | RPS4X | -0.87 | 0 |
| Pit mucous | Pit mucous | RPL13 | -0.88 | 0 |
| Pit mucous | Pit mucous | EIF3E | -0.89 | 0 |
| Pit mucous | Pit mucous | RPL22 | -0.9 | 0 |
| Pit mucous | Pit mucous | FTL | -0.9 | 0 |
| Pit mucous | Pit mucous | RPS13 | -0.91 | 0 |
| Pit mucous | Pit mucous | RPL32 | -0.92 | 0 |
| Pit mucous | Pit mucous | RPS6 | -0.92 | 0 |
| Pit mucous | Pit mucous | RPS5 | -0.92 | 0 |
| Pit mucous | Pit mucous | RPS15A | -0.95 | 0 |
| Pit mucous | Pit mucous | RPL4 | -0.96 | 0 |
| Pit mucous | Pit mucous | RPL37A | -0.97 | 0 |
| Pit mucous | Pit mucous | RPL26 | -0.98 | 0 |
| Pit mucous | Pit mucous | GAPDH | -1.01 | 0 |
| Pit mucous | Pit mucous | RPL17 | -1.02 | 0 |
| Pit mucous | Pit mucous | CLDN7 | -1.04 | 0 |
| Pit mucous | Pit mucous | RPS3A | -1.04 | 0 |
| Pit mucous | Pit mucous | RPS4Y1 | -1.05 | 0 |
| Pit mucous | Pit mucous | RPS8 | -1.06 | 0 |
| Pit mucous | Pit mucous | SLC25A6 | -1.06 | 0 |
| Pit mucous | Pit mucous | RPL3 | -1.06 | 0 |
| Pit mucous | Pit mucous | RPS19 | -1.1 | 0 |
| Pit mucous | Pit mucous | GAST | -1.11 | 0 |
| Pit mucous | Pit mucous | RPL5 | -1.12 | 0 |
| Pit mucous | Pit mucous | RPS27A | -1.15 | 0 |
| Pit mucous | Pit mucous | RPS7 | -1.18 | 0 |
| Pit mucous | Pit mucous | RPL34 | -1.21 | 0 |
| Pit mucous | Pit mucous | RPL10A | -1.24 | 0 |
| Pit mucous | Pit mucous | RPL21 | -1.25 | 0 |
| Pit mucous | Pit mucous | ADIRF | -1.26 | 0 |
| Pit mucous | Pit mucous | RPL6 | -1.32 | 0 |
| Pit mucous | Pit mucous | RPS2 | -1.33 | 0 |
| Pit mucous | Pit mucous | RPL15 | -1.35 | 0 |
| Pit mucous | Pit mucous | PIGR | -1.39 | 0 |
| Pit mucous | Pit mucous | CLDN4 | -1.42 | 0 |
| Pit mucous | Pit mucous | CD74 | -1.54 | 0 |
| Pit mucous | Pit mucous | RPL7 | -1.71 | 0 |
| Pit mucous | Pit mucous | MT1H | 1.11 | 5.00E-305 |
| Pit mucous | Pit mucous | RPS24 | -0.56 | 2.01E-302 |
| Pit mucous | Pit mucous | RPL24 | -0.53 | 1.58E-300 |
| Pit mucous | Pit mucous | EZR | 0.63 | 6.97E-300 |
| Pit mucous | Pit mucous | ATP5F1A | -0.61 | 8.20E-300 |
| Pit mucous | Pit mucous | RPL23A | -0.5 | 1.17E-296 |
| Pit mucous | Pit mucous | MT2A | 0.93 | 5.22E-296 |
| Pit mucous | Pit mucous | CALM1 | -0.66 | 1.65E-293 |
| Pit mucous | Pit mucous | SPCS1 | -0.63 | 3.03E-293 |
| Pit mucous | Pit mucous | MT1E | 0.79 | 3.27E-293 |
| Pit mucous | Pit mucous | RPL12 | -0.48 | 3.70E-293 |
| Pit mucous | Pit mucous | RPS27 | -0.52 | 2.09E-292 |
| Pit mucous | Pit mucous | RPS21 | -0.56 | 4.25E-292 |
| Pit mucous | Pit mucous | REG4 | -2.22 | 1.52E-291 |
| Pit mucous | Pit mucous | NQO1 | 0.59 | 3.59E-291 |
| Pit mucous | Pit mucous | GSTP1 | 0.44 | 7.47E-291 |
| Pit mucous | Pit mucous | TFF3 | -2.07 | 4.50E-290 |
| Pit mucous | Pit mucous | STARD10 | 0.7 | 5.92E-289 |
| Pit mucous | Pit mucous | UQCRB | -0.62 | 1.13E-287 |
| Pit mucous | Pit mucous | RHOB | 0.72 | 4.02E-287 |
| Pit mucous | Pit mucous | RFLNA | 0.78 | 1.41E-284 |
| Pit mucous | Pit mucous | NPC2 | -0.62 | 1.13E-282 |
| Pit mucous | Pit mucous | VAMP8 | 0.65 | 5.00E-282 |
| Pit mucous | Pit mucous | UQCRH | -0.56 | 8.42E-282 |
| Pit mucous | Pit mucous | CLDN3 | -0.94 | 1.83E-281 |
| Pit mucous | Pit mucous | ATP5MC1 | -0.6 | 8.35E-272 |
| Pit mucous | Pit mucous | LDHA | -0.73 | 2.43E-271 |
| Pit mucous | Pit mucous | PRDX5 | 0.54 | 1.93E-270 |
| Pit mucous | Pit mucous | HSPB1 | 0.46 | 6.06E-269 |
| Pit mucous | Pit mucous | RPS25 | -0.48 | 3.18E-268 |
| Pit mucous | Pit mucous | ATP2A3 | 0.74 | 9.04E-268 |
| Pit mucous | Pit mucous | TPI1 | -0.58 | 8.71E-267 |
| Pit mucous | Pit mucous | RPS14 | -0.4 | 1.96E-266 |
| Pit mucous | Pit mucous | SH3BGRL3 | 0.41 | 6.44E-266 |
| Pit mucous | Pit mucous | PKM | -0.61 | 3.16E-262 |
| Pit mucous | Pit mucous | LDHB | -0.65 | 4.58E-262 |
| Pit mucous | Pit mucous | FABP1 | -2.71 | 6.39E-261 |
| Pit mucous | Pit mucous | EIF3F | -0.53 | 1.46E-258 |
| Pit mucous | Pit mucous | PPA1 | -0.59 | 4.26E-258 |
| Pit mucous | Pit mucous | BTF3 | -0.53 | 4.86E-258 |
| Pit mucous | Pit mucous | AC023090.1 | 0.7 | 6.87E-258 |
| Pit mucous | Pit mucous | ARPC1B | -0.61 | 2.94E-257 |
| Pit mucous | Pit mucous | HSPD1 | -0.62 | 1.21E-254 |
| Pit mucous | Pit mucous | RANBP1 | -0.58 | 6.58E-253 |
| Pit mucous | Pit mucous | REG1A | -2.37 | 5.62E-251 |
| Pit mucous | Pit mucous | RPS11 | -0.41 | 6.37E-249 |
| Pit mucous | Pit mucous | SMIM6 | 0.73 | 9.82E-249 |
| Pit mucous | Pit mucous | SMIM14 | 0.78 | 1.03E-247 |
| Pit mucous | Pit mucous | CYBA | -0.69 | 1.43E-246 |
| Pit mucous | Pit mucous | CYC1 | -0.59 | 2.03E-246 |
| Pit mucous | Pit mucous | IDH2 | -0.57 | 5.89E-246 |
| Pit mucous | Pit mucous | RPS29 | -0.42 | 2.45E-244 |
| Pit mucous | Pit mucous | NAP1L1 | -0.59 | 2.18E-242 |
| Pit mucous | Pit mucous | PSME2 | -0.62 | 3.42E-242 |
| Pit mucous | Pit mucous | FOXQ1 | 0.66 | 2.95E-241 |
| Pit mucous | Pit mucous | EIF3H | -0.48 | 3.01E-241 |
| Pit mucous | Pit mucous | CIRBP | -0.6 | 2.58E-240 |
| Pit mucous | Pit mucous | CD99 | -0.52 | 1.93E-239 |
| Pit mucous | Pit mucous | NDUFC2 | -0.54 | 1.36E-235 |
| Pit mucous | Pit mucous | RPL27A | -0.38 | 2.64E-235 |
| Pit mucous | Pit mucous | ANXA10 | 0.52 | 3.61E-235 |
| Pit mucous | Pit mucous | FAM177B | 0.55 | 3.41E-234 |
| Pit mucous | Pit mucous | HSPE1 | -0.59 | 2.93E-233 |
| Pit mucous | Pit mucous | KLF6 | 0.48 | 6.71E-233 |
| Pit mucous | Pit mucous | PEBP1 | -0.53 | 2.53E-231 |
| Pit mucous | Pit mucous | SRI | -0.55 | 1.41E-229 |
| Pit mucous | Pit mucous | UPK1B | 0.47 | 5.73E-228 |
| Pit mucous | Pit mucous | PRAP1 | -1.24 | 9.14E-228 |
| Pit mucous | Pit mucous | GPX1 | -0.46 | 1.85E-227 |
| Pit mucous | Pit mucous | EEF2 | -0.47 | 2.32E-224 |
| Pit mucous | Pit mucous | HLA-DRA | -1.04 | 2.94E-224 |
| Pit mucous | Pit mucous | PLA2G10 | 0.76 | 3.69E-223 |
| Pit mucous | Pit mucous | COX7A2L | -0.45 | 4.19E-222 |
| Pit mucous | Pit mucous | ATP5F1B | -0.52 | 1.33E-221 |
| Pit mucous | Pit mucous | LY6E | -0.59 | 4.52E-219 |
| Pit mucous | Pit mucous | PHB | -0.45 | 2.18E-218 |
| Pit mucous | Pit mucous | SYTL2 | 0.7 | 5.10E-218 |
| Pit mucous | Pit mucous | GIPR | 0.57 | 6.82E-218 |
| Pit mucous | Pit mucous | CISD1 | -0.44 | 1.50E-217 |
| Pit mucous | Pit mucous | SLC25A5 | -0.66 | 4.04E-217 |
| Pit mucous | Pit mucous | NPW | 0.62 | 1.33E-216 |
| Pit mucous | Pit mucous | CD44 | -0.58 | 4.88E-216 |
| Pit mucous | Pit mucous | REP15 | 0.55 | 1.27E-215 |
| Pit mucous | Pit mucous | EPCAM | -0.71 | 1.89E-215 |
| Pit mucous | Pit mucous | TSPAN1 | 0.63 | 1.17E-214 |
| Pit mucous | Pit mucous | PYCARD | -0.48 | 4.72E-214 |
| Pit mucous | Pit mucous | COMMD6 | -0.47 | 5.63E-214 |
| Pit mucous | Pit mucous | EIF3K | -0.44 | 1.73E-213 |
| Pit mucous | Pit mucous | PRDX4 | -0.51 | 2.78E-213 |
| Pit mucous | Pit mucous | ELOB | 0.41 | 3.93E-213 |
| Pit mucous | Pit mucous | CUTA | -0.44 | 6.52E-213 |
| Pit mucous | Pit mucous | CST3 | 0.34 | 6.58E-213 |
| Pit mucous | Pit mucous | PARK7 | -0.43 | 9.66E-213 |
| Pit mucous | Pit mucous | MLPH | 0.74 | 1.38E-212 |
| Pit mucous | Pit mucous | HSPA8 | -0.55 | 3.91E-212 |
| Pit mucous | Pit mucous | MIF | -0.52 | 6.88E-212 |
| Pit mucous | Pit mucous | PPP1R1B | -0.45 | 1.18E-211 |
| Pit mucous | Pit mucous | SUCLG1 | -0.46 | 6.00E-211 |
| Pit mucous | Pit mucous | OTUD1 | 0.67 | 1.21E-209 |
| Pit mucous | Pit mucous | NHP2 | -0.45 | 1.34E-209 |
| Pit mucous | Pit mucous | RPL27 | -0.43 | 1.10E-208 |
| Pit mucous | Pit mucous | GALNT6 | 0.71 | 2.10E-208 |
| Pit mucous | Pit mucous | EIF3M | -0.43 | 2.49E-207 |
| Pit mucous | Pit mucous | CLU | -0.81 | 6.39E-207 |
| Pit mucous | Pit mucous | FABP2 | -1.43 | 1.21E-206 |
| Pit mucous | Pit mucous | ETHE1 | -0.57 | 2.08E-206 |
| Pit mucous | Pit mucous | ANPEP | -1.34 | 1.26E-205 |
| Pit mucous | Pit mucous | H3F3B | -0.5 | 1.48E-204 |
| Pit mucous | Pit mucous | COX4I1 | -0.38 | 2.58E-204 |
| Pit mucous | Pit mucous | POLD4 | 0.65 | 3.05E-204 |
| Pit mucous | Pit mucous | SQSTM1 | 0.62 | 3.36E-204 |
| Pit mucous | Pit mucous | SCGB2A1 | 0.35 | 2.12E-203 |
| Pit mucous | Pit mucous | CDH17 | -0.69 | 1.11E-202 |
| Pit mucous | Pit mucous | HLA-DRB1 | -0.87 | 2.08E-201 |
| Pit mucous | Pit mucous | CTNNBL1 | 0.76 | 6.30E-201 |
| Pit mucous | Pit mucous | MT-CYB | -0.61 | 1.72E-200 |
| Pit mucous | Pit mucous | OLFM4 | -1.57 | 6.62E-200 |
| Pit mucous | Pit mucous | RSL1D1 | -0.42 | 1.68E-199 |
| Pit mucous | Pit mucous | EDN1 | -0.54 | 2.13E-199 |
| Pit mucous | Pit mucous | TUFM | -0.41 | 2.88E-199 |
| Pit mucous | Pit mucous | FBL | -0.41 | 4.04E-199 |
| Pit mucous | Pit mucous | SPTSSA | -0.39 | 6.53E-198 |
| Pit mucous | Pit mucous | SNRPE | -0.43 | 2.32E-197 |
| Pit mucous | Pit mucous | IMPDH2 | -0.45 | 6.44E-197 |
| Pit mucous | Pit mucous | YBX1 | -0.46 | 2.67E-196 |
| Pit mucous | Pit mucous | APP | -0.4 | 3.38E-196 |
| Pit mucous | Pit mucous | ATP5MC3 | -0.54 | 6.32E-196 |
| Pit mucous | Pit mucous | MRPL51 | -0.38 | 1.25E-195 |
| Pit mucous | Pit mucous | ATP1B3 | -0.49 | 2.85E-194 |
| Pit mucous | Pit mucous | ATP5F1C | -0.46 | 5.84E-193 |
| Pit mucous | Pit mucous | NDUFAB1 | -0.41 | 9.19E-192 |
| Pit mucous | Pit mucous | RBM3 | -0.44 | 1.20E-191 |
| Pit mucous | Pit mucous | GHITM | -0.4 | 7.75E-190 |
| Pit mucous | Pit mucous | SKP1 | -0.5 | 2.98E-189 |
| Pit mucous | Pit mucous | PSMB5 | -0.39 | 5.23E-189 |
| Pit mucous | Pit mucous | NME1 | -0.41 | 7.78E-189 |
| Pit mucous | Pit mucous | ATRAID | -0.4 | 2.01E-188 |
| Pit mucous | Pit mucous | ATP5PB | -0.42 | 2.18E-188 |
| Pit mucous | Pit mucous | UQCRC2 | -0.4 | 8.60E-188 |
| Pit mucous | Pit mucous | YWHAQ | -0.4 | 1.28E-187 |
| Pit mucous | Pit mucous | BICDL1 | 0.43 | 1.40E-187 |
| Pit mucous | Pit mucous | HSP90B1 | -0.48 | 8.88E-187 |
| Pit mucous | Pit mucous | ATP5PF | -0.5 | 9.79E-187 |
| Pit mucous | Pit mucous | MPC2 | -0.38 | 4.33E-186 |
| Pit mucous | Pit mucous | SLC25A3 | -0.44 | 1.91E-185 |
| Pit mucous | Pit mucous | KLF4 | 0.61 | 7.54E-185 |
| Pit mucous | Pit mucous | PSMA4 | -0.41 | 8.74E-184 |
| Pit mucous | Pit mucous | ATP5MPL | -0.44 | 1.46E-182 |
| Pit mucous | Pit mucous | HMGN1 | -0.43 | 1.43E-181 |
| Pit mucous | Pit mucous | SOD2 | -0.55 | 2.25E-181 |
| Pit mucous | Pit mucous | TXN | -0.66 | 6.14E-181 |
| Pit mucous | Pit mucous | IER3 | 0.5 | 5.18E-180 |
| Pit mucous | Pit mucous | UQCRFS1 | -0.42 | 7.55E-180 |
| Pit mucous | Pit mucous | RAB11FIP1 | 0.61 | 1.12E-179 |
| Pit mucous | Pit mucous | ERGIC3 | -0.38 | 1.21E-179 |
| Pit mucous | Pit mucous | MRPS33 | -0.34 | 1.23E-179 |
| Pit mucous | Pit mucous | HMGN2 | -0.53 | 2.08E-179 |
| Pit mucous | Pit mucous | TMEM54 | 0.56 | 4.02E-179 |
| Pit mucous | Pit mucous | STMN1 | -0.65 | 6.19E-179 |
| Pit mucous | Pit mucous | SEC62 | -0.41 | 6.64E-179 |
| Pit mucous | Pit mucous | EEF1G | -0.37 | 7.35E-179 |
| Pit mucous | Pit mucous | AL138930.1 | 0.5 | 3.17E-178 |
| Pit mucous | Pit mucous | TMEM147 | -0.35 | 7.65E-178 |
| Pit mucous | Pit mucous | IL17RE | 0.57 | 1.12E-177 |
| Pit mucous | Pit mucous | CXCL3 | -1.06 | 4.01E-177 |
| Pit mucous | Pit mucous | HMGN3 | -0.38 | 8.29E-177 |
| Pit mucous | Pit mucous | ESD | -0.36 | 6.99E-176 |
| Pit mucous | Pit mucous | BANF1 | -0.36 | 1.51E-175 |
| Pit mucous | Pit mucous | HSD17B11 | -0.38 | 1.58E-175 |
| Pit mucous | Pit mucous | SEC11A | -0.34 | 3.76E-175 |
| Pit mucous | Pit mucous | CNBP | -0.37 | 1.42E-174 |
| Pit mucous | Pit mucous | EIF3D | -0.37 | 7.52E-174 |
| Pit mucous | Pit mucous | MALAT1 | -0.47 | 1.23E-173 |
| Pit mucous | Pit mucous | RSL24D1 | -0.4 | 2.51E-172 |
| Pit mucous | Pit mucous | TM4SF4 | -0.84 | 8.83E-172 |
| Pit mucous | Pit mucous | NDUFB5 | -0.35 | 2.08E-171 |
| Pit mucous | Pit mucous | TMEM176B | -0.62 | 1.80E-169 |
| Pit mucous | Pit mucous | TRMT112 | -0.38 | 1.96E-169 |
| Pit mucous | Pit mucous | SRP14 | -0.41 | 2.56E-169 |
| Pit mucous | Pit mucous | PYURF | -0.36 | 1.69E-168 |
| Pit mucous | Pit mucous | SNRPD2 | -0.38 | 3.31E-168 |
| Pit mucous | Pit mucous | TCEAL9 | 0.66 | 3.84E-168 |
| Pit mucous | Pit mucous | IL1RN | 0.77 | 2.24E-167 |
| Pit mucous | Pit mucous | DECR1 | -0.4 | 4.67E-167 |
| Pit mucous | Pit mucous | PA2G4 | -0.37 | 8.42E-167 |
| Pit mucous | Pit mucous | GLRX | -0.46 | 3.89E-166 |
| Pit mucous | Pit mucous | ATP5PO | -0.4 | 1.14E-165 |
| Pit mucous | Pit mucous | PRDX3 | -0.32 | 2.19E-165 |
| Pit mucous | Pit mucous | APEX1 | -0.32 | 7.55E-165 |
| Pit mucous | Pit mucous | MT-CO2 | -0.83 | 1.60E-163 |
| Pit mucous | Pit mucous | CNPY2 | -0.32 | 2.18E-163 |
| Pit mucous | Pit mucous | LSM5 | -0.37 | 7.74E-163 |
| Pit mucous | Pit mucous | SFN | 0.47 | 8.68E-163 |
| Pit mucous | Pit mucous | MRPL12 | -0.36 | 1.33E-162 |
| Pit mucous | Pit mucous | BLOC1S1 | 0.53 | 2.46E-162 |
| Pit mucous | Pit mucous | ABCC5 | 0.61 | 2.92E-162 |
| Pit mucous | Pit mucous | MT-ATP6 | -0.63 | 6.96E-162 |
| Pit mucous | Pit mucous | ATP5MC2 | -0.37 | 1.15E-161 |
| Pit mucous | Pit mucous | DSC2 | 0.7 | 1.86E-161 |
| Pit mucous | Pit mucous | MDH2 | -0.36 | 2.33E-161 |
| Pit mucous | Pit mucous | PSMB1 | -0.34 | 2.79E-161 |
| Pit mucous | Pit mucous | RPS10 | -0.41 | 3.04E-161 |
| Pit mucous | Pit mucous | SEC61G | 0.47 | 8.95E-160 |
| Pit mucous | Pit mucous | CD9 | -0.54 | 1.67E-159 |
| Pit mucous | Pit mucous | MTCH2 | -0.35 | 2.44E-159 |
| Pit mucous | Pit mucous | CXCL2 | -0.94 | 2.57E-159 |
| Pit mucous | Pit mucous | CAMLG | -0.3 | 3.92E-159 |
| Pit mucous | Pit mucous | PSMA7 | -0.37 | 1.80E-158 |
| Pit mucous | Pit mucous | HLA-C | -0.56 | 4.51E-158 |
| Pit mucous | Pit mucous | MALL | -0.64 | 9.64E-158 |
| Pit mucous | Pit mucous | SLC44A4 | 0.65 | 2.43E-157 |
| Pit mucous | Pit mucous | CCT3 | -0.3 | 2.47E-157 |
| Pit mucous | Pit mucous | RAC1 | 0.55 | 2.09E-156 |
| Pit mucous | Pit mucous | HMGB1 | -0.44 | 5.49E-156 |
| Pit mucous | Pit mucous | PHB2 | -0.28 | 7.50E-156 |
| Pit mucous | Pit mucous | FOXA3 | 0.69 | 1.61E-155 |
| Pit mucous | Pit mucous | HSP90AB1 | -0.44 | 6.25E-155 |
| Pit mucous | Pit mucous | SSBP1 | -0.33 | 2.20E-154 |
| Pit mucous | Pit mucous | ECHS1 | -0.31 | 3.91E-154 |
| Pit mucous | Pit mucous | PSMA1 | -0.31 | 6.16E-154 |
| Pit mucous | Pit mucous | CLNS1A | -0.32 | 1.11E-153 |
| Pit mucous | Pit mucous | SGSM3 | 0.66 | 2.02E-153 |
| Pit mucous | Pit mucous | PHLDA1 | -0.49 | 2.16E-153 |
| Pit mucous | Pit mucous | RPL36 | -0.33 | 2.79E-153 |
| Pit mucous | Pit mucous | RAN | -0.37 | 3.20E-153 |
| Pit mucous | Pit mucous | GGCT | -0.33 | 3.23E-153 |
| Pit mucous | Pit mucous | NDUFS8 | -0.3 | 3.53E-153 |
| Pit mucous | Pit mucous | BLVRB | 0.61 | 5.32E-152 |
| Pit mucous | Pit mucous | PSMA3 | -0.3 | 1.63E-151 |
| Pit mucous | Pit mucous | HSD17B10 | -0.29 | 2.82E-151 |
| Pit mucous | Pit mucous | LMO7 | 0.58 | 7.87E-151 |
| Pit mucous | Pit mucous | IGSF9 | 0.45 | 1.87E-150 |
| Pit mucous | Pit mucous | CCT8 | -0.29 | 2.16E-150 |
| Pit mucous | Pit mucous | MYL6 | 0.28 | 5.46E-150 |
| Pit mucous | Pit mucous | MT1X | 0.81 | 1.51E-149 |
| Pit mucous | Pit mucous | IFITM3 | -0.69 | 1.56E-149 |
| Pit mucous | Pit mucous | ALDOB | -0.83 | 3.20E-149 |
| Pit mucous | Pit mucous | FKBP11 | -0.6 | 5.90E-149 |
| Pit mucous | Pit mucous | NDUFA9 | -0.3 | 1.16E-148 |
| Pit mucous | Pit mucous | WFDC2 | -0.51 | 4.33E-148 |
| Pit mucous | Pit mucous | MRPL13 | -0.32 | 8.55E-148 |
| Pit mucous | Pit mucous | VDAC3 | -0.3 | 3.14E-147 |
| Pit mucous | Pit mucous | SLC9A3R2 | 0.38 | 3.76E-147 |
| Pit mucous | Pit mucous | PGAM1 | -0.33 | 3.88E-147 |
| Pit mucous | Pit mucous | NDUFV1 | -0.28 | 4.26E-147 |
| Pit mucous | Pit mucous | AHNAK | 0.59 | 4.65E-146 |
| Pit mucous | Pit mucous | TIMM13 | -0.32 | 6.42E-146 |
| Pit mucous | Pit mucous | PPP1CC | -0.29 | 6.90E-146 |
| Pit mucous | Pit mucous | PDIA6 | -0.34 | 1.20E-145 |
| Pit mucous | Pit mucous | DMBT1 | -0.61 | 1.25E-145 |
| Pit mucous | Pit mucous | ST13 | -0.29 | 2.95E-145 |
| Pit mucous | Pit mucous | EID1 | -0.39 | 3.40E-145 |
| Pit mucous | Pit mucous | LRPAP1 | -0.28 | 5.99E-145 |
| Pit mucous | Pit mucous | GPR160 | -0.31 | 6.08E-145 |
| Pit mucous | Pit mucous | CNIH1 | -0.27 | 2.42E-143 |
| Pit mucous | Pit mucous | ALDOC | 0.45 | 2.74E-143 |
| Pit mucous | Pit mucous | ANXA2 | -0.53 | 2.81E-143 |
| Pit mucous | Pit mucous | ZFAS1 | -0.37 | 3.29E-143 |
| Pit mucous | Pit mucous | NDUFS4 | -0.27 | 2.22E-142 |
| Pit mucous | Pit mucous | C1QBP | -0.33 | 2.68E-142 |
| Pit mucous | Pit mucous | TOMM22 | -0.28 | 2.99E-142 |
| Pit mucous | Pit mucous | RBMX | -0.29 | 9.91E-141 |
| Pit mucous | Pit mucous | LSM3 | -0.32 | 1.08E-140 |
| Pit mucous | Pit mucous | SLC39A11 | 0.62 | 1.08E-140 |
| Pit mucous | Pit mucous | RGS2 | -0.51 | 1.28E-140 |
| Pit mucous | Pit mucous | JUNB | -0.87 | 1.40E-140 |
| Pit mucous | Pit mucous | CCND1 | 0.69 | 1.46E-140 |
| Pit mucous | Pit mucous | ERH | -0.32 | 1.69E-140 |
| Pit mucous | Pit mucous | TMEM205 | -0.26 | 2.59E-140 |
| Pit mucous | Pit mucous | EHBP1L1 | 0.43 | 3.07E-140 |
| Pit mucous | Pit mucous | EIF4EBP1 | -0.28 | 5.26E-140 |
| Pit mucous | Pit mucous | TMEM176A | -0.44 | 5.28E-140 |
| Pit mucous | Pit mucous | STOML2 | -0.28 | 6.45E-140 |
| Pit mucous | Pit mucous | 7-Sep | -0.35 | 7.90E-140 |
| Pit mucous | Pit mucous | TMEM106C | -0.29 | 4.56E-139 |
| Pit mucous | Pit mucous | COX5A | -0.35 | 4.96E-139 |
| Pit mucous | Pit mucous | PRXL2A | -0.28 | 6.12E-139 |
| Pit mucous | Pit mucous | PRMT1 | -0.3 | 3.15E-138 |
| Pit mucous | Pit mucous | ALG5 | -0.29 | 7.44E-138 |
| Pit mucous | Pit mucous | TMEM256 | -0.27 | 8.21E-138 |
| Pit mucous | Pit mucous | RAB27B | 0.63 | 1.10E-137 |
| Pit mucous | Pit mucous | ABLIM1 | 0.65 | 9.03E-137 |
| Pit mucous | Pit mucous | SEC11C | -0.46 | 1.22E-136 |
| Pit mucous | Pit mucous | GADD45B | -0.77 | 2.11E-136 |
| Pit mucous | Pit mucous | RPS26 | -0.35 | 1.18E-135 |
| Pit mucous | Pit mucous | TMEM230 | -0.26 | 1.28E-135 |
| Pit mucous | Pit mucous | FAU | -0.27 | 1.41E-135 |
| Pit mucous | Pit mucous | CCT2 | -0.28 | 1.43E-134 |
| Pit mucous | Pit mucous | SDCBP2 | 0.48 | 1.84E-134 |
| Pit mucous | Pit mucous | SDHB | -0.26 | 2.65E-134 |
| Pit mucous | Pit mucous | UQCRC1 | -0.31 | 3.00E-134 |
| Pit mucous | Pit mucous | GPRC5A | -0.41 | 5.81E-134 |
| Pit mucous | Pit mucous | C4orf3 | -0.3 | 6.67E-134 |
| Pit mucous | Pit mucous | GSTM3 | -0.3 | 1.10E-133 |
| Pit mucous | Pit mucous | GDI2 | -0.28 | 1.53E-133 |
| Pit mucous | Pit mucous | GPA33 | -0.39 | 1.82E-133 |
| Pit mucous | Pit mucous | VPS29 | -0.28 | 2.13E-133 |
| Pit mucous | Pit mucous | EPS8L1 | 0.63 | 7.21E-133 |
| Pit mucous | Pit mucous | AHCY | -0.25 | 8.42E-133 |
| Pit mucous | Pit mucous | PSME1 | -0.3 | 2.70E-132 |
| Pit mucous | Pit mucous | ATF4 | 0.55 | 7.02E-132 |
| Pit mucous | Pit mucous | H2AFZ | -0.47 | 1.42E-131 |
| Pit mucous | Pit mucous | TM4SF20 | -0.65 | 1.51E-131 |
| Pit mucous | Pit mucous | FAM3C | -0.3 | 1.71E-131 |
| Pit mucous | Pit mucous | SNRPB | -0.29 | 2.35E-131 |
| Pit mucous | Pit mucous | ANXA5 | -0.3 | 3.64E-131 |
| Pit mucous | Pit mucous | NDUFA5 | -0.28 | 4.32E-131 |
| Pit mucous | Pit mucous | TENT5A | 0.63 | 4.60E-131 |
| Pit mucous | Pit mucous | ASAH1 | -0.27 | 5.58E-131 |
| Pit mucous | Pit mucous | PTGES3 | -0.29 | 1.13E-130 |
| Pit mucous | Pit mucous | CTSC | -0.38 | 1.61E-130 |
| Pit mucous | Pit mucous | SMIM31 | -0.34 | 1.71E-130 |
| Pit mucous | Pit mucous | CKLF | -0.36 | 2.13E-130 |
| Pit mucous | Pit mucous | RTRAF | -0.27 | 3.75E-130 |
| Pit mucous | Pit mucous | EMC4 | -0.26 | 5.97E-130 |
| Pit mucous | Pit mucous | CLDN15 | -0.29 | 6.05E-130 |
| Pit mucous | Pit mucous | GTF3C6 | -0.26 | 7.51E-130 |
| Pit mucous | Pit mucous | GADD45GIP1 | -0.28 | 8.02E-130 |
| Pit mucous | Pit mucous | UBE2I | -0.26 | 8.51E-130 |
| Pit mucous | Pit mucous | RAMP1 | -0.3 | 1.71E-129 |
| Pit mucous | Pit mucous | DUT | -0.34 | 2.05E-129 |
| Pit mucous | Pit mucous | SNRPB2 | -0.26 | 3.54E-129 |
| Pit mucous | Pit mucous | SAT2 | -0.28 | 4.64E-129 |
| Pit mucous | Pit mucous | SELENOM | -0.49 | 7.37E-129 |
| Pit mucous | Pit mucous | PPIF | 0.65 | 7.41E-129 |
| Pit mucous | Pit mucous | MDH1 | -0.26 | 1.80E-128 |
| Pit mucous | Pit mucous | PRELID1 | -0.3 | 2.14E-128 |
| Pit mucous | Pit mucous | CNN3 | -0.36 | 3.12E-128 |
| Pit mucous | Pit mucous | NR4A1 | -0.31 | 3.27E-128 |
| Pit mucous | Pit mucous | CCDC88B | 0.61 | 4.52E-128 |
| Pit mucous | Pit mucous | JPT1 | 0.39 | 1.62E-127 |
| Pit mucous | Pit mucous | MRPS12 | -0.26 | 1.65E-127 |
| Pit mucous | Pit mucous | SLC7A11 | 0.57 | 2.65E-127 |
| Pit mucous | Pit mucous | LCN2 | -0.57 | 4.05E-127 |
| Pit mucous | Pit mucous | NDUFB9 | -0.35 | 1.11E-126 |
| Pit mucous | Pit mucous | SRSF7 | -0.33 | 1.72E-126 |
| Pit mucous | Pit mucous | DPP7 | -0.31 | 5.68E-126 |
| Pit mucous | Pit mucous | CD47 | -0.27 | 8.65E-126 |
| Pit mucous | Pit mucous | PON2 | -0.26 | 9.29E-126 |
| Pit mucous | Pit mucous | SELENOT | -0.27 | 1.01E-125 |
| Pit mucous | Pit mucous | C11orf58 | -0.27 | 1.21E-125 |
| Pit mucous | Pit mucous | NOP53 | -0.34 | 1.89E-125 |
| Pit mucous | Pit mucous | CARHSP1 | -0.3 | 2.00E-125 |
| Pit mucous | Pit mucous | MAPK3 | 0.63 | 3.97E-125 |
| Pit mucous | Pit mucous | PSMB6 | -0.26 | 1.08E-124 |
| Pit mucous | Pit mucous | ASS1 | -0.28 | 2.50E-124 |
| Pit mucous | Pit mucous | FTH1 | -0.34 | 3.03E-124 |
| Pit mucous | Pit mucous | SPATA5 | 0.34 | 3.76E-124 |
| Pit mucous | Pit mucous | SOD1 | -0.33 | 4.21E-124 |
| Pit mucous | Pit mucous | NCL | -0.27 | 6.07E-124 |
| Pit mucous | Pit mucous | SERPINA1 | -0.49 | 8.55E-124 |
| Pit mucous | Pit mucous | ENO1 | -0.35 | 1.07E-123 |
| Pit mucous | Pit mucous | CAMK2N1 | -0.3 | 2.13E-123 |
| Pit mucous | Pit mucous | VIM | -1.19 | 2.54E-123 |
| Pit mucous | Pit mucous | SLC39A5 | -0.26 | 3.51E-123 |
| Pit mucous | Pit mucous | ID1 | -0.5 | 4.95E-123 |
| Pit mucous | Pit mucous | CD320 | -0.68 | 6.34E-123 |
| Pit mucous | Pit mucous | NDUFA12 | -0.27 | 8.23E-123 |
| Pit mucous | Pit mucous | SH3BGRL | -0.27 | 1.64E-122 |
| Pit mucous | Pit mucous | C19orf48 | -0.26 | 5.73E-122 |
| Pit mucous | Pit mucous | SERPINB6 | -0.34 | 1.02E-121 |
| Pit mucous | Pit mucous | KHK | -0.37 | 2.24E-121 |
| Pit mucous | Pit mucous | YWHAH | 0.63 | 3.17E-121 |
| Pit mucous | Pit mucous | LAPTM4A | -0.35 | 1.97E-120 |
| Pit mucous | Pit mucous | RPL39 | -0.3 | 6.60E-120 |
| Pit mucous | Pit mucous | MT-ND1 | -0.46 | 2.55E-119 |
| Pit mucous | Pit mucous | CYBRD1 | -0.39 | 2.87E-119 |
| Pit mucous | Pit mucous | ISCU | -0.26 | 6.01E-119 |
| Pit mucous | Pit mucous | SUMO1 | -0.26 | 6.87E-119 |
| Pit mucous | Pit mucous | MT-ND2 | -0.4 | 1.92E-118 |
| Pit mucous | Pit mucous | ID3 | -0.57 | 2.82E-118 |
| Pit mucous | Pit mucous | SLC9A1 | 0.62 | 2.97E-118 |
| Pit mucous | Pit mucous | ANXA13 | -0.45 | 3.05E-118 |
| Pit mucous | Pit mucous | SRP9 | -0.25 | 5.21E-118 |
| Pit mucous | Pit mucous | NUCKS1 | -0.3 | 6.13E-118 |
| Pit mucous | Pit mucous | RBP2 | -1.47 | 1.12E-117 |
| Pit mucous | Pit mucous | CSTB | 0.41 | 1.20E-117 |
| Pit mucous | Pit mucous | FAM3D | -0.32 | 1.41E-117 |
| Pit mucous | Pit mucous | COX17 | 0.52 | 1.54E-117 |
| Pit mucous | Pit mucous | CPS1 | -0.29 | 1.98E-117 |
| Pit mucous | Pit mucous | PEPD | -0.27 | 7.98E-117 |
| Pit mucous | Pit mucous | HLA-DMA | -0.32 | 1.20E-116 |
| Pit mucous | Pit mucous | SPCS2 | -0.35 | 2.74E-115 |
| Pit mucous | Pit mucous | CAPN9 | 0.44 | 2.96E-115 |
| Pit mucous | Pit mucous | MRPL47 | -0.25 | 7.30E-115 |
| Pit mucous | Pit mucous | C6orf48 | -0.28 | 7.48E-115 |
| Pit mucous | Pit mucous | NDUFS7 | -0.27 | 3.18E-114 |
| Pit mucous | Pit mucous | ARL4A | -0.27 | 8.77E-114 |
| Pit mucous | Pit mucous | PFDN5 | -0.3 | 1.38E-113 |
| Pit mucous | Pit mucous | CACFD1 | 0.55 | 1.74E-113 |
| Pit mucous | Pit mucous | SUB1 | -0.32 | 2.76E-113 |
| Pit mucous | Pit mucous | INO80C | 0.7 | 1.46E-112 |
| Pit mucous | Pit mucous | PCBP2 | -0.26 | 5.74E-112 |
| Pit mucous | Pit mucous | F2RL1 | 0.62 | 3.26E-111 |
| Pit mucous | Pit mucous | ANO7 | 0.37 | 8.34E-111 |
| Pit mucous | Pit mucous | ATP5MG | -0.28 | 1.06E-110 |
| Pit mucous | Pit mucous | PMP22 | -0.45 | 1.76E-110 |
| Pit mucous | Pit mucous | TUBB | -0.34 | 1.25E-109 |
| Pit mucous | Pit mucous | BSG | -0.27 | 2.00E-109 |
| Pit mucous | Pit mucous | PCK1 | -0.51 | 7.20E-109 |
| Pit mucous | Pit mucous | DYNLL1 | 0.37 | 9.77E-109 |
| Pit mucous | Pit mucous | TMED2 | -0.26 | 2.18E-108 |
| Pit mucous | Pit mucous | RAP2B | 0.6 | 1.16E-107 |
| Pit mucous | Pit mucous | SI | -0.38 | 1.17E-107 |
| Pit mucous | Pit mucous | REEP6 | -0.26 | 1.97E-107 |
| Pit mucous | Pit mucous | HLA-DPB1 | -0.68 | 2.91E-107 |
| Pit mucous | Pit mucous | SNX3 | -0.25 | 3.01E-107 |
| Pit mucous | Pit mucous | B2M | -0.58 | 4.94E-107 |
| Pit mucous | Pit mucous | IGLL5 | -2.65 | 5.41E-107 |
| Pit mucous | Pit mucous | MGST2 | -0.33 | 5.63E-107 |
| Pit mucous | Pit mucous | FXYD5 | -0.29 | 1.30E-106 |
| Pit mucous | Pit mucous | NBEAL1 | -0.33 | 2.81E-106 |
| Pit mucous | Pit mucous | KLF10 | -0.28 | 3.21E-106 |
| Pit mucous | Pit mucous | IFITM1 | -0.64 | 5.45E-106 |
| Pit mucous | Pit mucous | SELENOS | -0.26 | 5.67E-106 |
| Pit mucous | Pit mucous | TMEM38A | 0.48 | 7.06E-106 |
| Pit mucous | Pit mucous | EIF1 | -0.3 | 7.19E-105 |
| Pit mucous | Pit mucous | VDAC2 | -0.27 | 7.38E-104 |
| Pit mucous | Pit mucous | UGCG | 0.59 | 9.07E-104 |
| Pit mucous | Pit mucous | GATM | -0.27 | 1.16E-103 |
| Pit mucous | Pit mucous | RNF223 | 0.49 | 1.36E-102 |
| Pit mucous | Pit mucous | POLR2L | 0.35 | 6.56E-102 |
| Pit mucous | Pit mucous | CYP3A5 | 0.62 | 2.25E-101 |
| Pit mucous | Pit mucous | CTSZ | -0.28 | 6.07E-101 |
| Pit mucous | Pit mucous | RIOK3 | 0.61 | 6.30E-101 |
| Pit mucous | Pit mucous | DDC | -0.25 | 1.68E-100 |
| Pit mucous | Pit mucous | ARL4C | 0.39 | 9.10E-100 |
| Pit mucous | Pit mucous | RPL36AL | 0.31 | 7.51E-99 |
| Pit mucous | Pit mucous | FAM102A | 0.61 | 1.54E-98 |
| Pit mucous | Pit mucous | FA2H | 0.53 | 2.38E-98 |
| Pit mucous | Pit mucous | KRT7 | -0.33 | 5.84E-98 |
| Pit mucous | Pit mucous | SARAF | -0.25 | 7.30E-98 |
| Pit mucous | Pit mucous | FUOM | -0.26 | 2.31E-97 |
| Pit mucous | Pit mucous | RPS27L | -0.31 | 8.18E-97 |
| Pit mucous | Pit mucous | BST2 | -0.38 | 1.15E-95 |
| Pit mucous | Pit mucous | HSPA5 | -0.38 | 1.87E-95 |
| Pit mucous | Pit mucous | ADM | -0.3 | 2.03E-94 |
| Pit mucous | Pit mucous | TMPRSS15 | -0.53 | 1.10E-93 |
| Pit mucous | Pit mucous | ABHD2 | 0.59 | 2.03E-93 |
| Pit mucous | Pit mucous | MYL12A | 0.31 | 2.74E-93 |
| Pit mucous | Pit mucous | IFITM2 | -0.56 | 8.90E-93 |
| Pit mucous | Pit mucous | TUBA1C | 0.49 | 9.87E-93 |
| Pit mucous | Pit mucous | RHOA | 0.43 | 1.37E-92 |
| Pit mucous | Pit mucous | RBM47 | 0.55 | 1.61E-92 |
| Pit mucous | Pit mucous | TUBA1A | -0.71 | 2.49E-92 |
| Pit mucous | Pit mucous | MUC13 | -0.41 | 3.18E-92 |
| Pit mucous | Pit mucous | SDC4 | 0.54 | 9.02E-91 |
| Pit mucous | Pit mucous | PTMA | -0.34 | 1.10E-90 |
| Pit mucous | Pit mucous | CD59 | 0.44 | 2.74E-90 |
| Pit mucous | Pit mucous | MTTP | -0.42 | 6.80E-90 |
| Pit mucous | Pit mucous | POMP | -0.3 | 8.27E-90 |
| Pit mucous | Pit mucous | EPS8 | 0.58 | 3.38E-89 |
| Pit mucous | Pit mucous | CXCL1 | -0.52 | 3.89E-89 |
| Pit mucous | Pit mucous | ID2 | -0.39 | 5.77E-89 |
| Pit mucous | Pit mucous | BRI3 | 0.6 | 1.17E-88 |
| Pit mucous | Pit mucous | HIST1H4C | -0.44 | 3.28E-88 |
| Pit mucous | Pit mucous | PCLAF | -0.35 | 3.84E-87 |
| Pit mucous | Pit mucous | MYADM | -0.29 | 7.98E-87 |
| Pit mucous | Pit mucous | HLA-DRB5 | -0.33 | 3.00E-85 |
| Pit mucous | Pit mucous | SPATS2L | 0.6 | 3.18E-84 |
| Pit mucous | Pit mucous | NR2F6 | 0.55 | 1.69E-83 |
| Pit mucous | Pit mucous | RAB27A | 0.48 | 1.97E-83 |
| Pit mucous | Pit mucous | EMP3 | -0.46 | 2.00E-83 |
| Pit mucous | Pit mucous | AGPAT2 | -0.51 | 9.78E-83 |
| Pit mucous | Pit mucous | HLA-DPA1 | -0.56 | 4.43E-82 |
| Pit mucous | Pit mucous | EIF4A1 | -0.27 | 2.02E-81 |
| Pit mucous | Pit mucous | SRGN | -0.97 | 3.49E-81 |
| Pit mucous | Pit mucous | CEBPB | 0.52 | 2.32E-80 |
| Pit mucous | Pit mucous | LGALS1 | -1.14 | 4.84E-80 |
| Pit mucous | Pit mucous | ATP5ME | 0.28 | 6.83E-79 |
| Pit mucous | Pit mucous | HLA-DQB1 | -0.33 | 1.42E-78 |
| Pit mucous | Pit mucous | ALDH3A1 | 0.43 | 1.85E-78 |
| Pit mucous | Pit mucous | MAL2 | 0.46 | 1.17E-77 |
| Pit mucous | Pit mucous | LASP1 | 0.55 | 5.60E-77 |
| Pit mucous | Pit mucous | AC020656.1 | -0.96 | 1.36E-76 |
| Pit mucous | Pit mucous | COX6C | -0.41 | 1.51E-76 |
| Pit mucous | Pit mucous | ARHGDIB | -0.47 | 2.34E-76 |
| Pit mucous | Pit mucous | NR4A2 | -0.3 | 2.71E-76 |
| Pit mucous | Pit mucous | ETNK1 | 0.52 | 2.26E-74 |
| Pit mucous | Pit mucous | SCP2 | 0.39 | 2.78E-74 |
| Pit mucous | Pit mucous | RHOF | 0.52 | 1.20E-73 |
| Pit mucous | Pit mucous | PLXNB2 | 0.55 | 1.22E-73 |
| Pit mucous | Pit mucous | MIA | -0.39 | 8.47E-73 |
| Pit mucous | Pit mucous | SELENOP | -0.31 | 8.86E-72 |
| Pit mucous | Pit mucous | INF2 | 0.54 | 1.07E-70 |
| Pit mucous | Pit mucous | C15orf48 | 0.34 | 2.09E-70 |
| Pit mucous | Pit mucous | HSP90AA1 | -0.36 | 3.14E-70 |
| Pit mucous | Pit mucous | DEPP1 | -0.3 | 6.00E-70 |
| Pit mucous | Pit mucous | ERBIN | 0.47 | 6.22E-69 |
| Pit mucous | Pit mucous | UBL3 | 0.55 | 8.90E-69 |
| Pit mucous | Pit mucous | ZBTB7C | 0.37 | 9.69E-69 |
| Pit mucous | Pit mucous | ELF3 | 0.28 | 1.01E-68 |
| Pit mucous | Pit mucous | SULT1C2 | 0.48 | 8.27E-68 |
| Pit mucous | Pit mucous | RGS10 | -0.27 | 1.19E-67 |
| Pit mucous | Pit mucous | SULT1A1 | -0.27 | 3.27E-67 |
| Pit mucous | Pit mucous | DNAJB1 | -0.4 | 3.96E-67 |
| Pit mucous | Pit mucous | PER3 | 0.35 | 8.06E-67 |
| Pit mucous | Pit mucous | HSD17B2 | -0.25 | 2.36E-66 |
| Pit mucous | Pit mucous | TPPP3 | -0.32 | 1.20E-65 |
| Pit mucous | Pit mucous | GDF15 | -0.3 | 1.52E-65 |
| Pit mucous | Pit mucous | LGALSL | 0.45 | 5.53E-65 |
| Pit mucous | Pit mucous | NDUFA1 | 0.3 | 5.19E-64 |
| Pit mucous | Pit mucous | TSPAN8 | -0.61 | 1.16E-63 |
| Pit mucous | Pit mucous | SLC44A2 | 0.48 | 5.52E-63 |
| Pit mucous | Pit mucous | CCL20 | -0.46 | 1.62E-62 |
| Pit mucous | Pit mucous | ANKRD28 | -0.41 | 1.88E-62 |
| Pit mucous | Pit mucous | CYR61 | -0.29 | 2.68E-62 |
| Pit mucous | Pit mucous | PARD6B | 0.55 | 3.00E-62 |
| Pit mucous | Pit mucous | PLCXD1 | 0.52 | 3.26E-62 |
| Pit mucous | Pit mucous | ITPKA | 0.29 | 3.96E-62 |
| Pit mucous | Pit mucous | SPINT1 | 0.53 | 4.38E-62 |
| Pit mucous | Pit mucous | CAPG | 0.46 | 1.22E-61 |
| Pit mucous | Pit mucous | PTGER4 | 0.45 | 1.23E-61 |
| Pit mucous | Pit mucous | SAP30 | 0.34 | 3.50E-61 |
| Pit mucous | Pit mucous | TST | 0.53 | 3.63E-61 |
| Pit mucous | Pit mucous | SOX4 | -0.27 | 9.60E-61 |
| Pit mucous | Pit mucous | ZFP36 | -0.54 | 2.14E-60 |
| Pit mucous | Pit mucous | AC105446.1 | 0.3 | 1.06E-59 |
| Pit mucous | Pit mucous | PRR4 | -0.68 | 1.12E-59 |
| Pit mucous | Pit mucous | LMO4 | 0.49 | 2.61E-59 |
| Pit mucous | Pit mucous | SST | -1.69 | 1.92E-57 |
| Pit mucous | Pit mucous | SH3BGRL2 | 0.5 | 2.50E-57 |
| Pit mucous | Pit mucous | ACTN4 | 0.42 | 4.35E-57 |
| Pit mucous | Pit mucous | JCHAIN | -2.77 | 4.59E-57 |
| Pit mucous | Pit mucous | TRNP1 | 0.42 | 4.80E-57 |
| Pit mucous | Pit mucous | SCIN | 0.38 | 5.63E-57 |
| Pit mucous | Pit mucous | MICALL2 | 0.47 | 9.52E-57 |
| Pit mucous | Pit mucous | DGAT1 | -0.32 | 1.22E-56 |
| Pit mucous | Pit mucous | SDCBP | 0.42 | 1.37E-56 |
| Pit mucous | Pit mucous | HMGCS1 | 0.52 | 1.84E-56 |
| Pit mucous | Pit mucous | CTNNA1 | 0.48 | 1.83E-55 |
| Pit mucous | Pit mucous | MUC6 | -1.08 | 2.72E-55 |
| Pit mucous | Pit mucous | CREM | -0.45 | 7.70E-55 |
| Pit mucous | Pit mucous | SOX21 | 0.32 | 1.04E-54 |
| Pit mucous | Pit mucous | TIMP1 | -0.33 | 1.32E-54 |
| Pit mucous | Pit mucous | LGALS9 | 0.49 | 2.72E-54 |
| Pit mucous | Pit mucous | C6orf222 | 0.46 | 3.87E-54 |
| Pit mucous | Pit mucous | ARL14 | 0.38 | 5.42E-54 |
| Pit mucous | Pit mucous | IL1R2 | 0.43 | 5.84E-54 |
| Pit mucous | Pit mucous | ATP5F1D | 0.39 | 1.27E-53 |
| Pit mucous | Pit mucous | GALE | 0.5 | 2.10E-53 |
| Pit mucous | Pit mucous | COL17A1 | 0.42 | 2.19E-53 |
| Pit mucous | Pit mucous | SDC1 | 0.53 | 3.51E-52 |
| Pit mucous | Pit mucous | RGS1 | -0.82 | 3.60E-52 |
| Pit mucous | Pit mucous | LGALS3 | -0.48 | 4.69E-52 |
| Pit mucous | Pit mucous | IL32 | -0.48 | 5.59E-52 |
| Pit mucous | Pit mucous | SPIRE2 | 0.36 | 1.28E-51 |
| Pit mucous | Pit mucous | TMBIM1 | 0.51 | 2.65E-51 |
| Pit mucous | Pit mucous | CBR1 | 0.3 | 6.21E-51 |
| Pit mucous | Pit mucous | PPARD | 0.39 | 9.28E-51 |
| Pit mucous | Pit mucous | COTL1 | 0.46 | 1.65E-50 |
| Pit mucous | Pit mucous | DUSP1 | 0.36 | 2.16E-50 |
| Pit mucous | Pit mucous | PIP4K2C | 0.37 | 1.49E-49 |
| Pit mucous | Pit mucous | KLF5 | 0.43 | 2.98E-49 |
| Pit mucous | Pit mucous | IFNGR2 | 0.48 | 5.71E-49 |
| Pit mucous | Pit mucous | LIPH | 0.55 | 6.20E-49 |
| Pit mucous | Pit mucous | P4HB | 0.3 | 4.05E-48 |
| Pit mucous | Pit mucous | ARF6 | 0.44 | 9.29E-48 |
| Pit mucous | Pit mucous | ITPR3 | 0.45 | 1.39E-47 |
| Pit mucous | Pit mucous | ZNF165 | 0.48 | 1.91E-47 |
| Pit mucous | Pit mucous | ITM2B | -0.26 | 2.18E-47 |
| Pit mucous | Pit mucous | TICAM1 | 0.57 | 2.51E-47 |
| Pit mucous | Pit mucous | CYP2C18 | 0.43 | 5.03E-47 |
| Pit mucous | Pit mucous | SSTR1 | 0.37 | 6.27E-47 |
| Pit mucous | Pit mucous | FAM174B | 0.34 | 2.44E-46 |
| Pit mucous | Pit mucous | FLNB | 0.48 | 3.10E-46 |
| Pit mucous | Pit mucous | MMP1 | -0.41 | 7.67E-46 |
| Pit mucous | Pit mucous | TMEM63B | 0.43 | 3.86E-45 |
| Pit mucous | Pit mucous | UBL5 | 0.29 | 5.83E-45 |
| Pit mucous | Pit mucous | DDX60 | 0.34 | 1.81E-44 |
| Pit mucous | Pit mucous | KCNK1 | 0.49 | 4.68E-43 |
| Pit mucous | Pit mucous | AAMDC | 0.45 | 4.90E-43 |
| Pit mucous | Pit mucous | YOD1 | 0.56 | 6.29E-43 |
| Pit mucous | Pit mucous | ST14 | 0.48 | 9.46E-41 |
| Pit mucous | Pit mucous | PRKCD | 0.32 | 1.73E-40 |
| Pit mucous | Pit mucous | SLC22A23 | 0.36 | 3.24E-40 |
| Pit mucous | Pit mucous | LIPF | -1.57 | 6.37E-40 |
| Pit mucous | Pit mucous | NUPR1 | 0.45 | 2.13E-39 |
| Pit mucous | Pit mucous | JUP | 0.48 | 5.83E-39 |
| Pit mucous | Pit mucous | MXD1 | 0.44 | 1.25E-38 |
| Pit mucous | Pit mucous | CRIP2 | -0.26 | 4.37E-38 |
| Pit mucous | Pit mucous | MT-CO1 | -0.43 | 1.29E-37 |
| Pit mucous | Pit mucous | XIST | 0.3 | 1.36E-37 |
| Pit mucous | Pit mucous | PET100 | 0.41 | 1.65E-37 |
| Pit mucous | Pit mucous | ZBTB43 | 0.49 | 1.80E-37 |
| Pit mucous | Pit mucous | RARRES2 | -0.42 | 2.36E-37 |
| Pit mucous | Pit mucous | SLC45A4 | 0.29 | 5.82E-37 |
| Pit mucous | Pit mucous | ARPC1A | 0.41 | 5.86E-37 |
| Pit mucous | Pit mucous | PKN1 | 0.27 | 6.18E-37 |
| Pit mucous | Pit mucous | ERO1B | -0.26 | 4.48E-36 |
| Pit mucous | Pit mucous | AKR1C3 | 0.31 | 1.85E-35 |
| Pit mucous | Pit mucous | CXCL8 | -0.81 | 5.02E-35 |
| Pit mucous | Pit mucous | SVIP | 0.41 | 2.81E-34 |
| Pit mucous | Pit mucous | DUSP5 | 0.43 | 3.60E-34 |
| Pit mucous | Pit mucous | PKP3 | 0.44 | 6.92E-34 |
| Pit mucous | Pit mucous | SIPA1L3 | 0.39 | 1.07E-33 |
| Pit mucous | Pit mucous | MDK | -0.25 | 1.44E-33 |
| Pit mucous | Pit mucous | CAPN5 | 0.43 | 1.84E-33 |
| Pit mucous | Pit mucous | ATF3 | 0.31 | 4.59E-33 |
| Pit mucous | Pit mucous | DSG2 | 0.5 | 1.42E-32 |
| Pit mucous | Pit mucous | FAM241A | 0.3 | 3.46E-32 |
| Pit mucous | Pit mucous | NBL1 | 0.43 | 8.95E-32 |
| Pit mucous | Pit mucous | TAX1BP3 | 0.41 | 1.32E-31 |
| Pit mucous | Pit mucous | FAM107B | 0.41 | 2.30E-31 |
| Pit mucous | Pit mucous | MYEOV | 0.29 | 3.81E-31 |
| Pit mucous | Pit mucous | TSC22D1 | 0.34 | 1.40E-30 |
| Pit mucous | Pit mucous | LAMA3 | 0.44 | 1.50E-30 |
| Pit mucous | Pit mucous | PXDC1 | 0.28 | 1.64E-30 |
| Pit mucous | Pit mucous | FAM3B | 0.4 | 3.29E-30 |
| Pit mucous | Pit mucous | CTNND1 | 0.44 | 6.54E-30 |
| Pit mucous | Pit mucous | RASSF6 | 0.4 | 7.39E-30 |
| Pit mucous | Pit mucous | ANO1 | 0.3 | 1.41E-29 |
| Pit mucous | Pit mucous | STK24 | 0.44 | 1.65E-29 |
| Pit mucous | Pit mucous | QSOX1 | 0.4 | 3.60E-29 |
| Pit mucous | Pit mucous | MYRF | 0.39 | 3.73E-29 |
| Pit mucous | Pit mucous | S100A4 | -0.67 | 1.77E-28 |
| Pit mucous | Pit mucous | AMN | 0.35 | 2.63E-28 |
| Pit mucous | Pit mucous | CTSD | 0.28 | 3.21E-28 |
| Pit mucous | Pit mucous | SLC44A1 | 0.42 | 4.42E-28 |
| Pit mucous | Pit mucous | CENPV | 0.32 | 4.50E-28 |
| Pit mucous | Pit mucous | MT-CO3 | -0.4 | 1.48E-27 |
| Pit mucous | Pit mucous | RARRES3 | 0.35 | 1.92E-27 |
| Pit mucous | Pit mucous | TMEM30B | 0.44 | 9.12E-27 |
| Pit mucous | Pit mucous | TSKU | 0.28 | 1.26E-26 |
| Pit mucous | Pit mucous | ITPKC | 0.45 | 1.46E-26 |
| Pit mucous | Pit mucous | SLC38A2 | 0.4 | 2.14E-26 |
| Pit mucous | Pit mucous | CDH1 | 0.39 | 3.03E-26 |
| Pit mucous | Pit mucous | EPN1 | 0.45 | 1.24E-25 |
| Pit mucous | Pit mucous | CREB3L1 | 0.42 | 1.43E-25 |
| Pit mucous | Pit mucous | DHCR24 | 0.39 | 1.52E-25 |
| Pit mucous | Pit mucous | HBB | 0.4 | 1.53E-25 |
| Pit mucous | Pit mucous | PGC | -1.58 | 1.56E-25 |
| Pit mucous | Pit mucous | EIF2AK3 | 0.3 | 5.84E-25 |
| Pit mucous | Pit mucous | SRCAP | 0.4 | 7.15E-25 |
| Pit mucous | Pit mucous | PAQR8 | 0.32 | 2.20E-24 |
| Pit mucous | Pit mucous | SIDT2 | 0.3 | 5.03E-24 |
| Pit mucous | Pit mucous | MT-ND4 | -0.29 | 8.11E-24 |
| Pit mucous | Pit mucous | ARHGAP12 | 0.37 | 1.30E-23 |
| Pit mucous | Pit mucous | BAIAP2L1 | 0.43 | 9.90E-23 |
| Pit mucous | Pit mucous | CD164 | 0.37 | 1.30E-22 |
| Pit mucous | Pit mucous | ATOX1 | 0.37 | 6.08E-22 |
| Pit mucous | Pit mucous | MIR22HG | 0.39 | 1.31E-21 |
| Pit mucous | Pit mucous | UGDH | 0.4 | 1.90E-21 |
| Pit mucous | Pit mucous | TLE4 | 0.37 | 1.91E-21 |
| Pit mucous | Pit mucous | CHMP2A | 0.35 | 2.47E-21 |
| Pit mucous | Pit mucous | MMP28 | 0.32 | 7.70E-21 |
| Pit mucous | Pit mucous | GALNT5 | 0.31 | 1.05E-20 |
| Pit mucous | Pit mucous | SUN1 | 0.39 | 1.40E-20 |
| Pit mucous | Pit mucous | KRT10 | 0.36 | 2.01E-20 |
| Pit mucous | Pit mucous | PTK2B | 0.31 | 2.22E-20 |
| Pit mucous | Pit mucous | FZD5 | 0.3 | 2.74E-20 |
| Pit mucous | Pit mucous | LPIN2 | 0.34 | 3.12E-20 |
| Pit mucous | Pit mucous | GNB2 | 0.38 | 5.55E-20 |
| Pit mucous | Pit mucous | OCLN | 0.4 | 7.02E-20 |
| Pit mucous | Pit mucous | SRC | 0.34 | 7.38E-20 |
| Pit mucous | Pit mucous | BCAR1 | 0.38 | 9.72E-20 |
| Pit mucous | Pit mucous | B4GALT1 | 0.35 | 1.89E-19 |
| Pit mucous | Pit mucous | MT-ND5 | -0.26 | 2.14E-19 |
| Pit mucous | Pit mucous | LPCAT4 | 0.34 | 2.19E-19 |
| Pit mucous | Pit mucous | TCF7L2 | 0.42 | 2.58E-19 |
| Pit mucous | Pit mucous | VEGFA | 0.38 | 2.81E-19 |
| Pit mucous | Pit mucous | NEDD9 | 0.4 | 4.00E-19 |
| Pit mucous | Pit mucous | DYNLRB1 | 0.37 | 4.01E-19 |
| Pit mucous | Pit mucous | B3GNT5 | 0.4 | 5.51E-19 |
| Pit mucous | Pit mucous | TES | 0.4 | 7.15E-19 |
| Pit mucous | Pit mucous | AES | 0.37 | 7.85E-19 |
| Pit mucous | Pit mucous | ARID3B | 0.34 | 9.28E-19 |
| Pit mucous | Pit mucous | ZNF430 | 0.29 | 9.89E-19 |
| Pit mucous | Pit mucous | ISG20 | 0.26 | 1.53E-18 |
| Pit mucous | Pit mucous | TLNRD1 | 0.31 | 1.62E-18 |
| Pit mucous | Pit mucous | ENTPD5 | 0.3 | 2.95E-18 |
| Pit mucous | Pit mucous | GCLC | 0.36 | 6.12E-18 |
| Pit mucous | Pit mucous | TMEM160 | 0.35 | 7.15E-18 |
| Pit mucous | Pit mucous | PLCD3 | 0.33 | 7.42E-18 |
| Pit mucous | Pit mucous | SCNN1A | 0.35 | 8.64E-18 |
| Pit mucous | Pit mucous | BAD | 0.38 | 9.91E-18 |
| Pit mucous | Pit mucous | RHOC | 0.27 | 3.31E-17 |
| Pit mucous | Pit mucous | ABCC3 | 0.38 | 4.75E-17 |
| Pit mucous | Pit mucous | NPTN | 0.33 | 4.91E-17 |
| Pit mucous | Pit mucous | C11orf86 | -0.27 | 1.59E-16 |
| Pit mucous | Pit mucous | SSR4 | -0.44 | 3.28E-16 |
| Pit mucous | Pit mucous | B3GNT7 | 0.27 | 4.79E-16 |
| Pit mucous | Pit mucous | LAMTOR4 | 0.29 | 5.59E-16 |
| Pit mucous | Pit mucous | DPM2 | 0.45 | 7.80E-16 |
| Pit mucous | Pit mucous | ITGAV | 0.35 | 2.51E-15 |
| Pit mucous | Pit mucous | GSTA1 | -0.25 | 6.81E-15 |
| Pit mucous | Pit mucous | TMEM134 | 0.38 | 7.36E-15 |
| Pit mucous | Pit mucous | BPIFB1 | -0.55 | 8.81E-15 |
| Pit mucous | Pit mucous | PCBP1 | 0.31 | 9.88E-15 |
| Pit mucous | Pit mucous | MT1M | 0.35 | 1.19E-14 |
| Pit mucous | Pit mucous | RNF128 | 0.35 | 1.24E-14 |
| Pit mucous | Pit mucous | RNPEPL1 | 0.36 | 1.40E-14 |
| Pit mucous | Pit mucous | GAREM1 | 0.29 | 2.04E-14 |
| Pit mucous | Pit mucous | BAG1 | 0.36 | 2.86E-14 |
| Pit mucous | Pit mucous | FEM1C | 0.31 | 3.07E-14 |
| Pit mucous | Pit mucous | SYNJ2 | 0.32 | 3.57E-14 |
| Pit mucous | Pit mucous | EDEM3 | 0.34 | 3.85E-14 |
| Pit mucous | Pit mucous | NET1 | 0.39 | 4.48E-14 |
| Pit mucous | Pit mucous | AKAP13 | 0.36 | 4.69E-14 |
| Pit mucous | Pit mucous | SDR16C5 | 0.29 | 5.44E-14 |
| Pit mucous | Pit mucous | F11R | 0.4 | 5.63E-14 |
| Pit mucous | Pit mucous | CHCHD10 | 0.35 | 5.66E-14 |
| Pit mucous | Pit mucous | ETS2 | 0.3 | 8.32E-14 |
| Pit mucous | Pit mucous | TRIOBP | 0.29 | 9.52E-14 |
| Pit mucous | Pit mucous | NR1D1 | 0.32 | 1.14E-13 |
| Pit mucous | Pit mucous | ATP8B1 | 0.37 | 1.26E-13 |
| Pit mucous | Pit mucous | SLC50A1 | 0.33 | 1.38E-13 |
| Pit mucous | Pit mucous | PLLP | 0.36 | 2.18E-13 |
| Pit mucous | Pit mucous | DSP | 0.38 | 2.77E-13 |
| Pit mucous | Pit mucous | MRPL52 | 0.31 | 3.34E-13 |
| Pit mucous | Pit mucous | C1orf116 | 0.33 | 3.83E-13 |
| Pit mucous | Pit mucous | HACD3 | 0.36 | 4.78E-13 |
| Pit mucous | Pit mucous | PTTG1IP | 0.37 | 4.95E-13 |
| Pit mucous | Pit mucous | TSC22D2 | 0.35 | 7.13E-13 |
| Pit mucous | Pit mucous | CTTN | 0.37 | 8.21E-13 |
| Pit mucous | Pit mucous | TMEM246 | 0.26 | 9.66E-13 |
| Pit mucous | Pit mucous | RAPGEFL1 | 0.26 | 9.93E-13 |
| Pit mucous | Pit mucous | TNIP1 | 0.4 | 1.02E-12 |
| Pit mucous | Pit mucous | ARHGDIA | 0.34 | 1.48E-12 |
| Pit mucous | Pit mucous | NDUFA3 | 0.25 | 1.83E-12 |
| Pit mucous | Pit mucous | C3orf52 | 0.33 | 2.17E-12 |
| Pit mucous | Pit mucous | RAB5B | 0.31 | 3.59E-12 |
| Pit mucous | Pit mucous | AHR | 0.34 | 3.60E-12 |
| Pit mucous | Pit mucous | LDLR | 0.34 | 4.70E-12 |
| Pit mucous | Pit mucous | NFKB1 | 0.35 | 6.12E-12 |
| Pit mucous | Pit mucous | LYST | 0.26 | 7.04E-12 |
| Pit mucous | Pit mucous | RAP1A | 0.37 | 8.30E-12 |
| Pit mucous | Pit mucous | CDKN1A | 0.3 | 9.23E-12 |
| Pit mucous | Pit mucous | HERPUD1 | -0.3 | 1.62E-11 |
| Pit mucous | Pit mucous | PNPLA2 | 0.37 | 1.66E-11 |
| Pit mucous | Pit mucous | ITGB6 | 0.26 | 1.97E-11 |
| Pit mucous | Pit mucous | H1F0 | 0.38 | 2.99E-11 |
| Pit mucous | Pit mucous | WEE1 | 0.36 | 4.67E-11 |
| Pit mucous | Pit mucous | TM4SF5 | 0.39 | 7.00E-11 |
| Pit mucous | Pit mucous | GPCPD1 | 0.27 | 9.30E-11 |
| Pit mucous | Pit mucous | MYO5C | 0.26 | 1.01E-10 |
| Pit mucous | Pit mucous | BACE2 | 0.35 | 1.51E-10 |
| Pit mucous | Pit mucous | STXBP2 | 0.38 | 1.60E-10 |
| Pit mucous | Pit mucous | JAG1 | 0.31 | 2.01E-10 |
| Pit mucous | Pit mucous | HNF4A | 0.34 | 2.65E-10 |
| Pit mucous | Pit mucous | RNF103 | 0.31 | 5.00E-10 |
| Pit mucous | Pit mucous | TMCO3 | 0.31 | 1.02E-09 |
| Pit mucous | Pit mucous | RAB11A | 0.29 | 1.18E-09 |
| Pit mucous | Pit mucous | ASAP2 | 0.28 | 1.19E-09 |
| Pit mucous | Pit mucous | TRIM31 | 0.37 | 1.24E-09 |
| Pit mucous | Pit mucous | PRSS8 | 0.36 | 1.36E-09 |
| Pit mucous | Pit mucous | EIF4EBP2 | 0.34 | 1.62E-09 |
| Pit mucous | Pit mucous | DOP1B | 0.25 | 1.67E-09 |
| Pit mucous | Pit mucous | RPS6KA4 | 0.28 | 2.42E-09 |
| Pit mucous | Pit mucous | DNM2 | 0.35 | 2.58E-09 |
| Pit mucous | Pit mucous | ADIPOR2 | 0.36 | 4.66E-09 |
| Pit mucous | Pit mucous | TPM4 | 0.32 | 5.34E-09 |
| Pit mucous | Pit mucous | HPCAL1 | 0.29 | 5.57E-09 |
| Pit mucous | Pit mucous | RNF145 | 0.27 | 5.72E-09 |
| Pit mucous | Pit mucous | ITGB4 | 0.34 | 7.90E-09 |
| Pit mucous | Pit mucous | CYP4F12 | 0.26 | 8.68E-09 |
| Pit mucous | Pit mucous | RETSAT | 0.32 | 2.06E-08 |
| Pit mucous | Pit mucous | ANG | 0.31 | 2.72E-08 |
| Pit mucous | Pit mucous | DDX17 | 0.32 | 2.75E-08 |
| Pit mucous | Pit mucous | LGMN | 0.29 | 2.91E-08 |
| Pit mucous | Pit mucous | SSBP3 | 0.3 | 3.56E-08 |
| Pit mucous | Pit mucous | FRMD4B | 0.33 | 4.19E-08 |
| Pit mucous | Pit mucous | PCSK1N | -0.58 | 5.10E-08 |
| Pit mucous | Pit mucous | SMPDL3A | 0.32 | 5.84E-08 |
| Pit mucous | Pit mucous | ABHD17C | 0.35 | 6.07E-08 |
| Pit mucous | Pit mucous | RIPK4 | 0.28 | 6.54E-08 |
| Pit mucous | Pit mucous | ME1 | 0.25 | 1.31E-07 |
| Pit mucous | Pit mucous | INAVA | 0.34 | 1.72E-07 |
| Pit mucous | Pit mucous | CHP1 | 0.28 | 1.84E-07 |
| Pit mucous | Pit mucous | CLDN23 | 0.28 | 1.92E-07 |
| Pit mucous | Pit mucous | MYH14 | 0.31 | 2.39E-07 |
| Pit mucous | Pit mucous | CYTH1 | 0.3 | 3.09E-07 |
| Pit mucous | Pit mucous | PLAUR | 0.25 | 3.90E-07 |
| Pit mucous | Pit mucous | SIRT7 | 0.3 | 4.55E-07 |
| Pit mucous | Pit mucous | LRRFIP1 | 0.27 | 5.25E-07 |
| Pit mucous | Pit mucous | MSMO1 | 0.31 | 1.44E-06 |
| Pit mucous | Pit mucous | DAZAP2 | 0.28 | 1.45E-06 |
| Pit mucous | Pit mucous | NDRG1 | 0.31 | 2.98E-06 |
| Pit mucous | Pit mucous | UBA1 | 0.34 | 3.37E-06 |
| Pit mucous | Pit mucous | FUT2 | 0.31 | 5.83E-06 |
| Pit mucous | Pit mucous | KIAA1522 | 0.3 | 5.99E-06 |
| Pit mucous | Pit mucous | ODC1 | 0.36 | 6.77E-06 |
| Pit mucous | Pit mucous | TNKS1BP1 | 0.27 | 6.93E-06 |
| Pit mucous | Pit mucous | ARHGEF16 | 0.26 | 7.72E-06 |
| Pit mucous | Pit mucous | PTOV1 | 0.26 | 8.53E-06 |
| Pit mucous | Pit mucous | REG4 | 1.61 | 0 |
| Pit mucous | Pit mucous | MT-CO2 | 1.1 | 0 |
| Pit mucous | Pit mucous | MT-CO1 | 1.09 | 0 |
| Pit mucous | Pit mucous | PHGR1 | 1.09 | 0 |
| Pit mucous | Pit mucous | KRT20 | 0.98 | 0 |
| Pit mucous | Pit mucous | MT-ATP6 | 0.96 | 0 |
| Pit mucous | Pit mucous | MT-CO3 | 0.96 | 0 |
| Pit mucous | Pit mucous | CLDN4 | 0.95 | 0 |
| Pit mucous | Pit mucous | MT-ND5 | 0.95 | 0 |
| Pit mucous | Pit mucous | MT-CYB | 0.94 | 0 |
| Pit mucous | Pit mucous | DMBT1 | 0.93 | 0 |
| Pit mucous | Pit mucous | CES2 | 0.92 | 0 |
| Pit mucous | Pit mucous | KRT19 | 0.92 | 0 |
| Pit mucous | Pit mucous | PRSS3 | 0.91 | 0 |
| Pit mucous | Pit mucous | CEACAM6 | 0.9 | 0 |
| Pit mucous | Pit mucous | CLDN7 | 0.88 | 0 |
| Pit mucous | Pit mucous | CDHR5 | 0.87 | 0 |
| Pit mucous | Pit mucous | MALAT1 | 0.86 | 0 |
| Pit mucous | Pit mucous | S100A6 | 0.86 | 0 |
| Pit mucous | Pit mucous | S100A14 | 0.85 | 0 |
| Pit mucous | Pit mucous | MT-ND1 | 0.84 | 0 |
| Pit mucous | Pit mucous | MT-ND4 | 0.83 | 0 |
| Pit mucous | Pit mucous | LGALS4 | 0.8 | 0 |
| Pit mucous | Pit mucous | MALL | 0.77 | 0 |
| Pit mucous | Pit mucous | CDH17 | 0.72 | 0 |
| Pit mucous | Pit mucous | KRT8 | 0.7 | 0 |
| Pit mucous | Pit mucous | FABP1 | 0.66 | 0 |
| Pit mucous | Pit mucous | GPA33 | 0.65 | 0 |
| Pit mucous | Pit mucous | ANPEP | 0.64 | 0 |
| Pit mucous | Pit mucous | PRAP1 | 0.58 | 0 |
| Pit mucous | Pit mucous | EEF1A1 | -0.74 | 0 |
| Pit mucous | Pit mucous | RPS28 | -0.76 | 0 |
| Pit mucous | Pit mucous | RPS4X | -0.77 | 0 |
| Pit mucous | Pit mucous | RPL10 | -0.78 | 0 |
| Pit mucous | Pit mucous | PTMA | -0.98 | 0 |
| Pit mucous | Pit mucous | PGC | -3.24 | 0 |
| Pit mucous | Pit mucous | GAST | -4.54 | 0 |
| Pit mucous | Pit mucous | MUC13 | 0.6 | 3.56E-298 |
| Pit mucous | Pit mucous | PIGR | 0.78 | 1.67E-297 |
| Pit mucous | Pit mucous | NAPRT | 0.71 | 9.97E-297 |
| Pit mucous | Pit mucous | TMPRSS15 | 0.49 | 1.55E-296 |
| Pit mucous | Pit mucous | ALDOB | 0.39 | 1.70E-294 |
| Pit mucous | Pit mucous | MT-ND2 | 0.7 | 7.00E-289 |
| Pit mucous | Pit mucous | FTL | -0.72 | 4.70E-287 |
| Pit mucous | Pit mucous | LGALS3 | 0.7 | 2.40E-282 |
| Pit mucous | Pit mucous | RPL3 | -0.74 | 3.37E-282 |
| Pit mucous | Pit mucous | RPLP1 | -0.66 | 3.11E-281 |
| Pit mucous | Pit mucous | MUC17 | 0.56 | 2.78E-280 |
| Pit mucous | Pit mucous | KRT7 | 0.83 | 3.38E-278 |
| Pit mucous | Pit mucous | RPL39 | -0.79 | 4.44E-269 |
| Pit mucous | Pit mucous | RPS15A | -0.69 | 1.90E-250 |
| Pit mucous | Pit mucous | GCNT3 | 0.61 | 9.48E-250 |
| Pit mucous | Pit mucous | RPS25 | -0.71 | 2.14E-249 |
| Pit mucous | Pit mucous | AOC1 | 0.59 | 1.05E-243 |
| Pit mucous | Pit mucous | SLC22A18 | 0.59 | 5.37E-243 |
| Pit mucous | Pit mucous | KRT18 | 0.55 | 4.78E-237 |
| Pit mucous | Pit mucous | AGPAT2 | 0.53 | 8.19E-235 |
| Pit mucous | Pit mucous | TAGLN2 | -0.94 | 9.83E-232 |
| Pit mucous | Pit mucous | SULT1A1 | 0.48 | 2.07E-227 |
| Pit mucous | Pit mucous | TFF3 | 1.2 | 3.67E-227 |
| Pit mucous | Pit mucous | GKN1 | -3 | 4.77E-225 |
| Pit mucous | Pit mucous | S100A10 | 0.6 | 2.32E-221 |
| Pit mucous | Pit mucous | C15orf48 | 0.67 | 7.01E-219 |
| Pit mucous | Pit mucous | RPL9 | -0.6 | 2.75E-218 |
| Pit mucous | Pit mucous | MISP | 0.61 | 4.92E-217 |
| Pit mucous | Pit mucous | RPL41 | -0.52 | 5.58E-217 |
| Pit mucous | Pit mucous | MT-ND3 | 0.52 | 8.33E-216 |
| Pit mucous | Pit mucous | RPL32 | -0.6 | 1.50E-215 |
| Pit mucous | Pit mucous | EIF1 | -0.56 | 5.45E-215 |
| Pit mucous | Pit mucous | SI | 0.4 | 1.77E-214 |
| Pit mucous | Pit mucous | SUMO2 | -0.79 | 5.64E-212 |
| Pit mucous | Pit mucous | RPS14 | -0.5 | 1.99E-211 |
| Pit mucous | Pit mucous | RPL23A | -0.63 | 1.33E-209 |
| Pit mucous | Pit mucous | UQCRC1 | 0.6 | 3.68E-209 |
| Pit mucous | Pit mucous | FAU | -0.5 | 3.48E-208 |
| Pit mucous | Pit mucous | MYO15B | 0.49 | 2.87E-203 |
| Pit mucous | Pit mucous | MSMB | -1.55 | 5.01E-200 |
| Pit mucous | Pit mucous | RPS10 | -0.69 | 4.70E-199 |
| Pit mucous | Pit mucous | RPS27A | -0.54 | 4.91E-197 |
| Pit mucous | Pit mucous | GKN2 | -2.43 | 4.48E-196 |
| Pit mucous | Pit mucous | RPL17 | -0.87 | 2.51E-195 |
| Pit mucous | Pit mucous | RPLP2 | -0.54 | 1.64E-193 |
| Pit mucous | Pit mucous | RPL34 | -0.58 | 5.63E-191 |
| Pit mucous | Pit mucous | RPL15 | -0.6 | 1.26E-190 |
| Pit mucous | Pit mucous | RPL35A | -0.56 | 4.73E-189 |
| Pit mucous | Pit mucous | CDX1 | 0.4 | 1.24E-182 |
| Pit mucous | Pit mucous | CIRBP | -0.88 | 7.60E-180 |
| Pit mucous | Pit mucous | FOSB | -1.08 | 7.87E-180 |
| Pit mucous | Pit mucous | RPS3A | -0.58 | 1.55E-179 |
| Pit mucous | Pit mucous | TMEM54 | 0.58 | 3.38E-178 |
| Pit mucous | Pit mucous | ASS1 | 0.57 | 6.27E-178 |
| Pit mucous | Pit mucous | NPM1 | -0.79 | 1.45E-177 |
| Pit mucous | Pit mucous | RPL5 | -0.68 | 2.68E-176 |
| Pit mucous | Pit mucous | ATP5F1D | 0.64 | 2.00E-174 |
| Pit mucous | Pit mucous | SMIM24 | 0.51 | 2.29E-174 |
| Pit mucous | Pit mucous | C11orf86 | 0.31 | 9.42E-174 |
| Pit mucous | Pit mucous | TMSB4X | -0.82 | 3.48E-173 |
| Pit mucous | Pit mucous | CEACAM5 | 0.54 | 4.42E-172 |
| Pit mucous | Pit mucous | TFF2 | -1.66 | 3.44E-170 |
| Pit mucous | Pit mucous | ASL | 0.53 | 4.97E-170 |
| Pit mucous | Pit mucous | TM4SF20 | 0.34 | 1.43E-169 |
| Pit mucous | Pit mucous | CLDN3 | 0.53 | 1.83E-169 |
| Pit mucous | Pit mucous | TESC | -0.92 | 1.33E-168 |
| Pit mucous | Pit mucous | RPS7 | -0.57 | 1.28E-167 |
| Pit mucous | Pit mucous | EPCAM | 0.51 | 9.40E-167 |
| Pit mucous | Pit mucous | CTSD | 0.58 | 1.66E-166 |
| Pit mucous | Pit mucous | CXCL17 | -1.01 | 1.04E-164 |
| Pit mucous | Pit mucous | H3F3A | -0.53 | 6.20E-164 |
| Pit mucous | Pit mucous | MAP2K2 | 0.48 | 2.31E-163 |
| Pit mucous | Pit mucous | RPS12 | -0.59 | 8.81E-160 |
| Pit mucous | Pit mucous | SMIM31 | 0.44 | 6.18E-159 |
| Pit mucous | Pit mucous | SPINK1 | -1.21 | 7.49E-158 |
| Pit mucous | Pit mucous | H3F3B | -0.54 | 6.44E-155 |
| Pit mucous | Pit mucous | FOS | -1.38 | 9.19E-155 |
| Pit mucous | Pit mucous | CDHR2 | 0.4 | 1.99E-154 |
| Pit mucous | Pit mucous | PKP3 | 0.49 | 2.33E-153 |
| Pit mucous | Pit mucous | RPS3 | -0.46 | 1.38E-152 |
| Pit mucous | Pit mucous | LYZ | -1.47 | 7.91E-152 |
| Pit mucous | Pit mucous | DGAT1 | 0.43 | 1.43E-151 |
| Pit mucous | Pit mucous | SHD | 0.35 | 4.74E-151 |
| Pit mucous | Pit mucous | HNRNPA1 | -0.62 | 6.36E-151 |
| Pit mucous | Pit mucous | RPL36A | -0.7 | 1.25E-150 |
| Pit mucous | Pit mucous | NBEAL1 | -0.73 | 2.55E-150 |
| Pit mucous | Pit mucous | ATP6V1G1 | -0.64 | 4.46E-150 |
| Pit mucous | Pit mucous | NDUFS7 | 0.56 | 5.07E-150 |
| Pit mucous | Pit mucous | RPL12 | -0.47 | 8.00E-149 |
| Pit mucous | Pit mucous | MYO7B | 0.35 | 1.88E-148 |
| Pit mucous | Pit mucous | RPL4 | -0.63 | 6.09E-145 |
| Pit mucous | Pit mucous | ADH4 | 0.43 | 6.28E-145 |
| Pit mucous | Pit mucous | HMGN2 | -0.82 | 9.35E-144 |
| Pit mucous | Pit mucous | TMEM258 | -0.6 | 4.01E-143 |
| Pit mucous | Pit mucous | SLC26A3 | 0.45 | 7.11E-143 |
| Pit mucous | Pit mucous | SLC39A5 | 0.41 | 9.67E-142 |
| Pit mucous | Pit mucous | OTOP3 | 0.31 | 1.16E-140 |
| Pit mucous | Pit mucous | RPL24 | -0.51 | 1.71E-140 |
| Pit mucous | Pit mucous | HEPH | 0.36 | 1.44E-138 |
| Pit mucous | Pit mucous | RPS27 | -0.5 | 1.53E-138 |
| Pit mucous | Pit mucous | TXNDC17 | 0.5 | 4.45E-137 |
| Pit mucous | Pit mucous | CAMK2N1 | 0.43 | 7.87E-137 |
| Pit mucous | Pit mucous | AMN | 0.34 | 7.86E-136 |
| Pit mucous | Pit mucous | NAP1L1 | -0.66 | 1.07E-135 |
| Pit mucous | Pit mucous | GPT | 0.34 | 1.66E-135 |
| Pit mucous | Pit mucous | ACTG1 | -0.49 | 2.10E-135 |
| Pit mucous | Pit mucous | RPL31 | -0.52 | 3.60E-135 |
| Pit mucous | Pit mucous | C6orf141 | 0.47 | 1.10E-134 |
| Pit mucous | Pit mucous | ESPN | 0.28 | 1.34E-134 |
| Pit mucous | Pit mucous | RPS20 | -0.51 | 2.74E-134 |
| Pit mucous | Pit mucous | SFN | 0.43 | 5.23E-134 |
| Pit mucous | Pit mucous | ALDH3A1 | -0.88 | 7.41E-134 |
| Pit mucous | Pit mucous | RPL19 | -0.42 | 3.72E-133 |
| Pit mucous | Pit mucous | PFDN5 | -0.48 | 3.99E-133 |
| Pit mucous | Pit mucous | RPS6 | -0.44 | 3.91E-131 |
| Pit mucous | Pit mucous | TMSB10 | -0.46 | 1.19E-130 |
| Pit mucous | Pit mucous | MYH14 | 0.45 | 2.29E-130 |
| Pit mucous | Pit mucous | RPS23 | -0.44 | 2.75E-130 |
| Pit mucous | Pit mucous | COX7B | 0.62 | 2.80E-130 |
| Pit mucous | Pit mucous | SERPINB6 | 0.44 | 2.91E-130 |
| Pit mucous | Pit mucous | RPL11 | -0.4 | 1.49E-129 |
| Pit mucous | Pit mucous | MEP1A | 0.28 | 6.09E-129 |
| Pit mucous | Pit mucous | SDCBP2 | 0.4 | 5.55E-127 |
| Pit mucous | Pit mucous | RPL14 | -0.46 | 5.62E-127 |
| Pit mucous | Pit mucous | ZFP36 | -1.14 | 1.18E-125 |
| Pit mucous | Pit mucous | GLUL | -0.78 | 9.84E-125 |
| Pit mucous | Pit mucous | ONECUT2 | 0.31 | 4.66E-123 |
| Pit mucous | Pit mucous | ATP5MC2 | -0.5 | 5.12E-123 |
| Pit mucous | Pit mucous | SERPINB5 | 0.47 | 8.84E-123 |
| Pit mucous | Pit mucous | GALM | 0.39 | 3.80E-122 |
| Pit mucous | Pit mucous | RPL26 | -0.47 | 3.63E-121 |
| Pit mucous | Pit mucous | RPL18 | -0.38 | 7.46E-120 |
| Pit mucous | Pit mucous | ABHD11-AS1 | 0.32 | 1.05E-119 |
| Pit mucous | Pit mucous | RPS13 | -0.42 | 2.72E-119 |
| Pit mucous | Pit mucous | TM6SF2 | 0.3 | 3.02E-119 |
| Pit mucous | Pit mucous | H2AFZ | -0.76 | 5.29E-119 |
| Pit mucous | Pit mucous | CYC1 | 0.47 | 7.02E-119 |
| Pit mucous | Pit mucous | TRMT112 | -0.62 | 1.84E-118 |
| Pit mucous | Pit mucous | RPS8 | -0.45 | 4.00E-118 |
| Pit mucous | Pit mucous | MARCKSL1 | -0.67 | 6.62E-117 |
| Pit mucous | Pit mucous | TMEM45B | 0.4 | 4.06E-116 |
| Pit mucous | Pit mucous | AC020656.1 | -1.54 | 8.82E-115 |
| Pit mucous | Pit mucous | SEC61G | -0.55 | 4.29E-114 |
| Pit mucous | Pit mucous | MOGAT3 | 0.28 | 1.26E-113 |
| Pit mucous | Pit mucous | SST | -2.74 | 3.94E-113 |
| Pit mucous | Pit mucous | SPINK4 | 1.54 | 1.47E-112 |
| Pit mucous | Pit mucous | TPT1 | -0.43 | 1.64E-112 |
| Pit mucous | Pit mucous | RPL27 | -0.48 | 5.87E-112 |
| Pit mucous | Pit mucous | S100A16 | 0.42 | 2.13E-111 |
| Pit mucous | Pit mucous | CLU | -0.96 | 4.73E-109 |
| Pit mucous | Pit mucous | EGR1 | -0.92 | 5.44E-109 |
| Pit mucous | Pit mucous | HSD17B2 | 0.3 | 1.51E-108 |
| Pit mucous | Pit mucous | UNC13D | 0.28 | 1.10E-107 |
| Pit mucous | Pit mucous | BTF3 | -0.44 | 5.07E-107 |
| Pit mucous | Pit mucous | TSPAN3 | 0.46 | 1.50E-106 |
| Pit mucous | Pit mucous | MT-ND4L | 0.45 | 1.19E-104 |
| Pit mucous | Pit mucous | ANXA10 | 0.45 | 2.70E-104 |
| Pit mucous | Pit mucous | SULT1A2 | 0.26 | 1.29E-103 |
| Pit mucous | Pit mucous | DUT | -0.54 | 3.08E-103 |
| Pit mucous | Pit mucous | TJP3 | 0.34 | 3.57E-103 |
| Pit mucous | Pit mucous | TRIM15 | 0.32 | 4.40E-103 |
| Pit mucous | Pit mucous | CALM2 | -0.44 | 5.53E-101 |
| Pit mucous | Pit mucous | FABP5 | -0.97 | 6.30E-101 |
| Pit mucous | Pit mucous | DST | 0.42 | 6.75E-101 |
| Pit mucous | Pit mucous | RPL38 | -0.49 | 7.74E-101 |
| Pit mucous | Pit mucous | UPP1 | 0.37 | 8.55E-101 |
| Pit mucous | Pit mucous | PI3 | 0.32 | 1.58E-100 |
| Pit mucous | Pit mucous | RPL7A | -0.41 | 2.31E-100 |
| Pit mucous | Pit mucous | COL17A1 | 0.31 | 2.47E-100 |
| Pit mucous | Pit mucous | RPL6 | -0.46 | 6.81E-99 |
| Pit mucous | Pit mucous | RPS29 | -0.42 | 9.44E-99 |
| Pit mucous | Pit mucous | BTG2 | -0.71 | 1.78E-98 |
| Pit mucous | Pit mucous | BEX3 | -0.58 | 4.94E-97 |
| Pit mucous | Pit mucous | HSP90AB1 | -0.5 | 1.36E-96 |
| Pit mucous | Pit mucous | OST4 | -0.44 | 3.34E-96 |
| Pit mucous | Pit mucous | RPL10A | -0.43 | 3.69E-96 |
| Pit mucous | Pit mucous | SULT1B1 | 0.31 | 5.10E-96 |
| Pit mucous | Pit mucous | TSPAN1 | 0.47 | 6.29E-96 |
| Pit mucous | Pit mucous | COMMD6 | -0.5 | 6.38E-96 |
| Pit mucous | Pit mucous | CHP2 | 0.28 | 3.52E-95 |
| Pit mucous | Pit mucous | TMC5 | 0.49 | 1.77E-94 |
| Pit mucous | Pit mucous | PPDPF | -0.49 | 1.87E-94 |
| Pit mucous | Pit mucous | CYSTM1 | -0.57 | 1.88E-94 |
| Pit mucous | Pit mucous | RPLP0 | -0.38 | 2.16E-94 |
| Pit mucous | Pit mucous | FAM3D | 0.31 | 2.42E-94 |
| Pit mucous | Pit mucous | SLC6A8 | 0.28 | 3.34E-94 |
| Pit mucous | Pit mucous | FXYD5 | -0.56 | 4.85E-94 |
| Pit mucous | Pit mucous | ISG15 | 0.62 | 8.36E-94 |
| Pit mucous | Pit mucous | UBA52 | -0.34 | 2.29E-92 |
| Pit mucous | Pit mucous | RPL7 | -0.32 | 3.16E-92 |
| Pit mucous | Pit mucous | PLEC | 0.43 | 2.29E-91 |
| Pit mucous | Pit mucous | RHOD | 0.32 | 3.42E-91 |
| Pit mucous | Pit mucous | FUOM | 0.35 | 5.26E-91 |
| Pit mucous | Pit mucous | CDKN2B | 0.25 | 5.50E-91 |
| Pit mucous | Pit mucous | DDX5 | -0.48 | 1.16E-90 |
| Pit mucous | Pit mucous | SLC12A2 | -0.54 | 1.49E-90 |
| Pit mucous | Pit mucous | RPS21 | -0.46 | 5.63E-90 |
| Pit mucous | Pit mucous | RACK1 | -0.38 | 1.04E-89 |
| Pit mucous | Pit mucous | ANXA2 | 0.33 | 3.05E-89 |
| Pit mucous | Pit mucous | RPL35 | -0.31 | 4.19E-89 |
| Pit mucous | Pit mucous | GSDMD | 0.39 | 1.90E-88 |
| Pit mucous | Pit mucous | HMGB1 | -0.53 | 2.88E-88 |
| Pit mucous | Pit mucous | HES1 | 0.44 | 1.06E-87 |
| Pit mucous | Pit mucous | SLC6A20 | 0.28 | 1.12E-87 |
| Pit mucous | Pit mucous | GDPD3 | 0.3 | 5.56E-87 |
| Pit mucous | Pit mucous | EIF4A2 | -0.51 | 1.53E-86 |
| Pit mucous | Pit mucous | MORF4L1 | -0.53 | 4.68E-86 |
| Pit mucous | Pit mucous | JUNB | -1 | 8.30E-86 |
| Pit mucous | Pit mucous | VIM | -1.3 | 3.91E-85 |
| Pit mucous | Pit mucous | ATP5MC3 | 0.37 | 9.44E-85 |
| Pit mucous | Pit mucous | RPL37 | -0.5 | 1.50E-84 |
| Pit mucous | Pit mucous | RSL24D1 | -0.53 | 2.39E-84 |
| Pit mucous | Pit mucous | EIF3E | -0.52 | 3.04E-84 |
| Pit mucous | Pit mucous | GABARAP | -0.52 | 4.12E-84 |
| Pit mucous | Pit mucous | SRSF3 | -0.52 | 9.40E-84 |
| Pit mucous | Pit mucous | RPL21 | -0.32 | 2.30E-83 |
| Pit mucous | Pit mucous | IER2 | -0.68 | 5.29E-83 |
| Pit mucous | Pit mucous | SERF2 | -0.34 | 6.46E-83 |
| Pit mucous | Pit mucous | CLDN18 | -0.73 | 1.60E-82 |
| Pit mucous | Pit mucous | RPSA | -0.4 | 4.92E-82 |
| Pit mucous | Pit mucous | RPL13A | -0.29 | 5.55E-82 |
| Pit mucous | Pit mucous | LAPTM4A | -0.55 | 7.60E-82 |
| Pit mucous | Pit mucous | RPL27A | -0.31 | 1.51E-81 |
| Pit mucous | Pit mucous | CD44 | -0.55 | 1.83E-81 |
| Pit mucous | Pit mucous | FKBP1A | -0.49 | 3.58E-81 |
| Pit mucous | Pit mucous | CST3 | -0.47 | 3.58E-81 |
| Pit mucous | Pit mucous | TRIM31 | 0.37 | 3.78E-81 |
| Pit mucous | Pit mucous | ETFB | 0.38 | 1.67E-80 |
| Pit mucous | Pit mucous | PABPC1 | -0.44 | 3.96E-80 |
| Pit mucous | Pit mucous | VILL | 0.29 | 4.36E-80 |
| Pit mucous | Pit mucous | JTB | -0.45 | 5.57E-80 |
| Pit mucous | Pit mucous | GADD45B | -0.94 | 9.86E-80 |
| Pit mucous | Pit mucous | STMN1 | -0.65 | 1.20E-79 |
| Pit mucous | Pit mucous | NACA | -0.39 | 2.24E-79 |
| Pit mucous | Pit mucous | MYO1A | 0.27 | 2.84E-79 |
| Pit mucous | Pit mucous | CHCHD10 | 0.37 | 3.24E-79 |
| Pit mucous | Pit mucous | SKP1 | -0.44 | 4.35E-79 |
| Pit mucous | Pit mucous | RPL13 | -0.3 | 6.63E-79 |
| Pit mucous | Pit mucous | MT1X | -0.64 | 1.32E-78 |
| Pit mucous | Pit mucous | HLA-B | -0.47 | 1.86E-78 |
| Pit mucous | Pit mucous | HMGN3 | -0.5 | 3.37E-78 |
| Pit mucous | Pit mucous | RPL36 | -0.31 | 6.19E-78 |
| Pit mucous | Pit mucous | MAPK3 | 0.32 | 1.36E-77 |
| Pit mucous | Pit mucous | YWHAE | -0.45 | 1.62E-77 |
| Pit mucous | Pit mucous | VIL1 | 0.28 | 2.85E-77 |
| Pit mucous | Pit mucous | CORO2A | 0.3 | 4.34E-77 |
| Pit mucous | Pit mucous | SLC4A7 | 0.26 | 1.62E-76 |
| Pit mucous | Pit mucous | RPL30 | -0.39 | 1.65E-76 |
| Pit mucous | Pit mucous | EID1 | -0.57 | 2.59E-76 |
| Pit mucous | Pit mucous | RPL23 | -0.47 | 2.59E-76 |
| Pit mucous | Pit mucous | MT-ATP8 | 0.35 | 3.61E-76 |
| Pit mucous | Pit mucous | NDUFA1 | 0.38 | 5.39E-76 |
| Pit mucous | Pit mucous | CYP3A5 | 0.36 | 1.16E-75 |
| Pit mucous | Pit mucous | SERP1 | -0.42 | 8.75E-75 |
| Pit mucous | Pit mucous | MARVELD3 | 0.27 | 1.85E-74 |
| Pit mucous | Pit mucous | RPS2 | -0.38 | 5.66E-74 |
| Pit mucous | Pit mucous | TCEAL9 | -0.56 | 1.24E-73 |
| Pit mucous | Pit mucous | PCBP2 | -0.46 | 1.59E-73 |
| Pit mucous | Pit mucous | EIF1AX | -0.5 | 2.40E-73 |
| Pit mucous | Pit mucous | ETHE1 | 0.45 | 2.99E-73 |
| Pit mucous | Pit mucous | COX5B | 0.42 | 3.26E-73 |
| Pit mucous | Pit mucous | RPS24 | -0.37 | 7.23E-73 |
| Pit mucous | Pit mucous | RHOF | 0.28 | 7.42E-73 |
| Pit mucous | Pit mucous | SPCS2 | -0.55 | 1.12E-72 |
| Pit mucous | Pit mucous | SAT1 | -0.47 | 1.13E-72 |
| Pit mucous | Pit mucous | MUC2 | 1.04 | 1.16E-71 |
| Pit mucous | Pit mucous | PLCD3 | 0.27 | 3.53E-71 |
| Pit mucous | Pit mucous | LINC02404 | 0.26 | 3.73E-71 |
| Pit mucous | Pit mucous | ZFAS1 | -0.49 | 6.04E-71 |
| Pit mucous | Pit mucous | RNASE1 | -0.74 | 7.29E-71 |
| Pit mucous | Pit mucous | ACTB | -0.39 | 9.15E-71 |
| Pit mucous | Pit mucous | CUTA | -0.48 | 9.92E-71 |
| Pit mucous | Pit mucous | RPL22L1 | -0.5 | 1.93E-70 |
| Pit mucous | Pit mucous | TOMM7 | -0.41 | 4.46E-70 |
| Pit mucous | Pit mucous | DUSP1 | -0.71 | 5.79E-70 |
| Pit mucous | Pit mucous | EIF4A1 | -0.41 | 8.10E-70 |
| Pit mucous | Pit mucous | DSC2 | 0.27 | 9.34E-70 |
| Pit mucous | Pit mucous | NFKBIA | -0.61 | 9.38E-70 |
| Pit mucous | Pit mucous | ERH | -0.45 | 1.13E-69 |
| Pit mucous | Pit mucous | CLDN15 | 0.3 | 2.28E-69 |
| Pit mucous | Pit mucous | NUCKS1 | -0.49 | 3.05E-69 |
| Pit mucous | Pit mucous | C19orf33 | 0.32 | 6.55E-69 |
| Pit mucous | Pit mucous | RPS5 | -0.3 | 9.81E-69 |
| Pit mucous | Pit mucous | TMEM123 | -0.42 | 4.68E-68 |
| Pit mucous | Pit mucous | LGALS1 | -1.32 | 5.40E-68 |
| Pit mucous | Pit mucous | MVP | 0.3 | 8.80E-68 |
| Pit mucous | Pit mucous | CXCL8 | -1.26 | 4.12E-67 |
| Pit mucous | Pit mucous | RPL18A | -0.25 | 4.82E-67 |
| Pit mucous | Pit mucous | HMGN1 | -0.47 | 5.60E-67 |
| Pit mucous | Pit mucous | CD99 | -0.5 | 5.95E-67 |
| Pit mucous | Pit mucous | HNRNPA0 | -0.48 | 1.58E-65 |
| Pit mucous | Pit mucous | HERPUD1 | -0.74 | 1.64E-65 |
| Pit mucous | Pit mucous | VKORC1 | -0.37 | 1.85E-65 |
| Pit mucous | Pit mucous | ZFP36L1 | -0.6 | 8.12E-65 |
| Pit mucous | Pit mucous | TMEM59 | -0.44 | 9.92E-65 |
| Pit mucous | Pit mucous | CBLC | 0.31 | 1.23E-64 |
| Pit mucous | Pit mucous | NDUFA13 | 0.34 | 1.41E-64 |
| Pit mucous | Pit mucous | TACSTD2 | -0.62 | 1.62E-64 |
| Pit mucous | Pit mucous | BRK1 | -0.41 | 1.99E-64 |
| Pit mucous | Pit mucous | NDUFS6 | 0.34 | 4.69E-64 |
| Pit mucous | Pit mucous | KLF10 | -0.47 | 9.38E-64 |
| Pit mucous | Pit mucous | ITLN1 | 0.6 | 2.22E-63 |
| Pit mucous | Pit mucous | HNRNPDL | -0.45 | 2.88E-63 |
| Pit mucous | Pit mucous | LMO7 | 0.31 | 2.96E-63 |
| Pit mucous | Pit mucous | MUC6 | -1.42 | 4.25E-63 |
| Pit mucous | Pit mucous | SLC44A4 | 0.3 | 5.68E-63 |
| Pit mucous | Pit mucous | SET | -0.46 | 6.80E-63 |
| Pit mucous | Pit mucous | BAIAP2L2 | 0.27 | 6.84E-63 |
| Pit mucous | Pit mucous | PNRC1 | -0.49 | 7.02E-63 |
| Pit mucous | Pit mucous | B2M | -0.44 | 1.93E-62 |
| Pit mucous | Pit mucous | PYCARD | 0.4 | 5.78E-62 |
| Pit mucous | Pit mucous | RHOC | 0.31 | 1.19E-61 |
| Pit mucous | Pit mucous | C11orf58 | -0.45 | 1.65E-61 |
| Pit mucous | Pit mucous | TMA7 | -0.31 | 2.43E-61 |
| Pit mucous | Pit mucous | NDUFB7 | 0.37 | 4.20E-61 |
| Pit mucous | Pit mucous | TSC22D3 | -1.04 | 6.06E-61 |
| Pit mucous | Pit mucous | NDUFS2 | 0.32 | 6.26E-61 |
| Pit mucous | Pit mucous | IER3 | -0.59 | 6.26E-61 |
| Pit mucous | Pit mucous | KRTCAP2 | -0.47 | 6.74E-61 |
| Pit mucous | Pit mucous | RANBP1 | -0.48 | 9.98E-61 |
| Pit mucous | Pit mucous | CXCL2 | -0.96 | 2.08E-60 |
| Pit mucous | Pit mucous | FLNB | 0.32 | 2.58E-60 |
| Pit mucous | Pit mucous | ACTN4 | 0.29 | 3.10E-60 |
| Pit mucous | Pit mucous | ABCC3 | 0.26 | 4.12E-60 |
| Pit mucous | Pit mucous | SUB1 | -0.4 | 4.87E-60 |
| Pit mucous | Pit mucous | VAPA | -0.42 | 5.33E-60 |
| Pit mucous | Pit mucous | SARAF | -0.48 | 6.19E-60 |
| Pit mucous | Pit mucous | RPL22 | -0.33 | 8.57E-60 |
| Pit mucous | Pit mucous | TSPAN8 | 0.26 | 1.79E-59 |
| Pit mucous | Pit mucous | NPC2 | -0.46 | 2.60E-59 |
| Pit mucous | Pit mucous | GPX2 | -0.61 | 4.21E-59 |
| Pit mucous | Pit mucous | TMED2 | -0.37 | 6.47E-59 |
| Pit mucous | Pit mucous | ATP5F1E | -0.3 | 6.75E-59 |
| Pit mucous | Pit mucous | HNRNPC | -0.41 | 3.07E-58 |
| Pit mucous | Pit mucous | OSTC | -0.38 | 5.13E-58 |
| Pit mucous | Pit mucous | AKR1C3 | 0.4 | 6.42E-58 |
| Pit mucous | Pit mucous | CAST | 0.29 | 8.88E-58 |
| Pit mucous | Pit mucous | MRPL12 | 0.38 | 1.18E-57 |
| Pit mucous | Pit mucous | DMKN | -0.31 | 1.85E-57 |
| Pit mucous | Pit mucous | MDK | 0.26 | 4.60E-57 |
| Pit mucous | Pit mucous | AK4 | 0.26 | 5.03E-57 |
| Pit mucous | Pit mucous | TUBA1A | -0.76 | 9.83E-57 |
| Pit mucous | Pit mucous | CRIP2 | -0.51 | 1.93E-56 |
| Pit mucous | Pit mucous | SEC61B | -0.37 | 2.74E-56 |
| Pit mucous | Pit mucous | RAN | -0.43 | 4.21E-56 |
| Pit mucous | Pit mucous | KIAA1324 | -0.38 | 6.09E-56 |
| Pit mucous | Pit mucous | TRIM54 | 0.28 | 6.16E-56 |
| Pit mucous | Pit mucous | SNRPD2 | -0.38 | 1.74E-55 |
| Pit mucous | Pit mucous | ATP6V0E1 | -0.4 | 2.92E-55 |
| Pit mucous | Pit mucous | RPS18 | -0.26 | 3.14E-55 |
| Pit mucous | Pit mucous | RBMX | -0.42 | 5.61E-55 |
| Pit mucous | Pit mucous | HADHA | 0.3 | 1.16E-54 |
| Pit mucous | Pit mucous | NFE2L2 | -0.43 | 1.67E-54 |
| Pit mucous | Pit mucous | NCL | -0.41 | 3.25E-54 |
| Pit mucous | Pit mucous | SVIP | -0.46 | 3.46E-54 |
| Pit mucous | Pit mucous | RBP2 | -0.44 | 4.54E-54 |
| Pit mucous | Pit mucous | MT1E | -0.56 | 5.95E-54 |
| Pit mucous | Pit mucous | CA9 | -0.36 | 6.56E-54 |
| Pit mucous | Pit mucous | LGMN | -0.39 | 8.59E-54 |
| Pit mucous | Pit mucous | SNRPE | -0.39 | 8.74E-54 |
| Pit mucous | Pit mucous | SNHG8 | -0.42 | 1.63E-53 |
| Pit mucous | Pit mucous | SCGB2A1 | -0.59 | 1.98E-53 |
| Pit mucous | Pit mucous | INF2 | 0.29 | 6.66E-53 |
| Pit mucous | Pit mucous | DAD1 | -0.38 | 8.65E-53 |
| Pit mucous | Pit mucous | C6orf48 | -0.39 | 9.60E-53 |
| Pit mucous | Pit mucous | TMEM230 | -0.39 | 1.20E-52 |
| Pit mucous | Pit mucous | SELENOM | -0.48 | 1.22E-52 |
| Pit mucous | Pit mucous | SPATS2L | 0.34 | 1.40E-52 |
| Pit mucous | Pit mucous | GMDS | 0.31 | 1.48E-52 |
| Pit mucous | Pit mucous | CYBA | 0.26 | 1.66E-52 |
| Pit mucous | Pit mucous | HINT1 | -0.3 | 3.83E-52 |
| Pit mucous | Pit mucous | C7orf50 | -0.32 | 6.08E-52 |
| Pit mucous | Pit mucous | HNRNPF | -0.41 | 1.10E-51 |
| Pit mucous | Pit mucous | KLK11 | -0.32 | 1.27E-51 |
| Pit mucous | Pit mucous | POLD4 | 0.3 | 1.44E-51 |
| Pit mucous | Pit mucous | RRBP1 | 0.3 | 1.61E-51 |
| Pit mucous | Pit mucous | TUBB | -0.52 | 2.81E-51 |
| Pit mucous | Pit mucous | MUC12 | 0.29 | 3.19E-51 |
| Pit mucous | Pit mucous | SIVA1 | -0.39 | 3.28E-51 |
| Pit mucous | Pit mucous | ACAA1 | 0.27 | 1.06E-50 |
| Pit mucous | Pit mucous | PRSS23 | -0.47 | 1.30E-50 |
| Pit mucous | Pit mucous | SEC11A | -0.36 | 1.39E-50 |
| Pit mucous | Pit mucous | STAP2 | 0.28 | 2.35E-50 |
| Pit mucous | Pit mucous | PPP1CB | -0.42 | 2.96E-50 |
| Pit mucous | Pit mucous | ITM2B | -0.45 | 3.19E-50 |
| Pit mucous | Pit mucous | KRT10 | -0.44 | 5.20E-50 |
| Pit mucous | Pit mucous | ST14 | 0.28 | 6.43E-50 |
| Pit mucous | Pit mucous | VAMP2 | -0.45 | 7.17E-50 |
| Pit mucous | Pit mucous | PTGES3 | -0.38 | 7.96E-50 |
| Pit mucous | Pit mucous | SRGN | -1.03 | 1.79E-49 |
| Pit mucous | Pit mucous | MXD1 | 0.25 | 2.13E-49 |
| Pit mucous | Pit mucous | PPP1R15A | -0.46 | 2.48E-49 |
| Pit mucous | Pit mucous | TMEM134 | -0.4 | 4.35E-49 |
| Pit mucous | Pit mucous | DNAJB1 | -0.65 | 4.75E-49 |
| Pit mucous | Pit mucous | BTG1 | -0.58 | 2.58E-48 |
| Pit mucous | Pit mucous | NFIC | -0.3 | 2.77E-48 |
| Pit mucous | Pit mucous | TBCA | -0.36 | 4.03E-48 |
| Pit mucous | Pit mucous | VSIG1 | -0.47 | 4.19E-48 |
| Pit mucous | Pit mucous | SCAND1 | 0.28 | 4.26E-48 |
| Pit mucous | Pit mucous | SEC62 | -0.42 | 5.06E-48 |
| Pit mucous | Pit mucous | GPRC5A | 0.33 | 7.18E-48 |
| Pit mucous | Pit mucous | SRSF2 | -0.43 | 7.62E-48 |
| Pit mucous | Pit mucous | RHOB | -0.52 | 8.13E-48 |
| Pit mucous | Pit mucous | LAMA3 | 0.3 | 8.47E-48 |
| Pit mucous | Pit mucous | TCEAL4 | -0.29 | 9.05E-48 |
| Pit mucous | Pit mucous | NPDC1 | -0.35 | 9.67E-48 |
| Pit mucous | Pit mucous | SUMO1 | -0.36 | 1.24E-47 |
| Pit mucous | Pit mucous | TP53I3 | 0.27 | 1.30E-47 |
| Pit mucous | Pit mucous | NHP2 | -0.37 | 1.37E-47 |
| Pit mucous | Pit mucous | GPX4 | -0.39 | 3.57E-47 |
| Pit mucous | Pit mucous | MEAF6 | -0.32 | 6.22E-47 |
| Pit mucous | Pit mucous | CDK4 | -0.26 | 8.74E-47 |
| Pit mucous | Pit mucous | MIF | -0.33 | 1.31E-46 |
| Pit mucous | Pit mucous | EEF2 | -0.31 | 1.32E-46 |
| Pit mucous | Pit mucous | GATM | -0.33 | 1.48E-46 |
| Pit mucous | Pit mucous | CD59 | -0.39 | 1.57E-46 |
| Pit mucous | Pit mucous | MZT2B | -0.39 | 2.48E-46 |
| Pit mucous | Pit mucous | AURKAIP1 | 0.3 | 3.07E-46 |
| Pit mucous | Pit mucous | EMP3 | -0.5 | 1.00E-45 |
| Pit mucous | Pit mucous | KCNE2 | -0.27 | 1.18E-45 |
| Pit mucous | Pit mucous | SNX3 | -0.36 | 1.35E-45 |
| Pit mucous | Pit mucous | GTF3A | -0.31 | 1.43E-45 |
| Pit mucous | Pit mucous | IMPDH2 | -0.38 | 2.29E-45 |
| Pit mucous | Pit mucous | PCSK1N | -1.06 | 2.68E-45 |
| Pit mucous | Pit mucous | TMED10 | -0.33 | 3.07E-45 |
| Pit mucous | Pit mucous | ZG16 | 1.26 | 3.50E-45 |
| Pit mucous | Pit mucous | ATP6V1F | -0.36 | 3.73E-45 |
| Pit mucous | Pit mucous | TGIF1 | -0.36 | 5.11E-45 |
| Pit mucous | Pit mucous | LSM5 | -0.35 | 8.43E-45 |
| Pit mucous | Pit mucous | EPS8L1 | 0.26 | 8.89E-45 |
| Pit mucous | Pit mucous | BTG3 | -0.27 | 1.19E-44 |
| Pit mucous | Pit mucous | SNU13 | -0.35 | 1.28E-44 |
| Pit mucous | Pit mucous | LSM2 | -0.31 | 1.47E-44 |
| Pit mucous | Pit mucous | CXCL3 | -0.93 | 1.79E-44 |
| Pit mucous | Pit mucous | MUCL3 | -0.71 | 2.76E-44 |
| Pit mucous | Pit mucous | CDK11A | 0.27 | 3.32E-44 |
| Pit mucous | Pit mucous | ARF4 | -0.35 | 5.08E-44 |
| Pit mucous | Pit mucous | COX6B1 | 0.29 | 5.11E-44 |
| Pit mucous | Pit mucous | C12orf75 | -0.5 | 5.33E-44 |
| Pit mucous | Pit mucous | GATA6 | -0.31 | 5.57E-44 |
| Pit mucous | Pit mucous | SSR4 | -0.62 | 7.28E-44 |
| Pit mucous | Pit mucous | GABARAPL2 | -0.39 | 7.53E-44 |
| Pit mucous | Pit mucous | NUPR1 | -0.37 | 8.48E-44 |
| Pit mucous | Pit mucous | FOXQ1 | -0.56 | 1.06E-43 |
| Pit mucous | Pit mucous | RPL37A | -0.32 | 1.11E-43 |
| Pit mucous | Pit mucous | MT1G | -0.37 | 1.17E-43 |
| Pit mucous | Pit mucous | FBXW5 | 0.28 | 1.21E-43 |
| Pit mucous | Pit mucous | YBX1 | -0.36 | 1.46E-43 |
| Pit mucous | Pit mucous | CYTOR | -0.45 | 2.82E-43 |
| Pit mucous | Pit mucous | SNHG9 | -0.35 | 4.24E-43 |
| Pit mucous | Pit mucous | LAGE3 | -0.28 | 5.55E-43 |
| Pit mucous | Pit mucous | ACADVL | 0.27 | 8.33E-43 |
| Pit mucous | Pit mucous | HIGD2A | -0.32 | 1.19E-42 |
| Pit mucous | Pit mucous | MYADM | -0.36 | 2.03E-42 |
| Pit mucous | Pit mucous | SNHG7 | -0.29 | 2.04E-42 |
| Pit mucous | Pit mucous | XIST | -0.36 | 2.58E-42 |
| Pit mucous | Pit mucous | YWHAQ | -0.38 | 2.58E-42 |
| Pit mucous | Pit mucous | UBE2D3 | -0.32 | 2.89E-42 |
| Pit mucous | Pit mucous | BLOC1S1 | 0.29 | 3.12E-42 |
| Pit mucous | Pit mucous | ID3 | -0.62 | 3.45E-42 |
| Pit mucous | Pit mucous | NOP53 | -0.36 | 4.61E-42 |
| Pit mucous | Pit mucous | TSTD1 | -0.34 | 4.61E-42 |
| Pit mucous | Pit mucous | PAPSS1 | -0.31 | 5.25E-42 |
| Pit mucous | Pit mucous | CD55 | 0.27 | 7.20E-42 |
| Pit mucous | Pit mucous | CD320 | -0.68 | 7.72E-42 |
| Pit mucous | Pit mucous | MT1F | -0.41 | 8.01E-42 |
| Pit mucous | Pit mucous | SRP9 | -0.34 | 9.35E-42 |
| Pit mucous | Pit mucous | LY6E | -0.41 | 1.25E-41 |
| Pit mucous | Pit mucous | HMGB2 | -0.44 | 1.44E-41 |
| Pit mucous | Pit mucous | AKAP9 | 0.26 | 1.46E-41 |
| Pit mucous | Pit mucous | PSMB10 | 0.26 | 2.64E-41 |
| Pit mucous | Pit mucous | MUC5AC | -0.69 | 2.75E-41 |
| Pit mucous | Pit mucous | KDELR2 | -0.32 | 5.88E-41 |
| Pit mucous | Pit mucous | ANAPC16 | -0.34 | 6.32E-41 |
| Pit mucous | Pit mucous | KLF2 | -0.61 | 9.80E-41 |
| Pit mucous | Pit mucous | WDR83OS | -0.32 | 1.12E-40 |
| Pit mucous | Pit mucous | TRAPPC6A | -0.31 | 1.28E-40 |
| Pit mucous | Pit mucous | SSR3 | -0.35 | 1.31E-40 |
| Pit mucous | Pit mucous | ROMO1 | 0.26 | 2.70E-40 |
| Pit mucous | Pit mucous | NDUFAF8 | -0.3 | 3.31E-40 |
| Pit mucous | Pit mucous | ATF3 | -0.52 | 4.13E-40 |
| Pit mucous | Pit mucous | RGS1 | -0.92 | 4.44E-40 |
| Pit mucous | Pit mucous | CCNI | -0.37 | 5.84E-40 |
| Pit mucous | Pit mucous | HSP90AA1 | -0.41 | 6.44E-40 |
| Pit mucous | Pit mucous | BANF1 | -0.33 | 1.11E-39 |
| Pit mucous | Pit mucous | POLR2F | -0.32 | 1.22E-39 |
| Pit mucous | Pit mucous | PPP1R16A | 0.27 | 2.71E-39 |
| Pit mucous | Pit mucous | CCNG1 | -0.31 | 3.11E-39 |
| Pit mucous | Pit mucous | PPIB | -0.32 | 6.31E-39 |
| Pit mucous | Pit mucous | UCP2 | -0.29 | 6.39E-39 |
| Pit mucous | Pit mucous | TCEAL8 | -0.29 | 9.28E-39 |
| Pit mucous | Pit mucous | SRP14 | -0.3 | 9.41E-39 |
| Pit mucous | Pit mucous | PSME2 | 0.26 | 1.28E-38 |
| Pit mucous | Pit mucous | CDC42 | -0.35 | 1.97E-38 |
| Pit mucous | Pit mucous | LSM7 | -0.32 | 2.52E-38 |
| Pit mucous | Pit mucous | RUNX1 | -0.33 | 2.63E-38 |
| Pit mucous | Pit mucous | SMIM6 | -0.4 | 2.74E-38 |
| Pit mucous | Pit mucous | PYURF | -0.3 | 4.10E-38 |
| Pit mucous | Pit mucous | DNAJB9 | -0.45 | 4.86E-38 |
| Pit mucous | Pit mucous | DEK | -0.35 | 5.14E-38 |
| Pit mucous | Pit mucous | NDUFB3 | 0.35 | 5.48E-38 |
| Pit mucous | Pit mucous | CAMLG | -0.28 | 7.78E-38 |
| Pit mucous | Pit mucous | SERBP1 | -0.34 | 8.16E-38 |
| Pit mucous | Pit mucous | FAM107B | -0.38 | 9.81E-38 |
| Pit mucous | Pit mucous | TMEM147 | -0.32 | 1.25E-37 |
| Pit mucous | Pit mucous | SSR2 | -0.32 | 1.38E-37 |
| Pit mucous | Pit mucous | S100A4 | -0.92 | 1.59E-37 |
| Pit mucous | Pit mucous | TCEA1 | -0.32 | 1.80E-37 |
| Pit mucous | Pit mucous | IFI27 | 0.42 | 1.90E-37 |
| Pit mucous | Pit mucous | CLNS1A | -0.27 | 2.31E-37 |
| Pit mucous | Pit mucous | PPIA | -0.26 | 2.85E-37 |
| Pit mucous | Pit mucous | ANAPC11 | -0.31 | 3.04E-37 |
| Pit mucous | Pit mucous | EPB41L4A-AS1 | -0.27 | 3.16E-37 |
| Pit mucous | Pit mucous | CCDC85B | -0.34 | 3.29E-37 |
| Pit mucous | Pit mucous | PLP2 | -0.35 | 1.23E-36 |
| Pit mucous | Pit mucous | DPP7 | -0.28 | 2.29E-36 |
| Pit mucous | Pit mucous | POLR2K | -0.29 | 2.59E-36 |
| Pit mucous | Pit mucous | ATRAID | -0.32 | 2.66E-36 |
| Pit mucous | Pit mucous | NDUFV1 | 0.26 | 3.11E-36 |
| Pit mucous | Pit mucous | ANP32B | -0.33 | 3.75E-36 |
| Pit mucous | Pit mucous | RHEB | -0.31 | 8.45E-36 |
| Pit mucous | Pit mucous | ATP5PF | 0.28 | 8.52E-36 |
| Pit mucous | Pit mucous | METTL9 | -0.28 | 1.55E-35 |
| Pit mucous | Pit mucous | SMDT1 | -0.29 | 2.31E-35 |
| Pit mucous | Pit mucous | SERTAD1 | -0.37 | 5.68E-35 |
| Pit mucous | Pit mucous | DDX17 | -0.36 | 5.68E-35 |
| Pit mucous | Pit mucous | XBP1 | -0.41 | 6.00E-35 |
| Pit mucous | Pit mucous | SOCS3 | -0.64 | 6.39E-35 |
| Pit mucous | Pit mucous | NR4A1 | -0.43 | 6.90E-35 |
| Pit mucous | Pit mucous | HMGA1 | -0.38 | 8.63E-35 |
| Pit mucous | Pit mucous | FAM162A | 0.27 | 9.41E-35 |
| Pit mucous | Pit mucous | RARRES2 | -0.53 | 1.15E-34 |
| Pit mucous | Pit mucous | SNRPF | -0.33 | 1.58E-34 |
| Pit mucous | Pit mucous | CNPY2 | -0.29 | 1.98E-34 |
| Pit mucous | Pit mucous | TSPAN13 | -0.29 | 4.34E-34 |
| Pit mucous | Pit mucous | IGFBP2 | -0.34 | 4.98E-34 |
| Pit mucous | Pit mucous | CNN3 | -0.32 | 5.70E-34 |
| Pit mucous | Pit mucous | ALDOA | 0.27 | 7.38E-34 |
| Pit mucous | Pit mucous | SLC9A3R2 | -0.43 | 9.72E-34 |
| Pit mucous | Pit mucous | SFPQ | -0.34 | 1.06E-33 |
| Pit mucous | Pit mucous | SMIM30 | -0.31 | 1.63E-33 |
| Pit mucous | Pit mucous | FOXP1 | -0.34 | 2.31E-33 |
| Pit mucous | Pit mucous | PTMS | -0.33 | 2.43E-33 |
| Pit mucous | Pit mucous | CD81 | -0.27 | 4.06E-33 |
| Pit mucous | Pit mucous | TOMM20 | -0.3 | 4.64E-33 |
| Pit mucous | Pit mucous | REXO2 | -0.25 | 7.04E-33 |
| Pit mucous | Pit mucous | IRF2BP2 | -0.32 | 9.43E-33 |
| Pit mucous | Pit mucous | MYC | -0.29 | 9.82E-33 |
| Pit mucous | Pit mucous | CEBPB | -0.35 | 1.02E-32 |
| Pit mucous | Pit mucous | PCNP | -0.29 | 2.04E-32 |
| Pit mucous | Pit mucous | ERP29 | -0.3 | 2.22E-32 |
| Pit mucous | Pit mucous | FKBP11 | -0.49 | 2.33E-32 |
| Pit mucous | Pit mucous | ATP5IF1 | 0.28 | 2.97E-32 |
| Pit mucous | Pit mucous | YPEL5 | -0.51 | 3.23E-32 |
| Pit mucous | Pit mucous | TOMM5 | -0.3 | 3.85E-32 |
| Pit mucous | Pit mucous | MT-ND6 | 0.27 | 4.75E-32 |
| Pit mucous | Pit mucous | PRKAR1A | -0.29 | 5.93E-32 |
| Pit mucous | Pit mucous | EEF1G | -0.29 | 6.48E-32 |
| Pit mucous | Pit mucous | IFITM2 | -0.54 | 7.73E-32 |
| Pit mucous | Pit mucous | ARL6IP1 | -0.36 | 8.91E-32 |
| Pit mucous | Pit mucous | FAAP20 | -0.28 | 8.96E-32 |
| Pit mucous | Pit mucous | AGR3 | 0.27 | 9.07E-32 |
| Pit mucous | Pit mucous | FAR1 | -0.29 | 1.31E-31 |
| Pit mucous | Pit mucous | MT2A | -0.26 | 1.39E-31 |
| Pit mucous | Pit mucous | TMEM14B | -0.28 | 1.91E-31 |
| Pit mucous | Pit mucous | MTHFD2 | -0.31 | 2.17E-31 |
| Pit mucous | Pit mucous | HNRNPH1 | -0.36 | 2.60E-31 |
| Pit mucous | Pit mucous | TMEM98 | -0.29 | 2.95E-31 |
| Pit mucous | Pit mucous | CCT4 | -0.26 | 5.65E-31 |
| Pit mucous | Pit mucous | ESD | -0.28 | 6.29E-31 |
| Pit mucous | Pit mucous | JUN | -0.72 | 9.45E-31 |
| Pit mucous | Pit mucous | MRPL33 | -0.32 | 9.89E-31 |
| Pit mucous | Pit mucous | PAIP2 | -0.29 | 1.21E-30 |
| Pit mucous | Pit mucous | PSAPL1 | -0.43 | 1.31E-30 |
| Pit mucous | Pit mucous | SNRPD1 | -0.3 | 1.38E-30 |
| Pit mucous | Pit mucous | MZT2A | -0.31 | 1.55E-30 |
| Pit mucous | Pit mucous | PDIA6 | -0.29 | 1.73E-30 |
| Pit mucous | Pit mucous | RBX1 | -0.29 | 2.06E-30 |
| Pit mucous | Pit mucous | DDOST | -0.28 | 2.44E-30 |
| Pit mucous | Pit mucous | UBE2B | -0.28 | 2.47E-30 |
| Pit mucous | Pit mucous | APOL1 | 0.26 | 2.59E-30 |
| Pit mucous | Pit mucous | ERO1B | -0.37 | 3.47E-30 |
| Pit mucous | Pit mucous | EIF3L | -0.31 | 4.33E-30 |
| Pit mucous | Pit mucous | JUND | -0.34 | 4.89E-30 |
| Pit mucous | Pit mucous | RAD23A | -0.28 | 7.55E-30 |
| Pit mucous | Pit mucous | HIPK2 | -0.26 | 8.47E-30 |
| Pit mucous | Pit mucous | MRPL51 | -0.27 | 1.01E-29 |
| Pit mucous | Pit mucous | CREM | -0.54 | 1.69E-29 |
| Pit mucous | Pit mucous | PRMT1 | -0.28 | 3.77E-29 |
| Pit mucous | Pit mucous | RNASE4 | -0.27 | 3.87E-29 |
| Pit mucous | Pit mucous | C12orf57 | -0.27 | 4.41E-29 |
| Pit mucous | Pit mucous | EIF3F | -0.28 | 5.61E-29 |
| Pit mucous | Pit mucous | EIF3D | -0.29 | 1.71E-28 |
| Pit mucous | Pit mucous | FBL | -0.3 | 2.17E-28 |
| Pit mucous | Pit mucous | COX16 | -0.26 | 2.30E-28 |
| Pit mucous | Pit mucous | POLR2I | -0.27 | 2.30E-28 |
| Pit mucous | Pit mucous | PRR4 | -0.54 | 3.01E-28 |
| Pit mucous | Pit mucous | SNRPB | -0.3 | 3.22E-28 |
| Pit mucous | Pit mucous | EIF1B | -0.28 | 3.39E-28 |
| Pit mucous | Pit mucous | NEDD8 | -0.26 | 1.15E-27 |
| Pit mucous | Pit mucous | COPS9 | -0.27 | 1.37E-27 |
| Pit mucous | Pit mucous | ATP5MD | 0.33 | 1.39E-27 |
| Pit mucous | Pit mucous | DEPP1 | -0.38 | 1.51E-27 |
| Pit mucous | Pit mucous | AAMDC | -0.33 | 1.51E-27 |
| Pit mucous | Pit mucous | HNRNPK | -0.27 | 1.85E-27 |
| Pit mucous | Pit mucous | RPS4Y1 | -0.46 | 2.01E-27 |
| Pit mucous | Pit mucous | KDM6B | -0.31 | 2.23E-27 |
| Pit mucous | Pit mucous | ST13 | -0.28 | 4.06E-27 |
| Pit mucous | Pit mucous | RBBP4 | -0.25 | 4.19E-27 |
| Pit mucous | Pit mucous | QDPR | -0.27 | 4.39E-27 |
| Pit mucous | Pit mucous | HDAC2 | -0.25 | 4.80E-27 |
| Pit mucous | Pit mucous | UBE2D2 | -0.27 | 5.34E-27 |
| Pit mucous | Pit mucous | CYB5A | -0.26 | 5.34E-27 |
| Pit mucous | Pit mucous | SYPL1 | -0.29 | 6.07E-27 |
| Pit mucous | Pit mucous | YBX3 | -0.29 | 7.64E-27 |
| Pit mucous | Pit mucous | ILF2 | -0.26 | 9.93E-27 |
| Pit mucous | Pit mucous | MCL1 | -0.32 | 1.13E-26 |
| Pit mucous | Pit mucous | HNRNPA3 | -0.27 | 1.22E-26 |
| Pit mucous | Pit mucous | NUDC | -0.27 | 1.36E-26 |
| Pit mucous | Pit mucous | KLF6 | -0.32 | 1.41E-26 |
| Pit mucous | Pit mucous | SNRPG | -0.27 | 1.58E-26 |
| Pit mucous | Pit mucous | UQCC2 | -0.28 | 1.72E-26 |
| Pit mucous | Pit mucous | NQO1 | -0.36 | 2.03E-26 |
| Pit mucous | Pit mucous | SQSTM1 | -0.36 | 2.04E-26 |
| Pit mucous | Pit mucous | SELENOK | -0.3 | 2.41E-26 |
| Pit mucous | Pit mucous | CMTM6 | -0.27 | 2.47E-26 |
| Pit mucous | Pit mucous | TNFAIP3 | -0.42 | 2.50E-26 |
| Pit mucous | Pit mucous | UXT | -0.26 | 3.10E-26 |
| Pit mucous | Pit mucous | PPP2CA | -0.27 | 3.11E-26 |
| Pit mucous | Pit mucous | ANKRD28 | -0.45 | 3.39E-26 |
| Pit mucous | Pit mucous | SIK1 | -0.38 | 3.43E-26 |
| Pit mucous | Pit mucous | NUCB2 | -0.26 | 5.23E-26 |
| Pit mucous | Pit mucous | CARHSP1 | -0.25 | 8.25E-26 |
| Pit mucous | Pit mucous | TRA2B | -0.31 | 8.51E-26 |
| Pit mucous | Pit mucous | NAA38 | -0.27 | 8.98E-26 |
| Pit mucous | Pit mucous | UBL3 | -0.32 | 1.06E-25 |
| Pit mucous | Pit mucous | U2AF1 | -0.28 | 1.52E-25 |
| Pit mucous | Pit mucous | RNPS1 | -0.28 | 2.39E-25 |
| Pit mucous | Pit mucous | RWDD1 | -0.28 | 3.36E-25 |
| Pit mucous | Pit mucous | UBB | -0.25 | 3.42E-25 |
| Pit mucous | Pit mucous | PARK7 | -0.27 | 3.67E-25 |
| Pit mucous | Pit mucous | PSMB1 | -0.26 | 4.45E-25 |
| Pit mucous | Pit mucous | RBM8A | -0.29 | 4.91E-25 |
| Pit mucous | Pit mucous | MIR4435-2HG | -0.26 | 7.02E-25 |
| Pit mucous | Pit mucous | CYR61 | -0.31 | 8.33E-25 |
| Pit mucous | Pit mucous | LDHB | -0.34 | 1.17E-24 |
| Pit mucous | Pit mucous | GLO1 | -0.25 | 1.34E-24 |
| Pit mucous | Pit mucous | TSPYL1 | -0.26 | 1.64E-24 |
| Pit mucous | Pit mucous | TRAM1 | -0.27 | 1.77E-24 |
| Pit mucous | Pit mucous | TMEM219 | -0.26 | 1.83E-24 |
| Pit mucous | Pit mucous | ITM2C | -0.4 | 2.04E-24 |
| Pit mucous | Pit mucous | TM4SF1 | -0.39 | 2.71E-24 |
| Pit mucous | Pit mucous | RND1 | -0.29 | 3.07E-24 |
| Pit mucous | Pit mucous | TPPP3 | -0.33 | 3.27E-24 |
| Pit mucous | Pit mucous | GTF2I | -0.27 | 6.22E-24 |
| Pit mucous | Pit mucous | PFDN2 | -0.25 | 7.61E-24 |
| Pit mucous | Pit mucous | TSC22D1 | -0.36 | 9.48E-24 |
| Pit mucous | Pit mucous | RSL1D1 | -0.27 | 1.04E-23 |
| Pit mucous | Pit mucous | SDC4 | -0.33 | 1.11E-23 |
| Pit mucous | Pit mucous | SPCS1 | -0.27 | 1.60E-23 |
| Pit mucous | Pit mucous | SSBP3 | -0.26 | 2.00E-23 |
| Pit mucous | Pit mucous | WEE1 | -0.28 | 2.54E-23 |
| Pit mucous | Pit mucous | MIA | -0.45 | 2.96E-23 |
| Pit mucous | Pit mucous | SMIM14 | -0.32 | 3.42E-23 |
| Pit mucous | Pit mucous | CD74 | -0.75 | 6.51E-23 |
| Pit mucous | Pit mucous | ARID5B | -0.29 | 8.08E-23 |
| Pit mucous | Pit mucous | BIK | -0.28 | 9.49E-23 |
| Pit mucous | Pit mucous | SDCBP | -0.32 | 1.27E-22 |
| Pit mucous | Pit mucous | ELOB | 0.26 | 1.94E-22 |
| Pit mucous | Pit mucous | GNAQ | -0.25 | 2.03E-22 |
| Pit mucous | Pit mucous | TBCB | -0.26 | 2.23E-22 |
| Pit mucous | Pit mucous | IDS | -0.29 | 3.81E-22 |
| Pit mucous | Pit mucous | UQCRB | 0.25 | 4.01E-22 |
| Pit mucous | Pit mucous | TUBA1B | -0.43 | 4.73E-22 |
| Pit mucous | Pit mucous | PRDX4 | -0.32 | 5.00E-22 |
| Pit mucous | Pit mucous | MMP1 | -0.56 | 5.04E-22 |
| Pit mucous | Pit mucous | ID2 | -0.48 | 5.10E-22 |
| Pit mucous | Pit mucous | SGK1 | -0.3 | 5.75E-22 |
| Pit mucous | Pit mucous | TFF1 | -0.81 | 8.33E-22 |
| Pit mucous | Pit mucous | ERLEC1 | -0.25 | 1.24E-21 |
| Pit mucous | Pit mucous | MANF | -0.31 | 1.24E-21 |
| Pit mucous | Pit mucous | IGLL5 | -2.34 | 1.34E-21 |
| Pit mucous | Pit mucous | GPX1 | -0.26 | 3.04E-21 |
| Pit mucous | Pit mucous | SPCS3 | -0.27 | 1.09E-20 |
| Pit mucous | Pit mucous | SLC3A2 | -0.26 | 1.45E-20 |
| Pit mucous | Pit mucous | YWHAH | -0.31 | 4.13E-20 |
| Pit mucous | Pit mucous | COX6A1 | 0.28 | 4.95E-20 |
| Pit mucous | Pit mucous | BRI3 | -0.32 | 8.39E-20 |
| Pit mucous | Pit mucous | PCLAF | -0.29 | 1.24E-19 |
| Pit mucous | Pit mucous | 7-Sep | -0.29 | 1.76E-19 |
| Pit mucous | Pit mucous | ISCU | -0.26 | 4.33E-19 |
| Pit mucous | Pit mucous | ALDH1A1 | -0.3 | 6.24E-19 |
| Pit mucous | Pit mucous | SH3BGRL | -0.25 | 1.02E-18 |
| Pit mucous | Pit mucous | SEC11C | -0.39 | 1.37E-18 |
| Pit mucous | Pit mucous | PAM | -0.26 | 1.39E-18 |
| Pit mucous | Pit mucous | CLK1 | -0.26 | 2.64E-18 |
| Pit mucous | Pit mucous | SOX4 | -0.36 | 3.40E-18 |
| Pit mucous | Pit mucous | GCLC | -0.26 | 5.02E-18 |
| Pit mucous | Pit mucous | ARHGDIB | -0.46 | 5.05E-18 |
| Pit mucous | Pit mucous | HLA-DPB1 | -0.6 | 1.22E-17 |
| Pit mucous | Pit mucous | RAB11FIP1 | -0.32 | 1.49E-17 |
| Pit mucous | Pit mucous | BIRC3 | -0.3 | 3.22E-17 |
| Pit mucous | Pit mucous | CTSE | -0.32 | 3.25E-17 |
| Pit mucous | Pit mucous | KLK1 | 0.27 | 3.52E-17 |
| Pit mucous | Pit mucous | NAMPT | -0.26 | 7.48E-17 |
| Pit mucous | Pit mucous | BRD2 | -0.26 | 8.05E-17 |
| Pit mucous | Pit mucous | SLC38A2 | -0.26 | 9.39E-17 |
| Pit mucous | Pit mucous | SH3BGRL2 | -0.27 | 1.71E-16 |
| Pit mucous | Pit mucous | ARF6 | -0.3 | 2.25E-16 |
| Pit mucous | Pit mucous | TIMP1 | -0.43 | 3.19E-16 |
| Pit mucous | Pit mucous | CKLF | -0.26 | 3.78E-16 |
| Pit mucous | Pit mucous | HSPA1A | -0.62 | 5.80E-16 |
| Pit mucous | Pit mucous | IFNGR2 | -0.26 | 8.35E-16 |
| Pit mucous | Pit mucous | SERPINA1 | -0.26 | 1.44E-15 |
| Pit mucous | Pit mucous | TGFBR2 | -0.25 | 2.80E-15 |
| Pit mucous | Pit mucous | HSPB1 | -0.37 | 3.08E-15 |
| Pit mucous | Pit mucous | DDIT4 | -0.42 | 3.39E-15 |
| Pit mucous | Pit mucous | OLFM4 | -0.52 | 3.40E-15 |
| Pit mucous | Pit mucous | VSIG2 | -0.3 | 3.94E-15 |
| Pit mucous | Pit mucous | NEDD9 | -0.27 | 5.63E-15 |
| Pit mucous | Pit mucous | TOB1 | -0.25 | 8.80E-15 |
| Pit mucous | Pit mucous | IRF1 | -0.34 | 1.27E-14 |
| Pit mucous | Pit mucous | SLC7A8 | -0.29 | 1.30E-14 |
| Pit mucous | Pit mucous | BPIFB1 | -0.73 | 1.31E-14 |
| Pit mucous | Pit mucous | MAFF | -0.28 | 2.30E-14 |
| Pit mucous | Pit mucous | RFLNA | -0.28 | 3.05E-14 |
| Pit mucous | Pit mucous | CXCL1 | -0.39 | 5.81E-14 |
| Pit mucous | Pit mucous | CHP1 | -0.25 | 6.53E-14 |
| Pit mucous | Pit mucous | TXNIP | -0.34 | 7.06E-14 |
| Pit mucous | Pit mucous | SOD2 | -0.38 | 1.48E-13 |
| Pit mucous | Pit mucous | CKS2 | -0.25 | 4.72E-13 |
| Pit mucous | Pit mucous | GDF15 | -0.3 | 5.87E-13 |
| Pit mucous | Pit mucous | DNAJA1 | -0.26 | 1.11E-12 |
| Pit mucous | Pit mucous | AC023090.1 | -0.26 | 1.52E-12 |
| Pit mucous | Pit mucous | HLA-DPA1 | -0.47 | 1.68E-12 |
| Pit mucous | Pit mucous | UGCG | -0.28 | 3.56E-12 |
| Pit mucous | Pit mucous | LASP1 | -0.26 | 6.08E-12 |
| Pit mucous | Pit mucous | COTL1 | -0.25 | 6.22E-11 |
| Pit mucous | Pit mucous | F2RL1 | -0.25 | 2.35E-08 |
| Pit mucous | Pit mucous | PHLDA1 | -0.29 | 4.40E-08 |
| Pit mucous | Pit mucous | HIST1H4C | -0.41 | 5.44E-08 |
| Pit mucous | Pit mucous | ZFP36L2 | -0.25 | 4.77E-07 |
| Pit mucous | Pit mucous | IGFBP4 | -0.29 | 1.65E-06 |
| Pit mucous | Pit mucous | IFITM1 | -0.45 | 3.08E-06 |
| Pit mucous | Pit mucous | TFF3 | 2.37 | 0 |
| Pit mucous | Pit mucous | MT-ND5 | 1.05 | 0 |
| Pit mucous | Pit mucous | MT-CYB | 1.02 | 0 |
| Pit mucous | Pit mucous | MT-ATP6 | 1 | 0 |
| Pit mucous | Pit mucous | MT-CO1 | 0.99 | 0 |
| Pit mucous | Pit mucous | MT-CO3 | 0.98 | 0 |
| Pit mucous | Pit mucous | MT-ND1 | 0.97 | 0 |
| Pit mucous | Pit mucous | REG4 | 0.96 | 0 |
| Pit mucous | Pit mucous | MT-ND4 | 0.93 | 0 |
| Pit mucous | Pit mucous | MT-ND2 | 0.93 | 0 |
| Pit mucous | Pit mucous | MT-CO2 | 0.92 | 0 |
| Pit mucous | Pit mucous | CLDN4 | 0.74 | 0 |
| Pit mucous | Pit mucous | TMSB4X | -1.04 | 0 |
| Pit mucous | Pit mucous | CYSTM1 | -1.05 | 0 |
| Pit mucous | Pit mucous | GAST | -4.16 | 0 |
| Pit mucous | Pit mucous | ACTB | -0.82 | 2.53E-304 |
| Pit mucous | Pit mucous | PGC | -3.03 | 1.06E-300 |
| Pit mucous | Pit mucous | B2M | -0.87 | 2.27E-284 |
| Pit mucous | Pit mucous | GKN1 | -3.54 | 4.80E-263 |
| Pit mucous | Pit mucous | OLFM4 | 0.85 | 3.53E-246 |
| Pit mucous | Pit mucous | GKN2 | -2.66 | 8.35E-236 |
| Pit mucous | Pit mucous | FTH1 | -0.58 | 1.55E-226 |
| Pit mucous | Pit mucous | TFF2 | -1.55 | 1.44E-220 |
| Pit mucous | Pit mucous | ITLN1 | 2.09 | 1.53E-220 |
| Pit mucous | Pit mucous | CTSE | -0.95 | 9.97E-215 |
| Pit mucous | Pit mucous | FTL | -0.68 | 1.88E-213 |
| Pit mucous | Pit mucous | MSMB | -1.56 | 1.06E-212 |
| Pit mucous | Pit mucous | MT-ND3 | 0.62 | 2.54E-211 |
| Pit mucous | Pit mucous | TAGLN2 | -0.87 | 3.29E-209 |
| Pit mucous | Pit mucous | KRT18 | 0.54 | 3.08E-208 |
| Pit mucous | Pit mucous | TFF1 | -1.83 | 1.30E-201 |
| Pit mucous | Pit mucous | CA2 | -1.07 | 5.91E-191 |
| Pit mucous | Pit mucous | SPINK4 | 2.77 | 2.47E-189 |
| Pit mucous | Pit mucous | HLA-B | -0.69 | 2.23E-186 |
| Pit mucous | Pit mucous | MALAT1 | 0.53 | 1.31E-185 |
| Pit mucous | Pit mucous | CLDN18 | -1 | 4.94E-171 |
| Pit mucous | Pit mucous | SUMO2 | -0.62 | 1.83E-163 |
| Pit mucous | Pit mucous | MUC5AC | -1.32 | 7.32E-153 |
| Pit mucous | Pit mucous | RNASE1 | -0.87 | 4.38E-151 |
| Pit mucous | Pit mucous | H3F3A | -0.46 | 7.70E-151 |
| Pit mucous | Pit mucous | MUC2 | 1.5 | 2.67E-150 |
| Pit mucous | Pit mucous | CD59 | -0.68 | 4.97E-150 |
| Pit mucous | Pit mucous | DUSP1 | -0.91 | 2.99E-148 |
| Pit mucous | Pit mucous | PSCA | -1.72 | 1.22E-147 |
| Pit mucous | Pit mucous | RPS19 | 0.49 | 2.80E-146 |
| Pit mucous | Pit mucous | SERF2 | -0.42 | 6.10E-144 |
| Pit mucous | Pit mucous | ITM2B | -0.67 | 1.02E-142 |
| Pit mucous | Pit mucous | EIF1 | -0.43 | 4.07E-142 |
| Pit mucous | Pit mucous | TPT1 | -0.47 | 7.08E-142 |
| Pit mucous | Pit mucous | FXYD3 | -0.64 | 2.41E-141 |
| Pit mucous | Pit mucous | ACTG1 | -0.5 | 3.53E-140 |
| Pit mucous | Pit mucous | RPS18 | 0.43 | 1.02E-139 |
| Pit mucous | Pit mucous | CALM2 | -0.5 | 1.11E-139 |
| Pit mucous | Pit mucous | CLDN3 | 0.64 | 1.44E-136 |
| Pit mucous | Pit mucous | CXCL17 | -0.9 | 1.82E-136 |
| Pit mucous | Pit mucous | MT-ND4L | 0.63 | 1.11E-132 |
| Pit mucous | Pit mucous | CRIP1 | -0.78 | 1.22E-132 |
| Pit mucous | Pit mucous | PPDPF | -0.6 | 8.55E-132 |
| Pit mucous | Pit mucous | VSIG2 | -0.75 | 1.40E-131 |
| Pit mucous | Pit mucous | CST3 | -0.59 | 1.63E-130 |
| Pit mucous | Pit mucous | ZFP36 | -1.05 | 3.68E-127 |
| Pit mucous | Pit mucous | TESC | -0.76 | 8.38E-127 |
| Pit mucous | Pit mucous | EEF1A1 | -0.39 | 6.83E-125 |
| Pit mucous | Pit mucous | TMEM59 | -0.57 | 3.03E-124 |
| Pit mucous | Pit mucous | RPS2 | 0.44 | 2.07E-123 |
| Pit mucous | Pit mucous | FOSB | -0.84 | 1.86E-120 |
| Pit mucous | Pit mucous | KLF6 | -0.57 | 2.18E-120 |
| Pit mucous | Pit mucous | CD63 | -0.44 | 1.08E-118 |
| Pit mucous | Pit mucous | FKBP1A | -0.57 | 1.78E-117 |
| Pit mucous | Pit mucous | ALDH3A1 | -0.79 | 2.71E-117 |
| Pit mucous | Pit mucous | SST | -2.74 | 5.95E-116 |
| Pit mucous | Pit mucous | HES1 | 0.66 | 6.85E-115 |
| Pit mucous | Pit mucous | MUCL3 | -1.17 | 6.08E-113 |
| Pit mucous | Pit mucous | DMBT1 | 0.56 | 1.03E-112 |
| Pit mucous | Pit mucous | RHOA | -0.53 | 6.37E-111 |
| Pit mucous | Pit mucous | PTMA | -0.42 | 5.36E-109 |
| Pit mucous | Pit mucous | UBE2D3 | -0.5 | 2.09E-108 |
| Pit mucous | Pit mucous | CFL1 | -0.45 | 9.30E-108 |
| Pit mucous | Pit mucous | RPL10 | -0.35 | 8.54E-107 |
| Pit mucous | Pit mucous | CDC42 | -0.53 | 1.74E-106 |
| Pit mucous | Pit mucous | KLF2 | -0.78 | 1.19E-104 |
| Pit mucous | Pit mucous | NQO1 | -0.59 | 1.72E-104 |
| Pit mucous | Pit mucous | PIGR | 0.44 | 3.75E-103 |
| Pit mucous | Pit mucous | HSPB1 | -0.64 | 5.90E-103 |
| Pit mucous | Pit mucous | VAPA | -0.49 | 6.41E-103 |
| Pit mucous | Pit mucous | DDX5 | -0.47 | 8.72E-102 |
| Pit mucous | Pit mucous | TMBIM6 | -0.43 | 8.72E-102 |
| Pit mucous | Pit mucous | PRDX1 | -0.5 | 1.49E-101 |
| Pit mucous | Pit mucous | SEC61G | -0.45 | 2.88E-101 |
| Pit mucous | Pit mucous | GABARAP | -0.49 | 7.67E-101 |
| Pit mucous | Pit mucous | KRT7 | 0.61 | 1.80E-99 |
| Pit mucous | Pit mucous | PRDX5 | -0.47 | 2.45E-99 |
| Pit mucous | Pit mucous | ATP6V1G1 | -0.44 | 4.19E-99 |
| Pit mucous | Pit mucous | RPL28 | 0.38 | 3.66E-98 |
| Pit mucous | Pit mucous | TM4SF1 | -0.66 | 1.57E-97 |
| Pit mucous | Pit mucous | HPGD | -0.75 | 1.73E-97 |
| Pit mucous | Pit mucous | TRIM54 | 0.49 | 3.72E-97 |
| Pit mucous | Pit mucous | OAZ1 | -0.4 | 7.17E-97 |
| Pit mucous | Pit mucous | LAPTM4A | -0.5 | 1.10E-95 |
| Pit mucous | Pit mucous | C12orf75 | -0.61 | 4.42E-93 |
| Pit mucous | Pit mucous | MORF4L1 | -0.46 | 5.15E-93 |
| Pit mucous | Pit mucous | RPS15 | 0.37 | 7.74E-93 |
| Pit mucous | Pit mucous | GLUL | -0.61 | 2.64E-92 |
| Pit mucous | Pit mucous | BTG1 | -0.67 | 1.28E-91 |
| Pit mucous | Pit mucous | TMSB10 | -0.37 | 6.76E-91 |
| Pit mucous | Pit mucous | VAMP8 | -0.53 | 2.51E-90 |
| Pit mucous | Pit mucous | SDCBP | -0.52 | 1.03E-89 |
| Pit mucous | Pit mucous | ALDH1A1 | -0.51 | 1.74E-89 |
| Pit mucous | Pit mucous | BRK1 | -0.43 | 2.39E-89 |
| Pit mucous | Pit mucous | JUND | -0.54 | 7.60E-89 |
| Pit mucous | Pit mucous | RPL36AL | -0.39 | 3.74E-88 |
| Pit mucous | Pit mucous | ARPC2 | -0.46 | 5.50E-88 |
| Pit mucous | Pit mucous | SERP1 | -0.41 | 6.38E-88 |
| Pit mucous | Pit mucous | H3F3B | -0.35 | 7.25E-88 |
| Pit mucous | Pit mucous | TMED10 | -0.41 | 5.36E-87 |
| Pit mucous | Pit mucous | PPP1CB | -0.47 | 2.08E-86 |
| Pit mucous | Pit mucous | AC020656.1 | -1.33 | 7.11E-86 |
| Pit mucous | Pit mucous | CIRBP | -0.53 | 7.33E-86 |
| Pit mucous | Pit mucous | EIF4A2 | -0.44 | 1.55E-85 |
| Pit mucous | Pit mucous | CD99 | -0.47 | 2.98E-85 |
| Pit mucous | Pit mucous | VIM | -1.31 | 2.75E-84 |
| Pit mucous | Pit mucous | ATP6V0E1 | -0.41 | 2.73E-83 |
| Pit mucous | Pit mucous | TSC22D3 | -1.05 | 6.26E-83 |
| Pit mucous | Pit mucous | CLDN7 | 0.27 | 1.07E-82 |
| Pit mucous | Pit mucous | FAU | -0.3 | 1.80E-81 |
| Pit mucous | Pit mucous | PFN1 | -0.38 | 1.78E-80 |
| Pit mucous | Pit mucous | PSAP | -0.47 | 3.01E-80 |
| Pit mucous | Pit mucous | MUC1 | -0.73 | 6.16E-80 |
| Pit mucous | Pit mucous | SERTAD1 | -0.5 | 7.69E-80 |
| Pit mucous | Pit mucous | MCL1 | -0.44 | 8.78E-80 |
| Pit mucous | Pit mucous | RPS10 | -0.42 | 8.78E-80 |
| Pit mucous | Pit mucous | SLPI | -0.59 | 1.08E-79 |
| Pit mucous | Pit mucous | SARAF | -0.46 | 1.34E-79 |
| Pit mucous | Pit mucous | MGST3 | -0.57 | 1.06E-78 |
| Pit mucous | Pit mucous | SNX3 | -0.4 | 2.16E-78 |
| Pit mucous | Pit mucous | ATF3 | -0.57 | 4.42E-78 |
| Pit mucous | Pit mucous | NAP1L1 | -0.46 | 2.70E-77 |
| Pit mucous | Pit mucous | CLIC1 | -0.38 | 2.74E-77 |
| Pit mucous | Pit mucous | TAX1BP3 | -0.47 | 3.23E-77 |
| Pit mucous | Pit mucous | ATP5F1E | -0.35 | 8.30E-77 |
| Pit mucous | Pit mucous | MYL12B | -0.42 | 3.65E-76 |
| Pit mucous | Pit mucous | MMP1 | -0.74 | 6.21E-76 |
| Pit mucous | Pit mucous | DAD1 | -0.39 | 6.19E-75 |
| Pit mucous | Pit mucous | HLA-C | -0.55 | 2.59E-74 |
| Pit mucous | Pit mucous | RHOB | -0.53 | 3.10E-74 |
| Pit mucous | Pit mucous | VSIG1 | -0.57 | 2.93E-73 |
| Pit mucous | Pit mucous | MYL6 | -0.33 | 7.98E-73 |
| Pit mucous | Pit mucous | SMIM6 | -0.49 | 1.26E-72 |
| Pit mucous | Pit mucous | YPEL5 | -0.55 | 1.27E-72 |
| Pit mucous | Pit mucous | CHP1 | -0.44 | 1.46E-72 |
| Pit mucous | Pit mucous | GABARAPL2 | -0.43 | 1.47E-72 |
| Pit mucous | Pit mucous | FOS | -0.96 | 7.60E-72 |
| Pit mucous | Pit mucous | EGR1 | -0.74 | 8.00E-72 |
| Pit mucous | Pit mucous | SAT1 | -0.44 | 2.51E-71 |
| Pit mucous | Pit mucous | MT-ATP8 | 0.48 | 2.58E-71 |
| Pit mucous | Pit mucous | SCP2 | -0.41 | 2.66E-71 |
| Pit mucous | Pit mucous | NDFIP1 | -0.35 | 2.75E-71 |
| Pit mucous | Pit mucous | SUB1 | -0.4 | 4.38E-71 |
| Pit mucous | Pit mucous | NFE2L2 | -0.41 | 9.40E-71 |
| Pit mucous | Pit mucous | RHEB | -0.37 | 1.76E-70 |
| Pit mucous | Pit mucous | ARL6IP1 | -0.42 | 3.90E-70 |
| Pit mucous | Pit mucous | SPCS2 | -0.45 | 4.65E-70 |
| Pit mucous | Pit mucous | AKR1B10 | -0.69 | 1.43E-69 |
| Pit mucous | Pit mucous | BRI3 | -0.45 | 2.06E-69 |
| Pit mucous | Pit mucous | SMDT1 | -0.38 | 3.82E-69 |
| Pit mucous | Pit mucous | H2AFZ | -0.53 | 4.70E-69 |
| Pit mucous | Pit mucous | SELENOT | -0.37 | 5.12E-69 |
| Pit mucous | Pit mucous | LGALS1 | -1.31 | 6.30E-69 |
| Pit mucous | Pit mucous | OST4 | -0.32 | 4.04E-68 |
| Pit mucous | Pit mucous | SQSTM1 | -0.46 | 6.93E-68 |
| Pit mucous | Pit mucous | JPT1 | -0.51 | 8.28E-68 |
| Pit mucous | Pit mucous | PRDX6 | -0.39 | 1.61E-67 |
| Pit mucous | Pit mucous | HNRNPF | -0.38 | 2.90E-67 |
| Pit mucous | Pit mucous | CAPZA2 | -0.38 | 9.12E-67 |
| Pit mucous | Pit mucous | YWHAE | -0.37 | 1.56E-66 |
| Pit mucous | Pit mucous | S100P | -0.8 | 1.29E-65 |
| Pit mucous | Pit mucous | RPS28 | -0.32 | 2.05E-65 |
| Pit mucous | Pit mucous | CCNI | -0.38 | 7.07E-65 |
| Pit mucous | Pit mucous | CLU | -0.71 | 8.01E-65 |
| Pit mucous | Pit mucous | NPC2 | -0.41 | 1.27E-64 |
| Pit mucous | Pit mucous | MRPL12 | 0.46 | 1.36E-64 |
| Pit mucous | Pit mucous | VAMP2 | -0.45 | 1.84E-64 |
| Pit mucous | Pit mucous | EMP1 | -0.52 | 2.47E-64 |
| Pit mucous | Pit mucous | SEC61B | -0.32 | 9.09E-64 |
| Pit mucous | Pit mucous | HMGN2 | -0.48 | 2.00E-63 |
| Pit mucous | Pit mucous | ARF6 | -0.42 | 4.19E-63 |
| Pit mucous | Pit mucous | MYL12A | -0.4 | 4.29E-63 |
| Pit mucous | Pit mucous | RAB7A | -0.36 | 1.53E-62 |
| Pit mucous | Pit mucous | REG1A | 0.26 | 1.60E-62 |
| Pit mucous | Pit mucous | YWHAH | -0.43 | 1.69E-62 |
| Pit mucous | Pit mucous | DYNLL1 | -0.36 | 1.69E-62 |
| Pit mucous | Pit mucous | ANAPC16 | -0.35 | 2.03E-62 |
| Pit mucous | Pit mucous | PNRC1 | -0.44 | 3.44E-62 |
| Pit mucous | Pit mucous | TMEM258 | -0.33 | 4.02E-62 |
| Pit mucous | Pit mucous | SDC4 | -0.46 | 7.12E-62 |
| Pit mucous | Pit mucous | PTP4A1 | -0.37 | 1.58E-61 |
| Pit mucous | Pit mucous | EID1 | -0.43 | 2.15E-61 |
| Pit mucous | Pit mucous | SRGN | -1.07 | 1.12E-60 |
| Pit mucous | Pit mucous | BEX3 | -0.45 | 1.71E-60 |
| Pit mucous | Pit mucous | NEDD8 | -0.34 | 1.82E-60 |
| Pit mucous | Pit mucous | AHNAK | -0.41 | 2.26E-60 |
| Pit mucous | Pit mucous | COX8A | -0.35 | 3.40E-60 |
| Pit mucous | Pit mucous | DAZAP2 | -0.37 | 3.83E-60 |
| Pit mucous | Pit mucous | EIF1AX | -0.34 | 5.97E-60 |
| Pit mucous | Pit mucous | ARF4 | -0.34 | 7.25E-60 |
| Pit mucous | Pit mucous | SPINK1 | -0.73 | 2.21E-59 |
| Pit mucous | Pit mucous | CD164 | -0.36 | 2.29E-59 |
| Pit mucous | Pit mucous | FAM3B | -0.39 | 1.91E-58 |
| Pit mucous | Pit mucous | TMEM14A | -0.31 | 1.96E-58 |
| Pit mucous | Pit mucous | RAB5C | -0.36 | 2.64E-58 |
| Pit mucous | Pit mucous | DDX17 | -0.38 | 3.33E-58 |
| Pit mucous | Pit mucous | SOCS3 | -0.68 | 4.51E-58 |
| Pit mucous | Pit mucous | SRSF3 | -0.36 | 1.09E-57 |
| Pit mucous | Pit mucous | S100A4 | -0.95 | 2.20E-57 |
| Pit mucous | Pit mucous | UGCG | -0.44 | 3.53E-57 |
| Pit mucous | Pit mucous | ISG20 | -0.42 | 9.41E-57 |
| Pit mucous | Pit mucous | PPP2CA | -0.32 | 1.91E-56 |
| Pit mucous | Pit mucous | SKP1 | -0.26 | 2.69E-56 |
| Pit mucous | Pit mucous | TMED2 | -0.27 | 3.65E-56 |
| Pit mucous | Pit mucous | LGMN | -0.34 | 4.01E-56 |
| Pit mucous | Pit mucous | AADAC | -0.43 | 4.35E-56 |
| Pit mucous | Pit mucous | SRSF5 | -0.36 | 4.81E-56 |
| Pit mucous | Pit mucous | ANXA10 | -0.47 | 8.38E-56 |
| Pit mucous | Pit mucous | LINC01133 | -0.58 | 8.54E-56 |
| Pit mucous | Pit mucous | CBR1 | -0.6 | 1.34E-55 |
| Pit mucous | Pit mucous | HERPUD1 | -0.6 | 3.25E-55 |
| Pit mucous | Pit mucous | UBE2B | -0.32 | 4.02E-55 |
| Pit mucous | Pit mucous | ARPC1A | -0.36 | 4.32E-55 |
| Pit mucous | Pit mucous | LGALS4 | 0.37 | 4.65E-55 |
| Pit mucous | Pit mucous | TSPO | -0.35 | 5.55E-55 |
| Pit mucous | Pit mucous | TMEM219 | -0.32 | 6.36E-55 |
| Pit mucous | Pit mucous | RPL13 | 0.31 | 7.97E-55 |
| Pit mucous | Pit mucous | KDELR2 | -0.31 | 3.14E-54 |
| Pit mucous | Pit mucous | RAB11A | -0.37 | 3.21E-54 |
| Pit mucous | Pit mucous | S100A11 | -0.38 | 3.96E-54 |
| Pit mucous | Pit mucous | UBL3 | -0.39 | 4.03E-54 |
| Pit mucous | Pit mucous | YWHAZ | -0.34 | 5.52E-54 |
| Pit mucous | Pit mucous | SRP9 | -0.27 | 1.04E-53 |
| Pit mucous | Pit mucous | NAMPT | -0.35 | 1.33E-53 |
| Pit mucous | Pit mucous | PTGES3 | -0.32 | 1.59E-53 |
| Pit mucous | Pit mucous | COPS9 | -0.32 | 1.68E-53 |
| Pit mucous | Pit mucous | IER3 | -0.53 | 2.48E-53 |
| Pit mucous | Pit mucous | PLP2 | -0.34 | 3.15E-53 |
| Pit mucous | Pit mucous | FXYD5 | -0.37 | 3.34E-53 |
| Pit mucous | Pit mucous | TCEAL9 | -0.43 | 4.78E-53 |
| Pit mucous | Pit mucous | RAB1A | -0.31 | 6.41E-53 |
| Pit mucous | Pit mucous | NOP10 | -0.31 | 6.72E-53 |
| Pit mucous | Pit mucous | VKORC1 | -0.3 | 9.05E-53 |
| Pit mucous | Pit mucous | TACSTD2 | -0.51 | 1.38E-52 |
| Pit mucous | Pit mucous | PHGR1 | -0.35 | 2.03E-52 |
| Pit mucous | Pit mucous | SOX4 | 0.49 | 2.15E-52 |
| Pit mucous | Pit mucous | DYNLRB1 | -0.33 | 9.70E-52 |
| Pit mucous | Pit mucous | ATP5MC2 | -0.27 | 1.00E-51 |
| Pit mucous | Pit mucous | SMIM14 | -0.38 | 1.28E-51 |
| Pit mucous | Pit mucous | ERH | -0.32 | 1.29E-51 |
| Pit mucous | Pit mucous | ARF5 | -0.32 | 1.33E-51 |
| Pit mucous | Pit mucous | YOD1 | -0.41 | 1.78E-51 |
| Pit mucous | Pit mucous | FAM107B | -0.36 | 3.62E-51 |
| Pit mucous | Pit mucous | ERBIN | -0.33 | 4.14E-51 |
| Pit mucous | Pit mucous | OSTC | -0.26 | 4.41E-51 |
| Pit mucous | Pit mucous | BTG2 | -0.47 | 5.97E-51 |
| Pit mucous | Pit mucous | PLAC8 | -0.56 | 6.35E-51 |
| Pit mucous | Pit mucous | KRTCAP2 | -0.37 | 1.07E-50 |
| Pit mucous | Pit mucous | PRSS23 | -0.46 | 2.06E-50 |
| Pit mucous | Pit mucous | TNFAIP3 | -0.46 | 3.02E-50 |
| Pit mucous | Pit mucous | TMEM134 | -0.34 | 3.31E-50 |
| Pit mucous | Pit mucous | RAC1 | -0.35 | 3.44E-50 |
| Pit mucous | Pit mucous | CSRNP1 | -0.35 | 5.78E-50 |
| Pit mucous | Pit mucous | DUT | -0.34 | 5.79E-50 |
| Pit mucous | Pit mucous | SUMO1 | -0.25 | 6.00E-50 |
| Pit mucous | Pit mucous | CAP1 | -0.34 | 6.36E-50 |
| Pit mucous | Pit mucous | TUBA1A | -0.73 | 1.66E-49 |
| Pit mucous | Pit mucous | PCBP2 | -0.31 | 2.96E-49 |
| Pit mucous | Pit mucous | GPX4 | -0.38 | 3.56E-49 |
| Pit mucous | Pit mucous | SEC11A | -0.28 | 4.13E-49 |
| Pit mucous | Pit mucous | IRF1 | -0.43 | 7.19E-49 |
| Pit mucous | Pit mucous | SYPL1 | -0.27 | 8.32E-49 |
| Pit mucous | Pit mucous | TMEM256 | -0.29 | 1.01E-48 |
| Pit mucous | Pit mucous | GPX2 | -0.53 | 1.04E-48 |
| Pit mucous | Pit mucous | HIGD1A | -0.43 | 1.11E-48 |
| Pit mucous | Pit mucous | NDUFA4 | -0.27 | 1.36E-48 |
| Pit mucous | Pit mucous | KLK1 | 0.58 | 2.91E-48 |
| Pit mucous | Pit mucous | RAP1B | -0.31 | 3.16E-48 |
| Pit mucous | Pit mucous | F2RL1 | -0.38 | 3.74E-48 |
| Pit mucous | Pit mucous | P4HB | -0.29 | 5.34E-48 |
| Pit mucous | Pit mucous | CHMP5 | -0.3 | 6.47E-48 |
| Pit mucous | Pit mucous | CDK2AP2 | -0.31 | 8.42E-48 |
| Pit mucous | Pit mucous | JTB | -0.28 | 1.07E-47 |
| Pit mucous | Pit mucous | LASP1 | -0.34 | 2.03E-47 |
| Pit mucous | Pit mucous | ATRAID | -0.27 | 4.84E-47 |
| Pit mucous | Pit mucous | ARHGDIA | -0.32 | 4.84E-47 |
| Pit mucous | Pit mucous | SELENOF | -0.27 | 5.16E-47 |
| Pit mucous | Pit mucous | SPCS1 | -0.34 | 5.76E-47 |
| Pit mucous | Pit mucous | PIM1 | -0.34 | 6.51E-47 |
| Pit mucous | Pit mucous | ATP6V1F | -0.29 | 9.94E-47 |
| Pit mucous | Pit mucous | CSTB | -0.32 | 1.25E-46 |
| Pit mucous | Pit mucous | NFKBIA | -0.43 | 1.25E-46 |
| Pit mucous | Pit mucous | ARPC5 | -0.31 | 1.73E-46 |
| Pit mucous | Pit mucous | EIF4G2 | -0.3 | 2.42E-46 |
| Pit mucous | Pit mucous | ZNF706 | -0.26 | 2.62E-46 |
| Pit mucous | Pit mucous | JUNB | -0.76 | 2.96E-46 |
| Pit mucous | Pit mucous | SPINT2 | -0.32 | 3.95E-46 |
| Pit mucous | Pit mucous | AAMDC | -0.34 | 5.28E-46 |
| Pit mucous | Pit mucous | SRP14 | -0.3 | 6.42E-46 |
| Pit mucous | Pit mucous | MAL2 | -0.38 | 7.12E-46 |
| Pit mucous | Pit mucous | HNRNPA0 | -0.34 | 1.03E-45 |
| Pit mucous | Pit mucous | RBM47 | -0.32 | 1.22E-45 |
| Pit mucous | Pit mucous | CDKN1A | -0.39 | 1.25E-45 |
| Pit mucous | Pit mucous | TMPRSS2 | -0.32 | 1.31E-45 |
| Pit mucous | Pit mucous | SLC9A1 | -0.32 | 1.40E-45 |
| Pit mucous | Pit mucous | IER2 | -0.45 | 1.69E-45 |
| Pit mucous | Pit mucous | SIK1 | -0.39 | 2.43E-45 |
| Pit mucous | Pit mucous | CAPZB | -0.31 | 3.36E-45 |
| Pit mucous | Pit mucous | PFDN5 | -0.26 | 3.82E-45 |
| Pit mucous | Pit mucous | DHRS7 | -0.3 | 3.98E-45 |
| Pit mucous | Pit mucous | TOMM7 | -0.29 | 7.08E-45 |
| Pit mucous | Pit mucous | RAP1A | -0.3 | 7.26E-45 |
| Pit mucous | Pit mucous | TSC22D1 | -0.36 | 7.77E-45 |
| Pit mucous | Pit mucous | HLA-A | -0.36 | 9.13E-45 |
| Pit mucous | Pit mucous | FAM102A | -0.34 | 1.29E-44 |
| Pit mucous | Pit mucous | UBL5 | -0.26 | 1.89E-44 |
| Pit mucous | Pit mucous | PARD6B | -0.35 | 2.11E-44 |
| Pit mucous | Pit mucous | IDS | -0.34 | 2.16E-44 |
| Pit mucous | Pit mucous | SVIP | -0.37 | 2.22E-44 |
| Pit mucous | Pit mucous | SGMS2 | -0.35 | 2.39E-44 |
| Pit mucous | Pit mucous | TRMT112 | -0.31 | 2.43E-44 |
| Pit mucous | Pit mucous | CYB5A | -0.29 | 2.64E-44 |
| Pit mucous | Pit mucous | PRR13 | -0.37 | 2.94E-44 |
| Pit mucous | Pit mucous | ARPC3 | -0.28 | 3.01E-44 |
| Pit mucous | Pit mucous | PLA2G16 | -0.28 | 3.02E-44 |
| Pit mucous | Pit mucous | AKR7A3 | -0.44 | 3.11E-44 |
| Pit mucous | Pit mucous | CLTA | -0.31 | 3.33E-44 |
| Pit mucous | Pit mucous | KRT10 | -0.34 | 5.82E-44 |
| Pit mucous | Pit mucous | ARF1 | -0.28 | 6.52E-44 |
| Pit mucous | Pit mucous | CNBP | -0.28 | 6.61E-44 |
| Pit mucous | Pit mucous | SH3BGRL2 | -0.31 | 8.50E-44 |
| Pit mucous | Pit mucous | COX17 | -0.32 | 1.55E-43 |
| Pit mucous | Pit mucous | BIRC3 | -0.39 | 1.83E-43 |
| Pit mucous | Pit mucous | TSPYL1 | -0.26 | 2.13E-43 |
| Pit mucous | Pit mucous | PCSK1N | -1.06 | 3.05E-43 |
| Pit mucous | Pit mucous | CXADR | -0.27 | 3.66E-43 |
| Pit mucous | Pit mucous | ACTR3 | -0.3 | 5.14E-43 |
| Pit mucous | Pit mucous | RGS1 | -0.92 | 5.16E-43 |
| Pit mucous | Pit mucous | IL1RN | -0.39 | 6.01E-43 |
| Pit mucous | Pit mucous | MAFF | -0.33 | 6.70E-43 |
| Pit mucous | Pit mucous | CMTM6 | -0.28 | 1.50E-42 |
| Pit mucous | Pit mucous | SUMO3 | -0.26 | 1.71E-42 |
| Pit mucous | Pit mucous | DNAJB9 | -0.39 | 2.83E-42 |
| Pit mucous | Pit mucous | GNG5 | -0.28 | 3.90E-42 |
| Pit mucous | Pit mucous | CYCS | -0.3 | 7.23E-42 |
| Pit mucous | Pit mucous | FAR1 | -0.27 | 1.07E-41 |
| Pit mucous | Pit mucous | EMP3 | -0.48 | 1.31E-41 |
| Pit mucous | Pit mucous | CALM1 | -0.38 | 1.31E-41 |
| Pit mucous | Pit mucous | KLF10 | -0.31 | 1.54E-41 |
| Pit mucous | Pit mucous | SMIM22 | -0.43 | 1.74E-41 |
| Pit mucous | Pit mucous | PERP | -0.28 | 2.15E-41 |
| Pit mucous | Pit mucous | PDE4C | -0.42 | 2.25E-41 |
| Pit mucous | Pit mucous | ABHD2 | -0.31 | 2.54E-41 |
| Pit mucous | Pit mucous | POMP | -0.28 | 2.79E-41 |
| Pit mucous | Pit mucous | MIDN | -0.35 | 2.97E-41 |
| Pit mucous | Pit mucous | RF00598 | 0.42 | 3.38E-41 |
| Pit mucous | Pit mucous | LSM4 | -0.27 | 3.75E-41 |
| Pit mucous | Pit mucous | UBE2J1 | -0.28 | 4.53E-41 |
| Pit mucous | Pit mucous | CANX | -0.26 | 6.18E-41 |
| Pit mucous | Pit mucous | LEPROT | -0.25 | 9.03E-41 |
| Pit mucous | Pit mucous | LAMTOR1 | -0.26 | 9.21E-41 |
| Pit mucous | Pit mucous | KCNE2 | -0.25 | 9.61E-41 |
| Pit mucous | Pit mucous | SLC7A8 | -0.39 | 1.36E-40 |
| Pit mucous | Pit mucous | HNRNPC | -0.25 | 1.53E-40 |
| Pit mucous | Pit mucous | ITGB1 | -0.29 | 2.49E-40 |
| Pit mucous | Pit mucous | FOSL2 | -0.29 | 2.49E-40 |
| Pit mucous | Pit mucous | FAM3D | 0.32 | 2.73E-40 |
| Pit mucous | Pit mucous | TRAM1 | -0.27 | 2.83E-40 |
| Pit mucous | Pit mucous | TM9SF3 | -0.27 | 3.10E-40 |
| Pit mucous | Pit mucous | C4orf3 | -0.27 | 5.73E-40 |
| Pit mucous | Pit mucous | PDIA6 | -0.26 | 5.88E-40 |
| Pit mucous | Pit mucous | DHCR24 | -0.28 | 7.51E-40 |
| Pit mucous | Pit mucous | KLF4 | -0.35 | 1.07E-39 |
| Pit mucous | Pit mucous | ARL6IP5 | -0.26 | 1.12E-39 |
| Pit mucous | Pit mucous | GADD45B | -0.68 | 1.12E-39 |
| Pit mucous | Pit mucous | FOXQ1 | -0.45 | 1.13E-39 |
| Pit mucous | Pit mucous | ANAPC11 | -0.27 | 1.15E-39 |
| Pit mucous | Pit mucous | HNRNPK | -0.27 | 1.49E-39 |
| Pit mucous | Pit mucous | CALR | -0.28 | 1.84E-39 |
| Pit mucous | Pit mucous | PBX1 | -0.26 | 2.51E-39 |
| Pit mucous | Pit mucous | APH1A | -0.25 | 2.94E-39 |
| Pit mucous | Pit mucous | TMEM50A | -0.26 | 2.99E-39 |
| Pit mucous | Pit mucous | CYTOR | -0.41 | 3.90E-39 |
| Pit mucous | Pit mucous | DDT | -0.27 | 4.37E-39 |
| Pit mucous | Pit mucous | SRSF2 | -0.31 | 5.76E-39 |
| Pit mucous | Pit mucous | NINJ1 | -0.25 | 6.76E-39 |
| Pit mucous | Pit mucous | SUCLG2 | -0.25 | 9.44E-39 |
| Pit mucous | Pit mucous | PTP4A2 | -0.27 | 1.05E-38 |
| Pit mucous | Pit mucous | FAM3C | -0.28 | 1.12E-38 |
| Pit mucous | Pit mucous | MARCKSL1 | -0.32 | 1.61E-38 |
| Pit mucous | Pit mucous | ANXA7 | -0.25 | 1.66E-38 |
| Pit mucous | Pit mucous | CRIP2 | -0.41 | 1.74E-38 |
| Pit mucous | Pit mucous | IFI27 | -0.45 | 1.86E-38 |
| Pit mucous | Pit mucous | SPCS3 | -0.27 | 2.21E-38 |
| Pit mucous | Pit mucous | GHITM | -0.27 | 2.99E-38 |
| Pit mucous | Pit mucous | IFNGR2 | -0.3 | 3.66E-38 |
| Pit mucous | Pit mucous | RBX1 | -0.27 | 5.02E-38 |
| Pit mucous | Pit mucous | COTL1 | -0.33 | 6.87E-38 |
| Pit mucous | Pit mucous | SLC25A5 | -0.29 | 7.60E-38 |
| Pit mucous | Pit mucous | 7-Sep | -0.29 | 7.82E-38 |
| Pit mucous | Pit mucous | ETNK1 | -0.29 | 1.27E-37 |
| Pit mucous | Pit mucous | PSAPL1 | -0.5 | 1.56E-37 |
| Pit mucous | Pit mucous | MTCH1 | -0.25 | 2.55E-37 |
| Pit mucous | Pit mucous | AC012306.2 | 0.3 | 4.67E-37 |
| Pit mucous | Pit mucous | ANXA5 | -0.28 | 6.93E-37 |
| Pit mucous | Pit mucous | PCBP1 | -0.26 | 1.68E-36 |
| Pit mucous | Pit mucous | RPL29 | 0.25 | 2.83E-36 |
| Pit mucous | Pit mucous | CSKMT | 0.31 | 4.47E-36 |
| Pit mucous | Pit mucous | XIST | -0.33 | 8.87E-36 |
| Pit mucous | Pit mucous | MIR22HG | -0.27 | 9.57E-36 |
| Pit mucous | Pit mucous | CTTN | -0.25 | 1.08E-35 |
| Pit mucous | Pit mucous | TXNRD1 | -0.29 | 1.14E-35 |
| Pit mucous | Pit mucous | TIMP1 | -0.51 | 1.30E-35 |
| Pit mucous | Pit mucous | HMGB2 | -0.37 | 1.49E-35 |
| Pit mucous | Pit mucous | B4GALT1 | -0.29 | 1.59E-35 |
| Pit mucous | Pit mucous | LMO4 | -0.31 | 1.76E-35 |
| Pit mucous | Pit mucous | SLC44A1 | -0.28 | 1.84E-35 |
| Pit mucous | Pit mucous | ISCU | -0.27 | 2.58E-35 |
| Pit mucous | Pit mucous | ZFP36L1 | -0.35 | 2.93E-35 |
| Pit mucous | Pit mucous | TMBIM1 | -0.28 | 3.24E-35 |
| Pit mucous | Pit mucous | GNAQ | -0.25 | 7.00E-35 |
| Pit mucous | Pit mucous | LDHA | -0.38 | 9.40E-35 |
| Pit mucous | Pit mucous | MUC6 | -1.18 | 1.18E-34 |
| Pit mucous | Pit mucous | SELENOK | -0.26 | 1.21E-34 |
| Pit mucous | Pit mucous | RUNX1 | -0.26 | 1.22E-34 |
| Pit mucous | Pit mucous | PTMS | -0.31 | 1.56E-34 |
| Pit mucous | Pit mucous | HLA-E | -0.39 | 1.62E-34 |
| Pit mucous | Pit mucous | SFPQ | -0.29 | 1.73E-34 |
| Pit mucous | Pit mucous | CYP2S1 | -0.35 | 1.74E-34 |
| Pit mucous | Pit mucous | HRASLS2 | -0.43 | 2.05E-34 |
| Pit mucous | Pit mucous | JAG1 | -0.27 | 2.11E-34 |
| Pit mucous | Pit mucous | ELL2 | -0.27 | 2.36E-34 |
| Pit mucous | Pit mucous | ARID5B | -0.28 | 2.47E-34 |
| Pit mucous | Pit mucous | SULT1C2 | -0.28 | 2.87E-34 |
| Pit mucous | Pit mucous | EZR | -0.29 | 3.15E-34 |
| Pit mucous | Pit mucous | RHOC | -0.38 | 5.35E-34 |
| Pit mucous | Pit mucous | ABCC5 | -0.26 | 5.44E-34 |
| Pit mucous | Pit mucous | RBM8A | -0.25 | 5.54E-34 |
| Pit mucous | Pit mucous | PRKAR1A | -0.25 | 5.97E-34 |
| Pit mucous | Pit mucous | CYBRD1 | -0.3 | 6.65E-34 |
| Pit mucous | Pit mucous | SKIL | -0.26 | 7.40E-34 |
| Pit mucous | Pit mucous | MAP1LC3B | -0.26 | 1.32E-33 |
| Pit mucous | Pit mucous | CEBPB | -0.33 | 1.34E-33 |
| Pit mucous | Pit mucous | AKR1C2 | -0.39 | 1.35E-33 |
| Pit mucous | Pit mucous | DDX3X | -0.27 | 2.08E-33 |
| Pit mucous | Pit mucous | IFI6 | -0.31 | 2.87E-33 |
| Pit mucous | Pit mucous | IFITM2 | -0.52 | 3.50E-33 |
| Pit mucous | Pit mucous | PPP1CA | -0.27 | 3.65E-33 |
| Pit mucous | Pit mucous | JCHAIN | -3.17 | 3.94E-33 |
| Pit mucous | Pit mucous | TPM4 | -0.28 | 4.26E-33 |
| Pit mucous | Pit mucous | INO80C | -0.27 | 5.04E-33 |
| Pit mucous | Pit mucous | CA9 | -0.27 | 5.28E-33 |
| Pit mucous | Pit mucous | LAMB3 | -0.37 | 6.83E-33 |
| Pit mucous | Pit mucous | TUBB | -0.34 | 1.11E-32 |
| Pit mucous | Pit mucous | GOLM1 | -0.3 | 1.49E-32 |
| Pit mucous | Pit mucous | CTSZ | -0.28 | 1.60E-32 |
| Pit mucous | Pit mucous | PAM | -0.28 | 1.81E-32 |
| Pit mucous | Pit mucous | NEDD9 | -0.26 | 2.98E-32 |
| Pit mucous | Pit mucous | IGFBP4 | -0.4 | 5.22E-32 |
| Pit mucous | Pit mucous | AKR1C1 | -0.42 | 1.29E-31 |
| Pit mucous | Pit mucous | RFLNA | -0.31 | 1.62E-31 |
| Pit mucous | Pit mucous | BHLHE40 | -0.28 | 3.26E-31 |
| Pit mucous | Pit mucous | CREM | -0.51 | 3.65E-31 |
| Pit mucous | Pit mucous | TM4SF4 | -0.55 | 5.57E-31 |
| Pit mucous | Pit mucous | ABLIM1 | -0.27 | 5.73E-31 |
| Pit mucous | Pit mucous | RAP2B | -0.31 | 5.77E-31 |
| Pit mucous | Pit mucous | ITPKC | -0.33 | 6.13E-31 |
| Pit mucous | Pit mucous | GCLC | -0.28 | 8.91E-31 |
| Pit mucous | Pit mucous | RIOK3 | -0.28 | 1.28E-30 |
| Pit mucous | Pit mucous | SCGB2A1 | -0.51 | 1.86E-30 |
| Pit mucous | Pit mucous | CLK1 | -0.26 | 2.08E-30 |
| Pit mucous | Pit mucous | SGK1 | -0.26 | 2.58E-30 |
| Pit mucous | Pit mucous | AKR1C3 | -0.33 | 5.34E-30 |
| Pit mucous | Pit mucous | SELENOP | -0.37 | 9.22E-30 |
| Pit mucous | Pit mucous | ARHGDIB | -0.46 | 4.33E-29 |
| Pit mucous | Pit mucous | ANKRD28 | -0.43 | 4.70E-29 |
| Pit mucous | Pit mucous | ATF4 | -0.26 | 5.75E-29 |
| Pit mucous | Pit mucous | RND1 | -0.27 | 1.15E-28 |
| Pit mucous | Pit mucous | CXCL8 | -0.9 | 1.31E-28 |
| Pit mucous | Pit mucous | MIR4435-2HG | -0.25 | 1.50E-28 |
| Pit mucous | Pit mucous | LEPROTL1 | -0.27 | 1.88E-28 |
| Pit mucous | Pit mucous | RAB27B | -0.32 | 2.10E-28 |
| Pit mucous | Pit mucous | RAN | -0.25 | 2.14E-28 |
| Pit mucous | Pit mucous | TOB1 | -0.25 | 2.51E-28 |
| Pit mucous | Pit mucous | EPS8 | -0.25 | 2.87E-28 |
| Pit mucous | Pit mucous | LITAF | -0.26 | 2.96E-28 |
| Pit mucous | Pit mucous | AC023090.1 | -0.31 | 2.97E-28 |
| Pit mucous | Pit mucous | SSR4 | -0.48 | 3.52E-28 |
| Pit mucous | Pit mucous | B3GNT5 | -0.26 | 1.23E-27 |
| Pit mucous | Pit mucous | ID3 | -0.47 | 1.25E-27 |
| Pit mucous | Pit mucous | ANXA1 | -0.5 | 1.84E-27 |
| Pit mucous | Pit mucous | MT-ND6 | 0.37 | 2.31E-27 |
| Pit mucous | Pit mucous | NUPR1 | -0.3 | 2.79E-27 |
| Pit mucous | Pit mucous | ZG16 | 1.74 | 4.84E-27 |
| Pit mucous | Pit mucous | GLRX | -0.28 | 5.25E-27 |
| Pit mucous | Pit mucous | PRR4 | -0.7 | 6.78E-27 |
| Pit mucous | Pit mucous | PPP1R15A | -0.29 | 6.84E-27 |
| Pit mucous | Pit mucous | PLA2G10 | -0.28 | 8.75E-27 |
| Pit mucous | Pit mucous | STMN1 | -0.33 | 1.04E-26 |
| Pit mucous | Pit mucous | OTUD1 | -0.27 | 1.39E-26 |
| Pit mucous | Pit mucous | VAMP5 | -0.28 | 2.73E-26 |
| Pit mucous | Pit mucous | UBC | -0.28 | 3.08E-26 |
| Pit mucous | Pit mucous | TSPAN1 | -0.26 | 3.12E-26 |
| Pit mucous | Pit mucous | PEBP1 | -0.28 | 3.15E-26 |
| Pit mucous | Pit mucous | SELENOW | -0.29 | 3.29E-26 |
| Pit mucous | Pit mucous | RPL17 | -0.28 | 6.80E-26 |
| Pit mucous | Pit mucous | ERO1B | -0.35 | 7.64E-26 |
| Pit mucous | Pit mucous | CXCL2 | -0.61 | 1.23E-25 |
| Pit mucous | Pit mucous | HIST1H1C | -0.26 | 1.25E-25 |
| Pit mucous | Pit mucous | EPHA2 | -0.27 | 1.66E-25 |
| Pit mucous | Pit mucous | MS4A8 | -0.28 | 2.38E-25 |
| Pit mucous | Pit mucous | RARRES3 | -0.27 | 4.98E-25 |
| Pit mucous | Pit mucous | SEC11C | -0.32 | 7.96E-25 |
| Pit mucous | Pit mucous | GALNT6 | -0.26 | 1.48E-24 |
| Pit mucous | Pit mucous | RAB11FIP1 | -0.28 | 1.94E-24 |
| Pit mucous | Pit mucous | ADIRF | -0.57 | 1.94E-24 |
| Pit mucous | Pit mucous | RHOF | -0.25 | 2.02E-24 |
| Pit mucous | Pit mucous | TENT5A | -0.27 | 2.54E-24 |
| Pit mucous | Pit mucous | FABP1 | -1.42 | 3.07E-24 |
| Pit mucous | Pit mucous | ARPC1B | -0.28 | 3.22E-24 |
| Pit mucous | Pit mucous | RARRES2 | -0.48 | 1.26E-23 |
| Pit mucous | Pit mucous | HLA-DPB1 | -0.56 | 1.96E-23 |
| Pit mucous | Pit mucous | RAMP1 | 0.28 | 2.47E-23 |
| Pit mucous | Pit mucous | C11orf86 | -0.45 | 4.90E-23 |
| Pit mucous | Pit mucous | HLA-DPA1 | -0.5 | 9.45E-23 |
| Pit mucous | Pit mucous | CAPN8 | -0.28 | 1.69E-22 |
| Pit mucous | Pit mucous | HLA-DRA | -0.73 | 1.71E-22 |
| Pit mucous | Pit mucous | MT2A | -0.5 | 3.32E-22 |
| Pit mucous | Pit mucous | LY6E | -0.26 | 1.62E-21 |
| Pit mucous | Pit mucous | TXNIP | -0.35 | 2.66E-21 |
| Pit mucous | Pit mucous | ACSL5 | -0.25 | 2.83E-21 |
| Pit mucous | Pit mucous | RRBP1 | 0.27 | 3.39E-21 |
| Pit mucous | Pit mucous | ID2 | -0.33 | 5.31E-21 |
| Pit mucous | Pit mucous | NDUFS7 | 0.33 | 5.80E-21 |
| Pit mucous | Pit mucous | HLA-DRB1 | -0.55 | 1.26E-20 |
| Pit mucous | Pit mucous | ITM2C | -0.33 | 1.81E-20 |
| Pit mucous | Pit mucous | IL32 | -0.59 | 3.10E-20 |
| Pit mucous | Pit mucous | SLC9A3R2 | -0.38 | 3.52E-20 |
| Pit mucous | Pit mucous | TMPRSS15 | -0.41 | 3.57E-20 |
| Pit mucous | Pit mucous | TMEM54 | -0.27 | 8.84E-20 |
| Pit mucous | Pit mucous | DNAJB1 | -0.36 | 2.18E-19 |
| Pit mucous | Pit mucous | DEPP1 | -0.31 | 2.19E-19 |
| Pit mucous | Pit mucous | ID1 | -0.33 | 4.97E-19 |
| Pit mucous | Pit mucous | PMP22 | -0.28 | 3.33E-18 |
| Pit mucous | Pit mucous | TCIM | 0.31 | 2.78E-17 |
| Pit mucous | Pit mucous | SOD2 | -0.33 | 4.27E-17 |
| Pit mucous | Pit mucous | SDCBP2 | -0.32 | 6.74E-17 |
| Pit mucous | Pit mucous | GADD45GIP1 | 0.29 | 1.02E-16 |
| Pit mucous | Pit mucous | TMEM176B | -0.37 | 6.33E-16 |
| Pit mucous | Pit mucous | LYZ | -0.75 | 1.91E-15 |
| Pit mucous | Pit mucous | EIF3J | 0.28 | 5.82E-15 |
| Pit mucous | Pit mucous | FABP5 | -0.48 | 1.05E-14 |
| Pit mucous | Pit mucous | PHLDA1 | -0.25 | 2.80E-14 |
| Pit mucous | Pit mucous | HLA-DQB1 | -0.26 | 2.64E-13 |
| Pit mucous | Pit mucous | TUBA1B | -0.28 | 2.88E-13 |
| Pit mucous | Pit mucous | BPIFB1 | -0.67 | 6.85E-13 |
| Pit mucous | Pit mucous | BST2 | -0.26 | 2.24E-12 |
| Pit mucous | Pit mucous | MIA | -0.31 | 3.08E-12 |
| Pit mucous | Pit mucous | RBP2 | -1.19 | 7.69E-12 |
| Pit mucous | Pit mucous | TM4SF20 | -0.32 | 1.33E-10 |
| Pit mucous | Pit mucous | CD74 | -0.61 | 6.84E-10 |
| Pit mucous | Pit mucous | JUN | -0.44 | 3.53E-09 |
| Pit mucous | Pit mucous | IFITM1 | -0.44 | 4.05E-09 |
| Pit mucous | Pit mucous | WFDC2 | 0.41 | 1.53E-08 |
| Pit mucous | Pit mucous | ALDOB | -0.5 | 6.33E-08 |
| Pit mucous | Pit mucous | GSTA1 | -0.3 | 7.19E-07 |
| Pit mucous | Pit mucous | CXCL3 | -0.5 | 1.41E-06 |
| Pit mucous | Pit mucous | RPL7 | 0.3 | 4.07E-06 |
| Pit mucous | Pit mucous | CD320 | -0.41 | 5.17E-06 |
| Pit mucous | Pit mucous | OLFM4 | 1.65 | 0 |
| Pit mucous | Pit mucous | PCLAF | 1.1 | 0 |
| Pit mucous | Pit mucous | RANBP1 | 1.07 | 0 |
| Pit mucous | Pit mucous | H2AFZ | 1.06 | 0 |
| Pit mucous | Pit mucous | HSPD1 | 1 | 0 |
| Pit mucous | Pit mucous | HSPE1 | 0.94 | 0 |
| Pit mucous | Pit mucous | IDH2 | 0.87 | 0 |
| Pit mucous | Pit mucous | PTTG1 | 0.85 | 0 |
| Pit mucous | Pit mucous | STMN1 | 0.84 | 0 |
| Pit mucous | Pit mucous | HMGB1 | 0.84 | 0 |
| Pit mucous | Pit mucous | UBE2C | 0.8 | 0 |
| Pit mucous | Pit mucous | HMGB2 | 0.79 | 0 |
| Pit mucous | Pit mucous | CDKN3 | 0.71 | 0 |
| Pit mucous | Pit mucous | RPS2 | 0.68 | 0 |
| Pit mucous | Pit mucous | RRM2 | 0.67 | 0 |
| Pit mucous | Pit mucous | TMEM106C | 0.67 | 0 |
| Pit mucous | Pit mucous | CDC20 | 0.67 | 0 |
| Pit mucous | Pit mucous | TK1 | 0.62 | 0 |
| Pit mucous | Pit mucous | REG4 | 0.61 | 0 |
| Pit mucous | Pit mucous | CPS1 | 0.59 | 0 |
| Pit mucous | Pit mucous | CCNB1 | 0.59 | 0 |
| Pit mucous | Pit mucous | DTYMK | 0.59 | 0 |
| Pit mucous | Pit mucous | BIRC5 | 0.58 | 0 |
| Pit mucous | Pit mucous | TOP2A | 0.57 | 0 |
| Pit mucous | Pit mucous | MAD2L1 | 0.55 | 0 |
| Pit mucous | Pit mucous | CENPW | 0.54 | 0 |
| Pit mucous | Pit mucous | MKI67 | 0.53 | 0 |
| Pit mucous | Pit mucous | CENPF | 0.51 | 0 |
| Pit mucous | Pit mucous | ZWINT | 0.49 | 0 |
| Pit mucous | Pit mucous | UBE2T | 0.47 | 0 |
| Pit mucous | Pit mucous | GGH | 0.45 | 0 |
| Pit mucous | Pit mucous | CENPN | 0.45 | 0 |
| Pit mucous | Pit mucous | SMC2 | 0.44 | 0 |
| Pit mucous | Pit mucous | SMC4 | 0.43 | 0 |
| Pit mucous | Pit mucous | CENPM | 0.41 | 0 |
| Pit mucous | Pit mucous | CDK1 | 0.41 | 0 |
| Pit mucous | Pit mucous | NUSAP1 | 0.41 | 0 |
| Pit mucous | Pit mucous | GINS2 | 0.39 | 0 |
| Pit mucous | Pit mucous | F12 | 0.39 | 0 |
| Pit mucous | Pit mucous | PBK | 0.37 | 0 |
| Pit mucous | Pit mucous | CENPU | 0.35 | 0 |
| Pit mucous | Pit mucous | KIF20B | 0.33 | 0 |
| Pit mucous | Pit mucous | PRC1 | 0.33 | 0 |
| Pit mucous | Pit mucous | FEN1 | 0.32 | 0 |
| Pit mucous | Pit mucous | CENPK | 0.31 | 0 |
| Pit mucous | Pit mucous | TPX2 | 0.3 | 0 |
| Pit mucous | Pit mucous | AQP1 | 0.3 | 0 |
| Pit mucous | Pit mucous | CCNB2 | 0.3 | 0 |
| Pit mucous | Pit mucous | PLK1 | 0.28 | 0 |
| Pit mucous | Pit mucous | ASF1B | 0.27 | 0 |
| Pit mucous | Pit mucous | RAD51AP1 | 0.26 | 0 |
| Pit mucous | Pit mucous | TFF1 | -2.93 | 0 |
| Pit mucous | Pit mucous | TYMS | 0.32 | 2.89E-294 |
| Pit mucous | Pit mucous | TUBA1B | 0.9 | 2.51E-291 |
| Pit mucous | Pit mucous | TUBB | 0.79 | 3.76E-278 |
| Pit mucous | Pit mucous | MCM7 | 0.42 | 1.03E-277 |
| Pit mucous | Pit mucous | TFF2 | -2.68 | 2.41E-276 |
| Pit mucous | Pit mucous | GAST | -4.42 | 1.14E-273 |
| Pit mucous | Pit mucous | VRK1 | 0.3 | 1.95E-268 |
| Pit mucous | Pit mucous | NUDT1 | 0.4 | 2.52E-267 |
| Pit mucous | Pit mucous | TRIM54 | 0.5 | 1.49E-262 |
| Pit mucous | Pit mucous | GGCT | 0.65 | 6.55E-261 |
| Pit mucous | Pit mucous | DMBT1 | 0.75 | 4.69E-260 |
| Pit mucous | Pit mucous | ENO1 | 0.77 | 7.45E-258 |
| Pit mucous | Pit mucous | PGC | -3.17 | 4.17E-256 |
| Pit mucous | Pit mucous | EXOSC8 | 0.42 | 2.64E-254 |
| Pit mucous | Pit mucous | PAICS | 0.41 | 9.84E-253 |
| Pit mucous | Pit mucous | C19orf48 | 0.51 | 5.40E-248 |
| Pit mucous | Pit mucous | CLDN4 | 0.62 | 1.51E-247 |
| Pit mucous | Pit mucous | CCDC34 | 0.44 | 1.64E-246 |
| Pit mucous | Pit mucous | CENPX | 0.71 | 3.01E-236 |
| Pit mucous | Pit mucous | PCNA | 0.56 | 4.55E-236 |
| Pit mucous | Pit mucous | MUC5AC | -2.24 | 1.52E-234 |
| Pit mucous | Pit mucous | HELLS | 0.3 | 7.61E-234 |
| Pit mucous | Pit mucous | HMGN2 | 0.75 | 1.09E-229 |
| Pit mucous | Pit mucous | HMGB3 | 0.39 | 5.72E-227 |
| Pit mucous | Pit mucous | GKN1 | -3.96 | 3.43E-225 |
| Pit mucous | Pit mucous | KPNA2 | 0.51 | 7.46E-225 |
| Pit mucous | Pit mucous | DHFR | 0.32 | 3.39E-224 |
| Pit mucous | Pit mucous | CLDN7 | 0.53 | 1.19E-222 |
| Pit mucous | Pit mucous | TXN | 0.7 | 1.50E-220 |
| Pit mucous | Pit mucous | KRT18 | 0.63 | 1.58E-220 |
| Pit mucous | Pit mucous | NME1 | 0.59 | 2.25E-220 |
| Pit mucous | Pit mucous | RAN | 0.71 | 1.06E-219 |
| Pit mucous | Pit mucous | GAPDH | 0.61 | 9.90E-218 |
| Pit mucous | Pit mucous | EIF4EBP1 | 0.55 | 1.17E-216 |
| Pit mucous | Pit mucous | HSP90AA1 | 0.6 | 1.49E-216 |
| Pit mucous | Pit mucous | PTMA | 0.55 | 2.54E-213 |
| Pit mucous | Pit mucous | CACYBP | 0.48 | 1.51E-212 |
| Pit mucous | Pit mucous | CYC1 | 0.65 | 6.12E-209 |
| Pit mucous | Pit mucous | RRM1 | 0.31 | 6.14E-208 |
| Pit mucous | Pit mucous | ATP5MC1 | 0.65 | 7.41E-207 |
| Pit mucous | Pit mucous | LSM5 | 0.64 | 3.05E-205 |
| Pit mucous | Pit mucous | CLDN18 | -1.36 | 1.17E-203 |
| Pit mucous | Pit mucous | PHB | 0.6 | 2.54E-201 |
| Pit mucous | Pit mucous | DKC1 | 0.36 | 4.15E-201 |
| Pit mucous | Pit mucous | TMSB4X | -1.03 | 6.06E-201 |
| Pit mucous | Pit mucous | DEK | 0.54 | 1.18E-199 |
| Pit mucous | Pit mucous | PA2G4 | 0.56 | 1.62E-199 |
| Pit mucous | Pit mucous | CKS2 | 0.57 | 3.14E-198 |
| Pit mucous | Pit mucous | CCT2 | 0.45 | 7.24E-193 |
| Pit mucous | Pit mucous | CCL25 | 0.73 | 1.81E-191 |
| Pit mucous | Pit mucous | CKS1B | 0.48 | 1.62E-190 |
| Pit mucous | Pit mucous | MRPL12 | 0.58 | 5.49E-187 |
| Pit mucous | Pit mucous | MT-CO2 | 0.55 | 5.88E-187 |
| Pit mucous | Pit mucous | REG1A | 1.18 | 1.19E-186 |
| Pit mucous | Pit mucous | CLDN3 | 0.5 | 1.27E-186 |
| Pit mucous | Pit mucous | ATP5MC3 | 0.55 | 7.04E-186 |
| Pit mucous | Pit mucous | NOP58 | 0.4 | 1.95E-184 |
| Pit mucous | Pit mucous | GKN2 | -2.79 | 1.75E-182 |
| Pit mucous | Pit mucous | AGR3 | 0.66 | 2.04E-181 |
| Pit mucous | Pit mucous | GMNN | 0.3 | 1.53E-179 |
| Pit mucous | Pit mucous | RPA3 | 0.45 | 1.58E-179 |
| Pit mucous | Pit mucous | HSPA8 | 0.6 | 6.02E-179 |
| Pit mucous | Pit mucous | HIST1H4C | 1 | 2.28E-177 |
| Pit mucous | Pit mucous | PRDX2 | 0.57 | 1.99E-176 |
| Pit mucous | Pit mucous | CYSTM1 | -0.92 | 2.23E-176 |
| Pit mucous | Pit mucous | TPRKB | 0.47 | 3.57E-173 |
| Pit mucous | Pit mucous | DNAJC9 | 0.26 | 7.85E-173 |
| Pit mucous | Pit mucous | ITGB3BP | 0.3 | 2.57E-171 |
| Pit mucous | Pit mucous | MSMB | -1.63 | 3.90E-171 |
| Pit mucous | Pit mucous | MUC1 | -1.37 | 7.68E-171 |
| Pit mucous | Pit mucous | SERPINB5 | 0.46 | 3.30E-170 |
| Pit mucous | Pit mucous | PSCA | -2.15 | 2.82E-168 |
| Pit mucous | Pit mucous | RPS3 | 0.4 | 5.07E-168 |
| Pit mucous | Pit mucous | EIF2S1 | 0.37 | 1.27E-167 |
| Pit mucous | Pit mucous | DCTPP1 | 0.44 | 1.54E-166 |
| Pit mucous | Pit mucous | LSM3 | 0.59 | 4.23E-166 |
| Pit mucous | Pit mucous | PSMG1 | 0.31 | 1.12E-165 |
| Pit mucous | Pit mucous | TCP1 | 0.45 | 2.51E-165 |
| Pit mucous | Pit mucous | RPL7 | 0.84 | 6.44E-165 |
| Pit mucous | Pit mucous | RPS18 | 0.39 | 1.64E-164 |
| Pit mucous | Pit mucous | MCM3 | 0.28 | 1.85E-163 |
| Pit mucous | Pit mucous | HINT1 | 0.54 | 1.91E-163 |
| Pit mucous | Pit mucous | BRIX1 | 0.29 | 1.29E-162 |
| Pit mucous | Pit mucous | CCT6A | 0.47 | 1.56E-160 |
| Pit mucous | Pit mucous | PPP1R1B | 0.44 | 5.60E-160 |
| Pit mucous | Pit mucous | VPS29 | 0.53 | 1.58E-159 |
| Pit mucous | Pit mucous | C1QBP | 0.51 | 1.94E-159 |
| Pit mucous | Pit mucous | SSBP1 | 0.53 | 1.98E-158 |
| Pit mucous | Pit mucous | MGST1 | 0.57 | 2.78E-158 |
| Pit mucous | Pit mucous | TIMM13 | 0.53 | 3.08E-158 |
| Pit mucous | Pit mucous | SLC25A5 | 0.7 | 7.20E-158 |
| Pit mucous | Pit mucous | NPM1 | 0.66 | 1.17E-156 |
| Pit mucous | Pit mucous | DNMT1 | 0.28 | 1.26E-156 |
| Pit mucous | Pit mucous | NHP2 | 0.51 | 1.04E-155 |
| Pit mucous | Pit mucous | GCHFR | 0.33 | 5.89E-155 |
| Pit mucous | Pit mucous | BUB3 | 0.37 | 1.60E-154 |
| Pit mucous | Pit mucous | FABP5 | 0.33 | 3.09E-153 |
| Pit mucous | Pit mucous | TPI1 | 0.54 | 1.14E-152 |
| Pit mucous | Pit mucous | SNRPD1 | 0.49 | 1.04E-151 |
| Pit mucous | Pit mucous | GP2 | 0.34 | 2.11E-150 |
| Pit mucous | Pit mucous | ANP32E | 0.29 | 2.30E-149 |
| Pit mucous | Pit mucous | CBX3 | 0.46 | 3.31E-149 |
| Pit mucous | Pit mucous | MTCH2 | 0.46 | 3.86E-149 |
| Pit mucous | Pit mucous | WDR34 | 0.38 | 1.07E-148 |
| Pit mucous | Pit mucous | CST3 | -0.76 | 1.11E-148 |
| Pit mucous | Pit mucous | MRPS25 | 0.4 | 1.78E-148 |
| Pit mucous | Pit mucous | SNRPA1 | 0.3 | 1.86E-147 |
| Pit mucous | Pit mucous | MT-ATP6 | 0.49 | 4.62E-147 |
| Pit mucous | Pit mucous | MRPL13 | 0.45 | 1.53E-146 |
| Pit mucous | Pit mucous | OTC | 0.3 | 2.32E-146 |
| Pit mucous | Pit mucous | EEF1B2 | 0.49 | 5.17E-146 |
| Pit mucous | Pit mucous | MRPL15 | 0.34 | 2.07E-144 |
| Pit mucous | Pit mucous | PRKDC | 0.31 | 1.69E-143 |
| Pit mucous | Pit mucous | NAE1 | 0.29 | 1.22E-142 |
| Pit mucous | Pit mucous | VDAC3 | 0.45 | 1.81E-142 |
| Pit mucous | Pit mucous | EPCAM | 0.47 | 5.60E-142 |
| Pit mucous | Pit mucous | C20orf27 | 0.26 | 1.02E-139 |
| Pit mucous | Pit mucous | LBR | 0.31 | 1.48E-139 |
| Pit mucous | Pit mucous | LDHB | 0.63 | 1.73E-139 |
| Pit mucous | Pit mucous | RUVBL2 | 0.3 | 1.31E-138 |
| Pit mucous | Pit mucous | FXYD3 | -0.85 | 1.66E-138 |
| Pit mucous | Pit mucous | GSTM4 | 0.3 | 6.66E-138 |
| Pit mucous | Pit mucous | SNRPB | 0.5 | 8.09E-138 |
| Pit mucous | Pit mucous | PPDPF | -0.74 | 6.28E-137 |
| Pit mucous | Pit mucous | CDC123 | 0.32 | 1.02E-136 |
| Pit mucous | Pit mucous | UQCRH | 0.47 | 1.17E-136 |
| Pit mucous | Pit mucous | AHCY | 0.43 | 1.26E-136 |
| Pit mucous | Pit mucous | LSM4 | 0.49 | 2.13E-136 |
| Pit mucous | Pit mucous | RPS19 | 0.39 | 3.54E-136 |
| Pit mucous | Pit mucous | VSIG2 | -1 | 3.73E-136 |
| Pit mucous | Pit mucous | FARSB | 0.29 | 7.99E-136 |
| Pit mucous | Pit mucous | SNORC | 0.26 | 1.89E-135 |
| Pit mucous | Pit mucous | SNRPG | 0.52 | 1.55E-133 |
| Pit mucous | Pit mucous | STOML2 | 0.45 | 8.27E-133 |
| Pit mucous | Pit mucous | UCHL3 | 0.37 | 5.58E-132 |
| Pit mucous | Pit mucous | SIGMAR1 | 0.28 | 2.41E-131 |
| Pit mucous | Pit mucous | MRPL16 | 0.29 | 2.49E-131 |
| Pit mucous | Pit mucous | ANP32B | 0.42 | 4.87E-131 |
| Pit mucous | Pit mucous | CLDN15 | 0.32 | 6.69E-131 |
| Pit mucous | Pit mucous | PKM | 0.51 | 1.24E-130 |
| Pit mucous | Pit mucous | RPS5 | 0.38 | 3.77E-130 |
| Pit mucous | Pit mucous | POLD2 | 0.33 | 6.49E-130 |
| Pit mucous | Pit mucous | S100P | -1.29 | 2.27E-129 |
| Pit mucous | Pit mucous | GNL3 | 0.31 | 3.02E-129 |
| Pit mucous | Pit mucous | MRPS26 | 0.36 | 8.14E-129 |
| Pit mucous | Pit mucous | HOXB7 | 0.25 | 2.99E-128 |
| Pit mucous | Pit mucous | JUND | -0.81 | 4.38E-128 |
| Pit mucous | Pit mucous | ANXA2 | 0.48 | 6.56E-128 |
| Pit mucous | Pit mucous | EEF1E1 | 0.28 | 2.39E-127 |
| Pit mucous | Pit mucous | SNRPE | 0.47 | 6.04E-127 |
| Pit mucous | Pit mucous | HMGN1 | 0.51 | 8.42E-127 |
| Pit mucous | Pit mucous | IMPDH2 | 0.44 | 3.29E-126 |
| Pit mucous | Pit mucous | HNRNPA1 | 0.47 | 1.25E-125 |
| Pit mucous | Pit mucous | PSMD14 | 0.34 | 1.68E-125 |
| Pit mucous | Pit mucous | NDUFA9 | 0.38 | 1.83E-125 |
| Pit mucous | Pit mucous | ATP5F1A | 0.51 | 2.87E-125 |
| Pit mucous | Pit mucous | HSP90AB1 | 0.48 | 4.48E-125 |
| Pit mucous | Pit mucous | MLEC | 0.4 | 2.40E-124 |
| Pit mucous | Pit mucous | SUCLG1 | 0.4 | 4.10E-124 |
| Pit mucous | Pit mucous | MRPS12 | 0.39 | 2.13E-123 |
| Pit mucous | Pit mucous | ATP5F1C | 0.5 | 4.16E-123 |
| Pit mucous | Pit mucous | PLEK2 | 0.31 | 2.97E-122 |
| Pit mucous | Pit mucous | PPA1 | 0.53 | 6.51E-122 |
| Pit mucous | Pit mucous | DPEP1 | 0.28 | 1.02E-121 |
| Pit mucous | Pit mucous | PIGR | 0.45 | 2.29E-120 |
| Pit mucous | Pit mucous | TRAP1 | 0.27 | 3.19E-120 |
| Pit mucous | Pit mucous | PSMA4 | 0.46 | 4.82E-120 |
| Pit mucous | Pit mucous | KLK1 | 0.27 | 6.89E-120 |
| Pit mucous | Pit mucous | MRPL4 | 0.34 | 1.01E-119 |
| Pit mucous | Pit mucous | RPS4Y1 | 0.48 | 1.64E-119 |
| Pit mucous | Pit mucous | H2AFV | 0.39 | 8.26E-119 |
| Pit mucous | Pit mucous | HSPBP1 | 0.29 | 1.65E-118 |
| Pit mucous | Pit mucous | NOP56 | 0.29 | 6.33E-118 |
| Pit mucous | Pit mucous | RPL8 | 0.37 | 9.68E-118 |
| Pit mucous | Pit mucous | CCT3 | 0.38 | 3.36E-117 |
| Pit mucous | Pit mucous | ABHD11 | 0.28 | 4.08E-117 |
| Pit mucous | Pit mucous | RPS3A | 0.47 | 6.91E-117 |
| Pit mucous | Pit mucous | CKLF | 0.29 | 7.32E-117 |
| Pit mucous | Pit mucous | MRPL19 | 0.28 | 1.10E-116 |
| Pit mucous | Pit mucous | CCT5 | 0.35 | 1.93E-116 |
| Pit mucous | Pit mucous | PSMA3 | 0.4 | 3.47E-116 |
| Pit mucous | Pit mucous | QTRT1 | 0.28 | 4.09E-116 |
| Pit mucous | Pit mucous | C1orf35 | 0.28 | 4.94E-116 |
| Pit mucous | Pit mucous | CNIH4 | 0.36 | 2.19E-115 |
| Pit mucous | Pit mucous | MRPS35 | 0.33 | 2.61E-115 |
| Pit mucous | Pit mucous | SSB | 0.31 | 3.43E-115 |
| Pit mucous | Pit mucous | CASP6 | 0.26 | 1.33E-114 |
| Pit mucous | Pit mucous | UQCRC1 | 0.4 | 3.97E-114 |
| Pit mucous | Pit mucous | TOMM40 | 0.25 | 6.70E-114 |
| Pit mucous | Pit mucous | PSME2 | 0.41 | 1.42E-113 |
| Pit mucous | Pit mucous | ATP1B3 | 0.29 | 2.95E-113 |
| Pit mucous | Pit mucous | HPRT1 | 0.27 | 7.53E-113 |
| Pit mucous | Pit mucous | BOLA3 | 0.36 | 9.18E-112 |
| Pit mucous | Pit mucous | TUFM | 0.44 | 2.13E-111 |
| Pit mucous | Pit mucous | RPL10A | 0.36 | 2.19E-111 |
| Pit mucous | Pit mucous | SUPT16H | 0.26 | 3.60E-111 |
| Pit mucous | Pit mucous | SRPK1 | 0.27 | 6.48E-111 |
| Pit mucous | Pit mucous | NDUFAB1 | 0.46 | 1.13E-110 |
| Pit mucous | Pit mucous | MRPS34 | 0.38 | 3.12E-110 |
| Pit mucous | Pit mucous | ETHE1 | 0.38 | 6.29E-110 |
| Pit mucous | Pit mucous | COA3 | 0.41 | 1.16E-109 |
| Pit mucous | Pit mucous | UQCRFS1 | 0.46 | 2.14E-109 |
| Pit mucous | Pit mucous | PGAM1 | 0.4 | 3.27E-109 |
| Pit mucous | Pit mucous | RNASE1 | -1.15 | 1.48E-108 |
| Pit mucous | Pit mucous | ILF2 | 0.34 | 2.82E-108 |
| Pit mucous | Pit mucous | HADH | 0.3 | 5.44E-108 |
| Pit mucous | Pit mucous | XRCC5 | 0.33 | 1.55E-107 |
| Pit mucous | Pit mucous | CCT4 | 0.36 | 1.93E-107 |
| Pit mucous | Pit mucous | ECHS1 | 0.41 | 2.36E-107 |
| Pit mucous | Pit mucous | EBNA1BP2 | 0.26 | 1.12E-106 |
| Pit mucous | Pit mucous | NASP | 0.26 | 1.43E-106 |
| Pit mucous | Pit mucous | CD9 | 0.49 | 1.65E-106 |
| Pit mucous | Pit mucous | HSPA9 | 0.32 | 2.91E-106 |
| Pit mucous | Pit mucous | PPIA | 0.37 | 4.69E-106 |
| Pit mucous | Pit mucous | RPLP0 | 0.34 | 5.89E-106 |
| Pit mucous | Pit mucous | MRPL37 | 0.31 | 6.45E-106 |
| Pit mucous | Pit mucous | NDUFV1 | 0.36 | 6.58E-106 |
| Pit mucous | Pit mucous | GADD45GIP1 | 0.43 | 1.68E-105 |
| Pit mucous | Pit mucous | S100A10 | 0.33 | 6.86E-105 |
| Pit mucous | Pit mucous | NDUFS8 | 0.42 | 7.83E-105 |
| Pit mucous | Pit mucous | MRPL47 | 0.3 | 9.23E-105 |
| Pit mucous | Pit mucous | SERF2 | -0.43 | 7.69E-104 |
| Pit mucous | Pit mucous | MDH1 | 0.36 | 8.35E-104 |
| Pit mucous | Pit mucous | CCT7 | 0.33 | 8.51E-104 |
| Pit mucous | Pit mucous | RSL1D1 | 0.39 | 1.66E-103 |
| Pit mucous | Pit mucous | SLC25A39 | 0.39 | 5.85E-103 |
| Pit mucous | Pit mucous | CCT8 | 0.37 | 1.56E-102 |
| Pit mucous | Pit mucous | KRTCAP3 | 0.32 | 2.64E-102 |
| Pit mucous | Pit mucous | MRPL1 | 0.26 | 3.18E-102 |
| Pit mucous | Pit mucous | SDHAF3 | 0.28 | 5.64E-102 |
| Pit mucous | Pit mucous | MRPS7 | 0.29 | 5.69E-102 |
| Pit mucous | Pit mucous | SIVA1 | 0.37 | 7.04E-102 |
| Pit mucous | Pit mucous | RPL21 | 0.41 | 5.83E-101 |
| Pit mucous | Pit mucous | HNRNPA3 | 0.42 | 1.02E-100 |
| Pit mucous | Pit mucous | MRPL3 | 0.31 | 1.39E-100 |
| Pit mucous | Pit mucous | MUCL3 | -1.36 | 1.79E-100 |
| Pit mucous | Pit mucous | RPS6 | 0.33 | 2.22E-100 |
| Pit mucous | Pit mucous | RAMP1 | 0.31 | 5.04E-100 |
| Pit mucous | Pit mucous | ATP5PF | 0.41 | 4.25E-99 |
| Pit mucous | Pit mucous | SNRPF | 0.39 | 8.77E-99 |
| Pit mucous | Pit mucous | PRMT1 | 0.29 | 1.11E-98 |
| Pit mucous | Pit mucous | NCL | 0.4 | 1.71E-98 |
| Pit mucous | Pit mucous | AK6 | 0.38 | 1.90E-98 |
| Pit mucous | Pit mucous | CDK4 | 0.27 | 2.18E-98 |
| Pit mucous | Pit mucous | KLF6 | -0.64 | 2.49E-98 |
| Pit mucous | Pit mucous | TRIAP1 | 0.25 | 2.50E-98 |
| Pit mucous | Pit mucous | ATP5PB | 0.42 | 2.50E-98 |
| Pit mucous | Pit mucous | PPA2 | 0.31 | 5.47E-98 |
| Pit mucous | Pit mucous | FAM136A | 0.27 | 1.10E-97 |
| Pit mucous | Pit mucous | NUDCD2 | 0.25 | 1.77E-97 |
| Pit mucous | Pit mucous | SMIM22 | -0.76 | 7.71E-97 |
| Pit mucous | Pit mucous | MRPL50 | 0.26 | 1.23E-96 |
| Pit mucous | Pit mucous | CBWD1 | 0.29 | 1.24E-96 |
| Pit mucous | Pit mucous | MT-CYB | 0.37 | 2.42E-96 |
| Pit mucous | Pit mucous | FBL | 0.34 | 4.94E-96 |
| Pit mucous | Pit mucous | PHB2 | 0.33 | 5.31E-96 |
| Pit mucous | Pit mucous | SRI | 0.41 | 6.95E-96 |
| Pit mucous | Pit mucous | MRPL11 | 0.29 | 7.64E-96 |
| Pit mucous | Pit mucous | TPT1 | -0.46 | 8.57E-96 |
| Pit mucous | Pit mucous | EMP2 | 0.3 | 1.26E-95 |
| Pit mucous | Pit mucous | CARHSP1 | 0.28 | 1.52E-94 |
| Pit mucous | Pit mucous | SNRNP25 | 0.26 | 2.44E-94 |
| Pit mucous | Pit mucous | MINOS1 | 0.4 | 5.05E-94 |
| Pit mucous | Pit mucous | MT-ND4 | 0.35 | 9.87E-94 |
| Pit mucous | Pit mucous | PRDX3 | 0.4 | 9.99E-94 |
| Pit mucous | Pit mucous | RPL6 | 0.33 | 1.12E-93 |
| Pit mucous | Pit mucous | EIF2S2 | 0.39 | 1.12E-93 |
| Pit mucous | Pit mucous | HDAC2 | 0.3 | 1.12E-93 |
| Pit mucous | Pit mucous | PPM1G | 0.27 | 1.32E-93 |
| Pit mucous | Pit mucous | CLNS1A | 0.32 | 1.87E-93 |
| Pit mucous | Pit mucous | MRPL32 | 0.28 | 8.47E-93 |
| Pit mucous | Pit mucous | SERBP1 | 0.38 | 8.55E-93 |
| Pit mucous | Pit mucous | PSMA7 | 0.39 | 1.63E-92 |
| Pit mucous | Pit mucous | EBP | 0.28 | 3.22E-92 |
| Pit mucous | Pit mucous | GCSH | 0.25 | 5.35E-92 |
| Pit mucous | Pit mucous | LGALS3 | 0.3 | 8.29E-92 |
| Pit mucous | Pit mucous | PRDX4 | 0.27 | 8.49E-92 |
| Pit mucous | Pit mucous | COX5A | 0.38 | 9.74E-92 |
| Pit mucous | Pit mucous | MRPL42 | 0.33 | 5.69E-91 |
| Pit mucous | Pit mucous | XRN2 | 0.27 | 1.23E-90 |
| Pit mucous | Pit mucous | RBBP7 | 0.25 | 1.83E-90 |
| Pit mucous | Pit mucous | MPC2 | 0.39 | 2.86E-90 |
| Pit mucous | Pit mucous | ATP5MPL | 0.37 | 3.31E-90 |
| Pit mucous | Pit mucous | DDX21 | 0.31 | 1.98E-89 |
| Pit mucous | Pit mucous | XRCC6 | 0.29 | 2.22E-89 |
| Pit mucous | Pit mucous | PEBP1 | 0.4 | 3.37E-89 |
| Pit mucous | Pit mucous | IFI27 | -0.84 | 7.48E-89 |
| Pit mucous | Pit mucous | CTSE | -0.77 | 1.89E-88 |
| Pit mucous | Pit mucous | HMGN5 | 0.26 | 2.05E-88 |
| Pit mucous | Pit mucous | LDHA | 0.37 | 2.63E-88 |
| Pit mucous | Pit mucous | PRELID1 | 0.43 | 3.44E-88 |
| Pit mucous | Pit mucous | HSPH1 | 0.3 | 3.48E-88 |
| Pit mucous | Pit mucous | ALG5 | 0.27 | 5.30E-88 |
| Pit mucous | Pit mucous | SST | -2.75 | 6.65E-88 |
| Pit mucous | Pit mucous | GSN | -0.95 | 1.11E-87 |
| Pit mucous | Pit mucous | BANF1 | 0.36 | 3.06E-87 |
| Pit mucous | Pit mucous | PSMD7 | 0.29 | 5.04E-87 |
| Pit mucous | Pit mucous | NDUFS7 | 0.36 | 7.97E-87 |
| Pit mucous | Pit mucous | SOX4 | 0.41 | 1.32E-86 |
| Pit mucous | Pit mucous | NAA20 | 0.27 | 1.53E-86 |
| Pit mucous | Pit mucous | PSMC3 | 0.29 | 2.32E-86 |
| Pit mucous | Pit mucous | NUTF2 | 0.28 | 2.49E-86 |
| Pit mucous | Pit mucous | MRPS16 | 0.26 | 5.89E-86 |
| Pit mucous | Pit mucous | IMP4 | 0.27 | 6.12E-86 |
| Pit mucous | Pit mucous | EIF3M | 0.37 | 8.48E-86 |
| Pit mucous | Pit mucous | ENY2 | 0.35 | 3.22E-85 |
| Pit mucous | Pit mucous | RPS27A | 0.35 | 7.20E-85 |
| Pit mucous | Pit mucous | ETFB | 0.35 | 9.89E-85 |
| Pit mucous | Pit mucous | UQCRC2 | 0.37 | 2.18E-84 |
| Pit mucous | Pit mucous | ACP1 | 0.31 | 3.78E-84 |
| Pit mucous | Pit mucous | CIAO2A | 0.35 | 4.68E-84 |
| Pit mucous | Pit mucous | DNPH1 | 0.35 | 6.25E-84 |
| Pit mucous | Pit mucous | GLRX3 | 0.27 | 1.52E-83 |
| Pit mucous | Pit mucous | OLA1 | 0.26 | 1.93E-83 |
| Pit mucous | Pit mucous | THOC7 | 0.27 | 6.17E-83 |
| Pit mucous | Pit mucous | CISD1 | 0.28 | 6.93E-83 |
| Pit mucous | Pit mucous | NDUFS2 | 0.28 | 7.95E-83 |
| Pit mucous | Pit mucous | RPS23 | 0.29 | 9.43E-83 |
| Pit mucous | Pit mucous | NUCKS1 | 0.28 | 2.15E-82 |
| Pit mucous | Pit mucous | YWHAQ | 0.35 | 3.66E-82 |
| Pit mucous | Pit mucous | PYCARD | 0.31 | 7.28E-82 |
| Pit mucous | Pit mucous | AURKAIP1 | 0.38 | 8.57E-82 |
| Pit mucous | Pit mucous | LGALS4 | 0.26 | 9.91E-82 |
| Pit mucous | Pit mucous | SQSTM1 | -0.71 | 1.01E-81 |
| Pit mucous | Pit mucous | FABP2 | -0.53 | 1.18E-81 |
| Pit mucous | Pit mucous | TKT | 0.31 | 3.87E-81 |
| Pit mucous | Pit mucous | ESD | 0.32 | 6.00E-81 |
| Pit mucous | Pit mucous | ISOC2 | 0.31 | 4.23E-80 |
| Pit mucous | Pit mucous | SYNCRIP | 0.28 | 5.10E-80 |
| Pit mucous | Pit mucous | B2M | -0.66 | 5.47E-80 |
| Pit mucous | Pit mucous | HACD3 | 0.28 | 8.21E-80 |
| Pit mucous | Pit mucous | TECR | 0.3 | 9.12E-80 |
| Pit mucous | Pit mucous | UBE2V2 | 0.28 | 2.73E-79 |
| Pit mucous | Pit mucous | CA2 | -0.94 | 4.84E-79 |
| Pit mucous | Pit mucous | CRIP1 | -0.84 | 6.19E-79 |
| Pit mucous | Pit mucous | LSM6 | 0.26 | 7.85E-79 |
| Pit mucous | Pit mucous | PTGES3 | 0.39 | 9.77E-79 |
| Pit mucous | Pit mucous | HNRNPR | 0.26 | 1.64E-78 |
| Pit mucous | Pit mucous | BTG1 | -0.86 | 1.65E-78 |
| Pit mucous | Pit mucous | TIMM10 | 0.26 | 3.04E-78 |
| Pit mucous | Pit mucous | ERH | 0.38 | 4.90E-78 |
| Pit mucous | Pit mucous | BTF3 | 0.39 | 1.37E-77 |
| Pit mucous | Pit mucous | EIF5A | 0.33 | 1.50E-77 |
| Pit mucous | Pit mucous | AIMP1 | 0.28 | 3.51E-76 |
| Pit mucous | Pit mucous | CXCL17 | -0.81 | 3.89E-76 |
| Pit mucous | Pit mucous | H2AFY | 0.33 | 4.74E-76 |
| Pit mucous | Pit mucous | HMGA1 | 0.37 | 4.86E-76 |
| Pit mucous | Pit mucous | RPL22L1 | 0.35 | 6.07E-76 |
| Pit mucous | Pit mucous | PDCD5 | 0.29 | 6.57E-76 |
| Pit mucous | Pit mucous | C19orf33 | -0.72 | 1.76E-75 |
| Pit mucous | Pit mucous | SDHD | 0.32 | 4.28E-75 |
| Pit mucous | Pit mucous | ATP5F1B | 0.39 | 4.28E-75 |
| Pit mucous | Pit mucous | MKKS | 0.26 | 7.80E-75 |
| Pit mucous | Pit mucous | MRPL18 | 0.28 | 1.99E-74 |
| Pit mucous | Pit mucous | LSM7 | 0.31 | 2.17E-74 |
| Pit mucous | Pit mucous | MRPS33 | 0.27 | 3.14E-74 |
| Pit mucous | Pit mucous | MT-CO1 | 0.27 | 4.52E-74 |
| Pit mucous | Pit mucous | FOSB | -0.91 | 4.69E-74 |
| Pit mucous | Pit mucous | MRPS15 | 0.26 | 1.08E-73 |
| Pit mucous | Pit mucous | KIF5B | 0.28 | 1.17E-73 |
| Pit mucous | Pit mucous | KLF2 | -0.92 | 1.33E-73 |
| Pit mucous | Pit mucous | MDH2 | 0.36 | 2.34E-73 |
| Pit mucous | Pit mucous | MRPL51 | 0.32 | 4.72E-73 |
| Pit mucous | Pit mucous | ADRM1 | 0.28 | 2.27E-72 |
| Pit mucous | Pit mucous | DDX18 | 0.26 | 2.43E-72 |
| Pit mucous | Pit mucous | COX4I1 | 0.26 | 6.71E-72 |
| Pit mucous | Pit mucous | MGST2 | 0.33 | 8.34E-72 |
| Pit mucous | Pit mucous | COX7B | 0.36 | 2.82E-71 |
| Pit mucous | Pit mucous | CD63 | -0.47 | 2.93E-71 |
| Pit mucous | Pit mucous | HNRNPA2B1 | 0.32 | 3.64E-71 |
| Pit mucous | Pit mucous | HNRNPM | 0.3 | 3.83E-71 |
| Pit mucous | Pit mucous | UQCC3 | 0.27 | 4.38E-71 |
| Pit mucous | Pit mucous | PSMB5 | 0.31 | 6.53E-71 |
| Pit mucous | Pit mucous | SERPINB6 | 0.26 | 1.51E-70 |
| Pit mucous | Pit mucous | HMGCS2 | 0.26 | 1.87E-69 |
| Pit mucous | Pit mucous | VDAC1 | 0.39 | 2.51E-69 |
| Pit mucous | Pit mucous | RBM3 | 0.36 | 2.65E-69 |
| Pit mucous | Pit mucous | NDUFB3 | 0.32 | 2.73E-69 |
| Pit mucous | Pit mucous | SMS | 0.25 | 5.26E-69 |
| Pit mucous | Pit mucous | HLA-B | -0.54 | 6.80E-69 |
| Pit mucous | Pit mucous | ISG20 | -0.76 | 7.98E-69 |
| Pit mucous | Pit mucous | AP2S1 | 0.32 | 9.97E-69 |
| Pit mucous | Pit mucous | UBE2S | 0.29 | 1.14E-68 |
| Pit mucous | Pit mucous | DUSP1 | -0.88 | 1.31E-68 |
| Pit mucous | Pit mucous | NDUFA12 | 0.28 | 1.91E-68 |
| Pit mucous | Pit mucous | STRAP | 0.28 | 3.87E-68 |
| Pit mucous | Pit mucous | PSMB2 | 0.27 | 4.51E-68 |
| Pit mucous | Pit mucous | PSMA5 | 0.27 | 9.96E-68 |
| Pit mucous | Pit mucous | DUT | 0.29 | 5.05E-67 |
| Pit mucous | Pit mucous | SAT1 | -0.52 | 7.40E-67 |
| Pit mucous | Pit mucous | JCHAIN | -3.21 | 1.78E-66 |
| Pit mucous | Pit mucous | EIF3I | 0.3 | 2.08E-66 |
| Pit mucous | Pit mucous | METTL26 | 0.25 | 2.26E-66 |
| Pit mucous | Pit mucous | HLA-A | -0.56 | 4.55E-66 |
| Pit mucous | Pit mucous | UBE2N | 0.26 | 9.04E-66 |
| Pit mucous | Pit mucous | CD59 | -0.66 | 1.19E-65 |
| Pit mucous | Pit mucous | PSAP | -0.63 | 7.27E-65 |
| Pit mucous | Pit mucous | EIF4A1 | 0.3 | 8.32E-65 |
| Pit mucous | Pit mucous | HPGD | -0.84 | 3.26E-64 |
| Pit mucous | Pit mucous | MT-ND5 | 0.42 | 5.96E-64 |
| Pit mucous | Pit mucous | POMP | 0.34 | 6.87E-64 |
| Pit mucous | Pit mucous | SRP9 | 0.35 | 1.15E-63 |
| Pit mucous | Pit mucous | PYURF | 0.28 | 1.77E-63 |
| Pit mucous | Pit mucous | SLIRP | 0.36 | 2.01E-63 |
| Pit mucous | Pit mucous | KRT7 | 0.25 | 4.65E-63 |
| Pit mucous | Pit mucous | PDCD6 | 0.29 | 6.81E-63 |
| Pit mucous | Pit mucous | APRT | 0.31 | 8.77E-63 |
| Pit mucous | Pit mucous | RAB5IF | 0.31 | 1.03E-62 |
| Pit mucous | Pit mucous | ZFP36 | -1.07 | 1.56E-62 |
| Pit mucous | Pit mucous | NDUFA6 | 0.3 | 1.58E-62 |
| Pit mucous | Pit mucous | EI24 | 0.29 | 2.04E-62 |
| Pit mucous | Pit mucous | RTRAF | 0.28 | 2.57E-62 |
| Pit mucous | Pit mucous | NDUFA10 | 0.27 | 3.68E-62 |
| Pit mucous | Pit mucous | TMSB10 | -0.37 | 4.29E-62 |
| Pit mucous | Pit mucous | NDUFC2 | 0.32 | 8.33E-62 |
| Pit mucous | Pit mucous | VSIG1 | -0.66 | 1.82E-61 |
| Pit mucous | Pit mucous | EIF3L | 0.31 | 2.44E-61 |
| Pit mucous | Pit mucous | TPM3 | 0.27 | 7.54E-61 |
| Pit mucous | Pit mucous | HSPA6 | 0.29 | 1.03E-60 |
| Pit mucous | Pit mucous | PSMB8 | 0.25 | 2.25E-60 |
| Pit mucous | Pit mucous | PCBD1 | 0.25 | 5.14E-60 |
| Pit mucous | Pit mucous | CYCS | 0.31 | 1.17E-59 |
| Pit mucous | Pit mucous | ATP5F1E | -0.36 | 1.41E-59 |
| Pit mucous | Pit mucous | TOMM22 | 0.26 | 1.82E-59 |
| Pit mucous | Pit mucous | TESC | -0.73 | 2.21E-59 |
| Pit mucous | Pit mucous | CHCHD2 | 0.28 | 3.65E-59 |
| Pit mucous | Pit mucous | PSMA1 | 0.29 | 3.85E-59 |
| Pit mucous | Pit mucous | MT-ND1 | 0.4 | 4.12E-59 |
| Pit mucous | Pit mucous | SRSF3 | 0.31 | 6.85E-59 |
| Pit mucous | Pit mucous | GTF3C6 | 0.25 | 4.12E-58 |
| Pit mucous | Pit mucous | UGCG | -0.61 | 4.24E-58 |
| Pit mucous | Pit mucous | MT-CO3 | 0.34 | 4.25E-58 |
| Pit mucous | Pit mucous | EIF6 | 0.26 | 5.37E-58 |
| Pit mucous | Pit mucous | ATP5MD | 0.35 | 1.23E-57 |
| Pit mucous | Pit mucous | EIF3J | 0.28 | 1.43E-57 |
| Pit mucous | Pit mucous | RPL26 | 0.28 | 5.38E-57 |
| Pit mucous | Pit mucous | PLAC8 | -0.8 | 1.61E-56 |
| Pit mucous | Pit mucous | RPL3 | 0.26 | 8.04E-56 |
| Pit mucous | Pit mucous | VIM | -1.28 | 2.31E-55 |
| Pit mucous | Pit mucous | S100A6 | -0.54 | 2.59E-55 |
| Pit mucous | Pit mucous | ANPEP | -0.29 | 3.60E-55 |
| Pit mucous | Pit mucous | ATP5PO | 0.31 | 4.69E-55 |
| Pit mucous | Pit mucous | SOD1 | 0.27 | 3.30E-54 |
| Pit mucous | Pit mucous | SLC25A3 | 0.29 | 6.84E-54 |
| Pit mucous | Pit mucous | PSMB6 | 0.25 | 7.90E-53 |
| Pit mucous | Pit mucous | ATP5IF1 | 0.35 | 1.61E-52 |
| Pit mucous | Pit mucous | U2AF1 | 0.25 | 1.69E-52 |
| Pit mucous | Pit mucous | OCIAD2 | 0.32 | 2.30E-52 |
| Pit mucous | Pit mucous | PRDX5 | -0.43 | 3.76E-51 |
| Pit mucous | Pit mucous | DBI | 0.34 | 4.37E-51 |
| Pit mucous | Pit mucous | RPS7 | 0.29 | 5.60E-51 |
| Pit mucous | Pit mucous | PARK7 | 0.27 | 1.11E-50 |
| Pit mucous | Pit mucous | GDI2 | 0.32 | 1.17E-50 |
| Pit mucous | Pit mucous | EIF3E | 0.38 | 1.43E-50 |
| Pit mucous | Pit mucous | NDUFB9 | 0.26 | 2.72E-50 |
| Pit mucous | Pit mucous | MRPL57 | 0.26 | 2.33E-49 |
| Pit mucous | Pit mucous | SMIM14 | -0.57 | 2.51E-49 |
| Pit mucous | Pit mucous | DDT | 0.28 | 4.42E-49 |
| Pit mucous | Pit mucous | SSR4 | -0.73 | 1.75E-48 |
| Pit mucous | Pit mucous | SLPI | -0.71 | 3.38E-48 |
| Pit mucous | Pit mucous | RPSA | 0.26 | 4.49E-47 |
| Pit mucous | Pit mucous | ALDH3A1 | -0.73 | 5.82E-47 |
| Pit mucous | Pit mucous | YPEL5 | -0.67 | 6.63E-47 |
| Pit mucous | Pit mucous | AHNAK | -0.58 | 9.75E-47 |
| Pit mucous | Pit mucous | SLC25A6 | 0.26 | 1.20E-46 |
| Pit mucous | Pit mucous | RPL34 | 0.29 | 3.68E-46 |
| Pit mucous | Pit mucous | EIF3K | 0.25 | 6.07E-46 |
| Pit mucous | Pit mucous | MZT2A | 0.26 | 9.54E-46 |
| Pit mucous | Pit mucous | PDIA6 | 0.27 | 9.64E-46 |
| Pit mucous | Pit mucous | GLUL | -0.67 | 5.90E-45 |
| Pit mucous | Pit mucous | MT-ND6 | 0.29 | 1.20E-44 |
| Pit mucous | Pit mucous | YBX1 | 0.27 | 2.10E-44 |
| Pit mucous | Pit mucous | LGALS1 | -1.29 | 5.28E-44 |
| Pit mucous | Pit mucous | BCAS1 | -0.54 | 1.65E-43 |
| Pit mucous | Pit mucous | RPS15 | 0.25 | 4.85E-43 |
| Pit mucous | Pit mucous | FABP1 | -1.05 | 2.35E-42 |
| Pit mucous | Pit mucous | MUC6 | -1.47 | 2.94E-42 |
| Pit mucous | Pit mucous | NDUFB10 | 0.25 | 6.25E-42 |
| Pit mucous | Pit mucous | SPINK1 | -0.88 | 2.11E-41 |
| Pit mucous | Pit mucous | ANXA10 | -0.58 | 3.76E-41 |
| Pit mucous | Pit mucous | IER3 | -0.66 | 1.27E-40 |
| Pit mucous | Pit mucous | RPL36AL | -0.32 | 2.48E-40 |
| Pit mucous | Pit mucous | NDUFS6 | 0.25 | 1.21E-39 |
| Pit mucous | Pit mucous | SMIM6 | -0.51 | 2.62E-39 |
| Pit mucous | Pit mucous | LAMB3 | -0.63 | 6.49E-39 |
| Pit mucous | Pit mucous | SRGN | -1.04 | 1.26E-38 |
| Pit mucous | Pit mucous | TSC22D3 | -1.04 | 1.30E-38 |
| Pit mucous | Pit mucous | HES1 | 0.34 | 3.46E-37 |
| Pit mucous | Pit mucous | RFLNA | -0.46 | 5.31E-37 |
| Pit mucous | Pit mucous | SOCS3 | -0.76 | 7.39E-37 |
| Pit mucous | Pit mucous | SCGB2A1 | -0.59 | 8.91E-37 |
| Pit mucous | Pit mucous | EGR1 | -0.79 | 1.85E-36 |
| Pit mucous | Pit mucous | AC020656.1 | -1.28 | 1.89E-36 |
| Pit mucous | Pit mucous | SEC61G | -0.4 | 2.24E-36 |
| Pit mucous | Pit mucous | MCL1 | -0.48 | 4.06E-36 |
| Pit mucous | Pit mucous | TAGLN2 | -0.53 | 3.37E-35 |
| Pit mucous | Pit mucous | IFI6 | -0.47 | 4.79E-35 |
| Pit mucous | Pit mucous | MIDN | -0.51 | 9.14E-35 |
| Pit mucous | Pit mucous | EZR | -0.43 | 3.01E-34 |
| Pit mucous | Pit mucous | RARRES3 | -0.44 | 1.22E-33 |
| Pit mucous | Pit mucous | PLA2G10 | -0.49 | 3.05E-33 |
| Pit mucous | Pit mucous | TUBA1A | -0.72 | 4.92E-33 |
| Pit mucous | Pit mucous | CFD | -0.27 | 9.35E-33 |
| Pit mucous | Pit mucous | ATF3 | -0.62 | 1.04E-32 |
| Pit mucous | Pit mucous | ITPKC | -0.48 | 1.97E-32 |
| Pit mucous | Pit mucous | UBL3 | -0.43 | 2.32E-32 |
| Pit mucous | Pit mucous | HLA-E | -0.56 | 1.21E-31 |
| Pit mucous | Pit mucous | PCSK1N | -1.04 | 1.95E-31 |
| Pit mucous | Pit mucous | XIST | -0.37 | 2.68E-31 |
| Pit mucous | Pit mucous | UBC | -0.36 | 7.66E-31 |
| Pit mucous | Pit mucous | LINC01133 | -0.6 | 9.21E-31 |
| Pit mucous | Pit mucous | HRASLS2 | -0.5 | 3.62E-30 |
| Pit mucous | Pit mucous | PSAPL1 | -0.51 | 7.08E-30 |
| Pit mucous | Pit mucous | ITM2B | -0.46 | 3.48E-29 |
| Pit mucous | Pit mucous | CDKN1A | -0.53 | 7.70E-29 |
| Pit mucous | Pit mucous | VAMP8 | -0.49 | 8.88E-29 |
| Pit mucous | Pit mucous | TSPAN1 | -0.49 | 4.14E-28 |
| Pit mucous | Pit mucous | FOXQ1 | -0.59 | 9.74E-28 |
| Pit mucous | Pit mucous | TCEAL9 | -0.48 | 1.83E-27 |
| Pit mucous | Pit mucous | CTSD | -0.49 | 2.97E-26 |
| Pit mucous | Pit mucous | RGS1 | -0.91 | 4.34E-26 |
| Pit mucous | Pit mucous | TRNP1 | -0.33 | 9.70E-26 |
| Pit mucous | Pit mucous | AKR1C2 | -0.45 | 1.13E-25 |
| Pit mucous | Pit mucous | DDX5 | -0.33 | 1.31E-25 |
| Pit mucous | Pit mucous | RPS10 | -0.3 | 2.05E-25 |
| Pit mucous | Pit mucous | SELENOM | -0.4 | 2.68E-25 |
| Pit mucous | Pit mucous | MIF | 0.27 | 6.47E-25 |
| Pit mucous | Pit mucous | SLC7A8 | -0.41 | 6.71E-25 |
| Pit mucous | Pit mucous | ATF4 | -0.4 | 1.68E-24 |
| Pit mucous | Pit mucous | CSRNP1 | -0.43 | 4.02E-24 |
| Pit mucous | Pit mucous | ATP6V1G1 | -0.34 | 6.93E-24 |
| Pit mucous | Pit mucous | MIR22HG | -0.4 | 1.62E-23 |
| Pit mucous | Pit mucous | RAB27A | -0.36 | 3.25E-23 |
| Pit mucous | Pit mucous | BEX3 | -0.39 | 4.75E-23 |
| Pit mucous | Pit mucous | GABARAPL1 | -0.29 | 8.17E-23 |
| Pit mucous | Pit mucous | ADAM28 | -0.26 | 1.01E-22 |
| Pit mucous | Pit mucous | PFDN5 | -0.25 | 1.28E-22 |
| Pit mucous | Pit mucous | SERTAD1 | -0.44 | 1.41E-22 |
| Pit mucous | Pit mucous | CEBPB | -0.4 | 1.58E-22 |
| Pit mucous | Pit mucous | ELL2 | -0.32 | 3.20E-22 |
| Pit mucous | Pit mucous | KRT10 | -0.39 | 1.41E-21 |
| Pit mucous | Pit mucous | CRIP2 | -0.46 | 1.45E-21 |
| Pit mucous | Pit mucous | OST4 | -0.29 | 1.47E-21 |
| Pit mucous | Pit mucous | UBL5 | -0.27 | 1.77E-21 |
| Pit mucous | Pit mucous | MAFF | -0.42 | 2.81E-21 |
| Pit mucous | Pit mucous | IRF1 | -0.51 | 4.50E-21 |
| Pit mucous | Pit mucous | RHOB | -0.5 | 5.11E-21 |
| Pit mucous | Pit mucous | TACSTD2 | -0.48 | 5.76E-21 |
| Pit mucous | Pit mucous | SLC9A3R2 | -0.43 | 6.78E-21 |
| Pit mucous | Pit mucous | YOD1 | -0.42 | 1.24E-20 |
| Pit mucous | Pit mucous | KLF4 | -0.47 | 1.94E-20 |
| Pit mucous | Pit mucous | LMO4 | -0.42 | 2.16E-20 |
| Pit mucous | Pit mucous | PNRC1 | -0.44 | 2.55E-20 |
| Pit mucous | Pit mucous | RUNX1 | -0.32 | 2.81E-20 |
| Pit mucous | Pit mucous | BRI3 | -0.39 | 3.09E-20 |
| Pit mucous | Pit mucous | HSPB1 | -0.36 | 3.60E-20 |
| Pit mucous | Pit mucous | ERBIN | -0.3 | 4.16E-20 |
| Pit mucous | Pit mucous | HLA-C | -0.43 | 6.13E-20 |
| Pit mucous | Pit mucous | FAM102A | -0.43 | 7.54E-20 |
| Pit mucous | Pit mucous | RAP2B | -0.44 | 7.97E-20 |
| Pit mucous | Pit mucous | PRSS23 | -0.43 | 1.04E-19 |
| Pit mucous | Pit mucous | IDS | -0.36 | 3.01E-19 |
| Pit mucous | Pit mucous | LMO7 | -0.49 | 6.26E-19 |
| Pit mucous | Pit mucous | SLC9A1 | -0.34 | 1.19E-18 |
| Pit mucous | Pit mucous | RAB27B | -0.41 | 2.35E-18 |
| Pit mucous | Pit mucous | POLD4 | -0.42 | 2.45E-18 |
| Pit mucous | Pit mucous | PRR4 | -0.72 | 2.79E-18 |
| Pit mucous | Pit mucous | PDE4C | -0.51 | 9.44E-18 |
| Pit mucous | Pit mucous | GADD45B | -0.77 | 1.00E-17 |
| Pit mucous | Pit mucous | MLPH | -0.38 | 1.07E-17 |
| Pit mucous | Pit mucous | TMEM59 | -0.32 | 1.36E-17 |
| Pit mucous | Pit mucous | VAMP5 | -0.3 | 1.53E-17 |
| Pit mucous | Pit mucous | TMPRSS15 | -0.45 | 1.70E-17 |
| Pit mucous | Pit mucous | SDC4 | -0.46 | 2.06E-17 |
| Pit mucous | Pit mucous | CACFD1 | -0.28 | 2.40E-17 |
| Pit mucous | Pit mucous | EIF4A2 | -0.3 | 4.42E-17 |
| Pit mucous | Pit mucous | GALNT6 | -0.37 | 4.90E-17 |
| Pit mucous | Pit mucous | FAM107B | -0.38 | 5.20E-17 |
| Pit mucous | Pit mucous | VAMP2 | -0.43 | 7.28E-17 |
| Pit mucous | Pit mucous | ETNK1 | -0.36 | 8.67E-17 |
| Pit mucous | Pit mucous | AAMDC | -0.39 | 9.16E-17 |
| Pit mucous | Pit mucous | BHLHE40 | -0.35 | 9.94E-17 |
| Pit mucous | Pit mucous | BTG2 | -0.5 | 1.56E-16 |
| Pit mucous | Pit mucous | ALDH1A1 | -0.4 | 1.99E-16 |
| Pit mucous | Pit mucous | NPDC1 | -0.32 | 3.05E-16 |
| Pit mucous | Pit mucous | JUNB | -0.75 | 3.09E-16 |
| Pit mucous | Pit mucous | EFHD2 | -0.45 | 3.17E-16 |
| Pit mucous | Pit mucous | RASSF6 | -0.29 | 4.92E-16 |
| Pit mucous | Pit mucous | SYTL2 | -0.36 | 5.44E-16 |
| Pit mucous | Pit mucous | MIA | -0.49 | 6.89E-16 |
| Pit mucous | Pit mucous | ERO1B | -0.35 | 7.27E-16 |
| Pit mucous | Pit mucous | S100A11 | -0.29 | 9.44E-16 |
| Pit mucous | Pit mucous | CCNL1 | -0.38 | 9.46E-16 |
| Pit mucous | Pit mucous | PHGR1 | -0.8 | 1.15E-15 |
| Pit mucous | Pit mucous | SGMS2 | -0.39 | 1.22E-15 |
| Pit mucous | Pit mucous | CHP1 | -0.39 | 1.55E-15 |
| Pit mucous | Pit mucous | STARD10 | -0.4 | 3.95E-15 |
| Pit mucous | Pit mucous | B4GALNT3 | -0.28 | 4.40E-15 |
| Pit mucous | Pit mucous | BPIFB1 | -0.75 | 5.28E-15 |
| Pit mucous | Pit mucous | ACOX1 | -0.28 | 5.52E-15 |
| Pit mucous | Pit mucous | IFNGR2 | -0.33 | 6.12E-15 |
| Pit mucous | Pit mucous | VILL | -0.41 | 7.37E-15 |
| Pit mucous | Pit mucous | NDRG1 | -0.35 | 8.13E-15 |
| Pit mucous | Pit mucous | PDK4 | -0.3 | 1.18E-14 |
| Pit mucous | Pit mucous | ABHD2 | -0.39 | 2.19E-14 |
| Pit mucous | Pit mucous | PAM | -0.3 | 3.67E-14 |
| Pit mucous | Pit mucous | AKR1C1 | -0.52 | 4.42E-14 |
| Pit mucous | Pit mucous | AKAP13 | -0.36 | 6.40E-14 |
| Pit mucous | Pit mucous | RIOK3 | -0.37 | 7.94E-14 |
| Pit mucous | Pit mucous | SLC7A11 | -0.29 | 8.76E-14 |
| Pit mucous | Pit mucous | B3GNT5 | -0.37 | 1.01E-13 |
| Pit mucous | Pit mucous | CCNI | -0.34 | 1.28E-13 |
| Pit mucous | Pit mucous | BIRC3 | -0.37 | 1.30E-13 |
| Pit mucous | Pit mucous | METTL7A | -0.3 | 1.65E-13 |
| Pit mucous | Pit mucous | SLC44A4 | -0.38 | 1.66E-13 |
| Pit mucous | Pit mucous | ITGAV | -0.25 | 3.48E-13 |
| Pit mucous | Pit mucous | FOS | -0.73 | 4.39E-13 |
| Pit mucous | Pit mucous | IGFBP4 | -0.4 | 1.07E-12 |
| Pit mucous | Pit mucous | CYP2S1 | -0.38 | 1.27E-12 |
| Pit mucous | Pit mucous | C12orf75 | -0.43 | 1.62E-12 |
| Pit mucous | Pit mucous | TSC22D2 | -0.31 | 1.69E-12 |
| Pit mucous | Pit mucous | SDCBP | -0.39 | 2.22E-12 |
| Pit mucous | Pit mucous | KDM6B | -0.33 | 3.36E-12 |
| Pit mucous | Pit mucous | SIK1 | -0.44 | 3.80E-12 |
| Pit mucous | Pit mucous | DDX17 | -0.37 | 6.70E-12 |
| Pit mucous | Pit mucous | SLC38A2 | -0.35 | 7.17E-12 |
| Pit mucous | Pit mucous | NAMPT | -0.31 | 1.19E-11 |
| Pit mucous | Pit mucous | ARF6 | -0.38 | 1.38E-11 |
| Pit mucous | Pit mucous | PNPLA2 | -0.26 | 1.77E-11 |
| Pit mucous | Pit mucous | C11orf86 | -0.47 | 2.15E-11 |
| Pit mucous | Pit mucous | SULT1C2 | -0.35 | 2.16E-11 |
| Pit mucous | Pit mucous | IGLL5 | -2.67 | 2.30E-11 |
| Pit mucous | Pit mucous | MS4A8 | -0.32 | 2.49E-11 |
| Pit mucous | Pit mucous | OASL | -0.3 | 3.06E-11 |
| Pit mucous | Pit mucous | CYTOR | -0.39 | 3.20E-11 |
| Pit mucous | Pit mucous | ABLIM1 | -0.38 | 3.34E-11 |
| Pit mucous | Pit mucous | OTUD1 | -0.29 | 3.65E-11 |
| Pit mucous | Pit mucous | RHOF | -0.33 | 3.82E-11 |
| Pit mucous | Pit mucous | SH3BGRL3 | -0.25 | 4.75E-11 |
| Pit mucous | Pit mucous | SDCBP2 | -0.45 | 5.12E-11 |
| Pit mucous | Pit mucous | SGSM3 | -0.32 | 6.09E-11 |
| Pit mucous | Pit mucous | REG3A | -0.47 | 6.92E-11 |
| Pit mucous | Pit mucous | IER2 | -0.36 | 7.00E-11 |
| Pit mucous | Pit mucous | PRKAR1A | -0.27 | 8.20E-11 |
| Pit mucous | Pit mucous | EPS8L1 | -0.29 | 8.75E-11 |
| Pit mucous | Pit mucous | CDHR5 | -0.39 | 9.51E-11 |
| Pit mucous | Pit mucous | IFI27L2 | -0.31 | 1.45E-10 |
| Pit mucous | Pit mucous | AC023090.1 | -0.3 | 1.52E-10 |
| Pit mucous | Pit mucous | ATP2A3 | -0.27 | 1.53E-10 |
| Pit mucous | Pit mucous | TIPARP | -0.28 | 1.67E-10 |
| Pit mucous | Pit mucous | ZG16B | -0.36 | 1.98E-10 |
| Pit mucous | Pit mucous | CAPN8 | -0.46 | 2.13E-10 |
| Pit mucous | Pit mucous | FKBP11 | -0.26 | 3.21E-10 |
| Pit mucous | Pit mucous | SERP1 | -0.26 | 4.66E-10 |
| Pit mucous | Pit mucous | PLXNB2 | -0.32 | 4.76E-10 |
| Pit mucous | Pit mucous | TNFAIP3 | -0.43 | 5.86E-10 |
| Pit mucous | Pit mucous | RABAC1 | -0.28 | 6.08E-10 |
| Pit mucous | Pit mucous | JUN | -0.51 | 6.20E-10 |
| Pit mucous | Pit mucous | NEDD4L | -0.3 | 6.71E-10 |
| Pit mucous | Pit mucous | RAC1 | -0.33 | 6.81E-10 |
| Pit mucous | Pit mucous | EMP3 | -0.42 | 8.74E-10 |
| Pit mucous | Pit mucous | SLC39A11 | -0.27 | 1.06E-09 |
| Pit mucous | Pit mucous | RND1 | -0.31 | 1.08E-09 |
| Pit mucous | Pit mucous | FKBP2 | -0.29 | 1.38E-09 |
| Pit mucous | Pit mucous | RBP2 | -0.91 | 1.46E-09 |
| Pit mucous | Pit mucous | FOXA3 | -0.34 | 1.67E-09 |
| Pit mucous | Pit mucous | PELI1 | -0.26 | 1.76E-09 |
| Pit mucous | Pit mucous | HMGCS1 | -0.36 | 1.91E-09 |
| Pit mucous | Pit mucous | FAM3B | -0.29 | 1.91E-09 |
| Pit mucous | Pit mucous | MXD1 | -0.33 | 2.30E-09 |
| Pit mucous | Pit mucous | GNAQ | -0.28 | 2.38E-09 |
| Pit mucous | Pit mucous | NBEAL1 | -0.29 | 2.81E-09 |
| Pit mucous | Pit mucous | PARD6B | -0.38 | 3.39E-09 |
| Pit mucous | Pit mucous | BCAR1 | -0.26 | 3.46E-09 |
| Pit mucous | Pit mucous | UBE2H | -0.26 | 6.09E-09 |
| Pit mucous | Pit mucous | GPAT3 | -0.26 | 6.27E-09 |
| Pit mucous | Pit mucous | RASEF | -0.38 | 6.39E-09 |
| Pit mucous | Pit mucous | GABARAP | -0.3 | 1.00E-08 |
| Pit mucous | Pit mucous | TIMP1 | -0.53 | 1.00E-08 |
| Pit mucous | Pit mucous | SELENOP | -0.45 | 1.02E-08 |
| Pit mucous | Pit mucous | F2RL1 | -0.36 | 1.05E-08 |
| Pit mucous | Pit mucous | SH3BGRL2 | -0.28 | 1.06E-08 |
| Pit mucous | Pit mucous | MAL2 | -0.38 | 1.13E-08 |
| Pit mucous | Pit mucous | LASP1 | -0.34 | 1.18E-08 |
| Pit mucous | Pit mucous | B4GALT1 | -0.31 | 1.48E-08 |
| Pit mucous | Pit mucous | PPP1R15A | -0.26 | 3.21E-08 |
| Pit mucous | Pit mucous | AFF4 | -0.26 | 3.91E-08 |
| Pit mucous | Pit mucous | HLA-DPB1 | -0.56 | 3.98E-08 |
| Pit mucous | Pit mucous | JAG1 | -0.27 | 5.54E-08 |
| Pit mucous | Pit mucous | S100A4 | -0.87 | 5.89E-08 |
| Pit mucous | Pit mucous | COX17 | -0.28 | 7.04E-08 |
| Pit mucous | Pit mucous | EPHA2 | -0.34 | 8.62E-08 |
| Pit mucous | Pit mucous | CCDC107 | -0.28 | 1.16E-07 |
| Pit mucous | Pit mucous | IFITM1 | -0.31 | 1.88E-07 |
| Pit mucous | Pit mucous | TM4SF1 | -0.34 | 2.91E-07 |
| Pit mucous | Pit mucous | VSIR | -0.25 | 3.28E-07 |
| Pit mucous | Pit mucous | SSBP3 | -0.25 | 3.39E-07 |
| Pit mucous | Pit mucous | IL1RN | -0.39 | 4.27E-07 |
| Pit mucous | Pit mucous | HERPUD1 | -0.53 | 4.60E-07 |
| Pit mucous | Pit mucous | SELENOK | -0.3 | 5.10E-07 |
| Pit mucous | Pit mucous | NUPR1 | -0.26 | 1.05E-06 |
| Pit mucous | Pit mucous | QSOX1 | -0.31 | 1.18E-06 |
| Pit mucous | Pit mucous | MAP2K3 | -0.29 | 1.73E-06 |
| Pit mucous | Pit mucous | PIM1 | -0.3 | 1.97E-06 |
| Pit mucous | Pit mucous | TAX1BP3 | -0.31 | 2.34E-06 |
| Pit mucous | Pit mucous | FOSL2 | -0.28 | 2.36E-06 |
| Pit mucous | Pit mucous | DDX3X | -0.32 | 2.41E-06 |
| Pit mucous | Pit mucous | EPN1 | -0.28 | 2.50E-06 |
| Pit mucous | Pit mucous | CLU | -0.56 | 2.58E-06 |
| Pit mucous | Pit mucous | CLTB | -0.33 | 2.80E-06 |
| Pit mucous | Pit mucous | ANKRD28 | -0.41 | 2.82E-06 |
| Pit mucous | Pit mucous | RAB11FIP1 | -0.33 | 3.75E-06 |
| Pit mucous | Pit mucous | TENT5A | -0.37 | 4.20E-06 |
| Pit mucous | Pit mucous | LDLR | -0.27 | 4.41E-06 |
| Pit mucous | Pit mucous | NEDD9 | -0.31 | 4.49E-06 |
| Pit mucous | Pit mucous | NQO1 | -0.28 | 5.33E-06 |
| Pit mucous | Pit mucous | CXCL2 | -0.4 | 5.72E-06 |
| Pit mucous | Pit mucous | PTP4A1 | -0.29 | 7.99E-06 |
| CD8T | CD8Tcm | CCL5 | 3.74 | 0 |
| CD8T | CD8Tcm | CD52 | 2.96 | 0 |
| CD8T | CD8Tcm | KLRB1 | 2.76 | 0 |
| CD8T | CD8Tcm | S100A4 | 2.75 | 0 |
| CD8T | CD8Tcm | CD7 | 2.56 | 0 |
| CD8T | CD8Tcm | CD3D | 2.48 | 0 |
| CD8T | CD8Tcm | CREM | 2.48 | 0 |
| CD8T | CD8Tcm | CXCR4 | 2.4 | 0 |
| CD8T | CD8Tcm | GZMA | 2.35 | 0 |
| CD8T | CD8Tcm | NKG7 | 2.32 | 0 |
| CD8T | CD8Tcm | HCST | 2.29 | 0 |
| CD8T | CD8Tcm | SRGN | 2.25 | 0 |
| CD8T | CD8Tcm | LINC01871 | 2.15 | 0 |
| CD8T | CD8Tcm | CD3E | 2.1 | 0 |
| CD8T | CD8Tcm | ARHGDIB | 2.07 | 0 |
| CD8T | CD8Tcm | GZMB | 2.04 | 0 |
| CD8T | CD8Tcm | CD69 | 2 | 0 |
| CD8T | CD8Tcm | TSC22D3 | 2 | 0 |
| CD8T | CD8Tcm | GNLY | 1.93 | 0 |
| CD8T | CD8Tcm | BTG1 | 1.92 | 0 |
| CD8T | CD8Tcm | RGS1 | 1.9 | 0 |
| CD8T | CD8Tcm | PTPRCAP | 1.88 | 0 |
| CD8T | CD8Tcm | IL32 | 1.83 | 0 |
| CD8T | CD8Tcm | IFITM2 | 1.83 | 0 |
| CD8T | CD8Tcm | XCL2 | 1.83 | 0 |
| CD8T | CD8Tcm | CD2 | 1.81 | 0 |
| CD8T | CD8Tcm | CST7 | 1.79 | 0 |
| CD8T | CD8Tcm | CORO1A | 1.77 | 0 |
| CD8T | CD8Tcm | PTPRC | 1.74 | 0 |
| CD8T | CD8Tcm | SAMSN1 | 1.73 | 0 |
| CD8T | CD8Tcm | RGCC | 1.72 | 0 |
| CD8T | CD8Tcm | ALOX5AP | 1.68 | 0 |
| CD8T | CD8Tcm | RUNX3 | 1.59 | 0 |
| CD8T | CD8Tcm | LEPROTL1 | 1.59 | 0 |
| CD8T | CD8Tcm | LAPTM5 | 1.58 | 0 |
| CD8T | CD8Tcm | HOPX | 1.54 | 0 |
| CD8T | CD8Tcm | CD8A | 1.52 | 0 |
| CD8T | CD8Tcm | LTB | 1.51 | 0 |
| CD8T | CD8Tcm | ANXA1 | 1.49 | 0 |
| CD8T | CD8Tcm | CD3G | 1.48 | 0 |
| CD8T | CD8Tcm | CLEC2B | 1.48 | 0 |
| CD8T | CD8Tcm | NR4A2 | 1.45 | 0 |
| CD8T | CD8Tcm | VIM | 1.45 | 0 |
| CD8T | CD8Tcm | RAC2 | 1.38 | 0 |
| CD8T | CD8Tcm | EVL | 1.38 | 0 |
| CD8T | CD8Tcm | CD96 | 1.36 | 0 |
| CD8T | CD8Tcm | CD37 | 1.34 | 0 |
| CD8T | CD8Tcm | STK17B | 1.34 | 0 |
| CD8T | CD8Tcm | DUSP2 | 1.32 | 0 |
| CD8T | CD8Tcm | GZMH | 1.32 | 0 |
| CD8T | CD8Tcm | IFNG | 1.31 | 0 |
| CD8T | CD8Tcm | IL7R | 1.29 | 0 |
| CD8T | CD8Tcm | GZMM | 1.28 | 0 |
| CD8T | CD8Tcm | LSP1 | 1.28 | 0 |
| CD8T | CD8Tcm | CTSW | 1.28 | 0 |
| CD8T | CD8Tcm | LCK | 1.27 | 0 |
| CD8T | CD8Tcm | XCL1 | 1.25 | 0 |
| CD8T | CD8Tcm | RORA | 1.25 | 0 |
| CD8T | CD8Tcm | TMSB4X | 1.24 | 0 |
| CD8T | CD8Tcm | CD8B | 1.23 | 0 |
| CD8T | CD8Tcm | GPR65 | 1.22 | 0 |
| CD8T | CD8Tcm | B2M | 1.22 | 0 |
| CD8T | CD8Tcm | ACAP1 | 1.21 | 0 |
| CD8T | CD8Tcm | KLRC1 | 1.21 | 0 |
| CD8T | CD8Tcm | CD53 | 1.21 | 0 |
| CD8T | CD8Tcm | GPR183 | 1.21 | 0 |
| CD8T | CD8Tcm | STAT4 | 1.18 | 0 |
| CD8T | CD8Tcm | GPSM3 | 1.17 | 0 |
| CD8T | CD8Tcm | LDLRAD4 | 1.16 | 0 |
| CD8T | CD8Tcm | CD48 | 1.15 | 0 |
| CD8T | CD8Tcm | GMFG | 1.13 | 0 |
| CD8T | CD8Tcm | IFITM1 | 1.12 | 0 |
| CD8T | CD8Tcm | AC114760.2 | 1.12 | 0 |
| CD8T | CD8Tcm | FYN | 1.11 | 0 |
| CD8T | CD8Tcm | HLA-B | 1.1 | 0 |
| CD8T | CD8Tcm | ARL4C | 1.1 | 0 |
| CD8T | CD8Tcm | HLA-C | 1.09 | 0 |
| CD8T | CD8Tcm | FYB1 | 1.08 | 0 |
| CD8T | CD8Tcm | WIPF1 | 1.07 | 0 |
| CD8T | CD8Tcm | GNG2 | 1.07 | 0 |
| CD8T | CD8Tcm | LCP1 | 1.07 | 0 |
| CD8T | CD8Tcm | ITM2A | 1.05 | 0 |
| CD8T | CD8Tcm | SYTL3 | 1.05 | 0 |
| CD8T | CD8Tcm | LAT | 0.98 | 0 |
| CD8T | CD8Tcm | GYPC | 0.98 | 0 |
| CD8T | CD8Tcm | SH3BGRL3 | 0.98 | 0 |
| CD8T | CD8Tcm | LGALS1 | 0.96 | 0 |
| CD8T | CD8Tcm | RPS27 | 0.96 | 0 |
| CD8T | CD8Tcm | FNBP1 | 0.95 | 0 |
| CD8T | CD8Tcm | SPOCK2 | 0.95 | 0 |
| CD8T | CD8Tcm | RHOH | 0.94 | 0 |
| CD8T | CD8Tcm | CELF2 | 0.94 | 0 |
| CD8T | CD8Tcm | KLRD1 | 0.89 | 0 |
| CD8T | CD8Tcm | PTPN7 | 0.88 | 0 |
| CD8T | CD8Tcm | LIMD2 | 0.84 | 0 |
| CD8T | CD8Tcm | SH2D2A | 0.84 | 0 |
| CD8T | CD8Tcm | DOK2 | 0.83 | 0 |
| CD8T | CD8Tcm | PRF1 | 0.81 | 0 |
| CD8T | CD8Tcm | CXCR6 | 0.8 | 0 |
| CD8T | CD8Tcm | TBC1D10C | 0.79 | 0 |
| CD8T | CD8Tcm | PTPN22 | 0.78 | 0 |
| CD8T | CD8Tcm | RPS29 | 0.77 | 0 |
| CD8T | CD8Tcm | CD247 | 0.76 | 0 |
| CD8T | CD8Tcm | IL2RB | 0.75 | 0 |
| CD8T | CD8Tcm | JAML | 0.74 | 0 |
| CD8T | CD8Tcm | ICOS | 0.73 | 0 |
| CD8T | CD8Tcm | SLA | 0.72 | 0 |
| CD8T | CD8Tcm | RGL4 | 0.72 | 0 |
| CD8T | CD8Tcm | SLA2 | 0.71 | 0 |
| CD8T | CD8Tcm | CLEC2D | 0.71 | 0 |
| CD8T | CD8Tcm | RPLP1 | 0.7 | 0 |
| CD8T | CD8Tcm | ARHGAP9 | 0.69 | 0 |
| CD8T | CD8Tcm | MALAT1 | 0.69 | 0 |
| CD8T | CD8Tcm | ITGA4 | 0.69 | 0 |
| CD8T | CD8Tcm | ITGB2 | 0.66 | 0 |
| CD8T | CD8Tcm | MATK | 0.65 | 0 |
| CD8T | CD8Tcm | RPLP2 | 0.65 | 0 |
| CD8T | CD8Tcm | GATA3 | 0.64 | 0 |
| CD8T | CD8Tcm | PDCD1 | 0.63 | 0 |
| CD8T | CD8Tcm | GPR171 | 0.62 | 0 |
| CD8T | CD8Tcm | SIT1 | 0.62 | 0 |
| CD8T | CD8Tcm | LAG3 | 0.62 | 0 |
| CD8T | CD8Tcm | SPN | 0.56 | 0 |
| CD8T | CD8Tcm | CMTM3 | 0.54 | 0 |
| CD8T | CD8Tcm | SKAP1 | 0.53 | 0 |
| CD8T | CD8Tcm | MYO1G | 0.52 | 0 |
| CD8T | CD8Tcm | CD6 | 0.51 | 0 |
| CD8T | CD8Tcm | MT-CO3 | -1.02 | 0 |
| CD8T | CD8Tcm | GSTP1 | -1.09 | 0 |
| CD8T | CD8Tcm | SMIM22 | -1.4 | 0 |
| CD8T | CD8Tcm | C19orf33 | -1.45 | 0 |
| CD8T | CD8Tcm | CST3 | -1.45 | 0 |
| CD8T | CD8Tcm | FXYD3 | -1.47 | 0 |
| CD8T | CD8Tcm | CYSTM1 | -1.61 | 0 |
| CD8T | CD8Tcm | IFI27 | -1.62 | 0 |
| CD8T | CD8Tcm | SPINK1 | -1.78 | 0 |
| CD8T | CD8Tcm | AGR2 | -1.82 | 0 |
| CD8T | CD8Tcm | KRT18 | -1.84 | 0 |
| CD8T | CD8Tcm | KRT8 | -1.87 | 0 |
| CD8T | CD8Tcm | KRT19 | -1.88 | 0 |
| CD8T | CD8Tcm | TSPAN8 | -1.9 | 0 |
| CD8T | CD8Tcm | IKZF1 | 0.5 | 1.52E-304 |
| CD8T | CD8Tcm | EIF1 | 0.73 | 9.79E-297 |
| CD8T | CD8Tcm | ELF3 | -1.18 | 2.32E-289 |
| CD8T | CD8Tcm | APOBEC3G | 0.7 | 9.73E-288 |
| CD8T | CD8Tcm | CYTIP | 1.11 | 2.16E-280 |
| CD8T | CD8Tcm | LGALS4 | -1.6 | 6.99E-280 |
| CD8T | CD8Tcm | FMNL1 | 0.55 | 3.05E-277 |
| CD8T | CD8Tcm | RPS25 | 0.72 | 6.46E-277 |
| CD8T | CD8Tcm | RPS27A | 0.54 | 3.10E-272 |
| CD8T | CD8Tcm | SFN | -1.38 | 4.03E-272 |
| CD8T | CD8Tcm | STK4 | 1.1 | 5.98E-271 |
| CD8T | CD8Tcm | S100A14 | -1.52 | 1.28E-268 |
| CD8T | CD8Tcm | TNFAIP3 | 1.34 | 2.04E-267 |
| CD8T | CD8Tcm | RPS15A | 0.56 | 2.73E-267 |
| CD8T | CD8Tcm | AC004687.1 | 0.56 | 7.13E-267 |
| CD8T | CD8Tcm | TM4SF1 | -1.36 | 1.96E-263 |
| CD8T | CD8Tcm | ZNF331 | 1.11 | 7.11E-263 |
| CD8T | CD8Tcm | TGFB1 | 0.88 | 1.21E-260 |
| CD8T | CD8Tcm | EPCAM | -1.41 | 7.30E-258 |
| CD8T | CD8Tcm | CTSE | -1.3 | 3.19E-257 |
| CD8T | CD8Tcm | ADIRF | -1.57 | 3.15E-256 |
| CD8T | CD8Tcm | PIGR | -1.67 | 4.90E-256 |
| CD8T | CD8Tcm | RPL30 | 0.72 | 5.34E-256 |
| CD8T | CD8Tcm | PHLDA2 | -1.28 | 1.32E-255 |
| CD8T | CD8Tcm | S100P | -1.56 | 2.71E-255 |
| CD8T | CD8Tcm | HLA-A | 0.81 | 1.33E-254 |
| CD8T | CD8Tcm | NQO1 | -1.14 | 2.01E-253 |
| CD8T | CD8Tcm | MT-ND4 | -0.89 | 8.10E-248 |
| CD8T | CD8Tcm | CXCR3 | 0.67 | 2.08E-247 |
| CD8T | CD8Tcm | SPINT2 | -1.04 | 7.19E-246 |
| CD8T | CD8Tcm | ITGA1 | 0.72 | 1.59E-244 |
| CD8T | CD8Tcm | 1-Sep | 0.83 | 1.18E-243 |
| CD8T | CD8Tcm | EVI2A | 0.7 | 2.67E-241 |
| CD8T | CD8Tcm | NEAT1 | -0.91 | 9.31E-241 |
| CD8T | CD8Tcm | CLDN18 | -1.35 | 1.39E-240 |
| CD8T | CD8Tcm | MT-CO2 | -1.18 | 5.69E-240 |
| CD8T | CD8Tcm | 6-Sep | 0.77 | 1.36E-237 |
| CD8T | CD8Tcm | FAU | 0.59 | 2.39E-237 |
| CD8T | CD8Tcm | RPL21 | 0.54 | 6.41E-236 |
| CD8T | CD8Tcm | ID2 | 1.32 | 1.49E-235 |
| CD8T | CD8Tcm | GPX2 | -1.26 | 1.57E-235 |
| CD8T | CD8Tcm | IER3 | -1.22 | 1.62E-235 |
| CD8T | CD8Tcm | BIN2 | 0.55 | 1.39E-232 |
| CD8T | CD8Tcm | ANXA10 | -1.28 | 4.15E-232 |
| CD8T | CD8Tcm | SERPINB9 | 0.74 | 2.71E-231 |
| CD8T | CD8Tcm | CKLF | 1.31 | 1.18E-230 |
| CD8T | CD8Tcm | YPEL5 | 1.19 | 1.08E-229 |
| CD8T | CD8Tcm | S100A16 | -1.25 | 1.56E-229 |
| CD8T | CD8Tcm | RPL23A | 0.62 | 2.74E-229 |
| CD8T | CD8Tcm | RPL28 | 0.54 | 9.52E-228 |
| CD8T | CD8Tcm | PPP1R18 | 0.71 | 1.13E-227 |
| CD8T | CD8Tcm | TXN | -1.16 | 1.76E-227 |
| CD8T | CD8Tcm | IL2RG | 1.14 | 5.94E-226 |
| CD8T | CD8Tcm | GLIPR1 | 0.72 | 2.17E-224 |
| CD8T | CD8Tcm | LGALS3BP | -0.99 | 6.16E-224 |
| CD8T | CD8Tcm | EMP3 | 1.04 | 1.86E-222 |
| CD8T | CD8Tcm | PIK3R1 | 0.97 | 3.05E-222 |
| CD8T | CD8Tcm | LYZ | -1.71 | 8.37E-220 |
| CD8T | CD8Tcm | PDE4D | 0.97 | 2.20E-216 |
| CD8T | CD8Tcm | TSPYL2 | 1.04 | 2.81E-216 |
| CD8T | CD8Tcm | CA2 | -1.33 | 9.28E-215 |
| CD8T | CD8Tcm | MT-ATP6 | -1.01 | 2.49E-214 |
| CD8T | CD8Tcm | PHGR1 | -2.08 | 1.18E-212 |
| CD8T | CD8Tcm | MGST2 | -1.06 | 6.13E-211 |
| CD8T | CD8Tcm | TNFRSF1B | 0.77 | 1.66E-210 |
| CD8T | CD8Tcm | ANKRD28 | 0.99 | 1.67E-210 |
| CD8T | CD8Tcm | ARL14 | -1.19 | 8.58E-210 |
| CD8T | CD8Tcm | S100A6 | -1.03 | 1.35E-209 |
| CD8T | CD8Tcm | SARAF | 1.23 | 3.63E-207 |
| CD8T | CD8Tcm | RPL10 | 0.43 | 3.55E-206 |
| CD8T | CD8Tcm | AGR3 | -1.3 | 6.38E-205 |
| CD8T | CD8Tcm | ZFP36L2 | 1.49 | 1.06E-203 |
| CD8T | CD8Tcm | SLC2A3 | 0.76 | 1.07E-201 |
| CD8T | CD8Tcm | HCLS1 | 0.6 | 2.07E-200 |
| CD8T | CD8Tcm | PIP4K2A | 0.76 | 4.98E-199 |
| CD8T | CD8Tcm | CYTOR | 1.01 | 8.89E-199 |
| CD8T | CD8Tcm | KLF4 | -1 | 1.18E-197 |
| CD8T | CD8Tcm | TPM1 | -0.92 | 1.53E-197 |
| CD8T | CD8Tcm | GLIPR2 | 0.69 | 3.27E-197 |
| CD8T | CD8Tcm | EVI2B | 0.8 | 5.85E-194 |
| CD8T | CD8Tcm | MT1E | -1.26 | 1.97E-193 |
| CD8T | CD8Tcm | RNASE1 | -1.2 | 4.98E-193 |
| CD8T | CD8Tcm | MSN | 0.75 | 1.04E-192 |
| CD8T | CD8Tcm | PPP2R5C | 1.18 | 1.33E-192 |
| CD8T | CD8Tcm | MT-CO1 | -0.94 | 4.33E-192 |
| CD8T | CD8Tcm | UQCRQ | -0.82 | 1.17E-191 |
| CD8T | CD8Tcm | RPS16 | 0.54 | 3.70E-191 |
| CD8T | CD8Tcm | RBM47 | -0.93 | 3.31E-190 |
| CD8T | CD8Tcm | ETS2 | -0.91 | 4.91E-189 |
| CD8T | CD8Tcm | AC058791.1 | 0.88 | 8.89E-189 |
| CD8T | CD8Tcm | ANXA2 | -0.96 | 1.24E-188 |
| CD8T | CD8Tcm | ZEB2 | 0.51 | 1.92E-185 |
| CD8T | CD8Tcm | AKR1B10 | -1.25 | 7.10E-185 |
| CD8T | CD8Tcm | AC016831.4 | 1.02 | 1.33E-184 |
| CD8T | CD8Tcm | KLF5 | -0.95 | 3.84E-184 |
| CD8T | CD8Tcm | HSPB1 | -0.99 | 5.48E-184 |
| CD8T | CD8Tcm | SMIM24 | -1.08 | 6.04E-184 |
| CD8T | CD8Tcm | LMO7 | -0.96 | 2.53E-181 |
| CD8T | CD8Tcm | RHOB | -0.94 | 2.78E-181 |
| CD8T | CD8Tcm | MT-ND1 | -0.81 | 6.66E-181 |
| CD8T | CD8Tcm | SRSF7 | 1.21 | 1.04E-180 |
| CD8T | CD8Tcm | TSPAN1 | -0.93 | 2.48E-180 |
| CD8T | CD8Tcm | GPRC5A | -0.96 | 1.59E-179 |
| CD8T | CD8Tcm | FCGRT | -0.82 | 8.90E-179 |
| CD8T | CD8Tcm | SPRY1 | 1.08 | 1.34E-178 |
| CD8T | CD8Tcm | MUC1 | -1.11 | 4.04E-178 |
| CD8T | CD8Tcm | VSIG2 | -0.91 | 3.30E-177 |
| CD8T | CD8Tcm | TMEM54 | -1.01 | 1.85E-175 |
| CD8T | CD8Tcm | P4HB | -0.76 | 2.46E-174 |
| CD8T | CD8Tcm | GOLM1 | -0.9 | 1.85E-172 |
| CD8T | CD8Tcm | MAL2 | -0.92 | 2.55E-172 |
| CD8T | CD8Tcm | RPS10 | 0.54 | 2.25E-169 |
| CD8T | CD8Tcm | STARD10 | -0.87 | 3.10E-169 |
| CD8T | CD8Tcm | TACSTD2 | -1.05 | 2.94E-168 |
| CD8T | CD8Tcm | UBA52 | 0.57 | 1.27E-167 |
| CD8T | CD8Tcm | MT1G | -1.45 | 5.37E-166 |
| CD8T | CD8Tcm | AKR7A3 | -0.97 | 1.16E-165 |
| CD8T | CD8Tcm | TMC5 | -0.88 | 3.42E-165 |
| CD8T | CD8Tcm | LGALS3 | -1.05 | 2.64E-164 |
| CD8T | CD8Tcm | PDLIM1 | -0.83 | 4.31E-164 |
| CD8T | CD8Tcm | FHL2 | -0.84 | 7.82E-164 |
| CD8T | CD8Tcm | MT-CYB | -0.82 | 8.18E-164 |
| CD8T | CD8Tcm | ZFP36 | 0.83 | 4.40E-163 |
| CD8T | CD8Tcm | TSC22D1 | -0.87 | 6.95E-163 |
| CD8T | CD8Tcm | CDH1 | -0.83 | 2.34E-162 |
| CD8T | CD8Tcm | PRMT9 | 0.66 | 3.38E-162 |
| CD8T | CD8Tcm | ID1 | -1.04 | 1.06E-161 |
| CD8T | CD8Tcm | CYP3A5 | -0.86 | 1.91E-161 |
| CD8T | CD8Tcm | CTNNA1 | -0.79 | 9.03E-161 |
| CD8T | CD8Tcm | ALDH1A1 | -0.87 | 8.43E-160 |
| CD8T | CD8Tcm | MDK | -1.09 | 1.16E-158 |
| CD8T | CD8Tcm | LPXN | 0.53 | 2.59E-155 |
| CD8T | CD8Tcm | CYB5A | -0.71 | 2.83E-155 |
| CD8T | CD8Tcm | MT-ND3 | -0.73 | 1.06E-154 |
| CD8T | CD8Tcm | LDLR | -0.73 | 1.76E-154 |
| CD8T | CD8Tcm | PRDX1 | -0.71 | 2.44E-153 |
| CD8T | CD8Tcm | TM9SF3 | -0.74 | 4.31E-153 |
| CD8T | CD8Tcm | LBH | 0.7 | 4.51E-153 |
| CD8T | CD8Tcm | CD59 | -0.76 | 5.51E-153 |
| CD8T | CD8Tcm | HLA-E | 0.85 | 6.95E-153 |
| CD8T | CD8Tcm | BLVRB | -0.76 | 1.26E-152 |
| CD8T | CD8Tcm | RASSF5 | 0.65 | 7.30E-152 |
| CD8T | CD8Tcm | CLDN4 | -1.48 | 1.12E-151 |
| CD8T | CD8Tcm | SLC44A4 | -0.78 | 2.53E-151 |
| CD8T | CD8Tcm | H3F3B | 0.49 | 7.60E-151 |
| CD8T | CD8Tcm | PRKCH | 0.52 | 1.85E-150 |
| CD8T | CD8Tcm | RPL27 | 0.56 | 2.61E-150 |
| CD8T | CD8Tcm | NPC2 | -0.74 | 4.83E-150 |
| CD8T | CD8Tcm | LINC00513 | 0.68 | 1.17E-148 |
| CD8T | CD8Tcm | RPL32 | 0.41 | 5.26E-147 |
| CD8T | CD8Tcm | AKR1C3 | -0.89 | 8.40E-147 |
| CD8T | CD8Tcm | DSP | -0.77 | 1.30E-146 |
| CD8T | CD8Tcm | ACTG1 | -0.61 | 1.91E-146 |
| CD8T | CD8Tcm | REG4 | -2.28 | 6.84E-146 |
| CD8T | CD8Tcm | SDCBP2 | -0.95 | 8.53E-146 |
| CD8T | CD8Tcm | TSPO | -0.69 | 4.49E-145 |
| CD8T | CD8Tcm | S100A11 | -0.72 | 1.33E-144 |
| CD8T | CD8Tcm | SRSF2 | 1.09 | 2.11E-143 |
| CD8T | CD8Tcm | CBR1 | -0.94 | 4.06E-143 |
| CD8T | CD8Tcm | SERPINB1 | -0.72 | 5.25E-143 |
| CD8T | CD8Tcm | GSN | -0.94 | 1.21E-142 |
| CD8T | CD8Tcm | MPST | -0.69 | 1.67E-142 |
| CD8T | CD8Tcm | RRBP1 | -0.68 | 2.03E-142 |
| CD8T | CD8Tcm | PDE4B | 0.61 | 8.59E-142 |
| CD8T | CD8Tcm | RPS7 | 0.47 | 4.07E-141 |
| CD8T | CD8Tcm | EEF1A1 | 0.39 | 5.63E-140 |
| CD8T | CD8Tcm | LAMB3 | -0.83 | 2.17E-139 |
| CD8T | CD8Tcm | SERPINB6 | -0.79 | 3.99E-139 |
| CD8T | CD8Tcm | EPHA2 | -0.77 | 4.45E-139 |
| CD8T | CD8Tcm | ATP1B1 | -0.78 | 1.47E-138 |
| CD8T | CD8Tcm | LSR | -0.64 | 1.01E-137 |
| CD8T | CD8Tcm | RAB11FIP1 | -0.68 | 2.36E-137 |
| CD8T | CD8Tcm | CALM1 | 0.73 | 2.88E-137 |
| CD8T | CD8Tcm | ATP5MC1 | -0.68 | 1.72E-136 |
| CD8T | CD8Tcm | ANXA4 | -0.72 | 3.25E-136 |
| CD8T | CD8Tcm | CD151 | -0.63 | 4.16E-136 |
| CD8T | CD8Tcm | TRIB1 | -0.72 | 5.64E-136 |
| CD8T | CD8Tcm | ALDH2 | -0.7 | 1.34E-135 |
| CD8T | CD8Tcm | PDE4C | -0.88 | 2.29E-135 |
| CD8T | CD8Tcm | RPS12 | 0.4 | 4.62E-134 |
| CD8T | CD8Tcm | RPL27A | 0.41 | 9.19E-134 |
| CD8T | CD8Tcm | FKBP2 | -0.6 | 1.99E-133 |
| CD8T | CD8Tcm | TSPAN3 | -0.65 | 3.16E-133 |
| CD8T | CD8Tcm | AKNA | 0.69 | 3.85E-133 |
| CD8T | CD8Tcm | RPL41 | 0.33 | 1.13E-132 |
| CD8T | CD8Tcm | TAGAP | 0.5 | 1.13E-131 |
| CD8T | CD8Tcm | EGR1 | -0.95 | 1.52E-131 |
| CD8T | CD8Tcm | TPD52 | -0.63 | 4.06E-131 |
| CD8T | CD8Tcm | KLF2 | -0.91 | 9.20E-131 |
| CD8T | CD8Tcm | CHMP4B | -0.6 | 3.16E-130 |
| CD8T | CD8Tcm | MGST3 | -0.76 | 7.94E-129 |
| CD8T | CD8Tcm | C15orf48 | -0.97 | 8.76E-129 |
| CD8T | CD8Tcm | MGST1 | -0.73 | 1.04E-128 |
| CD8T | CD8Tcm | NAPRT | -0.79 | 1.07E-128 |
| CD8T | CD8Tcm | COX5B | -0.6 | 1.32E-128 |
| CD8T | CD8Tcm | JUP | -0.65 | 2.14E-128 |
| CD8T | CD8Tcm | PSAP | -0.6 | 8.02E-128 |
| CD8T | CD8Tcm | UQCR10 | -0.66 | 1.51E-127 |
| CD8T | CD8Tcm | PLAUR | -0.75 | 2.20E-127 |
| CD8T | CD8Tcm | ABI3 | 0.46 | 3.95E-127 |
| CD8T | CD8Tcm | PRDX5 | -0.61 | 6.48E-127 |
| CD8T | CD8Tcm | ATF3 | -0.77 | 1.12E-126 |
| CD8T | CD8Tcm | AGPAT2 | -0.96 | 1.49E-126 |
| CD8T | CD8Tcm | GADD45A | -0.63 | 1.27E-125 |
| CD8T | CD8Tcm | ARPC1A | -0.61 | 2.29E-125 |
| CD8T | CD8Tcm | GAST | -1.26 | 3.38E-125 |
| CD8T | CD8Tcm | KDELR2 | -0.59 | 3.62E-125 |
| CD8T | CD8Tcm | YWHAE | -0.6 | 3.65E-125 |
| CD8T | CD8Tcm | TENT5A | -0.75 | 4.51E-125 |
| CD8T | CD8Tcm | CHST12 | 0.69 | 1.15E-124 |
| CD8T | CD8Tcm | PDIA4 | -0.61 | 4.27E-124 |
| CD8T | CD8Tcm | NR3C1 | 0.86 | 4.48E-124 |
| CD8T | CD8Tcm | CD99 | 0.94 | 2.09E-123 |
| CD8T | CD8Tcm | FOXQ1 | -0.86 | 2.13E-123 |
| CD8T | CD8Tcm | RPS20 | 0.45 | 2.30E-123 |
| CD8T | CD8Tcm | PFN1 | 0.48 | 2.36E-123 |
| CD8T | CD8Tcm | RPL38 | 0.59 | 4.76E-123 |
| CD8T | CD8Tcm | HES1 | -0.93 | 6.54E-123 |
| CD8T | CD8Tcm | MT-ND5 | -0.73 | 7.62E-123 |
| CD8T | CD8Tcm | TUBA1C | -0.6 | 9.82E-123 |
| CD8T | CD8Tcm | DNPH1 | -0.55 | 1.04E-122 |
| CD8T | CD8Tcm | TNFRSF12A | -0.69 | 1.30E-122 |
| CD8T | CD8Tcm | GMDS | -0.64 | 1.53E-121 |
| CD8T | CD8Tcm | IDS | 0.97 | 1.72E-121 |
| CD8T | CD8Tcm | ITGA6 | -0.64 | 2.89E-121 |
| CD8T | CD8Tcm | RNF166 | 0.49 | 3.82E-120 |
| CD8T | CD8Tcm | SDC4 | -0.68 | 4.01E-120 |
| CD8T | CD8Tcm | APP | -0.61 | 4.05E-120 |
| CD8T | CD8Tcm | SULT1C2 | -0.71 | 9.22E-120 |
| CD8T | CD8Tcm | CTNND1 | -0.64 | 1.74E-119 |
| CD8T | CD8Tcm | LIMA1 | -0.6 | 2.75E-119 |
| CD8T | CD8Tcm | COA3 | -0.58 | 3.75E-119 |
| CD8T | CD8Tcm | SQSTM1 | -0.61 | 4.88E-119 |
| CD8T | CD8Tcm | PTMA | 0.37 | 4.98E-119 |
| CD8T | CD8Tcm | CD63 | -0.58 | 5.78E-119 |
| CD8T | CD8Tcm | HSP90B1 | -0.64 | 7.76E-119 |
| CD8T | CD8Tcm | HSBP1 | -0.54 | 1.42E-118 |
| CD8T | CD8Tcm | CNOT6L | 0.72 | 1.46E-118 |
| CD8T | CD8Tcm | MISP | -0.7 | 1.78E-118 |
| CD8T | CD8Tcm | LIPH | -0.74 | 1.13E-117 |
| CD8T | CD8Tcm | UQCRC1 | -0.59 | 1.22E-117 |
| CD8T | CD8Tcm | ATP5MC3 | -0.68 | 1.72E-117 |
| CD8T | CD8Tcm | MT1X | -1.07 | 2.85E-117 |
| CD8T | CD8Tcm | SLPI | -0.82 | 3.80E-117 |
| CD8T | CD8Tcm | SPINT1 | -0.62 | 4.39E-117 |
| CD8T | CD8Tcm | RPS19 | 0.34 | 4.88E-117 |
| CD8T | CD8Tcm | PRSS3 | -0.88 | 2.42E-116 |
| CD8T | CD8Tcm | TXNDC17 | -0.6 | 5.73E-116 |
| CD8T | CD8Tcm | GRN | -0.59 | 6.01E-116 |
| CD8T | CD8Tcm | KMT2E | 0.97 | 7.34E-116 |
| CD8T | CD8Tcm | CXADR | -0.62 | 1.82E-115 |
| CD8T | CD8Tcm | CXCL3 | -1.18 | 1.29E-114 |
| CD8T | CD8Tcm | NECTIN2 | -0.57 | 1.50E-114 |
| CD8T | CD8Tcm | RPL37 | 0.45 | 7.04E-114 |
| CD8T | CD8Tcm | CD44 | 0.96 | 7.52E-114 |
| CD8T | CD8Tcm | RPS21 | 0.5 | 2.47E-113 |
| CD8T | CD8Tcm | ATP5ME | -0.55 | 4.74E-113 |
| CD8T | CD8Tcm | TSPAN13 | -0.59 | 1.86E-112 |
| CD8T | CD8Tcm | DSTN | -0.55 | 2.22E-112 |
| CD8T | CD8Tcm | ITGB7 | 0.48 | 7.11E-112 |
| CD8T | CD8Tcm | EMP1 | -0.79 | 1.58E-111 |
| CD8T | CD8Tcm | ATOX1 | -0.52 | 1.58E-111 |
| CD8T | CD8Tcm | IDH1 | -0.61 | 4.55E-111 |
| CD8T | CD8Tcm | CLDN7 | -0.93 | 7.74E-111 |
| CD8T | CD8Tcm | F2RL1 | -0.67 | 7.74E-110 |
| CD8T | CD8Tcm | PPDPF | -0.62 | 8.03E-110 |
| CD8T | CD8Tcm | RPS4X | 0.35 | 1.44E-107 |
| CD8T | CD8Tcm | UGDH | -0.59 | 2.23E-107 |
| CD8T | CD8Tcm | CLTA | -0.47 | 3.26E-107 |
| CD8T | CD8Tcm | SMIM14 | -0.65 | 3.71E-107 |
| CD8T | CD8Tcm | HDLBP | -0.51 | 4.44E-107 |
| CD8T | CD8Tcm | APLP2 | -0.52 | 1.88E-106 |
| CD8T | CD8Tcm | HSPA1B | -0.56 | 2.51E-106 |
| CD8T | CD8Tcm | KCNK1 | -0.61 | 4.79E-106 |
| CD8T | CD8Tcm | FAM3B | -0.6 | 5.66E-106 |
| CD8T | CD8Tcm | RPL9 | 0.38 | 7.56E-106 |
| CD8T | CD8Tcm | CD55 | -0.64 | 1.49E-105 |
| CD8T | CD8Tcm | S100A13 | -0.62 | 2.14E-105 |
| CD8T | CD8Tcm | NET1 | -0.61 | 3.86E-105 |
| CD8T | CD8Tcm | RPL19 | 0.35 | 4.16E-105 |
| CD8T | CD8Tcm | QSOX1 | -0.57 | 4.40E-105 |
| CD8T | CD8Tcm | TIMM13 | -0.49 | 1.65E-104 |
| CD8T | CD8Tcm | TFF3 | -1.78 | 1.68E-104 |
| CD8T | CD8Tcm | CES2 | -0.87 | 2.78E-104 |
| CD8T | CD8Tcm | SEM1 | -0.48 | 2.86E-104 |
| CD8T | CD8Tcm | ETFB | -0.49 | 4.90E-104 |
| CD8T | CD8Tcm | ID3 | -0.84 | 5.27E-104 |
| CD8T | CD8Tcm | CDC42SE2 | 0.98 | 9.17E-104 |
| CD8T | CD8Tcm | NCF1 | 0.48 | 2.32E-103 |
| CD8T | CD8Tcm | CCND1 | -0.65 | 5.76E-103 |
| CD8T | CD8Tcm | SERINC2 | -0.53 | 9.24E-103 |
| CD8T | CD8Tcm | RAB13 | -0.53 | 1.47E-102 |
| CD8T | CD8Tcm | TAX1BP3 | -0.54 | 2.18E-102 |
| CD8T | CD8Tcm | ERRFI1 | -0.6 | 2.28E-102 |
| CD8T | CD8Tcm | CLU | -0.86 | 2.50E-102 |
| CD8T | CD8Tcm | PPA1 | -0.54 | 6.66E-102 |
| CD8T | CD8Tcm | RPL31 | 0.39 | 7.63E-102 |
| CD8T | CD8Tcm | CTTN | -0.54 | 2.95E-101 |
| CD8T | CD8Tcm | ACADVL | -0.47 | 4.46E-101 |
| CD8T | CD8Tcm | DSG2 | -0.59 | 4.65E-101 |
| CD8T | CD8Tcm | SLIRP | -0.49 | 1.06E-100 |
| CD8T | CD8Tcm | ATP8B1 | -0.58 | 1.40E-100 |
| CD8T | CD8Tcm | AKR1A1 | -0.45 | 1.84E-100 |
| CD8T | CD8Tcm | CD9 | -0.67 | 7.42E-100 |
| CD8T | CD8Tcm | GSTA1 | -0.89 | 1.93E-99 |
| CD8T | CD8Tcm | PLPP2 | -0.51 | 2.01E-99 |
| CD8T | CD8Tcm | B3GNT5 | -0.62 | 2.09E-99 |
| CD8T | CD8Tcm | PRSS8 | -0.56 | 2.11E-99 |
| CD8T | CD8Tcm | NDUFC1 | -0.45 | 2.49E-99 |
| CD8T | CD8Tcm | PFDN5 | 0.48 | 1.16E-98 |
| CD8T | CD8Tcm | LAD1 | -0.54 | 2.52E-98 |
| CD8T | CD8Tcm | REL | 1 | 3.13E-98 |
| CD8T | CD8Tcm | ABHD2 | -0.54 | 3.67E-98 |
| CD8T | CD8Tcm | CXCL17 | -0.81 | 1.11E-97 |
| CD8T | CD8Tcm | SOX4 | -0.81 | 1.37E-97 |
| CD8T | CD8Tcm | ST14 | -0.55 | 1.85E-97 |
| CD8T | CD8Tcm | UQCRH | -0.53 | 2.26E-97 |
| CD8T | CD8Tcm | KDELR1 | -0.43 | 2.78E-97 |
| CD8T | CD8Tcm | SPATS2L | -0.56 | 2.82E-97 |
| CD8T | CD8Tcm | MGAT4A | 0.85 | 2.86E-97 |
| CD8T | CD8Tcm | TM4SF5 | -0.59 | 3.48E-97 |
| CD8T | CD8Tcm | RAB25 | -0.48 | 4.21E-97 |
| CD8T | CD8Tcm | UGCG | -0.59 | 6.22E-97 |
| CD8T | CD8Tcm | IGFBP2 | -0.59 | 1.02E-96 |
| CD8T | CD8Tcm | SCP2 | -0.5 | 1.64E-96 |
| CD8T | CD8Tcm | PLAC8 | -0.75 | 1.91E-96 |
| CD8T | CD8Tcm | ALKBH7 | -0.42 | 8.19E-96 |
| CD8T | CD8Tcm | CAPN8 | -0.63 | 1.25E-95 |
| CD8T | CD8Tcm | C6orf132 | -0.51 | 2.40E-95 |
| CD8T | CD8Tcm | TMPRSS2 | -0.57 | 3.25E-95 |
| CD8T | CD8Tcm | PPIC | -0.54 | 9.23E-95 |
| CD8T | CD8Tcm | EPS8 | -0.59 | 1.13E-94 |
| CD8T | CD8Tcm | HPGD | -0.76 | 1.45E-94 |
| CD8T | CD8Tcm | TMEM98 | -0.49 | 8.36E-94 |
| CD8T | CD8Tcm | FTL | -0.59 | 1.66E-93 |
| CD8T | CD8Tcm | SELENBP1 | -0.5 | 2.88E-93 |
| CD8T | CD8Tcm | CYC1 | -0.52 | 1.02E-92 |
| CD8T | CD8Tcm | OAZ1 | 0.51 | 1.11E-92 |
| CD8T | CD8Tcm | SPTBN1 | -0.48 | 1.36E-92 |
| CD8T | CD8Tcm | RPS3 | 0.28 | 7.43E-92 |
| CD8T | CD8Tcm | CANX | -0.39 | 1.27E-91 |
| CD8T | CD8Tcm | BCL11B | 0.51 | 1.59E-91 |
| CD8T | CD8Tcm | MSMB | -1.13 | 2.08E-91 |
| CD8T | CD8Tcm | IL1RN | -0.64 | 2.99E-91 |
| CD8T | CD8Tcm | CLDN3 | -0.84 | 6.28E-91 |
| CD8T | CD8Tcm | TSTA3 | -0.42 | 6.63E-91 |
| CD8T | CD8Tcm | RPL15 | 0.33 | 6.78E-91 |
| CD8T | CD8Tcm | RPL35A | 0.35 | 6.91E-91 |
| CD8T | CD8Tcm | PARD6B | -0.6 | 1.06E-90 |
| CD8T | CD8Tcm | ALDH3A1 | -0.74 | 1.86E-90 |
| CD8T | CD8Tcm | FABP5 | -0.85 | 1.91E-90 |
| CD8T | CD8Tcm | EMP2 | -0.51 | 3.44E-90 |
| CD8T | CD8Tcm | TCEAL9 | -0.62 | 4.76E-90 |
| CD8T | CD8Tcm | ATP5F1A | -0.47 | 7.33E-90 |
| CD8T | CD8Tcm | TMEM141 | -0.41 | 9.81E-90 |
| CD8T | CD8Tcm | LAMA3 | -0.56 | 1.29E-89 |
| CD8T | CD8Tcm | ETNK1 | -0.48 | 2.97E-89 |
| CD8T | CD8Tcm | PLXNB2 | -0.55 | 3.05E-89 |
| CD8T | CD8Tcm | NDUFS2 | -0.44 | 4.34E-89 |
| CD8T | CD8Tcm | POLD4 | -0.46 | 4.83E-89 |
| CD8T | CD8Tcm | TMED10 | -0.39 | 5.01E-89 |
| CD8T | CD8Tcm | LINC01133 | -0.74 | 1.28E-88 |
| CD8T | CD8Tcm | PPP1R16A | -0.52 | 1.45E-88 |
| CD8T | CD8Tcm | AK1 | -0.46 | 2.26E-88 |
| CD8T | CD8Tcm | RAC1 | -0.46 | 2.30E-88 |
| CD8T | CD8Tcm | COX16 | -0.39 | 2.31E-88 |
| CD8T | CD8Tcm | RPL39 | 0.29 | 2.44E-88 |
| CD8T | CD8Tcm | ECHS1 | -0.44 | 3.30E-88 |
| CD8T | CD8Tcm | TMEM258 | -0.48 | 4.25E-88 |
| CD8T | CD8Tcm | TST | -0.5 | 4.77E-88 |
| CD8T | CD8Tcm | CHMP2A | -0.39 | 6.26E-88 |
| CD8T | CD8Tcm | PCBD1 | -0.39 | 7.38E-88 |
| CD8T | CD8Tcm | TUBB2A | -0.45 | 9.46E-88 |
| CD8T | CD8Tcm | CMBL | -0.51 | 1.10E-87 |
| CD8T | CD8Tcm | MYH14 | -0.53 | 1.41E-87 |
| CD8T | CD8Tcm | DDT | -0.45 | 1.58E-87 |
| CD8T | CD8Tcm | HBEGF | -0.51 | 2.08E-87 |
| CD8T | CD8Tcm | MAPK3 | -0.54 | 2.72E-87 |
| CD8T | CD8Tcm | TTC39C | 0.49 | 6.78E-87 |
| CD8T | CD8Tcm | EFNA1 | -0.55 | 1.27E-86 |
| CD8T | CD8Tcm | AP1M2 | -0.45 | 1.65E-86 |
| CD8T | CD8Tcm | RAB8B | 0.62 | 1.83E-86 |
| CD8T | CD8Tcm | MIDN | -0.45 | 2.01E-86 |
| CD8T | CD8Tcm | PSMB5 | -0.4 | 5.18E-86 |
| CD8T | CD8Tcm | SELENOP | -0.69 | 7.55E-86 |
| CD8T | CD8Tcm | ISOC2 | -0.41 | 8.23E-86 |
| CD8T | CD8Tcm | FAM120A | -0.45 | 8.90E-86 |
| CD8T | CD8Tcm | TMBIM6 | -0.43 | 1.46E-85 |
| CD8T | CD8Tcm | COX5A | -0.43 | 2.04E-85 |
| CD8T | CD8Tcm | CTSH | -0.42 | 2.24E-85 |
| CD8T | CD8Tcm | MT2A | -0.88 | 2.36E-85 |
| CD8T | CD8Tcm | GABARAPL1 | 0.78 | 2.70E-85 |
| CD8T | CD8Tcm | RAB1A | -0.36 | 3.20E-85 |
| CD8T | CD8Tcm | ASL | -0.44 | 3.77E-85 |
| CD8T | CD8Tcm | FABP1 | -2.42 | 6.54E-85 |
| CD8T | CD8Tcm | PLA2G10 | -0.58 | 1.38E-84 |
| CD8T | CD8Tcm | FBP1 | -0.46 | 1.71E-84 |
| CD8T | CD8Tcm | AKR1C1 | -0.73 | 1.81E-84 |
| CD8T | CD8Tcm | HIGD1A | -0.56 | 2.41E-84 |
| CD8T | CD8Tcm | HMGA1 | -0.5 | 2.45E-84 |
| CD8T | CD8Tcm | RPS27L | -0.48 | 4.99E-84 |
| CD8T | CD8Tcm | MGLL | -0.48 | 7.04E-84 |
| CD8T | CD8Tcm | PKP3 | -0.55 | 7.83E-84 |
| CD8T | CD8Tcm | REG1A | -2.13 | 8.36E-84 |
| CD8T | CD8Tcm | SLC44A1 | -0.51 | 8.58E-84 |
| CD8T | CD8Tcm | MALL | -0.79 | 9.90E-84 |
| CD8T | CD8Tcm | GLUL | -0.55 | 1.07E-83 |
| CD8T | CD8Tcm | DSC2 | -0.61 | 1.12E-83 |
| CD8T | CD8Tcm | SGMS2 | -0.56 | 1.18E-83 |
| CD8T | CD8Tcm | MRPL14 | -0.39 | 1.38E-83 |
| CD8T | CD8Tcm | RPL14 | 0.38 | 1.87E-83 |
| CD8T | CD8Tcm | TMED3 | -0.39 | 2.19E-83 |
| CD8T | CD8Tcm | RNF125 | 0.61 | 2.37E-83 |
| CD8T | CD8Tcm | PLS1 | -0.53 | 3.23E-83 |
| CD8T | CD8Tcm | NDUFB1 | -0.41 | 5.68E-83 |
| CD8T | CD8Tcm | ATP5PF | -0.47 | 8.18E-83 |
| CD8T | CD8Tcm | ATP5PO | -0.43 | 1.90E-82 |
| CD8T | CD8Tcm | H2AFJ | -0.36 | 2.29E-82 |
| CD8T | CD8Tcm | RPL6 | 0.37 | 2.53E-82 |
| CD8T | CD8Tcm | PRDX2 | -0.43 | 2.58E-82 |
| CD8T | CD8Tcm | ACTB | 0.43 | 2.60E-82 |
| CD8T | CD8Tcm | PRDX3 | -0.39 | 2.91E-82 |
| CD8T | CD8Tcm | PNRC1 | 0.99 | 3.09E-82 |
| CD8T | CD8Tcm | MRPL52 | -0.38 | 4.32E-82 |
| CD8T | CD8Tcm | CTSD | -0.43 | 6.73E-82 |
| CD8T | CD8Tcm | NDUFB9 | -0.44 | 7.29E-82 |
| CD8T | CD8Tcm | RPL13A | 0.26 | 1.19E-81 |
| CD8T | CD8Tcm | NDUFB7 | -0.42 | 1.94E-81 |
| CD8T | CD8Tcm | PFKL | -0.39 | 2.24E-81 |
| CD8T | CD8Tcm | NDUFS6 | -0.4 | 2.73E-81 |
| CD8T | CD8Tcm | MRPL23 | -0.39 | 3.63E-81 |
| CD8T | CD8Tcm | MUC13 | -0.73 | 3.78E-81 |
| CD8T | CD8Tcm | RPL26 | 0.32 | 3.87E-81 |
| CD8T | CD8Tcm | NDUFS8 | -0.34 | 5.39E-81 |
| CD8T | CD8Tcm | RPL17 | 0.45 | 9.34E-81 |
| CD8T | CD8Tcm | EI24 | -0.37 | 1.79E-80 |
| CD8T | CD8Tcm | CRB3 | -0.42 | 1.87E-80 |
| CD8T | CD8Tcm | NEDD4L | -0.47 | 2.99E-80 |
| CD8T | CD8Tcm | F11R | -0.47 | 3.48E-80 |
| CD8T | CD8Tcm | CSTB | -0.46 | 4.45E-80 |
| CD8T | CD8Tcm | ACTN4 | -0.42 | 5.74E-80 |
| CD8T | CD8Tcm | KRTCAP3 | -0.4 | 7.05E-80 |
| CD8T | CD8Tcm | MARCKSL1 | -0.46 | 7.79E-80 |
| CD8T | CD8Tcm | CHP1 | -0.42 | 8.70E-80 |
| CD8T | CD8Tcm | TES | -0.4 | 1.49E-79 |
| CD8T | CD8Tcm | NDUFB3 | -0.41 | 1.84E-79 |
| CD8T | CD8Tcm | NCOA7 | -0.46 | 1.94E-79 |
| CD8T | CD8Tcm | MYDGF | -0.37 | 2.50E-79 |
| CD8T | CD8Tcm | RPSA | 0.42 | 4.96E-79 |
| CD8T | CD8Tcm | FLNB | -0.49 | 9.88E-79 |
| CD8T | CD8Tcm | MT-ND4L | -0.49 | 1.42E-78 |
| CD8T | CD8Tcm | TALDO1 | -0.35 | 1.55E-78 |
| CD8T | CD8Tcm | ITPRID2 | -0.44 | 2.29E-78 |
| CD8T | CD8Tcm | TCIM | -0.58 | 4.25E-78 |
| CD8T | CD8Tcm | TXNRD1 | -0.47 | 6.66E-78 |
| CD8T | CD8Tcm | KLF3 | -0.43 | 8.86E-78 |
| CD8T | CD8Tcm | GALE | -0.46 | 1.17E-77 |
| CD8T | CD8Tcm | GFPT1 | -0.43 | 1.17E-77 |
| CD8T | CD8Tcm | MRPL27 | -0.35 | 1.51E-77 |
| CD8T | CD8Tcm | VSIG1 | -0.66 | 1.88E-77 |
| CD8T | CD8Tcm | ATP5MPL | -0.46 | 2.30E-77 |
| CD8T | CD8Tcm | RAB11A | -0.4 | 2.66E-77 |
| CD8T | CD8Tcm | ELF1 | 0.94 | 8.12E-77 |
| CD8T | CD8Tcm | MYO6 | -0.42 | 9.05E-77 |
| CD8T | CD8Tcm | ZNHIT1 | -0.32 | 1.33E-76 |
| CD8T | CD8Tcm | ZNF706 | -0.37 | 2.08E-76 |
| CD8T | CD8Tcm | LGALS9 | -0.46 | 5.50E-76 |
| CD8T | CD8Tcm | ERGIC3 | -0.32 | 6.35E-76 |
| CD8T | CD8Tcm | ETFA | -0.36 | 6.72E-76 |
| CD8T | CD8Tcm | PRDX4 | -0.44 | 6.94E-76 |
| CD8T | CD8Tcm | ATP5IF1 | -0.43 | 8.57E-76 |
| CD8T | CD8Tcm | VIL1 | -0.49 | 9.03E-76 |
| CD8T | CD8Tcm | CYBA | 0.46 | 9.90E-76 |
| CD8T | CD8Tcm | EIF2S2 | -0.34 | 2.08E-75 |
| CD8T | CD8Tcm | PTTG1IP | -0.39 | 2.16E-75 |
| CD8T | CD8Tcm | BSG | -0.38 | 2.57E-75 |
| CD8T | CD8Tcm | ABCC3 | -0.45 | 5.14E-75 |
| CD8T | CD8Tcm | VPS35 | -0.39 | 5.79E-75 |
| CD8T | CD8Tcm | VDAC1 | -0.41 | 6.06E-75 |
| CD8T | CD8Tcm | CDC42EP1 | -0.41 | 6.20E-75 |
| CD8T | CD8Tcm | DYNLT1 | -0.36 | 6.33E-75 |
| CD8T | CD8Tcm | RAB10 | -0.35 | 1.33E-74 |
| CD8T | CD8Tcm | DDIT4 | 0.88 | 1.86E-74 |
| CD8T | CD8Tcm | PTPRF | -0.43 | 2.31E-74 |
| CD8T | CD8Tcm | FAM3D | -0.52 | 2.39E-74 |
| CD8T | CD8Tcm | MAP3K8 | 0.71 | 3.93E-74 |
| CD8T | CD8Tcm | ODF2L | 0.68 | 4.20E-74 |
| CD8T | CD8Tcm | JUNB | 0.52 | 6.17E-74 |
| CD8T | CD8Tcm | RGS10 | 0.65 | 7.21E-74 |
| CD8T | CD8Tcm | ATP5F1E | 0.35 | 7.69E-74 |
| CD8T | CD8Tcm | TMEM30B | -0.47 | 8.38E-74 |
| CD8T | CD8Tcm | SDC1 | -0.46 | 1.56E-73 |
| CD8T | CD8Tcm | LCN2 | -0.81 | 1.77E-73 |
| CD8T | CD8Tcm | CHMP3 | -0.3 | 1.91E-73 |
| CD8T | CD8Tcm | HDGF | -0.35 | 2.47E-73 |
| CD8T | CD8Tcm | PRAP1 | -1.02 | 2.75E-73 |
| CD8T | CD8Tcm | NDUFS7 | -0.36 | 4.00E-73 |
| CD8T | CD8Tcm | BACE2 | -0.45 | 5.95E-73 |
| CD8T | CD8Tcm | ITGB1 | -0.37 | 8.03E-73 |
| CD8T | CD8Tcm | SLC25A5 | -0.57 | 8.18E-73 |
| CD8T | CD8Tcm | DST | -0.47 | 1.01E-72 |
| CD8T | CD8Tcm | CD164 | -0.32 | 1.25E-72 |
| CD8T | CD8Tcm | BCAS1 | -0.57 | 1.28E-72 |
| CD8T | CD8Tcm | SH3YL1 | -0.33 | 1.38E-72 |
| CD8T | CD8Tcm | MVP | -0.38 | 1.64E-72 |
| CD8T | CD8Tcm | EPN1 | -0.39 | 1.69E-72 |
| CD8T | CD8Tcm | OCLN | -0.47 | 1.88E-72 |
| CD8T | CD8Tcm | GNG12 | -0.43 | 2.10E-72 |
| CD8T | CD8Tcm | ADI1 | -0.3 | 2.50E-72 |
| CD8T | CD8Tcm | CTSB | -0.39 | 2.55E-72 |
| CD8T | CD8Tcm | MCRIP2 | -0.32 | 3.83E-72 |
| CD8T | CD8Tcm | MRPL12 | -0.39 | 5.13E-72 |
| CD8T | CD8Tcm | HSPA1A | -0.43 | 5.36E-72 |
| CD8T | CD8Tcm | PTGER4 | 0.76 | 6.49E-72 |
| CD8T | CD8Tcm | RPN1 | -0.33 | 8.82E-72 |
| CD8T | CD8Tcm | EFHD2 | -0.43 | 1.31E-71 |
| CD8T | CD8Tcm | LGMN | -0.47 | 1.32E-71 |
| CD8T | CD8Tcm | PLEKHJ1 | -0.3 | 1.48E-71 |
| CD8T | CD8Tcm | MUC5AC | -1.3 | 1.58E-71 |
| CD8T | CD8Tcm | GALNT3 | -0.39 | 2.25E-71 |
| CD8T | CD8Tcm | H1F0 | -0.44 | 2.51E-71 |
| CD8T | CD8Tcm | BRI3 | -0.47 | 2.96E-71 |
| CD8T | CD8Tcm | RPS23 | 0.3 | 3.11E-71 |
| CD8T | CD8Tcm | VILL | -0.5 | 3.18E-71 |
| CD8T | CD8Tcm | SUB1 | 0.57 | 4.43E-71 |
| CD8T | CD8Tcm | TJP1 | -0.43 | 4.75E-71 |
| CD8T | CD8Tcm | MLPH | -0.48 | 5.35E-71 |
| CD8T | CD8Tcm | RAB27B | -0.53 | 5.70E-71 |
| CD8T | CD8Tcm | ERBB3 | -0.4 | 6.13E-71 |
| CD8T | CD8Tcm | DDX24 | 0.88 | 1.05E-70 |
| CD8T | CD8Tcm | DYNC1I2 | -0.34 | 2.30E-70 |
| CD8T | CD8Tcm | SH3BGRL2 | -0.47 | 2.62E-70 |
| CD8T | CD8Tcm | INF2 | -0.46 | 3.22E-70 |
| CD8T | CD8Tcm | ST6GALNAC1 | -0.4 | 3.45E-70 |
| CD8T | CD8Tcm | YIF1A | -0.28 | 4.36E-70 |
| CD8T | CD8Tcm | SYNGR2 | -0.29 | 5.62E-70 |
| CD8T | CD8Tcm | EML4 | 0.88 | 6.90E-70 |
| CD8T | CD8Tcm | SEPHS2 | -0.31 | 7.18E-70 |
| CD8T | CD8Tcm | COX4I1 | -0.36 | 7.41E-70 |
| CD8T | CD8Tcm | ANG | -0.43 | 9.02E-70 |
| CD8T | CD8Tcm | CXCL8 | -1.15 | 1.09E-69 |
| CD8T | CD8Tcm | KRT20 | -0.97 | 1.17E-69 |
| CD8T | CD8Tcm | ANXA3 | -0.42 | 1.17E-69 |
| CD8T | CD8Tcm | IFNGR2 | -0.45 | 1.21E-69 |
| CD8T | CD8Tcm | GPX1 | -0.35 | 1.23E-69 |
| CD8T | CD8Tcm | PDIA3 | -0.35 | 1.31E-69 |
| CD8T | CD8Tcm | SUCLG2 | -0.33 | 1.48E-69 |
| CD8T | CD8Tcm | BRD9 | 0.63 | 1.65E-69 |
| CD8T | CD8Tcm | MMP1 | -0.79 | 1.97E-69 |
| CD8T | CD8Tcm | HMGCS1 | -0.43 | 1.97E-69 |
| CD8T | CD8Tcm | TIMM8B | -0.33 | 2.67E-69 |
| CD8T | CD8Tcm | EIF4E | -0.34 | 2.99E-69 |
| CD8T | CD8Tcm | CDH17 | -0.65 | 3.22E-69 |
| CD8T | CD8Tcm | INAVA | -0.39 | 3.77E-69 |
| CD8T | CD8Tcm | VMP1 | -0.34 | 4.55E-69 |
| CD8T | CD8Tcm | SERPINB5 | -0.47 | 5.83E-69 |
| CD8T | CD8Tcm | NME1 | -0.34 | 6.75E-69 |
| CD8T | CD8Tcm | XBP1 | -0.35 | 7.47E-69 |
| CD8T | CD8Tcm | LAPTM4A | -0.36 | 7.54E-69 |
| CD8T | CD8Tcm | COX6B1 | -0.39 | 8.23E-69 |
| CD8T | CD8Tcm | ANPEP | -1.23 | 9.21E-69 |
| CD8T | CD8Tcm | LLGL2 | -0.33 | 9.60E-69 |
| CD8T | CD8Tcm | GNAQ | -0.43 | 1.02E-68 |
| CD8T | CD8Tcm | DEGS2 | -0.38 | 1.30E-68 |
| CD8T | CD8Tcm | STAP2 | -0.37 | 1.49E-68 |
| CD8T | CD8Tcm | RHPN2 | -0.42 | 2.15E-68 |
| CD8T | CD8Tcm | PHB | -0.3 | 4.14E-68 |
| CD8T | CD8Tcm | H2AFY | -0.29 | 4.32E-68 |
| CD8T | CD8Tcm | TINAGL1 | -0.4 | 6.41E-68 |
| CD8T | CD8Tcm | AADAC | -0.56 | 7.28E-68 |
| CD8T | CD8Tcm | GPR160 | -0.38 | 8.26E-68 |
| CD8T | CD8Tcm | LRRC59 | -0.29 | 8.62E-68 |
| CD8T | CD8Tcm | UQCC3 | -0.32 | 9.73E-68 |
| CD8T | CD8Tcm | ASPH | -0.37 | 1.34E-67 |
| CD8T | CD8Tcm | MIR22HG | -0.37 | 1.75E-67 |
| CD8T | CD8Tcm | ZNF593 | -0.31 | 2.56E-67 |
| CD8T | CD8Tcm | USH1C | -0.35 | 2.66E-67 |
| CD8T | CD8Tcm | PPP1R1B | -0.39 | 2.77E-67 |
| CD8T | CD8Tcm | PPP2CB | -0.41 | 2.88E-67 |
| CD8T | CD8Tcm | LASP1 | -0.38 | 2.96E-67 |
| CD8T | CD8Tcm | TMBIM1 | -0.34 | 2.98E-67 |
| CD8T | CD8Tcm | MT1F | -0.51 | 3.50E-67 |
| CD8T | CD8Tcm | NDUFC2 | -0.36 | 4.51E-67 |
| CD8T | CD8Tcm | EEF1D | 0.36 | 4.73E-67 |
| CD8T | CD8Tcm | TSPAN15 | -0.37 | 5.21E-67 |
| CD8T | CD8Tcm | EDN1 | -0.52 | 5.49E-67 |
| CD8T | CD8Tcm | MAP3K13 | -0.31 | 5.73E-67 |
| CD8T | CD8Tcm | ANXA11 | -0.3 | 5.81E-67 |
| CD8T | CD8Tcm | ADAM9 | -0.37 | 7.20E-67 |
| CD8T | CD8Tcm | SRI | -0.38 | 7.23E-67 |
| CD8T | CD8Tcm | SMIM6 | -0.47 | 7.29E-67 |
| CD8T | CD8Tcm | AHCYL1 | -0.34 | 7.33E-67 |
| CD8T | CD8Tcm | ABHD17C | -0.4 | 1.40E-66 |
| CD8T | CD8Tcm | EIF6 | -0.34 | 1.65E-66 |
| CD8T | CD8Tcm | PERP | -0.35 | 2.07E-66 |
| CD8T | CD8Tcm | KIAA1324 | -0.41 | 2.17E-66 |
| CD8T | CD8Tcm | TJP2 | -0.39 | 2.30E-66 |
| CD8T | CD8Tcm | PLLP | -0.4 | 2.64E-66 |
| CD8T | CD8Tcm | OLFM4 | -1.43 | 3.39E-66 |
| CD8T | CD8Tcm | CTSZ | -0.44 | 3.41E-66 |
| CD8T | CD8Tcm | CISD1 | -0.31 | 5.07E-66 |
| CD8T | CD8Tcm | FABP2 | -1.33 | 5.13E-66 |
| CD8T | CD8Tcm | EFNB2 | -0.43 | 7.42E-66 |
| CD8T | CD8Tcm | ATP5F1C | -0.34 | 9.32E-66 |
| CD8T | CD8Tcm | CHCHD5 | -0.28 | 1.22E-65 |
| CD8T | CD8Tcm | MYL12A | 0.56 | 1.27E-65 |
| CD8T | CD8Tcm | FOXA3 | -0.46 | 1.37E-65 |
| CD8T | CD8Tcm | TRIM16 | -0.36 | 1.47E-65 |
| CD8T | CD8Tcm | CAPNS1 | -0.31 | 1.80E-65 |
| CD8T | CD8Tcm | PEBP1 | -0.39 | 2.34E-65 |
| CD8T | CD8Tcm | TMEM176B | -0.63 | 2.43E-65 |
| CD8T | CD8Tcm | AAK1 | 0.7 | 2.71E-65 |
| CD8T | CD8Tcm | CKMT1B | -0.36 | 3.18E-65 |
| CD8T | CD8Tcm | ITGB4 | -0.39 | 3.78E-65 |
| CD8T | CD8Tcm | EIF4G1 | -0.33 | 4.00E-65 |
| CD8T | CD8Tcm | CD2AP | -0.36 | 4.35E-65 |
| CD8T | CD8Tcm | RPL11 | 0.25 | 4.43E-65 |
| CD8T | CD8Tcm | PNKD | -0.28 | 4.82E-65 |
| CD8T | CD8Tcm | TM9SF2 | -0.29 | 9.73E-65 |
| CD8T | CD8Tcm | GNA11 | -0.41 | 1.15E-64 |
| CD8T | CD8Tcm | AC026979.2 | 0.59 | 1.23E-64 |
| CD8T | CD8Tcm | CRYL1 | -0.38 | 1.31E-64 |
| CD8T | CD8Tcm | CHMP5 | -0.32 | 1.51E-64 |
| CD8T | CD8Tcm | SLC40A1 | -0.43 | 1.87E-64 |
| CD8T | CD8Tcm | TUBA1A | 0.46 | 2.01E-64 |
| CD8T | CD8Tcm | MRPS33 | -0.26 | 2.12E-64 |
| CD8T | CD8Tcm | SLC12A2 | -0.43 | 2.47E-64 |
| CD8T | CD8Tcm | STOML2 | -0.26 | 2.50E-64 |
| CD8T | CD8Tcm | ABLIM1 | -0.39 | 2.67E-64 |
| CD8T | CD8Tcm | COMT | -0.27 | 2.73E-64 |
| CD8T | CD8Tcm | SOX9 | -0.49 | 4.00E-64 |
| CD8T | CD8Tcm | BEX3 | -0.5 | 4.10E-64 |
| CD8T | CD8Tcm | MXD1 | -0.43 | 4.54E-64 |
| CD8T | CD8Tcm | AURKAIP1 | -0.33 | 5.55E-64 |
| CD8T | CD8Tcm | PDIA6 | -0.29 | 6.99E-64 |
| CD8T | CD8Tcm | DDAH1 | -0.36 | 7.10E-64 |
| CD8T | CD8Tcm | GATM | -0.41 | 7.85E-64 |
| CD8T | CD8Tcm | RPN2 | -0.25 | 8.16E-64 |
| CD8T | CD8Tcm | TESC | -0.51 | 8.81E-64 |
| CD8T | CD8Tcm | PRELID3B | -0.29 | 1.56E-63 |
| CD8T | CD8Tcm | AP1S1 | -0.29 | 1.56E-63 |
| CD8T | CD8Tcm | IMPDH2 | -0.34 | 2.13E-63 |
| CD8T | CD8Tcm | SLC25A3 | -0.36 | 2.68E-63 |
| CD8T | CD8Tcm | OCIAD2 | -0.43 | 2.79E-63 |
| CD8T | CD8Tcm | MUCL3 | -0.98 | 3.31E-63 |
| CD8T | CD8Tcm | PLPP5 | -0.33 | 3.74E-63 |
| CD8T | CD8Tcm | ALDH3A2 | -0.36 | 4.17E-63 |
| CD8T | CD8Tcm | RPL4 | 0.4 | 4.34E-63 |
| CD8T | CD8Tcm | HACD3 | -0.38 | 4.52E-63 |
| CD8T | CD8Tcm | OXNAD1 | 0.47 | 4.61E-63 |
| CD8T | CD8Tcm | SLC39A4 | -0.33 | 4.64E-63 |
| CD8T | CD8Tcm | GSTM3 | -0.32 | 4.88E-63 |
| CD8T | CD8Tcm | ATF4 | -0.31 | 4.89E-63 |
| CD8T | CD8Tcm | SMAGP | -0.3 | 7.26E-63 |
| CD8T | CD8Tcm | RIOK3 | -0.38 | 1.01E-62 |
| CD8T | CD8Tcm | TICAM1 | -0.38 | 1.23E-62 |
| CD8T | CD8Tcm | KRT7 | -0.47 | 1.24E-62 |
| CD8T | CD8Tcm | TM4SF4 | -0.82 | 1.26E-62 |
| CD8T | CD8Tcm | LRP10 | -0.3 | 1.62E-62 |
| CD8T | CD8Tcm | ARL6IP1 | -0.33 | 1.84E-62 |
| CD8T | CD8Tcm | TKT | -0.31 | 2.11E-62 |
| CD8T | CD8Tcm | FUT2 | -0.39 | 2.24E-62 |
| CD8T | CD8Tcm | COMMD6 | 0.55 | 2.85E-62 |
| CD8T | CD8Tcm | SUCLG1 | -0.29 | 3.68E-62 |
| CD8T | CD8Tcm | METTL7A | -0.43 | 3.84E-62 |
| CD8T | CD8Tcm | CXCL16 | -0.29 | 4.67E-62 |
| CD8T | CD8Tcm | NDUFB5 | -0.25 | 5.09E-62 |
| CD8T | CD8Tcm | CRACR2B | -0.33 | 5.09E-62 |
| CD8T | CD8Tcm | SHROOM3 | -0.39 | 5.36E-62 |
| CD8T | CD8Tcm | FAM102A | -0.36 | 5.70E-62 |
| CD8T | CD8Tcm | OSTC | -0.28 | 6.91E-62 |
| CD8T | CD8Tcm | NPDC1 | -0.41 | 7.79E-62 |
| CD8T | CD8Tcm | NDUFA2 | -0.26 | 1.25E-61 |
| CD8T | CD8Tcm | PTP4A2 | -0.28 | 1.34E-61 |
| CD8T | CD8Tcm | NDUFA10 | -0.26 | 1.41E-61 |
| CD8T | CD8Tcm | NDRG1 | -0.35 | 1.61E-61 |
| CD8T | CD8Tcm | NR4A1 | -0.32 | 2.64E-61 |
| CD8T | CD8Tcm | PTGR1 | -0.36 | 2.94E-61 |
| CD8T | CD8Tcm | INO80C | -0.37 | 3.55E-61 |
| CD8T | CD8Tcm | TCF7L2 | -0.39 | 4.19E-61 |
| CD8T | CD8Tcm | ITPKC | -0.45 | 5.49E-61 |
| CD8T | CD8Tcm | TNFRSF1A | -0.34 | 5.91E-61 |
| CD8T | CD8Tcm | FDFT1 | -0.29 | 6.59E-61 |
| CD8T | CD8Tcm | CYP2S1 | -0.46 | 8.34E-61 |
| CD8T | CD8Tcm | SEC61A1 | -0.33 | 9.45E-61 |
| CD8T | CD8Tcm | FBLIM1 | -0.38 | 1.13E-60 |
| CD8T | CD8Tcm | HSPD1 | -0.27 | 1.24E-60 |
| CD8T | CD8Tcm | SEC13 | -0.27 | 1.29E-60 |
| CD8T | CD8Tcm | YOD1 | -0.46 | 1.43E-60 |
| CD8T | CD8Tcm | CXCL2 | -0.85 | 1.60E-60 |
| CD8T | CD8Tcm | CERS6 | -0.36 | 1.66E-60 |
| CD8T | CD8Tcm | TCEA3 | -0.35 | 1.67E-60 |
| CD8T | CD8Tcm | PHPT1 | -0.26 | 1.69E-60 |
| CD8T | CD8Tcm | LY6E | 0.79 | 2.14E-60 |
| CD8T | CD8Tcm | MKKS | -0.26 | 2.24E-60 |
| CD8T | CD8Tcm | SLC9A1 | -0.42 | 2.24E-60 |
| CD8T | CD8Tcm | ELOVL5 | 0.5 | 2.67E-60 |
| CD8T | CD8Tcm | AHCY | -0.28 | 3.44E-60 |
| CD8T | CD8Tcm | HADH | -0.31 | 5.24E-60 |
| CD8T | CD8Tcm | QARS | -0.25 | 5.60E-60 |
| CD8T | CD8Tcm | CA9 | -0.42 | 7.44E-60 |
| CD8T | CD8Tcm | SMCO4 | -0.31 | 8.18E-60 |
| CD8T | CD8Tcm | PDLIM5 | -0.33 | 1.01E-59 |
| CD8T | CD8Tcm | AOC1 | -0.39 | 1.49E-59 |
| CD8T | CD8Tcm | VDAC2 | -0.29 | 1.49E-59 |
| CD8T | CD8Tcm | ANXA6 | 0.52 | 1.51E-59 |
| CD8T | CD8Tcm | PGRMC1 | -0.28 | 1.82E-59 |
| CD8T | CD8Tcm | PDXDC1 | -0.31 | 1.95E-59 |
| CD8T | CD8Tcm | ARHGAP5 | -0.34 | 2.34E-59 |
| CD8T | CD8Tcm | AAMDC | -0.39 | 2.57E-59 |
| CD8T | CD8Tcm | GALNT7 | -0.39 | 4.03E-59 |
| CD8T | CD8Tcm | RPL35 | 0.25 | 4.54E-59 |
| CD8T | CD8Tcm | SLC38A1 | 0.69 | 6.42E-59 |
| CD8T | CD8Tcm | GIPC1 | -0.32 | 6.48E-59 |
| CD8T | CD8Tcm | SPINT1-AS1 | -0.33 | 6.89E-59 |
| CD8T | CD8Tcm | PLIN3 | -0.31 | 1.04E-58 |
| CD8T | CD8Tcm | ATP5PD | -0.27 | 1.57E-58 |
| CD8T | CD8Tcm | TMEM205 | -0.26 | 2.48E-58 |
| CD8T | CD8Tcm | NCKAP1 | -0.33 | 3.43E-58 |
| CD8T | CD8Tcm | NDUFV3 | -0.27 | 3.45E-58 |
| CD8T | CD8Tcm | IMPA2 | -0.32 | 3.56E-58 |
| CD8T | CD8Tcm | F3 | -0.43 | 4.19E-58 |
| CD8T | CD8Tcm | NARS | -0.26 | 4.44E-58 |
| CD8T | CD8Tcm | SLC39A14 | -0.33 | 4.58E-58 |
| CD8T | CD8Tcm | TRIM2 | -0.32 | 4.77E-58 |
| CD8T | CD8Tcm | ATP5F1B | -0.32 | 4.95E-58 |
| CD8T | CD8Tcm | ARF4 | -0.28 | 5.90E-58 |
| CD8T | CD8Tcm | GGCT | -0.3 | 6.04E-58 |
| CD8T | CD8Tcm | EDF1 | -0.34 | 6.87E-58 |
| CD8T | CD8Tcm | TMEM134 | -0.27 | 6.89E-58 |
| CD8T | CD8Tcm | GNG5 | -0.28 | 7.50E-58 |
| CD8T | CD8Tcm | FRMD4B | -0.32 | 7.67E-58 |
| CD8T | CD8Tcm | BAIAP2L1 | -0.38 | 7.98E-58 |
| CD8T | CD8Tcm | ATP2A2 | -0.29 | 1.10E-57 |
| CD8T | CD8Tcm | PIK3IP1 | 0.48 | 1.14E-57 |
| CD8T | CD8Tcm | BCAR1 | -0.36 | 1.14E-57 |
| CD8T | CD8Tcm | RASEF | -0.48 | 1.22E-57 |
| CD8T | CD8Tcm | ARPC2 | 0.57 | 1.25E-57 |
| CD8T | CD8Tcm | HMGN3 | -0.26 | 1.36E-57 |
| CD8T | CD8Tcm | TP53I3 | -0.33 | 1.41E-57 |
| CD8T | CD8Tcm | PLK2 | -0.37 | 1.79E-57 |
| CD8T | CD8Tcm | RARRES3 | 0.74 | 2.30E-57 |
| CD8T | CD8Tcm | SELENOW | -0.33 | 2.70E-57 |
| CD8T | CD8Tcm | HTATIP2 | -0.28 | 4.21E-57 |
| CD8T | CD8Tcm | WASL | -0.3 | 4.32E-57 |
| CD8T | CD8Tcm | RPL5 | 0.32 | 4.99E-57 |
| CD8T | CD8Tcm | SLC22A18 | -0.37 | 7.32E-57 |
| CD8T | CD8Tcm | YIPF3 | -0.26 | 1.69E-56 |
| CD8T | CD8Tcm | ADM | -0.44 | 2.63E-56 |
| CD8T | CD8Tcm | PLEC | -0.33 | 3.90E-56 |
| CD8T | CD8Tcm | NBL1 | -0.32 | 3.91E-56 |
| CD8T | CD8Tcm | NORAD | -0.26 | 6.64E-56 |
| CD8T | CD8Tcm | PYCR1 | -0.32 | 7.55E-56 |
| CD8T | CD8Tcm | CDKN1A | -0.29 | 7.65E-56 |
| CD8T | CD8Tcm | MGAT4B | -0.33 | 1.01E-55 |
| CD8T | CD8Tcm | TNFRSF21 | -0.36 | 1.06E-55 |
| CD8T | CD8Tcm | TMC4 | -0.31 | 1.29E-55 |
| CD8T | CD8Tcm | ETHE1 | -0.37 | 1.55E-55 |
| CD8T | CD8Tcm | TUBB4B | -0.29 | 1.95E-55 |
| CD8T | CD8Tcm | CMAS | -0.3 | 1.98E-55 |
| CD8T | CD8Tcm | ACSL5 | -0.42 | 3.24E-55 |
| CD8T | CD8Tcm | RPL36A | 0.35 | 3.37E-55 |
| CD8T | CD8Tcm | IFT172 | -0.3 | 3.38E-55 |
| CD8T | CD8Tcm | TRIM31 | -0.39 | 3.59E-55 |
| CD8T | CD8Tcm | DECR1 | -0.31 | 4.08E-55 |
| CD8T | CD8Tcm | UBE2J1 | -0.27 | 8.15E-55 |
| CD8T | CD8Tcm | GCNT3 | -0.38 | 8.22E-55 |
| CD8T | CD8Tcm | SH3RF1 | -0.34 | 8.22E-55 |
| CD8T | CD8Tcm | C6orf141 | -0.4 | 9.20E-55 |
| CD8T | CD8Tcm | PDZK1IP1 | -0.35 | 9.36E-55 |
| CD8T | CD8Tcm | YWHAH | -0.28 | 9.58E-55 |
| CD8T | CD8Tcm | PGRMC2 | -0.26 | 1.21E-54 |
| CD8T | CD8Tcm | ASS1 | -0.33 | 1.50E-54 |
| CD8T | CD8Tcm | ADAM15 | -0.34 | 1.94E-54 |
| CD8T | CD8Tcm | LMO4 | -0.32 | 2.01E-54 |
| CD8T | CD8Tcm | PLEK2 | -0.29 | 2.20E-54 |
| CD8T | CD8Tcm | MYO5B | -0.32 | 2.23E-54 |
| CD8T | CD8Tcm | USP36 | 0.64 | 2.24E-54 |
| CD8T | CD8Tcm | WDR34 | -0.29 | 2.39E-54 |
| CD8T | CD8Tcm | ERGIC1 | -0.26 | 2.51E-54 |
| CD8T | CD8Tcm | EPS8L3 | -0.28 | 2.70E-54 |
| CD8T | CD8Tcm | MIR4458HG | -0.34 | 3.03E-54 |
| CD8T | CD8Tcm | FDPS | -0.26 | 3.44E-54 |
| CD8T | CD8Tcm | SRCAP | -0.33 | 3.50E-54 |
| CD8T | CD8Tcm | HSD17B2 | -0.44 | 3.87E-54 |
| CD8T | CD8Tcm | ACADS | -0.3 | 4.09E-54 |
| CD8T | CD8Tcm | IRF6 | -0.33 | 4.26E-54 |
| CD8T | CD8Tcm | AHNAK | -0.28 | 4.42E-54 |
| CD8T | CD8Tcm | ACOX1 | -0.33 | 5.11E-54 |
| CD8T | CD8Tcm | PSCA | -1.13 | 5.48E-54 |
| CD8T | CD8Tcm | ROMO1 | -0.29 | 5.92E-54 |
| CD8T | CD8Tcm | SGK1 | -0.35 | 6.64E-54 |
| CD8T | CD8Tcm | TMEM176A | -0.45 | 6.75E-54 |
| CD8T | CD8Tcm | MAOA | -0.32 | 8.15E-54 |
| CD8T | CD8Tcm | FOSL1 | -0.3 | 9.02E-54 |
| CD8T | CD8Tcm | SUN1 | -0.31 | 9.12E-54 |
| CD8T | CD8Tcm | SQLE | -0.34 | 1.10E-53 |
| CD8T | CD8Tcm | SCOC | -0.26 | 1.16E-53 |
| CD8T | CD8Tcm | CDHR5 | -0.51 | 1.24E-53 |
| CD8T | CD8Tcm | ZDHHC3 | -0.26 | 1.50E-53 |
| CD8T | CD8Tcm | CKMT1A | -0.29 | 1.86E-53 |
| CD8T | CD8Tcm | RETSAT | -0.35 | 2.01E-53 |
| CD8T | CD8Tcm | CKAP4 | -0.37 | 2.53E-53 |
| CD8T | CD8Tcm | NR2F6 | -0.37 | 2.71E-53 |
| CD8T | CD8Tcm | EZR | -0.35 | 2.99E-53 |
| CD8T | CD8Tcm | SLC35A3 | -0.27 | 3.21E-53 |
| CD8T | CD8Tcm | RASSF7 | -0.26 | 3.95E-53 |
| CD8T | CD8Tcm | ADD3 | -0.3 | 4.36E-53 |
| CD8T | CD8Tcm | KCNN4 | -0.32 | 4.70E-53 |
| CD8T | CD8Tcm | SSBP3 | -0.34 | 6.76E-53 |
| CD8T | CD8Tcm | GTF2I | -0.27 | 7.25E-53 |
| CD8T | CD8Tcm | TFF1 | -1.89 | 1.42E-52 |
| CD8T | CD8Tcm | CLTC | -0.27 | 1.44E-52 |
| CD8T | CD8Tcm | CREB3L1 | -0.34 | 1.45E-52 |
| CD8T | CD8Tcm | SMIM31 | -0.37 | 1.50E-52 |
| CD8T | CD8Tcm | MT-ND2 | -0.5 | 2.16E-52 |
| CD8T | CD8Tcm | HNRNPA1 | 0.39 | 3.81E-52 |
| CD8T | CD8Tcm | TOMM7 | 0.44 | 3.87E-52 |
| CD8T | CD8Tcm | STX3 | -0.3 | 4.01E-52 |
| CD8T | CD8Tcm | SEC61G | -0.37 | 4.44E-52 |
| CD8T | CD8Tcm | B4GALT5 | -0.32 | 4.96E-52 |
| CD8T | CD8Tcm | CENPX | -0.26 | 6.52E-52 |
| CD8T | CD8Tcm | RPS26 | 0.4 | 7.11E-52 |
| CD8T | CD8Tcm | FAM3C | -0.28 | 8.16E-52 |
| CD8T | CD8Tcm | YBX3 | -0.31 | 1.01E-51 |
| CD8T | CD8Tcm | ITGA2 | -0.33 | 1.18E-51 |
| CD8T | CD8Tcm | LMAN1 | -0.26 | 1.26E-51 |
| CD8T | CD8Tcm | DMBT1 | -0.56 | 1.46E-51 |
| CD8T | CD8Tcm | SPAG9 | -0.29 | 1.50E-51 |
| CD8T | CD8Tcm | MSRB2 | -0.27 | 1.73E-51 |
| CD8T | CD8Tcm | DHCR24 | -0.35 | 2.03E-51 |
| CD8T | CD8Tcm | MINOS1 | -0.28 | 3.19E-51 |
| CD8T | CD8Tcm | PDCD6IP | -0.25 | 3.38E-51 |
| CD8T | CD8Tcm | RNPEP | -0.3 | 3.39E-51 |
| CD8T | CD8Tcm | MAPK6 | -0.27 | 4.97E-51 |
| CD8T | CD8Tcm | PRXL2A | -0.26 | 5.37E-51 |
| CD8T | CD8Tcm | CLTB | -0.3 | 5.86E-51 |
| CD8T | CD8Tcm | HNF4A | -0.33 | 6.27E-51 |
| CD8T | CD8Tcm | VEGFA | -0.37 | 7.42E-51 |
| CD8T | CD8Tcm | DGAT1 | -0.5 | 7.59E-51 |
| CD8T | CD8Tcm | RPL24 | 0.33 | 7.81E-51 |
| CD8T | CD8Tcm | CAMK2N1 | -0.32 | 8.81E-51 |
| CD8T | CD8Tcm | IGLL5 | -2.09 | 8.92E-51 |
| CD8T | CD8Tcm | IFI16 | 0.54 | 1.06E-50 |
| CD8T | CD8Tcm | JAG1 | -0.38 | 1.07E-50 |
| CD8T | CD8Tcm | CD68 | -0.31 | 1.88E-50 |
| CD8T | CD8Tcm | PNP | -0.25 | 2.07E-50 |
| CD8T | CD8Tcm | TFF2 | -1.41 | 2.34E-50 |
| CD8T | CD8Tcm | EPS8L1 | -0.39 | 2.38E-50 |
| CD8T | CD8Tcm | WFDC2 | -0.43 | 2.49E-50 |
| CD8T | CD8Tcm | PARP8 | 0.49 | 2.64E-50 |
| CD8T | CD8Tcm | ZBTB43 | -0.28 | 3.04E-50 |
| CD8T | CD8Tcm | ATP5MF | -0.32 | 3.32E-50 |
| CD8T | CD8Tcm | CLDN23 | -0.34 | 3.77E-50 |
| CD8T | CD8Tcm | TOM1L1 | -0.27 | 4.66E-50 |
| CD8T | CD8Tcm | TMEM45B | -0.3 | 5.30E-50 |
| CD8T | CD8Tcm | FERMT1 | -0.29 | 5.79E-50 |
| CD8T | CD8Tcm | CLCN3 | -0.28 | 5.83E-50 |
| CD8T | CD8Tcm | IBTK | -0.28 | 6.51E-50 |
| CD8T | CD8Tcm | GSKIP | -0.25 | 1.13E-49 |
| CD8T | CD8Tcm | CNN3 | -0.37 | 1.34E-49 |
| CD8T | CD8Tcm | ALDOB | -0.74 | 2.59E-49 |
| CD8T | CD8Tcm | TIMP1 | -0.48 | 2.69E-49 |
| CD8T | CD8Tcm | PRR13 | -0.34 | 3.72E-49 |
| CD8T | CD8Tcm | GPAT3 | -0.34 | 3.92E-49 |
| CD8T | CD8Tcm | GPRC5C | -0.29 | 5.60E-49 |
| CD8T | CD8Tcm | LTBR | -0.25 | 6.11E-49 |
| CD8T | CD8Tcm | WEE1 | -0.32 | 6.66E-49 |
| CD8T | CD8Tcm | SAT1 | -0.32 | 6.77E-49 |
| CD8T | CD8Tcm | JPT1 | -0.36 | 1.07E-48 |
| CD8T | CD8Tcm | FAM84B | -0.3 | 1.17E-48 |
| CD8T | CD8Tcm | CTBP2 | -0.29 | 1.27E-48 |
| CD8T | CD8Tcm | TMEM59 | -0.33 | 1.44E-48 |
| CD8T | CD8Tcm | NAP1L1 | 0.7 | 1.60E-48 |
| CD8T | CD8Tcm | LAMC2 | -0.32 | 2.89E-48 |
| CD8T | CD8Tcm | GATA6 | -0.33 | 3.39E-48 |
| CD8T | CD8Tcm | NR0B2 | -0.37 | 3.48E-48 |
| CD8T | CD8Tcm | SULT1A1 | -0.41 | 4.16E-48 |
| CD8T | CD8Tcm | HID1 | -0.28 | 4.23E-48 |
| CD8T | CD8Tcm | CBLC | -0.25 | 4.55E-48 |
| CD8T | CD8Tcm | RALA | -0.25 | 4.59E-48 |
| CD8T | CD8Tcm | RNASE4 | -0.34 | 5.11E-48 |
| CD8T | CD8Tcm | ICAM3 | 0.7 | 5.88E-48 |
| CD8T | CD8Tcm | OAT | -0.27 | 7.30E-48 |
| CD8T | CD8Tcm | BCL10 | -0.25 | 8.63E-48 |
| CD8T | CD8Tcm | PON2 | -0.26 | 8.88E-48 |
| CD8T | CD8Tcm | AKR1C2 | -0.49 | 8.90E-48 |
| CD8T | CD8Tcm | SLC7A8 | -0.43 | 1.11E-47 |
| CD8T | CD8Tcm | RFLNA | -0.4 | 1.31E-47 |
| CD8T | CD8Tcm | SLC20A1 | -0.29 | 1.44E-47 |
| CD8T | CD8Tcm | TRAK1 | -0.28 | 1.53E-47 |
| CD8T | CD8Tcm | ARHGEF35 | -0.28 | 1.93E-47 |
| CD8T | CD8Tcm | MIA | -0.53 | 1.99E-47 |
| CD8T | CD8Tcm | RAP2B | -0.36 | 2.11E-47 |
| CD8T | CD8Tcm | APOBEC3C | 0.55 | 2.52E-47 |
| CD8T | CD8Tcm | PELI1 | -0.3 | 2.52E-47 |
| CD8T | CD8Tcm | BCL2L15 | -0.29 | 3.20E-47 |
| CD8T | CD8Tcm | GDF15 | -0.45 | 3.71E-47 |
| CD8T | CD8Tcm | RNF128 | -0.31 | 7.03E-47 |
| CD8T | CD8Tcm | SGSM3 | -0.29 | 7.21E-47 |
| CD8T | CD8Tcm | PRKCI | -0.31 | 7.80E-47 |
| CD8T | CD8Tcm | CAPN5 | -0.31 | 7.80E-47 |
| CD8T | CD8Tcm | ZG16B | -0.45 | 9.89E-47 |
| CD8T | CD8Tcm | IL18 | -0.27 | 1.40E-46 |
| CD8T | CD8Tcm | LAPTM4B | -0.26 | 1.62E-46 |
| CD8T | CD8Tcm | CLDN15 | -0.28 | 2.19E-46 |
| CD8T | CD8Tcm | RPS24 | 0.29 | 2.21E-46 |
| CD8T | CD8Tcm | RASSF6 | -0.33 | 2.22E-46 |
| CD8T | CD8Tcm | KLHL21 | -0.29 | 2.38E-46 |
| CD8T | CD8Tcm | ATP1B3 | 0.83 | 4.52E-46 |
| CD8T | CD8Tcm | DDR1 | -0.26 | 5.50E-46 |
| CD8T | CD8Tcm | VCL | -0.25 | 5.52E-46 |
| CD8T | CD8Tcm | KLF10 | -0.28 | 6.90E-46 |
| CD8T | CD8Tcm | FAM129B | -0.26 | 1.02E-45 |
| CD8T | CD8Tcm | GPA33 | -0.36 | 1.08E-45 |
| CD8T | CD8Tcm | CHMP4C | -0.26 | 1.13E-45 |
| CD8T | CD8Tcm | C3orf52 | -0.32 | 1.14E-45 |
| CD8T | CD8Tcm | CDS1 | -0.27 | 1.19E-45 |
| CD8T | CD8Tcm | CDC42EP4 | -0.27 | 1.44E-45 |
| CD8T | CD8Tcm | BAG3 | -0.29 | 1.50E-45 |
| CD8T | CD8Tcm | RAMP1 | -0.27 | 2.24E-45 |
| CD8T | CD8Tcm | ANKRD22 | -0.26 | 2.71E-45 |
| CD8T | CD8Tcm | MAP2K2 | -0.27 | 3.09E-45 |
| CD8T | CD8Tcm | ADGRE5 | 0.79 | 3.26E-45 |
| CD8T | CD8Tcm | GOLIM4 | -0.26 | 4.11E-45 |
| CD8T | CD8Tcm | SERF2 | -0.3 | 4.86E-45 |
| CD8T | CD8Tcm | COPG1 | -0.26 | 5.30E-45 |
| CD8T | CD8Tcm | ELOB | -0.31 | 5.49E-45 |
| CD8T | CD8Tcm | FAR1 | -0.28 | 6.22E-45 |
| CD8T | CD8Tcm | MYO1A | -0.31 | 7.01E-45 |
| CD8T | CD8Tcm | GCNT1 | -0.26 | 8.13E-45 |
| CD8T | CD8Tcm | CMTM7 | 0.44 | 8.42E-45 |
| CD8T | CD8Tcm | SOD2 | -0.3 | 8.64E-45 |
| CD8T | CD8Tcm | ERBB2 | -0.29 | 9.66E-45 |
| CD8T | CD8Tcm | AC020656.1 | -1.04 | 1.22E-44 |
| CD8T | CD8Tcm | PCK1 | -0.53 | 1.46E-44 |
| CD8T | CD8Tcm | LAMP2 | -0.25 | 1.84E-44 |
| CD8T | CD8Tcm | HMGCS2 | -0.32 | 1.93E-44 |
| CD8T | CD8Tcm | IGF2BP2 | -0.27 | 2.37E-44 |
| CD8T | CD8Tcm | TM4SF20 | -0.61 | 2.48E-44 |
| CD8T | CD8Tcm | FXYD5 | 0.74 | 2.55E-44 |
| CD8T | CD8Tcm | CDCP1 | -0.26 | 3.11E-44 |
| CD8T | CD8Tcm | ABRACL | 0.78 | 3.59E-44 |
| CD8T | CD8Tcm | GCLC | -0.35 | 3.86E-44 |
| CD8T | CD8Tcm | CORO1C | -0.26 | 4.21E-44 |
| CD8T | CD8Tcm | EDEM3 | -0.29 | 6.25E-44 |
| CD8T | CD8Tcm | MYO1C | -0.25 | 9.50E-44 |
| CD8T | CD8Tcm | SIK1 | -0.31 | 1.04E-43 |
| CD8T | CD8Tcm | PDCL3 | 0.71 | 1.37E-43 |
| CD8T | CD8Tcm | KIAA1522 | -0.27 | 1.49E-43 |
| CD8T | CD8Tcm | COX7A2 | -0.36 | 1.79E-43 |
| CD8T | CD8Tcm | SOCS3 | -0.53 | 2.66E-43 |
| CD8T | CD8Tcm | MT1H | -0.56 | 4.63E-43 |
| CD8T | CD8Tcm | CEACAM6 | -0.46 | 4.98E-43 |
| CD8T | CD8Tcm | LYAR | 0.59 | 5.44E-43 |
| CD8T | CD8Tcm | CYBRD1 | -0.39 | 5.51E-43 |
| CD8T | CD8Tcm | ESRP1 | -0.26 | 5.63E-43 |
| CD8T | CD8Tcm | MECOM | -0.28 | 8.25E-43 |
| CD8T | CD8Tcm | AZGP1 | -0.31 | 8.58E-43 |
| CD8T | CD8Tcm | HOOK2 | -0.25 | 1.16E-42 |
| CD8T | CD8Tcm | PLCB3 | -0.26 | 1.39E-42 |
| CD8T | CD8Tcm | FLNA | 0.44 | 2.02E-42 |
| CD8T | CD8Tcm | NFIB | -0.28 | 2.25E-42 |
| CD8T | CD8Tcm | RND3 | -0.33 | 2.33E-42 |
| CD8T | CD8Tcm | MEIS2 | -0.33 | 2.66E-42 |
| CD8T | CD8Tcm | MUC17 | -0.31 | 4.04E-42 |
| CD8T | CD8Tcm | TRNP1 | -0.34 | 5.93E-42 |
| CD8T | CD8Tcm | MYO15B | -0.32 | 6.70E-42 |
| CD8T | CD8Tcm | PKP2 | -0.25 | 7.04E-42 |
| CD8T | CD8Tcm | CCDC68 | -0.27 | 7.95E-42 |
| CD8T | CD8Tcm | NFIA | -0.27 | 9.74E-42 |
| CD8T | CD8Tcm | MSMO1 | -0.27 | 1.04E-41 |
| CD8T | CD8Tcm | SCD | -0.26 | 1.10E-41 |
| CD8T | CD8Tcm | SERPINA1 | -0.44 | 1.11E-41 |
| CD8T | CD8Tcm | FGFBP1 | -0.28 | 1.15E-41 |
| CD8T | CD8Tcm | SCNN1A | -0.29 | 1.43E-41 |
| CD8T | CD8Tcm | PBX1 | -0.31 | 1.59E-41 |
| CD8T | CD8Tcm | PRSS22 | -0.25 | 1.70E-41 |
| CD8T | CD8Tcm | KDELR3 | -0.26 | 2.10E-41 |
| CD8T | CD8Tcm | C11orf86 | -0.59 | 3.10E-41 |
| CD8T | CD8Tcm | OSTF1 | 0.74 | 3.54E-41 |
| CD8T | CD8Tcm | COX6C | -0.45 | 4.06E-41 |
| CD8T | CD8Tcm | ARHGAP12 | -0.28 | 5.11E-41 |
| CD8T | CD8Tcm | AC020916.1 | 0.78 | 5.17E-41 |
| CD8T | CD8Tcm | SOCS6 | -0.26 | 5.46E-41 |
| CD8T | CD8Tcm | RUNX1 | -0.26 | 7.20E-41 |
| CD8T | CD8Tcm | FAM83H | -0.25 | 7.59E-41 |
| CD8T | CD8Tcm | DDC | -0.27 | 7.62E-41 |
| CD8T | CD8Tcm | ACTN1 | -0.25 | 7.71E-41 |
| CD8T | CD8Tcm | RPL23 | 0.31 | 7.92E-41 |
| CD8T | CD8Tcm | PDK4 | -0.35 | 8.12E-41 |
| CD8T | CD8Tcm | RBP2 | -1.3 | 8.76E-41 |
| CD8T | CD8Tcm | 7-Sep | 0.75 | 9.15E-41 |
| CD8T | CD8Tcm | ITGAV | -0.29 | 9.21E-41 |
| CD8T | CD8Tcm | FAM83G | -0.27 | 9.54E-41 |
| CD8T | CD8Tcm | CDC42EP2 | -0.26 | 1.02E-40 |
| CD8T | CD8Tcm | VAMP2 | 0.68 | 1.04E-40 |
| CD8T | CD8Tcm | SULT1B1 | -0.29 | 1.96E-40 |
| CD8T | CD8Tcm | ATP5F1D | -0.27 | 6.14E-40 |
| CD8T | CD8Tcm | CXCL1 | -0.54 | 6.46E-40 |
| CD8T | CD8Tcm | PLCD3 | -0.27 | 8.22E-40 |
| CD8T | CD8Tcm | SLC39A11 | -0.33 | 8.66E-40 |
| CD8T | CD8Tcm | HRASLS2 | -0.46 | 9.89E-40 |
| CD8T | CD8Tcm | PCDH1 | -0.26 | 1.21E-39 |
| CD8T | CD8Tcm | CYP2C18 | -0.29 | 1.47E-39 |
| CD8T | CD8Tcm | KCNE3 | -0.26 | 1.53E-39 |
| CD8T | CD8Tcm | CKB | -0.28 | 2.34E-39 |
| CD8T | CD8Tcm | GALNT6 | -0.3 | 2.37E-39 |
| CD8T | CD8Tcm | SRSF5 | 0.7 | 2.56E-39 |
| CD8T | CD8Tcm | LGR4 | -0.25 | 5.02E-39 |
| CD8T | CD8Tcm | ANXA13 | -0.4 | 5.43E-39 |
| CD8T | CD8Tcm | PMP22 | -0.42 | 6.93E-39 |
| CD8T | CD8Tcm | SEZ6L2 | -0.26 | 6.93E-39 |
| CD8T | CD8Tcm | CPS1 | -0.25 | 1.56E-38 |
| CD8T | CD8Tcm | PAXX | 0.78 | 2.00E-38 |
| CD8T | CD8Tcm | COX6A1 | -0.31 | 2.14E-38 |
| CD8T | CD8Tcm | NDUFA1 | -0.28 | 2.37E-38 |
| CD8T | CD8Tcm | HLA-DRA | -0.64 | 6.62E-38 |
| CD8T | CD8Tcm | DNMBP | -0.26 | 9.48E-38 |
| CD8T | CD8Tcm | RSRP1 | 0.73 | 1.38E-37 |
| CD8T | CD8Tcm | MICALL2 | -0.26 | 2.99E-37 |
| CD8T | CD8Tcm | FTH1 | 0.43 | 3.88E-37 |
| CD8T | CD8Tcm | TUBA4A | 0.81 | 5.31E-37 |
| CD8T | CD8Tcm | RGS2 | 0.73 | 9.52E-37 |
| CD8T | CD8Tcm | ME1 | -0.26 | 1.55E-36 |
| CD8T | CD8Tcm | DEPP1 | -0.38 | 2.31E-36 |
| CD8T | CD8Tcm | EPHX1 | -0.25 | 2.43E-36 |
| CD8T | CD8Tcm | SI | -0.36 | 2.99E-36 |
| CD8T | CD8Tcm | KLK1 | -0.28 | 3.26E-36 |
| CD8T | CD8Tcm | FA2H | -0.26 | 4.17E-36 |
| CD8T | CD8Tcm | GKN2 | -1.51 | 4.97E-36 |
| CD8T | CD8Tcm | CLDND1 | 0.69 | 5.15E-36 |
| CD8T | CD8Tcm | TFPI | -0.26 | 6.82E-36 |
| CD8T | CD8Tcm | NRARP | -0.26 | 7.49E-36 |
| CD8T | CD8Tcm | INSR | -0.32 | 9.80E-36 |
| CD8T | CD8Tcm | C1orf116 | -0.25 | 1.78E-35 |
| CD8T | CD8Tcm | RIPK4 | -0.25 | 1.99E-35 |
| CD8T | CD8Tcm | AC023090.1 | -0.35 | 3.16E-35 |
| CD8T | CD8Tcm | TMEM63B | -0.26 | 4.11E-35 |
| CD8T | CD8Tcm | CACFD1 | -0.28 | 6.79E-35 |
| CD8T | CD8Tcm | KHK | -0.28 | 8.32E-35 |
| CD8T | CD8Tcm | MT1M | -0.33 | 1.19E-34 |
| CD8T | CD8Tcm | IL1R2 | -0.3 | 1.65E-34 |
| CD8T | CD8Tcm | ACY3 | -0.28 | 2.31E-34 |
| CD8T | CD8Tcm | CDKN1B | 0.49 | 3.00E-34 |
| CD8T | CD8Tcm | EMB | 0.54 | 3.75E-34 |
| CD8T | CD8Tcm | CITED2 | 0.62 | 5.43E-34 |
| CD8T | CD8Tcm | ISG15 | 0.61 | 1.68E-33 |
| CD8T | CD8Tcm | ATP5MD | -0.31 | 3.91E-33 |
| CD8T | CD8Tcm | PABPC1 | 0.39 | 5.86E-33 |
| CD8T | CD8Tcm | CAVIN3 | -0.26 | 7.37E-33 |
| CD8T | CD8Tcm | IER2 | -0.33 | 1.25E-32 |
| CD8T | CD8Tcm | MBNL1 | 0.67 | 1.26E-32 |
| CD8T | CD8Tcm | SLC7A11 | -0.32 | 2.23E-32 |
| CD8T | CD8Tcm | KLK11 | -0.27 | 3.33E-32 |
| CD8T | CD8Tcm | IGFBP4 | -0.4 | 5.74E-32 |
| CD8T | CD8Tcm | IFITM3 | -0.4 | 7.02E-32 |
| CD8T | CD8Tcm | ABCC5 | -0.26 | 8.14E-32 |
| CD8T | CD8Tcm | OTUD1 | -0.32 | 9.44E-32 |
| CD8T | CD8Tcm | MS4A8 | -0.33 | 9.58E-32 |
| CD8T | CD8Tcm | TMPRSS15 | -0.49 | 9.61E-32 |
| CD8T | CD8Tcm | NDUFA4 | -0.27 | 1.54E-31 |
| CD8T | CD8Tcm | HNRNPDL | 0.6 | 1.63E-31 |
| CD8T | CD8Tcm | PRSS23 | -0.38 | 7.78E-31 |
| CD8T | CD8Tcm | MYADM | 0.71 | 1.28E-30 |
| CD8T | CD8Tcm | CRIP1 | 0.4 | 2.52E-30 |
| CD8T | CD8Tcm | TPPP3 | -0.34 | 6.51E-30 |
| CD8T | CD8Tcm | TENT5C | 0.52 | 2.02E-29 |
| CD8T | CD8Tcm | ETS1 | 0.55 | 2.23E-29 |
| CD8T | CD8Tcm | ARID5A | 0.54 | 2.31E-29 |
| CD8T | CD8Tcm | CRIP2 | -0.34 | 3.27E-29 |
| CD8T | CD8Tcm | ITM2C | 0.59 | 1.47E-28 |
| CD8T | CD8Tcm | ARPC1B | 0.63 | 2.04E-28 |
| CD8T | CD8Tcm | CTGF | -0.27 | 2.07E-28 |
| CD8T | CD8Tcm | CEACAM5 | -0.35 | 2.45E-28 |
| CD8T | CD8Tcm | CYR61 | -0.32 | 2.90E-28 |
| CD8T | CD8Tcm | JCHAIN | -2.53 | 3.35E-28 |
| CD8T | CD8Tcm | HSPG2 | -0.27 | 3.62E-28 |
| CD8T | CD8Tcm | C4orf48 | 0.38 | 4.52E-28 |
| CD8T | CD8Tcm | RND1 | -0.27 | 8.06E-28 |
| CD8T | CD8Tcm | PAIP2 | 0.66 | 8.86E-28 |
| CD8T | CD8Tcm | TMA7 | 0.26 | 3.69E-27 |
| CD8T | CD8Tcm | B4GALNT3 | -0.26 | 6.92E-27 |
| CD8T | CD8Tcm | SELENOK | 0.45 | 5.55E-26 |
| CD8T | CD8Tcm | SLC9A3R2 | -0.39 | 8.65E-26 |
| CD8T | CD8Tcm | CBLB | 0.45 | 1.42E-25 |
| CD8T | CD8Tcm | BPIFB1 | -0.71 | 2.06E-25 |
| CD8T | CD8Tcm | HLA-DRB5 | -0.26 | 2.65E-25 |
| CD8T | CD8Tcm | RPL22 | 0.25 | 3.52E-25 |
| CD8T | CD8Tcm | NUPR1 | -0.32 | 3.67E-25 |
| CD8T | CD8Tcm | RHOG | 0.63 | 4.10E-25 |
| CD8T | CD8Tcm | TERF2IP | 0.59 | 4.49E-25 |
| CD8T | CD8Tcm | RPL36AL | 0.29 | 4.78E-25 |
| CD8T | CD8Tcm | HERPUD2 | 0.5 | 5.16E-25 |
| CD8T | CD8Tcm | PAM | -0.25 | 1.02E-24 |
| CD8T | CD8Tcm | PCLAF | -0.26 | 1.02E-24 |
| CD8T | CD8Tcm | SNHG15 | 0.59 | 1.28E-24 |
| CD8T | CD8Tcm | TUBA1B | 0.55 | 7.87E-24 |
| CD8T | CD8Tcm | SNRPD2 | 0.44 | 1.59E-23 |
| CD8T | CD8Tcm | JUND | -0.27 | 4.25E-23 |
| CD8T | CD8Tcm | SSR4 | -0.52 | 1.31E-22 |
| CD8T | CD8Tcm | DDX5 | 0.35 | 2.61E-22 |
| CD8T | CD8Tcm | FOSB | -0.28 | 5.16E-22 |
| CD8T | CD8Tcm | CCNH | 0.53 | 4.65E-21 |
| CD8T | CD8Tcm | CALM2 | -0.27 | 9.87E-21 |
| CD8T | CD8Tcm | C9orf78 | 0.64 | 9.96E-21 |
| CD8T | CD8Tcm | PGC | -1.6 | 1.06E-20 |
| CD8T | CD8Tcm | HMGB2 | 0.6 | 1.29E-20 |
| CD8T | CD8Tcm | UCP2 | 0.58 | 6.10E-20 |
| CD8T | CD8Tcm | HMGB1 | 0.31 | 6.23E-20 |
| CD8T | CD8Tcm | ARID4B | 0.6 | 1.26E-19 |
| CD8T | CD8Tcm | CCNI | 0.55 | 2.78E-19 |
| CD8T | CD8Tcm | SCGB2A1 | -0.39 | 1.37E-18 |
| CD8T | CD8Tcm | FGFR1OP2 | 0.57 | 1.38E-18 |
| CD8T | CD8Tcm | CIRBP | 0.36 | 1.41E-18 |
| CD8T | CD8Tcm | GYG1 | 0.46 | 2.55E-18 |
| CD8T | CD8Tcm | C1orf56 | 0.46 | 3.29E-18 |
| CD8T | CD8Tcm | CD320 | -0.4 | 4.87E-18 |
| CD8T | CD8Tcm | BIRC3 | 0.6 | 5.50E-18 |
| CD8T | CD8Tcm | DRAP1 | 0.64 | 7.24E-18 |
| CD8T | CD8Tcm | PER1 | 0.4 | 1.05E-17 |
| CD8T | CD8Tcm | ARL6IP5 | 0.6 | 3.44E-17 |
| CD8T | CD8Tcm | RBM39 | 0.49 | 4.38E-17 |
| CD8T | CD8Tcm | NPM1 | 0.25 | 4.56E-17 |
| CD8T | CD8Tcm | JUN | -0.31 | 8.81E-17 |
| CD8T | CD8Tcm | ITM2B | 0.29 | 1.27E-16 |
| CD8T | CD8Tcm | PSAPL1 | -0.38 | 2.82E-16 |
| CD8T | CD8Tcm | SUMO2 | 0.31 | 3.06E-16 |
| CD8T | CD8Tcm | OST4 | 0.27 | 5.68E-16 |
| CD8T | CD8Tcm | LITAF | 0.69 | 5.81E-16 |
| CD8T | CD8Tcm | PSME1 | 0.49 | 1.22E-15 |
| CD8T | CD8Tcm | DHX36 | 0.58 | 2.17E-15 |
| CD8T | CD8Tcm | CCND3 | 0.5 | 2.69E-15 |
| CD8T | CD8Tcm | HERPUD1 | -0.26 | 2.74E-15 |
| CD8T | CD8Tcm | PRR4 | -0.54 | 6.23E-15 |
| CD8T | CD8Tcm | SRSF3 | 0.46 | 1.53E-14 |
| CD8T | CD8Tcm | XIST | 0.38 | 1.87E-14 |
| CD8T | CD8Tcm | RNF213 | 0.55 | 3.32E-14 |
| CD8T | CD8Tcm | PHF20 | 0.39 | 8.03E-14 |
| CD8T | CD8Tcm | PELO | 0.46 | 8.90E-14 |
| CD8T | CD8Tcm | SRRT | 0.48 | 2.62E-13 |
| CD8T | CD8Tcm | STK17A | 0.53 | 3.08E-13 |
| CD8T | CD8Tcm | PRNP | 0.48 | 3.13E-13 |
| CD8T | CD8Tcm | UBE2D3 | 0.38 | 7.91E-13 |
| CD8T | CD8Tcm | HMGN1 | 0.42 | 8.07E-13 |
| CD8T | CD8Tcm | FAM49B | 0.53 | 1.07E-12 |
| CD8T | CD8Tcm | ARHGEF1 | 0.41 | 1.19E-12 |
| CD8T | CD8Tcm | NOP53 | 0.36 | 1.40E-12 |
| CD8T | CD8Tcm | G3BP2 | 0.64 | 1.45E-12 |
| CD8T | CD8Tcm | HLA-F | 0.52 | 2.11E-12 |
| CD8T | CD8Tcm | PDCD4 | 0.57 | 9.22E-12 |
| CD8T | CD8Tcm | PBXIP1 | 0.42 | 1.21E-11 |
| CD8T | CD8Tcm | RBM8A | 0.54 | 1.41E-11 |
| CD8T | CD8Tcm | SLC7A5 | 0.37 | 3.50E-11 |
| CD8T | CD8Tcm | DNAJB6 | 0.55 | 6.35E-11 |
| CD8T | CD8Tcm | PRPF38B | 0.59 | 9.29E-11 |
| CD8T | CD8Tcm | ARPC3 | 0.36 | 1.98E-10 |
| CD8T | CD8Tcm | FUS | 0.54 | 2.23E-10 |
| CD8T | CD8Tcm | NEU1 | 0.37 | 2.35E-10 |
| CD8T | CD8Tcm | HNRNPA0 | 0.47 | 4.78E-10 |
| CD8T | CD8Tcm | CDC42SE1 | 0.47 | 5.37E-10 |
| CD8T | CD8Tcm | C19orf66 | 0.4 | 1.50E-09 |
| CD8T | CD8Tcm | ISCA1 | 0.53 | 1.50E-09 |
| CD8T | CD8Tcm | ARRB2 | 0.46 | 1.53E-09 |
| CD8T | CD8Tcm | MED10 | 0.54 | 1.75E-09 |
| CD8T | CD8Tcm | HNRNPUL1 | 0.54 | 2.87E-09 |
| CD8T | CD8Tcm | RCAN3 | 0.36 | 6.05E-09 |
| CD8T | CD8Tcm | MUC6 | -0.71 | 6.42E-09 |
| CD8T | CD8Tcm | CNN2 | 0.44 | 2.00E-08 |
| CD8T | CD8Tcm | TRMT112 | 0.42 | 4.31E-08 |
| CD8T | CD8Tcm | MTFP1 | 0.46 | 1.09E-07 |
| CD8T | CD8Tcm | H2AFZ | 0.31 | 1.22E-07 |
| CD8T | CD8Tcm | PPP1R2 | 0.52 | 1.69E-07 |
| CD8T | CD8Tcm | EPC1 | 0.51 | 1.82E-07 |
| CD8T | CD8Tcm | BUB3 | 0.51 | 1.93E-07 |
| CD8T | CD8Tcm | PPP1CB | 0.52 | 4.70E-07 |
| CD8T | CD8Tcm | TPM3 | 0.4 | 5.44E-07 |
| CD8T | CD8Tcm | CHD1 | 0.45 | 1.05E-06 |
| CD8T | CD8Tcm | HIPK1 | 0.37 | 1.93E-06 |
| CD8T | CD8Tcm | RNMT | 0.43 | 2.03E-06 |
| CD8T | CD8Tcm | MCL1 | 0.38 | 2.82E-06 |
| CD8T | CD8Tcm | COTL1 | 0.47 | 2.94E-06 |
| CD8T | CD8Tcm | NKTR | 0.43 | 4.41E-06 |
| CD8T | CD8Tcm | CMC2 | 0.46 | 4.76E-06 |
| CD8T | CD8Tcm | STOM | 0.28 | 5.80E-06 |
| CD8T | CD8Tcm | GKN1 | -1.53 | 6.35E-06 |
| CD8T | CD8Tcm | DNAJC9 | 0.37 | 6.78E-06 |
| CD8T | CD8Tcm | CDC42 | 0.37 | 8.33E-06 |
| CD8T | CD8Tcm | OFD1 | 0.41 | 9.16E-06 |
| Pit mucous | Pit mucous | RBP2 | 3.22 | 0 |
| Pit mucous | Pit mucous | APOA4 | 3.22 | 0 |
| Pit mucous | Pit mucous | APOA1 | 3.21 | 0 |
| Pit mucous | Pit mucous | FABP2 | 3 | 0 |
| Pit mucous | Pit mucous | FABP1 | 2.91 | 0 |
| Pit mucous | Pit mucous | APOC3 | 2.68 | 0 |
| Pit mucous | Pit mucous | ANPEP | 2.38 | 0 |
| Pit mucous | Pit mucous | ALDOB | 2.33 | 0 |
| Pit mucous | Pit mucous | PRAP1 | 2.31 | 0 |
| Pit mucous | Pit mucous | PHGR1 | 2.21 | 0 |
| Pit mucous | Pit mucous | TM4SF20 | 2.08 | 0 |
| Pit mucous | Pit mucous | C11orf86 | 2.02 | 0 |
| Pit mucous | Pit mucous | ADIRF | 1.95 | 0 |
| Pit mucous | Pit mucous | AGPAT2 | 1.91 | 0 |
| Pit mucous | Pit mucous | KRT20 | 1.89 | 0 |
| Pit mucous | Pit mucous | MTTP | 1.88 | 0 |
| Pit mucous | Pit mucous | TMPRSS15 | 1.84 | 0 |
| Pit mucous | Pit mucous | C3orf85 | 1.83 | 0 |
| Pit mucous | Pit mucous | TM4SF4 | 1.81 | 0 |
| Pit mucous | Pit mucous | PCK1 | 1.8 | 0 |
| Pit mucous | Pit mucous | IL32 | 1.7 | 0 |
| Pit mucous | Pit mucous | ACSL5 | 1.69 | 0 |
| Pit mucous | Pit mucous | MUC13 | 1.69 | 0 |
| Pit mucous | Pit mucous | DGAT1 | 1.68 | 0 |
| Pit mucous | Pit mucous | MALL | 1.65 | 0 |
| Pit mucous | Pit mucous | GSTA1 | 1.65 | 0 |
| Pit mucous | Pit mucous | APOB | 1.65 | 0 |
| Pit mucous | Pit mucous | ACY3 | 1.63 | 0 |
| Pit mucous | Pit mucous | CDH17 | 1.61 | 0 |
| Pit mucous | Pit mucous | CBR1 | 1.6 | 0 |
| Pit mucous | Pit mucous | ACE2 | 1.58 | 0 |
| Pit mucous | Pit mucous | ANXA13 | 1.54 | 0 |
| Pit mucous | Pit mucous | SMIM24 | 1.53 | 0 |
| Pit mucous | Pit mucous | CYBRD1 | 1.51 | 0 |
| Pit mucous | Pit mucous | SULT1A1 | 1.51 | 0 |
| Pit mucous | Pit mucous | SI | 1.48 | 0 |
| Pit mucous | Pit mucous | LGALS3 | 1.47 | 0 |
| Pit mucous | Pit mucous | LDHA | 1.47 | 0 |
| Pit mucous | Pit mucous | PMP22 | 1.43 | 0 |
| Pit mucous | Pit mucous | CES2 | 1.43 | 0 |
| Pit mucous | Pit mucous | CDHR5 | 1.38 | 0 |
| Pit mucous | Pit mucous | SLC6A19 | 1.37 | 0 |
| Pit mucous | Pit mucous | MGST3 | 1.37 | 0 |
| Pit mucous | Pit mucous | HSD17B2 | 1.37 | 0 |
| Pit mucous | Pit mucous | CLDN7 | 1.36 | 0 |
| Pit mucous | Pit mucous | PLAC8 | 1.35 | 0 |
| Pit mucous | Pit mucous | TSPAN8 | 1.34 | 0 |
| Pit mucous | Pit mucous | LGALS4 | 1.34 | 0 |
| Pit mucous | Pit mucous | HIGD1A | 1.34 | 0 |
| Pit mucous | Pit mucous | GLRX | 1.33 | 0 |
| Pit mucous | Pit mucous | CREB3L3 | 1.32 | 0 |
| Pit mucous | Pit mucous | SDCBP2 | 1.32 | 0 |
| Pit mucous | Pit mucous | COX6C | 1.31 | 0 |
| Pit mucous | Pit mucous | KHK | 1.31 | 0 |
| Pit mucous | Pit mucous | ABCG2 | 1.3 | 0 |
| Pit mucous | Pit mucous | GPA33 | 1.26 | 0 |
| Pit mucous | Pit mucous | PRSS3 | 1.26 | 0 |
| Pit mucous | Pit mucous | EPCAM | 1.25 | 0 |
| Pit mucous | Pit mucous | FAM3C | 1.25 | 0 |
| Pit mucous | Pit mucous | HRASLS2 | 1.25 | 0 |
| Pit mucous | Pit mucous | VIL1 | 1.25 | 0 |
| Pit mucous | Pit mucous | CLDN3 | 1.24 | 0 |
| Pit mucous | Pit mucous | AKR7A3 | 1.23 | 0 |
| Pit mucous | Pit mucous | AKR1B10 | 1.23 | 0 |
| Pit mucous | Pit mucous | PRR13 | 1.22 | 0 |
| Pit mucous | Pit mucous | SULT1A2 | 1.22 | 0 |
| Pit mucous | Pit mucous | CDA | 1.21 | 0 |
| Pit mucous | Pit mucous | CLDN4 | 1.21 | 0 |
| Pit mucous | Pit mucous | S100A16 | 1.19 | 0 |
| Pit mucous | Pit mucous | SLC5A1 | 1.19 | 0 |
| Pit mucous | Pit mucous | S100A14 | 1.19 | 0 |
| Pit mucous | Pit mucous | HPGD | 1.19 | 0 |
| Pit mucous | Pit mucous | HTATIP2 | 1.18 | 0 |
| Pit mucous | Pit mucous | RHOC | 1.18 | 0 |
| Pit mucous | Pit mucous | S100A10 | 1.17 | 0 |
| Pit mucous | Pit mucous | APOBEC1 | 1.16 | 0 |
| Pit mucous | Pit mucous | ACTB | 1.16 | 0 |
| Pit mucous | Pit mucous | CIDEC | 1.16 | 0 |
| Pit mucous | Pit mucous | CTSZ | 1.16 | 0 |
| Pit mucous | Pit mucous | CIDEB | 1.14 | 0 |
| Pit mucous | Pit mucous | SLC26A3 | 1.12 | 0 |
| Pit mucous | Pit mucous | SERPINB6 | 1.12 | 0 |
| Pit mucous | Pit mucous | SELENOP | 1.12 | 0 |
| Pit mucous | Pit mucous | AADAC | 1.11 | 0 |
| Pit mucous | Pit mucous | NAPRT | 1.11 | 0 |
| Pit mucous | Pit mucous | OAT | 1.11 | 0 |
| Pit mucous | Pit mucous | CDHR2 | 1.1 | 0 |
| Pit mucous | Pit mucous | SFN | 1.1 | 0 |
| Pit mucous | Pit mucous | PSME2 | 1.09 | 0 |
| Pit mucous | Pit mucous | C15orf48 | 1.09 | 0 |
| Pit mucous | Pit mucous | PIGR | 1.09 | 0 |
| Pit mucous | Pit mucous | REG4 | 1.09 | 0 |
| Pit mucous | Pit mucous | TXNDC17 | 1.08 | 0 |
| Pit mucous | Pit mucous | MUC17 | 1.08 | 0 |
| Pit mucous | Pit mucous | CRYL1 | 1.08 | 0 |
| Pit mucous | Pit mucous | SLC9A3R1 | 1.08 | 0 |
| Pit mucous | Pit mucous | EIF6 | 1.07 | 0 |
| Pit mucous | Pit mucous | SLC25A5 | 1.06 | 0 |
| Pit mucous | Pit mucous | CYP2D6 | 1.06 | 0 |
| Pit mucous | Pit mucous | PEPD | 1.06 | 0 |
| Pit mucous | Pit mucous | DECR1 | 1.05 | 0 |
| Pit mucous | Pit mucous | ISG20 | 1.05 | 0 |
| Pit mucous | Pit mucous | MYO1A | 1.05 | 0 |
| Pit mucous | Pit mucous | PCK2 | 1.05 | 0 |
| Pit mucous | Pit mucous | SMIM31 | 1.04 | 0 |
| Pit mucous | Pit mucous | PLS1 | 1.04 | 0 |
| Pit mucous | Pit mucous | CDKN2B-AS1 | 1.04 | 0 |
| Pit mucous | Pit mucous | FUOM | 1.04 | 0 |
| Pit mucous | Pit mucous | CA1 | 1.03 | 0 |
| Pit mucous | Pit mucous | ANXA2 | 1.03 | 0 |
| Pit mucous | Pit mucous | SLC51B | 1.02 | 0 |
| Pit mucous | Pit mucous | JPT1 | 1.02 | 0 |
| Pit mucous | Pit mucous | ATP5MC3 | 1.01 | 0 |
| Pit mucous | Pit mucous | SLC15A1 | 0.98 | 0 |
| Pit mucous | Pit mucous | MISP | 0.98 | 0 |
| Pit mucous | Pit mucous | TM4SF5 | 0.98 | 0 |
| Pit mucous | Pit mucous | PLEC | 0.97 | 0 |
| Pit mucous | Pit mucous | VAMP8 | 0.97 | 0 |
| Pit mucous | Pit mucous | MYO15B | 0.96 | 0 |
| Pit mucous | Pit mucous | LINC02404 | 0.96 | 0 |
| Pit mucous | Pit mucous | MEP1A | 0.96 | 0 |
| Pit mucous | Pit mucous | ETHE1 | 0.96 | 0 |
| Pit mucous | Pit mucous | VDAC2 | 0.96 | 0 |
| Pit mucous | Pit mucous | PPP1R14D | 0.95 | 0 |
| Pit mucous | Pit mucous | PEBP1 | 0.95 | 0 |
| Pit mucous | Pit mucous | NDUFB9 | 0.94 | 0 |
| Pit mucous | Pit mucous | AOC1 | 0.94 | 0 |
| Pit mucous | Pit mucous | PPP1R16A | 0.94 | 0 |
| Pit mucous | Pit mucous | CFL1 | 0.94 | 0 |
| Pit mucous | Pit mucous | ANXA10 | 0.94 | 0 |
| Pit mucous | Pit mucous | MYL12B | 0.94 | 0 |
| Pit mucous | Pit mucous | ATP5PF | 0.94 | 0 |
| Pit mucous | Pit mucous | AGR3 | 0.93 | 0 |
| Pit mucous | Pit mucous | COX6A1 | 0.93 | 0 |
| Pit mucous | Pit mucous | FBP1 | 0.93 | 0 |
| Pit mucous | Pit mucous | SLC22A18 | 0.92 | 0 |
| Pit mucous | Pit mucous | SRI | 0.9 | 0 |
| Pit mucous | Pit mucous | HEBP1 | 0.9 | 0 |
| Pit mucous | Pit mucous | SLC35G1 | 0.9 | 0 |
| Pit mucous | Pit mucous | KRT8 | 0.9 | 0 |
| Pit mucous | Pit mucous | CHP2 | 0.9 | 0 |
| Pit mucous | Pit mucous | CALM1 | 0.89 | 0 |
| Pit mucous | Pit mucous | S100A6 | 0.89 | 0 |
| Pit mucous | Pit mucous | GNG12 | 0.88 | 0 |
| Pit mucous | Pit mucous | SLC39A4 | 0.88 | 0 |
| Pit mucous | Pit mucous | GK | 0.88 | 0 |
| Pit mucous | Pit mucous | ADA | 0.88 | 0 |
| Pit mucous | Pit mucous | RHOF | 0.88 | 0 |
| Pit mucous | Pit mucous | TDP2 | 0.88 | 0 |
| Pit mucous | Pit mucous | ACAA1 | 0.87 | 0 |
| Pit mucous | Pit mucous | TMEM54 | 0.87 | 0 |
| Pit mucous | Pit mucous | RAB30 | 0.87 | 0 |
| Pit mucous | Pit mucous | SCP2 | 0.86 | 0 |
| Pit mucous | Pit mucous | PPP2CB | 0.86 | 0 |
| Pit mucous | Pit mucous | ACY1 | 0.86 | 0 |
| Pit mucous | Pit mucous | ALPI | 0.86 | 0 |
| Pit mucous | Pit mucous | SOD1 | 0.86 | 0 |
| Pit mucous | Pit mucous | HHLA2 | 0.85 | 0 |
| Pit mucous | Pit mucous | GDA | 0.84 | 0 |
| Pit mucous | Pit mucous | ARPC1B | 0.84 | 0 |
| Pit mucous | Pit mucous | THOP1 | 0.84 | 0 |
| Pit mucous | Pit mucous | GDPD3 | 0.84 | 0 |
| Pit mucous | Pit mucous | SLC20A1 | 0.84 | 0 |
| Pit mucous | Pit mucous | LAPTM4B | 0.84 | 0 |
| Pit mucous | Pit mucous | AL365226.2 | 0.84 | 0 |
| Pit mucous | Pit mucous | SUCLG1 | 0.84 | 0 |
| Pit mucous | Pit mucous | UQCRQ | 0.83 | 0 |
| Pit mucous | Pit mucous | PFN1 | 0.83 | 0 |
| Pit mucous | Pit mucous | GNA11 | 0.83 | 0 |
| Pit mucous | Pit mucous | ATP1B3 | 0.82 | 0 |
| Pit mucous | Pit mucous | GUCA2B | 0.82 | 0 |
| Pit mucous | Pit mucous | MPP1 | 0.82 | 0 |
| Pit mucous | Pit mucous | REEP6 | 0.82 | 0 |
| Pit mucous | Pit mucous | ESPN | 0.82 | 0 |
| Pit mucous | Pit mucous | GPT | 0.81 | 0 |
| Pit mucous | Pit mucous | EMP1 | 0.81 | 0 |
| Pit mucous | Pit mucous | GCNT3 | 0.81 | 0 |
| Pit mucous | Pit mucous | SLC51A | 0.81 | 0 |
| Pit mucous | Pit mucous | GSTO1 | 0.81 | 0 |
| Pit mucous | Pit mucous | HSD17B11 | 0.8 | 0 |
| Pit mucous | Pit mucous | TPMT | 0.8 | 0 |
| Pit mucous | Pit mucous | COX7A2 | 0.8 | 0 |
| Pit mucous | Pit mucous | SLC25A3 | 0.8 | 0 |
| Pit mucous | Pit mucous | HIST1H1C | 0.79 | 0 |
| Pit mucous | Pit mucous | ISG15 | 0.79 | 0 |
| Pit mucous | Pit mucous | COX7B | 0.79 | 0 |
| Pit mucous | Pit mucous | SAT2 | 0.79 | 0 |
| Pit mucous | Pit mucous | ZG16 | 0.79 | 0 |
| Pit mucous | Pit mucous | CMAS | 0.79 | 0 |
| Pit mucous | Pit mucous | POLD4 | 0.78 | 0 |
| Pit mucous | Pit mucous | GHITM | 0.78 | 0 |
| Pit mucous | Pit mucous | GPAT3 | 0.77 | 0 |
| Pit mucous | Pit mucous | MME | 0.77 | 0 |
| Pit mucous | Pit mucous | CEACAM6 | 0.77 | 0 |
| Pit mucous | Pit mucous | OAS1 | 0.77 | 0 |
| Pit mucous | Pit mucous | NR0B2 | 0.76 | 0 |
| Pit mucous | Pit mucous | ANXA4 | 0.76 | 0 |
| Pit mucous | Pit mucous | ABHD11-AS1 | 0.76 | 0 |
| Pit mucous | Pit mucous | GSTA2 | 0.76 | 0 |
| Pit mucous | Pit mucous | CEACAM1 | 0.75 | 0 |
| Pit mucous | Pit mucous | CLDN15 | 0.75 | 0 |
| Pit mucous | Pit mucous | GBP3 | 0.75 | 0 |
| Pit mucous | Pit mucous | DSC2 | 0.75 | 0 |
| Pit mucous | Pit mucous | ACADS | 0.75 | 0 |
| Pit mucous | Pit mucous | SLC13A2 | 0.74 | 0 |
| Pit mucous | Pit mucous | AP3S1 | 0.73 | 0 |
| Pit mucous | Pit mucous | ATP5MF | 0.73 | 0 |
| Pit mucous | Pit mucous | SULT1B1 | 0.73 | 0 |
| Pit mucous | Pit mucous | MVP | 0.73 | 0 |
| Pit mucous | Pit mucous | MYL6 | 0.73 | 0 |
| Pit mucous | Pit mucous | TMEM45B | 0.73 | 0 |
| Pit mucous | Pit mucous | PRELID3B | 0.72 | 0 |
| Pit mucous | Pit mucous | HADHA | 0.72 | 0 |
| Pit mucous | Pit mucous | ADH4 | 0.72 | 0 |
| Pit mucous | Pit mucous | TMCC3 | 0.72 | 0 |
| Pit mucous | Pit mucous | OTOP3 | 0.72 | 0 |
| Pit mucous | Pit mucous | VNN1 | 0.72 | 0 |
| Pit mucous | Pit mucous | MXD1 | 0.72 | 0 |
| Pit mucous | Pit mucous | MYO7B | 0.71 | 0 |
| Pit mucous | Pit mucous | CAMK2N1 | 0.71 | 0 |
| Pit mucous | Pit mucous | HKDC1 | 0.71 | 0 |
| Pit mucous | Pit mucous | MT-CO2 | 0.71 | 0 |
| Pit mucous | Pit mucous | HNF4G | 0.7 | 0 |
| Pit mucous | Pit mucous | PTGR1 | 0.7 | 0 |
| Pit mucous | Pit mucous | ACO2 | 0.7 | 0 |
| Pit mucous | Pit mucous | RNF186 | 0.7 | 0 |
| Pit mucous | Pit mucous | MTCH2 | 0.69 | 0 |
| Pit mucous | Pit mucous | SLC7A9 | 0.69 | 0 |
| Pit mucous | Pit mucous | NCOA4 | 0.69 | 0 |
| Pit mucous | Pit mucous | TM6SF2 | 0.69 | 0 |
| Pit mucous | Pit mucous | SAR1B | 0.69 | 0 |
| Pit mucous | Pit mucous | C2orf88 | 0.69 | 0 |
| Pit mucous | Pit mucous | HADHB | 0.69 | 0 |
| Pit mucous | Pit mucous | MYH14 | 0.69 | 0 |
| Pit mucous | Pit mucous | B3GNT5 | 0.68 | 0 |
| Pit mucous | Pit mucous | COX5B | 0.68 | 0 |
| Pit mucous | Pit mucous | MEP1B | 0.68 | 0 |
| Pit mucous | Pit mucous | GPD1 | 0.68 | 0 |
| Pit mucous | Pit mucous | TNFRSF1A | 0.68 | 0 |
| Pit mucous | Pit mucous | ERICH4 | 0.68 | 0 |
| Pit mucous | Pit mucous | UPP1 | 0.68 | 0 |
| Pit mucous | Pit mucous | LPGAT1 | 0.67 | 0 |
| Pit mucous | Pit mucous | BTNL8 | 0.67 | 0 |
| Pit mucous | Pit mucous | SLC2A5 | 0.66 | 0 |
| Pit mucous | Pit mucous | DHRS1 | 0.66 | 0 |
| Pit mucous | Pit mucous | C1QTNF12 | 0.65 | 0 |
| Pit mucous | Pit mucous | TKFC | 0.65 | 0 |
| Pit mucous | Pit mucous | CTSA | 0.65 | 0 |
| Pit mucous | Pit mucous | OTC | 0.65 | 0 |
| Pit mucous | Pit mucous | COX6B1 | 0.65 | 0 |
| Pit mucous | Pit mucous | PLIN3 | 0.65 | 0 |
| Pit mucous | Pit mucous | ETFA | 0.65 | 0 |
| Pit mucous | Pit mucous | SDHD | 0.64 | 0 |
| Pit mucous | Pit mucous | GFER | 0.64 | 0 |
| Pit mucous | Pit mucous | PLIN2 | 0.64 | 0 |
| Pit mucous | Pit mucous | CD68 | 0.64 | 0 |
| Pit mucous | Pit mucous | SLC39A5 | 0.63 | 0 |
| Pit mucous | Pit mucous | IQGAP2 | 0.63 | 0 |
| Pit mucous | Pit mucous | TMEM253 | 0.62 | 0 |
| Pit mucous | Pit mucous | SURF4 | 0.62 | 0 |
| Pit mucous | Pit mucous | LAMA1 | 0.62 | 0 |
| Pit mucous | Pit mucous | C8G | 0.62 | 0 |
| Pit mucous | Pit mucous | CCL25 | 0.62 | 0 |
| Pit mucous | Pit mucous | PLCXD1 | 0.62 | 0 |
| Pit mucous | Pit mucous | FGFBP1 | 0.61 | 0 |
| Pit mucous | Pit mucous | FLNB | 0.61 | 0 |
| Pit mucous | Pit mucous | SLC46A1 | 0.6 | 0 |
| Pit mucous | Pit mucous | C1orf115 | 0.6 | 0 |
| Pit mucous | Pit mucous | RIOK3 | 0.6 | 0 |
| Pit mucous | Pit mucous | UGT2A3 | 0.6 | 0 |
| Pit mucous | Pit mucous | RHOD | 0.6 | 0 |
| Pit mucous | Pit mucous | XDH | 0.6 | 0 |
| Pit mucous | Pit mucous | PLGRKT | 0.6 | 0 |
| Pit mucous | Pit mucous | RGS2 | 0.59 | 0 |
| Pit mucous | Pit mucous | OGDH | 0.59 | 0 |
| Pit mucous | Pit mucous | ATP2B1 | 0.59 | 0 |
| Pit mucous | Pit mucous | RALA | 0.59 | 0 |
| Pit mucous | Pit mucous | PRSS8 | 0.59 | 0 |
| Pit mucous | Pit mucous | SDHA | 0.59 | 0 |
| Pit mucous | Pit mucous | CFDP1 | 0.59 | 0 |
| Pit mucous | Pit mucous | STAP2 | 0.58 | 0 |
| Pit mucous | Pit mucous | MOGAT2 | 0.58 | 0 |
| Pit mucous | Pit mucous | TMED4 | 0.58 | 0 |
| Pit mucous | Pit mucous | GOLT1A | 0.58 | 0 |
| Pit mucous | Pit mucous | REEP3 | 0.58 | 0 |
| Pit mucous | Pit mucous | DPP4 | 0.58 | 0 |
| Pit mucous | Pit mucous | COX4I1 | 0.57 | 0 |
| Pit mucous | Pit mucous | MOGAT3 | 0.56 | 0 |
| Pit mucous | Pit mucous | DST | 0.56 | 0 |
| Pit mucous | Pit mucous | BCL2L15 | 0.56 | 0 |
| Pit mucous | Pit mucous | RHPN2 | 0.55 | 0 |
| Pit mucous | Pit mucous | CNIH4 | 0.55 | 0 |
| Pit mucous | Pit mucous | PFKP | 0.55 | 0 |
| Pit mucous | Pit mucous | FKBP1B | 0.55 | 0 |
| Pit mucous | Pit mucous | ASS1 | 0.55 | 0 |
| Pit mucous | Pit mucous | PDLIM2 | 0.54 | 0 |
| Pit mucous | Pit mucous | PLCB3 | 0.54 | 0 |
| Pit mucous | Pit mucous | XPNPEP2 | 0.53 | 0 |
| Pit mucous | Pit mucous | AQP10 | 0.53 | 0 |
| Pit mucous | Pit mucous | BCL2L1 | 0.53 | 0 |
| Pit mucous | Pit mucous | COL17A1 | 0.53 | 0 |
| Pit mucous | Pit mucous | HEPH | 0.53 | 0 |
| Pit mucous | Pit mucous | TMEM82 | 0.52 | 0 |
| Pit mucous | Pit mucous | ACOX1 | 0.52 | 0 |
| Pit mucous | Pit mucous | AQP11 | 0.52 | 0 |
| Pit mucous | Pit mucous | BAIAP2L2 | 0.52 | 0 |
| Pit mucous | Pit mucous | PON2 | 0.52 | 0 |
| Pit mucous | Pit mucous | MAOA | 0.51 | 0 |
| Pit mucous | Pit mucous | EPS8L2 | 0.51 | 0 |
| Pit mucous | Pit mucous | NPC1L1 | 0.51 | 0 |
| Pit mucous | Pit mucous | ADTRP | 0.5 | 0 |
| Pit mucous | Pit mucous | MMP15 | 0.5 | 0 |
| Pit mucous | Pit mucous | ACOT7 | 0.5 | 0 |
| Pit mucous | Pit mucous | RPS6KA1 | 0.49 | 0 |
| Pit mucous | Pit mucous | POR | 0.49 | 0 |
| Pit mucous | Pit mucous | FUCA1 | 0.48 | 0 |
| Pit mucous | Pit mucous | CLRN3 | 0.48 | 0 |
| Pit mucous | Pit mucous | SLC4A7 | 0.48 | 0 |
| Pit mucous | Pit mucous | DNASE1 | 0.48 | 0 |
| Pit mucous | Pit mucous | AIFM2 | 0.47 | 0 |
| Pit mucous | Pit mucous | BTNL3 | 0.47 | 0 |
| Pit mucous | Pit mucous | SEC23A | 0.47 | 0 |
| Pit mucous | Pit mucous | GALM | 0.46 | 0 |
| Pit mucous | Pit mucous | CORO2A | 0.46 | 0 |
| Pit mucous | Pit mucous | CYP3A4 | 0.46 | 0 |
| Pit mucous | Pit mucous | TRIM15 | 0.46 | 0 |
| Pit mucous | Pit mucous | SLC6A20 | 0.45 | 0 |
| Pit mucous | Pit mucous | SLC27A4 | 0.45 | 0 |
| Pit mucous | Pit mucous | PEX26 | 0.45 | 0 |
| Pit mucous | Pit mucous | LEAP2 | 0.45 | 0 |
| Pit mucous | Pit mucous | TBX10 | 0.45 | 0 |
| Pit mucous | Pit mucous | SLC46A3 | 0.45 | 0 |
| Pit mucous | Pit mucous | UGT2B7 | 0.44 | 0 |
| Pit mucous | Pit mucous | LINC02535 | 0.44 | 0 |
| Pit mucous | Pit mucous | ABHD3 | 0.44 | 0 |
| Pit mucous | Pit mucous | C11orf24 | 0.43 | 0 |
| Pit mucous | Pit mucous | MAF | 0.43 | 0 |
| Pit mucous | Pit mucous | STOM | 0.43 | 0 |
| Pit mucous | Pit mucous | XRCC4 | 0.43 | 0 |
| Pit mucous | Pit mucous | XPNPEP1 | 0.43 | 0 |
| Pit mucous | Pit mucous | SNRK | 0.43 | 0 |
| Pit mucous | Pit mucous | SLC31A1 | 0.42 | 0 |
| Pit mucous | Pit mucous | TBX3 | 0.42 | 0 |
| Pit mucous | Pit mucous | UGT2B17 | 0.42 | 0 |
| Pit mucous | Pit mucous | SLC3A1 | 0.42 | 0 |
| Pit mucous | Pit mucous | SPPL2A | 0.41 | 0 |
| Pit mucous | Pit mucous | ENTPD8 | 0.41 | 0 |
| Pit mucous | Pit mucous | SULT1E1 | 0.41 | 0 |
| Pit mucous | Pit mucous | ACHE | 0.41 | 0 |
| Pit mucous | Pit mucous | CRYBG2 | 0.41 | 0 |
| Pit mucous | Pit mucous | SLC6A8 | 0.41 | 0 |
| Pit mucous | Pit mucous | SHD | 0.41 | 0 |
| Pit mucous | Pit mucous | ACOT8 | 0.41 | 0 |
| Pit mucous | Pit mucous | MYL4 | 0.4 | 0 |
| Pit mucous | Pit mucous | PTPRH | 0.4 | 0 |
| Pit mucous | Pit mucous | NR1H4 | 0.4 | 0 |
| Pit mucous | Pit mucous | RBKS | 0.4 | 0 |
| Pit mucous | Pit mucous | LIPA | 0.39 | 0 |
| Pit mucous | Pit mucous | PDZK1 | 0.39 | 0 |
| Pit mucous | Pit mucous | SLC37A4 | 0.39 | 0 |
| Pit mucous | Pit mucous | MIER3 | 0.39 | 0 |
| Pit mucous | Pit mucous | C4BPB | 0.39 | 0 |
| Pit mucous | Pit mucous | KCNK5 | 0.39 | 0 |
| Pit mucous | Pit mucous | SMLR1 | 0.38 | 0 |
| Pit mucous | Pit mucous | GOSR2 | 0.38 | 0 |
| Pit mucous | Pit mucous | METTL7B | 0.38 | 0 |
| Pit mucous | Pit mucous | CA13 | 0.38 | 0 |
| Pit mucous | Pit mucous | MARVELD3 | 0.38 | 0 |
| Pit mucous | Pit mucous | TMEM92 | 0.37 | 0 |
| Pit mucous | Pit mucous | DHDH | 0.37 | 0 |
| Pit mucous | Pit mucous | OSBPL1A | 0.37 | 0 |
| Pit mucous | Pit mucous | RALGPS2 | 0.37 | 0 |
| Pit mucous | Pit mucous | SSUH2 | 0.37 | 0 |
| Pit mucous | Pit mucous | GUCA2A | 0.37 | 0 |
| Pit mucous | Pit mucous | A1CF | 0.37 | 0 |
| Pit mucous | Pit mucous | NSMF | 0.36 | 0 |
| Pit mucous | Pit mucous | KALRN | 0.36 | 0 |
| Pit mucous | Pit mucous | APPL2 | 0.36 | 0 |
| Pit mucous | Pit mucous | SERPINE2 | 0.36 | 0 |
| Pit mucous | Pit mucous | SLC7A7 | 0.36 | 0 |
| Pit mucous | Pit mucous | MAOB | 0.35 | 0 |
| Pit mucous | Pit mucous | DDX60L | 0.35 | 0 |
| Pit mucous | Pit mucous | GGT1 | 0.35 | 0 |
| Pit mucous | Pit mucous | FOLH1 | 0.35 | 0 |
| Pit mucous | Pit mucous | GUCY2C | 0.35 | 0 |
| Pit mucous | Pit mucous | CYP27A1 | 0.34 | 0 |
| Pit mucous | Pit mucous | TMEM139 | 0.34 | 0 |
| Pit mucous | Pit mucous | TINAG | 0.34 | 0 |
| Pit mucous | Pit mucous | LINC00668 | 0.34 | 0 |
| Pit mucous | Pit mucous | GDPD2 | 0.34 | 0 |
| Pit mucous | Pit mucous | AC021218.1 | 0.34 | 0 |
| Pit mucous | Pit mucous | SLC36A1 | 0.34 | 0 |
| Pit mucous | Pit mucous | PITPNM1 | 0.33 | 0 |
| Pit mucous | Pit mucous | MS4A10 | 0.32 | 0 |
| Pit mucous | Pit mucous | SLC22A18AS | 0.32 | 0 |
| Pit mucous | Pit mucous | ENPP7 | 0.32 | 0 |
| Pit mucous | Pit mucous | CD36 | 0.32 | 0 |
| Pit mucous | Pit mucous | CDX2 | 0.32 | 0 |
| Pit mucous | Pit mucous | EDN3 | 0.31 | 0 |
| Pit mucous | Pit mucous | CYP4F2 | 0.31 | 0 |
| Pit mucous | Pit mucous | B3GNT8 | 0.31 | 0 |
| Pit mucous | Pit mucous | TONSL | 0.31 | 0 |
| Pit mucous | Pit mucous | TRIM36 | 0.31 | 0 |
| Pit mucous | Pit mucous | SLC25A20 | 0.3 | 0 |
| Pit mucous | Pit mucous | AC015917.2 | 0.3 | 0 |
| Pit mucous | Pit mucous | ABCG5 | 0.3 | 0 |
| Pit mucous | Pit mucous | CASP16P | 0.29 | 0 |
| Pit mucous | Pit mucous | PCSK5 | 0.28 | 0 |
| Pit mucous | Pit mucous | SH3D21 | 0.28 | 0 |
| Pit mucous | Pit mucous | BCO1 | 0.28 | 0 |
| Pit mucous | Pit mucous | NAT2 | 0.27 | 0 |
| Pit mucous | Pit mucous | SLC30A10 | 0.27 | 0 |
| Pit mucous | Pit mucous | SLC1A1 | 0.27 | 0 |
| Pit mucous | Pit mucous | AC233992.1 | 0.27 | 0 |
| Pit mucous | Pit mucous | SGK2 | 0.27 | 0 |
| Pit mucous | Pit mucous | ACMSD | 0.26 | 0 |
| Pit mucous | Pit mucous | SEMA6D | 0.25 | 0 |
| Pit mucous | Pit mucous | RPS15 | -0.83 | 0 |
| Pit mucous | Pit mucous | RPL28 | -0.89 | 0 |
| Pit mucous | Pit mucous | RPS11 | -0.9 | 0 |
| Pit mucous | Pit mucous | EEF1A1 | -0.91 | 0 |
| Pit mucous | Pit mucous | RPLP2 | -0.93 | 0 |
| Pit mucous | Pit mucous | RPL19 | -0.95 | 0 |
| Pit mucous | Pit mucous | RPS9 | -0.95 | 0 |
| Pit mucous | Pit mucous | RPL41 | -0.96 | 0 |
| Pit mucous | Pit mucous | RACK1 | -0.98 | 0 |
| Pit mucous | Pit mucous | RPS6 | -1 | 0 |
| Pit mucous | Pit mucous | RPL11 | -1 | 0 |
| Pit mucous | Pit mucous | RPS29 | -1.01 | 0 |
| Pit mucous | Pit mucous | RPLP0 | -1.03 | 0 |
| Pit mucous | Pit mucous | RPS28 | -1.07 | 0 |
| Pit mucous | Pit mucous | RPS14 | -1.09 | 0 |
| Pit mucous | Pit mucous | RPL37A | -1.09 | 0 |
| Pit mucous | Pit mucous | RPL14 | -1.11 | 0 |
| Pit mucous | Pit mucous | RPL21 | -1.12 | 0 |
| Pit mucous | Pit mucous | RPL10 | -1.12 | 0 |
| Pit mucous | Pit mucous | RPLP1 | -1.13 | 0 |
| Pit mucous | Pit mucous | RPS27A | -1.14 | 0 |
| Pit mucous | Pit mucous | RPS19 | -1.17 | 0 |
| Pit mucous | Pit mucous | RPL34 | -1.18 | 0 |
| Pit mucous | Pit mucous | RPL15 | -1.18 | 0 |
| Pit mucous | Pit mucous | RPS24 | -1.18 | 0 |
| Pit mucous | Pit mucous | RPL35 | -1.19 | 0 |
| Pit mucous | Pit mucous | RPL27A | -1.2 | 0 |
| Pit mucous | Pit mucous | RPS3A | -1.21 | 0 |
| Pit mucous | Pit mucous | RPL35A | -1.21 | 0 |
| Pit mucous | Pit mucous | RPS15A | -1.21 | 0 |
| Pit mucous | Pit mucous | RPL39 | -1.22 | 0 |
| Pit mucous | Pit mucous | RPS25 | -1.23 | 0 |
| Pit mucous | Pit mucous | RPL13A | -1.24 | 0 |
| Pit mucous | Pit mucous | RPL36 | -1.24 | 0 |
| Pit mucous | Pit mucous | RPS8 | -1.25 | 0 |
| Pit mucous | Pit mucous | RPL18A | -1.25 | 0 |
| Pit mucous | Pit mucous | RPS4X | -1.26 | 0 |
| Pit mucous | Pit mucous | RPS23 | -1.26 | 0 |
| Pit mucous | Pit mucous | RPL32 | -1.28 | 0 |
| Pit mucous | Pit mucous | RPS3 | -1.29 | 0 |
| Pit mucous | Pit mucous | RPL12 | -1.3 | 0 |
| Pit mucous | Pit mucous | RPL23A | -1.3 | 0 |
| Pit mucous | Pit mucous | RPL10A | -1.3 | 0 |
| Pit mucous | Pit mucous | RPL13 | -1.31 | 0 |
| Pit mucous | Pit mucous | RPS18 | -1.32 | 0 |
| Pit mucous | Pit mucous | RPL3 | -1.37 | 0 |
| Pit mucous | Pit mucous | RPL26 | -1.38 | 0 |
| Pit mucous | Pit mucous | RPS12 | -1.41 | 0 |
| Pit mucous | Pit mucous | RPS27 | -1.44 | 0 |
| Pit mucous | Pit mucous | RPL36A | -1.47 | 0 |
| Pit mucous | Pit mucous | RPL31 | -1.6 | 0 |
| Pit mucous | Pit mucous | PTMA | -1.63 | 0 |
| Pit mucous | Pit mucous | RPS2 | -1.71 | 0 |
| Pit mucous | Pit mucous | MUC5AC | -3.04 | 0 |
| Pit mucous | Pit mucous | RPS10 | -1.16 | 2.00E-306 |
| Pit mucous | Pit mucous | LMO7 | 0.67 | 4.15E-306 |
| Pit mucous | Pit mucous | GPRC5A | 0.75 | 1.56E-303 |
| Pit mucous | Pit mucous | ITPK1 | 0.39 | 1.66E-303 |
| Pit mucous | Pit mucous | RPL9 | -0.91 | 3.94E-303 |
| Pit mucous | Pit mucous | RPS27L | 0.68 | 1.89E-301 |
| Pit mucous | Pit mucous | CALM2 | 0.85 | 2.75E-301 |
| Pit mucous | Pit mucous | TMEM256 | 0.69 | 3.27E-301 |
| Pit mucous | Pit mucous | CISD1 | 0.61 | 5.34E-301 |
| Pit mucous | Pit mucous | GAST | -4.77 | 7.36E-301 |
| Pit mucous | Pit mucous | P4HB | 0.68 | 4.33E-300 |
| Pit mucous | Pit mucous | ADGRG6 | 0.34 | 5.20E-300 |
| Pit mucous | Pit mucous | PGAM1 | 0.61 | 5.43E-300 |
| Pit mucous | Pit mucous | MAX | 0.5 | 2.42E-299 |
| Pit mucous | Pit mucous | MYO1D | 0.36 | 3.03E-297 |
| Pit mucous | Pit mucous | CIAO2B | 0.73 | 4.43E-296 |
| Pit mucous | Pit mucous | RETSAT | 0.43 | 4.88E-296 |
| Pit mucous | Pit mucous | CASP7 | 0.37 | 8.57E-295 |
| Pit mucous | Pit mucous | CD2AP | 0.49 | 1.24E-294 |
| Pit mucous | Pit mucous | TTC38 | 0.36 | 1.35E-294 |
| Pit mucous | Pit mucous | PDZD3 | 0.25 | 4.32E-294 |
| Pit mucous | Pit mucous | CDH1 | 0.64 | 4.47E-294 |
| Pit mucous | Pit mucous | CKMT1B | 0.49 | 3.16E-293 |
| Pit mucous | Pit mucous | GSKIP | 0.5 | 1.98E-292 |
| Pit mucous | Pit mucous | PECR | 0.27 | 2.19E-292 |
| Pit mucous | Pit mucous | IL2RG | 0.46 | 2.15E-291 |
| Pit mucous | Pit mucous | ACOT11 | 0.32 | 5.36E-291 |
| Pit mucous | Pit mucous | STARD4 | 0.33 | 1.66E-290 |
| Pit mucous | Pit mucous | RPS13 | -0.97 | 3.09E-290 |
| Pit mucous | Pit mucous | COX7C | 0.6 | 4.53E-290 |
| Pit mucous | Pit mucous | RPL38 | -1.07 | 4.18E-288 |
| Pit mucous | Pit mucous | CIB1 | 0.7 | 1.24E-286 |
| Pit mucous | Pit mucous | SDHB | 0.54 | 4.04E-286 |
| Pit mucous | Pit mucous | CRAT | 0.3 | 2.95E-285 |
| Pit mucous | Pit mucous | MT-CO1 | 0.53 | 6.92E-285 |
| Pit mucous | Pit mucous | SERINC2 | 0.59 | 2.65E-284 |
| Pit mucous | Pit mucous | UQCR10 | 0.68 | 5.24E-284 |
| Pit mucous | Pit mucous | RPS20 | -1.04 | 6.87E-284 |
| Pit mucous | Pit mucous | MS4A8 | 0.28 | 7.10E-283 |
| Pit mucous | Pit mucous | DYNLT1 | 0.72 | 2.52E-282 |
| Pit mucous | Pit mucous | POMP | 0.72 | 2.65E-282 |
| Pit mucous | Pit mucous | HMOX1 | 0.31 | 5.18E-282 |
| Pit mucous | Pit mucous | CDKN2B | 0.27 | 1.58E-281 |
| Pit mucous | Pit mucous | HAAO | 0.34 | 2.14E-281 |
| Pit mucous | Pit mucous | TFF2 | -3.08 | 8.98E-281 |
| Pit mucous | Pit mucous | LYZ | -2.2 | 1.02E-280 |
| Pit mucous | Pit mucous | RPL37 | -1.19 | 5.54E-280 |
| Pit mucous | Pit mucous | CDCA3 | 0.26 | 6.08E-280 |
| Pit mucous | Pit mucous | MGST2 | 0.77 | 2.28E-279 |
| Pit mucous | Pit mucous | NPNT | 0.27 | 2.51E-279 |
| Pit mucous | Pit mucous | GUCD1 | 0.39 | 1.17E-278 |
| Pit mucous | Pit mucous | MAPK3 | 0.52 | 3.80E-278 |
| Pit mucous | Pit mucous | RPL7A | -0.96 | 4.45E-277 |
| Pit mucous | Pit mucous | CTSD | 0.64 | 2.78E-276 |
| Pit mucous | Pit mucous | WDR1 | 0.57 | 3.94E-276 |
| Pit mucous | Pit mucous | SMPD3 | 0.32 | 9.59E-276 |
| Pit mucous | Pit mucous | DNAJC15 | 0.62 | 1.32E-275 |
| Pit mucous | Pit mucous | GSDMD | 0.53 | 1.98E-275 |
| Pit mucous | Pit mucous | EDF1 | 0.6 | 4.05E-273 |
| Pit mucous | Pit mucous | TRIM31 | 0.46 | 2.50E-272 |
| Pit mucous | Pit mucous | ATP5MPL | 0.63 | 4.20E-272 |
| Pit mucous | Pit mucous | NDUFS2 | 0.52 | 4.58E-272 |
| Pit mucous | Pit mucous | ATP5MD | 0.71 | 1.29E-271 |
| Pit mucous | Pit mucous | RPL4 | -1.28 | 2.03E-271 |
| Pit mucous | Pit mucous | RPSA | -1.09 | 2.72E-271 |
| Pit mucous | Pit mucous | ADM | 0.52 | 5.57E-271 |
| Pit mucous | Pit mucous | RPL27 | -1.01 | 1.17E-270 |
| Pit mucous | Pit mucous | B2M | 0.57 | 2.09E-270 |
| Pit mucous | Pit mucous | CPS1 | 0.38 | 6.43E-270 |
| Pit mucous | Pit mucous | SEC22C | 0.32 | 9.91E-270 |
| Pit mucous | Pit mucous | SLC4A4 | 0.34 | 1.10E-269 |
| Pit mucous | Pit mucous | RTCB | 0.45 | 1.64E-269 |
| Pit mucous | Pit mucous | ARHGAP5 | 0.44 | 5.41E-266 |
| Pit mucous | Pit mucous | PRDM1 | 0.35 | 8.31E-266 |
| Pit mucous | Pit mucous | C17orf67 | 0.27 | 1.01E-265 |
| Pit mucous | Pit mucous | GNB1 | 0.48 | 5.22E-265 |
| Pit mucous | Pit mucous | COX8A | 0.6 | 8.00E-265 |
| Pit mucous | Pit mucous | ATP5F1B | 0.67 | 2.70E-264 |
| Pit mucous | Pit mucous | SLC25A37 | 0.51 | 6.97E-264 |
| Pit mucous | Pit mucous | MAPK13 | 0.44 | 1.19E-263 |
| Pit mucous | Pit mucous | PFKFB2 | 0.32 | 1.64E-263 |
| Pit mucous | Pit mucous | EMB | 0.29 | 9.28E-263 |
| Pit mucous | Pit mucous | MYL12A | 0.64 | 1.63E-262 |
| Pit mucous | Pit mucous | RAB8A | 0.41 | 3.16E-262 |
| Pit mucous | Pit mucous | CNDP2 | 0.51 | 3.69E-262 |
| Pit mucous | Pit mucous | NDUFB3 | 0.65 | 4.98E-262 |
| Pit mucous | Pit mucous | CYP4F12 | 0.31 | 4.72E-261 |
| Pit mucous | Pit mucous | TFPI | 0.31 | 6.23E-261 |
| Pit mucous | Pit mucous | NDUFA6 | 0.64 | 1.68E-260 |
| Pit mucous | Pit mucous | SERPINA1 | 0.65 | 2.28E-260 |
| Pit mucous | Pit mucous | SLC35C1 | 0.34 | 2.89E-260 |
| Pit mucous | Pit mucous | CHMP1B | 0.52 | 8.28E-260 |
| Pit mucous | Pit mucous | DMBT1 | 0.57 | 1.64E-259 |
| Pit mucous | Pit mucous | RPL23 | -1.22 | 3.37E-259 |
| Pit mucous | Pit mucous | AHCYL1 | 0.5 | 4.09E-258 |
| Pit mucous | Pit mucous | SMPDL3A | 0.36 | 1.07E-257 |
| Pit mucous | Pit mucous | RPL6 | -1.09 | 3.56E-257 |
| Pit mucous | Pit mucous | DUSP6 | 0.41 | 3.71E-257 |
| Pit mucous | Pit mucous | DDT | 0.69 | 4.27E-257 |
| Pit mucous | Pit mucous | MTMR11 | 0.29 | 5.68E-257 |
| Pit mucous | Pit mucous | TP53I3 | 0.4 | 3.29E-256 |
| Pit mucous | Pit mucous | CDC42EP2 | 0.38 | 7.10E-256 |
| Pit mucous | Pit mucous | ECHS1 | 0.58 | 2.79E-255 |
| Pit mucous | Pit mucous | RNF141 | 0.32 | 3.03E-255 |
| Pit mucous | Pit mucous | LRRC1 | 0.33 | 6.26E-255 |
| Pit mucous | Pit mucous | TJP3 | 0.34 | 9.02E-255 |
| Pit mucous | Pit mucous | MFSD2A | 0.39 | 1.27E-254 |
| Pit mucous | Pit mucous | FAM214B | 0.27 | 4.38E-254 |
| Pit mucous | Pit mucous | PGC | -3.63 | 1.06E-253 |
| Pit mucous | Pit mucous | HAS3 | 0.29 | 1.45E-253 |
| Pit mucous | Pit mucous | CAP1 | 0.58 | 1.34E-251 |
| Pit mucous | Pit mucous | CD55 | 0.62 | 2.03E-251 |
| Pit mucous | Pit mucous | RPL18 | -0.77 | 2.36E-251 |
| Pit mucous | Pit mucous | ATP1A1 | 0.63 | 3.08E-251 |
| Pit mucous | Pit mucous | TXN | 0.81 | 5.06E-251 |
| Pit mucous | Pit mucous | MPST | 0.64 | 1.04E-250 |
| Pit mucous | Pit mucous | SH3BGRL3 | 0.63 | 1.16E-250 |
| Pit mucous | Pit mucous | UQCRC1 | 0.6 | 4.02E-250 |
| Pit mucous | Pit mucous | TFG | 0.48 | 5.82E-250 |
| Pit mucous | Pit mucous | GSDMB | 0.32 | 6.54E-249 |
| Pit mucous | Pit mucous | FLVCR1 | 0.31 | 1.62E-248 |
| Pit mucous | Pit mucous | DPEP1 | 0.44 | 6.01E-248 |
| Pit mucous | Pit mucous | SLC20A2 | 0.31 | 8.63E-248 |
| Pit mucous | Pit mucous | RPL17 | -1.38 | 2.48E-246 |
| Pit mucous | Pit mucous | NDUFS3 | 0.53 | 5.56E-246 |
| Pit mucous | Pit mucous | USH1C | 0.43 | 2.41E-245 |
| Pit mucous | Pit mucous | C4orf3 | 0.61 | 1.22E-244 |
| Pit mucous | Pit mucous | CYP2C18 | 0.31 | 4.64E-244 |
| Pit mucous | Pit mucous | VPS28 | 0.57 | 7.49E-244 |
| Pit mucous | Pit mucous | ITM2B | 0.54 | 9.92E-244 |
| Pit mucous | Pit mucous | PRXL2A | 0.45 | 1.11E-243 |
| Pit mucous | Pit mucous | CHMP2A | 0.58 | 1.24E-243 |
| Pit mucous | Pit mucous | RPL22 | -1.01 | 1.17E-242 |
| Pit mucous | Pit mucous | ACAA2 | 0.44 | 5.47E-242 |
| Pit mucous | Pit mucous | SNX9 | 0.41 | 6.10E-242 |
| Pit mucous | Pit mucous | HIST1H2AC | 0.39 | 1.08E-240 |
| Pit mucous | Pit mucous | CAPN1 | 0.49 | 1.35E-240 |
| Pit mucous | Pit mucous | ADAM9 | 0.43 | 2.27E-240 |
| Pit mucous | Pit mucous | MGAT4A | 0.29 | 2.44E-239 |
| Pit mucous | Pit mucous | RPL5 | -1.11 | 3.78E-238 |
| Pit mucous | Pit mucous | SULT1A3 | 0.25 | 1.10E-237 |
| Pit mucous | Pit mucous | HNRNPA1 | -1.08 | 6.99E-237 |
| Pit mucous | Pit mucous | CYC1 | 0.69 | 7.00E-237 |
| Pit mucous | Pit mucous | IFRD1 | 0.52 | 7.66E-237 |
| Pit mucous | Pit mucous | FAM234A | 0.36 | 2.59E-236 |
| Pit mucous | Pit mucous | UQCRFS1 | 0.62 | 1.31E-235 |
| Pit mucous | Pit mucous | DHRS4L2 | 0.43 | 4.86E-233 |
| Pit mucous | Pit mucous | IFT20 | 0.49 | 7.73E-233 |
| Pit mucous | Pit mucous | PLCD3 | 0.36 | 1.52E-232 |
| Pit mucous | Pit mucous | UQCRC2 | 0.54 | 2.67E-232 |
| Pit mucous | Pit mucous | CYCS | 0.61 | 3.43E-231 |
| Pit mucous | Pit mucous | PICALM | 0.34 | 7.16E-231 |
| Pit mucous | Pit mucous | ESRRA | 0.37 | 1.24E-230 |
| Pit mucous | Pit mucous | PHYH | 0.35 | 9.87E-230 |
| Pit mucous | Pit mucous | RAB17 | 0.34 | 1.19E-229 |
| Pit mucous | Pit mucous | ZFAND2A | 0.37 | 1.89E-229 |
| Pit mucous | Pit mucous | FCGRT | 0.59 | 4.62E-229 |
| Pit mucous | Pit mucous | MYO5B | 0.39 | 8.57E-229 |
| Pit mucous | Pit mucous | LACTB2 | 0.33 | 2.37E-228 |
| Pit mucous | Pit mucous | IDH3A | 0.31 | 5.72E-228 |
| Pit mucous | Pit mucous | OCIAD2 | 0.64 | 1.62E-227 |
| Pit mucous | Pit mucous | CHMP5 | 0.5 | 2.03E-227 |
| Pit mucous | Pit mucous | PCYT1A | 0.26 | 5.19E-227 |
| Pit mucous | Pit mucous | MAP2K2 | 0.42 | 5.48E-227 |
| Pit mucous | Pit mucous | TMPRSS2 | 0.5 | 6.96E-227 |
| Pit mucous | Pit mucous | DNPEP | 0.39 | 2.34E-226 |
| Pit mucous | Pit mucous | FXYD3 | 0.58 | 2.38E-226 |
| Pit mucous | Pit mucous | MDK | 0.55 | 5.76E-226 |
| Pit mucous | Pit mucous | MUC1 | -1.64 | 6.37E-226 |
| Pit mucous | Pit mucous | ANXA11 | 0.56 | 1.33E-225 |
| Pit mucous | Pit mucous | UGP2 | 0.44 | 7.05E-225 |
| Pit mucous | Pit mucous | ABHD12 | 0.29 | 7.15E-225 |
| Pit mucous | Pit mucous | KRT19 | 0.65 | 4.92E-224 |
| Pit mucous | Pit mucous | NDRG1 | 0.48 | 3.06E-223 |
| Pit mucous | Pit mucous | IDNK | 0.27 | 3.79E-222 |
| Pit mucous | Pit mucous | WSB2 | 0.32 | 6.97E-222 |
| Pit mucous | Pit mucous | IBTK | 0.39 | 7.49E-222 |
| Pit mucous | Pit mucous | CTNNB1 | 0.45 | 8.00E-222 |
| Pit mucous | Pit mucous | GNA13 | 0.32 | 1.95E-221 |
| Pit mucous | Pit mucous | NPM1 | -1.28 | 2.70E-221 |
| Pit mucous | Pit mucous | NBEAL1 | -1.22 | 5.18E-221 |
| Pit mucous | Pit mucous | MT-ATP6 | 0.43 | 7.72E-221 |
| Pit mucous | Pit mucous | RNPEP | 0.36 | 1.39E-220 |
| Pit mucous | Pit mucous | CLDN23 | 0.42 | 2.54E-220 |
| Pit mucous | Pit mucous | RPS5 | -0.77 | 7.07E-220 |
| Pit mucous | Pit mucous | ETS2 | 0.53 | 2.01E-219 |
| Pit mucous | Pit mucous | CDX1 | 0.27 | 4.58E-219 |
| Pit mucous | Pit mucous | COX5A | 0.57 | 8.21E-219 |
| Pit mucous | Pit mucous | C6orf222 | 0.33 | 1.44E-218 |
| Pit mucous | Pit mucous | VDAC1 | 0.58 | 2.19E-218 |
| Pit mucous | Pit mucous | TMEM164 | 0.26 | 2.91E-218 |
| Pit mucous | Pit mucous | CDKN1A | 0.5 | 1.28E-216 |
| Pit mucous | Pit mucous | ETFB | 0.55 | 1.55E-216 |
| Pit mucous | Pit mucous | ATP5F1C | 0.59 | 2.93E-215 |
| Pit mucous | Pit mucous | DSG2 | 0.4 | 3.64E-215 |
| Pit mucous | Pit mucous | MOB3B | 0.28 | 2.94E-214 |
| Pit mucous | Pit mucous | DUSP5 | 0.34 | 1.23E-213 |
| Pit mucous | Pit mucous | DHRS9 | 0.27 | 4.55E-213 |
| Pit mucous | Pit mucous | GALC | 0.25 | 5.41E-212 |
| Pit mucous | Pit mucous | FAM32A | 0.42 | 1.48E-211 |
| Pit mucous | Pit mucous | APOL6 | 0.29 | 3.50E-211 |
| Pit mucous | Pit mucous | PPIC | 0.43 | 9.12E-211 |
| Pit mucous | Pit mucous | NDUFC2 | 0.64 | 5.28E-210 |
| Pit mucous | Pit mucous | ATP6V1D | 0.38 | 1.01E-209 |
| Pit mucous | Pit mucous | TPI1 | 0.58 | 1.34E-209 |
| Pit mucous | Pit mucous | RPS21 | -0.93 | 3.03E-209 |
| Pit mucous | Pit mucous | RCAN1 | 0.29 | 4.10E-209 |
| Pit mucous | Pit mucous | NDUFS7 | 0.51 | 1.46E-208 |
| Pit mucous | Pit mucous | LRP10 | 0.45 | 3.40E-208 |
| Pit mucous | Pit mucous | RIDA | 0.31 | 4.81E-208 |
| Pit mucous | Pit mucous | MPC1 | 0.43 | 8.00E-207 |
| Pit mucous | Pit mucous | SPINT1 | 0.47 | 2.34E-206 |
| Pit mucous | Pit mucous | TMBIM6 | 0.5 | 8.38E-206 |
| Pit mucous | Pit mucous | TMEM53 | 0.25 | 1.08E-205 |
| Pit mucous | Pit mucous | CYP3A5 | 0.49 | 7.67E-205 |
| Pit mucous | Pit mucous | MT-CYB | 0.34 | 2.08E-204 |
| Pit mucous | Pit mucous | GSTM4 | 0.4 | 1.20E-203 |
| Pit mucous | Pit mucous | RPL24 | -0.78 | 2.22E-203 |
| Pit mucous | Pit mucous | RPL29 | -0.71 | 2.84E-203 |
| Pit mucous | Pit mucous | ZBTB7B | 0.27 | 1.19E-202 |
| Pit mucous | Pit mucous | CKMT1A | 0.34 | 2.17E-201 |
| Pit mucous | Pit mucous | MAL2 | 0.58 | 4.24E-201 |
| Pit mucous | Pit mucous | NDUFA1 | 0.48 | 9.67E-201 |
| Pit mucous | Pit mucous | CHMP4B | 0.53 | 1.16E-200 |
| Pit mucous | Pit mucous | CXADR | 0.45 | 1.41E-200 |
| Pit mucous | Pit mucous | CTSS | 0.42 | 2.85E-200 |
| Pit mucous | Pit mucous | PDCD10 | 0.38 | 1.11E-199 |
| Pit mucous | Pit mucous | GAPDH | 0.49 | 4.11E-199 |
| Pit mucous | Pit mucous | AP1S1 | 0.45 | 1.30E-198 |
| Pit mucous | Pit mucous | HOXB7 | 0.27 | 1.39E-198 |
| Pit mucous | Pit mucous | PSMD1 | 0.35 | 2.22E-198 |
| Pit mucous | Pit mucous | CYP2S1 | 0.33 | 1.58E-197 |
| Pit mucous | Pit mucous | FAM177A1 | 0.47 | 1.17E-196 |
| Pit mucous | Pit mucous | VSIR | 0.31 | 1.47E-195 |
| Pit mucous | Pit mucous | SLC35B1 | 0.35 | 1.62E-195 |
| Pit mucous | Pit mucous | COX6A1P2 | 0.52 | 3.38E-195 |
| Pit mucous | Pit mucous | FBLIM1 | 0.31 | 3.60E-195 |
| Pit mucous | Pit mucous | MT1X | -1.6 | 4.42E-195 |
| Pit mucous | Pit mucous | RPL30 | -0.81 | 2.63E-194 |
| Pit mucous | Pit mucous | FABP5 | -1.5 | 1.17E-193 |
| Pit mucous | Pit mucous | AK1 | 0.45 | 2.02E-193 |
| Pit mucous | Pit mucous | FMO5 | 0.26 | 7.95E-193 |
| Pit mucous | Pit mucous | DERA | 0.26 | 1.63E-192 |
| Pit mucous | Pit mucous | PBLD | 0.28 | 3.34E-192 |
| Pit mucous | Pit mucous | IDH1 | 0.47 | 4.52E-192 |
| Pit mucous | Pit mucous | NQO2 | 0.28 | 6.92E-192 |
| Pit mucous | Pit mucous | YWHAZ | 0.58 | 1.57E-191 |
| Pit mucous | Pit mucous | EPB41L4B | 0.26 | 1.02E-189 |
| Pit mucous | Pit mucous | PLA2G16 | 0.38 | 1.97E-189 |
| Pit mucous | Pit mucous | LASP1 | 0.37 | 1.99E-189 |
| Pit mucous | Pit mucous | NDFIP2 | 0.34 | 3.09E-189 |
| Pit mucous | Pit mucous | MT-CO3 | 0.31 | 3.64E-189 |
| Pit mucous | Pit mucous | EPHX2 | 0.32 | 4.04E-189 |
| Pit mucous | Pit mucous | AK4 | 0.33 | 4.08E-189 |
| Pit mucous | Pit mucous | ANXA3 | 0.38 | 7.43E-189 |
| Pit mucous | Pit mucous | VILL | 0.26 | 9.47E-189 |
| Pit mucous | Pit mucous | RALB | 0.28 | 4.13E-188 |
| Pit mucous | Pit mucous | FOS | -1.83 | 9.70E-188 |
| Pit mucous | Pit mucous | ARFGAP3 | 0.32 | 2.64E-187 |
| Pit mucous | Pit mucous | TXNRD1 | 0.41 | 2.95E-187 |
| Pit mucous | Pit mucous | ST14 | 0.4 | 5.09E-187 |
| Pit mucous | Pit mucous | FAM83H | 0.28 | 2.30E-186 |
| Pit mucous | Pit mucous | LAMP1 | 0.38 | 4.24E-186 |
| Pit mucous | Pit mucous | ITGB4 | 0.35 | 8.39E-186 |
| Pit mucous | Pit mucous | DOK4 | 0.27 | 1.55E-185 |
| Pit mucous | Pit mucous | ARHGEF35 | 0.31 | 2.06E-185 |
| Pit mucous | Pit mucous | PRKAG2 | 0.25 | 3.95E-185 |
| Pit mucous | Pit mucous | TSPAN3 | 0.46 | 4.49E-184 |
| Pit mucous | Pit mucous | PANK3 | 0.29 | 9.97E-184 |
| Pit mucous | Pit mucous | CALM3 | 0.47 | 1.73E-183 |
| Pit mucous | Pit mucous | CAPZA2 | 0.47 | 6.17E-183 |
| Pit mucous | Pit mucous | UBE2L3 | 0.44 | 7.01E-183 |
| Pit mucous | Pit mucous | OPTN | 0.28 | 8.95E-182 |
| Pit mucous | Pit mucous | AGR2 | -1.3 | 1.14E-181 |
| Pit mucous | Pit mucous | TMEM167A | 0.41 | 1.56E-181 |
| Pit mucous | Pit mucous | TFF1 | -2.56 | 5.02E-181 |
| Pit mucous | Pit mucous | ERRFI1 | 0.44 | 5.80E-181 |
| Pit mucous | Pit mucous | ARL4A | 0.42 | 8.10E-181 |
| Pit mucous | Pit mucous | ATP5PO | 0.55 | 1.59E-180 |
| Pit mucous | Pit mucous | DAD1 | 0.61 | 5.82E-180 |
| Pit mucous | Pit mucous | SPRED2 | 0.28 | 6.51E-180 |
| Pit mucous | Pit mucous | CMPK1 | 0.46 | 9.15E-180 |
| Pit mucous | Pit mucous | RAB6A | 0.31 | 2.81E-178 |
| Pit mucous | Pit mucous | SDHC | 0.42 | 4.39E-178 |
| Pit mucous | Pit mucous | CMBL | 0.42 | 1.12E-177 |
| Pit mucous | Pit mucous | RAB5C | 0.4 | 1.82E-177 |
| Pit mucous | Pit mucous | HLA-A | 0.53 | 2.00E-177 |
| Pit mucous | Pit mucous | TOM1L1 | 0.3 | 2.06E-177 |
| Pit mucous | Pit mucous | CHKA | 0.28 | 2.93E-177 |
| Pit mucous | Pit mucous | HSP90AB1 | -0.93 | 3.57E-177 |
| Pit mucous | Pit mucous | FAM3B | 0.47 | 4.08E-177 |
| Pit mucous | Pit mucous | NDUFA4 | 0.48 | 7.39E-177 |
| Pit mucous | Pit mucous | FHL2 | 0.48 | 8.45E-177 |
| Pit mucous | Pit mucous | FAM49B | 0.29 | 1.32E-176 |
| Pit mucous | Pit mucous | RTCA | 0.26 | 1.65E-176 |
| Pit mucous | Pit mucous | PRDX6 | 0.58 | 2.41E-176 |
| Pit mucous | Pit mucous | ATP5PB | 0.48 | 4.54E-176 |
| Pit mucous | Pit mucous | TSG101 | 0.32 | 1.34E-175 |
| Pit mucous | Pit mucous | PDLIM5 | 0.33 | 6.15E-175 |
| Pit mucous | Pit mucous | STK24 | 0.3 | 1.04E-174 |
| Pit mucous | Pit mucous | ABRACL | 0.37 | 8.14E-174 |
| Pit mucous | Pit mucous | IL1RN | 0.34 | 4.87E-173 |
| Pit mucous | Pit mucous | ITGA6 | 0.41 | 1.03E-171 |
| Pit mucous | Pit mucous | ORMDL2 | 0.33 | 1.08E-171 |
| Pit mucous | Pit mucous | SLC35A3 | 0.34 | 1.66E-171 |
| Pit mucous | Pit mucous | ITGA2 | 0.31 | 7.56E-170 |
| Pit mucous | Pit mucous | ATP5PD | 0.49 | 4.25E-169 |
| Pit mucous | Pit mucous | GPX4 | 0.49 | 5.54E-169 |
| Pit mucous | Pit mucous | LETM1 | 0.25 | 6.83E-169 |
| Pit mucous | Pit mucous | SMCO4 | 0.34 | 7.82E-169 |
| Pit mucous | Pit mucous | SBDS | 0.46 | 4.86E-168 |
| Pit mucous | Pit mucous | FAM84A | 0.27 | 5.77E-168 |
| Pit mucous | Pit mucous | APLP2 | 0.38 | 7.40E-168 |
| Pit mucous | Pit mucous | CD9 | 0.5 | 1.94E-167 |
| Pit mucous | Pit mucous | ARPC5 | 0.4 | 2.20E-167 |
| Pit mucous | Pit mucous | HLA-F | 0.33 | 4.63E-167 |
| Pit mucous | Pit mucous | PTP4A1 | 0.38 | 1.09E-166 |
| Pit mucous | Pit mucous | BDH2 | 0.29 | 2.85E-166 |
| Pit mucous | Pit mucous | STXBP2 | 0.3 | 4.16E-166 |
| Pit mucous | Pit mucous | IER3 | -1.28 | 1.06E-165 |
| Pit mucous | Pit mucous | RPS7 | -0.8 | 1.20E-165 |
| Pit mucous | Pit mucous | TGOLN2 | 0.33 | 4.51E-165 |
| Pit mucous | Pit mucous | GIPC2 | 0.26 | 5.35E-165 |
| Pit mucous | Pit mucous | PIM1 | 0.31 | 1.07E-164 |
| Pit mucous | Pit mucous | SLC25A24 | 0.26 | 1.48E-164 |
| Pit mucous | Pit mucous | NDUFA9 | 0.38 | 3.92E-164 |
| Pit mucous | Pit mucous | PGRMC2 | 0.32 | 9.22E-164 |
| Pit mucous | Pit mucous | GPX2 | -1.26 | 3.69E-163 |
| Pit mucous | Pit mucous | PRDX1 | 0.52 | 5.54E-163 |
| Pit mucous | Pit mucous | C6orf141 | 0.26 | 9.56E-163 |
| Pit mucous | Pit mucous | GKN2 | -2.88 | 3.72E-162 |
| Pit mucous | Pit mucous | UBA52 | -0.61 | 1.55E-161 |
| Pit mucous | Pit mucous | SEC13 | 0.35 | 1.13E-160 |
| Pit mucous | Pit mucous | CHMP2B | 0.33 | 4.63E-160 |
| Pit mucous | Pit mucous | MRPL35 | 0.26 | 8.52E-159 |
| Pit mucous | Pit mucous | STARD10 | -1.05 | 8.74E-159 |
| Pit mucous | Pit mucous | SUCLA2 | 0.26 | 1.37E-158 |
| Pit mucous | Pit mucous | CDS1 | 0.27 | 3.23E-158 |
| Pit mucous | Pit mucous | MLN | -0.88 | 2.53E-157 |
| Pit mucous | Pit mucous | CTNNA1 | 0.36 | 3.47E-157 |
| Pit mucous | Pit mucous | ACTN4 | 0.43 | 4.82E-157 |
| Pit mucous | Pit mucous | NBL1 | 0.29 | 7.60E-157 |
| Pit mucous | Pit mucous | CCDC25 | 0.31 | 1.23E-156 |
| Pit mucous | Pit mucous | EPS8 | 0.32 | 1.23E-156 |
| Pit mucous | Pit mucous | MARCKSL1 | -1.05 | 2.96E-156 |
| Pit mucous | Pit mucous | SPINT2 | 0.47 | 5.48E-156 |
| Pit mucous | Pit mucous | ACADVL | 0.42 | 9.15E-156 |
| Pit mucous | Pit mucous | EPS8L3 | 0.28 | 9.99E-156 |
| Pit mucous | Pit mucous | GLO1 | 0.34 | 3.52E-153 |
| Pit mucous | Pit mucous | ARPC2 | 0.45 | 8.89E-153 |
| Pit mucous | Pit mucous | ATP1B1 | 0.49 | 8.89E-153 |
| Pit mucous | Pit mucous | RNF114 | 0.33 | 1.43E-152 |
| Pit mucous | Pit mucous | RER1 | 0.4 | 2.69E-152 |
| Pit mucous | Pit mucous | WASL | 0.27 | 1.30E-151 |
| Pit mucous | Pit mucous | MGAT4B | 0.3 | 1.05E-150 |
| Pit mucous | Pit mucous | UQCRB | 0.51 | 3.05E-150 |
| Pit mucous | Pit mucous | PDZD11 | 0.27 | 4.80E-150 |
| Pit mucous | Pit mucous | PPP1CC | 0.38 | 5.60E-150 |
| Pit mucous | Pit mucous | EPN1 | 0.32 | 5.73E-150 |
| Pit mucous | Pit mucous | ATP5F1A | 0.47 | 7.88E-150 |
| Pit mucous | Pit mucous | PAFAH1B1 | 0.28 | 1.05E-149 |
| Pit mucous | Pit mucous | NOP53 | -0.97 | 1.17E-149 |
| Pit mucous | Pit mucous | CYB5A | 0.49 | 1.22E-149 |
| Pit mucous | Pit mucous | MIF | -0.94 | 1.31E-148 |
| Pit mucous | Pit mucous | TST | 0.3 | 1.42E-148 |
| Pit mucous | Pit mucous | S100A11 | 0.46 | 1.47E-148 |
| Pit mucous | Pit mucous | DPH3 | 0.25 | 4.75E-148 |
| Pit mucous | Pit mucous | GLUL | -1.03 | 6.46E-148 |
| Pit mucous | Pit mucous | SELENOS | 0.42 | 1.36E-147 |
| Pit mucous | Pit mucous | UBE2A | 0.28 | 1.86E-147 |
| Pit mucous | Pit mucous | NACA | -0.75 | 3.88E-147 |
| Pit mucous | Pit mucous | AKR7A2 | 0.31 | 7.57E-146 |
| Pit mucous | Pit mucous | RAB1A | 0.41 | 1.09E-145 |
| Pit mucous | Pit mucous | SCCPDH | 0.29 | 1.62E-145 |
| Pit mucous | Pit mucous | CAST | 0.34 | 4.17E-145 |
| Pit mucous | Pit mucous | TOP1 | 0.31 | 6.32E-145 |
| Pit mucous | Pit mucous | ROMO1 | 0.43 | 9.25E-145 |
| Pit mucous | Pit mucous | KIF1C | 0.27 | 1.36E-144 |
| Pit mucous | Pit mucous | FOSL1 | 0.29 | 1.65E-144 |
| Pit mucous | Pit mucous | RFK | 0.26 | 1.87E-144 |
| Pit mucous | Pit mucous | ELOVL1 | 0.29 | 9.45E-144 |
| Pit mucous | Pit mucous | GKN1 | -3.82 | 1.21E-143 |
| Pit mucous | Pit mucous | TMCO1 | 0.39 | 2.57E-143 |
| Pit mucous | Pit mucous | BAX | 0.4 | 6.25E-143 |
| Pit mucous | Pit mucous | RPS16 | -0.57 | 1.02E-142 |
| Pit mucous | Pit mucous | ALDOA | 0.47 | 1.29E-142 |
| Pit mucous | Pit mucous | FTH1 | 0.38 | 1.30E-142 |
| Pit mucous | Pit mucous | SDC1 | 0.28 | 2.17E-142 |
| Pit mucous | Pit mucous | MRPS36 | 0.32 | 2.88E-142 |
| Pit mucous | Pit mucous | TSPAN1 | 0.43 | 4.04E-142 |
| Pit mucous | Pit mucous | CAPG | 0.35 | 7.14E-142 |
| Pit mucous | Pit mucous | ATOX1 | 0.39 | 1.24E-141 |
| Pit mucous | Pit mucous | GSTK1 | 0.42 | 1.55E-141 |
| Pit mucous | Pit mucous | PERP | 0.46 | 2.37E-141 |
| Pit mucous | Pit mucous | RAP1A | 0.29 | 2.54E-141 |
| Pit mucous | Pit mucous | NUCB1 | 0.33 | 3.39E-141 |
| Pit mucous | Pit mucous | ARPC3 | 0.45 | 5.42E-141 |
| Pit mucous | Pit mucous | FOXQ1 | -1.06 | 4.10E-140 |
| Pit mucous | Pit mucous | RNF181 | 0.32 | 8.76E-140 |
| Pit mucous | Pit mucous | ATP5IF1 | 0.42 | 1.47E-139 |
| Pit mucous | Pit mucous | FAM102A | 0.26 | 7.19E-139 |
| Pit mucous | Pit mucous | NECTIN2 | 0.37 | 1.07E-138 |
| Pit mucous | Pit mucous | ARL5B | 0.27 | 1.75E-138 |
| Pit mucous | Pit mucous | CXCL17 | -1.13 | 8.16E-138 |
| Pit mucous | Pit mucous | PFDN5 | -0.63 | 8.40E-138 |
| Pit mucous | Pit mucous | CXCL3 | -1.62 | 9.22E-138 |
| Pit mucous | Pit mucous | EPSTI1 | 0.28 | 2.75E-137 |
| Pit mucous | Pit mucous | CBLC | 0.26 | 6.33E-137 |
| Pit mucous | Pit mucous | CORO1B | 0.32 | 1.26E-136 |
| Pit mucous | Pit mucous | PSME1 | 0.43 | 1.52E-136 |
| Pit mucous | Pit mucous | GNG5 | 0.42 | 3.67E-136 |
| Pit mucous | Pit mucous | PSMA7 | 0.47 | 3.88E-136 |
| Pit mucous | Pit mucous | NT5C3A | 0.31 | 7.39E-136 |
| Pit mucous | Pit mucous | TMBIM1 | 0.31 | 1.80E-135 |
| Pit mucous | Pit mucous | ATP5MC2 | -0.7 | 2.67E-135 |
| Pit mucous | Pit mucous | HADH | 0.27 | 4.26E-135 |
| Pit mucous | Pit mucous | DNAJB1 | -1.21 | 2.44E-134 |
| Pit mucous | Pit mucous | CRIP1 | 0.41 | 4.15E-134 |
| Pit mucous | Pit mucous | RBCK1 | 0.32 | 1.03E-133 |
| Pit mucous | Pit mucous | PGK1 | 0.37 | 1.38E-133 |
| Pit mucous | Pit mucous | PYCARD | 0.43 | 3.25E-133 |
| Pit mucous | Pit mucous | AK2 | 0.32 | 4.33E-133 |
| Pit mucous | Pit mucous | HMGN2 | -1.05 | 4.42E-133 |
| Pit mucous | Pit mucous | PTPRF | 0.26 | 1.11E-131 |
| Pit mucous | Pit mucous | BLOC1S1 | 0.36 | 2.01E-131 |
| Pit mucous | Pit mucous | TMEM238L | 0.27 | 3.95E-131 |
| Pit mucous | Pit mucous | SCD | 0.32 | 1.63E-130 |
| Pit mucous | Pit mucous | TACSTD2 | -1.16 | 1.90E-130 |
| Pit mucous | Pit mucous | ARL14 | 0.32 | 2.07E-130 |
| Pit mucous | Pit mucous | RAB5IF | 0.35 | 2.28E-130 |
| Pit mucous | Pit mucous | ALDH3A2 | 0.26 | 3.04E-130 |
| Pit mucous | Pit mucous | FTL | 0.43 | 4.06E-130 |
| Pit mucous | Pit mucous | CTSB | 0.27 | 1.03E-129 |
| Pit mucous | Pit mucous | TPM1 | 0.35 | 1.16E-129 |
| Pit mucous | Pit mucous | YWHAB | 0.39 | 5.67E-129 |
| Pit mucous | Pit mucous | UQCRH | 0.42 | 1.42E-127 |
| Pit mucous | Pit mucous | TMEM106C | 0.25 | 1.46E-127 |
| Pit mucous | Pit mucous | MT1G | -1.49 | 3.16E-127 |
| Pit mucous | Pit mucous | MAP1LC3B | 0.33 | 5.34E-127 |
| Pit mucous | Pit mucous | MYH9 | 0.27 | 5.45E-127 |
| Pit mucous | Pit mucous | HSPA5 | 0.46 | 8.84E-127 |
| Pit mucous | Pit mucous | PPDPF | -0.71 | 3.44E-126 |
| Pit mucous | Pit mucous | F11R | 0.27 | 6.83E-126 |
| Pit mucous | Pit mucous | JUNB | -1.51 | 1.23E-124 |
| Pit mucous | Pit mucous | CAPZA1 | 0.31 | 1.35E-124 |
| Pit mucous | Pit mucous | HLA-C | 0.32 | 1.91E-124 |
| Pit mucous | Pit mucous | GMDS | 0.31 | 2.05E-124 |
| Pit mucous | Pit mucous | TMSB4X | -0.93 | 2.37E-124 |
| Pit mucous | Pit mucous | AKR1C3 | 0.4 | 2.86E-124 |
| Pit mucous | Pit mucous | MRPL13 | 0.3 | 7.19E-124 |
| Pit mucous | Pit mucous | ITPRID2 | 0.26 | 3.79E-122 |
| Pit mucous | Pit mucous | SH3GLB1 | 0.25 | 5.86E-122 |
| Pit mucous | Pit mucous | AURKAIP1 | 0.38 | 1.17E-121 |
| Pit mucous | Pit mucous | RAB11A | 0.36 | 3.01E-121 |
| Pit mucous | Pit mucous | PNP | 0.26 | 3.68E-121 |
| Pit mucous | Pit mucous | ARHGDIA | 0.32 | 4.79E-121 |
| Pit mucous | Pit mucous | CLCN3 | 0.27 | 8.15E-121 |
| Pit mucous | Pit mucous | EEF1D | -0.54 | 1.01E-119 |
| Pit mucous | Pit mucous | ASL | 0.27 | 2.86E-119 |
| Pit mucous | Pit mucous | RNF5 | 0.26 | 2.81E-118 |
| Pit mucous | Pit mucous | CEBPG | 0.26 | 4.00E-118 |
| Pit mucous | Pit mucous | ALDH2 | 0.31 | 5.70E-118 |
| Pit mucous | Pit mucous | EGR1 | -1.2 | 5.72E-118 |
| Pit mucous | Pit mucous | SPATS2L | 0.29 | 2.53E-117 |
| Pit mucous | Pit mucous | FBXW5 | 0.28 | 1.21E-116 |
| Pit mucous | Pit mucous | MRPL41 | 0.36 | 3.02E-116 |
| Pit mucous | Pit mucous | H2AFJ | 0.34 | 3.24E-116 |
| Pit mucous | Pit mucous | RAB10 | 0.3 | 2.02E-115 |
| Pit mucous | Pit mucous | FAU | -0.41 | 3.53E-115 |
| Pit mucous | Pit mucous | ACTR3 | 0.3 | 7.74E-115 |
| Pit mucous | Pit mucous | NAP1L1 | -0.72 | 4.79E-114 |
| Pit mucous | Pit mucous | FOSB | -1.2 | 2.59E-113 |
| Pit mucous | Pit mucous | MT1E | -1.13 | 2.84E-113 |
| Pit mucous | Pit mucous | TOMM7 | -0.68 | 3.17E-112 |
| Pit mucous | Pit mucous | HSP90AA1 | -0.79 | 4.13E-112 |
| Pit mucous | Pit mucous | SELENOW | 0.41 | 4.85E-111 |
| Pit mucous | Pit mucous | RAP1B | 0.28 | 9.30E-111 |
| Pit mucous | Pit mucous | C6orf132 | 0.26 | 9.41E-111 |
| Pit mucous | Pit mucous | NQO1 | 0.32 | 1.55E-110 |
| Pit mucous | Pit mucous | COA3 | 0.33 | 6.01E-110 |
| Pit mucous | Pit mucous | ARPC1A | 0.31 | 2.80E-109 |
| Pit mucous | Pit mucous | NCL | -0.81 | 2.03E-108 |
| Pit mucous | Pit mucous | MUC6 | -1.74 | 1.22E-107 |
| Pit mucous | Pit mucous | ZNF706 | 0.36 | 1.24E-107 |
| Pit mucous | Pit mucous | NDUFB5 | 0.29 | 1.60E-107 |
| Pit mucous | Pit mucous | MUCL3 | -1.45 | 2.25E-107 |
| Pit mucous | Pit mucous | NDUFS4 | 0.25 | 2.73E-107 |
| Pit mucous | Pit mucous | TUBA1C | 0.3 | 1.15E-106 |
| Pit mucous | Pit mucous | CTSE | 0.31 | 1.82E-106 |
| Pit mucous | Pit mucous | COQ4 | 0.26 | 1.45E-105 |
| Pit mucous | Pit mucous | NDUFV1 | 0.28 | 1.01E-104 |
| Pit mucous | Pit mucous | PPP1CA | 0.34 | 1.15E-104 |
| Pit mucous | Pit mucous | PRDX2 | 0.39 | 2.58E-104 |
| Pit mucous | Pit mucous | SERPINB1 | 0.35 | 3.82E-104 |
| Pit mucous | Pit mucous | TESC | -0.92 | 5.84E-103 |
| Pit mucous | Pit mucous | HMGN1 | -0.81 | 1.76E-101 |
| Pit mucous | Pit mucous | SPINK1 | -1.34 | 2.50E-101 |
| Pit mucous | Pit mucous | RARRES3 | 0.43 | 3.46E-101 |
| Pit mucous | Pit mucous | INSIG1 | 0.31 | 1.18E-100 |
| Pit mucous | Pit mucous | MPC2 | 0.29 | 6.03E-100 |
| Pit mucous | Pit mucous | TMEM123 | -0.64 | 1.04E-99 |
| Pit mucous | Pit mucous | FBL | -0.66 | 1.57E-99 |
| Pit mucous | Pit mucous | MSMB | -1.58 | 7.47E-99 |
| Pit mucous | Pit mucous | NDUFAB1 | 0.34 | 8.73E-99 |
| Pit mucous | Pit mucous | SNHG8 | -0.77 | 9.59E-99 |
| Pit mucous | Pit mucous | CSTB | 0.34 | 5.03E-98 |
| Pit mucous | Pit mucous | SNRPD2 | -0.75 | 9.21E-98 |
| Pit mucous | Pit mucous | TPD52 | 0.33 | 1.65E-97 |
| Pit mucous | Pit mucous | RTN4 | 0.3 | 5.57E-97 |
| Pit mucous | Pit mucous | ALDH3A1 | -0.93 | 5.77E-97 |
| Pit mucous | Pit mucous | PSMD8 | 0.27 | 1.80E-96 |
| Pit mucous | Pit mucous | TM4SF1 | 0.41 | 4.76E-96 |
| Pit mucous | Pit mucous | SLC12A2 | -0.67 | 5.42E-96 |
| Pit mucous | Pit mucous | RBM47 | 0.25 | 7.18E-96 |
| Pit mucous | Pit mucous | RPL8 | -0.39 | 1.09E-94 |
| Pit mucous | Pit mucous | IMPDH2 | -0.6 | 2.97E-94 |
| Pit mucous | Pit mucous | ALDH1A1 | 0.29 | 1.81E-93 |
| Pit mucous | Pit mucous | SOX4 | -0.94 | 1.88E-93 |
| Pit mucous | Pit mucous | CXCL8 | -1.56 | 2.05E-93 |
| Pit mucous | Pit mucous | VSIG1 | -0.81 | 7.72E-93 |
| Pit mucous | Pit mucous | CD164 | 0.28 | 1.58E-92 |
| Pit mucous | Pit mucous | TCEAL9 | -0.73 | 2.08E-92 |
| Pit mucous | Pit mucous | AC020656.1 | -1.63 | 5.26E-91 |
| Pit mucous | Pit mucous | TMSB10 | 0.3 | 1.31E-90 |
| Pit mucous | Pit mucous | PRELID1 | 0.35 | 5.21E-90 |
| Pit mucous | Pit mucous | DUT | -0.61 | 5.64E-90 |
| Pit mucous | Pit mucous | MT2A | -0.97 | 1.58E-89 |
| Pit mucous | Pit mucous | ALKBH7 | 0.29 | 2.42E-88 |
| Pit mucous | Pit mucous | FXYD5 | -0.66 | 2.12E-87 |
| Pit mucous | Pit mucous | NDUFB4 | 0.28 | 6.22E-87 |
| Pit mucous | Pit mucous | COMMD6 | -0.71 | 8.55E-87 |
| Pit mucous | Pit mucous | PSMB9 | 0.33 | 1.08E-86 |
| Pit mucous | Pit mucous | MZT2B | -0.76 | 3.21E-86 |
| Pit mucous | Pit mucous | TUBB2A | 0.25 | 5.38E-85 |
| Pit mucous | Pit mucous | PI3 | 0.38 | 1.28E-84 |
| Pit mucous | Pit mucous | NDUFC1 | 0.28 | 2.31E-84 |
| Pit mucous | Pit mucous | CD44 | -0.64 | 3.93E-84 |
| Pit mucous | Pit mucous | SSR4 | -0.92 | 4.06E-84 |
| Pit mucous | Pit mucous | HSPA1A | -1.28 | 5.87E-84 |
| Pit mucous | Pit mucous | MDH2 | 0.28 | 1.17E-83 |
| Pit mucous | Pit mucous | IFI27 | 0.73 | 1.73E-83 |
| Pit mucous | Pit mucous | TAGLN2 | -0.77 | 1.83E-83 |
| Pit mucous | Pit mucous | IER2 | -0.99 | 2.27E-83 |
| Pit mucous | Pit mucous | H2AFZ | -0.89 | 2.82E-83 |
| Pit mucous | Pit mucous | HMGB1 | -0.71 | 4.44E-82 |
| Pit mucous | Pit mucous | ATP5ME | 0.26 | 9.72E-82 |
| Pit mucous | Pit mucous | MINOS1 | 0.3 | 1.24E-81 |
| Pit mucous | Pit mucous | NDUFV2 | 0.26 | 4.72E-81 |
| Pit mucous | Pit mucous | LMO4 | -0.71 | 2.30E-80 |
| Pit mucous | Pit mucous | SF3B6 | 0.27 | 2.38E-80 |
| Pit mucous | Pit mucous | UBE2D3 | 0.27 | 3.69E-80 |
| Pit mucous | Pit mucous | TMEM258 | -0.63 | 6.86E-80 |
| Pit mucous | Pit mucous | NHP2 | -0.63 | 1.18E-79 |
| Pit mucous | Pit mucous | CDC42 | 0.26 | 3.59E-79 |
| Pit mucous | Pit mucous | NDUFS6 | 0.26 | 4.28E-78 |
| Pit mucous | Pit mucous | HMGN3 | -0.68 | 1.46E-77 |
| Pit mucous | Pit mucous | KIAA1324 | -0.54 | 2.28E-77 |
| Pit mucous | Pit mucous | LY6E | -0.68 | 4.85E-77 |
| Pit mucous | Pit mucous | ZFAS1 | -0.74 | 5.40E-77 |
| Pit mucous | Pit mucous | BEX3 | -0.61 | 6.06E-77 |
| Pit mucous | Pit mucous | EEF2 | -0.51 | 4.33E-76 |
| Pit mucous | Pit mucous | CLDN18 | -0.95 | 4.39E-76 |
| Pit mucous | Pit mucous | FIS1 | 0.25 | 1.30E-75 |
| Pit mucous | Pit mucous | NDUFB10 | 0.26 | 7.42E-75 |
| Pit mucous | Pit mucous | KRT10 | -0.65 | 5.68E-73 |
| Pit mucous | Pit mucous | DNPH1 | 0.25 | 7.32E-73 |
| Pit mucous | Pit mucous | SET | -0.68 | 1.26E-72 |
| Pit mucous | Pit mucous | RSL1D1 | -0.58 | 4.82E-72 |
| Pit mucous | Pit mucous | MLPH | -0.62 | 3.15E-71 |
| Pit mucous | Pit mucous | SST | -2.77 | 6.38E-71 |
| Pit mucous | Pit mucous | EIF1 | -0.39 | 1.59E-70 |
| Pit mucous | Pit mucous | CIRBP | -0.79 | 1.74E-70 |
| Pit mucous | Pit mucous | SUMO2 | -0.63 | 1.47E-69 |
| Pit mucous | Pit mucous | S100P | -0.89 | 8.22E-69 |
| Pit mucous | Pit mucous | CD59 | 0.26 | 1.30E-68 |
| Pit mucous | Pit mucous | CCND1 | -0.69 | 1.81E-68 |
| Pit mucous | Pit mucous | PABPC1 | -0.54 | 7.64E-68 |
| Pit mucous | Pit mucous | EEF1B2 | -0.59 | 6.15E-67 |
| Pit mucous | Pit mucous | NDUFAF8 | -0.45 | 2.72E-66 |
| Pit mucous | Pit mucous | SNRPF | -0.64 | 8.17E-66 |
| Pit mucous | Pit mucous | TRMT112 | -0.66 | 1.13E-65 |
| Pit mucous | Pit mucous | TUBB | -0.74 | 1.34E-65 |
| Pit mucous | Pit mucous | C6orf48 | -0.51 | 2.57E-65 |
| Pit mucous | Pit mucous | CHCHD2 | 0.25 | 5.85E-65 |
| Pit mucous | Pit mucous | HNRNPA0 | -0.67 | 1.53E-64 |
| Pit mucous | Pit mucous | EID1 | -0.65 | 1.62E-64 |
| Pit mucous | Pit mucous | CXCL2 | -1.13 | 4.29E-64 |
| Pit mucous | Pit mucous | ATP5MG | 0.25 | 2.05E-63 |
| Pit mucous | Pit mucous | GUK1 | 0.26 | 3.21E-63 |
| Pit mucous | Pit mucous | RPL22L1 | -0.66 | 1.05E-62 |
| Pit mucous | Pit mucous | STMN1 | -0.7 | 1.78E-62 |
| Pit mucous | Pit mucous | DDX21 | -0.59 | 3.31E-62 |
| Pit mucous | Pit mucous | GSTP1 | 0.28 | 1.03E-61 |
| Pit mucous | Pit mucous | NUCKS1 | -0.57 | 2.42E-61 |
| Pit mucous | Pit mucous | SNRPE | -0.61 | 5.24E-61 |
| Pit mucous | Pit mucous | BCAS1 | -0.65 | 1.04E-60 |
| Pit mucous | Pit mucous | JUN | -1.08 | 7.86E-60 |
| Pit mucous | Pit mucous | SNU13 | -0.6 | 1.01E-59 |
| Pit mucous | Pit mucous | RBMX | -0.58 | 2.67E-59 |
| Pit mucous | Pit mucous | PSCA | -1.66 | 3.50E-59 |
| Pit mucous | Pit mucous | PPP1R1B | -0.42 | 1.10E-58 |
| Pit mucous | Pit mucous | SOX9 | -0.55 | 2.79E-58 |
| Pit mucous | Pit mucous | EIF1AX | -0.64 | 3.68E-58 |
| Pit mucous | Pit mucous | APRT | -0.59 | 2.01E-57 |
| Pit mucous | Pit mucous | NME1 | -0.48 | 2.82E-57 |
| Pit mucous | Pit mucous | ZFP36L1 | -0.73 | 6.54E-57 |
| Pit mucous | Pit mucous | POLD2 | -0.37 | 3.68E-56 |
| Pit mucous | Pit mucous | SERBP1 | -0.61 | 7.04E-56 |
| Pit mucous | Pit mucous | NPDC1 | -0.49 | 3.51E-55 |
| Pit mucous | Pit mucous | TCEA3 | -0.4 | 6.79E-55 |
| Pit mucous | Pit mucous | HSPA1B | -0.97 | 7.96E-55 |
| Pit mucous | Pit mucous | FAAP20 | -0.49 | 8.27E-55 |
| Pit mucous | Pit mucous | YBX1 | -0.63 | 1.65E-54 |
| Pit mucous | Pit mucous | MZT2A | -0.59 | 2.20E-53 |
| Pit mucous | Pit mucous | VSIG2 | -0.72 | 2.32E-53 |
| Pit mucous | Pit mucous | C7orf50 | -0.39 | 2.97E-53 |
| Pit mucous | Pit mucous | C12orf57 | -0.61 | 5.69E-53 |
| Pit mucous | Pit mucous | NFIC | -0.37 | 6.38E-52 |
| Pit mucous | Pit mucous | LDHB | -0.58 | 1.35E-51 |
| Pit mucous | Pit mucous | FKBP11 | -0.68 | 1.95E-51 |
| Pit mucous | Pit mucous | MYC | -0.4 | 8.56E-51 |
| Pit mucous | Pit mucous | RANBP1 | -0.63 | 1.33E-50 |
| Pit mucous | Pit mucous | CCDC85B | -0.48 | 3.07E-50 |
| Pit mucous | Pit mucous | SNRPD1 | -0.56 | 3.76E-50 |
| Pit mucous | Pit mucous | RSL24D1 | -0.61 | 4.25E-50 |
| Pit mucous | Pit mucous | RAB27B | -0.57 | 5.25E-50 |
| Pit mucous | Pit mucous | PLAUR | 0.26 | 9.25E-50 |
| Pit mucous | Pit mucous | OLFM4 | -0.86 | 1.08E-49 |
| Pit mucous | Pit mucous | CDK4 | -0.31 | 1.22E-49 |
| Pit mucous | Pit mucous | EPB41L4A-AS1 | -0.37 | 1.31E-49 |
| Pit mucous | Pit mucous | ANKRD36C | -0.48 | 2.99E-49 |
| Pit mucous | Pit mucous | TCEAL8 | -0.38 | 5.62E-48 |
| Pit mucous | Pit mucous | REX1BD | -0.5 | 2.07E-47 |
| Pit mucous | Pit mucous | LSM7 | -0.56 | 2.31E-47 |
| Pit mucous | Pit mucous | VIM | -1.27 | 1.53E-46 |
| Pit mucous | Pit mucous | MIA | -0.61 | 2.95E-46 |
| Pit mucous | Pit mucous | KLK11 | -0.36 | 4.80E-46 |
| Pit mucous | Pit mucous | RASEF | -0.56 | 5.03E-46 |
| Pit mucous | Pit mucous | EIF3E | -0.64 | 7.63E-46 |
| Pit mucous | Pit mucous | MYADM | -0.45 | 1.00E-45 |
| Pit mucous | Pit mucous | SPINK4 | -1.08 | 1.46E-45 |
| Pit mucous | Pit mucous | MTHFD2 | -0.44 | 1.89E-45 |
| Pit mucous | Pit mucous | GNL3 | -0.33 | 2.05E-45 |
| Pit mucous | Pit mucous | SNHG19 | -0.34 | 5.00E-45 |
| Pit mucous | Pit mucous | CD81 | -0.37 | 2.59E-44 |
| Pit mucous | Pit mucous | SVIP | -0.6 | 3.83E-44 |
| Pit mucous | Pit mucous | CRIP2 | -0.54 | 4.01E-44 |
| Pit mucous | Pit mucous | RPL7 | -0.52 | 1.84E-43 |
| Pit mucous | Pit mucous | PRMT1 | -0.42 | 3.43E-43 |
| Pit mucous | Pit mucous | SOCS3 | -0.81 | 4.05E-43 |
| Pit mucous | Pit mucous | TOMM20 | -0.49 | 4.32E-43 |
| Pit mucous | Pit mucous | C1QBP | -0.44 | 7.92E-43 |
| Pit mucous | Pit mucous | DPP7 | -0.4 | 1.20E-42 |
| Pit mucous | Pit mucous | DMKN | -0.31 | 5.14E-42 |
| Pit mucous | Pit mucous | DDX5 | -0.47 | 5.23E-42 |
| Pit mucous | Pit mucous | SYTL1 | -0.31 | 6.11E-42 |
| Pit mucous | Pit mucous | HNRNPDL | -0.55 | 7.33E-42 |
| Pit mucous | Pit mucous | C19orf48 | -0.29 | 1.18E-41 |
| Pit mucous | Pit mucous | YBX3 | -0.43 | 1.87E-41 |
| Pit mucous | Pit mucous | LAGE3 | -0.33 | 7.51E-41 |
| Pit mucous | Pit mucous | SCGB2A1 | -0.6 | 9.72E-41 |
| Pit mucous | Pit mucous | CARHSP1 | -0.42 | 3.06E-40 |
| Pit mucous | Pit mucous | JTB | -0.5 | 3.40E-40 |
| Pit mucous | Pit mucous | TUBA1A | -0.77 | 4.29E-40 |
| Pit mucous | Pit mucous | SELENOM | -0.52 | 5.74E-40 |
| Pit mucous | Pit mucous | TTC3 | -0.43 | 8.63E-40 |
| Pit mucous | Pit mucous | C9orf16 | -0.52 | 1.85E-39 |
| Pit mucous | Pit mucous | PCBP2 | -0.51 | 2.68E-39 |
| Pit mucous | Pit mucous | CKAP4 | -0.38 | 4.36E-39 |
| Pit mucous | Pit mucous | TIMP1 | -0.77 | 8.83E-39 |
| Pit mucous | Pit mucous | BTG2 | -0.7 | 1.02E-38 |
| Pit mucous | Pit mucous | CUTA | -0.54 | 1.49E-38 |
| Pit mucous | Pit mucous | CDC42EP1 | -0.42 | 1.84E-38 |
| Pit mucous | Pit mucous | TMA7 | -0.33 | 2.06E-38 |
| Pit mucous | Pit mucous | ACTN1 | -0.28 | 2.28E-38 |
| Pit mucous | Pit mucous | ADD3 | -0.45 | 5.42E-38 |
| Pit mucous | Pit mucous | UQCC2 | -0.44 | 7.58E-38 |
| Pit mucous | Pit mucous | NFIA | -0.31 | 1.46E-37 |
| Pit mucous | Pit mucous | EIF3L | -0.54 | 1.53E-37 |
| Pit mucous | Pit mucous | SERF2 | -0.29 | 1.81E-37 |
| Pit mucous | Pit mucous | BAIAP2 | -0.32 | 2.81E-37 |
| Pit mucous | Pit mucous | UCP2 | -0.34 | 3.32E-37 |
| Pit mucous | Pit mucous | SNHG9 | -0.46 | 6.04E-37 |
| Pit mucous | Pit mucous | XIST | -0.39 | 7.36E-37 |
| Pit mucous | Pit mucous | DDX18 | -0.4 | 1.56E-36 |
| Pit mucous | Pit mucous | SMIM14 | -0.59 | 2.45E-36 |
| Pit mucous | Pit mucous | PAXX | -0.38 | 3.31E-36 |
| Pit mucous | Pit mucous | ANP32B | -0.48 | 3.53E-36 |
| Pit mucous | Pit mucous | CAVIN3 | -0.31 | 4.52E-36 |
| Pit mucous | Pit mucous | PYCR1 | -0.31 | 5.12E-36 |
| Pit mucous | Pit mucous | TRAPPC6A | -0.35 | 6.88E-36 |
| Pit mucous | Pit mucous | TCEAL4 | -0.29 | 8.82E-36 |
| Pit mucous | Pit mucous | RF00598 | -0.47 | 1.03E-35 |
| Pit mucous | Pit mucous | PRSS23 | -0.48 | 1.23E-35 |
| Pit mucous | Pit mucous | WDR43 | -0.27 | 2.41E-35 |
| Pit mucous | Pit mucous | GDF15 | -0.5 | 3.34E-35 |
| Pit mucous | Pit mucous | GADD45GIP1 | -0.51 | 3.62E-35 |
| Pit mucous | Pit mucous | CA9 | -0.38 | 3.97E-35 |
| Pit mucous | Pit mucous | PER2 | -0.32 | 6.05E-35 |
| Pit mucous | Pit mucous | CD320 | -0.71 | 6.37E-35 |
| Pit mucous | Pit mucous | NOP58 | -0.3 | 6.44E-35 |
| Pit mucous | Pit mucous | NOP56 | -0.31 | 6.88E-35 |
| Pit mucous | Pit mucous | AC023090.1 | -0.4 | 1.29E-34 |
| Pit mucous | Pit mucous | NASP | -0.32 | 1.91E-34 |
| Pit mucous | Pit mucous | DEPP1 | -0.5 | 2.18E-34 |
| Pit mucous | Pit mucous | KLF2 | -0.81 | 2.28E-34 |
| Pit mucous | Pit mucous | RAN | -0.54 | 2.93E-34 |
| Pit mucous | Pit mucous | NUPR1 | -0.39 | 3.53E-34 |
| Pit mucous | Pit mucous | LMNA | -0.43 | 4.83E-34 |
| Pit mucous | Pit mucous | C11orf58 | -0.49 | 5.17E-34 |
| Pit mucous | Pit mucous | EMP2 | -0.43 | 5.86E-34 |
| Pit mucous | Pit mucous | CXCL1 | -0.58 | 8.48E-34 |
| Pit mucous | Pit mucous | TGIF1 | -0.43 | 8.65E-34 |
| Pit mucous | Pit mucous | GTF3A | -0.38 | 9.48E-34 |
| Pit mucous | Pit mucous | KCNE2 | -0.27 | 1.83E-33 |
| Pit mucous | Pit mucous | APEX1 | -0.34 | 1.88E-33 |
| Pit mucous | Pit mucous | CLU | -0.91 | 2.43E-33 |
| Pit mucous | Pit mucous | TRA2B | -0.54 | 2.82E-33 |
| Pit mucous | Pit mucous | TAF1D | -0.38 | 3.10E-33 |
| Pit mucous | Pit mucous | PCSK1N | -1.05 | 6.52E-33 |
| Pit mucous | Pit mucous | C19orf53 | -0.49 | 6.63E-33 |
| Pit mucous | Pit mucous | SYNE2 | -0.33 | 6.93E-33 |
| Pit mucous | Pit mucous | EIF4A1 | -0.4 | 9.62E-33 |
| Pit mucous | Pit mucous | PAPSS1 | -0.32 | 9.98E-33 |
| Pit mucous | Pit mucous | TMEM97 | -0.26 | 1.60E-32 |
| Pit mucous | Pit mucous | NOLC1 | -0.25 | 2.20E-32 |
| Pit mucous | Pit mucous | MT1H | -0.6 | 3.12E-32 |
| Pit mucous | Pit mucous | DARS | -0.26 | 4.25E-32 |
| Pit mucous | Pit mucous | BTG3 | -0.27 | 4.27E-32 |
| Pit mucous | Pit mucous | TMEM160 | -0.42 | 5.66E-32 |
| Pit mucous | Pit mucous | ATP6V1G1 | -0.47 | 8.49E-32 |
| Pit mucous | Pit mucous | HNRNPA3 | -0.51 | 9.91E-32 |
| Pit mucous | Pit mucous | METAP2 | -0.42 | 1.86E-31 |
| Pit mucous | Pit mucous | PARP1 | -0.28 | 2.51E-31 |
| Pit mucous | Pit mucous | SLC9A3R2 | -0.47 | 3.52E-31 |
| Pit mucous | Pit mucous | MT1F | -0.54 | 5.44E-31 |
| Pit mucous | Pit mucous | PRKDC | -0.26 | 1.24E-30 |
| Pit mucous | Pit mucous | KDM6B | -0.44 | 2.91E-30 |
| Pit mucous | Pit mucous | IFITM2 | -0.59 | 3.68E-30 |
| Pit mucous | Pit mucous | EMP3 | -0.5 | 6.47E-30 |
| Pit mucous | Pit mucous | FOXP1 | -0.46 | 8.39E-30 |
| Pit mucous | Pit mucous | TRNP1 | -0.35 | 1.00E-29 |
| Pit mucous | Pit mucous | TMEM147 | -0.43 | 1.46E-29 |
| Pit mucous | Pit mucous | DNAJA1 | -0.58 | 2.07E-29 |
| Pit mucous | Pit mucous | CCDC34 | -0.27 | 2.18E-29 |
| Pit mucous | Pit mucous | EIF3F | -0.48 | 2.71E-29 |
| Pit mucous | Pit mucous | HNRNPH1 | -0.55 | 3.49E-29 |
| Pit mucous | Pit mucous | WDR74 | -0.26 | 4.19E-29 |
| Pit mucous | Pit mucous | EIF3A | -0.47 | 6.15E-29 |
| Pit mucous | Pit mucous | TMED3 | -0.42 | 7.63E-29 |
| Pit mucous | Pit mucous | SRGN | -1.03 | 7.74E-29 |
| Pit mucous | Pit mucous | CKB | -0.33 | 7.86E-29 |
| Pit mucous | Pit mucous | CYTOR | -0.45 | 1.00E-28 |
| Pit mucous | Pit mucous | BBX | -0.27 | 1.01E-28 |
| Pit mucous | Pit mucous | RNASE4 | -0.33 | 1.09E-28 |
| Pit mucous | Pit mucous | LGALS1 | -1.24 | 2.38E-28 |
| Pit mucous | Pit mucous | G3BP1 | -0.35 | 2.46E-28 |
| Pit mucous | Pit mucous | LCN2 | -0.69 | 2.72E-28 |
| Pit mucous | Pit mucous | MIR4435-2HG | -0.31 | 3.33E-28 |
| Pit mucous | Pit mucous | SLC25A6 | -0.34 | 4.92E-28 |
| Pit mucous | Pit mucous | MLXIP | -0.32 | 5.08E-28 |
| Pit mucous | Pit mucous | RAB40B | -0.29 | 6.53E-28 |
| Pit mucous | Pit mucous | IL1R2 | -0.3 | 7.67E-28 |
| Pit mucous | Pit mucous | SELENOH | -0.45 | 1.07E-27 |
| Pit mucous | Pit mucous | ILF3 | -0.35 | 1.52E-27 |
| Pit mucous | Pit mucous | PPIB | -0.46 | 1.57E-27 |
| Pit mucous | Pit mucous | HIPK2 | -0.32 | 1.64E-27 |
| Pit mucous | Pit mucous | BZW2 | -0.28 | 2.23E-27 |
| Pit mucous | Pit mucous | NUDC | -0.45 | 2.31E-27 |
| Pit mucous | Pit mucous | TPPP3 | -0.39 | 4.52E-27 |
| Pit mucous | Pit mucous | XBP1 | -0.56 | 4.59E-27 |
| Pit mucous | Pit mucous | IMPA2 | -0.33 | 6.22E-27 |
| Pit mucous | Pit mucous | RAB27A | -0.4 | 2.21E-26 |
| Pit mucous | Pit mucous | REXO2 | -0.29 | 3.63E-26 |
| Pit mucous | Pit mucous | PSAPL1 | -0.53 | 4.00E-26 |
| Pit mucous | Pit mucous | PPIA | -0.29 | 8.09E-26 |
| Pit mucous | Pit mucous | BTF3L4 | -0.26 | 8.73E-26 |
| Pit mucous | Pit mucous | JUND | -0.49 | 1.05E-25 |
| Pit mucous | Pit mucous | KRTCAP2 | -0.52 | 1.45E-25 |
| Pit mucous | Pit mucous | BACE2 | -0.39 | 1.64E-25 |
| Pit mucous | Pit mucous | LSM2 | -0.33 | 1.72E-25 |
| Pit mucous | Pit mucous | LIPF | -1.41 | 2.43E-25 |
| Pit mucous | Pit mucous | TSC22D3 | -1.04 | 3.82E-25 |
| Pit mucous | Pit mucous | RBBP4 | -0.36 | 3.98E-25 |
| Pit mucous | Pit mucous | ERO1B | -0.39 | 7.15E-25 |
| Pit mucous | Pit mucous | MAZ | -0.26 | 9.98E-25 |
| Pit mucous | Pit mucous | MT-ND4L | -0.44 | 1.02E-24 |
| Pit mucous | Pit mucous | EIF3G | -0.43 | 1.17E-24 |
| Pit mucous | Pit mucous | H3F3B | -0.35 | 1.35E-24 |
| Pit mucous | Pit mucous | HIST1H4C | -0.78 | 1.48E-24 |
| Pit mucous | Pit mucous | CYR61 | -0.34 | 2.16E-24 |
| Pit mucous | Pit mucous | ADAM28 | -0.28 | 2.16E-24 |
| Pit mucous | Pit mucous | PPP1R15A | -0.49 | 2.80E-24 |
| Pit mucous | Pit mucous | PRR4 | -0.75 | 7.54E-24 |
| Pit mucous | Pit mucous | PTMS | -0.37 | 8.74E-24 |
| Pit mucous | Pit mucous | CLNS1A | -0.31 | 1.05E-23 |
| Pit mucous | Pit mucous | RSBN1L | -0.28 | 1.20E-23 |
| Pit mucous | Pit mucous | H1FX | -0.26 | 1.35E-23 |
| Pit mucous | Pit mucous | UBE2S | -0.33 | 1.82E-23 |
| Pit mucous | Pit mucous | RNASET2 | -0.43 | 3.84E-23 |
| Pit mucous | Pit mucous | SLPI | -0.43 | 4.58E-23 |
| Pit mucous | Pit mucous | NAA38 | -0.45 | 5.98E-23 |
| Pit mucous | Pit mucous | ERP29 | -0.43 | 7.07E-23 |
| Pit mucous | Pit mucous | PRSS22 | -0.25 | 8.59E-23 |
| Pit mucous | Pit mucous | MECOM | -0.27 | 8.92E-23 |
| Pit mucous | Pit mucous | PCLAF | -0.37 | 1.33E-22 |
| Pit mucous | Pit mucous | KCNN4 | -0.3 | 1.43E-22 |
| Pit mucous | Pit mucous | HNRNPC | -0.44 | 1.60E-22 |
| Pit mucous | Pit mucous | MLEC | -0.36 | 1.69E-22 |
| Pit mucous | Pit mucous | CHID1 | -0.27 | 2.20E-22 |
| Pit mucous | Pit mucous | LUC7L3 | -0.35 | 2.93E-22 |
| Pit mucous | Pit mucous | ANG | -0.35 | 3.57E-22 |
| Pit mucous | Pit mucous | SNHG7 | -0.31 | 3.86E-22 |
| Pit mucous | Pit mucous | TMPO | -0.32 | 3.94E-22 |
| Pit mucous | Pit mucous | EIF3D | -0.41 | 5.07E-22 |
| Pit mucous | Pit mucous | SRRM2 | -0.45 | 5.32E-22 |
| Pit mucous | Pit mucous | RGS1 | -0.9 | 6.65E-22 |
| Pit mucous | Pit mucous | TRIM28 | -0.3 | 6.86E-22 |
| Pit mucous | Pit mucous | BRD2 | -0.45 | 7.83E-22 |
| Pit mucous | Pit mucous | HSPE1 | -0.47 | 9.32E-22 |
| Pit mucous | Pit mucous | PPIF | -0.32 | 1.12E-21 |
| Pit mucous | Pit mucous | RNPS1 | -0.38 | 2.18E-21 |
| Pit mucous | Pit mucous | FOXA3 | -0.43 | 2.54E-21 |
| Pit mucous | Pit mucous | MATR3 | -0.33 | 3.80E-21 |
| Pit mucous | Pit mucous | PRDX4 | -0.44 | 8.96E-21 |
| Pit mucous | Pit mucous | ANXA1 | -0.61 | 1.51E-20 |
| Pit mucous | Pit mucous | EEF1G | -0.37 | 1.55E-20 |
| Pit mucous | Pit mucous | ATRX | -0.28 | 1.56E-20 |
| Pit mucous | Pit mucous | HDAC2 | -0.33 | 1.56E-20 |
| Pit mucous | Pit mucous | NUCB2 | -0.34 | 3.71E-20 |
| Pit mucous | Pit mucous | CCT2 | -0.28 | 3.80E-20 |
| Pit mucous | Pit mucous | SF1 | -0.42 | 3.89E-20 |
| Pit mucous | Pit mucous | TSPO | -0.35 | 3.99E-20 |
| Pit mucous | Pit mucous | ARHGDIB | -0.5 | 7.74E-20 |
| Pit mucous | Pit mucous | HSPB1 | -0.54 | 1.16E-19 |
| Pit mucous | Pit mucous | SFPQ | -0.47 | 1.22E-19 |
| Pit mucous | Pit mucous | SYNGR2 | -0.42 | 1.32E-19 |
| Pit mucous | Pit mucous | MRPS26 | -0.28 | 2.66E-19 |
| Pit mucous | Pit mucous | EIF4B | -0.39 | 6.75E-19 |
| Pit mucous | Pit mucous | KLF10 | -0.43 | 7.35E-19 |
| Pit mucous | Pit mucous | RPS26 | -0.28 | 1.05E-18 |
| Pit mucous | Pit mucous | KCNE3 | -0.26 | 1.64E-18 |
| Pit mucous | Pit mucous | VKORC1 | -0.29 | 2.14E-18 |
| Pit mucous | Pit mucous | EIF4A3 | -0.37 | 3.13E-18 |
| Pit mucous | Pit mucous | HMGA1 | -0.5 | 4.04E-18 |
| Pit mucous | Pit mucous | EIF3M | -0.37 | 4.31E-18 |
| Pit mucous | Pit mucous | PA2G4 | -0.39 | 7.40E-18 |
| Pit mucous | Pit mucous | PHF14 | -0.26 | 8.13E-18 |
| Pit mucous | Pit mucous | IGLL5 | -2.44 | 8.14E-18 |
| Pit mucous | Pit mucous | BANF1 | -0.4 | 1.28E-17 |
| Pit mucous | Pit mucous | RUNX1 | -0.35 | 1.28E-17 |
| Pit mucous | Pit mucous | HNRNPR | -0.35 | 1.47E-17 |
| Pit mucous | Pit mucous | ERLEC1 | -0.33 | 1.55E-17 |
| Pit mucous | Pit mucous | SEC61G | -0.37 | 1.75E-17 |
| Pit mucous | Pit mucous | SGSM3 | -0.39 | 2.09E-17 |
| Pit mucous | Pit mucous | SSR3 | -0.43 | 2.61E-17 |
| Pit mucous | Pit mucous | HSPH1 | -0.39 | 2.89E-17 |
| Pit mucous | Pit mucous | PIGP | -0.25 | 3.14E-17 |
| Pit mucous | Pit mucous | PDCD5 | -0.34 | 3.22E-17 |
| Pit mucous | Pit mucous | LGMN | -0.36 | 3.47E-17 |
| Pit mucous | Pit mucous | LSM6 | -0.27 | 6.65E-17 |
| Pit mucous | Pit mucous | DDX17 | -0.44 | 8.62E-17 |
| Pit mucous | Pit mucous | NFKBIA | -0.49 | 1.47E-16 |
| Pit mucous | Pit mucous | IFI6 | -0.28 | 1.66E-16 |
| Pit mucous | Pit mucous | PRPF38B | -0.32 | 2.42E-16 |
| Pit mucous | Pit mucous | SEC62 | -0.45 | 4.05E-16 |
| Pit mucous | Pit mucous | HNRNPL | -0.28 | 4.26E-16 |
| Pit mucous | Pit mucous | SLC44A2 | -0.33 | 4.26E-16 |
| Pit mucous | Pit mucous | SQSTM1 | -0.43 | 5.99E-16 |
| Pit mucous | Pit mucous | RBBP6 | -0.34 | 6.13E-16 |
| Pit mucous | Pit mucous | MT1M | -0.28 | 6.74E-16 |
| Pit mucous | Pit mucous | SSR2 | -0.4 | 1.23E-15 |
| Pit mucous | Pit mucous | ATF4 | -0.42 | 1.68E-15 |
| Pit mucous | Pit mucous | RAD23A | -0.33 | 1.85E-15 |
| Pit mucous | Pit mucous | NME2 | -0.25 | 2.10E-15 |
| Pit mucous | Pit mucous | SEC63 | -0.26 | 2.21E-15 |
| Pit mucous | Pit mucous | EIF2S3 | -0.28 | 2.35E-15 |
| Pit mucous | Pit mucous | ARGLU1 | -0.45 | 3.29E-15 |
| Pit mucous | Pit mucous | DDIT4 | -0.59 | 3.57E-15 |
| Pit mucous | Pit mucous | MIR4458HG | -0.3 | 3.61E-15 |
| Pit mucous | Pit mucous | SLC38A2 | -0.45 | 8.22E-15 |
| Pit mucous | Pit mucous | BPTF | -0.26 | 9.26E-15 |
| Pit mucous | Pit mucous | S100A4 | -0.9 | 9.74E-15 |
| Pit mucous | Pit mucous | OST4 | -0.28 | 1.39E-14 |
| Pit mucous | Pit mucous | DEK | -0.39 | 1.98E-14 |
| Pit mucous | Pit mucous | ITM2C | -0.43 | 2.55E-14 |
| Pit mucous | Pit mucous | RBM25 | -0.36 | 2.60E-14 |
| Pit mucous | Pit mucous | MEAF6 | -0.33 | 2.84E-14 |
| Pit mucous | Pit mucous | TKT | -0.38 | 3.51E-14 |
| Pit mucous | Pit mucous | HSPD1 | -0.48 | 3.58E-14 |
| Pit mucous | Pit mucous | ILF2 | -0.3 | 4.33E-14 |
| Pit mucous | Pit mucous | BAD | -0.37 | 4.87E-14 |
| Pit mucous | Pit mucous | DGCR6L | -0.26 | 5.20E-14 |
| Pit mucous | Pit mucous | ARID5B | -0.33 | 6.71E-14 |
| Pit mucous | Pit mucous | NR4A1 | -0.5 | 7.37E-14 |
| Pit mucous | Pit mucous | ZNF428 | -0.27 | 7.62E-14 |
| Pit mucous | Pit mucous | SSB | -0.29 | 7.81E-14 |
| Pit mucous | Pit mucous | CKS2 | -0.39 | 9.96E-14 |
| Pit mucous | Pit mucous | SF3B5 | -0.37 | 1.33E-13 |
| Pit mucous | Pit mucous | NSA2 | -0.31 | 1.60E-13 |
| Pit mucous | Pit mucous | ST13 | -0.37 | 1.67E-13 |
| Pit mucous | Pit mucous | TMEM134 | -0.41 | 2.46E-13 |
| Pit mucous | Pit mucous | HMGB2 | -0.43 | 2.62E-13 |
| Pit mucous | Pit mucous | EIF3J | -0.39 | 3.03E-13 |
| Pit mucous | Pit mucous | ATP2A3 | -0.33 | 6.24E-13 |
| Pit mucous | Pit mucous | EIF5B | -0.38 | 1.23E-12 |
| Pit mucous | Pit mucous | EIF3B | -0.27 | 1.91E-12 |
| Pit mucous | Pit mucous | AKAP13 | -0.42 | 1.99E-12 |
| Pit mucous | Pit mucous | IFITM3 | -0.49 | 2.59E-12 |
| Pit mucous | Pit mucous | HNRNPD | -0.34 | 3.06E-12 |
| Pit mucous | Pit mucous | AMD1 | -0.35 | 3.61E-12 |
| Pit mucous | Pit mucous | DMAC1 | -0.3 | 4.55E-12 |
| Pit mucous | Pit mucous | OTUD1 | -0.32 | 6.14E-12 |
| Pit mucous | Pit mucous | DDX24 | -0.33 | 7.47E-12 |
| Pit mucous | Pit mucous | ANKRD28 | -0.45 | 8.23E-12 |
| Pit mucous | Pit mucous | TNFAIP3 | -0.48 | 8.73E-12 |
| Pit mucous | Pit mucous | TMED9 | -0.38 | 1.31E-11 |
| Pit mucous | Pit mucous | TOB1 | -0.41 | 1.37E-11 |
| Pit mucous | Pit mucous | IMP3 | -0.29 | 1.97E-11 |
| Pit mucous | Pit mucous | IGFBP2 | -0.38 | 4.80E-11 |
| Pit mucous | Pit mucous | EI24 | -0.33 | 7.36E-11 |
| Pit mucous | Pit mucous | METTL26 | -0.29 | 8.56E-11 |
| Pit mucous | Pit mucous | B4GALNT3 | -0.28 | 1.04E-10 |
| Pit mucous | Pit mucous | KPNB1 | -0.27 | 1.11E-10 |
| Pit mucous | Pit mucous | BUD23 | -0.3 | 1.23E-10 |
| Pit mucous | Pit mucous | SNRPG | -0.38 | 1.57E-10 |
| Pit mucous | Pit mucous | NONO | -0.28 | 1.95E-10 |
| Pit mucous | Pit mucous | WFDC2 | -0.42 | 1.97E-10 |
| Pit mucous | Pit mucous | RNASEH2C | -0.28 | 2.51E-10 |
| Pit mucous | Pit mucous | PHPT1 | -0.34 | 2.73E-10 |
| Pit mucous | Pit mucous | NME3 | -0.29 | 2.73E-10 |
| Pit mucous | Pit mucous | PDCD4 | -0.3 | 3.52E-10 |
| Pit mucous | Pit mucous | ATRAID | -0.34 | 3.72E-10 |
| Pit mucous | Pit mucous | UPF2 | -0.29 | 3.78E-10 |
| Pit mucous | Pit mucous | SREK1 | -0.25 | 5.37E-10 |
| Pit mucous | Pit mucous | METTL9 | -0.26 | 5.44E-10 |
| Pit mucous | Pit mucous | PLK2 | -0.26 | 5.74E-10 |
| Pit mucous | Pit mucous | SLC39A11 | -0.29 | 1.55E-09 |
| Pit mucous | Pit mucous | RAB13 | -0.3 | 1.98E-09 |
| Pit mucous | Pit mucous | PRKCI | -0.25 | 4.67E-09 |
| Pit mucous | Pit mucous | GGCT | -0.26 | 4.84E-09 |
| Pit mucous | Pit mucous | POLR2I | -0.31 | 5.60E-09 |
| Pit mucous | Pit mucous | AC020916.1 | -0.32 | 5.73E-09 |
| Pit mucous | Pit mucous | CHD2 | -0.31 | 5.95E-09 |
| Pit mucous | Pit mucous | HES1 | -0.56 | 6.26E-09 |
| Pit mucous | Pit mucous | TUBA1B | -0.51 | 7.91E-09 |
| Pit mucous | Pit mucous | H2AFV | -0.33 | 8.56E-09 |
| Pit mucous | Pit mucous | ERH | -0.36 | 9.03E-09 |
| Pit mucous | Pit mucous | TRAPPC2L | -0.3 | 1.04E-08 |
| Pit mucous | Pit mucous | VAMP2 | -0.42 | 1.10E-08 |
| Pit mucous | Pit mucous | PABPC4 | -0.29 | 1.22E-08 |
| Pit mucous | Pit mucous | GTF2I | -0.3 | 1.45E-08 |
| Pit mucous | Pit mucous | TCEA1 | -0.28 | 1.51E-08 |
| Pit mucous | Pit mucous | DDOST | -0.28 | 1.65E-08 |
| Pit mucous | Pit mucous | HNRNPM | -0.36 | 2.98E-08 |
| Pit mucous | Pit mucous | HLA-DPA1 | -0.5 | 3.42E-08 |
| Pit mucous | Pit mucous | SRRM1 | -0.33 | 3.43E-08 |
| Pit mucous | Pit mucous | ANXA5 | -0.39 | 3.46E-08 |
| Pit mucous | Pit mucous | GNAQ | -0.3 | 4.60E-08 |
| Pit mucous | Pit mucous | CREM | -0.5 | 6.67E-08 |
| Pit mucous | Pit mucous | BPIFB1 | -0.75 | 7.77E-08 |
| Pit mucous | Pit mucous | HLA-DPB1 | -0.58 | 8.68E-08 |
| Pit mucous | Pit mucous | FAM107B | -0.35 | 9.14E-08 |
| Pit mucous | Pit mucous | BAG1 | -0.34 | 9.17E-08 |
| Pit mucous | Pit mucous | TERF2IP | -0.26 | 9.32E-08 |
| Pit mucous | Pit mucous | LGALS9 | -0.35 | 1.46E-07 |
| Pit mucous | Pit mucous | AKR1C2 | -0.4 | 1.65E-07 |
| Pit mucous | Pit mucous | BST2 | -0.31 | 2.78E-07 |
| Pit mucous | Pit mucous | KLF9 | -0.27 | 3.20E-07 |
| Pit mucous | Pit mucous | DCXR | -0.31 | 5.58E-07 |
| Pit mucous | Pit mucous | GSN | -0.29 | 8.05E-07 |
| Pit mucous | Pit mucous | MRPS34 | -0.3 | 8.56E-07 |
| Pit mucous | Pit mucous | TFF3 | -1.67 | 9.28E-07 |
| Pit mucous | Pit mucous | CRACR2B | -0.28 | 1.03E-06 |
| Pit mucous | Pit mucous | ANP32A | -0.26 | 1.11E-06 |
| Pit mucous | Pit mucous | MRPL57 | -0.31 | 1.31E-06 |
| Pit mucous | Pit mucous | SLTM | -0.28 | 1.54E-06 |
| Pit mucous | Pit mucous | HNRNPU | -0.34 | 2.86E-06 |
| Pit mucous | Pit mucous | QSOX1 | -0.32 | 3.14E-06 |
| Pit mucous | Pit mucous | ZFP36 | -0.77 | 3.98E-06 |
| Pit mucous | Pit mucous | HNRNPK | -0.27 | 4.05E-06 |
| Pit mucous | Pit mucous | FAM133B | -0.32 | 4.51E-06 |
| Pit mucous | Pit mucous | CCNG1 | -0.27 | 6.20E-06 |
| Pit mucous | Pit mucous | CMTM6 | -0.3 | 6.54E-06 |
| Pit mucous | Pit mucous | NR4A2 | -0.33 | 6.63E-06 |
| Pit mucous | Pit mucous | OLFM4 | 1.22 | 0 |
| Pit mucous | Pit mucous | REG4 | 1.09 | 0 |
| Pit mucous | Pit mucous | GAPDH | 0.86 | 0 |
| Pit mucous | Pit mucous | LGALS4 | 0.85 | 0 |
| Pit mucous | Pit mucous | RPS2 | 0.84 | 0 |
| Pit mucous | Pit mucous | EEF1B2 | 0.82 | 0 |
| Pit mucous | Pit mucous | RPL10A | 0.8 | 0 |
| Pit mucous | Pit mucous | RPL4 | 0.79 | 0 |
| Pit mucous | Pit mucous | RPL5 | 0.79 | 0 |
| Pit mucous | Pit mucous | RPS7 | 0.78 | 0 |
| Pit mucous | Pit mucous | IDH2 | 0.78 | 0 |
| Pit mucous | Pit mucous | SLC25A6 | 0.77 | 0 |
| Pit mucous | Pit mucous | RPL6 | 0.76 | 0 |
| Pit mucous | Pit mucous | TFF3 | 0.75 | 0 |
| Pit mucous | Pit mucous | ETHE1 | 0.73 | 0 |
| Pit mucous | Pit mucous | LGALS3 | 0.73 | 0 |
| Pit mucous | Pit mucous | RPLP0 | 0.73 | 0 |
| Pit mucous | Pit mucous | RPSA | 0.71 | 0 |
| Pit mucous | Pit mucous | S100A10 | 0.71 | 0 |
| Pit mucous | Pit mucous | RACK1 | 0.71 | 0 |
| Pit mucous | Pit mucous | NACA | 0.7 | 0 |
| Pit mucous | Pit mucous | RPL7A | 0.7 | 0 |
| Pit mucous | Pit mucous | RPS5 | 0.68 | 0 |
| Pit mucous | Pit mucous | RPS3 | 0.67 | 0 |
| Pit mucous | Pit mucous | KRT7 | 0.66 | 0 |
| Pit mucous | Pit mucous | UQCRH | 0.65 | 0 |
| Pit mucous | Pit mucous | CLDN4 | 0.65 | 0 |
| Pit mucous | Pit mucous | RPS6 | 0.65 | 0 |
| Pit mucous | Pit mucous | IMPDH2 | 0.65 | 0 |
| Pit mucous | Pit mucous | RPL3 | 0.64 | 0 |
| Pit mucous | Pit mucous | DMBT1 | 0.64 | 0 |
| Pit mucous | Pit mucous | CLDN7 | 0.63 | 0 |
| Pit mucous | Pit mucous | PPP1R1B | 0.63 | 0 |
| Pit mucous | Pit mucous | RPL8 | 0.6 | 0 |
| Pit mucous | Pit mucous | EIF3L | 0.6 | 0 |
| Pit mucous | Pit mucous | RPL29 | 0.6 | 0 |
| Pit mucous | Pit mucous | RPL19 | 0.59 | 0 |
| Pit mucous | Pit mucous | PPA1 | 0.59 | 0 |
| Pit mucous | Pit mucous | PRSS3 | 0.58 | 0 |
| Pit mucous | Pit mucous | RPL18 | 0.57 | 0 |
| Pit mucous | Pit mucous | RPL14 | 0.57 | 0 |
| Pit mucous | Pit mucous | RPS16 | 0.57 | 0 |
| Pit mucous | Pit mucous | RPS23 | 0.57 | 0 |
| Pit mucous | Pit mucous | RPL12 | 0.57 | 0 |
| Pit mucous | Pit mucous | RPL13A | 0.55 | 0 |
| Pit mucous | Pit mucous | EEF1A1 | 0.55 | 0 |
| Pit mucous | Pit mucous | C1QBP | 0.54 | 0 |
| Pit mucous | Pit mucous | RPS9 | 0.54 | 0 |
| Pit mucous | Pit mucous | EIF3F | 0.53 | 0 |
| Pit mucous | Pit mucous | TRIM54 | 0.52 | 0 |
| Pit mucous | Pit mucous | HSPD1 | 0.51 | 0 |
| Pit mucous | Pit mucous | CPS1 | 0.51 | 0 |
| Pit mucous | Pit mucous | FAM3D | 0.48 | 0 |
| Pit mucous | Pit mucous | AHCY | 0.48 | 0 |
| Pit mucous | Pit mucous | PHB | 0.48 | 0 |
| Pit mucous | Pit mucous | HMGCS2 | 0.47 | 0 |
| Pit mucous | Pit mucous | TKT | 0.47 | 0 |
| Pit mucous | Pit mucous | RAMP1 | 0.47 | 0 |
| Pit mucous | Pit mucous | LEFTY1 | 0.46 | 0 |
| Pit mucous | Pit mucous | EIF3D | 0.45 | 0 |
| Pit mucous | Pit mucous | CLDN3 | 0.45 | 0 |
| Pit mucous | Pit mucous | RAB25 | 0.44 | 0 |
| Pit mucous | Pit mucous | LDHB | 0.44 | 0 |
| Pit mucous | Pit mucous | NME1 | 0.43 | 0 |
| Pit mucous | Pit mucous | SERPINB5 | 0.42 | 0 |
| Pit mucous | Pit mucous | FBL | 0.4 | 0 |
| Pit mucous | Pit mucous | EIF3I | 0.39 | 0 |
| Pit mucous | Pit mucous | MRPL12 | 0.39 | 0 |
| Pit mucous | Pit mucous | MRPS12 | 0.39 | 0 |
| Pit mucous | Pit mucous | PRAC1 | 0.39 | 0 |
| Pit mucous | Pit mucous | STOML2 | 0.39 | 0 |
| Pit mucous | Pit mucous | PHB2 | 0.38 | 0 |
| Pit mucous | Pit mucous | GDF15 | 0.38 | 0 |
| Pit mucous | Pit mucous | SLC25A39 | 0.38 | 0 |
| Pit mucous | Pit mucous | PCBD1 | 0.38 | 0 |
| Pit mucous | Pit mucous | OXA1L | 0.36 | 0 |
| Pit mucous | Pit mucous | CCT3 | 0.36 | 0 |
| Pit mucous | Pit mucous | PYCARD | 0.36 | 0 |
| Pit mucous | Pit mucous | MCRIP2 | 0.35 | 0 |
| Pit mucous | Pit mucous | PLPP2 | 0.35 | 0 |
| Pit mucous | Pit mucous | PTRHD1 | 0.34 | 0 |
| Pit mucous | Pit mucous | CCND2 | 0.34 | 0 |
| Pit mucous | Pit mucous | GP2 | 0.34 | 0 |
| Pit mucous | Pit mucous | EIF3M | 0.34 | 0 |
| Pit mucous | Pit mucous | SUCLG1 | 0.33 | 0 |
| Pit mucous | Pit mucous | KRTCAP3 | 0.32 | 0 |
| Pit mucous | Pit mucous | APEX1 | 0.32 | 0 |
| Pit mucous | Pit mucous | CLDN15 | 0.32 | 0 |
| Pit mucous | Pit mucous | GJB3 | 0.31 | 0 |
| Pit mucous | Pit mucous | NOB1 | 0.31 | 0 |
| Pit mucous | Pit mucous | MRPL4 | 0.31 | 0 |
| Pit mucous | Pit mucous | TMEM45B | 0.31 | 0 |
| Pit mucous | Pit mucous | STAP2 | 0.31 | 0 |
| Pit mucous | Pit mucous | SDHB | 0.31 | 0 |
| Pit mucous | Pit mucous | KLK1 | 0.3 | 0 |
| Pit mucous | Pit mucous | PLEK2 | 0.3 | 0 |
| Pit mucous | Pit mucous | OLA1 | 0.3 | 0 |
| Pit mucous | Pit mucous | FGFBP1 | 0.29 | 0 |
| Pit mucous | Pit mucous | IGBP1 | 0.29 | 0 |
| Pit mucous | Pit mucous | LAD1 | 0.29 | 0 |
| Pit mucous | Pit mucous | ZNF511 | 0.28 | 0 |
| Pit mucous | Pit mucous | SLC39A5 | 0.28 | 0 |
| Pit mucous | Pit mucous | CDX1 | 0.28 | 0 |
| Pit mucous | Pit mucous | MRPS33 | 0.27 | 0 |
| Pit mucous | Pit mucous | DCTPP1 | 0.27 | 0 |
| Pit mucous | Pit mucous | MRPL37 | 0.27 | 0 |
| Pit mucous | Pit mucous | ABHD14B | 0.27 | 0 |
| Pit mucous | Pit mucous | HSPBP1 | 0.26 | 0 |
| Pit mucous | Pit mucous | HSD17B10 | 0.26 | 0 |
| Pit mucous | Pit mucous | C19orf48 | 0.26 | 0 |
| Pit mucous | Pit mucous | ASS1 | 0.26 | 0 |
| Pit mucous | Pit mucous | MRPL11 | 0.26 | 0 |
| Pit mucous | Pit mucous | EBNA1BP2 | 0.26 | 0 |
| Pit mucous | Pit mucous | CCT2 | 0.25 | 0 |
| Pit mucous | Pit mucous | BTF3 | 0.55 | 4.11E-305 |
| Pit mucous | Pit mucous | RPL15 | 0.56 | 6.20E-305 |
| Pit mucous | Pit mucous | EIF4EBP1 | 0.27 | 6.04E-304 |
| Pit mucous | Pit mucous | RSL1D1 | 0.3 | 3.09E-300 |
| Pit mucous | Pit mucous | TOMM22 | 0.35 | 3.82E-300 |
| Pit mucous | Pit mucous | UQCRC2 | 0.35 | 7.56E-299 |
| Pit mucous | Pit mucous | MDH2 | 0.47 | 1.16E-296 |
| Pit mucous | Pit mucous | EDN1 | 0.32 | 2.38E-296 |
| Pit mucous | Pit mucous | TUFM | 0.49 | 2.71E-295 |
| Pit mucous | Pit mucous | RPS13 | 0.54 | 3.43E-294 |
| Pit mucous | Pit mucous | NHP2 | 0.4 | 1.68E-291 |
| Pit mucous | Pit mucous | ISOC2 | 0.25 | 2.17E-290 |
| Pit mucous | Pit mucous | RPS18 | 0.53 | 1.20E-288 |
| Pit mucous | Pit mucous | C8orf59 | 0.25 | 4.67E-285 |
| Pit mucous | Pit mucous | CXCL1 | 0.26 | 3.27E-280 |
| Pit mucous | Pit mucous | RPL11 | 0.45 | 1.37E-277 |
| Pit mucous | Pit mucous | RPL26 | 0.63 | 3.78E-276 |
| Pit mucous | Pit mucous | GTF3C6 | 0.27 | 1.97E-275 |
| Pit mucous | Pit mucous | EIF3H | 0.49 | 8.16E-275 |
| Pit mucous | Pit mucous | TNFRSF12A | 0.43 | 7.61E-273 |
| Pit mucous | Pit mucous | CES2 | 0.34 | 7.68E-273 |
| Pit mucous | Pit mucous | SLC9A3R1 | 0.26 | 4.39E-272 |
| Pit mucous | Pit mucous | NDUFB5 | 0.29 | 3.22E-266 |
| Pit mucous | Pit mucous | RPS8 | 0.51 | 4.94E-266 |
| Pit mucous | Pit mucous | TMEM147 | 0.3 | 5.60E-265 |
| Pit mucous | Pit mucous | CYC1 | 0.5 | 1.95E-264 |
| Pit mucous | Pit mucous | RPL9 | 0.47 | 1.03E-263 |
| Pit mucous | Pit mucous | SERINC2 | 0.28 | 1.93E-262 |
| Pit mucous | Pit mucous | PDCD6 | 0.33 | 9.53E-260 |
| Pit mucous | Pit mucous | PSMB5 | 0.33 | 1.18E-259 |
| Pit mucous | Pit mucous | TXN2 | 0.27 | 6.10E-253 |
| Pit mucous | Pit mucous | MT1M | 0.29 | 2.42E-251 |
| Pit mucous | Pit mucous | RAB5IF | 0.3 | 1.37E-248 |
| Pit mucous | Pit mucous | SLC25A5 | 0.53 | 8.31E-248 |
| Pit mucous | Pit mucous | GSTK1 | 0.37 | 1.56E-247 |
| Pit mucous | Pit mucous | NDUFV1 | 0.27 | 8.85E-247 |
| Pit mucous | Pit mucous | RPL22L1 | 0.38 | 4.19E-244 |
| Pit mucous | Pit mucous | CEACAM6 | 0.36 | 8.25E-244 |
| Pit mucous | Pit mucous | ATP5MC1 | 0.51 | 1.53E-241 |
| Pit mucous | Pit mucous | HMGA1 | 0.51 | 6.12E-241 |
| Pit mucous | Pit mucous | RPS20 | 0.5 | 1.42E-239 |
| Pit mucous | Pit mucous | MISP | 0.26 | 3.85E-239 |
| Pit mucous | Pit mucous | SERPINB1 | 0.51 | 1.50E-237 |
| Pit mucous | Pit mucous | RPLP1 | 0.42 | 1.72E-237 |
| Pit mucous | Pit mucous | RPS19 | 0.45 | 1.44E-235 |
| Pit mucous | Pit mucous | PDLIM1 | 0.35 | 1.92E-235 |
| Pit mucous | Pit mucous | NDUFAB1 | 0.39 | 2.73E-234 |
| Pit mucous | Pit mucous | ANXA2 | 0.58 | 9.33E-234 |
| Pit mucous | Pit mucous | S100A16 | 0.59 | 3.24E-232 |
| Pit mucous | Pit mucous | SERPINB6 | 0.37 | 9.21E-231 |
| Pit mucous | Pit mucous | RPL22 | 0.48 | 7.04E-229 |
| Pit mucous | Pit mucous | WFDC2 | 0.28 | 2.68E-228 |
| Pit mucous | Pit mucous | ARPC1B | 0.31 | 4.41E-228 |
| Pit mucous | Pit mucous | ENO1 | 0.49 | 7.23E-228 |
| Pit mucous | Pit mucous | RPS12 | 0.47 | 7.67E-228 |
| Pit mucous | Pit mucous | RPL13 | 0.42 | 2.45E-225 |
| Pit mucous | Pit mucous | KRT18 | 0.63 | 4.43E-225 |
| Pit mucous | Pit mucous | ATP5PB | 0.35 | 6.92E-225 |
| Pit mucous | Pit mucous | RPS26 | 0.44 | 8.32E-225 |
| Pit mucous | Pit mucous | EIF3K | 0.47 | 3.70E-223 |
| Pit mucous | Pit mucous | RPS4Y1 | 0.57 | 2.65E-222 |
| Pit mucous | Pit mucous | RPL7 | 0.35 | 3.90E-221 |
| Pit mucous | Pit mucous | RPL18A | 0.4 | 3.40E-220 |
| Pit mucous | Pit mucous | COX5A | 0.48 | 3.59E-220 |
| Pit mucous | Pit mucous | COX4I1 | 0.38 | 1.89E-217 |
| Pit mucous | Pit mucous | RPL30 | 0.45 | 2.02E-217 |
| Pit mucous | Pit mucous | ATP5F1B | 0.47 | 2.10E-216 |
| Pit mucous | Pit mucous | ATP5MC3 | 0.52 | 1.17E-214 |
| Pit mucous | Pit mucous | PIGR | 0.54 | 1.55E-214 |
| Pit mucous | Pit mucous | RPS4X | 0.47 | 3.58E-214 |
| Pit mucous | Pit mucous | EIF3G | 0.26 | 3.91E-214 |
| Pit mucous | Pit mucous | PRELID1 | 0.45 | 5.41E-213 |
| Pit mucous | Pit mucous | EPCAM | 0.44 | 2.74E-211 |
| Pit mucous | Pit mucous | RPL24 | 0.41 | 6.54E-210 |
| Pit mucous | Pit mucous | ALDH2 | 0.28 | 3.94E-209 |
| Pit mucous | Pit mucous | COA3 | 0.29 | 2.15E-208 |
| Pit mucous | Pit mucous | NPM1 | 0.51 | 9.02E-208 |
| Pit mucous | Pit mucous | MPC2 | 0.28 | 9.51E-208 |
| Pit mucous | Pit mucous | HNRNPA1 | 0.5 | 1.31E-207 |
| Pit mucous | Pit mucous | PKM | 0.42 | 2.57E-206 |
| Pit mucous | Pit mucous | CD9 | 0.4 | 4.67E-205 |
| Pit mucous | Pit mucous | RPL23 | 0.45 | 5.88E-205 |
| Pit mucous | Pit mucous | ATP5F1A | 0.42 | 1.38E-203 |
| Pit mucous | Pit mucous | ECHS1 | 0.29 | 6.01E-203 |
| Pit mucous | Pit mucous | DDT | 0.43 | 7.80E-202 |
| Pit mucous | Pit mucous | ECH1 | 0.27 | 2.80E-198 |
| Pit mucous | Pit mucous | RPL32 | 0.42 | 9.31E-197 |
| Pit mucous | Pit mucous | HSPA8 | 0.42 | 3.21E-195 |
| Pit mucous | Pit mucous | ATP5F1C | 0.34 | 1.02E-194 |
| Pit mucous | Pit mucous | SNRPE | 0.25 | 3.28E-194 |
| Pit mucous | Pit mucous | GKN1 | -3.8 | 7.11E-193 |
| Pit mucous | Pit mucous | SNRPB | 0.3 | 1.45E-192 |
| Pit mucous | Pit mucous | RPL28 | 0.38 | 3.20E-192 |
| Pit mucous | Pit mucous | GPX1 | 0.38 | 7.06E-192 |
| Pit mucous | Pit mucous | ERGIC3 | 0.35 | 2.77E-191 |
| Pit mucous | Pit mucous | RPL27 | 0.41 | 3.23E-191 |
| Pit mucous | Pit mucous | TSTA3 | 0.27 | 3.78E-190 |
| Pit mucous | Pit mucous | RPS3A | 0.42 | 5.56E-190 |
| Pit mucous | Pit mucous | UQCRFS1 | 0.33 | 9.48E-190 |
| Pit mucous | Pit mucous | DNPH1 | 0.36 | 3.14E-189 |
| Pit mucous | Pit mucous | EEF2 | 0.45 | 4.32E-187 |
| Pit mucous | Pit mucous | APRT | 0.41 | 5.91E-186 |
| Pit mucous | Pit mucous | CFD | -0.25 | 6.78E-185 |
| Pit mucous | Pit mucous | GAST | -3.85 | 1.22E-184 |
| Pit mucous | Pit mucous | PHLDA2 | 0.54 | 2.26E-184 |
| Pit mucous | Pit mucous | ZFAS1 | 0.38 | 5.90E-184 |
| Pit mucous | Pit mucous | TIMM13 | 0.38 | 6.83E-184 |
| Pit mucous | Pit mucous | AGR3 | 0.34 | 1.28E-183 |
| Pit mucous | Pit mucous | PGC | -3.24 | 1.67E-182 |
| Pit mucous | Pit mucous | ALDOA | 0.47 | 6.10E-182 |
| Pit mucous | Pit mucous | RPS14 | 0.36 | 4.75E-180 |
| Pit mucous | Pit mucous | PARK7 | 0.33 | 6.69E-179 |
| Pit mucous | Pit mucous | CD320 | -0.35 | 1.85E-176 |
| Pit mucous | Pit mucous | RPL27A | 0.38 | 3.24E-175 |
| Pit mucous | Pit mucous | RPL37A | 0.39 | 8.08E-171 |
| Pit mucous | Pit mucous | NDUFB9 | 0.36 | 1.24E-170 |
| Pit mucous | Pit mucous | PEBP1 | 0.38 | 5.84E-168 |
| Pit mucous | Pit mucous | UQCRC1 | 0.28 | 5.67E-167 |
| Pit mucous | Pit mucous | TFF1 | -2.1 | 9.07E-167 |
| Pit mucous | Pit mucous | TPI1 | 0.44 | 3.24E-166 |
| Pit mucous | Pit mucous | RPL10 | 0.34 | 4.01E-163 |
| Pit mucous | Pit mucous | YBX1 | 0.44 | 1.69E-162 |
| Pit mucous | Pit mucous | ITLN1 | 0.38 | 1.39E-161 |
| Pit mucous | Pit mucous | SLC25A3 | 0.38 | 1.20E-159 |
| Pit mucous | Pit mucous | HINT1 | 0.36 | 1.42E-158 |
| Pit mucous | Pit mucous | VDAC2 | 0.26 | 4.14E-158 |
| Pit mucous | Pit mucous | RPL35A | 0.36 | 8.93E-154 |
| Pit mucous | Pit mucous | S100A14 | 0.54 | 1.31E-153 |
| Pit mucous | Pit mucous | EIF3E | 0.36 | 7.43E-153 |
| Pit mucous | Pit mucous | REG1A | 0.79 | 7.74E-153 |
| Pit mucous | Pit mucous | ATP5PO | 0.36 | 2.15E-152 |
| Pit mucous | Pit mucous | FABP2 | -0.75 | 6.53E-152 |
| Pit mucous | Pit mucous | NDUFC2 | 0.31 | 3.07E-151 |
| Pit mucous | Pit mucous | RPL23A | 0.38 | 2.78E-150 |
| Pit mucous | Pit mucous | RAN | 0.31 | 1.49E-149 |
| Pit mucous | Pit mucous | PPIA | 0.37 | 2.64E-149 |
| Pit mucous | Pit mucous | FCGRT | 0.26 | 1.81E-144 |
| Pit mucous | Pit mucous | RPS21 | 0.34 | 3.59E-143 |
| Pit mucous | Pit mucous | RPL21 | 0.39 | 2.82E-142 |
| Pit mucous | Pit mucous | RBM3 | 0.32 | 3.38E-142 |
| Pit mucous | Pit mucous | CHCHD2 | 0.36 | 1.57E-141 |
| Pit mucous | Pit mucous | SERBP1 | 0.25 | 6.03E-139 |
| Pit mucous | Pit mucous | RPS24 | 0.45 | 6.18E-139 |
| Pit mucous | Pit mucous | GKN2 | -2.75 | 1.45E-137 |
| Pit mucous | Pit mucous | RPS27A | 0.3 | 3.38E-136 |
| Pit mucous | Pit mucous | HSPE1 | 0.31 | 4.74E-135 |
| Pit mucous | Pit mucous | TFF2 | -2.12 | 1.31E-134 |
| Pit mucous | Pit mucous | JUND | -1.01 | 2.85E-134 |
| Pit mucous | Pit mucous | GPRC5A | 0.28 | 3.00E-131 |
| Pit mucous | Pit mucous | RPL37 | 0.33 | 5.44E-129 |
| Pit mucous | Pit mucous | TSPAN8 | 0.31 | 6.00E-128 |
| Pit mucous | Pit mucous | RPS11 | 0.29 | 1.07E-126 |
| Pit mucous | Pit mucous | COX6A1 | 0.35 | 1.97E-124 |
| Pit mucous | Pit mucous | CCL25 | 0.39 | 5.95E-124 |
| Pit mucous | Pit mucous | UQCRB | 0.37 | 4.11E-123 |
| Pit mucous | Pit mucous | COX7B | 0.36 | 7.60E-122 |
| Pit mucous | Pit mucous | FKBP11 | -0.3 | 6.92E-121 |
| Pit mucous | Pit mucous | RPS15 | 0.25 | 1.47E-120 |
| Pit mucous | Pit mucous | SOD1 | 0.31 | 4.13E-119 |
| Pit mucous | Pit mucous | PRDX2 | 0.26 | 5.09E-119 |
| Pit mucous | Pit mucous | OCIAD2 | 0.28 | 8.38E-117 |
| Pit mucous | Pit mucous | KRT8 | 0.39 | 9.46E-116 |
| Pit mucous | Pit mucous | ATP5MC2 | 0.31 | 4.58E-115 |
| Pit mucous | Pit mucous | RPL36A | 0.33 | 9.12E-106 |
| Pit mucous | Pit mucous | MUC5AC | -1.79 | 1.51E-105 |
| Pit mucous | Pit mucous | MSMB | -1.71 | 1.58E-105 |
| Pit mucous | Pit mucous | RPS15A | 0.29 | 2.49E-103 |
| Pit mucous | Pit mucous | KRT19 | 0.46 | 5.55E-102 |
| Pit mucous | Pit mucous | RPL31 | 0.33 | 2.09E-99 |
| Pit mucous | Pit mucous | EIF4A1 | 0.28 | 4.57E-99 |
| Pit mucous | Pit mucous | TMSB4X | -0.81 | 3.77E-97 |
| Pit mucous | Pit mucous | RPL41 | 0.26 | 6.89E-97 |
| Pit mucous | Pit mucous | FABP1 | -0.86 | 4.69E-95 |
| Pit mucous | Pit mucous | ALDOB | -0.29 | 1.44E-94 |
| Pit mucous | Pit mucous | COX7C | 0.27 | 1.98E-94 |
| Pit mucous | Pit mucous | HLA-DRA | -0.39 | 3.15E-93 |
| Pit mucous | Pit mucous | CYCS | 0.27 | 9.48E-93 |
| Pit mucous | Pit mucous | RPS10 | 0.28 | 7.48E-90 |
| Pit mucous | Pit mucous | RPS25 | 0.26 | 2.57E-88 |
| Pit mucous | Pit mucous | ANPEP | -0.34 | 1.72E-87 |
| Pit mucous | Pit mucous | PFN1 | 0.29 | 2.75E-87 |
| Pit mucous | Pit mucous | RPL17 | 0.28 | 8.13E-87 |
| Pit mucous | Pit mucous | JPT1 | 0.25 | 9.87E-84 |
| Pit mucous | Pit mucous | CXCL8 | -0.41 | 6.76E-81 |
| Pit mucous | Pit mucous | IL32 | -0.29 | 3.59E-80 |
| Pit mucous | Pit mucous | IFITM1 | -0.37 | 1.16E-75 |
| Pit mucous | Pit mucous | ISG15 | -0.31 | 2.67E-67 |
| Pit mucous | Pit mucous | DGAT1 | -0.3 | 3.82E-65 |
| Pit mucous | Pit mucous | TMSB10 | -0.43 | 8.50E-64 |
| Pit mucous | Pit mucous | IFITM2 | -0.4 | 1.36E-63 |
| Pit mucous | Pit mucous | HIST1H4C | -0.34 | 6.09E-61 |
| Pit mucous | Pit mucous | CDHR5 | -0.28 | 4.47E-59 |
| Pit mucous | Pit mucous | NR4A2 | -0.26 | 2.68E-58 |
| Pit mucous | Pit mucous | RNASE1 | -1.24 | 9.65E-58 |
| Pit mucous | Pit mucous | PSCA | -1.73 | 7.15E-57 |
| Pit mucous | Pit mucous | PARD6B | -0.28 | 1.25E-56 |
| Pit mucous | Pit mucous | CLDN18 | -1 | 6.42E-56 |
| Pit mucous | Pit mucous | ARHGDIB | -0.35 | 1.92E-53 |
| Pit mucous | Pit mucous | KRT20 | -0.27 | 4.31E-53 |
| Pit mucous | Pit mucous | JCHAIN | -3.72 | 5.00E-53 |
| Pit mucous | Pit mucous | KLF6 | -0.56 | 6.60E-52 |
| Pit mucous | Pit mucous | CAPN8 | -0.26 | 3.56E-51 |
| Pit mucous | Pit mucous | RSRP1 | -0.25 | 1.24E-50 |
| Pit mucous | Pit mucous | CXCL3 | -0.34 | 3.11E-50 |
| Pit mucous | Pit mucous | MUCL3 | -1.2 | 4.40E-50 |
| Pit mucous | Pit mucous | SST | -2.52 | 2.18E-49 |
| Pit mucous | Pit mucous | TIMP1 | -0.29 | 4.46E-49 |
| Pit mucous | Pit mucous | ITM2C | -0.32 | 9.39E-49 |
| Pit mucous | Pit mucous | MXD1 | -0.25 | 2.74E-48 |
| Pit mucous | Pit mucous | MLPH | -0.26 | 3.40E-48 |
| Pit mucous | Pit mucous | CYP2S1 | -0.3 | 3.64E-47 |
| Pit mucous | Pit mucous | F2RL1 | -0.27 | 1.26E-46 |
| Pit mucous | Pit mucous | MUC1 | -1.12 | 1.06E-45 |
| Pit mucous | Pit mucous | UGDH | -0.27 | 1.67E-45 |
| Pit mucous | Pit mucous | SAT1 | -0.51 | 1.63E-44 |
| Pit mucous | Pit mucous | B2M | -0.56 | 1.80E-44 |
| Pit mucous | Pit mucous | MAFF | -0.28 | 6.61E-44 |
| Pit mucous | Pit mucous | GSTA1 | -0.29 | 1.10E-43 |
| Pit mucous | Pit mucous | NET1 | -0.25 | 1.23E-43 |
| Pit mucous | Pit mucous | ABLIM1 | -0.27 | 1.31E-43 |
| Pit mucous | Pit mucous | GNAQ | -0.26 | 3.94E-43 |
| Pit mucous | Pit mucous | VILL | -0.29 | 5.51E-43 |
| Pit mucous | Pit mucous | STMN1 | -0.26 | 6.99E-43 |
| Pit mucous | Pit mucous | B3GNT5 | -0.28 | 1.06E-42 |
| Pit mucous | Pit mucous | FOS | -1.09 | 1.43E-41 |
| Pit mucous | Pit mucous | CBR1 | -0.29 | 1.58E-41 |
| Pit mucous | Pit mucous | HEXIM1 | -0.26 | 4.07E-41 |
| Pit mucous | Pit mucous | RGS2 | -0.26 | 2.31E-40 |
| Pit mucous | Pit mucous | GCLC | -0.25 | 4.32E-40 |
| Pit mucous | Pit mucous | HLA-DPA1 | -0.32 | 7.82E-40 |
| Pit mucous | Pit mucous | HMGB2 | -0.29 | 2.09E-39 |
| Pit mucous | Pit mucous | CXCL2 | -0.52 | 2.30E-39 |
| Pit mucous | Pit mucous | FOSL2 | -0.27 | 2.60E-39 |
| Pit mucous | Pit mucous | GPBP1 | -0.27 | 4.00E-39 |
| Pit mucous | Pit mucous | TNFAIP3 | -0.33 | 4.56E-39 |
| Pit mucous | Pit mucous | PLXNB2 | -0.28 | 3.15E-38 |
| Pit mucous | Pit mucous | ZFP36L2 | -0.26 | 3.44E-38 |
| Pit mucous | Pit mucous | SELENOP | -0.39 | 1.02E-37 |
| Pit mucous | Pit mucous | FAM102A | -0.3 | 9.16E-37 |
| Pit mucous | Pit mucous | RASEF | -0.35 | 1.74E-36 |
| Pit mucous | Pit mucous | RIOK3 | -0.3 | 1.96E-36 |
| Pit mucous | Pit mucous | HLA-DPB1 | -0.42 | 2.30E-36 |
| Pit mucous | Pit mucous | VIM | -1.23 | 2.37E-36 |
| Pit mucous | Pit mucous | SLC9A1 | -0.25 | 2.81E-35 |
| Pit mucous | Pit mucous | FOXA3 | -0.28 | 1.85E-34 |
| Pit mucous | Pit mucous | HIGD1A | -0.25 | 1.12E-33 |
| Pit mucous | Pit mucous | AC020916.1 | -0.26 | 1.73E-33 |
| Pit mucous | Pit mucous | SYTL2 | -0.29 | 2.04E-33 |
| Pit mucous | Pit mucous | SH3BGRL2 | -0.28 | 1.27E-32 |
| Pit mucous | Pit mucous | TLE4 | -0.33 | 3.17E-32 |
| Pit mucous | Pit mucous | HSPA1B | -0.3 | 5.42E-32 |
| Pit mucous | Pit mucous | HSPA1A | -0.26 | 1.12E-31 |
| Pit mucous | Pit mucous | ANXA1 | -0.34 | 2.14E-31 |
| Pit mucous | Pit mucous | LASP1 | -0.3 | 3.61E-31 |
| Pit mucous | Pit mucous | WSB1 | -0.31 | 3.98E-31 |
| Pit mucous | Pit mucous | EFHD2 | -0.33 | 4.25E-31 |
| Pit mucous | Pit mucous | JUN | -0.94 | 4.57E-31 |
| Pit mucous | Pit mucous | SMIM22 | -0.62 | 5.79E-31 |
| Pit mucous | Pit mucous | SGSM3 | -0.28 | 1.53E-30 |
| Pit mucous | Pit mucous | MALAT1 | -0.71 | 2.87E-30 |
| Pit mucous | Pit mucous | DDX3X | -0.3 | 3.54E-30 |
| Pit mucous | Pit mucous | RAB27B | -0.3 | 3.70E-29 |
| Pit mucous | Pit mucous | CST3 | -0.42 | 4.90E-29 |
| Pit mucous | Pit mucous | SGK1 | -0.31 | 6.04E-29 |
| Pit mucous | Pit mucous | CSRNP1 | -0.3 | 1.43E-28 |
| Pit mucous | Pit mucous | SRGN | -1.05 | 7.58E-28 |
| Pit mucous | Pit mucous | C11orf86 | -0.31 | 1.63E-27 |
| Pit mucous | Pit mucous | ID2 | -0.34 | 1.80E-27 |
| Pit mucous | Pit mucous | BRI3 | -0.28 | 2.60E-27 |
| Pit mucous | Pit mucous | PTP4A1 | -0.27 | 2.65E-27 |
| Pit mucous | Pit mucous | TRAM1 | -0.29 | 6.06E-27 |
| Pit mucous | Pit mucous | C12orf75 | -0.32 | 8.05E-27 |
| Pit mucous | Pit mucous | SVIP | -0.31 | 1.72E-26 |
| Pit mucous | Pit mucous | TAGLN2 | -0.57 | 2.27E-26 |
| Pit mucous | Pit mucous | FOSB | -0.87 | 2.86E-26 |
| Pit mucous | Pit mucous | DDIT3 | -0.26 | 3.82E-26 |
| Pit mucous | Pit mucous | LGALS1 | -1.32 | 4.27E-26 |
| Pit mucous | Pit mucous | S100A4 | -0.82 | 5.66E-26 |
| Pit mucous | Pit mucous | PSAPL1 | -0.54 | 8.73E-25 |
| Pit mucous | Pit mucous | RUNX1 | -0.26 | 2.37E-24 |
| Pit mucous | Pit mucous | KRTCAP2 | -0.28 | 3.75E-24 |
| Pit mucous | Pit mucous | CREM | -0.47 | 4.50E-24 |
| Pit mucous | Pit mucous | AKR1C1 | -0.33 | 5.53E-24 |
| Pit mucous | Pit mucous | GALNT6 | -0.31 | 6.32E-24 |
| Pit mucous | Pit mucous | SNHG9 | -0.31 | 4.08E-23 |
| Pit mucous | Pit mucous | PPDPF | -0.42 | 5.31E-23 |
| Pit mucous | Pit mucous | ARF6 | -0.28 | 1.41E-22 |
| Pit mucous | Pit mucous | KRT10 | -0.25 | 1.55E-22 |
| Pit mucous | Pit mucous | ITPKC | -0.31 | 2.31E-22 |
| Pit mucous | Pit mucous | CXCL17 | -0.74 | 3.58E-22 |
| Pit mucous | Pit mucous | TRA2B | -0.27 | 5.69E-22 |
| Pit mucous | Pit mucous | ETNK1 | -0.33 | 6.71E-22 |
| Pit mucous | Pit mucous | DNAJB9 | -0.38 | 6.96E-22 |
| Pit mucous | Pit mucous | LMO4 | -0.27 | 9.39E-22 |
| Pit mucous | Pit mucous | IER2 | -0.74 | 4.46E-21 |
| Pit mucous | Pit mucous | SEC62 | -0.29 | 4.72E-21 |
| Pit mucous | Pit mucous | FOXQ1 | -0.47 | 5.18E-21 |
| Pit mucous | Pit mucous | NAP1L1 | -0.29 | 5.66E-21 |
| Pit mucous | Pit mucous | PMP22 | -0.25 | 6.91E-21 |
| Pit mucous | Pit mucous | IDS | -0.28 | 1.21E-20 |
| Pit mucous | Pit mucous | UBB | -0.3 | 2.65E-20 |
| Pit mucous | Pit mucous | DDX5 | -0.4 | 5.23E-20 |
| Pit mucous | Pit mucous | TENT5A | -0.37 | 1.07E-19 |
| Pit mucous | Pit mucous | CYSTM1 | -0.41 | 1.37E-19 |
| Pit mucous | Pit mucous | TXNIP | -0.36 | 1.57E-19 |
| Pit mucous | Pit mucous | CTSE | -0.6 | 3.22E-19 |
| Pit mucous | Pit mucous | ID3 | -0.34 | 5.33E-19 |
| Pit mucous | Pit mucous | VAMP2 | -0.39 | 6.09E-19 |
| Pit mucous | Pit mucous | SEC61G | -0.41 | 8.33E-19 |
| Pit mucous | Pit mucous | NFE2L2 | -0.35 | 8.64E-19 |
| Pit mucous | Pit mucous | LIPF | -1.53 | 3.61E-18 |
| Pit mucous | Pit mucous | RAP2B | -0.36 | 4.13E-18 |
| Pit mucous | Pit mucous | DDX17 | -0.36 | 4.79E-18 |
| Pit mucous | Pit mucous | ZFP36L1 | -0.33 | 1.30E-17 |
| Pit mucous | Pit mucous | CALM2 | -0.36 | 1.34E-17 |
| Pit mucous | Pit mucous | RGS1 | -0.91 | 1.53E-17 |
| Pit mucous | Pit mucous | JAG1 | -0.29 | 1.55E-17 |
| Pit mucous | Pit mucous | TM4SF4 | -0.33 | 1.85E-17 |
| Pit mucous | Pit mucous | RBM47 | -0.27 | 1.89E-17 |
| Pit mucous | Pit mucous | B4GALT1 | -0.3 | 2.92E-17 |
| Pit mucous | Pit mucous | ZFP36 | -1.04 | 2.95E-17 |
| Pit mucous | Pit mucous | UBC | -0.33 | 4.81E-17 |
| Pit mucous | Pit mucous | XBP1 | -0.27 | 5.73E-17 |
| Pit mucous | Pit mucous | PCSK1N | -1.03 | 6.23E-17 |
| Pit mucous | Pit mucous | LMO7 | -0.27 | 9.13E-17 |
| Pit mucous | Pit mucous | YOD1 | -0.35 | 1.93E-16 |
| Pit mucous | Pit mucous | VEGFA | -0.27 | 1.98E-16 |
| Pit mucous | Pit mucous | KLF10 | -0.34 | 3.26E-16 |
| Pit mucous | Pit mucous | UBL3 | -0.36 | 3.91E-16 |
| Pit mucous | Pit mucous | YWHAH | -0.37 | 6.68E-16 |
| Pit mucous | Pit mucous | CDKN1A | -0.3 | 8.34E-16 |
| Pit mucous | Pit mucous | HNRNPH1 | -0.38 | 9.00E-16 |
| Pit mucous | Pit mucous | RBP2 | -1.15 | 1.21E-15 |
| Pit mucous | Pit mucous | DHCR24 | -0.27 | 1.26E-15 |
| Pit mucous | Pit mucous | OASL | -0.27 | 1.98E-15 |
| Pit mucous | Pit mucous | TMBIM6 | -0.3 | 3.47E-15 |
| Pit mucous | Pit mucous | SCGB2A1 | -0.57 | 6.85E-15 |
| Pit mucous | Pit mucous | CLK1 | -0.38 | 9.43E-15 |
| Pit mucous | Pit mucous | JUNB | -1.02 | 9.80E-15 |
| Pit mucous | Pit mucous | SLPI | -0.35 | 1.02E-14 |
| Pit mucous | Pit mucous | STARD10 | -0.29 | 1.10E-14 |
| Pit mucous | Pit mucous | APOA1 | -1.13 | 1.31E-14 |
| Pit mucous | Pit mucous | BCAS1 | -0.37 | 3.21E-14 |
| Pit mucous | Pit mucous | SPINK1 | -0.76 | 1.34E-13 |
| Pit mucous | Pit mucous | LAPTM4A | -0.31 | 2.70E-13 |
| Pit mucous | Pit mucous | DUSP1 | -0.89 | 2.70E-13 |
| Pit mucous | Pit mucous | SERTAD1 | -0.26 | 5.25E-13 |
| Pit mucous | Pit mucous | SRSF5 | -0.3 | 5.38E-13 |
| Pit mucous | Pit mucous | IRF1 | -0.44 | 6.75E-13 |
| Pit mucous | Pit mucous | NAMPT | -0.34 | 9.85E-13 |
| Pit mucous | Pit mucous | ARGLU1 | -0.34 | 1.30E-12 |
| Pit mucous | Pit mucous | ZG16 | -0.36 | 1.94E-12 |
| Pit mucous | Pit mucous | MAL2 | -0.33 | 2.81E-12 |
| Pit mucous | Pit mucous | SDC4 | -0.39 | 3.54E-12 |
| Pit mucous | Pit mucous | MTTP | -0.28 | 4.30E-12 |
| Pit mucous | Pit mucous | NFKBIZ | -0.31 | 9.01E-12 |
| Pit mucous | Pit mucous | AADAC | -0.33 | 1.84E-11 |
| Pit mucous | Pit mucous | MUC6 | -0.95 | 2.68E-11 |
| Pit mucous | Pit mucous | PNRC1 | -0.42 | 3.54E-11 |
| Pit mucous | Pit mucous | ID1 | -0.32 | 3.77E-11 |
| Pit mucous | Pit mucous | LDLR | -0.33 | 1.13E-10 |
| Pit mucous | Pit mucous | AAMDC | -0.37 | 2.76E-10 |
| Pit mucous | Pit mucous | TM9SF3 | -0.28 | 3.93E-10 |
| Pit mucous | Pit mucous | CA2 | -0.7 | 5.41E-10 |
| Pit mucous | Pit mucous | BIRC3 | -0.36 | 5.57E-10 |
| Pit mucous | Pit mucous | MS4A8 | -0.28 | 9.16E-10 |
| Pit mucous | Pit mucous | SLC39A11 | -0.3 | 9.64E-10 |
| Pit mucous | Pit mucous | SIK1 | -0.44 | 1.38E-09 |
| Pit mucous | Pit mucous | VAPA | -0.25 | 1.40E-09 |
| Pit mucous | Pit mucous | FAR1 | -0.3 | 1.57E-09 |
| Pit mucous | Pit mucous | MT-ND4L | -0.4 | 2.32E-09 |
| Pit mucous | Pit mucous | TACSTD2 | -0.31 | 2.43E-09 |
| Pit mucous | Pit mucous | RAB11FIP1 | -0.31 | 3.83E-09 |
| Pit mucous | Pit mucous | ANKRD36C | -0.29 | 4.41E-09 |
| Pit mucous | Pit mucous | DAZAP2 | -0.28 | 7.96E-09 |
| Pit mucous | Pit mucous | HMGCS1 | -0.4 | 9.07E-09 |
| Pit mucous | Pit mucous | HLA-E | -0.32 | 1.48E-08 |
| Pit mucous | Pit mucous | HMGN2 | -0.27 | 1.87E-08 |
| Pit mucous | Pit mucous | VSIG1 | -0.6 | 2.09E-08 |
| Pit mucous | Pit mucous | KLF4 | -0.36 | 2.30E-08 |
| Pit mucous | Pit mucous | CLU | -0.55 | 3.95E-08 |
| Pit mucous | Pit mucous | TUBA1A | -0.71 | 8.13E-08 |
| Pit mucous | Pit mucous | SPCS2 | -0.37 | 9.88E-08 |
| Pit mucous | Pit mucous | FAM107B | -0.39 | 2.39E-07 |
| Pit mucous | Pit mucous | HSPB1 | -0.42 | 2.56E-07 |
| Pit mucous | Pit mucous | BHLHE40 | -0.37 | 2.82E-07 |
| Pit mucous | Pit mucous | OTUD1 | -0.29 | 3.14E-07 |
| Pit mucous | Pit mucous | RARRES2 | -0.44 | 4.77E-07 |
| Pit mucous | Pit mucous | S100P | -0.65 | 7.64E-07 |
| Pit mucous | Pit mucous | PRDX5 | -0.26 | 8.66E-07 |
| Pit mucous | Pit mucous | PDK4 | -0.28 | 9.82E-07 |
| Pit mucous | Pit mucous | MMP1 | -0.55 | 1.17E-06 |
| Pit mucous | Pit mucous | MORF4L1 | -0.29 | 1.49E-06 |
| Pit mucous | Pit mucous | RGCC | -0.36 | 1.79E-06 |
| Pit mucous | Pit mucous | CYTOR | -0.36 | 2.19E-06 |
| Pit mucous | Pit mucous | LAMB3 | -0.49 | 2.39E-06 |
| Pit mucous | Pit mucous | UGCG | -0.47 | 3.10E-06 |
| Pit mucous | Pit mucous | YPEL5 | -0.55 | 3.26E-06 |
| Pit mucous | Pit mucous | XIST | -0.27 | 6.38E-06 |
| Pit mucous | Pit mucous | ATF3 | -0.63 | 9.28E-06 |
| Fibroblasts | Fibroblasts | CXCL14 | 4.33 | 0 |
| Fibroblasts | Fibroblasts | LUM | 3.58 | 0 |
| Fibroblasts | Fibroblasts | DCN | 3.51 | 0 |
| Fibroblasts | Fibroblasts | POSTN | 3.17 | 0 |
| Fibroblasts | Fibroblasts | CCL11 | 3.03 | 0 |
| Fibroblasts | Fibroblasts | APOD | 2.9 | 0 |
| Fibroblasts | Fibroblasts | CFD | 2.88 | 0 |
| Fibroblasts | Fibroblasts | MFAP4 | 2.84 | 0 |
| Fibroblasts | Fibroblasts | LGALS1 | 2.76 | 0 |
| Fibroblasts | Fibroblasts | IGFBP5 | 2.76 | 0 |
| Fibroblasts | Fibroblasts | RARRES2 | 2.69 | 0 |
| Fibroblasts | Fibroblasts | PLAT | 2.67 | 0 |
| Fibroblasts | Fibroblasts | COL3A1 | 2.64 | 0 |
| Fibroblasts | Fibroblasts | CCL2 | 2.62 | 0 |
| Fibroblasts | Fibroblasts | CALD1 | 2.54 | 0 |
| Fibroblasts | Fibroblasts | COL6A2 | 2.49 | 0 |
| Fibroblasts | Fibroblasts | C1S | 2.39 | 0 |
| Fibroblasts | Fibroblasts | DPT | 2.38 | 0 |
| Fibroblasts | Fibroblasts | TMEM176B | 2.33 | 0 |
| Fibroblasts | Fibroblasts | PTGDS | 2.28 | 0 |
| Fibroblasts | Fibroblasts | COL1A2 | 2.27 | 0 |
| Fibroblasts | Fibroblasts | SERPINF1 | 2.23 | 0 |
| Fibroblasts | Fibroblasts | MMP2 | 2.16 | 0 |
| Fibroblasts | Fibroblasts | JUNB | 2.14 | 0 |
| Fibroblasts | Fibroblasts | COL1A1 | 2.1 | 0 |
| Fibroblasts | Fibroblasts | C1R | 2.09 | 0 |
| Fibroblasts | Fibroblasts | BMP4 | 2.07 | 0 |
| Fibroblasts | Fibroblasts | SERPING1 | 2.04 | 0 |
| Fibroblasts | Fibroblasts | COL6A1 | 1.93 | 0 |
| Fibroblasts | Fibroblasts | APOE | 1.92 | 0 |
| Fibroblasts | Fibroblasts | TMEM176A | 1.91 | 0 |
| Fibroblasts | Fibroblasts | VIM | 1.91 | 0 |
| Fibroblasts | Fibroblasts | AGT | 1.87 | 0 |
| Fibroblasts | Fibroblasts | SPON2 | 1.87 | 0 |
| Fibroblasts | Fibroblasts | IFITM3 | 1.85 | 0 |
| Fibroblasts | Fibroblasts | PDGFRA | 1.85 | 0 |
| Fibroblasts | Fibroblasts | GADD45B | 1.84 | 0 |
| Fibroblasts | Fibroblasts | SPARCL1 | 1.82 | 0 |
| Fibroblasts | Fibroblasts | IGFBP6 | 1.77 | 0 |
| Fibroblasts | Fibroblasts | SDC2 | 1.76 | 0 |
| Fibroblasts | Fibroblasts | JUN | 1.76 | 0 |
| Fibroblasts | Fibroblasts | ZFP36 | 1.76 | 0 |
| Fibroblasts | Fibroblasts | TIMP1 | 1.75 | 0 |
| Fibroblasts | Fibroblasts | SPARC | 1.74 | 0 |
| Fibroblasts | Fibroblasts | ID3 | 1.73 | 0 |
| Fibroblasts | Fibroblasts | FOS | 1.72 | 0 |
| Fibroblasts | Fibroblasts | IFITM1 | 1.72 | 0 |
| Fibroblasts | Fibroblasts | LSP1 | 1.71 | 0 |
| Fibroblasts | Fibroblasts | IGFBP3 | 1.68 | 0 |
| Fibroblasts | Fibroblasts | MEG3 | 1.67 | 0 |
| Fibroblasts | Fibroblasts | TPM2 | 1.67 | 0 |
| Fibroblasts | Fibroblasts | HSPA1A | 1.66 | 0 |
| Fibroblasts | Fibroblasts | CYR61 | 1.66 | 0 |
| Fibroblasts | Fibroblasts | TAGLN | 1.65 | 0 |
| Fibroblasts | Fibroblasts | NNMT | 1.65 | 0 |
| Fibroblasts | Fibroblasts | SELENOM | 1.6 | 0 |
| Fibroblasts | Fibroblasts | TUBA1A | 1.59 | 0 |
| Fibroblasts | Fibroblasts | MFGE8 | 1.59 | 0 |
| Fibroblasts | Fibroblasts | PTN | 1.58 | 0 |
| Fibroblasts | Fibroblasts | PCOLCE | 1.58 | 0 |
| Fibroblasts | Fibroblasts | CTSC | 1.56 | 0 |
| Fibroblasts | Fibroblasts | RGS10 | 1.56 | 0 |
| Fibroblasts | Fibroblasts | GPX3 | 1.55 | 0 |
| Fibroblasts | Fibroblasts | TCF21 | 1.55 | 0 |
| Fibroblasts | Fibroblasts | CFH | 1.54 | 0 |
| Fibroblasts | Fibroblasts | SOD3 | 1.51 | 0 |
| Fibroblasts | Fibroblasts | PLAC9 | 1.51 | 0 |
| Fibroblasts | Fibroblasts | NSG1 | 1.51 | 0 |
| Fibroblasts | Fibroblasts | IGFBP7 | 1.49 | 0 |
| Fibroblasts | Fibroblasts | FBLN1 | 1.49 | 0 |
| Fibroblasts | Fibroblasts | VCAN | 1.49 | 0 |
| Fibroblasts | Fibroblasts | SOCS3 | 1.48 | 0 |
| Fibroblasts | Fibroblasts | PPP1R14A | 1.48 | 0 |
| Fibroblasts | Fibroblasts | MYL9 | 1.47 | 0 |
| Fibroblasts | Fibroblasts | TGFBI | 1.46 | 0 |
| Fibroblasts | Fibroblasts | ABCA8 | 1.42 | 0 |
| Fibroblasts | Fibroblasts | S100A4 | 1.42 | 0 |
| Fibroblasts | Fibroblasts | LAPTM4A | 1.4 | 0 |
| Fibroblasts | Fibroblasts | LTBP4 | 1.38 | 0 |
| Fibroblasts | Fibroblasts | BST2 | 1.37 | 0 |
| Fibroblasts | Fibroblasts | NUPR1 | 1.34 | 0 |
| Fibroblasts | Fibroblasts | OLFML3 | 1.34 | 0 |
| Fibroblasts | Fibroblasts | GADD45G | 1.34 | 0 |
| Fibroblasts | Fibroblasts | CAV1 | 1.33 | 0 |
| Fibroblasts | Fibroblasts | FHL1 | 1.32 | 0 |
| Fibroblasts | Fibroblasts | A2M | 1.31 | 0 |
| Fibroblasts | Fibroblasts | SPRY1 | 1.28 | 0 |
| Fibroblasts | Fibroblasts | RBP1 | 1.28 | 0 |
| Fibroblasts | Fibroblasts | CTSK | 1.27 | 0 |
| Fibroblasts | Fibroblasts | S100A13 | 1.25 | 0 |
| Fibroblasts | Fibroblasts | VSTM2A | 1.25 | 0 |
| Fibroblasts | Fibroblasts | CYGB | 1.22 | 0 |
| Fibroblasts | Fibroblasts | GNG11 | 1.22 | 0 |
| Fibroblasts | Fibroblasts | LHFPL6 | 1.21 | 0 |
| Fibroblasts | Fibroblasts | ENHO | 1.2 | 0 |
| Fibroblasts | Fibroblasts | CRISPLD2 | 1.2 | 0 |
| Fibroblasts | Fibroblasts | EMP3 | 1.18 | 0 |
| Fibroblasts | Fibroblasts | TCF4 | 1.18 | 0 |
| Fibroblasts | Fibroblasts | PLPP3 | 1.17 | 0 |
| Fibroblasts | Fibroblasts | COX7A1 | 1.16 | 0 |
| Fibroblasts | Fibroblasts | MGP | 1.16 | 0 |
| Fibroblasts | Fibroblasts | PKIG | 1.16 | 0 |
| Fibroblasts | Fibroblasts | TFPI | 1.15 | 0 |
| Fibroblasts | Fibroblasts | EID1 | 1.15 | 0 |
| Fibroblasts | Fibroblasts | LAMA4 | 1.15 | 0 |
| Fibroblasts | Fibroblasts | EDNRB | 1.15 | 0 |
| Fibroblasts | Fibroblasts | SLC2A3 | 1.15 | 0 |
| Fibroblasts | Fibroblasts | ASPN | 1.15 | 0 |
| Fibroblasts | Fibroblasts | TSPAN4 | 1.15 | 0 |
| Fibroblasts | Fibroblasts | SGCE | 1.14 | 0 |
| Fibroblasts | Fibroblasts | SCPEP1 | 1.14 | 0 |
| Fibroblasts | Fibroblasts | PTGS1 | 1.14 | 0 |
| Fibroblasts | Fibroblasts | FXYD1 | 1.13 | 0 |
| Fibroblasts | Fibroblasts | AC245595.1 | 1.13 | 0 |
| Fibroblasts | Fibroblasts | PLPP1 | 1.12 | 0 |
| Fibroblasts | Fibroblasts | FENDRR | 1.11 | 0 |
| Fibroblasts | Fibroblasts | TXNIP | 1.11 | 0 |
| Fibroblasts | Fibroblasts | IFITM2 | 1.1 | 0 |
| Fibroblasts | Fibroblasts | EFEMP2 | 1.09 | 0 |
| Fibroblasts | Fibroblasts | ACTA2 | 1.07 | 0 |
| Fibroblasts | Fibroblasts | COL5A1 | 1.07 | 0 |
| Fibroblasts | Fibroblasts | NPY | 1.07 | 0 |
| Fibroblasts | Fibroblasts | FBLN5 | 1.06 | 0 |
| Fibroblasts | Fibroblasts | PLAU | 1.06 | 0 |
| Fibroblasts | Fibroblasts | ECM1 | 1.04 | 0 |
| Fibroblasts | Fibroblasts | EMILIN1 | 1.04 | 0 |
| Fibroblasts | Fibroblasts | CCDC80 | 1.04 | 0 |
| Fibroblasts | Fibroblasts | CPM | 1.03 | 0 |
| Fibroblasts | Fibroblasts | PMP22 | 1.03 | 0 |
| Fibroblasts | Fibroblasts | C11orf96 | 1.03 | 0 |
| Fibroblasts | Fibroblasts | PLTP | 1.02 | 0 |
| Fibroblasts | Fibroblasts | EFEMP1 | 1.02 | 0 |
| Fibroblasts | Fibroblasts | CLEC11A | 1.01 | 0 |
| Fibroblasts | Fibroblasts | CAVIN3 | 1.01 | 0 |
| Fibroblasts | Fibroblasts | COL6A3 | 1 | 0 |
| Fibroblasts | Fibroblasts | ACP5 | 1 | 0 |
| Fibroblasts | Fibroblasts | TMEM100 | 0.99 | 0 |
| Fibroblasts | Fibroblasts | PDGFD | 0.99 | 0 |
| Fibroblasts | Fibroblasts | DKK3 | 0.99 | 0 |
| Fibroblasts | Fibroblasts | AEBP1 | 0.99 | 0 |
| Fibroblasts | Fibroblasts | CXCL12 | 0.98 | 0 |
| Fibroblasts | Fibroblasts | PROCR | 0.98 | 0 |
| Fibroblasts | Fibroblasts | FRZB | 0.96 | 0 |
| Fibroblasts | Fibroblasts | EDIL3 | 0.96 | 0 |
| Fibroblasts | Fibroblasts | THY1 | 0.95 | 0 |
| Fibroblasts | Fibroblasts | GGT5 | 0.95 | 0 |
| Fibroblasts | Fibroblasts | AKR1B1 | 0.95 | 0 |
| Fibroblasts | Fibroblasts | F2R | 0.94 | 0 |
| Fibroblasts | Fibroblasts | SERPINH1 | 0.93 | 0 |
| Fibroblasts | Fibroblasts | SNAI2 | 0.92 | 0 |
| Fibroblasts | Fibroblasts | LINC01082 | 0.92 | 0 |
| Fibroblasts | Fibroblasts | MXRA8 | 0.92 | 0 |
| Fibroblasts | Fibroblasts | FXYD6 | 0.91 | 0 |
| Fibroblasts | Fibroblasts | LTBP1 | 0.91 | 0 |
| Fibroblasts | Fibroblasts | TMEM119 | 0.91 | 0 |
| Fibroblasts | Fibroblasts | PDPN | 0.9 | 0 |
| Fibroblasts | Fibroblasts | PTCH1 | 0.9 | 0 |
| Fibroblasts | Fibroblasts | FKBP10 | 0.89 | 0 |
| Fibroblasts | Fibroblasts | TSHZ2 | 0.89 | 0 |
| Fibroblasts | Fibroblasts | FSTL1 | 0.88 | 0 |
| Fibroblasts | Fibroblasts | TIMP2 | 0.87 | 0 |
| Fibroblasts | Fibroblasts | COL5A2 | 0.84 | 0 |
| Fibroblasts | Fibroblasts | VAMP5 | 0.83 | 0 |
| Fibroblasts | Fibroblasts | RAB34 | 0.81 | 0 |
| Fibroblasts | Fibroblasts | NR2F1 | 0.81 | 0 |
| Fibroblasts | Fibroblasts | CAVIN1 | 0.81 | 0 |
| Fibroblasts | Fibroblasts | MMP3 | 0.8 | 0 |
| Fibroblasts | Fibroblasts | PTGER2 | 0.79 | 0 |
| Fibroblasts | Fibroblasts | FOXF1 | 0.79 | 0 |
| Fibroblasts | Fibroblasts | AXL | 0.78 | 0 |
| Fibroblasts | Fibroblasts | PTGS2 | 0.78 | 0 |
| Fibroblasts | Fibroblasts | HAPLN1 | 0.77 | 0 |
| Fibroblasts | Fibroblasts | TGFB1I1 | 0.76 | 0 |
| Fibroblasts | Fibroblasts | NOVA1 | 0.76 | 0 |
| Fibroblasts | Fibroblasts | FILIP1L | 0.76 | 0 |
| Fibroblasts | Fibroblasts | MXRA5 | 0.76 | 0 |
| Fibroblasts | Fibroblasts | TRPA1 | 0.73 | 0 |
| Fibroblasts | Fibroblasts | TIMP3 | 0.72 | 0 |
| Fibroblasts | Fibroblasts | DIO2 | 0.72 | 0 |
| Fibroblasts | Fibroblasts | NID1 | 0.71 | 0 |
| Fibroblasts | Fibroblasts | SRPX | 0.7 | 0 |
| Fibroblasts | Fibroblasts | PDLIM3 | 0.69 | 0 |
| Fibroblasts | Fibroblasts | AKAP12 | 0.68 | 0 |
| Fibroblasts | Fibroblasts | TMEM204 | 0.67 | 0 |
| Fibroblasts | Fibroblasts | SOX6 | 0.67 | 0 |
| Fibroblasts | Fibroblasts | EMID1 | 0.67 | 0 |
| Fibroblasts | Fibroblasts | IL1R1 | 0.66 | 0 |
| Fibroblasts | Fibroblasts | TUSC3 | 0.66 | 0 |
| Fibroblasts | Fibroblasts | LOXL2 | 0.64 | 0 |
| Fibroblasts | Fibroblasts | GLT8D2 | 0.64 | 0 |
| Fibroblasts | Fibroblasts | PAPPA | 0.64 | 0 |
| Fibroblasts | Fibroblasts | COL15A1 | 0.64 | 0 |
| Fibroblasts | Fibroblasts | C2 | 0.64 | 0 |
| Fibroblasts | Fibroblasts | MSC | 0.64 | 0 |
| Fibroblasts | Fibroblasts | TCEAL7 | 0.63 | 0 |
| Fibroblasts | Fibroblasts | COL18A1 | 0.63 | 0 |
| Fibroblasts | Fibroblasts | BMP5 | 0.63 | 0 |
| Fibroblasts | Fibroblasts | MYLK | 0.62 | 0 |
| Fibroblasts | Fibroblasts | HTRA1 | 0.62 | 0 |
| Fibroblasts | Fibroblasts | TNXB | 0.62 | 0 |
| Fibroblasts | Fibroblasts | HACD4 | 0.61 | 0 |
| Fibroblasts | Fibroblasts | PRR16 | 0.61 | 0 |
| Fibroblasts | Fibroblasts | ANGPTL2 | 0.61 | 0 |
| Fibroblasts | Fibroblasts | FKBP7 | 0.6 | 0 |
| Fibroblasts | Fibroblasts | FN1 | 0.6 | 0 |
| Fibroblasts | Fibroblasts | MAMDC2 | 0.6 | 0 |
| Fibroblasts | Fibroblasts | BGN | 0.6 | 0 |
| Fibroblasts | Fibroblasts | CNRIP1 | 0.58 | 0 |
| Fibroblasts | Fibroblasts | CRYAB | 0.58 | 0 |
| Fibroblasts | Fibroblasts | FGF7 | 0.58 | 0 |
| Fibroblasts | Fibroblasts | C3 | 0.58 | 0 |
| Fibroblasts | Fibroblasts | PXDN | 0.57 | 0 |
| Fibroblasts | Fibroblasts | FAM92A | 0.57 | 0 |
| Fibroblasts | Fibroblasts | CDH11 | 0.57 | 0 |
| Fibroblasts | Fibroblasts | PCDH18 | 0.56 | 0 |
| Fibroblasts | Fibroblasts | PRKG1 | 0.56 | 0 |
| Fibroblasts | Fibroblasts | FIBIN | 0.56 | 0 |
| Fibroblasts | Fibroblasts | RCSD1 | 0.55 | 0 |
| Fibroblasts | Fibroblasts | NRG1 | 0.53 | 0 |
| Fibroblasts | Fibroblasts | CPED1 | 0.53 | 0 |
| Fibroblasts | Fibroblasts | PLXDC2 | 0.53 | 0 |
| Fibroblasts | Fibroblasts | SGCA | 0.52 | 0 |
| Fibroblasts | Fibroblasts | COL12A1 | 0.52 | 0 |
| Fibroblasts | Fibroblasts | C5orf66-AS1 | 0.52 | 0 |
| Fibroblasts | Fibroblasts | VCAM1 | 0.51 | 0 |
| Fibroblasts | Fibroblasts | COL4A5 | 0.51 | 0 |
| Fibroblasts | Fibroblasts | COL4A6 | 0.51 | 0 |
| Fibroblasts | Fibroblasts | NEXN | 0.51 | 0 |
| Fibroblasts | Fibroblasts | ABI3BP | 0.51 | 0 |
| Fibroblasts | Fibroblasts | MRC2 | 0.5 | 0 |
| Fibroblasts | Fibroblasts | FERMT2 | 0.5 | 0 |
| Fibroblasts | Fibroblasts | GJA1 | 0.5 | 0 |
| Fibroblasts | Fibroblasts | NDUFA4L2 | 0.5 | 0 |
| Fibroblasts | Fibroblasts | RBMS3 | 0.5 | 0 |
| Fibroblasts | Fibroblasts | INSC | 0.5 | 0 |
| Fibroblasts | Fibroblasts | ANTXR1 | 0.5 | 0 |
| Fibroblasts | Fibroblasts | FGFR1 | 0.5 | 0 |
| Fibroblasts | Fibroblasts | PTGES | 0.49 | 0 |
| Fibroblasts | Fibroblasts | SYNPO2 | 0.49 | 0 |
| Fibroblasts | Fibroblasts | ALDH1A3 | 0.49 | 0 |
| Fibroblasts | Fibroblasts | GPC6 | 0.49 | 0 |
| Fibroblasts | Fibroblasts | DDR2 | 0.49 | 0 |
| Fibroblasts | Fibroblasts | DLL1 | 0.48 | 0 |
| Fibroblasts | Fibroblasts | ISLR | 0.47 | 0 |
| Fibroblasts | Fibroblasts | WFDC1 | 0.47 | 0 |
| Fibroblasts | Fibroblasts | LTBP2 | 0.47 | 0 |
| Fibroblasts | Fibroblasts | ANKRD35 | 0.46 | 0 |
| Fibroblasts | Fibroblasts | ZNF667-AS1 | 0.46 | 0 |
| Fibroblasts | Fibroblasts | DSE | 0.45 | 0 |
| Fibroblasts | Fibroblasts | ADGRL3 | 0.44 | 0 |
| Fibroblasts | Fibroblasts | RCAN2 | 0.44 | 0 |
| Fibroblasts | Fibroblasts | FBN1 | 0.44 | 0 |
| Fibroblasts | Fibroblasts | GFRA1 | 0.44 | 0 |
| Fibroblasts | Fibroblasts | NDN | 0.43 | 0 |
| Fibroblasts | Fibroblasts | RCN3 | 0.43 | 0 |
| Fibroblasts | Fibroblasts | NEGR1 | 0.43 | 0 |
| Fibroblasts | Fibroblasts | GPR68 | 0.43 | 0 |
| Fibroblasts | Fibroblasts | TMEM47 | 0.43 | 0 |
| Fibroblasts | Fibroblasts | BICC1 | 0.43 | 0 |
| Fibroblasts | Fibroblasts | HGF | 0.42 | 0 |
| Fibroblasts | Fibroblasts | TBX2-AS1 | 0.42 | 0 |
| Fibroblasts | Fibroblasts | ADAMTSL1 | 0.41 | 0 |
| Fibroblasts | Fibroblasts | LSAMP | 0.41 | 0 |
| Fibroblasts | Fibroblasts | CERCAM | 0.4 | 0 |
| Fibroblasts | Fibroblasts | MRVI1 | 0.4 | 0 |
| Fibroblasts | Fibroblasts | WNT5A | 0.39 | 0 |
| Fibroblasts | Fibroblasts | COLEC11 | 0.39 | 0 |
| Fibroblasts | Fibroblasts | MMP11 | 0.39 | 0 |
| Fibroblasts | Fibroblasts | PLEKHH2 | 0.38 | 0 |
| Fibroblasts | Fibroblasts | PDE1A | 0.37 | 0 |
| Fibroblasts | Fibroblasts | PDZRN3 | 0.37 | 0 |
| Fibroblasts | Fibroblasts | NTF3 | 0.37 | 0 |
| Fibroblasts | Fibroblasts | FOXF2 | 0.36 | 0 |
| Fibroblasts | Fibroblasts | SLITRK6 | 0.36 | 0 |
| Fibroblasts | Fibroblasts | PAMR1 | 0.36 | 0 |
| Fibroblasts | Fibroblasts | PRKAR2B | 0.36 | 0 |
| Fibroblasts | Fibroblasts | LOX | 0.35 | 0 |
| Fibroblasts | Fibroblasts | BARX1 | 0.34 | 0 |
| Fibroblasts | Fibroblasts | FRMD6 | 0.34 | 0 |
| Fibroblasts | Fibroblasts | PTGDR2 | 0.34 | 0 |
| Fibroblasts | Fibroblasts | MRGPRF | 0.34 | 0 |
| Fibroblasts | Fibroblasts | OLFML1 | 0.33 | 0 |
| Fibroblasts | Fibroblasts | MMP19 | 0.32 | 0 |
| Fibroblasts | Fibroblasts | SHISA4 | 0.32 | 0 |
| Fibroblasts | Fibroblasts | FEZ1 | 0.32 | 0 |
| Fibroblasts | Fibroblasts | SRPX2 | 0.32 | 0 |
| Fibroblasts | Fibroblasts | MT-CO3 | -1.24 | 0 |
| Fibroblasts | Fibroblasts | ELF3 | -1.43 | 0 |
| Fibroblasts | Fibroblasts | SMIM22 | -1.5 | 0 |
| Fibroblasts | Fibroblasts | FXYD3 | -1.57 | 0 |
| Fibroblasts | Fibroblasts | C19orf33 | -1.59 | 0 |
| Fibroblasts | Fibroblasts | KRT18 | -1.62 | 0 |
| Fibroblasts | Fibroblasts | CYSTM1 | -1.66 | 0 |
| Fibroblasts | Fibroblasts | KRT8 | -1.81 | 0 |
| Fibroblasts | Fibroblasts | LYZ | -1.81 | 0 |
| Fibroblasts | Fibroblasts | AGR2 | -1.88 | 0 |
| Fibroblasts | Fibroblasts | SPINK1 | -1.89 | 0 |
| Fibroblasts | Fibroblasts | TSPAN8 | -1.97 | 0 |
| Fibroblasts | Fibroblasts | KRT19 | -2.03 | 0 |
| Fibroblasts | Fibroblasts | MT-CO2 | -1.39 | 3.20E-305 |
| Fibroblasts | Fibroblasts | VASN | 0.53 | 5.08E-304 |
| Fibroblasts | Fibroblasts | TUBB6 | 0.51 | 6.85E-301 |
| Fibroblasts | Fibroblasts | GYPC | 0.67 | 9.58E-298 |
| Fibroblasts | Fibroblasts | MT-CO1 | -1.26 | 2.05E-295 |
| Fibroblasts | Fibroblasts | CTSL | 0.7 | 3.88E-295 |
| Fibroblasts | Fibroblasts | EGR1 | 1.22 | 4.35E-295 |
| Fibroblasts | Fibroblasts | LAMC1 | 0.64 | 1.22E-294 |
| Fibroblasts | Fibroblasts | EHD2 | 0.49 | 1.69E-290 |
| Fibroblasts | Fibroblasts | MAFB | 0.88 | 1.82E-287 |
| Fibroblasts | Fibroblasts | S100P | -1.66 | 1.61E-282 |
| Fibroblasts | Fibroblasts | MALAT1 | 0.76 | 1.76E-282 |
| Fibroblasts | Fibroblasts | SFN | -1.57 | 9.39E-281 |
| Fibroblasts | Fibroblasts | HAAO | 0.94 | 2.23E-279 |
| Fibroblasts | Fibroblasts | BEX3 | 0.99 | 1.82E-274 |
| Fibroblasts | Fibroblasts | CD63 | 0.83 | 7.44E-274 |
| Fibroblasts | Fibroblasts | SSBP2 | 0.52 | 4.26E-270 |
| Fibroblasts | Fibroblasts | FOSB | 1.11 | 1.37E-269 |
| Fibroblasts | Fibroblasts | GPX8 | 0.37 | 2.23E-267 |
| Fibroblasts | Fibroblasts | PID1 | 0.5 | 1.01E-265 |
| Fibroblasts | Fibroblasts | EZR | -1.16 | 1.27E-264 |
| Fibroblasts | Fibroblasts | AL391121.1 | 0.47 | 1.66E-262 |
| Fibroblasts | Fibroblasts | CLDN18 | -1.58 | 3.23E-261 |
| Fibroblasts | Fibroblasts | MT-ND4 | -1.01 | 2.10E-259 |
| Fibroblasts | Fibroblasts | ANXA5 | 1.14 | 1.04E-258 |
| Fibroblasts | Fibroblasts | STC1 | 1 | 6.11E-255 |
| Fibroblasts | Fibroblasts | ENPP2 | 0.72 | 7.54E-254 |
| Fibroblasts | Fibroblasts | ADH1B | 1.11 | 1.29E-253 |
| Fibroblasts | Fibroblasts | ENG | 0.44 | 1.85E-249 |
| Fibroblasts | Fibroblasts | GEM | 1.46 | 1.95E-248 |
| Fibroblasts | Fibroblasts | B2M | 0.76 | 5.09E-248 |
| Fibroblasts | Fibroblasts | SEMA5A | 0.37 | 1.88E-247 |
| Fibroblasts | Fibroblasts | LGALS4 | -1.66 | 4.02E-247 |
| Fibroblasts | Fibroblasts | CTSE | -1.31 | 2.41E-243 |
| Fibroblasts | Fibroblasts | ZEB1 | 0.4 | 8.94E-242 |
| Fibroblasts | Fibroblasts | ANXA6 | 0.59 | 4.09E-241 |
| Fibroblasts | Fibroblasts | S100A14 | -1.53 | 1.86E-240 |
| Fibroblasts | Fibroblasts | SPINT2 | -1.14 | 6.20E-240 |
| Fibroblasts | Fibroblasts | PIGR | -1.72 | 5.56E-238 |
| Fibroblasts | Fibroblasts | ZFP36L1 | 1.11 | 6.42E-237 |
| Fibroblasts | Fibroblasts | CNN3 | 0.99 | 2.88E-236 |
| Fibroblasts | Fibroblasts | COL4A2 | 0.55 | 5.78E-233 |
| Fibroblasts | Fibroblasts | EPCAM | -1.4 | 1.75E-230 |
| Fibroblasts | Fibroblasts | TNFAIP2 | 0.6 | 1.57E-229 |
| Fibroblasts | Fibroblasts | LAMB1 | 0.52 | 1.68E-228 |
| Fibroblasts | Fibroblasts | DNAJB1 | 1.46 | 1.75E-228 |
| Fibroblasts | Fibroblasts | RBPMS | 0.6 | 1.82E-228 |
| Fibroblasts | Fibroblasts | SSPN | 0.61 | 5.70E-227 |
| Fibroblasts | Fibroblasts | IER2 | 1.15 | 3.12E-226 |
| Fibroblasts | Fibroblasts | DAB2 | 0.45 | 5.09E-226 |
| Fibroblasts | Fibroblasts | GMFG | 0.6 | 2.77E-222 |
| Fibroblasts | Fibroblasts | CLEC3B | 0.35 | 5.83E-221 |
| Fibroblasts | Fibroblasts | HLA-C | 0.75 | 1.60E-217 |
| Fibroblasts | Fibroblasts | GPX2 | -1.27 | 9.54E-217 |
| Fibroblasts | Fibroblasts | CIRBP | 0.86 | 1.16E-215 |
| Fibroblasts | Fibroblasts | TCEAL4 | 0.81 | 9.35E-215 |
| Fibroblasts | Fibroblasts | ITM2B | 0.76 | 4.50E-212 |
| Fibroblasts | Fibroblasts | HLA-B | 0.75 | 2.40E-211 |
| Fibroblasts | Fibroblasts | ZEB2 | 0.38 | 1.01E-209 |
| Fibroblasts | Fibroblasts | SELENOP | 1.01 | 1.08E-209 |
| Fibroblasts | Fibroblasts | ICAM1 | 0.76 | 1.94E-209 |
| Fibroblasts | Fibroblasts | CA2 | -1.42 | 3.22E-209 |
| Fibroblasts | Fibroblasts | RNASE1 | -1.37 | 8.42E-206 |
| Fibroblasts | Fibroblasts | VAMP8 | -1.05 | 1.68E-204 |
| Fibroblasts | Fibroblasts | ARL14 | -1.27 | 1.03E-201 |
| Fibroblasts | Fibroblasts | ADAMTS1 | 0.42 | 6.99E-201 |
| Fibroblasts | Fibroblasts | PHGR1 | -2.25 | 1.13E-200 |
| Fibroblasts | Fibroblasts | TNS2 | 0.35 | 3.86E-200 |
| Fibroblasts | Fibroblasts | VSIG2 | -1.13 | 4.20E-199 |
| Fibroblasts | Fibroblasts | ANXA10 | -1.24 | 4.69E-199 |
| Fibroblasts | Fibroblasts | MAGEH1 | 0.43 | 5.05E-199 |
| Fibroblasts | Fibroblasts | MT-ND3 | -0.89 | 5.92E-198 |
| Fibroblasts | Fibroblasts | CSF1 | 0.28 | 1.16E-196 |
| Fibroblasts | Fibroblasts | RDX | 0.58 | 2.80E-194 |
| Fibroblasts | Fibroblasts | ZC2HC1A | 0.41 | 2.66E-193 |
| Fibroblasts | Fibroblasts | PRNP | 0.77 | 7.00E-191 |
| Fibroblasts | Fibroblasts | MT1G | -1.53 | 4.66E-190 |
| Fibroblasts | Fibroblasts | MUC1 | -1.29 | 6.30E-190 |
| Fibroblasts | Fibroblasts | OCIAD2 | -1.1 | 2.49E-188 |
| Fibroblasts | Fibroblasts | FLNA | 0.6 | 3.35E-188 |
| Fibroblasts | Fibroblasts | ADM | 1.32 | 1.11E-187 |
| Fibroblasts | Fibroblasts | TACSTD2 | -1.23 | 5.32E-185 |
| Fibroblasts | Fibroblasts | JPT1 | -1.08 | 7.57E-185 |
| Fibroblasts | Fibroblasts | CPQ | 0.71 | 9.02E-184 |
| Fibroblasts | Fibroblasts | MT-ND1 | -0.86 | 1.73E-182 |
| Fibroblasts | Fibroblasts | AGR3 | -1.29 | 4.35E-182 |
| Fibroblasts | Fibroblasts | C9orf3 | 0.72 | 2.71E-181 |
| Fibroblasts | Fibroblasts | RAB11FIP1 | -0.99 | 3.78E-181 |
| Fibroblasts | Fibroblasts | IRF1 | 1.22 | 1.47E-180 |
| Fibroblasts | Fibroblasts | TPD52 | -0.99 | 5.62E-180 |
| Fibroblasts | Fibroblasts | MT-ATP6 | -0.99 | 1.97E-179 |
| Fibroblasts | Fibroblasts | EPHX1 | 0.77 | 1.17E-178 |
| Fibroblasts | Fibroblasts | CXCR4 | 0.58 | 6.79E-178 |
| Fibroblasts | Fibroblasts | HOXB2 | 0.43 | 7.32E-178 |
| Fibroblasts | Fibroblasts | PPT1 | 0.67 | 6.73E-177 |
| Fibroblasts | Fibroblasts | LY6E | 0.89 | 2.73E-176 |
| Fibroblasts | Fibroblasts | COL4A1 | 0.32 | 4.64E-176 |
| Fibroblasts | Fibroblasts | SOX4 | 0.92 | 2.01E-175 |
| Fibroblasts | Fibroblasts | HSPA1B | 1.25 | 3.91E-175 |
| Fibroblasts | Fibroblasts | TMEM45A | 0.47 | 1.42E-174 |
| Fibroblasts | Fibroblasts | EIF1 | 0.49 | 2.84E-174 |
| Fibroblasts | Fibroblasts | ARMCX1 | 0.4 | 9.72E-174 |
| Fibroblasts | Fibroblasts | GSTP1 | -0.77 | 2.02E-172 |
| Fibroblasts | Fibroblasts | H3F3B | 0.51 | 1.47E-171 |
| Fibroblasts | Fibroblasts | GRK5 | 0.62 | 2.18E-171 |
| Fibroblasts | Fibroblasts | UBB | 0.54 | 4.21E-171 |
| Fibroblasts | Fibroblasts | IFI16 | 0.57 | 5.34E-171 |
| Fibroblasts | Fibroblasts | RBM47 | -0.94 | 7.03E-170 |
| Fibroblasts | Fibroblasts | PCDH17 | 0.32 | 2.30E-169 |
| Fibroblasts | Fibroblasts | CTGF | 1.11 | 1.41E-168 |
| Fibroblasts | Fibroblasts | HOXA3 | 0.39 | 5.90E-168 |
| Fibroblasts | Fibroblasts | MAL2 | -1.01 | 1.74E-167 |
| Fibroblasts | Fibroblasts | AKR1B10 | -1.26 | 1.78E-167 |
| Fibroblasts | Fibroblasts | SMIM24 | -1.12 | 2.17E-167 |
| Fibroblasts | Fibroblasts | LMO7 | -1.02 | 4.02E-167 |
| Fibroblasts | Fibroblasts | EPB41L2 | 0.67 | 4.84E-166 |
| Fibroblasts | Fibroblasts | TSPAN12 | 0.8 | 6.64E-166 |
| Fibroblasts | Fibroblasts | GNAI1 | 0.47 | 1.32E-165 |
| Fibroblasts | Fibroblasts | ARL4D | 0.39 | 1.36E-164 |
| Fibroblasts | Fibroblasts | SKP1 | 0.66 | 1.64E-164 |
| Fibroblasts | Fibroblasts | TSC22D3 | 0.63 | 3.40E-164 |
| Fibroblasts | Fibroblasts | TSPAN1 | -0.97 | 8.12E-164 |
| Fibroblasts | Fibroblasts | 7-Sep | 0.84 | 3.78E-162 |
| Fibroblasts | Fibroblasts | CAV2 | 0.45 | 1.11E-161 |
| Fibroblasts | Fibroblasts | LMCD1 | 0.56 | 1.42E-159 |
| Fibroblasts | Fibroblasts | HMGA1 | -0.93 | 2.83E-159 |
| Fibroblasts | Fibroblasts | STARD10 | -0.91 | 2.72E-158 |
| Fibroblasts | Fibroblasts | TXN | -0.97 | 5.80E-158 |
| Fibroblasts | Fibroblasts | SLC22A17 | 0.29 | 1.76E-157 |
| Fibroblasts | Fibroblasts | TMEM173 | 0.48 | 4.41E-157 |
| Fibroblasts | Fibroblasts | PGF | 0.35 | 5.04E-157 |
| Fibroblasts | Fibroblasts | ISCU | 0.81 | 2.39E-156 |
| Fibroblasts | Fibroblasts | FLT3LG | 0.28 | 1.66E-155 |
| Fibroblasts | Fibroblasts | PHLDB1 | 0.46 | 5.54E-155 |
| Fibroblasts | Fibroblasts | GPC1 | 0.36 | 1.18E-154 |
| Fibroblasts | Fibroblasts | CD99 | 0.83 | 1.19E-154 |
| Fibroblasts | Fibroblasts | KLF5 | -0.9 | 1.68E-154 |
| Fibroblasts | Fibroblasts | PDLIM1 | 0.95 | 5.74E-153 |
| Fibroblasts | Fibroblasts | ITM2C | 0.69 | 1.05E-152 |
| Fibroblasts | Fibroblasts | PHLDA2 | -0.95 | 1.09E-152 |
| Fibroblasts | Fibroblasts | TMC5 | -0.93 | 2.17E-151 |
| Fibroblasts | Fibroblasts | A1BG | 0.42 | 1.27E-150 |
| Fibroblasts | Fibroblasts | MGST2 | -0.95 | 1.71E-149 |
| Fibroblasts | Fibroblasts | KANK2 | 0.44 | 1.99E-149 |
| Fibroblasts | Fibroblasts | CTSF | 0.46 | 1.04E-148 |
| Fibroblasts | Fibroblasts | RAB31 | 0.34 | 4.93E-148 |
| Fibroblasts | Fibroblasts | STOM | 0.57 | 1.28E-147 |
| Fibroblasts | Fibroblasts | PDLIM4 | 0.53 | 2.24E-147 |
| Fibroblasts | Fibroblasts | ANGPTL4 | 0.59 | 1.38E-146 |
| Fibroblasts | Fibroblasts | PPIB | 0.75 | 2.15E-145 |
| Fibroblasts | Fibroblasts | PLD3 | 0.76 | 2.03E-144 |
| Fibroblasts | Fibroblasts | CYP3A5 | -0.91 | 7.65E-143 |
| Fibroblasts | Fibroblasts | F3 | 1.06 | 7.63E-141 |
| Fibroblasts | Fibroblasts | RBP4 | 0.69 | 2.63E-139 |
| Fibroblasts | Fibroblasts | TWSG1 | 0.45 | 3.66E-139 |
| Fibroblasts | Fibroblasts | CLEC2B | 0.5 | 7.10E-139 |
| Fibroblasts | Fibroblasts | CDH1 | -0.82 | 8.71E-139 |
| Fibroblasts | Fibroblasts | THBS1 | 0.53 | 1.11E-137 |
| Fibroblasts | Fibroblasts | AKR7A3 | -0.92 | 2.01E-137 |
| Fibroblasts | Fibroblasts | LAMB3 | -0.9 | 2.54E-137 |
| Fibroblasts | Fibroblasts | TPST1 | 0.38 | 2.99E-137 |
| Fibroblasts | Fibroblasts | GOLM1 | -0.85 | 3.78E-137 |
| Fibroblasts | Fibroblasts | PQLC3 | 0.59 | 1.57E-136 |
| Fibroblasts | Fibroblasts | PDLIM7 | 0.53 | 2.00E-135 |
| Fibroblasts | Fibroblasts | CLDN4 | -1.58 | 7.59E-135 |
| Fibroblasts | Fibroblasts | PCSK6 | 0.41 | 2.20E-134 |
| Fibroblasts | Fibroblasts | SRP14 | 0.54 | 4.19E-134 |
| Fibroblasts | Fibroblasts | ATP6AP2 | 0.76 | 5.38E-134 |
| Fibroblasts | Fibroblasts | LSR | -0.73 | 9.74E-134 |
| Fibroblasts | Fibroblasts | HMGB1 | 0.56 | 4.24E-133 |
| Fibroblasts | Fibroblasts | CTSB | 0.87 | 1.89E-132 |
| Fibroblasts | Fibroblasts | GPRC5A | -0.87 | 3.89E-132 |
| Fibroblasts | Fibroblasts | FTH1 | 0.44 | 1.36E-131 |
| Fibroblasts | Fibroblasts | ARHGAP6 | 0.31 | 3.33E-131 |
| Fibroblasts | Fibroblasts | MT-CYB | -0.85 | 4.07E-131 |
| Fibroblasts | Fibroblasts | ACTG1 | -0.63 | 9.77E-131 |
| Fibroblasts | Fibroblasts | MRPS6 | 0.77 | 1.73E-130 |
| Fibroblasts | Fibroblasts | SDCBP2 | -0.98 | 1.90E-130 |
| Fibroblasts | Fibroblasts | TUBA1C | -0.78 | 1.00E-129 |
| Fibroblasts | Fibroblasts | NEU1 | 0.57 | 2.27E-129 |
| Fibroblasts | Fibroblasts | TSPO | -0.73 | 4.62E-129 |
| Fibroblasts | Fibroblasts | PDE5A | 0.35 | 1.21E-127 |
| Fibroblasts | Fibroblasts | GLIPR2 | 0.32 | 6.82E-127 |
| Fibroblasts | Fibroblasts | FTL | 0.54 | 4.04E-126 |
| Fibroblasts | Fibroblasts | MAGED2 | 0.73 | 6.49E-126 |
| Fibroblasts | Fibroblasts | MYADM | 0.85 | 1.90E-125 |
| Fibroblasts | Fibroblasts | ITGA6 | -0.75 | 5.67E-125 |
| Fibroblasts | Fibroblasts | MAP1B | 0.44 | 6.83E-125 |
| Fibroblasts | Fibroblasts | TFF1 | -2.05 | 8.86E-125 |
| Fibroblasts | Fibroblasts | SLC44A4 | -0.76 | 9.00E-124 |
| Fibroblasts | Fibroblasts | NFIC | 0.72 | 8.59E-123 |
| Fibroblasts | Fibroblasts | CEBPD | 0.73 | 1.23E-122 |
| Fibroblasts | Fibroblasts | HSD17B2 | 0.87 | 1.27E-122 |
| Fibroblasts | Fibroblasts | MSMB | -1.39 | 3.66E-122 |
| Fibroblasts | Fibroblasts | C12orf75 | -0.85 | 2.45E-121 |
| Fibroblasts | Fibroblasts | PLOD2 | 0.39 | 2.70E-120 |
| Fibroblasts | Fibroblasts | NQO1 | -0.76 | 2.74E-120 |
| Fibroblasts | Fibroblasts | OTULINL | 0.58 | 3.19E-120 |
| Fibroblasts | Fibroblasts | KLF6 | -0.67 | 6.32E-120 |
| Fibroblasts | Fibroblasts | EPHA2 | -0.78 | 4.54E-118 |
| Fibroblasts | Fibroblasts | RTL8C | 0.57 | 5.95E-118 |
| Fibroblasts | Fibroblasts | C6orf48 | 0.8 | 1.96E-117 |
| Fibroblasts | Fibroblasts | DSP | -0.74 | 2.37E-117 |
| Fibroblasts | Fibroblasts | MT-ND2 | -0.72 | 3.93E-117 |
| Fibroblasts | Fibroblasts | PRAF2 | 0.44 | 2.69E-116 |
| Fibroblasts | Fibroblasts | C15orf48 | -1.03 | 2.90E-116 |
| Fibroblasts | Fibroblasts | AKR1C3 | -0.87 | 3.21E-116 |
| Fibroblasts | Fibroblasts | TBXAS1 | 0.4 | 1.62E-115 |
| Fibroblasts | Fibroblasts | GMDS | -0.68 | 1.02E-114 |
| Fibroblasts | Fibroblasts | VKORC1 | 0.7 | 1.06E-114 |
| Fibroblasts | Fibroblasts | CARD16 | 0.8 | 1.47E-114 |
| Fibroblasts | Fibroblasts | EMP2 | 0.75 | 4.15E-114 |
| Fibroblasts | Fibroblasts | MISP | -0.79 | 4.43E-114 |
| Fibroblasts | Fibroblasts | CD302 | 0.55 | 4.23E-113 |
| Fibroblasts | Fibroblasts | APOC1 | 0.61 | 6.77E-113 |
| Fibroblasts | Fibroblasts | ZSCAN18 | 0.32 | 1.83E-112 |
| Fibroblasts | Fibroblasts | DPYSL2 | 0.5 | 3.96E-112 |
| Fibroblasts | Fibroblasts | F10 | 0.3 | 5.69E-112 |
| Fibroblasts | Fibroblasts | PROS1 | 0.33 | 1.90E-111 |
| Fibroblasts | Fibroblasts | MT-ND5 | -0.8 | 7.99E-111 |
| Fibroblasts | Fibroblasts | C12orf57 | 0.68 | 1.45E-110 |
| Fibroblasts | Fibroblasts | HSPB1 | 0.57 | 1.48E-110 |
| Fibroblasts | Fibroblasts | SERPINB1 | -0.69 | 2.75E-110 |
| Fibroblasts | Fibroblasts | SULT1C2 | -0.76 | 2.82E-110 |
| Fibroblasts | Fibroblasts | TFF2 | -1.67 | 9.95E-110 |
| Fibroblasts | Fibroblasts | ZBTB20 | 0.49 | 1.18E-109 |
| Fibroblasts | Fibroblasts | BSG | 0.65 | 3.04E-109 |
| Fibroblasts | Fibroblasts | TMEM54 | -0.81 | 9.92E-109 |
| Fibroblasts | Fibroblasts | COX4I1 | -0.48 | 3.38E-108 |
| Fibroblasts | Fibroblasts | MUC5AC | -1.52 | 3.63E-108 |
| Fibroblasts | Fibroblasts | ITGB1 | 0.77 | 4.15E-108 |
| Fibroblasts | Fibroblasts | NR3C1 | 0.51 | 2.66E-107 |
| Fibroblasts | Fibroblasts | TCEAL9 | 0.64 | 1.97E-106 |
| Fibroblasts | Fibroblasts | PPP1R15A | 0.78 | 2.17E-105 |
| Fibroblasts | Fibroblasts | ACTN4 | -0.69 | 3.44E-105 |
| Fibroblasts | Fibroblasts | SVIP | -0.67 | 8.71E-105 |
| Fibroblasts | Fibroblasts | PIP4P2 | 0.31 | 9.70E-105 |
| Fibroblasts | Fibroblasts | FSCN1 | 0.33 | 1.33E-104 |
| Fibroblasts | Fibroblasts | TM9SF3 | -0.63 | 2.98E-104 |
| Fibroblasts | Fibroblasts | ATRAID | 0.72 | 1.49E-103 |
| Fibroblasts | Fibroblasts | IKBIP | 0.3 | 2.98E-103 |
| Fibroblasts | Fibroblasts | CXADR | -0.65 | 1.21E-102 |
| Fibroblasts | Fibroblasts | PPDPF | -0.66 | 2.22E-102 |
| Fibroblasts | Fibroblasts | IFI27L2 | 0.69 | 5.03E-102 |
| Fibroblasts | Fibroblasts | CLDN7 | -0.99 | 6.29E-102 |
| Fibroblasts | Fibroblasts | UQCRH | -0.61 | 1.10E-101 |
| Fibroblasts | Fibroblasts | FOXQ1 | -0.87 | 4.34E-101 |
| Fibroblasts | Fibroblasts | KLF2 | -0.88 | 6.43E-101 |
| Fibroblasts | Fibroblasts | S100A16 | -0.81 | 2.26E-100 |
| Fibroblasts | Fibroblasts | LIPH | -0.71 | 2.57E-100 |
| Fibroblasts | Fibroblasts | LIMS1 | 0.63 | 1.74E-99 |
| Fibroblasts | Fibroblasts | PHLDA1 | 0.96 | 2.72E-99 |
| Fibroblasts | Fibroblasts | PRR13 | -0.7 | 2.83E-99 |
| Fibroblasts | Fibroblasts | TSTD1 | -0.59 | 5.29E-99 |
| Fibroblasts | Fibroblasts | NCALD | 0.35 | 1.19E-98 |
| Fibroblasts | Fibroblasts | SPINT1 | -0.64 | 1.61E-98 |
| Fibroblasts | Fibroblasts | C5orf56 | 0.41 | 1.78E-98 |
| Fibroblasts | Fibroblasts | HPGD | -0.87 | 2.57E-98 |
| Fibroblasts | Fibroblasts | C1orf21 | 0.71 | 6.15E-98 |
| Fibroblasts | Fibroblasts | ATP8B1 | -0.64 | 8.51E-98 |
| Fibroblasts | Fibroblasts | SH3BGRL3 | -0.65 | 1.13E-97 |
| Fibroblasts | Fibroblasts | TCIM | 1.1 | 2.77E-97 |
| Fibroblasts | Fibroblasts | REG4 | -2.21 | 4.32E-97 |
| Fibroblasts | Fibroblasts | PLAC8 | -0.87 | 5.32E-97 |
| Fibroblasts | Fibroblasts | TESC | -0.8 | 6.34E-97 |
| Fibroblasts | Fibroblasts | CD55 | -0.72 | 7.33E-97 |
| Fibroblasts | Fibroblasts | APLP2 | 0.81 | 1.08E-96 |
| Fibroblasts | Fibroblasts | HLA-A | 0.52 | 1.09E-96 |
| Fibroblasts | Fibroblasts | F2RL1 | -0.69 | 1.16E-96 |
| Fibroblasts | Fibroblasts | HSP90AA1 | 0.59 | 2.29E-96 |
| Fibroblasts | Fibroblasts | FABP5 | -0.94 | 3.90E-96 |
| Fibroblasts | Fibroblasts | SLPI | -0.79 | 1.24E-95 |
| Fibroblasts | Fibroblasts | SEC62 | 0.65 | 2.37E-95 |
| Fibroblasts | Fibroblasts | CXCL17 | -0.88 | 7.06E-94 |
| Fibroblasts | Fibroblasts | YWHAZ | -0.6 | 7.75E-94 |
| Fibroblasts | Fibroblasts | LDHB | 0.61 | 2.59E-92 |
| Fibroblasts | Fibroblasts | SH3BGRL | 0.68 | 8.38E-92 |
| Fibroblasts | Fibroblasts | LEPROT | 0.64 | 1.03E-91 |
| Fibroblasts | Fibroblasts | ARF6 | -0.65 | 1.30E-91 |
| Fibroblasts | Fibroblasts | SEMA4D | 0.33 | 3.04E-91 |
| Fibroblasts | Fibroblasts | TPM3 | -0.59 | 4.07E-91 |
| Fibroblasts | Fibroblasts | BEX4 | 0.36 | 1.01E-90 |
| Fibroblasts | Fibroblasts | ST14 | -0.61 | 6.00E-90 |
| Fibroblasts | Fibroblasts | PTMS | 0.69 | 7.60E-90 |
| Fibroblasts | Fibroblasts | SERPINE2 | 0.47 | 1.05E-89 |
| Fibroblasts | Fibroblasts | KCNK1 | -0.6 | 1.61E-89 |
| Fibroblasts | Fibroblasts | FAM3B | -0.6 | 2.74E-89 |
| Fibroblasts | Fibroblasts | DSTN | 0.57 | 2.88E-89 |
| Fibroblasts | Fibroblasts | B3GNT5 | -0.63 | 3.95E-89 |
| Fibroblasts | Fibroblasts | EFHD2 | -0.65 | 4.33E-89 |
| Fibroblasts | Fibroblasts | SERINC2 | -0.58 | 4.60E-89 |
| Fibroblasts | Fibroblasts | HSPA2 | 0.51 | 5.08E-89 |
| Fibroblasts | Fibroblasts | LDLRAD4 | 0.3 | 5.11E-89 |
| Fibroblasts | Fibroblasts | TCTN1 | 0.32 | 5.44E-89 |
| Fibroblasts | Fibroblasts | MORF4L1 | 0.59 | 5.64E-89 |
| Fibroblasts | Fibroblasts | SNAI1 | 0.37 | 3.50E-88 |
| Fibroblasts | Fibroblasts | PTPRE | 0.47 | 3.59E-88 |
| Fibroblasts | Fibroblasts | TYMP | 0.5 | 1.43E-87 |
| Fibroblasts | Fibroblasts | UQCR10 | -0.59 | 1.64E-87 |
| Fibroblasts | Fibroblasts | CYB5R3 | 0.66 | 2.00E-87 |
| Fibroblasts | Fibroblasts | SEMA4A | 0.45 | 2.09E-87 |
| Fibroblasts | Fibroblasts | RPS3 | -0.44 | 3.67E-87 |
| Fibroblasts | Fibroblasts | ISL1 | 0.34 | 1.65E-86 |
| Fibroblasts | Fibroblasts | LRRFIP1 | -0.56 | 2.72E-86 |
| Fibroblasts | Fibroblasts | TMPRSS2 | -0.59 | 4.86E-86 |
| Fibroblasts | Fibroblasts | LRP1 | 0.62 | 6.67E-86 |
| Fibroblasts | Fibroblasts | GSTA1 | -0.89 | 1.04E-85 |
| Fibroblasts | Fibroblasts | CBX6 | 0.42 | 1.18E-85 |
| Fibroblasts | Fibroblasts | TM4SF5 | -0.62 | 1.31E-85 |
| Fibroblasts | Fibroblasts | UBC | 0.51 | 1.45E-85 |
| Fibroblasts | Fibroblasts | ADH5 | 0.65 | 1.85E-85 |
| Fibroblasts | Fibroblasts | PERP | -0.57 | 3.49E-85 |
| Fibroblasts | Fibroblasts | FAM229B | 0.3 | 6.40E-85 |
| Fibroblasts | Fibroblasts | NAP1L1 | 0.63 | 8.20E-85 |
| Fibroblasts | Fibroblasts | SLC25A5 | -0.73 | 1.04E-84 |
| Fibroblasts | Fibroblasts | ANTXR2 | 0.37 | 1.21E-84 |
| Fibroblasts | Fibroblasts | CD74 | -1 | 1.26E-84 |
| Fibroblasts | Fibroblasts | REXO2 | 0.63 | 2.78E-84 |
| Fibroblasts | Fibroblasts | ALDH1A1 | -0.62 | 3.12E-84 |
| Fibroblasts | Fibroblasts | TFF3 | -1.93 | 3.53E-84 |
| Fibroblasts | Fibroblasts | ABHD2 | -0.58 | 4.65E-84 |
| Fibroblasts | Fibroblasts | CAPN8 | -0.65 | 1.61E-83 |
| Fibroblasts | Fibroblasts | PDE4C | -0.69 | 1.93E-83 |
| Fibroblasts | Fibroblasts | PRSS8 | -0.54 | 2.52E-83 |
| Fibroblasts | Fibroblasts | MARCKSL1 | -0.63 | 2.94E-83 |
| Fibroblasts | Fibroblasts | LINC01133 | -0.78 | 4.58E-83 |
| Fibroblasts | Fibroblasts | HMGN2 | 0.46 | 5.86E-83 |
| Fibroblasts | Fibroblasts | RPS18 | -0.44 | 1.32E-82 |
| Fibroblasts | Fibroblasts | INAFM1 | 0.36 | 1.32E-82 |
| Fibroblasts | Fibroblasts | RAB25 | -0.49 | 2.69E-82 |
| Fibroblasts | Fibroblasts | CD40 | 0.37 | 5.59E-82 |
| Fibroblasts | Fibroblasts | CES2 | -0.84 | 2.31E-81 |
| Fibroblasts | Fibroblasts | SDC4 | -0.62 | 1.13E-80 |
| Fibroblasts | Fibroblasts | COX5A | -0.54 | 1.83E-80 |
| Fibroblasts | Fibroblasts | BIK | -0.54 | 2.80E-80 |
| Fibroblasts | Fibroblasts | RPL8 | -0.38 | 3.27E-80 |
| Fibroblasts | Fibroblasts | C6orf132 | -0.5 | 3.91E-80 |
| Fibroblasts | Fibroblasts | RPS19 | -0.46 | 4.92E-80 |
| Fibroblasts | Fibroblasts | MYH14 | -0.58 | 7.88E-80 |
| Fibroblasts | Fibroblasts | RPL36 | -0.43 | 9.29E-80 |
| Fibroblasts | Fibroblasts | RCN1 | 0.61 | 1.28E-79 |
| Fibroblasts | Fibroblasts | FGFR4 | 0.37 | 1.70E-79 |
| Fibroblasts | Fibroblasts | DSG2 | -0.53 | 3.92E-79 |
| Fibroblasts | Fibroblasts | COX5B | -0.51 | 4.00E-79 |
| Fibroblasts | Fibroblasts | IL15RA | 0.33 | 7.19E-79 |
| Fibroblasts | Fibroblasts | CLDN3 | -0.84 | 1.01E-78 |
| Fibroblasts | Fibroblasts | SLC39A13 | 0.33 | 4.16E-78 |
| Fibroblasts | Fibroblasts | PFDN5 | 0.4 | 4.74E-78 |
| Fibroblasts | Fibroblasts | SGMS2 | -0.59 | 6.56E-78 |
| Fibroblasts | Fibroblasts | CXCL3 | -0.95 | 7.63E-78 |
| Fibroblasts | Fibroblasts | DEGS1 | 0.5 | 8.16E-78 |
| Fibroblasts | Fibroblasts | PLPP2 | -0.47 | 1.39E-77 |
| Fibroblasts | Fibroblasts | CYB5A | -0.52 | 1.45E-77 |
| Fibroblasts | Fibroblasts | LAD1 | -0.49 | 1.46E-77 |
| Fibroblasts | Fibroblasts | SOD2 | 1.11 | 2.43E-77 |
| Fibroblasts | Fibroblasts | PRSS3 | -0.44 | 2.94E-77 |
| Fibroblasts | Fibroblasts | FAM20C | 0.32 | 3.87E-77 |
| Fibroblasts | Fibroblasts | PLS1 | -0.57 | 5.06E-77 |
| Fibroblasts | Fibroblasts | CDKN1C | 0.45 | 5.15E-77 |
| Fibroblasts | Fibroblasts | PARD6B | -0.61 | 8.24E-77 |
| Fibroblasts | Fibroblasts | ACTN1 | 0.59 | 1.02E-76 |
| Fibroblasts | Fibroblasts | MT1X | -0.86 | 1.47E-76 |
| Fibroblasts | Fibroblasts | COTL1 | -0.57 | 1.79E-76 |
| Fibroblasts | Fibroblasts | GINM1 | 0.49 | 1.94E-76 |
| Fibroblasts | Fibroblasts | TSPAN13 | -0.52 | 2.05E-76 |
| Fibroblasts | Fibroblasts | PLEKHJ1 | -0.5 | 2.46E-76 |
| Fibroblasts | Fibroblasts | FBP1 | -0.5 | 4.19E-76 |
| Fibroblasts | Fibroblasts | SGCB | 0.46 | 6.54E-76 |
| Fibroblasts | Fibroblasts | HLA-DRA | -1 | 6.62E-76 |
| Fibroblasts | Fibroblasts | MXD1 | -0.6 | 1.41E-75 |
| Fibroblasts | Fibroblasts | AP1M2 | -0.45 | 2.25E-75 |
| Fibroblasts | Fibroblasts | TSTA3 | -0.5 | 2.59E-75 |
| Fibroblasts | Fibroblasts | MT1E | -0.76 | 3.06E-75 |
| Fibroblasts | Fibroblasts | COX8A | -0.5 | 4.65E-75 |
| Fibroblasts | Fibroblasts | GALNT3 | -0.48 | 1.67E-74 |
| Fibroblasts | Fibroblasts | PLA2G10 | -0.59 | 4.91E-74 |
| Fibroblasts | Fibroblasts | IL1RN | -0.64 | 1.37E-73 |
| Fibroblasts | Fibroblasts | SYNGR2 | -0.48 | 1.85E-73 |
| Fibroblasts | Fibroblasts | LLGL2 | -0.48 | 2.61E-73 |
| Fibroblasts | Fibroblasts | ABLIM1 | -0.56 | 5.07E-73 |
| Fibroblasts | Fibroblasts | NET1 | -0.49 | 9.04E-73 |
| Fibroblasts | Fibroblasts | ACSL4 | 0.41 | 1.31E-72 |
| Fibroblasts | Fibroblasts | RAP2B | -0.62 | 1.71E-72 |
| Fibroblasts | Fibroblasts | ALDH3A1 | -0.72 | 1.84E-72 |
| Fibroblasts | Fibroblasts | PKP3 | -0.54 | 2.09E-72 |
| Fibroblasts | Fibroblasts | ANXA1 | 0.52 | 2.38E-72 |
| Fibroblasts | Fibroblasts | S100A6 | -0.69 | 4.13E-72 |
| Fibroblasts | Fibroblasts | IDH1 | -0.5 | 8.87E-72 |
| Fibroblasts | Fibroblasts | SNX3 | 0.54 | 1.17E-71 |
| Fibroblasts | Fibroblasts | MUC13 | -0.73 | 1.38E-71 |
| Fibroblasts | Fibroblasts | SYNGR1 | 0.29 | 1.76E-71 |
| Fibroblasts | Fibroblasts | PSCA | -1.29 | 2.20E-71 |
| Fibroblasts | Fibroblasts | STXBP2 | -0.47 | 2.45E-71 |
| Fibroblasts | Fibroblasts | CAPG | -0.55 | 2.56E-71 |
| Fibroblasts | Fibroblasts | CRACR2B | -0.47 | 2.92E-71 |
| Fibroblasts | Fibroblasts | FAM102A | -0.54 | 5.88E-71 |
| Fibroblasts | Fibroblasts | GALE | -0.49 | 1.34E-70 |
| Fibroblasts | Fibroblasts | WARS | 0.49 | 3.82E-70 |
| Fibroblasts | Fibroblasts | TMEM59 | 0.43 | 4.32E-70 |
| Fibroblasts | Fibroblasts | CRB3 | -0.43 | 8.03E-70 |
[truncated: 755,417 more chars]
