# Supplementary material for: Development of a CD8+ T cell associated signature for predicting the prognosis and immunological characteristics of gastric cancer by integrating single-cell and bulk RNA-sequencing
Source: Sci Rep. 2024 Feb 24;14:4524. doi: 10.1038/s41598-024-54273-9 (PMC10894294; doi:10.1038/s41598-024-54273-9)
Supplement: Supplementary file 4 — Supplementary Legends. [file 41598_2024_54273_MOESM4_ESM.docx]

Supplementary Figure 1. ROC analysis of the performance of TMB, TIDE, MSI, and IPS scores in predicting prognosis.
